# Linking Hydroclimate to Fish Phenology and Habitat Use with Ichthyographs

**DOI:** 10.1371/journal.pone.0168831

**Published:** 2016-12-22

**Authors:** Rebecca L. Flitcroft, Sarah L. Lewis, Ivan Arismendi, Rachel LovellFord, Mary V. Santelmann, Mohammad Safeeq, Gordon Grant

**Affiliations:** 1 Pacific Northwest Research Station, USDA Forest Service, Corvallis, Oregon, United States of America; 2 College of Earth, Ocean, and Atmospheric Sciences, Oregon State University, Corvallis, Oregon, United States of America; 3 Department of Fisheries and Wildlife, Oregon State University, Corvallis, Oregon, United States of America; 4 Water Resources Department: State of Oregon, Salem, Oregon, United States of America; 5 Geosciences Department, University of California at Merced, Merced, California, United States of America; Aberystwyth University, UNITED KINGDOM

## Abstract

Streamflow and water temperature (hydroclimate) influence the life histories of aquatic biota. The relationship between streamflow and temperature varies with climate, hydrogeomorphic setting, and season. Life histories of native fishes reflect, in part, their adaptation to regional hydroclimate (flow and water temperature), local habitats, and natural disturbance regimes, all of which may be affected by water management. Alterations to natural hydroclimates, such as those caused by river regulation or climate change, can modify the suitability and variety of in-stream habitat for fishes throughout the year. Here, we present the *ichthyograph*, a new empirically-based graphical tool to help visualize relationships between hydroclimate and fish phenology. Generally, this graphical tool can be used to display a variety of phenotypic traits. We used long-term data sets of daily fish passage to examine linkages between hydroclimate and the expression of life-history phenology by native fishes. The ichthyograph may be used to characterize the environmental phenology for fishes across multiple spatio-temporal domains. We illustrate the ichthyograph in two applications to visualize: 1) river use for the community of fishes at a specific location; and 2) stream conditions at multiple locations within the river network for one species at different life-history stages. The novel, yet simple, ichthyograph offers a flexible framework to enable transformations in thinking regarding relationships between hydroclimate and aquatic species across space and time. The potential broad application of this innovative tool promotes synergism between assessments of physical characteristics and the biological needs of aquatic species.

## Introduction

In riverine systems, the magnitude and timing of streamflow and related attributes create the conditions in which aquatic biota can develop adaptations and diverse responses to the environment [1]. Streamflow and water temperature (hereafter hydroclimate) vary throughout the year in response to regional climate and the hydrogeomorphic setting of the stream. Historically consistent patterns of seasonal hydroclimate select for traits that enhance fitness among individuals and populations. In particular, consistent seasonal patterns lead to the development of phenological life-history patterns in aquatic species that reflect the linkage between environmental conditions and specific life stages or life-history characteristics [2]. Changes in regional climate patterns and human activities are altering streams [3] in ways that alter the time frame during which conditions appropriate for key life stages of fish occur. Understanding the range of variability in fish response to hydrologic conditions at different points in their life history, and the conditions they require at key life-history stages (such as spawning or juvenile emergence) may be critically important in helping biologists and managers mitigate the effects of future climate change or management actions on aquatic systems.

In the Pacific Northwest (PNW) of North America, as in other parts of the world, consistent patterns of regional climate and hydroclimate are interspersed with unpredictable episodic disturbances. (Naturally occurring disturbances such as volcanic eruptions, wildfire, extreme floods, windthrow and landslides, can both compromise aquatic habitats in the short term, and simultaneously enrich them over the long term [4]. Consequently, across a landscape or region, hydroclimate and disturbance interact to determine spatiotemporal variation in the physiochemical conditions of riverine habitat as a function of time since disturbance.

Many native salmonids of the PNW have anadromous life histories that are adapted to the relatively consistent seasonal patterns of hydroclimate, but with sufficient behavioral diversity to persist in the face of natural hydroclimate variation and disturbance regimes. Their phenology reflects the dynamic nature of stream habitat conditions in the region in both space and time. Species with broad behavioral diversity, such as Pacific salmon [5], have been observed to shift the timing of specific life stage events such as the spawning run in response to modifications in the hydrologic regime [6] or fisheries management [7].

**Fig 1:**
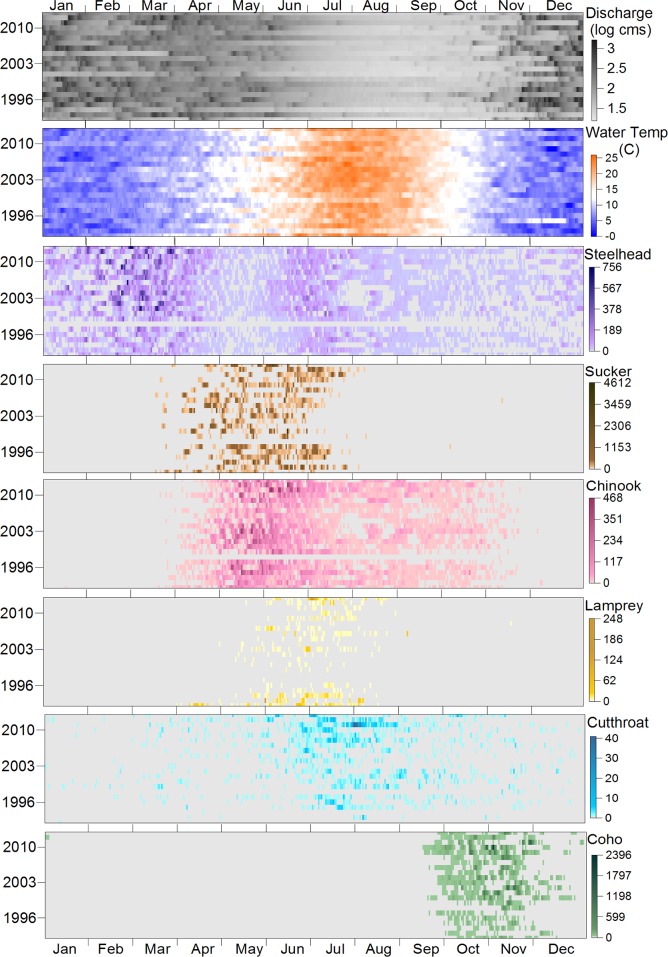
Daily streamflow, water temperature and fish counts for Winchester Dam, North Umpqua River, Oregon, USA from 1992 to 2013. Streamflow (top graph) from USGS gage station No. 14319500. Stream temperature (second graph from top) and fish counts courtesy Oregon Department of Fish and Wildlife for, in order: steelhead (anadromous *Oncorhynchus mykiss*), sucker (*Catostomus macrocheilus*), Chinook Salmon (*Oncorhynchus tshawytscha*), lamprey (*Entosphenus tridentatus*), cutthroat trout (*Oncorhynchus clarkii*), and Coho Salmon (*Oncorhynchus kisutch*). Fish count data unavailable for Jan–Oct 1998. This figure shows a multi-year timeline plot of environmental conditions (daily streamflow and water temperature) and the community of fishes moving upstream past Winchester Dam. Darker colors are associated with higher numbers and show strong seasonal patterns over time for all species. Some species have narrow upstream migration windows (i.e. Coho Salmon) while others move upstream during a wider time window (i.e. steelhead).

The annual progression of the climate cycle creates a shifting suite of hydroclimate and habitats at the same location in a stream over the course of a year. For example, a reach that is a pool in summer may become part of a large rapid under winter flood [8]. Different species are adapted to use different habitats at different locations at different times. For example, upstream fish movement at Winchester Dam, Oregon, USA, illustrates distinct patterns of use by species throughout the year (Fig 1). The partitioning of movement among species appears to coincide with different seasonal hydroclimate conditions. The range of conditions observed for species-specific migration reflects diverse or constrained life-history portfolios that may ultimately be linked to long-term population-scale resilience [9–11].

Rarely are aquatic species and hydroclimate measured at the same location for decades as they have been at Winchester Dam. Such unusually rich information may provide insight into the way that phenology is expressed as individual species or communities of fishes move through watersheds. Here, we use an extensive empirical data set to explore some relationships between hydroclimate and fish phenology [12], effectively identifying the timing of fish use of the river (the ichthyograph).

We illustrate the relationships observed between upstream fish movement and hydroclimate at Winchester Dam. For anadromous salmonids, upstream migration is associated with the spawning life-stage. At Winchester Dam, therefore, we can examine variability and predictability of spawning migration phenology. We first investigate and characterize species-specific patterns of movement revealed by twenty years of data on daily streamflow, stream temperature, and fish counts. Then, we use these general patterns to develop an ichthyograph for the upstream migration of six native fish species at a mid-river location. Finally, we present conceptual ichthyographs for one of these species, Coho Salmon (*Oncorhynchus kisutch*), a native, anadromous, and threatened salmonid [13], for multiple life stages and locations in an entire watershed.

## Methods and Materials

### Study location

**Fig 2:**
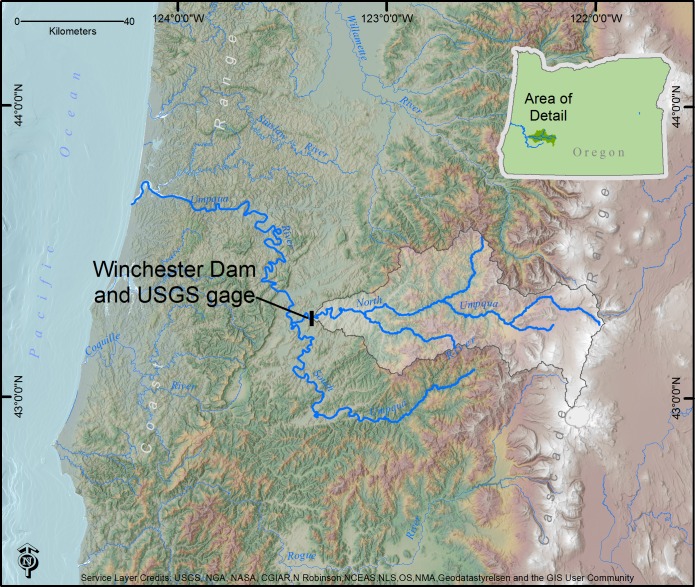
Location of Winchester Dam, OR, and upstream drainage basin. Winchester Dam was built in 1890 and upgraded in 1907 and now includes a timber-crib structure that is 4.9 m in height. While the dam does impound a shallow upstream reservoir, it is considered a “run-of-river” dam. A fish ladder allowing fish passage was installed in 1945 with a viewing window to monitor the upstream passage of all fishes past the dam. Continuously collected fish passage data at this location from 1992 and 2013 was used to develop the Winchester Dam ichthyograph.

Winchester Dam, is located at river kilometer 190 on the North Umpqua River, Oregon, USA (Fig 2). We describe the North Umpqua at Winchester Dam as a “mid-river” location because it is located above the main confluence of the North and South Umpqua (forming the mainstem Umpqua River), yet is too large (approximately 115 m wide with mean annual discharge of 105 m^3^s^-1^) to be considered an upper river location. The North Umpqua River above Winchester Dam (drainage area 3,500 km^2^) has its source in the porous, Quaternary andesitic and basaltic lavas of the High Cascades geologic province and then flows through the highly-dissected Tertiary volcanic formations of the Western Cascade Range. Precipitation on the North Umpqua is complicated with a snow-dominated regime characterizing the High Cascades and rain-dominated characterizing the Western Cascade Range. Downstream of Winchester Dam, the north and south forks of the Umpqua River join, and the mainstem Umpqua River flows through Eocene sandstones of the Coast Range to the Pacific Ocean [14]. In the maritime climate of the PNW, with cool, rainy winters and warm, dry summers, more than 75% of precipitation falls between November and March [15].

Winchester Dam was built in 1890 as a 1.2-m-high timber-crib structure, which was upgraded and raised to 4.9 m in 1907. The dam contained power-producing turbines until 1923, and currently provides recreational opportunities in the impounded reservoir. The dam is operated as a “run-of-river” dam, with no significant flow diversions upstream. A fish ladder allowing passage has been maintained at Winchester Dam since 1945.

### Data sets

Daily average streamflow data for the North Umpqua River at Winchester Dam from 1992–2013 were downloaded from the United States Geological Survey [16]. Census counts for fish migrating upstream during the same period (1992–2013) were collected by the Oregon Department of Fish and Wildlife using video tape equipment in a standard viewing window of the Winchester Dam fish ladder (the fish ladder is owned by the State of Oregon and operated by the Oregon Department of Fish and Wildlife) (S1 Dataset). Water temperature was recorded when an individual fish swam past the fish-viewing window and/or every six hours, and daily average temperature was calculated from these readings. Fish were not handled for the data collection, therefore a permit (including IACUC) was not required. Fish detection and temperature readings were occasionally limited by mechanical failure of the video equipment or high water turbidity that clouds the window (less than 1 day a year on average). Fish count data were unavailable for January through October of 1998 due to technical issues.

## Results

At Winchester Dam, a twenty-year record of daily streamflow, water temperature, and counts of fish movement up the fish ladder show that species movement upstream is clearly associated with temporal variation in hydroclimate at the study site (Fig 1). For example, LovellFord et al. [12] found in a regression analysis of data from Winchester Dam that Coho Salmon mid-river migration was initiated when water temperature dropped to18 degrees. As is typical of coastal river systems of the PNW, streamflow in the North Umpqua River increases and water temperatures cool with the onset of fall precipitation, then fluctuate through the winter and spring with each storm (Fig 1A and 1B). As winter rains end and summer air temperatures warm, streamflow decreases and water temperatures rise. While the date of the first fall storm, the highest water temperature of the summer, or the peak of fish migration varies from year to year, general patterns in the timing of fish response emerge from this data set. We recognize that fish passage at Winchester Dam is most likely driven by more than the environmental conditions on the day the fish move passed the dam, particularly for salmon for whom the dam is part of their spawning migration run. However, we have displayed discharge and temperature with fish passage on each day to best represent local environmental conditions at the time of fish movement.

Upstream fish movement past Winchester Dam (Fig 1C–1H) follows a predictable seasonal pattern. Winter steelhead (anadromous *Oncorhynchus mykiss*) passed Winchester Dam during high winter flows beginning in December. Spring movement upstream of the resident largescale sucker (*Catostomus macrocheilus*) is coincident with the initiation of spring Chinook Salmon (*Oncorhynchus tshawytscha*) migration, while lamprey (*Entosphenus tridentatus*) and summer steelhead (*O*. *mykiss*) begin migrating in early summer. Resident cutthroat trout (*Oncorhynchus clarkii*) migration upstream occurs over a longer time period, with a concentration in early summer coincident with warming of the lower river. Coho Salmon move upstream in a concentrated pulse in the fall as high stream temperatures associated with late-summer low flows begin to cool [12].

### The ichthyograph

**Fig 3:**
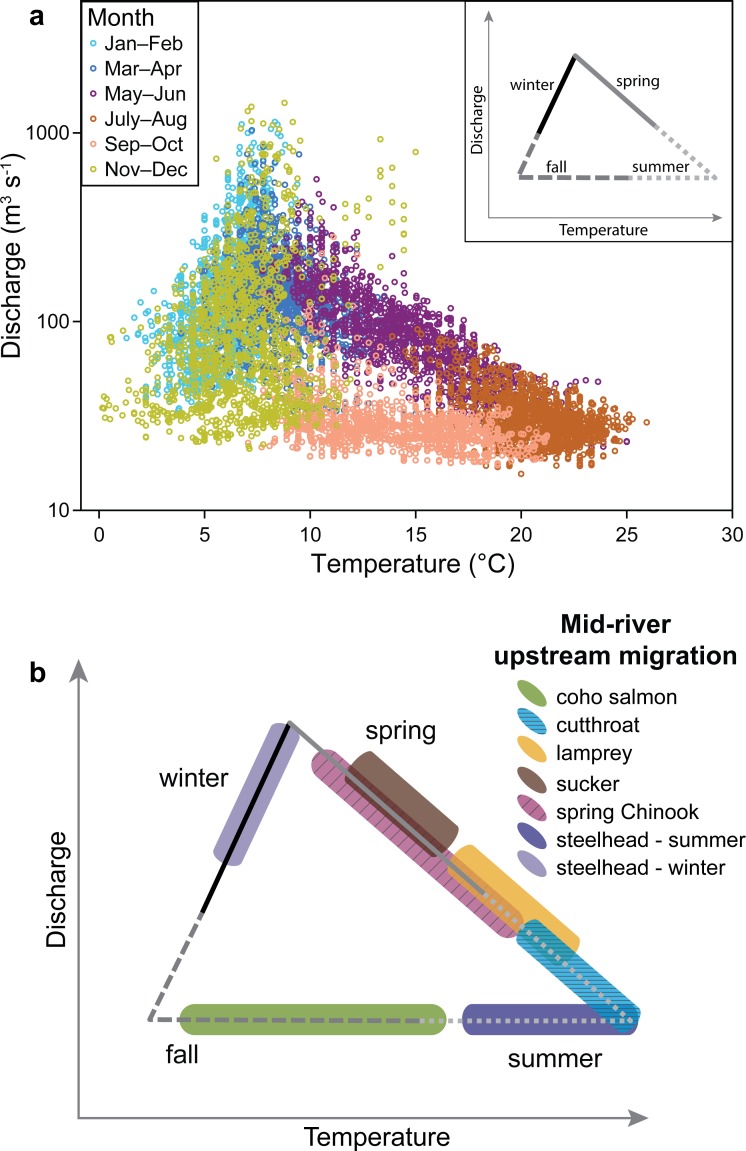
Streamflow and stream temperature related to fish passage timing at Winchester Dam, OR. Multiple years of streamflow and stream temperature, when plotted against one another on a graph, may show a cyclical pattern. Such is the case at Winchester Dam, OR when streamflow and stream temperature for the period of record (1992–2103) is plotted as: (a) average daily values; inset) generalized seasonal relationship creating an annual cycle of hydrologic conditions. When fish passage is overlaid on the framework of discharge and temperature, an ichthyograph is created: (b) ichthyograph of daily fish use at Winchester Dam based on data from Fig 1. Various other phenological traits could be plotted in this way, with ideal data based on empirical observation, as is the case at Winchester Dam, OR.

The relationship between streamflow and water temperature for the North Umpqua River (Fig 3A) demonstrates annual hysteresis [12]. Winter storms (Nov–Apr) generate the highest flows. As flow declines into late spring and summer, water temperatures increase. Baseflow conditions begin in mid-summer and continue through fall as water temperature decreases. The loop closes with the onset of winter rain events. We generalize this relationship as a simplified triangle of seasonal hydrologic conditions for this river system (Fig 3A inset).

To create an ichthyograph at a particular location, data regarding fish use of the river is used to code each daily point in the streamflow and water temperature space for the period of record. At Winchester Dam, the seasonal patterns and associated hydroclimate for upstream migration patterns of six native fish species (as shown in Fig 1) can be summarized in a single ichthyograph (Fig 3B). Further, we can identify potential emerging hazards for these fishes at specific life stages. For example, the highest water temperatures occur during the lowest flows in July and August, creating a potential “bottleneck” of physiologically stressful conditions at Winchester Dam for cold-water species such as coastal cutthroat trout, steelhead, and Chinook Salmon.

**Fig 4:**
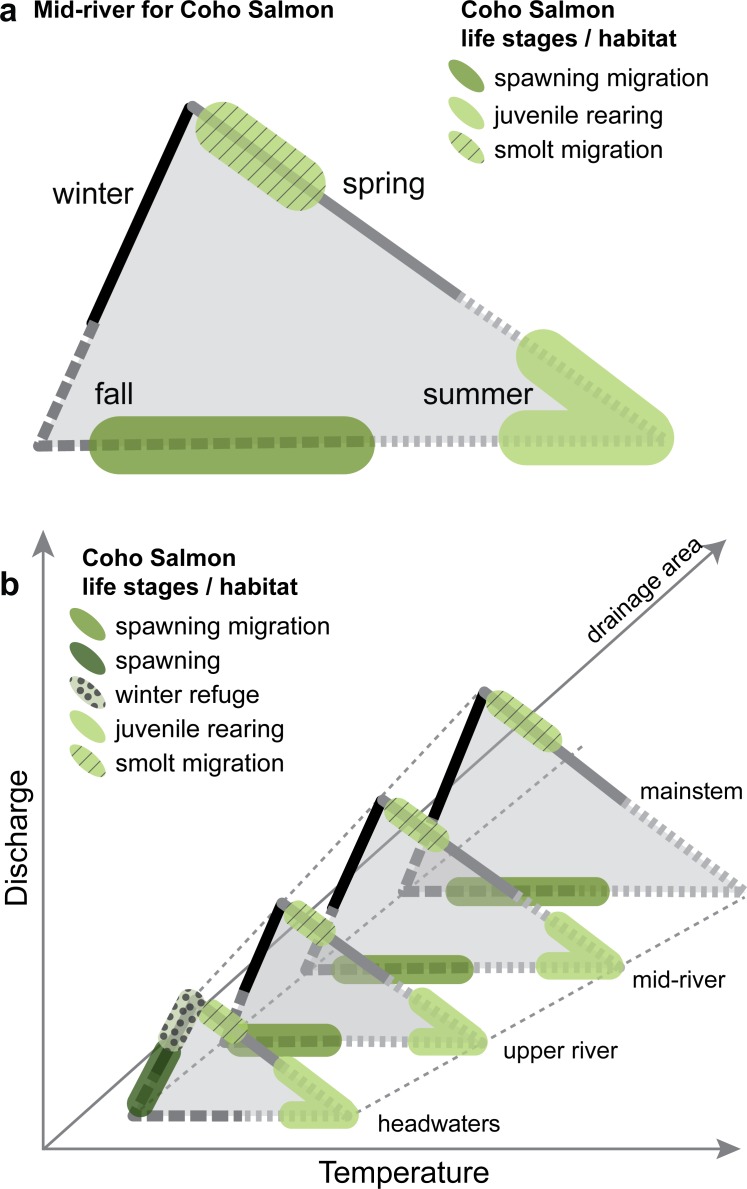
Conceptual ichthyographs for Coho Salmon. Conceptual ichthyographs for Coho Salmon use by life stage of: (a) a mid-river location such as Winchester dam, and (b) throughout the river network with generalized patterns of streamflow and stream temperature for different drainage areas. These conceptual ichthyographs are based on the empirical data available in this system (Fig 1), but also incorporate informal data collected as part of ongoing fish management in this system, and the description of life-stage specific habitat characteristics that can be taken from the peer reviewed literature. Other species specific traits could be mapped in this way, as could other interpretations of fish habitat use beyond specific life stages. Empirical ichthyographs that map daily discharge, temperature, and fish use could also be mapped where data are available.

We can also develop an ichthyograph for a single species and represent the historical range of hydrologic conditions for each life-history stage of that species throughout the stream system. This allows for a transition from a temporal “migration ichthyograph” as we developed in Fig 3B to spatiotemporal ichthographs that depict the relationship between hydroclimate, life history and habitat across a channel network. We illustrate this type of ichthyograph using general relationships between hydrologic conditions in river systems on the Oregon coast and the timing and location of habitat use by Coho Salmon (Fig 4). At a mid-river location (Fig 4A), adult Coho Salmon migrate upstream in the fall to spawn and smolt migrate downstream to the ocean in the early spring [17]. Additionally, juvenile fish that are most likely using smaller tributaries and seeps in summer may also use deep pools and cooler-water micro-habitats as refugia when main river temperatures are warmer than the physiological optimum. Further, the mainstem provides an important outmigration pathway for juvenile fish in summer in support of diverse life histories that include estuary rearing strategies [18]. By organizing this information as a single visualization, ichthyographs communicate the complex relationships between fish life history and stream habitat use in one location throughout the year.

If we repeat this process to create ichthyographs for multiple locations within a drainage basin that are occupied by Coho Salmon during different life stages, we can build a more complete picture of habitat use across time and space (Fig 4B). The absolute values and precise shape of the relationship between streamflow and temperature will change depending on location within a stream network and the influence of snowmelt and groundwater, but tend to vary predictably with drainage area within a single watershed. The resolution of the data collected can become part of the layout of the ichthyograph both in terms of time (daily, weekly, monthly counts) and space (one site, multiple sites within the river network). Both of these elements are important when considering interpretations of the data, and their broader applicability to other locations. For Coho Salmon, an ichthyograph for the lower river (the largest drainage area location) may show the fewest life stages present, but illustrates that at this location, Coho Salmon experience a wide range of hydroclimates in both their upstream and downstream migrations. In contrast, the headwaters (smallest drainage area) support the most life stages over a longer period of time requiring tolerance of a variable hydroclimate among multiple seasons (Fig 4B).

Viewed as a whole, this series of ichthyographs (Fig 4B) traces the life history and associated hydroclimate for Coho Salmon from spawning and emergent fry in the headwater reaches, through smolt migration downstream to the lower river in the spring. We then see the return migration in the fall as adult fish move upstream from the lower river, back to the headwaters to spawn. This series of ichthyographs creates a template of historical conditions against which we can compare projected future conditions under different management and climate scenarios. It provides a foundation from which it may be possible to explore phenotypic sensitivity to projected changes in hydroclimatic conditions throughout the river network.

## Discussion and Conclusions

The ichthyograph is a powerful empirical visualization tool that characterizes linkages between hydroclimate and fish life history, allowing an assessment of the range of variability in environmental conditions in which a population has evolved and persisted. It can be used to identify stages in a species’ life cycle where future hydroclimate might lie outside the conditions experienced by these fish populations in the past, and which may represent critically-vulnerable stages in the species’ life history. This is particularly useful for water management planning, or to anticipate vulnerabilities of native fishes to climate change or other anthropogenic activities.

Here, we have illustrated the application of the ichthyograph to the assemblage of native fishes in one portion of the river network, and for a single species in multiple locations throughout a river network. Locations with high-resolution data on both hydroclimate and fish use, such as Winchester Dam, can provide valuable insights into the way that fish phenology is expressed both for individual species and for the assemblage of fish species that use a river system. Further, while we have mapped upstream migration timing, other species traits (such as adult body size of upstream migrants, smolt age/size of downstream migrants) could be mapped onto relevant hydroclimatic variables.

Quantitative, empirically driven ichthyographs developed with long-term hydroclimate and fish datasets such as at Winchester Dam are ideal, but unfortunately, uncommon. However, long-term hydrologic and thermal datasets are available and methods exist to interpolate hydroclimate conditions at local to regional scales. Combined with information from the published literature about run timing or tolerances for temperature and discharge by individual species, it may be possible to extend the ichthyograph concept into less data-rich environments. Next steps will be to investigate whether ichthyographs can adequately represent fish use patterns in data-poor rivers. Development of regional community ichthyographs could be used to help fisheries managers track the timing and trends in fish migration. Timing of fish migration is an important consideration in the designation of regulations for commercial and recreational fish harvest. Further, knowledge of the timing of fish use throughout the year informs other management activities, including the timing of in-stream restoration, road construction, or water withdrawal.

In practice, the ichthyograph can be used for qualitative and graphical enquiries (as we have demonstrated here), but can as easily be used in a quantitative and predictive context. For example, density functions or percentiles could be plotted rather than graphical interpretations in Fig 3 to explore patterns in empirical data (from sources that repeatedly sample fishes using a variety of methods including electrofishing, snorkeling or smolt traps). Further, ichthyographs can be used to link hydroclimate to population parameters (e.g. graphing adult-to-adult or adult-to-smolt recruitment). These empirical ichthyographs should allow for the non-linear, yet cyclical patterns of temperature and discharge to be considered alongside data describing fish phenology and population characteristics to understand historic or current patterns of use, thereby informing predictive modeling and management.

One of the critical challenges facing fisheries and land managers is assessing the ability of populations to persist in a changing climate. Climate change is likely to influence both streamflow [19–20] and water temperature [3], thereby increasing the potential for physiological stress in fish populations [21]. Using the ichthyograph, managers can not only identify points in a species’ life cycle where hydrologic conditions may already be reaching physiological limits, but also determine whether future conditions are expected to lie outside the hydrologic conditions experienced in the past, and may represent critically-vulnerable time periods in a species’ life history. For example, in the Frasier River system, BC, there is an extensive body of literature describing physiological tolerances of sockeye salmon (*Oncorhynchus nerka*) to hydroclimate [22, 23]. Documented trends towards sub-optimal migration timing by sockeye salmon in this system [24] could be explored using ichthyographs developed at multiple river locations under current and future climate scenarios.

Climate change is but one of many anthropogenic disturbances that may cause changes in river flow and temperature. In many systems, anthropogenic changes to hydrologic conditions already exceed predicted future effects from climate [3]. Water control and diversion devices, floodplain stabilization, and road construction have compromised natural processes that resulted in a diversity of connected and complex river habitats, across short (seasonal) and longer (annual, decadal, or longer) time steps. The powerful influence of land management, while responsible for declines in fish populations in the past, may be an important opportunity for fish habitat restoration that could be critical to the survival of fish populations in the future. Ichthyographs can be a useful tool to help identify the location and nature of restoration actions that will address fish habitat needs at key points in their life history by highlighting the critical relationship between fish phenology and hydroclimate across a range of spatiotemporal scales.

## Supporting Information

S1 Dataset Winchester Dam Fish Counts, Discharge and Water Temperature from 1991–2014. (PDF)
