# Supplementary material for: Linking Hydroclimate to Fish Phenology and Habitat Use with Ichthyographs
Source: PLoS One. 2016 Dec 22;11(12):e0168831. doi: 10.1371/journal.pone.0168831 (PMC5179265; doi:10.1371/journal.pone.0168831)
Supplement: S1 Dataset — (PDF) [file pone.0168831.s001.pdf]

| Date | Discharge (cms) | Temp (C°) | Chinook | Coho | Steelhead | Cutthroat | Lamprey | Sucker |
|---|---|---|---|---|---|---|---|---|
| 10/24/1991 | 29.17 | | 10 | 31 | 7 | 0 | 0 | 0 |
| 10/25/1991 | 30.87 | | 5 | 25 | 5 | 0 | 0 | 0 |
| 10/26/1991 | 82.40 | | 13 | 239 | 16 | 0 | 0 | 0 |
| 10/27/1991 | 69.38 | | 4 | 51 | 4 | 0 | 0 | 0 |
| 10/28/1991 | 49.27 | | 0 | 78 | 15 | 0 | 0 | 0 |
| 10/29/1991 | 46.72 | | 0 | 8 | 0 | 0 | 0 | 0 |
| 10/30/1991 | 61.73 | | 0 | 3 | 0 | 0 | 0 | 0 |
| 10/31/1991 | 41.34 | | 0 | 36 | 6 | 0 | 0 | 0 |
| 11/1/1991 | 34.83 | | 0 | 1 | 0 | 0 | 0 | 0 |
| 11/2/1991 | 32.28 | | 0 | 2 | 1 | 0 | 0 | 0 |
| 11/3/1991 | 30.87 | | 0 | 0 | 0 | 0 | 0 | 0 |
| 11/4/1991 | 30.30 | | 0 | 8 | 2 | 0 | 0 | 0 |
| 11/5/1991 | 29.73 | | 1 | 23 | 3 | 0 | 0 | 0 |
| 11/6/1991 | 47.57 | | 1 | 238 | 32 | 0 | 1 | 0 |
| 11/7/1991 | 62.58 | | 4 | 384 | 29 | 0 | 0 | 4 |
| 11/8/1991 | 50.69 | | 0 | 105 | 13 | 0 | 0 | 2 |
| 11/9/1991 | 41.06 | | 0 | 57 | 9 | 0 | 0 | 0 |
| 11/10/1991 | 38.23 | | 0 | 22 | 2 | 0 | 0 | 0 |
| 11/11/1991 | 34.26 | | 2 | 19 | 4 | 0 | 0 | 0 |
| 11/12/1991 | 34.26 | | 1 | 15 | 7 | 0 | 0 | 0 |
| 11/13/1991 | 83.53 | | 0 | 14 | 3 | 0 | 0 | 0 |
| 11/14/1991 | 112.70 | | 2 | 53 | 6 | 0 | 0 | 3 |
| 11/15/1991 | 82.12 | | 0 | 11 | 2 | 0 | 0 | 1 |
| 11/16/1991 | 59.18 | | 0 | 1 | 1 | 0 | 0 | 0 |
| 11/17/1991 | 117.51 | | 0 | 9 | 4 | 0 | 0 | 0 |
| 11/18/1991 | 246.64 | | 0 | 1 | 2 | 0 | 0 | 0 |
| 11/19/1991 | 166.22 | | 0 | 6 | 1 | 0 | 0 | 1 |
| 11/20/1991 | 237.30 | | 0 | 4 | 9 | 0 | 0 | 1 |
| 11/21/1991 | 280.90 | | 0 | 1 | 4 | 0 | 0 | 0 |
| 11/22/1991 | 164.52 | | 1 | 11 | 1 | 0 | 0 | 0 |
| 11/23/1991 | 111.85 | | 0 | 3 | 8 | 0 | 0 | 0 |
| 11/24/1991 | 93.16 | | 0 | 7 | 9 | 0 | 0 | 1 |
| 11/25/1991 | 123.18 | | 0 | 6 | 11 | 0 | 0 | 0 |
| 11/26/1991 | 226.82 | | 0 | 10 | 20 | 0 | 0 | 0 |
| 11/27/1991 | 351.13 | | 0 | 2 | 1 | 0 | 0 | 0 |
| 11/28/1991 | 224.55 | | 0 | 0 | 5 | 0 | 0 | 0 |
| 11/29/1991 | 158.01 | | 0 | 2 | 1 | 0 | 0 | 0 |
| 11/30/1991 | 125.73 | | 0 | 0 | 1 | 0 | 0 | 0 |
| 12/1/1991 | 99.68 | | 0 | 2 | 2 | 0 | 0 | 0 |
| 12/2/1991 | 106.75 | | 0 | 1 | 5 | 0 | 0 | 0 |
| 12/3/1991 | 135.35 | | 0 | 0 | 1 | 0 | 0 | 0 |
| 12/4/1991 | 110.44 | | 0 | 2 | 5 | 0 | 0 | 0 |
| 12/5/1991 | 98.54 | | 0 | 0 | 8 | 0 | 0 | 0 |
| 12/6/1991 | 441.74 | | 0 | 1 | 5 | 0 | 0 | 0 |
| 12/7/1991 | 719.25 | | 0 | 0 | 0 | 0 | 0 | 0 |

| Date | Discharge (cms) | Temp (C°) | Chinook | Coho | Steelhead | Cutthroat | Lamprey | Sucker |
|---|---|---|---|---|---|---|---|---|
| 12/8/1991 | 351.13 | | 0 | 0 | 4 | 0 | 0 | 0 |
| 12/9/1991 | 218.89 | | 0 | 6 | 5 | 0 | 0 | 0 |
| 12/10/1991 | 162.26 | | 0 | 2 | 8 | 0 | 0 | 0 |
| 12/11/1991 | 131.96 | | 0 | 0 | 6 | 0 | 0 | 0 |
| 12/12/1991 | 117.23 | | 0 | 0 | 4 | 0 | 0 | 0 |
| 12/13/1991 | 116.38 | | 0 | 0 | 2 | 0 | 0 | 0 |
| 12/14/1991 | 107.32 | | 0 | 1 | 6 | 0 | 0 | 0 |
| 12/15/1991 | 95.99 | | 0 | 0 | 0 | 0 | 0 | 0 |
| 12/16/1991 | 88.07 | | 0 | 0 | 1 | 0 | 0 | 0 |
| 12/17/1991 | 80.14 | | 0 | 0 | 0 | 0 | 0 | 0 |
| 12/18/1991 | 80.42 | | 0 | 0 | 2 | 0 | 0 | 0 |
| 12/19/1991 | 108.45 | | 0 | 1 | 5 | 0 | 0 | 0 |
| 12/20/1991 | 100.24 | | 0 | 0 | 2 | 0 | 0 | 0 |
| 12/21/1991 | 94.01 | | 0 | 1 | 1 | 0 | 0 | 0 |
| 12/22/1991 | 91.75 | | 0 | 0 | 2 | 0 | 0 | 0 |
| 12/23/1991 | 86.93 | | 0 | 0 | 3 | 0 | 0 | 0 |
| 12/24/1991 | 78.72 | | 0 | 0 | 1 | 0 | 0 | 0 |
| 12/25/1991 | 73.62 | | 0 | 0 | 3 | 0 | 0 | 0 |
| 12/26/1991 | 70.23 | | 0 | 0 | 3 | 0 | 0 | 0 |
| 12/27/1991 | 69.09 | | 0 | 3 | 8 | 0 | 0 | 0 |
| 12/28/1991 | 62.01 | | 0 | 0 | 23 | 0 | 0 | 0 |
| 12/29/1991 | 64.56 | | 0 | 0 | 37 | 0 | 0 | 0 |
| 12/30/1991 | 63.43 | | 0 | 0 | 2 | 0 | 0 | 0 |
| 12/31/1991 | 56.35 | | 0 | 0 | 2 | 0 | 0 | 0 |
| 1/1/1992 | 48.70 | 4.44 | 0 | 0 | 1 | 0 | 0 | 0 |
| 1/2/1992 | 48.42 | 4.44 | 0 | 0 | 0 | 0 | 0 | 0 |
| 1/3/1992 | 57.77 | 4.44 | 0 | 0 | 1 | 0 | 0 | 0 |
| 1/4/1992 | 64.85 | 5.00 | 0 | 0 | 8 | 0 | 0 | 0 |
| 1/5/1992 | 90.33 | 5.56 | 0 | 0 | 35 | 0 | 0 | 0 |
| 1/6/1992 | 86.08 | 5.56 | 0 | 2 | 7 | 0 | 0 | 0 |
| 1/7/1992 | 82.69 | 5.56 | 0 | 0 | 9 | 0 | 0 | 0 |
| 1/8/1992 | 72.21 | 5.56 | 0 | 0 | 0 | 0 | 0 | 0 |
| 1/9/1992 | 65.98 | 5.00 | 0 | 0 | 0 | 0 | 0 | 0 |
| 1/10/1992 | 64.85 | 5.00 | 0 | 0 | 2 | 0 | 0 | 0 |
| 1/11/1992 | 82.40 | 5.56 | 0 | 0 | 13 | 1 | 0 | 0 |
| 1/12/1992 | 91.18 | 5.00 | 0 | 0 | 7 | 0 | 0 | 0 |
| 1/13/1992 | 84.10 | 5.00 | 0 | 0 | 2 | 0 | 0 | 0 |
| 1/14/1992 | 75.89 | 5.56 | 0 | 0 | 9 | 0 | 0 | 0 |
| 1/15/1992 | 70.51 | 5.56 | 0 | 0 | 8 | 0 | 0 | 0 |
| 1/16/1992 | 70.51 | 5.56 | 2 | 0 | 46 | 0 | 0 | 0 |
| 1/17/1992 | 80.99 | 6.11 | 1 | 0 | 78 | 0 | 0 | 0 |
| 1/18/1992 | 76.46 | 5.56 | 0 | 0 | 3 | 0 | 0 | 0 |
| 1/19/1992 | 71.08 | 4.44 | 0 | 0 | 0 | 0 | 0 | 0 |
| 1/20/1992 | 64.00 | 3.33 | 0 | 0 | 0 | 0 | 0 | 0 |
| 1/21/1992 | 61.16 | 3.89 | 0 | 0 | 0 | 0 | 0 | 0 |

| Date | Discharge (cms) | Temp (C°) | Chinook | Coho | Steelhead | Cutthroat | Lamprey | Sucker |
|---|---|---|---|---|---|---|---|---|
| 1/22/1992 | 55.78 | 5.00 | 0 | 0 | 1 | 0 | 0 | 0 |
| 1/23/1992 | 52.67 | 5.00 | 0 | 0 | 4 | 0 | 0 | 0 |
| 1/24/1992 | 50.69 | 5.00 | 0 | 0 | 2 | 0 | 0 | 0 |
| 1/25/1992 | 51.25 | 6.11 | 0 | 0 | 67 | 0 | 0 | 0 |
| 1/26/1992 | 56.07 | 6.11 | 0 | 0 | 57 | 0 | 0 | 0 |
| 1/27/1992 | 56.92 | 6.67 | 0 | 0 | 84 | 0 | 0 | 0 |
| 1/28/1992 | 78.15 | 7.22 | 0 | 0 | 135 | 0 | 0 | 3 |
| 1/29/1992 | 112.13 | 7.22 | 0 | 0 | 95 | 0 | 0 | 3 |
| 1/30/1992 | 90.61 | 7.22 | 0 | 0 | 48 | 0 | 0 | 0 |
| 1/31/1992 | 76.17 | 7.22 | 0 | 0 | 27 | 0 | 0 | 0 |
| 2/1/1992 | 104.77 | 7.22 | 0 | 0 | 151 | 0 | 0 | 5 |
| 2/2/1992 | 107.04 | 7.22 | 0 | 0 | 15 | 0 | 0 | 1 |
| 2/3/1992 | 91.75 | 6.67 | 0 | 0 | 3 | 0 | 0 | 0 |
| 2/4/1992 | 81.55 | 5.56 | 0 | 0 | 3 | 0 | 0 | 0 |
| 2/5/1992 | 70.79 | 5.56 | 0 | 0 | 4 | 0 | 0 | 0 |
| 2/6/1992 | 65.70 | 6.11 | 0 | 0 | 4 | 0 | 0 | 0 |
| 2/7/1992 | 63.15 | 5.56 | 0 | 0 | 5 | 0 | 0 | 0 |
| 2/8/1992 | 57.20 | 6.11 | 0 | 0 | 2 | 0 | 0 | 0 |
| 2/9/1992 | 58.33 | 6.11 | 0 | 0 | 15 | 0 | 0 | 0 |
| 2/10/1992 | 53.80 | 7.22 | 0 | 0 | 29 | 0 | 0 | 0 |
| 2/11/1992 | 51.54 | 7.22 | 0 | 0 | 51 | 0 | 0 | 0 |
| 2/12/1992 | 49.84 | 7.78 | 0 | 0 | 42 | 0 | 0 | 0 |
| 2/13/1992 | 50.97 | 7.78 | 0 | 0 | 32 | 0 | 0 | 0 |
| 2/14/1992 | 49.55 | 7.22 | 0 | 0 | 22 | 0 | 0 | 0 |
| 2/15/1992 | 52.39 | 6.67 | 0 | 0 | 14 | 0 | 0 | 0 |
| 2/16/1992 | 50.69 | 6.67 | 0 | 0 | 11 | 0 | 0 | 0 |
| 2/17/1992 | 60.03 | 6.67 | 0 | 0 | 5 | 0 | 0 | 0 |
| 2/18/1992 | 66.83 | 7.22 | 0 | 0 | 25 | 0 | 0 | 1 |
| 2/19/1992 | 86.37 | 7.22 | 0 | 0 | 191 | 0 | 0 | 0 |
| 2/20/1992 | 153.19 | 7.78 | 0 | 0 | 332 | 0 | 0 | 3 |
| 2/21/1992 | 241.26 | 8.33 | 0 | 0 | 94 | 0 | 0 | 1 |
| 2/22/1992 | 274.96 | 8.33 | 0 | 0 | 81 | 0 | 0 | 1 |
| 2/23/1992 | 221.44 | 8.33 | 0 | 0 | 35 | 0 | 0 | 0 |
| 2/24/1992 | 158.57 | 8.33 | 0 | 0 | 42 | 0 | 0 | 0 |
| 2/25/1992 | 126.58 | 8.89 | 0 | 0 | 69 | 0 | 0 | 1 |
| 2/26/1992 | 108.17 | 8.89 | 0 | 0 | 41 | 0 | 0 | 2 |
| 2/27/1992 | 95.43 | 9.44 | 0 | 0 | 41 | 0 | 0 | 2 |
| 2/28/1992 | 84.95 | 8.89 | 0 | 0 | 39 | 0 | 0 | 0 |
| 2/29/1992 | 77.59 | 8.33 | 0 | 0 | 37 | 0 | 0 | 0 |
| 3/1/1992 | 74.19 | 9.44 | 0 | 0 | 40 | 0 | 0 | 0 |
| 3/2/1992 | 68.81 | 9.44 | 0 | 0 | 14 | 0 | 0 | 0 |
| 3/3/1992 | 62.30 | 9.44 | 0 | 0 | 20 | 0 | 0 | 2 |
| 3/4/1992 | 58.62 | 8.89 | 0 | 0 | 6 | 0 | 0 | 0 |
| 3/5/1992 | 66.26 | 8.89 | 0 | 0 | 28 | 0 | 0 | 0 |
| 3/6/1992 | 61.16 | 8.89 | 0 | 0 | 6 | 0 | 0 | 0 |

| Date | Discharge (cms) | Temp (C°) | Chinook | Coho | Steelhead | Cutthroat | Lamprey | Sucker |
|---|---|---|---|---|---|---|---|---|
| 3/7/1992 | 56.07 | 9.44 | 0 | 0 | 81 | 0 | 0 | 0 |
| 3/8/1992 | 54.37 | 8.89 | 0 | 0 | 5 | 0 | 0 | 0 |
| 3/9/1992 | 52.67 | 8.89 | 0 | 0 | 20 | 0 | 0 | 0 |
| 3/10/1992 | 50.40 | 8.89 | 0 | 0 | 14 | 0 | 0 | 0 |
| 3/11/1992 | 48.14 | 9.44 | 0 | 0 | 29 | 0 | 0 | 0 |
| 3/12/1992 | 46.72 | 8.89 | 0 | 0 | 81 | 0 | 0 | 1 |
| 3/13/1992 | 46.44 | 10.00 | 0 | 0 | 68 | 0 | 0 | 4 |
| 3/14/1992 | 44.74 | 9.44 | 1 | 0 | 42 | 0 | 0 | 6 |
| 3/15/1992 | 44.17 | 8.89 | 0 | 0 | 23 | 0 | 0 | 4 |
| 3/16/1992 | 43.32 | 9.44 | 0 | 0 | 44 | 0 | 0 | 0 |
| 3/17/1992 | 46.44 | 10.00 | 1 | 0 | 151 | 0 | 0 | 127 |
| 3/18/1992 | 46.44 | 10.00 | 0 | 0 | 16 | 0 | 0 | 5 |
| 3/19/1992 | 43.89 | 10.00 | 5 | 0 | 43 | 0 | 0 | 2 |
| 3/20/1992 | 42.76 | 10.56 | 0 | 0 | 26 | 0 | 0 | 24 |
| 3/21/1992 | 41.34 | | 0 | 0 | 95 | 0 | 0 | 0 |
| 3/22/1992 | 40.21 | | 0 | 0 | 0 | 0 | 0 | 0 |
| 3/23/1992 | 36.81 | 11.67 | 1 | 0 | 150 | 0 | 0 | 142 |
| 3/24/1992 | 38.79 | 12.22 | 8 | 0 | 157 | 0 | 0 | 306 |
| 3/25/1992 | 38.51 | 12.22 | 1 | 0 | 109 | 0 | 0 | 326 |
| 3/26/1992 | 36.81 | 11.11 | 2 | 0 | 54 | 0 | 0 | 60 |
| 3/27/1992 | 37.66 | 10.56 | 1 | 0 | 5 | 0 | 0 | 0 |
| 3/28/1992 | 37.38 | 11.67 | 4 | 0 | 42 | 0 | 0 | 0 |
| 3/29/1992 | 36.53 | 12.22 | 11 | 0 | 85 | 0 | 0 | 31 |
| 3/30/1992 | 37.10 | 11.67 | 13 | 0 | 103 | 0 | 0 | 377 |
| 3/31/1992 | 36.81 | 12.78 | 8 | 0 | 46 | 0 | 5 | 267 |
| 4/1/1992 | 32.85 | 13.33 | 5 | 0 | 26 | 0 | 0 | 138 |
| 4/2/1992 | 32.28 | 15.00 | 15 | 0 | 68 | 0 | 1 | 617 |
| 4/3/1992 | 32.00 | 14.44 | 14 | 0 | 23 | 0 | 2 | 338 |
| 4/4/1992 | 32.00 | 12.22 | 12 | 0 | 23 | 0 | 0 | 6 |
| 4/5/1992 | 33.70 | 11.11 | 5 | 0 | 10 | 0 | 1 | 0 |
| 4/6/1992 | 35.11 | 11.67 | 7 | 0 | 17 | 0 | 0 | 0 |
| 4/7/1992 | 35.68 | 10.56 | 4 | 0 | 15 | 0 | 0 | 0 |
| 4/8/1992 | 35.11 | 10.00 | 26 | 0 | 16 | 0 | 0 | 0 |
| 4/9/1992 | 92.88 | 10.00 | 18 | 0 | 26 | 0 | 18 | 7 |
| 4/10/1992 | 404.93 | 10.00 | 1 | 0 | 3 | 0 | 4 | 0 |
| 4/11/1992 | 213.23 | 10.00 | 0 | 0 | 13 | 0 | 1 | 87 |
| 4/12/1992 | 158.57 | 10.56 | 6 | 0 | 17 | 0 | 33 | 286 |
| 4/13/1992 | 164.24 | 10.56 | 7 | 0 | 12 | 0 | 22 | 69 |
| 4/14/1992 | 143.85 | 11.67 | 10 | 0 | 9 | 0 | 40 | 307 |
| 4/15/1992 | 121.20 | 12.22 | 28 | 0 | 11 | 0 | 15 | 689 |
| 4/16/1992 | 105.91 | 11.11 | 23 | 0 | 16 | 0 | 11 | 420 |
| 4/17/1992 | 106.47 | 10.56 | 5 | 0 | 12 | 0 | 30 | 140 |
| 4/18/1992 | 311.49 | 10.00 | 0 | 0 | 3 | 0 | 18 | 0 |
| 4/19/1992 | 308.65 | 10.56 | 0 | 0 | 4 | 0 | 0 | 0 |
| 4/20/1992 | 197.65 | 10.56 | 3 | 0 | 9 | 0 | 1 | 2 |

| Date | Discharge (cms) | Temp (C°) | Chinook | Coho | Steelhead | Cutthroat | Lamprey | Sucker |
|---|---|---|---|---|---|---|---|---|
| 4/21/1992 | 143.28 | 10.56 | 11 | 0 | 6 | 0 | 17 | 1 |
| 4/22/1992 | 117.80 | | 0 | 0 | 0 | 0 | 0 | 0 |
| 4/23/1992 | 99.39 | | 0 | 0 | 0 | 0 | 0 | 0 |
| 4/24/1992 | 84.67 | 11.11 | 33 | 0 | 5 | 0 | 6 | 77 |
| 4/25/1992 | 82.40 | 12.22 | 1 | 0 | 2 | 0 | 0 | 42 |
| 4/26/1992 | 74.47 | 12.22 | 0 | 0 | 0 | 0 | 0 | 0 |
| 4/27/1992 | 70.23 | 12.22 | 0 | 0 | 0 | 0 | 0 | 0 |
| 4/28/1992 | 65.98 | 14.44 | 31 | 0 | 12 | 0 | 0 | 245 |
| 4/29/1992 | 58.33 | | 41 | 0 | 15 | 0 | 0 | 42 |
| 4/30/1992 | 60.31 | 13.33 | 159 | 0 | 10 | 0 | 4 | 225 |
| 5/1/1992 | 75.61 | 13.89 | 27 | 0 | 1 | 0 | 0 | 8 |
| 5/2/1992 | 72.49 | 14.44 | 15 | 0 | 0 | 0 | 0 | 33 |
| 5/3/1992 | 64.56 | 15.56 | 4 | 0 | 0 | 0 | 0 | 1 |
| 5/4/1992 | 57.77 | 16.67 | 60 | 0 | 0 | 0 | 0 | 108 |
| 5/5/1992 | 50.97 | 17.78 | 30 | 0 | 2 | 0 | 0 | 35 |
| 5/6/1992 | 50.69 | 18.33 | 161 | 0 | 3 | 0 | 0 | 424 |
| 5/7/1992 | 45.59 | 18.89 | 162 | 0 | 3 | 0 | 2 | 326 |
| 5/8/1992 | 45.59 | 17.78 | 101 | 0 | 6 | 0 | 4 | 99 |
| 5/9/1992 | 43.32 | 17.78 | 72 | 0 | 4 | 0 | 0 | 48 |
| 5/10/1992 | 41.63 | 17.78 | 147 | 0 | 1 | 0 | 0 | 116 |
| 5/11/1992 | 40.21 | 17.22 | 91 | 0 | 5 | 0 | 2 | 4 |
| 5/12/1992 | 39.64 | 16.11 | 126 | 0 | 3 | 0 | 0 | 0 |
| 5/13/1992 | 39.93 | 17.22 | 95 | 0 | 4 | 0 | 1 | 4 |
| 5/14/1992 | 36.25 | 17.22 | 147 | 0 | 4 | 0 | 3 | 43 |
| 5/15/1992 | 32.56 | 18.33 | 76 | 0 | 6 | 0 | 0 | 88 |
| 5/16/1992 | 32.00 | 18.33 | 53 | 0 | 7 | 0 | 4 | 118 |
| 5/17/1992 | 32.28 | 19.44 | 59 | 0 | 10 | 0 | 9 | 96 |
| 5/18/1992 | 30.87 | 19.44 | 63 | 0 | 8 | 0 | 17 | 150 |
| 5/19/1992 | 30.02 | 18.33 | 69 | 0 | 7 | 0 | 22 | 101 |
| 5/20/1992 | 34.83 | 17.22 | 51 | 0 | 7 | 0 | 1 | 25 |
| 5/21/1992 | 39.93 | 17.22 | 63 | 0 | 2 | 0 | 0 | 45 |
| 5/22/1992 | 34.26 | 17.78 | 69 | 0 | 5 | 0 | 0 | 64 |
| 5/23/1992 | 33.41 | 18.89 | 42 | 0 | 5 | 0 | 3 | 472 |
| 5/24/1992 | 33.41 | 20.00 | 45 | 0 | 2 | 0 | 10 | 225 |
| 5/25/1992 | 28.88 | 19.44 | 25 | 0 | 4 | 0 | 4 | 66 |
| 5/26/1992 | 32.85 | 20.00 | 25 | 0 | 0 | 0 | 0 | 10 |
| 5/27/1992 | 29.73 | 20.00 | 19 | 0 | 1 | 0 | 0 | 1 |
| 5/28/1992 | 28.29 | 20.00 | 31 | 0 | 19 | 0 | 0 | 5 |
| 5/29/1992 | 31.43 | 20.00 | 37 | 0 | 0 | 0 | 0 | 2 |
| 5/30/1992 | 27.69 | 20.00 | 33 | 0 | 17 | 0 | 1 | 25 |
| 5/31/1992 | 27.86 | 20.00 | 115 | 0 | 18 | 0 | 4 | 17 |
| 6/1/1992 | 26.99 | 23.33 | 55 | 0 | 6 | 0 | 3 | 18 |
| 6/2/1992 | 25.88 | 21.11 | 26 | 0 | 9 | 0 | 21 | 38 |
| 6/3/1992 | 28.88 | 21.11 | 24 | 0 | 6 | 0 | 15 | 23 |
| 6/4/1992 | 30.02 | 21.11 | 24 | 0 | 1 | 0 | 2 | 59 |

| Date | Discharge (cms) | Temp (C°) | Chinook | Coho | Steelhead | Cutthroat | Lamprey | Sucker |
|---|---|---|---|---|---|---|---|---|
| 6/5/1992 | 25.51 | 20.56 | 33 | 0 | 4 | 0 | 5 | 16 |
| 6/6/1992 | 24.38 | 20.56 | 25 | 0 | 2 | 0 | 3 | 12 |
| 6/7/1992 | 23.36 | 21.11 | 16 | 0 | 3 | 0 | 0 | 1 |
| 6/8/1992 | 23.33 | 20.00 | 9 | 0 | 0 | 0 | 3 | 2 |
| 6/9/1992 | 22.96 | | 2 | 0 | 1 | 0 | 9 | 5 |
| 6/10/1992 | 22.94 | | 13 | 0 | 0 | 0 | 13 | 3 |
| 6/11/1992 | 23.08 | | 12 | 0 | 1 | 0 | 6 | 0 |
| 6/12/1992 | 24.21 | | 27 | 0 | 3 | 0 | 0 | 0 |
| 6/13/1992 | 28.60 | 16.67 | 9 | 0 | 1 | 0 | 0 | 0 |
| 6/14/1992 | 36.81 | 16.11 | 30 | 0 | 4 | 0 | 0 | 5 |
| 6/15/1992 | 33.98 | 16.11 | 51 | 0 | 35 | 0 | 0 | 40 |
| 6/16/1992 | 28.60 | 16.11 | 44 | 0 | 24 | 0 | 0 | 0 |
| 6/17/1992 | 26.45 | 17.78 | 23 | 0 | 28 | 0 | 0 | 9 |
| 6/18/1992 | 25.20 | 20.00 | 84 | 0 | 40 | 0 | 0 | 12 |
| 6/19/1992 | 24.13 | 21.11 | 50 | 0 | 26 | 0 | 2 | 18 |
| 6/20/1992 | 23.36 | 22.22 | 56 | 0 | 17 | 0 | 24 | 7 |
| 6/21/1992 | 22.91 | 23.89 | 39 | 0 | 23 | 0 | 26 | 3 |
| 6/22/1992 | 23.13 | 25.00 | 22 | 0 | 22 | 0 | 55 | 13 |
| 6/23/1992 | 22.99 | 25.00 | 4 | 0 | 14 | 0 | 32 | 15 |
| 6/24/1992 | 23.45 | 24.44 | 2 | 0 | 1 | 0 | 20 | 35 |
| 6/25/1992 | 21.66 | 23.89 | 1 | 0 | 0 | 0 | 18 | 20 |
| 6/26/1992 | 21.41 | 23.33 | 0 | 0 | 1 | 0 | 9 | 13 |
| 6/27/1992 | 22.80 | 22.78 | 0 | 0 | 3 | 0 | 1 | 3 |
| 6/28/1992 | 22.96 | 21.67 | 2 | 0 | 0 | 0 | 5 | 4 |
| 6/29/1992 | 30.02 | 19.44 | 0 | 0 | 0 | 0 | 0 | 10 |
| 6/30/1992 | 38.79 | 19.44 | 5 | 0 | 1 | 0 | 0 | 334 |
| 7/1/1992 | 34.55 | 20.00 | 3 | 0 | 6 | 0 | 6 | 78 |
| 7/2/1992 | 35.11 | 20.00 | 11 | 0 | 7 | 0 | 4 | 145 |
| 7/3/1992 | 28.88 | 20.56 | 14 | 0 | 6 | 0 | 27 | 5 |
| 7/4/1992 | 28.32 | 20.56 | 40 | 0 | 27 | 0 | 17 | 1 |
| 7/5/1992 | 31.15 | | 11 | 0 | 6 | 0 | 1 | 0 |
| 7/6/1992 | 32.28 | | 31 | 0 | 54 | 0 | 0 | 0 |
| 7/7/1992 | 31.71 | | 25 | 0 | 33 | 0 | 0 | 0 |
| 7/8/1992 | 26.56 | 21.11 | 25 | 0 | 58 | 0 | 7 | 0 |
| 7/9/1992 | 24.86 | 21.67 | 36 | 0 | 53 | 0 | 4 | 0 |
| 7/10/1992 | 24.32 | 22.22 | 56 | 0 | 62 | 0 | 17 | 0 |
| 7/11/1992 | 25.37 | 22.22 | 35 | 0 | 31 | 0 | 5 | 3 |
| 7/12/1992 | 23.59 | 23.33 | 17 | 0 | 26 | 0 | 21 | 4 |
| 7/13/1992 | 23.19 | 23.33 | 16 | 0 | 22 | 0 | 24 | 0 |
| 7/14/1992 | 22.94 | 22.78 | 5 | 0 | 11 | 0 | 13 | 0 |
| 7/15/1992 | 23.02 | 23.33 | 4 | 0 | 10 | 0 | 16 | 1 |
| 7/16/1992 | 22.54 | 23.89 | 2 | 0 | 5 | 0 | 8 | 0 |
| 7/17/1992 | 20.61 | 23.33 | 0 | 0 | 4 | 0 | 6 | 0 |
| 7/18/1992 | 20.53 | 24.44 | 1 | 0 | 0 | 0 | 11 | 0 |
| 7/19/1992 | 20.59 | 24.44 | 0 | 0 | 1 | 0 | 9 | 0 |

| Date | Discharge (cms) | Temp (C°) | Chinook | Coho | Steelhead | Cutthroat | Lamprey | Sucker |
|---|---|---|---|---|---|---|---|---|
| 7/20/1992 | 20.33 | 23.33 | 0 | 0 | 1 | 0 | 12 | 0 |
| 7/21/1992 | 20.30 | 22.22 | 0 | 0 | 0 | 0 | 5 | 0 |
| 7/22/1992 | 21.21 | 21.11 | 0 | 0 | 2 | 0 | 1 | 0 |
| 7/23/1992 | 22.54 | 18.89 | 3 | 0 | 0 | 0 | 0 | 0 |
| 7/24/1992 | 21.92 | 20.56 | 14 | 0 | 0 | 0 | 0 | 1 |
| 7/25/1992 | 21.95 | 21.67 | 20 | 0 | 3 | 0 | 4 | 0 |
| 7/26/1992 | 21.49 | 22.78 | 46 | 0 | 6 | 0 | 4 | 0 |
| 7/27/1992 | 20.36 | 23.33 | 23 | 0 | 9 | 0 | 13 | 0 |
| 7/28/1992 | 20.13 | 23.33 | 20 | 0 | 33 | 0 | 2 | 0 |
| 7/29/1992 | 19.40 | 23.33 | 19 | 0 | 16 | 0 | 10 | 0 |
| 7/30/1992 | 19.45 | 23.89 | 7 | 0 | 19 | 0 | 6 | 0 |
| 7/31/1992 | 19.23 | 23.89 | 3 | 0 | 17 | 0 | 8 | 0 |
| 8/1/1992 | 19.26 | 22.78 | 1 | 0 | 5 | 0 | 19 | 0 |
| 8/2/1992 | 19.23 | 22.78 | 1 | 0 | 1 | 0 | 1 | 0 |
| 8/3/1992 | 18.97 | 22.78 | 2 | 0 | 5 | 0 | 1 | 1 |
| 8/4/1992 | 19.03 | 22.22 | 2 | 0 | 0 | 0 | 2 | 0 |
| 8/5/1992 | 18.94 | 21.67 | 2 | 0 | 2 | 0 | 0 | 0 |
| 8/6/1992 | 18.92 | 21.67 | 2 | 0 | 1 | 0 | 0 | 0 |
| 8/7/1992 | 18.94 | 21.67 | 5 | 0 | 1 | 0 | 0 | 0 |
| 8/8/1992 | 18.86 | 21.11 | 10 | 0 | 2 | 0 | 0 | 0 |
| 8/9/1992 | 18.77 | 21.11 | 11 | 0 | 0 | 0 | 0 | 0 |
| 8/10/1992 | 18.80 | 21.67 | 9 | 0 | 5 | 0 | 0 | 0 |
| 8/11/1992 | 18.66 | 22.22 | 10 | 0 | 11 | 0 | 1 | 0 |
| 8/12/1992 | 18.66 | 22.22 | 14 | 0 | 17 | 0 | 1 | 0 |
| 8/13/1992 | 18.41 | 22.78 | 15 | 0 | 17 | 0 | 1 | 0 |
| 8/14/1992 | 17.78 | 23.33 | 2 | 0 | 14 | 0 | 2 | 0 |
| 8/15/1992 | 17.67 | 22.78 | 5 | 0 | 7 | 0 | 0 | 0 |
| 8/16/1992 | 17.64 | 22.78 | 3 | 0 | 8 | 0 | 0 | 0 |
| 8/17/1992 | 17.44 | 23.33 | 2 | 0 | 1 | 0 | 0 | 0 |
| 8/18/1992 | 17.27 | 22.78 | 2 | 0 | 0 | 0 | 0 | 0 |
| 8/19/1992 | 17.30 | 23.33 | 0 | 0 | 0 | 0 | 0 | 0 |
| 8/20/1992 | 17.10 | 22.78 | 0 | 0 | 0 | 0 | 1 | 0 |
| 8/21/1992 | 17.22 | 21.67 | 1 | 0 | 0 | 0 | 0 | 0 |
| 8/22/1992 | 17.47 | 19.44 | 0 | 0 | 0 | 0 | 0 | 0 |
| 8/23/1992 | 17.67 | 19.44 | 2 | 0 | 0 | 0 | 0 | 0 |
| 8/24/1992 | 17.64 | 18.89 | 0 | 0 | 0 | 0 | 0 | 0 |
| 8/25/1992 | 17.56 | 18.89 | 18 | 0 | 6 | 0 | 0 | 0 |
| 8/26/1992 | 17.33 | 18.89 | 17 | 0 | 11 | 0 | 0 | 0 |
| 8/27/1992 | 17.02 | 18.89 | 10 | 0 | 17 | 0 | 0 | 0 |
| 8/28/1992 | 17.10 | 19.44 | 15 | 0 | 23 | 0 | 0 | 1 |
| 8/29/1992 | 16.91 | 18.89 | 19 | 0 | 14 | 0 | 1 | 0 |
| 8/30/1992 | 17.05 | 18.89 | 11 | 0 | 14 | 0 | 0 | 0 |
| 8/31/1992 | 17.16 | 18.33 | 15 | 0 | 26 | 0 | 0 | 0 |
| 9/1/1992 | 17.36 | 18.89 | 10 | 0 | 11 | 0 | 0 | 0 |
| 9/2/1992 | 17.27 | 18.89 | 4 | 0 | 14 | 0 | 0 | 0 |

| Date | Discharge (cms) | Temp (C°) | Chinook | Coho | Steelhead | Cutthroat | Lamprey | Sucker |
|---|---|---|---|---|---|---|---|---|
| 9/3/1992 | 17.36 | 18.89 | 10 | 0 | 5 | 0 | 0 | 0 |
| 9/4/1992 | 17.70 | 18.89 | 7 | 0 | 13 | 0 | 0 | 0 |
| 9/5/1992 | 18.41 | 18.33 | 2 | 0 | 12 | 0 | 0 | 0 |
| 9/6/1992 | 18.83 | 17.22 | 12 | 0 | 5 | 0 | 0 | 0 |
| 9/7/1992 | 18.69 | 16.67 | 2 | 0 | 6 | 0 | 0 | 0 |
| 9/8/1992 | 18.07 | 16.11 | 2 | 0 | 2 | 0 | 0 | 0 |
| 9/9/1992 | 17.73 | 16.67 | 8 | 0 | 11 | 0 | 0 | 0 |
| 9/10/1992 | 17.81 | 16.67 | 6 | 0 | 8 | 0 | 0 | 0 |
| 9/11/1992 | 18.04 | 17.22 | 1 | 0 | 1 | 0 | 0 | 0 |
| 9/12/1992 | 18.38 | 16.11 | 0 | 0 | 0 | 0 | 0 | 0 |
| 9/13/1992 | 18.35 | 15.56 | 0 | 0 | 0 | 0 | 0 | 0 |
| 9/14/1992 | 18.35 | 15.00 | 0 | 0 | 0 | 0 | 0 | 0 |
| 9/15/1992 | 18.24 | 14.44 | 2 | 0 | 2 | 0 | 0 | 0 |
| 9/16/1992 | 18.69 | 14.44 | 0 | 0 | 2 | 0 | 0 | 0 |
| 9/17/1992 | 19.09 | 14.44 | 0 | 0 | 0 | 0 | 0 | 0 |
| 9/18/1992 | 21.15 | 15.56 | 8 | 0 | 13 | 0 | 0 | 0 |
| 9/19/1992 | 22.51 | 15.56 | 0 | 0 | 0 | 0 | 0 | 0 |
| 9/20/1992 | 21.89 | 16.11 | 4 | 0 | 11 | 0 | 0 | 0 |
| 9/21/1992 | 21.75 | 16.67 | 18 | 8 | 10 | 0 | 0 | 0 |
| 9/22/1992 | 22.14 | 16.67 | 11 | 10 | 11 | 0 | 0 | 0 |
| 9/23/1992 | 21.44 | 16.11 | 1 | 0 | 9 | 0 | 0 | 0 |
| 9/24/1992 | 23.02 | 16.11 | 0 | 0 | 0 | 0 | 0 | 0 |
| 9/25/1992 | 27.58 | 15.56 | 22 | 7 | 21 | 0 | 0 | 0 |
| 9/26/1992 | 27.78 | 16.11 | 5 | 6 | 8 | 0 | 0 | 0 |
| 9/27/1992 | 25.34 | 16.11 | 11 | 2 | 4 | 0 | 0 | 0 |
| 9/28/1992 | 23.28 | 16.11 | 5 | 3 | 8 | 0 | 0 | 0 |
| 9/29/1992 | 22.40 | 14.44 | 0 | 2 | 3 | 0 | 0 | 0 |
| 9/30/1992 | 22.34 | 13.89 | 0 | 0 | 0 | 0 | 0 | 0 |
| 10/1/1992 | 22.68 | 13.33 | 2 | 3 | 3 | 0 | 0 | 0 |
| 10/2/1992 | 22.77 | 13.33 | 1 | 7 | 1 | 0 | 0 | 0 |
| 10/3/1992 | 23.30 | 12.78 | 18 | 10 | 4 | 0 | 0 | 0 |
| 10/4/1992 | 23.70 | 12.78 | 11 | 9 | 3 | 0 | 0 | 0 |
| 10/5/1992 | 23.87 | 12.78 | 5 | 22 | 3 | 0 | 0 | 0 |
| 10/6/1992 | 23.25 | 12.22 | 3 | 26 | 0 | 0 | 0 | 0 |
| 10/7/1992 | 22.85 | 12.22 | 1 | 15 | 0 | 0 | 0 | 0 |
| 10/8/1992 | 21.69 | 11.67 | 1 | 13 | 0 | 0 | 0 | 0 |
| 10/9/1992 | 20.53 | 11.67 | 3 | 14 | 2 | 0 | 0 | 0 |
| 10/10/1992 | 19.37 | 11.67 | 5 | 10 | 2 | 0 | 0 | 0 |
| 10/11/1992 | 19.34 | 11.67 | 7 | 13 | 0 | 0 | 0 | 0 |
| 10/12/1992 | 19.51 | 11.67 | 7 | 16 | 1 | 0 | 0 | 0 |
| 10/13/1992 | 19.34 | 11.11 | 4 | 30 | 0 | 0 | 0 | 0 |
| 10/14/1992 | 19.26 | 11.11 | 1 | 24 | 4 | 0 | 0 | 0 |
| 10/15/1992 | 19.20 | 10.00 | 2 | 0 | 2 | 0 | 0 | 0 |
| 10/16/1992 | 19.31 | 10.00 | 0 | 1 | 1 | 0 | 0 | 0 |
| 10/17/1992 | 19.31 | 10.56 | 0 | 12 | 1 | 0 | 0 | 0 |

| Date | Discharge (cms) | Temp (C°) | Chinook | Coho | Steelhead | Cutthroat | Lamprey | Sucker |
|---|---|---|---|---|---|---|---|---|
| 10/18/1992 | 19.34 | 11.11 | 2 | 11 | 5 | 0 | 0 | 0 |
| 10/19/1992 | 19.37 | 11.67 | 4 | 33 | 4 | 0 | 0 | 0 |
| 10/20/1992 | 19.57 | 11.11 | 4 | 18 | 13 | 0 | 0 | 0 |
| 10/21/1992 | 21.72 | 11.11 | 5 | 47 | 11 | 0 | 0 | 0 |
| 10/22/1992 | 28.60 | 11.11 | 1 | 11 | 3 | 0 | 0 | 0 |
| 10/23/1992 | 23.96 | 11.11 | 6 | 130 | 16 | 0 | 0 | 0 |
| 10/24/1992 | 21.92 | 11.67 | 2 | 77 | 10 | 0 | 0 | 0 |
| 10/25/1992 | 20.90 | 11.67 | 3 | 39 | 8 | 0 | 0 | 0 |
| 10/26/1992 | 21.04 | 11.11 | 2 | 20 | 2 | 0 | 0 | 0 |
| 10/27/1992 | 20.42 | 10.56 | 1 | 2 | 1 | 0 | 0 | 0 |
| 10/28/1992 | 20.44 | 11.11 | 3 | 22 | 5 | 0 | 0 | 0 |
| 10/29/1992 | 21.86 | 10.56 | 2 | 14 | 1 | 0 | 0 | 0 |
| 10/30/1992 | 32.56 | 10.00 | 0 | 21 | 6 | 0 | 0 | 0 |
| 10/31/1992 | 53.52 | 9.44 | 0 | 95 | 4 | 0 | 0 | 0 |
| 11/1/1992 | 103.07 | 9.44 | 0 | 0 | 0 | 0 | 0 | 0 |
| 11/2/1992 | 190.57 | 9.44 | 0 | 44 | 3 | 0 | 0 | 0 |
| 11/3/1992 | 99.11 | 9.44 | 2 | 145 | 24 | 0 | 0 | 0 |
| 11/4/1992 | 58.90 | 8.89 | 2 | 192 | 21 | 0 | 0 | 0 |
| 11/5/1992 | 47.01 | 8.89 | 1 | 116 | 10 | 0 | 0 | 0 |
| 11/6/1992 | 43.61 | 10.00 | 2 | 113 | 17 | 0 | 0 | 0 |
| 11/7/1992 | 39.36 | 10.00 | 1 | 85 | 12 | 0 | 0 | 0 |
| 11/8/1992 | 54.65 | 9.44 | 0 | 0 | 0 | 0 | 0 | 0 |
| 11/9/1992 | 75.61 | 8.89 | 1 | 55 | 5 | 0 | 0 | 0 |
| 11/10/1992 | 73.06 | 7.22 | 0 | 16 | 1 | 0 | 0 | 0 |
| 11/11/1992 | 54.09 | 5.56 | 1 | 70 | 3 | 0 | 0 | 0 |
| 11/12/1992 | 43.04 | 6.67 | 0 | 1 | 1 | 0 | 0 | 0 |
| 11/13/1992 | 42.76 | 6.67 | 1 | 7 | 5 | 0 | 0 | 0 |
| 11/14/1992 | 40.49 | 6.11 | 0 | 2 | 1 | 0 | 0 | 0 |
| 11/15/1992 | 36.53 | 6.67 | 1 | 0 | 0 | 0 | 0 | 0 |
| 11/16/1992 | 34.26 | 6.67 | 0 | 8 | 4 | 0 | 0 | 0 |
| 11/17/1992 | 33.13 | 6.67 | 0 | 7 | 2 | 0 | 0 | 0 |
| 11/18/1992 | 29.73 | 7.22 | 1 | 9 | 8 | 0 | 0 | 0 |
| 11/19/1992 | 35.96 | 7.22 | 0 | 10 | 9 | 0 | 0 | 0 |
| 11/20/1992 | 66.83 | 6.67 | 0 | 17 | 9 | 0 | 0 | 0 |
| 11/21/1992 | 62.01 | 6.67 | 0 | 21 | 3 | 0 | 0 | 0 |
| 11/22/1992 | 382.28 | 7.22 | 0 | 20 | 4 | 0 | 0 | 0 |
| 11/23/1992 | 214.08 | 7.22 | 0 | 91 | 9 | 0 | 0 | 0 |
| 11/24/1992 | 116.95 | 5.56 | 0 | 19 | 0 | 0 | 0 | 0 |
| 11/25/1992 | 81.27 | 6.11 | 0 | 10 | 1 | 0 | 0 | 0 |
| 11/26/1992 | 67.11 | 6.11 | 0 | 14 | 2 | 0 | 0 | 0 |
| 11/27/1992 | 64.00 | 6.67 | 0 | 14 | 9 | 0 | 0 | 0 |
| 11/28/1992 | 102.51 | 6.67 | 0 | 19 | 11 | 0 | 0 | 0 |
| 11/29/1992 | 94.01 | 6.11 | 0 | 3 | 4 | 0 | 0 | 0 |
| 11/30/1992 | 74.76 | 5.56 | 0 | 1 | 0 | 0 | 0 | 0 |
| 12/1/1992 | 82.97 | 6.11 | 0 | 3 | 4 | 0 | 0 | 0 |

| Date | Discharge (cms) | Temp (C°) | Chinook | Coho | Steelhead | Cutthroat | Lamprey | Sucker |
|---|---|---|---|---|---|---|---|---|
| 12/2/1992 | 177.83 | 7.22 | 0 | 4 | 14 | 0 | 0 | 0 |
| 12/3/1992 | 197.65 | 6.11 | 0 | 3 | 0 | 0 | 0 | 0 |
| 12/4/1992 | 124.59 | 5.56 | 0 | 0 | 0 | 0 | 0 | 0 |
| 12/5/1992 | 95.99 | 3.89 | 0 | 2 | 0 | 0 | 0 | 0 |
| 12/6/1992 | 79.57 | 4.44 | 0 | 0 | 2 | 0 | 0 | 0 |
| 12/7/1992 | 83.25 | 5.00 | 0 | 0 | 3 | 0 | 0 | 0 |
| 12/8/1992 | 96.28 | 5.56 | 0 | 1 | 6 | 0 | 0 | 0 |
| 12/9/1992 | 314.32 | 6.11 | 1 | 7 | 13 | 0 | 0 | 0 |
| 12/10/1992 | 336.97 | 7.22 | 0 | 4 | 15 | 0 | 0 | 0 |
| 12/11/1992 | 342.63 | 6.11 | 0 | 17 | 13 | 0 | 0 | 0 |
| 12/12/1992 | 212.38 | | 0 | 0 | 5 | 0 | 0 | 0 |
| 12/13/1992 | 156.31 | | 0 | 0 | 0 | 0 | 0 | 0 |
| 12/14/1992 | 125.44 | 5.00 | 0 | 1 | 7 | 0 | 0 | 0 |
| 12/15/1992 | 117.23 | 5.56 | 0 | 0 | 15 | 0 | 0 | 0 |
| 12/16/1992 | 109.02 | 5.00 | 0 | 0 | 4 | 0 | 0 | 0 |
| 12/17/1992 | 138.19 | 4.44 | 0 | 0 | 1 | 0 | 0 | 0 |
| 12/18/1992 | 126.01 | 4.44 | 0 | 0 | 4 | 0 | 0 | 0 |
| 12/19/1992 | 106.19 | 4.44 | 1 | 0 | 3 | 0 | 0 | 0 |
| 12/20/1992 | 99.11 | 5.00 | 0 | 0 | 8 | 0 | 0 | 0 |
| 12/21/1992 | 127.43 | 6.11 | 0 | 0 | 32 | 0 | 0 | 0 |
| 12/22/1992 | 184.06 | 6.11 | 0 | 1 | 29 | 0 | 0 | 0 |
| 12/23/1992 | 148.66 | 6.11 | 0 | 1 | 15 | 0 | 0 | 0 |
| 12/24/1992 | 118.93 | 5.56 | 0 | 0 | 6 | 0 | 0 | 0 |
| 12/25/1992 | 107.60 | 5.00 | 0 | 0 | 4 | 0 | 0 | 0 |
| 12/26/1992 | 97.69 | 4.44 | 0 | 0 | 3 | 0 | 0 | 0 |
| 12/27/1992 | 142.72 | 5.00 | 0 | 0 | 24 | 0 | 0 | 0 |
| 12/28/1992 | 263.63 | 5.56 | 0 | 0 | 13 | 0 | 0 | 0 |
| 12/29/1992 | 234.75 | 5.56 | 0 | 0 | 22 | 0 | 0 | 0 |
| 12/30/1992 | 171.60 | 4.44 | 1 | 1 | 1 | 0 | 0 | 0 |
| 12/31/1992 | 184.06 | 5.56 | 0 | 1 | 16 | 0 | 0 | 0 |
| 1/1/1993 | 212.38 | 5.00 | 0 | 0 | 16 | 0 | 0 | 0 |
| 1/2/1993 | 155.74 | 4.44 | 0 | 0 | 21 | 0 | 0 | 0 |
| 1/3/1993 | 113.27 | 4.44 | 0 | 0 | 9 | 0 | 0 | 0 |
| 1/4/1993 | 120.35 | 4.44 | 0 | 0 | 8 | 0 | 0 | 0 |
| 1/5/1993 | 141.58 | 3.89 | 0 | 0 | 1 | 0 | 0 | 0 |
| 1/6/1993 | 113.27 | 3.89 | 0 | 0 | 1 | 0 | 0 | 0 |
| 1/7/1993 | 94.86 | 3.33 | 0 | 0 | 0 | 0 | 0 | 0 |
| 1/8/1993 | 109.59 | 3.89 | 0 | 0 | 17 | 0 | 0 | 0 |
| 1/9/1993 | 108.17 | 4.44 | 0 | 0 | 8 | 0 | 0 | 0 |
| 1/10/1993 | 94.86 | 3.89 | 0 | 0 | 3 | 0 | 0 | 0 |
| 1/11/1993 | 82.69 | 3.89 | 0 | 0 | 2 | 0 | 0 | 0 |
| 1/12/1993 | 77.02 | 3.33 | 0 | 0 | 2 | 0 | 0 | 0 |
| 1/13/1993 | 71.36 | 3.33 | 0 | 0 | 1 | 0 | 0 | 0 |
| 1/14/1993 | 79.57 | 3.89 | 0 | 0 | 12 | 0 | 0 | 0 |
| 1/15/1993 | 102.79 | 3.89 | 0 | 0 | 14 | 0 | 0 | 0 |

| Date | Discharge (cms) | Temp (C°) | Chinook | Coho | Steelhead | Cutthroat | Lamprey | Sucker |
|---|---|---|---|---|---|---|---|---|
| 1/16/1993 | 101.09 | 5.00 | 0 | 0 | 11 | 0 | 0 | 0 |
| 1/17/1993 | 94.86 | 4.44 | 0 | 0 | 23 | 0 | 0 | 0 |
| 1/18/1993 | 91.75 | 3.89 | 0 | 0 | 6 | 0 | 0 | 0 |
| 1/19/1993 | 85.80 | 4.44 | 0 | 0 | 28 | 0 | 0 | 0 |
| 1/20/1993 | 328.48 | 5.56 | 0 | 0 | 81 | 0 | 0 | 0 |
| 1/21/1993 | 436.08 | 6.11 | 0 | 0 | 15 | 0 | 0 | 0 |
| 1/22/1993 | 430.42 | | 0 | 0 | 2 | 0 | 0 | 0 |
| 1/23/1993 | 302.99 | | 0 | 0 | 54 | 0 | 0 | 0 |
| 1/24/1993 | 196.52 | | 0 | 0 | 62 | 0 | 0 | 0 |
| 1/25/1993 | 170.47 | 6.67 | 0 | 0 | 13 | 0 | 0 | 0 |
| 1/26/1993 | 176.13 | 5.56 | 0 | 0 | 10 | 0 | 0 | 0 |
| 1/27/1993 | 169.05 | 5.56 | 0 | 0 | 8 | 0 | 0 | 0 |
| 1/28/1993 | 162.82 | 6.11 | 0 | 0 | 49 | 0 | 0 | 0 |
| 1/29/1993 | 156.88 | 6.11 | 0 | 0 | 32 | 0 | 0 | 0 |
| 1/30/1993 | 141.02 | | 0 | 0 | 8 | 0 | 0 | 0 |
| 1/31/1993 | 130.54 | | 0 | 0 | 24 | 0 | 0 | 0 |
| 2/1/1993 | 122.05 | 4.44 | 0 | 0 | 2 | 0 | 0 | 0 |
| 2/2/1993 | 115.25 | 5.00 | 0 | 0 | 4 | 0 | 0 | 0 |
| 2/3/1993 | 119.78 | 6.11 | 0 | 0 | 46 | 0 | 0 | 0 |
| 2/4/1993 | 137.90 | 6.11 | 0 | 0 | 27 | 0 | 0 | 0 |
| 2/5/1993 | 165.94 | 6.67 | 0 | 0 | 68 | 0 | 0 | 0 |
| 2/6/1993 | 193.97 | 6.67 | 0 | 0 | 60 | 0 | 0 | 0 |
| 2/7/1993 | 169.62 | 6.67 | 0 | 0 | 19 | 0 | 0 | 0 |
| 2/8/1993 | 157.16 | 6.11 | 0 | 0 | 17 | 0 | 0 | 0 |
| 2/9/1993 | 161.41 | 7.22 | 0 | 0 | 49 | 0 | 0 | 0 |
| 2/10/1993 | 154.04 | 6.11 | 0 | 0 | 10 | 0 | 0 | 0 |
| 2/11/1993 | 142.15 | 6.11 | 0 | 0 | 12 | 0 | 0 | 0 |
| 2/12/1993 | 143.28 | 6.11 | 0 | 0 | 33 | 0 | 0 | 0 |
| 2/13/1993 | 144.42 | 5.56 | 0 | 0 | 6 | 0 | 0 | 0 |
| 2/14/1993 | 127.71 | 5.56 | 0 | 0 | 11 | 0 | 0 | 0 |
| 2/15/1993 | 111.00 | 3.89 | 0 | 0 | 5 | 0 | 0 | 0 |
| 2/16/1993 | 95.99 | 3.33 | 0 | 0 | 0 | 0 | 0 | 0 |
| 2/17/1993 | 86.65 | 2.78 | 0 | 0 | 0 | 0 | 0 | 0 |
| 2/18/1993 | 81.27 | 3.33 | 0 | 0 | 0 | 0 | 0 | 0 |
| 2/19/1993 | 104.77 | 3.33 | 0 | 0 | 6 | 0 | 0 | 0 |
| 2/20/1993 | 175.56 | 3.89 | 0 | 0 | 5 | 0 | 0 | 0 |
| 2/21/1993 | 161.97 | 3.89 | 0 | 0 | 5 | 0 | 0 | 0 |
| 2/22/1993 | 155.46 | 4.44 | 0 | 0 | 8 | 0 | 0 | 0 |
| 2/23/1993 | 149.23 | 3.89 | 0 | 0 | 16 | 0 | 0 | 0 |
| 2/24/1993 | 129.41 | 4.44 | 0 | 0 | 12 | 0 | 0 | 0 |
| 2/25/1993 | 109.30 | 4.44 | 0 | 0 | 4 | 0 | 0 | 0 |
| 2/26/1993 | 97.69 | 3.89 | 0 | 0 | 0 | 0 | 0 | 0 |
| 2/27/1993 | 89.48 | 3.89 | 0 | 0 | 0 | 0 | 0 | 0 |
| 2/28/1993 | 83.53 | 4.44 | 0 | 0 | 4 | 0 | 0 | 0 |
| 3/1/1993 | 86.08 | 3.89 | 0 | 0 | 8 | 0 | 0 | 0 |

| Date | Discharge (cms) | Temp (C°) | Chinook | Coho | Steelhead | Cutthroat | Lamprey | Sucker |
|---|---|---|---|---|---|---|---|---|
| 3/2/1993 | 102.79 | 5.56 | 0 | 0 | 33 | 0 | 0 | 0 |
| 3/3/1993 | 156.88 | 6.11 | 0 | 0 | 296 | 0 | 0 | 0 |
| 3/4/1993 | 208.70 | 6.11 | 0 | 0 | 159 | 0 | 0 | 0 |
| 3/5/1993 | 222.00 | 7.22 | 0 | 0 | 66 | 0 | 0 | 0 |
| 3/6/1993 | 207.00 | 7.78 | 0 | 0 | 77 | 0 | 0 | 0 |
| 3/7/1993 | 210.68 | 7.22 | 0 | 0 | 86 | 0 | 0 | 0 |
| 3/8/1993 | 206.15 | 7.22 | 0 | 0 | 34 | 0 | 0 | 0 |
| 3/9/1993 | 195.39 | 7.78 | 0 | 0 | 47 | 0 | 0 | 7 |
| 3/10/1993 | 191.71 | 7.78 | 0 | 0 | 53 | 0 | 0 | 3 |
| 3/11/1993 | 199.63 | 8.33 | 0 | 0 | 39 | 0 | 0 | 4 |
| 3/12/1993 | 171.03 | 7.78 | 0 | 0 | 28 | 0 | 0 | 0 |
| 3/13/1993 | 152.06 | 7.78 | 0 | 0 | 34 | 0 | 0 | 0 |
| 3/14/1993 | 150.65 | 8.33 | 0 | 0 | 66 | 0 | 0 | 1 |
| 3/15/1993 | 230.22 | 7.78 | 0 | 0 | 255 | 0 | 0 | 18 |
| 3/16/1993 | 549.35 | 7.22 | 0 | 0 | 0 | 0 | 0 | 0 |
| 3/17/1993 | 671.11 | 7.78 | 0 | 0 | 0 | 0 | 0 | 0 |
| 3/18/1993 | 707.92 | 7.78 | 0 | 0 | 0 | 0 | 0 | 0 |
| 3/19/1993 | 586.16 | 7.78 | 0 | 0 | 1 | 0 | 0 | 0 |
| 3/20/1993 | 416.26 | 8.33 | 0 | 0 | 100 | 0 | 0 | 0 |
| 3/21/1993 | 325.64 | 7.78 | 0 | 0 | 29 | 0 | 0 | 0 |
| 3/22/1993 | 255.98 | 7.78 | 0 | 0 | 37 | 0 | 0 | 0 |
| 3/23/1993 | 441.74 | 8.33 | 0 | 0 | 46 | 0 | 0 | 0 |
| 3/24/1993 | 484.22 | 7.78 | 0 | 0 | 2 | 0 | 0 | 0 |
| 3/25/1993 | 325.64 | 7.22 | 0 | 0 | 5 | 0 | 0 | 0 |
| 3/26/1993 | 246.36 | 7.78 | 0 | 0 | 0 | 0 | 0 | 0 |
| 3/27/1993 | 193.97 | 7.78 | 0 | 0 | 135 | 0 | 0 | 0 |
| 3/28/1993 | 163.95 | 7.78 | 0 | 0 | 15 | 0 | 0 | 0 |
| 3/29/1993 | 144.42 | 9.44 | 0 | 0 | 110 | 0 | 0 | 0 |
| 3/30/1993 | 133.09 | 8.89 | 1 | 0 | 83 | 0 | 0 | 4 |
| 3/31/1993 | 124.59 | 8.33 | 2 | 0 | 41 | 0 | 0 | 1 |
| 4/1/1993 | 132.24 | 7.78 | 1 | 0 | 29 | 0 | 0 | 0 |
| 4/2/1993 | 158.86 | 7.78 | 0 | 0 | 50 | 0 | 0 | 0 |
| 4/3/1993 | 399.27 | 7.78 | 0 | 0 | 49 | 0 | 0 | 0 |
| 4/4/1993 | 549.35 | 7.78 | 0 | 0 | 0 | 0 | 0 | 0 |
| 4/5/1993 | 416.26 | 7.78 | 0 | 0 | 48 | 0 | 0 | 0 |
| 4/6/1993 | 305.82 | 7.78 | 1 | 0 | 44 | 0 | 0 | 0 |
| 4/7/1993 | 259.67 | 8.89 | 0 | 0 | 52 | 0 | 0 | 0 |
| 4/8/1993 | 245.22 | 9.44 | 2 | 0 | 133 | 1 | 0 | 20 |
| 4/9/1993 | 322.81 | 8.33 | 1 | 0 | 20 | 0 | 0 | 0 |
| 4/10/1993 | 387.94 | 7.78 | 0 | 0 | 2 | 0 | 0 | 0 |
| 4/11/1993 | 376.61 | 7.78 | 0 | 0 | 1 | 0 | 0 | 0 |
| 4/12/1993 | 305.82 | 7.78 | 0 | 0 | 0 | 0 | 0 | 0 |
| 4/13/1993 | 246.92 | 7.78 | 1 | 0 | 11 | 0 | 0 | 0 |
| 4/14/1993 | 213.79 | 8.33 | 1 | 0 | 5 | 0 | 0 | 0 |
| 4/15/1993 | 197.09 | 8.89 | 0 | 0 | 25 | 0 | 0 | 5 |

| Date | Discharge (cms) | Temp (C°) | Chinook | Coho | Steelhead | Cutthroat | Lamprey | Sucker |
|---|---|---|---|---|---|---|---|---|
| 4/16/1993 | 187.74 | 7.78 | 0 | 0 | 30 | 0 | 0 | 1 |
| 4/17/1993 | 193.12 | 8.33 | 1 | 0 | 29 | 0 | 0 | 3 |
| 4/18/1993 | 288.83 | 7.78 | 2 | 0 | 23 | 0 | 0 | 0 |
| 4/19/1993 | 291.66 | 8.33 | 0 | 0 | 14 | 0 | 0 | 0 |
| 4/20/1993 | 228.23 | 8.33 | 3 | 0 | 25 | 0 | 0 | 0 |
| 4/21/1993 | 190.29 | 9.44 | 2 | 0 | 21 | 0 | 0 | 77 |
| 4/22/1993 | 166.22 | 8.89 | 8 | 0 | 27 | 0 | 0 | 46 |
| 4/23/1993 | 168.77 | 8.33 | 4 | 0 | 7 | 0 | 0 | 5 |
| 4/24/1993 | 208.13 | 8.33 | 19 | 0 | 20 | 0 | 0 | 4 |
| 4/25/1993 | 214.64 | 8.33 | 14 | 0 | 9 | 0 | 0 | 12 |
| 4/26/1993 | 217.47 | 8.33 | 2 | 0 | 2 | 0 | 0 | 0 |
| 4/27/1993 | 193.40 | 10.00 | 3 | 0 | 7 | 0 | 0 | 13 |
| 4/28/1993 | 169.90 | 9.44 | 22 | 0 | 19 | 0 | 0 | 113 |
| 4/29/1993 | 153.76 | 10.56 | 20 | 0 | 18 | 0 | 0 | 862 |
| 4/30/1993 | 154.89 | 11.11 | 59 | 0 | 19 | 1 | 4 | 735 |
| 5/1/1993 | 144.13 | 10.00 | 42 | 0 | 6 | 0 | 0 | 33 |
| 5/2/1993 | 135.07 | 11.11 | 8 | 0 | 2 | 0 | 0 | 82 |
| 5/3/1993 | 153.19 | 11.11 | 101 | 0 | 18 | 0 | 0 | 416 |
| 5/4/1993 | 244.09 | 8.89 | 10 | 0 | 2 | 0 | 0 | 6 |
| 5/5/1993 | 209.26 | 9.44 | 0 | 0 | 0 | 0 | 0 | 0 |
| 5/6/1993 | 204.45 | 10.56 | 0 | 0 | 0 | 0 | 0 | 0 |
| 5/7/1993 | 244.09 | 8.33 | 0 | 0 | 0 | 0 | 0 | 0 |
| 5/8/1993 | 274.96 | 9.44 | 0 | 0 | 0 | 0 | 0 | 0 |
| 5/9/1993 | 265.90 | 10.56 | 0 | 0 | 0 | 0 | 0 | 0 |
| 5/10/1993 | 220.31 | 11.11 | 30 | 0 | 9 | 0 | 0 | 112 |
| 5/11/1993 | 188.02 | 11.11 | 42 | 0 | 8 | 0 | 0 | 13 |
| 5/12/1993 | 164.52 | 11.11 | 55 | 0 | 6 | 0 | 0 | 71 |
| 5/13/1993 | 148.10 | 11.11 | 249 | 0 | 5 | 0 | 0 | 10 |
| 5/14/1993 | 131.96 | 11.67 | 118 | 0 | 5 | 0 | 0 | 2 |
| 5/15/1993 | 130.82 | 12.78 | 93 | 0 | 0 | 0 | 0 | 183 |
| 5/16/1993 | 124.59 | 13.89 | 76 | 0 | 3 | 0 | 0 | 94 |
| 5/17/1993 | 124.31 | 13.89 | 159 | 0 | 13 | 0 | 0 | 410 |
| 5/18/1993 | 125.44 | 13.33 | 164 | 0 | 8 | 0 | 0 | 168 |
| 5/19/1993 | 127.14 | 13.89 | 150 | 0 | 5 | 0 | 0 | 87 |
| 5/20/1993 | 145.83 | 12.78 | 47 | 0 | 0 | 0 | 0 | 22 |
| 5/21/1993 | 143.28 | 11.11 | 65 | 0 | 4 | 0 | 0 | 20 |
| 5/22/1993 | 137.90 | 12.78 | 19 | 0 | 0 | 0 | 0 | 6 |
| 5/23/1993 | 127.71 | 13.89 | 23 | 0 | 4 | 0 | 0 | 0 |
| 5/24/1993 | 119.50 | 13.33 | 60 | 0 | 1 | 0 | 0 | 194 |
| 5/25/1993 | 131.67 | 12.22 | 93 | 0 | 1 | 0 | 0 | 210 |
| 5/26/1993 | 154.33 | 11.11 | 82 | 0 | 3 | 0 | 0 | 4 |
| 5/27/1993 | 146.11 | 11.11 | 82 | 0 | 2 | 0 | 0 | 21 |
| 5/28/1993 | 140.45 | 11.11 | 85 | 0 | 3 | 0 | 0 | 17 |
| 5/29/1993 | 132.52 | 11.67 | 39 | 0 | 3 | 0 | 0 | 52 |
| 5/30/1993 | 123.46 | | 107 | 0 | 12 | 0 | 0 | 112 |

| Date | Discharge (cms) | Temp (C°) | Chinook | Coho | Steelhead | Cutthroat | Lamprey | Sucker |
|---|---|---|---|---|---|---|---|---|
| 5/31/1993 | 236.45 | 11.67 | 37 | 0 | 3 | 0 | 149 | 23 |
| 6/1/1993 | 311.49 | 10.56 | 8 | 0 | 0 | 0 | 6 | 4 |
| 6/2/1993 | 277.22 | 10.56 | 10 | 0 | 0 | 0 | 0 | 1 |
| 6/3/1993 | 261.93 | 10.56 | 5 | 0 | 2 | 0 | 0 | 2 |
| 6/4/1993 | 227.38 | 10.56 | 26 | 0 | 2 | 0 | 0 | 9 |
| 6/5/1993 | 279.77 | 10.00 | 5 | 0 | 1 | 0 | 11 | 0 |
| 6/6/1993 | 270.71 | 10.00 | 13 | 0 | 0 | 0 | 0 | 2 |
| 6/7/1993 | 231.63 | 10.56 | 26 | 0 | 0 | 0 | 1 | 1 |
| 6/8/1993 | 210.68 | 11.11 | 68 | 0 | 13 | 0 | 27 | 10 |
| 6/9/1993 | 179.53 | 11.11 | 116 | 0 | 14 | 0 | 6 | 66 |
| 6/10/1993 | 157.44 | 11.67 | 52 | 0 | 11 | 0 | 19 | 259 |
| 6/11/1993 | 139.89 | 12.22 | 129 | 0 | 21 | 0 | 11 | 466 |
| 6/12/1993 | 137.34 | 11.67 | 12 | 0 | 2 | 0 | 0 | 61 |
| 6/13/1993 | 124.31 | 12.78 | 26 | 0 | 10 | 0 | 10 | 623 |
| 6/14/1993 | 110.44 | 13.89 | 98 | 0 | 25 | 0 | 2 | 1717 |
| 6/15/1993 | 101.66 | 13.89 | 167 | 0 | 48 | 0 | 13 | 1470 |
| 6/16/1993 | 96.84 | 14.44 | 6 | 0 | 3 | 0 | 0 | 43 |
| 6/17/1993 | 91.18 | 15.56 | 17 | 0 | 2 | 0 | 0 | 1336 |
| 6/18/1993 | 87.22 | 16.67 | 30 | 0 | 7 | 0 | 0 | 1476 |
| 6/19/1993 | 83.82 | 15.56 | 0 | 0 | 0 | 0 | 0 | 0 |
| 6/20/1993 | 82.12 | 17.22 | 25 | 0 | 20 | 0 | 0 | 725 |
| 6/21/1993 | 79.85 | 15.00 | 51 | 0 | 5 | 0 | 0 | 36 |
| 6/22/1993 | 76.74 | 14.44 | 31 | 0 | 49 | 0 | 0 | 39 |
| 6/23/1993 | 73.34 | 14.44 | 33 | 0 | 63 | 0 | 0 | 14 |
| 6/24/1993 | 66.26 | 15.00 | 45 | 0 | 70 | 0 | 10 | 1477 |
| 6/25/1993 | 62.86 | 16.67 | 108 | 0 | 76 | 0 | 24 | 2152 |
| 6/26/1993 | 61.45 | 17.22 | 130 | 0 | 69 | 0 | 44 | 893 |
| 6/27/1993 | 61.16 | 16.11 | 78 | 0 | 37 | 0 | 2 | 15 |
| 6/28/1993 | 59.75 | 16.11 | 0 | 0 | 0 | 0 | 0 | 0 |
| 6/29/1993 | 61.16 | 16.11 | 0 | 0 | 0 | 0 | 0 | 0 |
| 6/30/1993 | 73.34 | 16.11 | 67 | 0 | 80 | 0 | 0 | 427 |
| 7/1/1993 | 59.18 | 15.00 | 62 | 0 | 111 | 0 | 0 | 35 |
| 7/2/1993 | 48.99 | 16.11 | 22 | 0 | 63 | 0 | 0 | 57 |
| 7/3/1993 | 45.59 | 17.22 | 15 | 0 | 22 | 0 | 0 | 438 |
| 7/4/1993 | 45.31 | 17.22 | 18 | 0 | 38 | 0 | 0 | 367 |
| 7/5/1993 | 44.74 | 16.67 | 29 | 0 | 79 | 0 | 2 | 18 |
| 7/6/1993 | 43.89 | | 0 | 0 | 0 | 0 | 0 | 0 |
| 7/7/1993 | 43.04 | 17.22 | 15 | 0 | 44 | 0 | 0 | 76 |
| 7/8/1993 | 43.32 | 17.78 | 26 | 0 | 72 | 0 | 0 | 540 |
| 7/9/1993 | 43.89 | 17.78 | 15 | 0 | 50 | 0 | 0 | 239 |
| 7/10/1993 | 43.89 | 18.33 | 41 | 0 | 73 | 0 | 0 | 136 |
| 7/11/1993 | 43.89 | 17.22 | 13 | 0 | 30 | 0 | 2 | 1 |
| 7/12/1993 | 43.89 | 16.67 | 51 | 0 | 143 | 0 | 0 | 418 |
| 7/13/1993 | 41.63 | 16.11 | 0 | 0 | 0 | 0 | 0 | 0 |
| 7/14/1993 | 39.36 | 15.56 | 5 | 0 | 29 | 0 | 0 | 0 |

| Date | Discharge (cms) | Temp (C°) | Chinook | Coho | Steelhead | Cutthroat | Lamprey | Sucker |
|---|---|---|---|---|---|---|---|---|
| 7/15/1993 | 39.36 | 16.11 | 10 | 0 | 77 | 0 | 0 | 0 |
| 7/16/1993 | 41.91 | 16.67 | 18 | 0 | 68 | 0 | 0 | 179 |
| 7/17/1993 | 41.91 | 16.67 | 18 | 0 | 39 | 0 | 1 | 91 |
| 7/18/1993 | 38.23 | 15.56 | 7 | 0 | 25 | 0 | 0 | 0 |
| 7/19/1993 | 38.51 | 16.11 | 15 | 0 | 67 | 0 | 0 | 0 |
| 7/20/1993 | 39.93 | 16.11 | 16 | 0 | 41 | 0 | 0 | 1 |
| 7/21/1993 | 39.64 | 16.11 | 24 | 0 | 85 | 0 | 5 | 15 |
| 7/22/1993 | 43.32 | 15.00 | 22 | 0 | 63 | 0 | 0 | 59 |
| 7/23/1993 | 83.25 | 13.89 | 54 | 0 | 71 | 0 | 0 | 304 |
| 7/24/1993 | 71.92 | 15.56 | 17 | 0 | 28 | 0 | 0 | 508 |
| 7/25/1993 | 53.80 | 15.00 | 33 | 0 | 53 | 0 | 0 | 221 |
| 7/26/1993 | 46.72 | 16.67 | 9 | 0 | 25 | 0 | 1 | 8 |
| 7/27/1993 | 44.17 | 17.78 | 35 | 0 | 53 | 0 | 16 | 10 |
| 7/28/1993 | 41.63 | 16.67 | 44 | 0 | 45 | 0 | 0 | 9 |
| 7/29/1993 | 42.76 | 17.22 | 19 | 0 | 22 | 2 | 0 | 0 |
| 7/30/1993 | 40.21 | 17.78 | 17 | 0 | 29 | 0 | 0 | 0 |
| 7/31/1993 | 39.08 | 18.33 | 32 | 0 | 33 | 1 | 0 | 1 |
| 8/1/1993 | 39.64 | 18.89 | 27 | 0 | 21 | 0 | 0 | 10 |
| 8/2/1993 | 37.10 | 20.00 | 56 | 0 | 27 | 2 | 42 | 22 |
| 8/3/1993 | 36.53 | 21.11 | 59 | 0 | 46 | 0 | 26 | 14 |
| 8/4/1993 | 37.38 | 21.11 | 38 | 0 | 20 | 15 | 11 | 3 |
| 8/5/1993 | 37.38 | 20.56 | 28 | 0 | 15 | 2 | 27 | 2 |
| 8/6/1993 | 35.40 | 19.44 | 15 | 0 | 10 | 2 | 0 | 0 |
| 8/7/1993 | 33.13 | 18.89 | 2 | 0 | 0 | 0 | 0 | 2 |
| 8/8/1993 | 33.98 | 19.44 | 0 | 0 | 2 | 0 | 0 | 1 |
| 8/9/1993 | 33.98 | 19.44 | 1 | 0 | 3 | 0 | 0 | 1 |
| 8/10/1993 | 32.28 | 18.89 | 4 | 0 | 4 | 0 | 0 | 0 |
| 8/11/1993 | 32.28 | 18.89 | 4 | 0 | 2 | 0 | 0 | 0 |
| 8/12/1993 | 29.73 | 18.89 | 2 | 0 | 2 | 0 | 0 | 0 |
| 8/13/1993 | 29.73 | 17.22 | 14 | 0 | 9 | 0 | 0 | 0 |
| 8/14/1993 | 32.00 | 16.11 | 1 | 0 | 11 | 0 | 0 | 0 |
| 8/15/1993 | 33.13 | 16.67 | 2 | 0 | 8 | 0 | 0 | 0 |
| 8/16/1993 | 40.78 | 16.11 | 18 | 0 | 10 | 0 | 0 | 0 |
| 8/17/1993 | 38.23 | 16.11 | 2 | 0 | 7 | 0 | 0 | 0 |
| 8/18/1993 | 35.11 | 17.22 | 13 | 0 | 13 | 0 | 0 | 0 |
| 8/19/1993 | 36.81 | 16.67 | 39 | 0 | 21 | 0 | 0 | 0 |
| 8/20/1993 | 36.81 | 15.56 | 16 | 0 | 13 | 0 | 0 | 0 |
| 8/21/1993 | 42.48 | 16.67 | 10 | 0 | 7 | 0 | 0 | 0 |
| 8/22/1993 | 39.08 | 17.78 | 14 | 0 | 10 | 0 | 0 | 0 |
| 8/23/1993 | 35.40 | 17.22 | 33 | 0 | 25 | 0 | 0 | 0 |
| 8/24/1993 | 34.55 | 16.67 | 3 | 0 | 15 | 0 | 0 | 0 |
| 8/25/1993 | 32.28 | 16.11 | 7 | 0 | 7 | 0 | 0 | 0 |
| 8/26/1993 | 33.70 | 16.11 | 11 | 0 | 6 | 0 | 0 | 0 |
| 8/27/1993 | 31.71 | 16.11 | 10 | 0 | 14 | 0 | 0 | 0 |
| 8/28/1993 | 30.87 | 16.11 | 1 | 0 | 14 | 0 | 0 | 0 |

| Date | Discharge (cms) | Temp (C°) | Chinook | Coho | Steelhead | Cutthroat | Lamprey | Sucker |
|---|---|---|---|---|---|---|---|---|
| 8/29/1993 | 30.30 | 16.11 | 10 | 0 | 6 | 0 | 0 | 0 |
| 8/30/1993 | 30.02 | 16.67 | 14 | 0 | 12 | 0 | 0 | 0 |
| 8/31/1993 | 29.17 | 17.22 | 14 | 0 | 16 | 1 | 0 | 0 |
| 9/1/1993 | 29.73 | 17.22 | 35 | 0 | 6 | 0 | 0 | 0 |
| 9/2/1993 | 31.15 | 17.22 | 20 | 0 | 7 | 0 | 0 | 0 |
| 9/3/1993 | 29.73 | 17.78 | 8 | 1 | 10 | 0 | 0 | 0 |
| 9/4/1993 | 27.58 | 17.78 | 5 | 0 | 10 | 0 | 0 | 0 |
| 9/5/1993 | 25.97 | 16.67 | 7 | 0 | 15 | 0 | 0 | 0 |
| 9/6/1993 | 26.84 | 17.22 | 2 | 0 | 7 | 0 | 0 | 0 |
| 9/7/1993 | 27.55 | 18.33 | 10 | 0 | 6 | 0 | 0 | 0 |
| 9/8/1993 | 26.96 | 18.89 | 4 | 10 | 2 | 0 | 0 | 0 |
| 9/9/1993 | 26.45 | 18.89 | 13 | 5 | 2 | 0 | 0 | 0 |
| 9/10/1993 | 26.16 | 18.89 | 10 | 4 | 1 | 0 | 0 | 0 |
| 9/11/1993 | 26.36 | 17.78 | 14 | 2 | 5 | 0 | 0 | 0 |
| 9/12/1993 | 27.21 | 16.67 | 0 | 1 | 0 | 0 | 0 | 0 |
| 9/13/1993 | 27.16 | 16.11 | 0 | 0 | 0 | 0 | 0 | 0 |
| 9/14/1993 | 28.60 | 15.56 | 0 | 0 | 4 | 0 | 0 | 0 |
| 9/15/1993 | 29.45 | 15.56 | 0 | 0 | 8 | 0 | 0 | 0 |
| 9/16/1993 | 26.16 | 15.00 | 2 | 2 | 1 | 0 | 0 | 0 |
| 9/17/1993 | 23.76 | 15.00 | 0 | 0 | 0 | 0 | 0 | 0 |
| 9/18/1993 | 24.75 | 14.44 | 0 | 0 | 0 | 0 | 0 | 0 |
| 9/19/1993 | 24.55 | 13.33 | 0 | 0 | 4 | 0 | 0 | 0 |
| 9/20/1993 | 28.29 | 12.78 | 0 | 0 | 0 | 0 | 0 | 0 |
| 9/21/1993 | 28.88 | 12.78 | 0 | 0 | 0 | 0 | 0 | 0 |
| 9/22/1993 | 28.23 | 12.78 | 0 | 0 | 0 | 0 | 0 | 0 |
| 9/23/1993 | 26.79 | 12.22 | 0 | 0 | 0 | 0 | 0 | 0 |
| 9/24/1993 | 27.44 | 12.22 | 0 | 0 | 0 | 0 | 0 | 0 |
| 9/25/1993 | 26.19 | 12.78 | 0 | 0 | 0 | 0 | 0 | 0 |
| 9/26/1993 | 24.52 | 13.33 | 2 | 4 | 11 | 0 | 0 | 0 |
| 9/27/1993 | 25.91 | 13.89 | 0 | 0 | 0 | 0 | 0 | 0 |
| 9/28/1993 | 27.30 | 13.89 | 3 | 13 | 0 | 0 | 0 | 0 |
| 9/29/1993 | 25.94 | 13.89 | 0 | 0 | 0 | 0 | 0 | 0 |
| 9/30/1993 | 26.96 | 13.89 | 3 | 24 | 4 | 0 | 0 | 0 |
| 10/1/1993 | 28.60 | 13.89 | 3 | 28 | 3 | 0 | 0 | 0 |
| 10/2/1993 | 28.60 | 13.33 | 0 | 34 | 1 | 0 | 0 | 0 |
| 10/3/1993 | 26.42 | 11.11 | 6 | 34 | 4 | 0 | 0 | 0 |
| 10/4/1993 | 26.19 | 12.78 | 4 | 28 | 14 | 0 | 0 | 0 |
| 10/5/1993 | 26.39 | 13.33 | 7 | 12 | 5 | 0 | 0 | 0 |
| 10/6/1993 | 26.31 | 12.22 | 5 | 13 | 2 | 0 | 0 | 0 |
| 10/7/1993 | 26.16 | 12.78 | 0 | 12 | 0 | 0 | 0 | 0 |
| 10/8/1993 | 25.60 | 12.22 | 2 | 10 | 3 | 0 | 0 | 0 |
| 10/9/1993 | 25.57 | 11.67 | 3 | 5 | 1 | 0 | 0 | 0 |
| 10/10/1993 | 25.34 | 12.22 | 2 | 3 | 2 | 0 | 0 | 0 |
| 10/11/1993 | 25.74 | 12.22 | 4 | 8 | 5 | 0 | 0 | 0 |
| 10/12/1993 | 28.32 | 12.22 | 6 | 33 | 7 | 0 | 0 | 0 |

| Date | Discharge (cms) | Temp (C°) | Chinook | Coho | Steelhead | Cutthroat | Lamprey | Sucker |
|---|---|---|---|---|---|---|---|---|
| 10/13/1993 | 27.67 | 12.78 | 3 | 23 | 8 | 0 | 0 | 0 |
| 10/14/1993 | 27.55 | 12.78 | 14 | 27 | 5 | 0 | 0 | 0 |
| 10/15/1993 | 28.26 | 12.22 | 4 | 27 | 8 | 0 | 0 | 0 |
| 10/16/1993 | 30.02 | 11.11 | 2 | 39 | 6 | 0 | 0 | 0 |
| 10/17/1993 | 32.56 | 11.11 | 0 | 15 | 3 | 0 | 0 | 0 |
| 10/18/1993 | 28.88 | 10.00 | 1 | 11 | 3 | 0 | 0 | 0 |
| 10/19/1993 | 26.84 | 9.44 | 0 | 5 | 2 | 0 | 0 | 0 |
| 10/20/1993 | 26.42 | 8.89 | 0 | 4 | 2 | 0 | 0 | 0 |
| 10/21/1993 | 25.43 | 10.00 | 1 | 4 | 2 | 0 | 0 | 0 |
| 10/22/1993 | 25.26 | 10.00 | 1 | 6 | 0 | 0 | 0 | 0 |
| 10/23/1993 | 24.69 | 9.44 | 1 | 1 | 0 | 0 | 0 | 0 |
| 10/24/1993 | 25.88 | 9.44 | 0 | 0 | 0 | 0 | 0 | 0 |
| 10/25/1993 | 24.98 | 8.89 | 0 | 0 | 0 | 0 | 0 | 0 |
| 10/26/1993 | 24.81 | 8.89 | 0 | 20 | 3 | 2 | 0 | 0 |
| 10/27/1993 | 24.66 | | 0 | 0 | 0 | 0 | 0 | 0 |
| 10/28/1993 | 24.49 | | 0 | 0 | 0 | 0 | 0 | 0 |
| 10/29/1993 | 23.90 | 8.33 | 0 | 0 | 0 | 0 | 0 | 0 |
| 10/30/1993 | 23.11 | 8.33 | 0 | 0 | 0 | 0 | 0 | 0 |
| 10/31/1993 | 23.62 | 8.89 | 0 | 11 | 7 | 0 | 0 | 0 |
| 11/1/1993 | 27.18 | 8.89 | 0 | 8 | 0 | 0 | 0 | 0 |
| 11/2/1993 | 27.55 | 8.33 | 0 | 16 | 2 | 0 | 0 | 0 |
| 11/3/1993 | 27.44 | 7.78 | 0 | 1 | 0 | 1 | 0 | 0 |
| 11/4/1993 | 27.21 | 7.78 | 0 | 0 | 0 | 0 | 0 | 0 |
| 11/5/1993 | 26.90 | 7.22 | 0 | 0 | 0 | 0 | 0 | 0 |
| 11/6/1993 | 24.64 | 7.22 | 0 | 0 | 0 | 0 | 0 | 0 |
| 11/7/1993 | 23.98 | 6.11 | 0 | 0 | 0 | 0 | 0 | 0 |
| 11/8/1993 | 24.32 | 5.56 | 0 | 0 | 1 | 0 | 0 | 0 |
| 11/9/1993 | 25.06 | 5.56 | 0 | 0 | 0 | 0 | 0 | 0 |
| 11/10/1993 | 24.81 | 5.56 | 0 | 0 | 0 | 0 | 0 | 0 |
| 11/11/1993 | 25.09 | 5.56 | 0 | 0 | 0 | 0 | 0 | 0 |
| 11/12/1993 | 24.81 | 6.11 | 0 | 0 | 0 | 0 | 0 | 0 |
| 11/13/1993 | 23.90 | 6.11 | 0 | 0 | 0 | 0 | 0 | 0 |
| 11/14/1993 | 23.79 | 5.00 | 0 | 0 | 0 | 0 | 0 | 0 |
| 11/15/1993 | 24.15 | 4.44 | 0 | 0 | 0 | 0 | 0 | 0 |
| 11/16/1993 | 23.33 | 5.00 | 0 | 6 | 0 | 0 | 0 | 0 |
| 11/17/1993 | 25.06 | 5.00 | 0 | 0 | 0 | 0 | 0 | 0 |
| 11/18/1993 | 25.32 | 5.00 | 0 | 0 | 0 | 0 | 0 | 0 |
| 11/19/1993 | 24.89 | 5.00 | 0 | 0 | 0 | 0 | 0 | 0 |
| 11/20/1993 | 24.38 | 5.00 | 0 | 0 | 0 | 0 | 0 | 0 |
| 11/21/1993 | 24.04 | 3.89 | 0 | 0 | 0 | 0 | 0 | 0 |
| 11/22/1993 | 26.05 | 3.33 | 0 | 0 | 0 | 0 | 0 | 0 |
| 11/23/1993 | 29.17 | 3.33 | 0 | 0 | 0 | 0 | 0 | 0 |
| 11/24/1993 | 29.17 | 2.78 | 0 | 0 | 0 | 0 | 0 | 0 |
| 11/25/1993 | 28.03 | 2.22 | 0 | 0 | 0 | 0 | 0 | 0 |
| 11/26/1993 | 24.44 | 2.22 | 0 | 0 | 0 | 0 | 0 | 0 |

| Date | Discharge (cms) | Temp (C°) | Chinook | Coho | Steelhead | Cutthroat | Lamprey | Sucker |
|---|---|---|---|---|---|---|---|---|
| 11/27/1993 | 24.27 | 2.22 | 0 | 0 | 0 | 0 | 0 | 0 |
| 11/28/1993 | 26.05 | 3.33 | 0 | 0 | 0 | 0 | 0 | 0 |
| 11/29/1993 | 29.73 | 3.89 | 0 | 0 | 0 | 0 | 0 | 0 |
| 11/30/1993 | 46.16 | 5.00 | 0 | 0 | 0 | 0 | 0 | 0 |
| 12/1/1993 | 42.48 | 6.11 | 0 | 114 | 10 | 0 | 0 | 0 |
| 12/2/1993 | 97.98 | 6.11 | 0 | 138 | 20 | 0 | 0 | 0 |
| 12/3/1993 | 53.24 | 5.56 | 7 | 72 | 29 | 0 | 0 | 0 |
| 12/4/1993 | 45.87 | 6.11 | 2 | 36 | 6 | 0 | 0 | 0 |
| 12/5/1993 | 61.45 | 5.56 | 1 | 29 | 12 | 0 | 0 | 0 |
| 12/6/1993 | 48.42 | 5.00 | 1 | 13 | 3 | 0 | 0 | 0 |
| 12/7/1993 | 44.74 | 5.00 | 0 | 19 | 3 | 0 | 0 | 0 |
| 12/8/1993 | 201.33 | 6.11 | 0 | 9 | 12 | 0 | 0 | 0 |
| 12/9/1993 | 208.98 | 6.67 | 0 | 11 | 26 | 0 | 0 | 0 |
| 12/10/1993 | 119.21 | 6.67 | 0 | 6 | 20 | 0 | 0 | 0 |
| 12/11/1993 | 123.46 | 6.67 | 0 | 2 | 46 | 0 | 0 | 0 |
| 12/12/1993 | 160.27 | 6.11 | 0 | 0 | 12 | 0 | 0 | 0 |
| 12/13/1993 | 105.34 | 5.56 | 0 | 2 | 5 | 0 | 0 | 0 |
| 12/14/1993 | 82.97 | 5.56 | 0 | 0 | 7 | 0 | 0 | 0 |
| 12/15/1993 | 71.92 | 6.11 | 0 | 0 | 22 | 0 | 0 | 0 |
| 12/16/1993 | 66.83 | 5.00 | 0 | 0 | 1 | 0 | 0 | 0 |
| 12/17/1993 | 59.75 | 4.44 | 0 | 0 | 2 | 0 | 0 | 0 |
| 12/18/1993 | 48.99 | 3.33 | 0 | 0 | 0 | 0 | 0 | 0 |
| 12/19/1993 | 45.59 | 2.78 | 0 | 0 | 0 | 0 | 0 | 0 |
| 12/20/1993 | 43.89 | 2.22 | 0 | 0 | 0 | 0 | 0 | 0 |
| 12/21/1993 | 42.19 | 2.22 | 0 | 0 | 0 | 0 | 0 | 0 |
| 12/22/1993 | 37.10 | 2.22 | 0 | 0 | 0 | 0 | 0 | 0 |
| 12/23/1993 | 35.96 | 2.22 | 0 | 0 | 0 | 0 | 0 | 0 |
| 12/24/1993 | 32.85 | 1.67 | 0 | 0 | 0 | 0 | 0 | 0 |
| 12/25/1993 | 31.71 | 1.11 | 0 | 0 | 0 | 0 | 0 | 0 |
| 12/26/1993 | 32.28 | 1.67 | 0 | 0 | 0 | 0 | 0 | 0 |
| 12/27/1993 | 32.56 | 1.67 | 0 | 0 | 0 | 0 | 0 | 0 |
| 12/28/1993 | 33.13 | 2.78 | 0 | 0 | 0 | 0 | 0 | 0 |
| 12/29/1993 | 31.71 | 2.78 | 0 | 0 | 0 | 0 | 0 | 0 |
| 12/30/1993 | 30.30 | 3.33 | 0 | 0 | 5 | 0 | 0 | 0 |
| 12/31/1993 | 31.71 | 3.89 | 0 | 0 | 2 | 0 | 0 | 0 |
| 1/1/1994 | 34.55 | 5.00 | 0 | 0 | 1 | 0 | 0 | 0 |
| 1/2/1994 | 65.70 | 5.56 | 0 | 0 | 14 | 0 | 0 | 0 |
| 1/3/1994 | 195.39 | 7.22 | 0 | 0 | 0 | 0 | 0 | 0 |
| 1/4/1994 | 139.60 | 7.78 | 0 | 0 | 0 | 0 | 0 | 0 |
| 1/5/1994 | 215.49 | 7.22 | 0 | 0 | 0 | 0 | 0 | 0 |
| 1/6/1994 | 223.70 | 6.11 | 0 | 0 | 0 | 0 | 0 | 0 |
| 1/7/1994 | 150.08 | 6.11 | 0 | 0 | 10 | 0 | 0 | 0 |
| 1/8/1994 | 119.21 | 6.11 | 0 | 0 | 0 | 0 | 0 | 0 |
| 1/9/1994 | 209.26 | 6.67 | 0 | 0 | 0 | 0 | 0 | 0 |
| 1/10/1994 | 228.52 | 6.67 | 0 | 0 | 48 | 0 | 0 | 0 |

| Date | Discharge (cms) | Temp (C°) | Chinook | Coho | Steelhead | Cutthroat | Lamprey | Sucker |
|---|---|---|---|---|---|---|---|---|
| 1/11/1994 | 172.73 | 6.67 | 0 | 0 | 23 | 0 | 0 | 0 |
| 1/12/1994 | 158.29 | 6.11 | 0 | 0 | 32 | 0 | 0 | 0 |
| 1/13/1994 | 126.29 | 6.11 | 0 | 0 | 18 | 0 | 0 | 0 |
| 1/14/1994 | 108.74 | 5.56 | 0 | 0 | 0 | 0 | 0 | 0 |
| 1/15/1994 | 96.56 | 5.56 | 0 | 0 | 0 | 0 | 0 | 0 |
| 1/16/1994 | 83.82 | 5.56 | 0 | 0 | 0 | 0 | 0 | 0 |
| 1/17/1994 | 74.19 | 4.44 | 0 | 0 | 0 | 0 | 0 | 0 |
| 1/18/1994 | 64.85 | 4.44 | 0 | 0 | 21 | 0 | 0 | 0 |
| 1/19/1994 | 58.90 | 3.33 | 0 | 0 | 0 | 0 | 0 | 1 |
| 1/20/1994 | 58.05 | 3.33 | 0 | 0 | 0 | 0 | 0 | 0 |
| 1/21/1994 | 55.22 | 4.44 | 0 | 0 | 1 | 0 | 0 | 0 |
| 1/22/1994 | 52.67 | 6.11 | 0 | 0 | 13 | 0 | 0 | 0 |
| 1/23/1994 | 60.60 | 6.67 | 0 | 0 | 17 | 0 | 0 | 0 |
| 1/24/1994 | 71.08 | 7.22 | 0 | 0 | 36 | 0 | 0 | 0 |
| 1/25/1994 | 78.44 | 6.67 | 0 | 0 | 0 | 0 | 0 | 1 |
| 1/26/1994 | 70.51 | 5.00 | 0 | 0 | 6 | 0 | 0 | 0 |
| 1/27/1994 | 61.45 | 5.00 | 0 | 0 | 0 | 0 | 0 | 0 |
| 1/28/1994 | 55.22 | 5.00 | 0 | 0 | 7 | 0 | 0 | 0 |
| 1/29/1994 | 52.10 | 4.44 | 0 | 0 | 0 | 0 | 0 | 0 |
| 1/30/1994 | 49.84 | 3.33 | 0 | 0 | 0 | 0 | 0 | 0 |
| 1/31/1994 | 47.29 | 2.78 | 0 | 0 | 0 | 0 | 0 | 0 |
| 2/1/1994 | 44.74 | 2.78 | 0 | 0 | 0 | 0 | 0 | 0 |
| 2/2/1994 | 45.02 | 2.22 | 0 | 0 | 0 | 0 | 0 | 0 |
| 2/3/1994 | 43.89 | 2.22 | 0 | 0 | 0 | 0 | 0 | 0 |
| 2/4/1994 | 42.76 | 2.22 | 0 | 0 | 0 | 0 | 0 | 0 |
| 2/5/1994 | 39.93 | 2.22 | 0 | 0 | 0 | 0 | 0 | 0 |
| 2/6/1994 | 39.08 | 2.22 | 0 | 0 | 0 | 0 | 0 | 0 |
| 2/7/1994 | 41.06 | 2.78 | 0 | 0 | 0 | 0 | 0 | 0 |
| 2/8/1994 | 38.79 | 3.33 | 0 | 0 | 0 | 0 | 0 | 0 |
| 2/9/1994 | 37.66 | 3.33 | 0 | 0 | 0 | 0 | 0 | 0 |
| 2/10/1994 | 48.14 | 4.44 | 0 | 0 | 6 | 0 | 0 | 0 |
| 2/11/1994 | 50.40 | 4.44 | 0 | 0 | 0 | 0 | 0 | 0 |
| 2/12/1994 | 47.86 | 3.89 | 0 | 0 | 2 | 0 | 0 | 0 |
| 2/13/1994 | 46.44 | 3.33 | 0 | 0 | 0 | 0 | 0 | 0 |
| 2/14/1994 | 56.63 | 4.44 | 0 | 0 | 18 | 0 | 0 | 0 |
| 2/15/1994 | 62.58 | 5.00 | 0 | 0 | 26 | 0 | 0 | 0 |
| 2/16/1994 | 62.01 | 5.56 | 0 | 0 | 39 | 0 | 0 | 0 |
| 2/17/1994 | 72.21 | 5.56 | 0 | 0 | 55 | 0 | 0 | 0 |
| 2/18/1994 | 105.06 | 5.56 | 0 | 0 | 55 | 0 | 0 | 0 |
| 2/19/1994 | 97.41 | 5.00 | 0 | 0 | 1 | 0 | 0 | 0 |
| 2/20/1994 | 85.52 | 5.56 | 0 | 0 | 20 | 0 | 0 | 0 |
| 2/21/1994 | 90.90 | 5.56 | 0 | 0 | 71 | 0 | 0 | 0 |
| 2/22/1994 | 90.33 | 5.56 | 0 | 0 | 55 | 0 | 0 | 0 |
| 2/23/1994 | 86.37 | 5.00 | 0 | 0 | 49 | 0 | 0 | 0 |
| 2/24/1994 | 222.85 | 6.11 | 0 | 0 | 195 | 0 | 0 | 0 |

| Date | Discharge (cms) | Temp (C°) | Chinook | Coho | Steelhead | Cutthroat | Lamprey | Sucker |
|---|---|---|---|---|---|---|---|---|
| 2/25/1994 | 260.23 | 6.67 | 0 | 0 | 40 | 0 | 0 | 0 |
| 2/26/1994 | 221.44 | 7.22 | 0 | 0 | 75 | 0 | 0 | 0 |
| 2/27/1994 | 268.44 | 7.22 | 0 | 0 | 166 | 0 | 0 | 0 |
| 2/28/1994 | 234.18 | 7.78 | 0 | 0 | 63 | 0 | 0 | 0 |
| 3/1/1994 | 195.67 | 7.78 | 0 | 0 | 83 | 0 | 0 | 0 |
| 3/2/1994 | 177.55 | 7.78 | 0 | 0 | 108 | 0 | 0 | 0 |
| 3/3/1994 | 168.49 | 8.33 | 0 | 0 | 124 | 0 | 0 | 0 |
| 3/4/1994 | 172.45 | 8.33 | 0 | 0 | 90 | 0 | 0 | 0 |
| 3/5/1994 | 147.81 | 8.33 | 0 | 0 | 42 | 0 | 0 | 0 |
| 3/6/1994 | 121.48 | 7.22 | 0 | 0 | 9 | 0 | 0 | 0 |
| 3/7/1994 | 102.51 | 6.67 | 0 | 0 | 4 | 0 | 0 | 0 |
| 3/8/1994 | 88.07 | 6.67 | 0 | 0 | 9 | 0 | 0 | 0 |
| 3/9/1994 | 83.53 | 7.78 | 0 | 0 | 43 | 0 | 0 | 0 |
| 3/10/1994 | 76.74 | 8.33 | 0 | 0 | 51 | 0 | 0 | 0 |
| 3/11/1994 | 70.23 | 8.33 | 0 | 0 | 12 | 0 | 0 | 0 |
| 3/12/1994 | 65.41 | 7.78 | 0 | 0 | 15 | 0 | 0 | 0 |
| 3/13/1994 | 62.86 | 7.22 | 0 | 0 | 6 | 0 | 0 | 0 |
| 3/14/1994 | 60.31 | 8.33 | 0 | 0 | 17 | 0 | 0 | 0 |
| 3/15/1994 | 57.77 | 8.33 | 0 | 0 | 83 | 0 | 0 | 0 |
| 3/16/1994 | 60.03 | 8.33 | 0 | 0 | 158 | 0 | 0 | 0 |
| 3/17/1994 | 61.16 | 8.89 | 0 | 0 | 5 | 0 | 0 | 0 |
| 3/18/1994 | 55.78 | 7.78 | 0 | 0 | 0 | 0 | 0 | 0 |
| 3/19/1994 | 65.41 | 7.78 | 0 | 0 | 34 | 0 | 0 | 0 |
| 3/20/1994 | 60.60 | 6.67 | 0 | 0 | 3 | 0 | 0 | 0 |
| 3/21/1994 | 61.16 | 6.11 | 0 | 0 | 0 | 0 | 0 | 0 |
| 3/22/1994 | 71.64 | 5.56 | 0 | 0 | 0 | 0 | 0 | 0 |
| 3/23/1994 | 68.81 | 5.00 | 0 | 0 | 0 | 0 | 0 | 0 |
| 3/24/1994 | 65.70 | 7.22 | 0 | 0 | 0 | 0 | 0 | 0 |
| 3/25/1994 | 67.11 | 7.78 | 0 | 0 | 13 | 0 | 0 | 0 |
| 3/26/1994 | 67.39 | 8.33 | 0 | 0 | 19 | 0 | 0 | 0 |
| 3/27/1994 | 60.31 | 8.89 | 2 | 0 | 122 | 0 | 0 | 0 |
| 3/28/1994 | 58.33 | 10.00 | 3 | 0 | 249 | 0 | 0 | 1 |
| 3/29/1994 | 56.35 | 10.00 | 2 | 0 | 185 | 0 | 0 | 0 |
| 3/30/1994 | 80.99 | 9.44 | 2 | 0 | 140 | 0 | 0 | 0 |
| 3/31/1994 | 158.57 | 8.89 | 3 | 0 | 45 | 0 | 0 | 0 |
| 4/1/1994 | 148.38 | 9.44 | 0 | 0 | 21 | 0 | 0 | 0 |
| 4/2/1994 | 116.38 | 9.44 | 0 | 0 | 35 | 0 | 0 | 0 |
| 4/3/1994 | 101.37 | 9.44 | 0 | 0 | 53 | 0 | 0 | 0 |
| 4/4/1994 | 85.23 | 10.00 | 2 | 0 | 35 | 0 | 0 | 0 |
| 4/5/1994 | 75.32 | 8.33 | 1 | 0 | 8 | 0 | 0 | 0 |
| 4/6/1994 | 80.42 | 7.78 | 0 | 0 | 2 | 0 | 0 | 0 |
| 4/7/1994 | 111.29 | 8.33 | 2 | 0 | 19 | 0 | 0 | 0 |
| 4/8/1994 | 118.36 | 7.78 | 0 | 0 | 23 | 0 | 0 | 0 |
| 4/9/1994 | 166.22 | 8.33 | 7 | 0 | 47 | 0 | 0 | 0 |
| 4/10/1994 | 229.08 | 8.89 | 3 | 0 | 27 | 0 | 0 | 0 |

| Date | Discharge (cms) | Temp (C°) | Chinook | Coho | Steelhead | Cutthroat | Lamprey | Sucker |
|---|---|---|---|---|---|---|---|---|
| 4/11/1994 | 196.24 | 8.89 | 0 | 0 | 35 | 0 | 0 | 0 |
| 4/12/1994 | 149.51 | 9.44 | 2 | 0 | 26 | 0 | 0 | 0 |
| 4/13/1994 | 123.74 | 9.44 | 2 | 0 | 38 | 0 | 0 | 0 |
| 4/14/1994 | 106.19 | 10.56 | 1 | 0 | 17 | 0 | 0 | 0 |
| 4/15/1994 | 92.31 | 11.11 | 6 | 0 | 46 | 0 | 0 | 0 |
| 4/16/1994 | 84.38 | 11.11 | 25 | 0 | 40 | 0 | 0 | 0 |
| 4/17/1994 | 80.70 | 11.11 | 5 | 0 | 16 | 0 | 0 | 0 |
| 4/18/1994 | 77.59 | 12.22 | 19 | 0 | 34 | 1 | 0 | 0 |
| 4/19/1994 | 76.74 | 13.33 | 7 | 0 | 23 | 0 | 0 | 0 |
| 4/20/1994 | 75.61 | 12.78 | 0 | 0 | 7 | 0 | 0 | 0 |
| 4/21/1994 | 72.77 | 13.89 | 53 | 0 | 22 | 0 | 0 | 3 |
| 4/22/1994 | 68.81 | 12.78 | 35 | 0 | 5 | 0 | 0 | 0 |
| 4/23/1994 | 64.28 | 11.67 | 3 | 0 | 7 | 0 | 0 | 0 |
| 4/24/1994 | 62.30 | 11.11 | 7 | 0 | 3 | 0 | 0 | 0 |
| 4/25/1994 | 62.58 | 10.56 | 19 | 0 | 10 | 0 | 0 | 0 |
| 4/26/1994 | 60.88 | 10.56 | 11 | 0 | 7 | 0 | 0 | 0 |
| 4/27/1994 | 58.90 | 10.56 | 17 | 0 | 6 | 0 | 0 | 0 |
| 4/28/1994 | 59.18 | 10.56 | 35 | 0 | 4 | 0 | 0 | 0 |
| 4/29/1994 | 54.65 | 11.11 | 61 | 0 | 15 | 0 | 0 | 1 |
| 4/30/1994 | 58.05 | 12.22 | 18 | 0 | 6 | 0 | 0 | 0 |
| 5/1/1994 | 58.90 | 11.67 | 0 | 0 | 0 | 0 | 0 | 0 |
| 5/2/1994 | 55.50 | 11.67 | 37 | 0 | 8 | 0 | 0 | 5 |
| 5/3/1994 | 51.25 | 11.67 | 104 | 0 | 13 | 0 | 0 | 474 |
| 5/4/1994 | 52.95 | 12.22 | 47 | 0 | 4 | 0 | 0 | 210 |
| 5/5/1994 | 59.47 | 13.33 | 68 | 0 | 3 | 0 | 0 | 1150 |
| 5/6/1994 | 57.48 | 15.00 | 34 | 0 | 3 | 0 | 0 | 572 |
| 5/7/1994 | 55.22 | 16.11 | 39 | 0 | 0 | 0 | 0 | 219 |
| 5/8/1994 | 53.24 | 16.67 | 22 | 0 | 1 | 0 | 0 | 87 |
| 5/9/1994 | 52.10 | 16.67 | 28 | 0 | 0 | 0 | 1 | 914 |
| 5/10/1994 | 51.82 | 16.67 | 0 | 0 | 0 | 0 | 0 | 365 |
| 5/11/1994 | 50.12 | 16.67 | 99 | 0 | 3 | 0 | 3 | 804 |
| 5/12/1994 | 48.70 | 16.11 | 60 | 0 | 9 | 0 | 1 | 9 |
| 5/13/1994 | 47.01 | 15.56 | 67 | 0 | 2 | 0 | 0 | 193 |
| 5/14/1994 | 45.02 | 15.00 | 108 | 0 | 7 | 0 | 0 | 7 |
| 5/15/1994 | 44.17 | 13.33 | 74 | 0 | 4 | 0 | 0 | 0 |
| 5/16/1994 | 46.72 | 13.89 | 26 | 0 | 1 | 0 | 0 | 21 |
| 5/17/1994 | 48.42 | 13.33 | 43 | 0 | 5 | 0 | 0 | 11 |
| 5/18/1994 | 48.14 | 13.33 | 59 | 0 | 7 | 0 | 0 | 4 |
| 5/19/1994 | 46.72 | 12.78 | 44 | 0 | 6 | 0 | 0 | 37 |
| 5/20/1994 | 47.29 | 12.22 | 26 | 0 | 4 | 0 | 0 | 0 |
| 5/21/1994 | 49.55 | 13.33 | 19 | 0 | 5 | 0 | 0 | 0 |
| 5/22/1994 | 48.99 | 15.00 | 47 | 0 | 5 | 0 | 0 | 74 |
| 5/23/1994 | 47.29 | 16.11 | 83 | 0 | 10 | 0 | 5 | 2543 |
| 5/24/1994 | 45.02 | 16.67 | 81 | 0 | 3 | 0 | 15 | 799 |
| 5/25/1994 | 43.61 | 17.78 | 59 | 0 | 3 | 0 | 10 | 181 |

| Date | Discharge (cms) | Temp (C°) | Chinook | Coho | Steelhead | Cutthroat | Lamprey | Sucker |
|---|---|---|---|---|---|---|---|---|
| 5/26/1994 | 41.34 | 16.67 | 75 | 0 | 8 | 0 | 2 | 657 |
| 5/27/1994 | 40.49 | 16.67 | 36 | 0 | 6 | 0 | 3 | 248 |
| 5/28/1994 | 40.49 | 15.56 | 95 | 0 | 11 | 0 | 1 | 16 |
| 5/29/1994 | 39.64 | 15.00 | 76 | 0 | 9 | 0 | 0 | 5 |
| 5/30/1994 | 39.36 | 16.11 | 37 | 0 | 3 | 0 | 1 | 0 |
| 5/31/1994 | 39.08 | 15.56 | 64 | 0 | 15 | 0 | 2 | 996 |
| 6/1/1994 | 38.79 | 16.67 | 22 | 0 | 5 | 0 | 6 | 231 |
| 6/2/1994 | 39.08 | 16.67 | 18 | 0 | 8 | 0 | 9 | 597 |
| 6/3/1994 | 37.10 | 15.56 | 23 | 0 | 1 | 0 | 1 | 22 |
| 6/4/1994 | 35.68 | 15.56 | 2 | 0 | 1 | 0 | 0 | 0 |
| 6/5/1994 | 33.70 | 15.56 | 2 | 0 | 0 | 0 | 0 | 0 |
| 6/6/1994 | 38.79 | 15.00 | 19 | 0 | 19 | 0 | 0 | 0 |
| 6/7/1994 | 64.85 | 14.44 | 40 | 0 | 0 | 0 | 0 | 165 |
| 6/8/1994 | 62.01 | 15.00 | 11 | 0 | 19 | 0 | 0 | 191 |
| 6/9/1994 | 50.69 | 16.11 | 25 | 0 | 26 | 0 | 9 | 1314 |
| 6/10/1994 | 46.72 | 17.78 | 30 | 0 | 11 | 0 | 21 | 732 |
| 6/11/1994 | 43.04 | 18.33 | 17 | 0 | 5 | 0 | 12 | 39 |
| 6/12/1994 | 40.78 | 17.78 | 30 | 0 | 9 | 0 | 20 | 1 |
| 6/13/1994 | 39.08 | 17.22 | 39 | 0 | 20 | 0 | 3 | 0 |
| 6/14/1994 | 39.64 | 16.11 | 4 | 0 | 1 | 0 | 0 | 0 |
| 6/15/1994 | 41.34 | 16.11 | 14 | 0 | 14 | 0 | 0 | 2 |
| 6/16/1994 | 41.34 | 16.67 | 32 | 0 | 14 | 0 | 0 | 0 |
| 6/17/1994 | 38.51 | 15.56 | 104 | 0 | 31 | 0 | 1 | 0 |
| 6/18/1994 | 37.38 | 16.67 | 9 | 0 | 4 | 0 | 0 | 0 |
| 6/19/1994 | 37.10 | 17.22 | 52 | 0 | 22 | 0 | 3 | 3 |
| 6/20/1994 | 35.68 | 18.33 | 25 | 0 | 7 | 0 | 2 | 0 |
| 6/21/1994 | 33.41 | 17.22 | 0 | 0 | 0 | 0 | 0 | 0 |
| 6/22/1994 | 32.28 | 16.67 | 18 | 0 | 35 | 0 | 1 | 72 |
| 6/23/1994 | 32.85 | 17.78 | 0 | 0 | 0 | 0 | 0 | 0 |
| 6/24/1994 | 31.71 | 18.89 | 64 | 0 | 71 | 0 | 3 | 5 |
| 6/25/1994 | 30.02 | 17.78 | 59 | 0 | 53 | 0 | 1 | 1 |
| 6/26/1994 | 30.58 | 18.89 | 45 | 0 | 41 | 0 | 6 | 0 |
| 6/27/1994 | 30.30 | 20.00 | 46 | 0 | 33 | 0 | 17 | 17 |
| 6/28/1994 | 29.73 | 21.11 | 46 | 0 | 61 | 0 | 27 | 114 |
| 6/29/1994 | 29.17 | 19.44 | 36 | 0 | 64 | 0 | 20 | 18 |
| 6/30/1994 | 27.86 | 20.00 | 39 | 0 | 83 | 0 | 9 | 3 |
| 7/1/1994 | 27.38 | 20.56 | 34 | 0 | 45 | 0 | 12 | 13 |
| 7/2/1994 | 27.35 | 20.00 | 31 | 0 | 49 | 0 | 15 | 27 |
| 7/3/1994 | 27.89 | 20.56 | 29 | 0 | 33 | 0 | 5 | 0 |
| 7/4/1994 | 26.42 | 20.56 | 23 | 0 | 51 | 0 | 8 | 3 |
| 7/5/1994 | 26.31 | 19.44 | 12 | 0 | 40 | 0 | 5 | 4 |
| 7/6/1994 | 27.27 | 20.00 | 12 | 0 | 44 | 0 | 1 | 0 |
| 7/7/1994 | 25.85 | 21.11 | 20 | 0 | 41 | 0 | 11 | 7 |
| 7/8/1994 | 25.34 | 22.22 | 9 | 0 | 39 | 0 | 13 | 2 |
| 7/9/1994 | 23.84 | 22.22 | 21 | 0 | 42 | 0 | 9 | 0 |

| Date | Discharge (cms) | Temp (C°) | Chinook | Coho | Steelhead | Cutthroat | Lamprey | Sucker |
|---|---|---|---|---|---|---|---|---|
| 7/10/1994 | 22.63 | 22.22 | 20 | 0 | 44 | 0 | 17 | 1 |
| 7/11/1994 | 24.41 | 22.22 | 9 | 0 | 33 | 0 | 19 | 2 |
| 7/12/1994 | 24.24 | 21.67 | 9 | 0 | 27 | 0 | 22 | 9 |
| 7/13/1994 | 24.55 | 21.67 | 4 | 0 | 16 | 0 | 8 | 1 |
| 7/14/1994 | 24.64 | 21.67 | 4 | 0 | 11 | 0 | 5 | 4 |
| 7/15/1994 | 24.66 | 21.11 | 1 | 0 | 9 | 0 | 0 | 0 |
| 7/16/1994 | 24.01 | 22.22 | 1 | 0 | 6 | 0 | 5 | 2 |
| 7/17/1994 | 23.45 | 22.78 | 2 | 0 | 5 | 0 | 13 | 1 |
| 7/18/1994 | 23.05 | 22.78 | 5 | 0 | 0 | 0 | 3 | 3 |
| 7/19/1994 | 23.47 | 23.33 | 0 | 0 | 0 | 0 | 8 | 3 |
| 7/20/1994 | 23.90 | 24.44 | 0 | 0 | 1 | 0 | 17 | 8 |
| 7/21/1994 | 23.81 | 23.89 | 0 | 0 | 0 | 0 | 5 | 5 |
| 7/22/1994 | 22.54 | 22.78 | 2 | 0 | 0 | 0 | 3 | 1 |
| 7/23/1994 | 22.31 | 23.33 | 0 | 0 | 0 | 0 | 0 | 0 |
| 7/24/1994 | 23.70 | 22.22 | 0 | 0 | 0 | 0 | 0 | 0 |
| 7/25/1994 | 25.80 | 21.67 | 0 | 0 | 0 | 0 | 1 | 0 |
| 7/26/1994 | 24.69 | 22.22 | 0 | 0 | 0 | 0 | 0 | 1 |
| 7/27/1994 | 23.11 | 22.78 | 0 | 0 | 0 | 0 | 2 | 0 |
| 7/28/1994 | 22.17 | 22.22 | 4 | 0 | 0 | 0 | 0 | 0 |
| 7/29/1994 | 23.22 | 21.11 | 1 | 0 | 0 | 0 | 1 | 1 |
| 7/30/1994 | 21.75 | 20.56 | 1 | 0 | 0 | 0 | 0 | 0 |
| 7/31/1994 | 20.59 | 21.67 | 5 | 0 | 1 | 0 | 0 | 2 |
| 8/1/1994 | 20.64 | 21.67 | 14 | 0 | 1 | 0 | 0 | 0 |
| 8/2/1994 | 21.69 | 21.67 | 21 | 0 | 1 | 0 | 0 | 0 |
| 8/3/1994 | 20.73 | 20.56 | 21 | 0 | 2 | 0 | 0 | 0 |
| 8/4/1994 | 20.36 | 21.11 | 37 | 0 | 2 | 0 | 0 | 0 |
| 8/5/1994 | 20.25 | 20.56 | 22 | 0 | 0 | 0 | 0 | 0 |
| 8/6/1994 | 20.19 | 20.00 | 22 | 0 | 3 | 0 | 0 | 0 |
| 8/7/1994 | 20.16 | 19.44 | 24 | 0 | 4 | 0 | 0 | 0 |
| 8/8/1994 | 20.02 | 19.44 | 12 | 0 | 13 | 0 | 0 | 0 |
| 8/9/1994 | 19.65 | 19.44 | 6 | 0 | 12 | 0 | 0 | 1 |
| 8/10/1994 | 18.97 | 20.56 | 11 | 0 | 11 | 0 | 1 | 0 |
| 8/11/1994 | 19.00 | 20.00 | 15 | 0 | 20 | 0 | 1 | 1 |
| 8/12/1994 | 19.00 | 20.56 | 20 | 0 | 27 | 0 | 0 | 7 |
| 8/13/1994 | 18.97 | 21.11 | 22 | 0 | 68 | 0 | 0 | 3 |
| 8/14/1994 | 18.94 | 21.11 | 21 | 0 | 58 | 0 | 3 | 2 |
| 8/15/1994 | 18.92 | 21.67 | 17 | 0 | 75 | 0 | 0 | 1 |
| 8/16/1994 | 18.77 | 20.56 | 21 | 0 | 63 | 0 | 0 | 0 |
| 8/17/1994 | 18.58 | 20.00 | 13 | 0 | 39 | 0 | 0 | 1 |
| 8/18/1994 | 20.53 | 20.00 | 12 | 0 | 44 | 0 | 0 | 2 |
| 8/19/1994 | 24.69 | | 0 | 0 | 0 | 0 | 0 | 0 |
| 8/20/1994 | 21.07 | | 0 | 0 | 0 | 0 | 0 | 0 |
| 8/21/1994 | 19.17 | | 0 | 0 | 0 | 0 | 0 | 0 |
| 8/22/1994 | 15.55 | 20.00 | 20 | 0 | 42 | 0 | 0 | 0 |
| 8/23/1994 | 20.67 | 19.44 | 16 | 0 | 19 | 0 | 0 | 0 |

| Date | Discharge (cms) | Temp (C°) | Chinook | Coho | Steelhead | Cutthroat | Lamprey | Sucker |
|---|---|---|---|---|---|---|---|---|
| 8/24/1994 | 20.59 | 19.44 | 11 | 0 | 9 | 0 | 0 | 0 |
| 8/25/1994 | 20.67 | 18.89 | 10 | 0 | 15 | 0 | 0 | 0 |
| 8/26/1994 | 20.61 | 18.89 | 13 | 0 | 25 | 0 | 0 | 3 |
| 8/27/1994 | 20.56 | 18.89 | 14 | 0 | 4 | 0 | 0 | 0 |
| 8/28/1994 | 20.44 | 17.78 | 10 | 0 | 3 | 0 | 0 | 0 |
| 8/29/1994 | 20.44 | 17.78 | 1 | 0 | 0 | 0 | 0 | 0 |
| 8/30/1994 | 20.42 | 18.33 | 3 | 0 | 6 | 0 | 0 | 2 |
| 8/31/1994 | 20.02 | 18.89 | 10 | 0 | 11 | 0 | 0 | 3 |
| 9/1/1994 | 19.62 | 17.78 | 17 | 0 | 5 | 0 | 0 | 6 |
| 9/2/1994 | 19.57 | 17.22 | 7 | 0 | 3 | 0 | 0 | 0 |
| 9/3/1994 | 19.43 | 17.22 | 3 | 0 | 12 | 0 | 0 | 0 |
| 9/4/1994 | 19.96 | 16.67 | 8 | 0 | 21 | 0 | 0 | 0 |
| 9/5/1994 | 20.16 | 17.78 | 8 | 0 | 1 | 0 | 0 | 0 |
| 9/6/1994 | 19.94 | 18.33 | 18 | 0 | 7 | 0 | 0 | 0 |
| 9/7/1994 | 19.48 | 18.33 | 11 | 0 | 6 | 0 | 0 | 0 |
| 9/8/1994 | 19.51 | 17.22 | 10 | 0 | 14 | 0 | 0 | 0 |
| 9/9/1994 | 20.25 | 16.11 | 2 | 0 | 7 | 0 | 0 | 0 |
| 9/10/1994 | 20.53 | 15.56 | 4 | 0 | 1 | 0 | 0 | 0 |
| 9/11/1994 | 21.35 | 15.00 | 4 | 0 | 34 | 0 | 0 | 0 |
| 9/12/1994 | 21.61 | 14.44 | 0 | 0 | 16 | 0 | 0 | 0 |
| 9/13/1994 | 21.27 | 15.00 | 4 | 0 | 22 | 0 | 0 | 0 |
| 9/14/1994 | 20.61 | 15.56 | 6 | 0 | 14 | 0 | 0 | 0 |
| 9/15/1994 | 20.22 | 16.11 | 3 | 0 | 18 | 0 | 0 | 0 |
| 9/16/1994 | 20.22 | 16.39 | 8 | 0 | 17 | 0 | 0 | 0 |
| 9/17/1994 | 19.91 | 15.00 | 13 | 0 | 11 | 0 | 0 | 0 |
| 9/18/1994 | 19.77 | 14.72 | 6 | 0 | 5 | 0 | 0 | 0 |
| 9/19/1994 | 19.62 | 16.67 | 3 | 0 | 15 | 0 | 0 | 0 |
| 9/20/1994 | 19.51 | 17.22 | 7 | 0 | 15 | 0 | 0 | 0 |
| 9/21/1994 | 19.60 | 17.50 | 3 | 0 | 9 | 0 | 0 | 0 |
| 9/22/1994 | 19.71 | 17.22 | 5 | 0 | 12 | 0 | 0 | 0 |
| 9/23/1994 | 19.51 | 16.67 | 6 | 0 | 5 | 0 | 0 | 0 |
| 9/24/1994 | 19.34 | 16.94 | 4 | 0 | 8 | 0 | 0 | 0 |
| 9/25/1994 | 19.51 | 16.94 | 6 | 2 | 12 | 0 | 0 | 0 |
| 9/26/1994 | 19.51 | 16.39 | 7 | 5 | 10 | 0 | 0 | 0 |
| 9/27/1994 | 19.43 | 16.94 | 3 | 1 | 2 | 0 | 0 | 0 |
| 9/28/1994 | 19.62 | 16.39 | 3 | 0 | 0 | 0 | 0 | 0 |
| 9/29/1994 | 22.23 | 15.56 | 6 | 2 | 16 | 0 | 0 | 2 |
| 9/30/1994 | 28.18 | | 0 | 0 | 12 | 0 | 0 | 0 |
| 10/1/1994 | 24.66 | 16.11 | 3 | 2 | 0 | 0 | 0 | 0 |
| 10/2/1994 | 22.23 | 15.56 | 0 | 0 | 4 | 0 | 0 | 0 |
| 10/3/1994 | 21.63 | 14.44 | 0 | 0 | 0 | 0 | 0 | 0 |
| 10/4/1994 | 21.29 | 13.33 | 3 | 0 | 0 | 0 | 0 | 0 |
| 10/5/1994 | 20.39 | 13.33 | 0 | 0 | 0 | 0 | 0 | 0 |
| 10/6/1994 | 20.10 | 12.78 | 2 | 19 | 14 | 0 | 0 | 0 |
| 10/7/1994 | 19.96 | 12.78 | 4 | 30 | 8 | 0 | 0 | 0 |

| Date | Discharge (cms) | Temp (C°) | Chinook | Coho | Steelhead | Cutthroat | Lamprey | Sucker |
|---|---|---|---|---|---|---|---|---|
| 10/8/1994 | 20.30 | 12.78 | 9 | 25 | 5 | 0 | 0 | 0 |
| 10/9/1994 | 20.59 | 12.22 | 4 | 3 | 1 | 0 | 0 | 0 |
| 10/10/1994 | 20.70 | 11.11 | 2 | 3 | 0 | 0 | 0 | 0 |
| 10/11/1994 | 20.47 | 11.11 | 0 | 0 | 0 | 0 | 0 | 0 |
| 10/12/1994 | 20.33 | 11.11 | 1 | 0 | 0 | 0 | 0 | 0 |
| 10/13/1994 | 20.22 | 10.56 | 6 | 7 | 3 | 0 | 0 | 0 |
| 10/14/1994 | 21.52 | 11.11 | 4 | 2 | 0 | 0 | 0 | 0 |
| 10/15/1994 | 26.67 | 10.56 | 5 | 0 | 0 | 0 | 0 | 0 |
| 10/16/1994 | 29.45 | 9.44 | 0 | 3 | 4 | 0 | 0 | 0 |
| 10/17/1994 | 25.71 | 10.00 | 3 | 11 | 3 | 0 | 0 | 0 |
| 10/18/1994 | 24.32 | 10.00 | 10 | 22 | 16 | 0 | 0 | 0 |
| 10/19/1994 | 23.64 | 10.56 | 7 | 22 | 4 | 0 | 0 | 0 |
| 10/20/1994 | 22.88 | 10.56 | 3 | 24 | 2 | 0 | 0 | 0 |
| 10/21/1994 | 23.02 | 10.56 | 1 | 1 | 0 | 0 | 0 | 0 |
| 10/22/1994 | 24.15 | 9.44 | 6 | 2 | 7 | 0 | 0 | 0 |
| 10/23/1994 | 24.52 | 9.44 | 3 | 5 | 4 | 0 | 0 | 0 |
| 10/24/1994 | 23.90 | 9.44 | 2 | 6 | 2 | 0 | 0 | 0 |
| 10/25/1994 | 23.45 | 9.44 | 0 | 2 | 4 | 0 | 0 | 0 |
| 10/26/1994 | 24.66 | 9.44 | 1 | 4 | 2 | 0 | 0 | 0 |
| 10/27/1994 | 26.87 | 10.00 | 5 | 1 | 0 | 0 | 0 | 0 |
| 10/28/1994 | 63.43 | 10.00 | 3 | 68 | 26 | 0 | 0 | 0 |
| 10/29/1994 | 52.67 | 9.44 | 8 | 117 | 11 | 0 | 0 | 0 |
| 10/30/1994 | 32.56 | 8.89 | 3 | 42 | 8 | 0 | 0 | 0 |
| 10/31/1994 | 27.27 | 8.89 | 0 | 0 | 0 | 0 | 0 | 0 |
| 11/1/1994 | 32.85 | 8.33 | 1 | 79 | 11 | 0 | 0 | 0 |
| 11/2/1994 | 56.92 | 7.50 | 3 | 57 | 14 | 0 | 0 | 0 |
| 11/3/1994 | 43.89 | 6.39 | 1 | 12 | 2 | 0 | 0 | 0 |
| 11/4/1994 | 39.93 | 6.11 | 1 | 6 | 0 | 0 | 0 | 0 |
| 11/5/1994 | 143.00 | 6.11 | 0 | 71 | 0 | 0 | 0 | 0 |
| 11/6/1994 | 73.34 | 6.11 | 3 | 140 | 19 | 0 | 0 | 0 |
| 11/7/1994 | 68.53 | 5.56 | 3 | 40 | 8 | 0 | 0 | 0 |
| 11/8/1994 | 55.78 | 5.00 | 0 | 8 | 3 | 0 | 0 | 0 |
| 11/9/1994 | 54.65 | 5.28 | 2 | 25 | 1 | 0 | 0 | 0 |
| 11/10/1994 | 95.43 | 5.00 | 0 | 25 | 10 | 0 | 0 | 0 |
| 11/11/1994 | 59.18 | 4.72 | 0 | 26 | 5 | 0 | 0 | 0 |
| 11/12/1994 | 47.86 | 5.28 | 1 | 24 | 3 | 0 | 0 | 0 |
| 11/13/1994 | 45.59 | 5.28 | 1 | 11 | 2 | 0 | 0 | 0 |
| 11/14/1994 | 41.06 | 5.00 | 0 | 1 | 0 | 0 | 0 | 0 |
| 11/15/1994 | 38.51 | 5.28 | 0 | 0 | 0 | 0 | 0 | 0 |
| 11/16/1994 | 48.14 | 5.00 | 0 | 5 | 3 | 0 | 0 | 0 |
| 11/17/1994 | 85.23 | 4.44 | 0 | 38 | 10 | 0 | 0 | 0 |
| 11/18/1994 | 69.94 | 3.33 | 0 | 6 | 2 | 0 | 0 | 1 |
| 11/19/1994 | 50.40 | 2.78 | 0 | 1 | 1 | 0 | 0 | 0 |
| 11/20/1994 | 94.86 | 3.33 | 0 | 2 | 6 | 0 | 0 | 0 |
| 11/21/1994 | 116.95 | 3.61 | 0 | 32 | 4 | 0 | 0 | 0 |

| Date | Discharge (cms) | Temp (C°) | Chinook | Coho | Steelhead | Cutthroat | Lamprey | Sucker |
|---|---|---|---|---|---|---|---|---|
| 11/22/1994 | 76.46 | 3.06 | 0 | 0 | 3 | 0 | 0 | 0 |
| 11/23/1994 | 59.47 | 2.50 | 0 | 0 | 1 | 0 | 0 | 0 |
| 11/24/1994 | 79.00 | 3.33 | 0 | 7 | 12 | 0 | 0 | 0 |
| 11/25/1994 | 187.46 | 4.72 | 0 | 24 | 26 | 0 | 0 | 0 |
| 11/26/1994 | 152.06 | 5.00 | 0 | 41 | 9 | 0 | 0 | 0 |
| 11/27/1994 | 108.17 | 5.00 | 0 | 19 | 21 | 0 | 0 | 0 |
| 11/28/1994 | 116.95 | 5.28 | 0 | 3 | 21 | 0 | 0 | 0 |
| 11/29/1994 | 97.69 | 5.56 | 0 | 5 | 10 | 0 | 0 | 0 |
| 11/30/1994 | 211.81 | 6.11 | 0 | 3 | 12 | 0 | 0 | 0 |
| 12/1/1994 | 487.05 | 6.67 | 0 | 0 | 0 | 0 | 0 | 0 |
| 12/2/1994 | 342.63 | 6.11 | 0 | 6 | 31 | 0 | 0 | 0 |
| 12/3/1994 | 231.35 | 5.56 | 0 | 0 | 2 | 0 | 0 | 0 |
| 12/4/1994 | 156.88 | 3.89 | 0 | 0 | 0 | 0 | 0 | 0 |
| 12/5/1994 | 116.95 | 2.78 | 0 | 0 | 1 | 0 | 0 | 0 |
| 12/6/1994 | 103.07 | 2.78 | 0 | 0 | 0 | 0 | 0 | 0 |
| 12/7/1994 | 90.61 | 3.06 | 0 | 1 | 0 | 0 | 0 | 0 |
| 12/8/1994 | 78.15 | 2.78 | 0 | 0 | 0 | 0 | 0 | 0 |
| 12/9/1994 | 67.11 | 2.78 | 0 | 0 | 0 | 0 | 0 | 0 |
| 12/10/1994 | 63.43 | 2.50 | 0 | 0 | 0 | 0 | 0 | 0 |
| 12/11/1994 | 63.43 | 2.78 | 0 | 0 | 0 | 0 | 0 | 0 |
| 12/12/1994 | 71.92 | 3.33 | 0 | 0 | 23 | 0 | 0 | 1 |
| 12/13/1994 | 73.62 | 3.61 | 0 | 0 | 5 | 0 | 0 | 0 |
| 12/14/1994 | 70.23 | 3.33 | 0 | 1 | 0 | 0 | 0 | 0 |
| 12/15/1994 | 74.47 | 3.33 | 0 | 0 | 0 | 0 | 0 | 0 |
| 12/16/1994 | 90.61 | 4.17 | 0 | 1 | 0 | 0 | 0 | 0 |
| 12/17/1994 | 161.12 | 5.28 | 0 | 2 | 42 | 0 | 0 | 0 |
| 12/18/1994 | 230.50 | 5.56 | 0 | 0 | 82 | 0 | 0 | 0 |
| 12/19/1994 | 220.87 | 5.56 | 0 | 0 | 84 | 0 | 0 | 0 |
| 12/20/1994 | 169.62 | 5.00 | 0 | 1 | 38 | 0 | 0 | 1 |
| 12/21/1994 | 148.95 | 5.28 | 0 | 4 | 45 | 0 | 0 | 0 |
| 12/22/1994 | 137.90 | 4.44 | 0 | 0 | 9 | 0 | 0 | 0 |
| 12/23/1994 | 121.76 | 3.89 | 0 | 0 | 2 | 0 | 0 | 0 |
| 12/24/1994 | 114.68 | 4.17 | 0 | 0 | 9 | 0 | 0 | 0 |
| 12/25/1994 | 113.55 | 4.72 | 0 | 0 | 24 | 0 | 0 | 0 |
| 12/26/1994 | 101.09 | 5.28 | 0 | 0 | 1 | 0 | 0 | 0 |
| 12/27/1994 | 152.63 | 6.11 | 0 | 0 | 0 | 0 | 0 | 0 |
| 12/28/1994 | 190.01 | 5.56 | 0 | 0 | 77 | 0 | 0 | 0 |
| 12/29/1994 | 153.19 | 5.00 | 0 | 0 | 4 | 0 | 0 | 0 |
| 12/30/1994 | 120.35 | 3.89 | 0 | 0 | 2 | 0 | 0 | 0 |
| 12/31/1994 | 100.24 | 2.78 | 1 | 0 | 1 | 0 | 1 | 1 |
| 1/1/1995 | 87.22 | 3.33 | 0 | 0 | 0 | 0 | 0 | 1 |
| 1/2/1995 | 75.89 | 3.33 | 0 | 0 | 6 | 0 | 0 | 0 |
| 1/3/1995 | 67.11 | 2.78 | 0 | 0 | 0 | 0 | 0 | 0 |
| 1/4/1995 | 64.28 | 2.78 | 0 | 0 | 0 | 0 | 0 | 0 |
| 1/5/1995 | 61.45 | 3.33 | 0 | 0 | 5 | 0 | 0 | 0 |

| Date | Discharge (cms) | Temp (C°) | Chinook | Coho | Steelhead | Cutthroat | Lamprey | Sucker |
|---|---|---|---|---|---|---|---|---|
| 1/6/1995 | 74.47 | 4.44 | 0 | 0 | 11 | 0 | 0 | 0 |
| 1/7/1995 | 83.82 | 5.00 | 0 | 0 | 36 | 0 | 0 | 0 |
| 1/8/1995 | 94.30 | 5.56 | 0 | 0 | 55 | 0 | 0 | 0 |
| 1/9/1995 | 325.64 | 6.67 | 0 | 0 | 84 | 0 | 0 | 0 |
| 1/10/1995 | 518.20 | 6.67 | 0 | 0 | 0 | 0 | 0 | 0 |
| 1/11/1995 | 421.92 | 6.67 | 0 | 0 | 0 | 0 | 0 | 0 |
| 1/12/1995 | 368.12 | 6.11 | 0 | 0 | 46 | 0 | 0 | 0 |
| 1/13/1995 | 515.37 | 6.67 | 0 | 0 | 6 | 0 | 0 | 0 |
| 1/14/1995 | 838.18 | 6.67 | 0 | 0 | 0 | 0 | 0 | 0 |
| 1/15/1995 | 569.17 | 6.11 | 0 | 0 | 1 | 0 | 0 | 0 |
| 1/16/1995 | 404.93 | 5.56 | 0 | 0 | 4 | 0 | 0 | 0 |
| 1/17/1995 | 278.92 | 5.56 | 0 | 0 | 7 | 0 | 0 | 0 |
| 1/18/1995 | 246.36 | 5.56 | 0 | 0 | 58 | 0 | 0 | 0 |
| 1/19/1995 | 246.64 | 5.00 | 0 | 0 | 50 | 0 | 0 | 0 |
| 1/20/1995 | 201.62 | 5.00 | 0 | 1 | 65 | 0 | 0 | 0 |
| 1/21/1995 | 167.07 | 5.00 | 0 | 0 | 47 | 0 | 0 | 0 |
| 1/22/1995 | 141.02 | 4.44 | 0 | 0 | 22 | 0 | 0 | 0 |
| 1/23/1995 | 124.88 | 5.00 | 0 | 0 | 53 | 0 | 0 | 0 |
| 1/24/1995 | 114.12 | 5.00 | 0 | 0 | 71 | 0 | 0 | 0 |
| 1/25/1995 | 104.77 | 5.00 | 0 | 0 | 35 | 0 | 0 | 0 |
| 1/26/1995 | 97.13 | 5.00 | 0 | 0 | 65 | 0 | 0 | 0 |
| 1/27/1995 | 96.28 | 5.00 | 0 | 0 | 44 | 0 | 0 | 0 |
| 1/28/1995 | 96.84 | 5.56 | 0 | 0 | 76 | 0 | 0 | 0 |
| 1/29/1995 | 137.34 | 6.11 | 0 | 0 | 218 | 0 | 0 | 0 |
| 1/30/1995 | 203.60 | 7.22 | 0 | 0 | 311 | 0 | 0 | 0 |
| 1/31/1995 | 416.26 | 7.22 | 0 | 0 | 65 | 0 | 0 | 0 |
| 2/1/1995 | 580.50 | 7.22 | 0 | 0 | 0 | 0 | 0 | 0 |
| 2/2/1995 | 560.67 | 6.67 | 0 | 0 | 0 | 0 | 0 | 0 |
| 2/3/1995 | 328.48 | 6.67 | 0 | 0 | 119 | 0 | 0 | 0 |
| 2/4/1995 | 245.22 | 6.67 | 0 | 0 | 104 | 0 | 0 | 0 |
| 2/5/1995 | 201.33 | 6.67 | 0 | 0 | 47 | 0 | 0 | 0 |
| 2/6/1995 | 169.62 | 6.67 | 0 | 0 | 71 | 0 | 0 | 0 |
| 2/7/1995 | 147.81 | 6.67 | 0 | 0 | 27 | 0 | 0 | 0 |
| 2/8/1995 | 125.73 | 6.11 | 0 | 0 | 16 | 0 | 0 | 0 |
| 2/9/1995 | 114.40 | 5.56 | 0 | 0 | 4 | 0 | 0 | 0 |
| 2/10/1995 | 102.22 | 5.00 | 0 | 0 | 5 | 0 | 0 | 0 |
| 2/11/1995 | 93.16 | 4.44 | 0 | 0 | 4 | 0 | 0 | 0 |
| 2/12/1995 | 89.76 | 5.00 | 0 | 0 | 8 | 0 | 0 | 0 |
| 2/13/1995 | 103.36 | 4.44 | 0 | 0 | 11 | 0 | 0 | 0 |
| 2/14/1995 | 92.60 | 3.89 | 0 | 0 | 0 | 0 | 0 | 0 |
| 2/15/1995 | 80.99 | 3.89 | 0 | 0 | 0 | 0 | 0 | 0 |
| 2/16/1995 | 76.74 | 3.33 | 0 | 0 | 1 | 0 | 0 | 0 |
| 2/17/1995 | 107.32 | 4.44 | 0 | 0 | 6 | 0 | 0 | 0 |
| 2/18/1995 | 450.24 | 6.67 | 0 | 0 | 35 | 0 | 0 | 0 |
| 2/19/1995 | 288.83 | 7.78 | 0 | 0 | 257 | 0 | 0 | 1 |

| Date | Discharge (cms) | Temp (C°) | Chinook | Coho | Steelhead | Cutthroat | Lamprey | Sucker |
|---|---|---|---|---|---|---|---|---|
| 2/20/1995 | 213.23 | 7.22 | 0 | 0 | 187 | 0 | 0 | 1 |
| 2/21/1995 | 162.82 | 7.78 | 0 | 0 | 89 | 0 | 0 | 1 |
| 2/22/1995 | 143.85 | 7.78 | 0 | 0 | 101 | 0 | 0 | 2 |
| 2/23/1995 | 122.90 | 7.22 | 0 | 0 | 58 | 0 | 0 | 0 |
| 2/24/1995 | 115.82 | 6.67 | 0 | 0 | 42 | 0 | 0 | 0 |
| 2/25/1995 | 110.15 | 7.78 | 0 | 0 | 49 | 0 | 0 | 1 |
| 2/26/1995 | 102.79 | 7.78 | 0 | 0 | 34 | 0 | 0 | 2 |
| 2/27/1995 | 94.30 | | 0 | 0 | 24 | 0 | 0 | 0 |
| 2/28/1995 | 89.76 | 7.78 | 0 | 0 | 5 | 0 | 0 | 0 |
| 3/1/1995 | 84.67 | 7.22 | 0 | 0 | 4 | 0 | 0 | 0 |
| 3/2/1995 | 80.14 | 6.67 | 0 | 0 | 2 | 0 | 0 | 0 |
| 3/3/1995 | 81.55 | 6.67 | 0 | 0 | 3 | 0 | 0 | 0 |
| 3/4/1995 | 86.93 | 6.67 | 0 | 0 | 4 | 0 | 0 | 0 |
| 3/5/1995 | 93.45 | 6.67 | 0 | 0 | 1 | 0 | 0 | 0 |
| 3/6/1995 | 91.18 | 5.56 | 0 | 0 | 1 | 0 | 0 | 0 |
| 3/7/1995 | 82.69 | 5.00 | 0 | 0 | 0 | 0 | 0 | 0 |
| 3/8/1995 | 77.59 | 5.56 | 0 | 0 | 2 | 0 | 0 | 0 |
| 3/9/1995 | 92.31 | 7.22 | 0 | 0 | 30 | 0 | 0 | 0 |
| 3/10/1995 | 163.11 | 6.67 | 0 | 0 | 170 | 0 | 0 | 1 |
| 3/11/1995 | 165.94 | 7.22 | 0 | 0 | 64 | 0 | 0 | 0 |
| 3/12/1995 | 147.81 | 7.22 | 0 | 0 | 91 | 0 | 0 | 0 |
| 3/13/1995 | 145.27 | 7.22 | 0 | 0 | 84 | 0 | 0 | 1 |
| 3/14/1995 | 153.48 | 7.78 | 0 | 0 | 216 | 0 | 0 | 6 |
| 3/15/1995 | 276.09 | 7.22 | 0 | 0 | 40 | 0 | 0 | 1 |
| 3/16/1995 | 227.67 | 7.22 | 0 | 0 | 15 | 0 | 0 | 0 |
| 3/17/1995 | 173.58 | 7.22 | 0 | 0 | 42 | 0 | 0 | 0 |
| 3/18/1995 | 203.88 | 8.33 | 0 | 0 | 189 | 0 | 0 | 2 |
| 3/19/1995 | 294.50 | 7.22 | 0 | 0 | 13 | 0 | 0 | 0 |
| 3/20/1995 | 308.65 | 7.22 | 0 | 0 | 8 | 0 | 0 | 0 |
| 3/21/1995 | 297.33 | 6.67 | 0 | 0 | 3 | 0 | 0 | 0 |
| 3/22/1995 | 230.50 | 6.11 | 0 | 0 | 0 | 0 | 0 | 0 |
| 3/23/1995 | 184.63 | 5.00 | 0 | 0 | 0 | 0 | 0 | 0 |
| 3/24/1995 | 157.72 | 5.56 | 0 | 0 | 3 | 0 | 0 | 0 |
| 3/25/1995 | 138.47 | 5.56 | 0 | 0 | 9 | 0 | 0 | 0 |
| 3/26/1995 | 120.91 | 6.11 | 0 | 0 | 2 | 0 | 0 | 0 |
| 3/27/1995 | 111.00 | 6.67 | 0 | 0 | 7 | 0 | 0 | 0 |
| 3/28/1995 | 106.75 | 7.78 | 0 | 0 | 7 | 0 | 0 | 0 |
| 3/29/1995 | 103.64 | 7.78 | 0 | 0 | 32 | 0 | 0 | 0 |
| 3/30/1995 | 96.84 | 8.33 | 0 | 0 | 29 | 0 | 0 | 2 |
| 3/31/1995 | 89.20 | 7.22 | 0 | 0 | 26 | 0 | 0 | 0 |
| 4/1/1995 | 89.20 | 8.89 | 0 | 0 | 90 | 0 | 0 | 45 |
| 4/2/1995 | 84.38 | 8.89 | 2 | 0 | 32 | 0 | 0 | 19 |
| 4/3/1995 | 81.27 | 9.44 | 0 | 0 | 18 | 0 | 0 | 21 |
| 4/4/1995 | 78.44 | 8.33 | 0 | 0 | 30 | 0 | 0 | 1 |
| 4/5/1995 | 76.74 | 8.33 | 2 | 0 | 57 | 0 | 0 | 7 |

| Date | Discharge (cms) | Temp (C°) | Chinook | Coho | Steelhead | Cutthroat | Lamprey | Sucker |
|---|---|---|---|---|---|---|---|---|
| 4/6/1995 | 93.45 | 8.89 | 6 | 0 | 79 | 0 | 0 | 52 |
| 4/7/1995 | 118.65 | 8.33 | 4 | 0 | 61 | 3 | 0 | 61 |
| 4/8/1995 | 216.62 | 7.22 | 1 | 0 | 6 | 0 | 0 | 2 |
| 4/9/1995 | 241.83 | 6.67 | 0 | 0 | 2 | 0 | 0 | 0 |
| 4/10/1995 | 205.58 | 6.67 | 0 | 0 | 0 | 0 | 0 | 0 |
| 4/11/1995 | 189.72 | 7.78 | 1 | 0 | 17 | 0 | 0 | 0 |
| 4/12/1995 | 200.20 | 8.33 | 16 | 0 | 107 | 0 | 0 | 10 |
| 4/13/1995 | 300.16 | 7.22 | 1 | 0 | 13 | 0 | 0 | 3 |
| 4/14/1995 | 325.64 | 7.22 | 0 | 0 | 0 | 0 | 0 | 0 |
| 4/15/1995 | 250.89 | 6.11 | 0 | 0 | 2 | 0 | 0 | 0 |
| 4/16/1995 | 195.39 | 7.22 | 1 | 0 | 2 | 0 | 0 | 0 |
| 4/17/1995 | 162.26 | 6.67 | 7 | 0 | 11 | 0 | 0 | 0 |
| 4/18/1995 | 163.95 | 6.67 | 2 | 0 | 6 | 0 | 0 | 0 |
| 4/19/1995 | 150.36 | 6.67 | 0 | 0 | 3 | 0 | 0 | 0 |
| 4/20/1995 | 175.28 | 6.67 | 10 | 0 | 5 | 0 | 0 | 0 |
| 4/21/1995 | 201.90 | 7.78 | 3 | 0 | 5 | 0 | 0 | 0 |
| 4/22/1995 | 183.21 | 8.89 | 9 | 0 | 21 | 0 | 0 | 10 |
| 4/23/1995 | 168.77 | 9.44 | 1 | 0 | 3 | 0 | 0 | 99 |
| 4/24/1995 | 156.31 | 11.11 | 2 | 0 | 2 | 0 | 0 | 212 |
| 4/25/1995 | 144.42 | 10.56 | 13 | 0 | 13 | 0 | 0 | 539 |
| 4/26/1995 | 127.14 | 9.44 | 35 | 0 | 7 | 0 | 0 | 6 |
| 4/27/1995 | 123.18 | 8.89 | 12 | 0 | 6 | 0 | 0 | 0 |
| 4/28/1995 | 129.41 | 8.89 | 71 | 0 | 2 | 0 | 0 | 25 |
| 4/29/1995 | 135.35 | 8.89 | 63 | 0 | 5 | 0 | 0 | 14 |
| 4/30/1995 | 165.37 | 9.44 | 51 | 0 | 8 | 0 | 0 | 28 |
| 5/1/1995 | 175.85 | 10.00 | 33 | 0 | 11 | 0 | 0 | 135 |
| 5/2/1995 | 294.50 |  | 58 | 0 | 7 | 0 | 0 | 34 |
| 5/3/1995 | 276.37 | 8.89 | 2 | 0 | 0 | 0 | 0 | 0 |
| 5/4/1995 | 216.34 | 8.89 | 16 | 0 | 7 | 0 | 0 | 0 |
| 5/5/1995 | 204.73 | 8.33 | 27 | 0 | 4 | 0 | 0 | 0 |
| 5/6/1995 | 188.02 | 7.78 | 2 | 0 | 0 | 0 | 0 | 0 |
| 5/7/1995 | 175.85 | 9.44 | 0 | 0 | 1 | 0 | 1 | 0 |
| 5/8/1995 | 158.29 | 10.00 | 22 | 0 | 2 | 0 | 1 | 5 |
| 5/9/1995 | 143.28 | 10.00 | 71 | 0 | 2 | 0 | 0 | 26 |
| 5/10/1995 | 134.51 | 9.44 | 157 | 0 | 7 | 0 | 0 | 1 |
| 5/11/1995 | 141.30 | 9.44 | 141 | 0 | 5 | 0 | 0 | 1 |
| 5/12/1995 | 169.90 | 9.44 | 65 | 0 | 5 | 0 | 0 | 0 |
| 5/13/1995 | 161.97 | 9.44 | 48 | 0 | 1 | 0 | 0 | 0 |
| 5/14/1995 | 141.02 | 10.56 | 1 | 0 | 0 | 0 | 0 | 31 |
| 5/15/1995 | 125.16 | 11.11 | 352 | 0 | 10 | 0 | 0 | 383 |
| 5/16/1995 | 113.83 | 11.11 | 279 | 0 | 6 | 0 | 0 | 261 |
| 5/17/1995 | 108.17 | 11.11 | 257 | 0 | 11 | 0 | 0 | 84 |
| 5/18/1995 | 102.51 | 12.78 | 121 | 0 | 1 | 0 | 0 | 123 |
| 5/19/1995 | 99.11 | 13.33 | 173 | 0 | 2 | 0 | 1 | 342 |
| 5/20/1995 | 94.30 | 13.89 | 112 | 0 | 4 | 0 | 0 | 324 |

| Date | Discharge (cms) | Temp (C°) | Chinook | Coho | Steelhead | Cutthroat | Lamprey | Sucker |
|---|---|---|---|---|---|---|---|---|
| 5/21/1995 | 92.31 | 14.44 | 280 | 0 | 3 | 0 | 2 | 294 |
| 5/22/1995 | 92.31 | 15.00 | 338 | 0 | 3 | 0 | 1 | 137 |
| 5/23/1995 | 88.91 | 15.00 | 117 | 0 | 2 | 0 | 0 | 77 |
| 5/24/1995 | 84.67 | 14.44 | 110 | 0 | 2 | 0 | 0 | 61 |
| 5/25/1995 | 78.44 | 15.56 | 104 | 0 | 0 | 0 | 0 | 132 |
| 5/26/1995 | 79.57 | 15.00 | 198 | 0 | 0 | 0 | 0 | 179 |
| 5/27/1995 | 76.46 | 15.00 | 64 | 0 | 1 | 0 | 0 | 62 |
| 5/28/1995 | 74.76 | 16.11 | 121 | 0 | 2 | 0 | 0 | 237 |
| 5/29/1995 | 72.21 | 16.67 | 275 | 0 | 4 | 0 | 1 | 321 |
| 5/30/1995 | 70.51 | 17.22 | 167 | 0 | 1 | 0 | 0 | 283 |
| 5/31/1995 | 70.51 | 17.22 | 65 | 0 | 2 | 0 | 0 | 374 |
| 6/1/1995 | 70.23 | 17.22 | 180 | 0 | 10 | 0 | 2 | 277 |
| 6/2/1995 | 66.26 | 16.67 | 48 | 0 | 6 | 0 | 2 | 681 |
| 6/3/1995 | 68.53 | 15.56 | 103 | 0 | 6 | 0 | 0 | 84 |
| 6/4/1995 | 63.71 | 14.44 | 154 | 0 | 17 | 1 | 1 | 84 |
| 6/5/1995 | 65.98 | 13.89 | 126 | 0 | 7 | 0 | 0 | 3 |
| 6/6/1995 | 75.04 | 12.22 | 17 | 0 | 6 | 0 | 0 | 1 |
| 6/7/1995 | 87.22 | 12.22 | 18 | 0 | 4 | 0 | 0 | 0 |
| 6/8/1995 | 89.76 | 13.33 | 33 | 0 | 3 | 0 | 0 | 78 |
| 6/9/1995 | 76.17 | 14.44 | 64 | 0 | 12 | 1 | 0 | 416 |
| 6/10/1995 | 69.66 | 13.89 | 94 | 0 | 15 | 0 | 0 | 276 |
| 6/11/1995 | 67.96 | 15.00 | 18 | 0 | 4 | 0 | 0 | 41 |
| 6/12/1995 | 66.54 | 14.44 | 23 | 0 | 6 | 0 | 0 | 5 |
| 6/13/1995 | 62.01 | | 21 | 0 | 14 | 0 | 0 | 178 |
| 6/14/1995 | 60.88 | 12.78 | 28 | 0 | 12 | 0 | 0 | 2 |
| 6/15/1995 | 85.23 | 13.89 | 12 | 0 | 5 | 0 | 1 | 413 |
| 6/16/1995 | 100.81 | 12.22 | 35 | 0 | 2 | 0 | 0 | 24 |
| 6/17/1995 | 84.95 | 12.78 | 31 | 0 | 17 | 0 | 1 | 2 |
| 6/18/1995 | 83.82 | 11.67 | 26 | 0 | 10 | 0 | 0 | 0 |
| 6/19/1995 | 140.17 | 11.67 | 11 | 0 | 9 | 0 | 2 | 217 |
| 6/20/1995 | 177.26 | 10.56 | 0 | 0 | 7 | 0 | 0 | 1 |
| 6/21/1995 | 146.40 | 11.67 | 1 | 0 | 6 | 0 | 0 | 4 |
| 6/22/1995 | 117.51 | 14.44 | 11 | 0 | 18 | 0 | 3 | 277 |
| 6/23/1995 | 98.54 | 16.11 | 30 | 0 | 28 | 0 | 4 | 41 |
| 6/24/1995 | 85.52 | 17.22 | 176 | 0 | 40 | 1 | 3 | 67 |
| 6/25/1995 | 77.59 | 17.78 | 120 | 0 | 29 | 1 | 0 | 196 |
| 6/26/1995 | 71.64 | 17.78 | 109 | 0 | 11 | 0 | 0 | 411 |
| 6/27/1995 | 67.11 | 18.33 | 122 | 0 | 56 | 0 | 4 | 860 |
| 6/28/1995 | 62.58 | 18.33 | 104 | 0 | 11 | 1 | 0 | 523 |
| 6/29/1995 | 56.63 | 18.89 | 111 | 0 | 87 | 1 | 0 | 58 |
| 6/30/1995 | 52.67 | 19.44 | 95 | 0 | 60 | 0 | 0 | 520 |
| 7/1/1995 | 51.54 | | 134 | 0 | 81 | 3 | 1 | 234 |
| 7/2/1995 | 50.40 | 18.89 | 75 | 0 | 52 | 1 | 3 | 149 |
| 7/3/1995 | 48.70 | 18.89 | 51 | 0 | 33 | 0 | 0 | 26 |
| 7/4/1995 | 48.70 | 18.89 | 39 | 0 | 41 | 0 | 0 | 43 |

| Date | Discharge (cms) | Temp (C°) | Chinook | Coho | Steelhead | Cutthroat | Lamprey | Sucker |
|---|---|---|---|---|---|---|---|---|
| 7/5/1995 | 45.87 | 18.89 | 22 | 0 | 34 | 2 | 0 | 54 |
| 7/6/1995 | 45.02 | 18.33 | 16 | 0 | 3 | 0 | 0 | 53 |
| 7/7/1995 | 45.59 | 17.78 | 20 | 0 | 51 | 0 | 0 | 64 |
| 7/8/1995 | 46.72 | 18.89 | 13 | 0 | 20 | 1 | 0 | 94 |
| 7/9/1995 | 46.44 | 18.33 | 48 | 0 | 58 | 1 | 1 | 154 |
| 7/10/1995 | 72.21 | 16.11 | 31 | 0 | 32 | 0 | 0 | 2349 |
| 7/11/1995 | 60.88 | 16.11 | 41 | 0 | 52 | 0 | 0 | 177 |
| 7/12/1995 | 52.95 | 16.11 | 37 | 0 | 89 | 0 | 0 | 7 |
| 7/13/1995 | 50.69 | 16.67 | 55 | 0 | 128 | 0 | 0 | 9 |
| 7/14/1995 | 43.89 | 17.78 | 55 | 0 | 142 | 0 | 0 | 0 |
| 7/15/1995 | 45.02 | 18.33 | 92 | 0 | 219 | 0 | 0 | 3 |
| 7/16/1995 | 45.02 | 19.44 | 53 | 0 | 116 | 0 | 3 | 13 |
| 7/17/1995 | 44.74 | 20.56 | 65 | 0 | 118 | 2 | 2 | 9 |
| 7/18/1995 | 38.79 | 19.44 | 81 | 0 | 148 | 4 | 0 | 2 |
| 7/19/1995 | 39.36 | 20.56 | 23 | 0 | 49 | 6 | 2 | 1 |
| 7/20/1995 | 43.04 | 20.56 | 20 | 0 | 58 | 5 | 1 | 5 |
| 7/21/1995 | 38.23 | 20.00 | 26 | 0 | 34 | 6 | 2 | 2 |
| 7/22/1995 | 36.81 | 19.44 | 8 | 0 | 29 | 2 | 0 | 1 |
| 7/23/1995 | 38.79 | 20.00 | 6 | 0 | 11 | 1 | 0 | 0 |
| 7/24/1995 | 35.40 | 20.00 | 3 | 0 | 13 | 1 | 0 | 0 |
| 7/25/1995 | 35.40 | 20.00 | 1 | 0 | 0 | 0 | 0 | 0 |
| 7/26/1995 | 35.11 | 19.44 | 13 | 0 | 24 | 1 | 0 | 0 |
| 7/27/1995 | 35.11 |  | 41 | 0 | 28 | 1 | 0 | 0 |
| 7/28/1995 | 34.83 |  | 51 | 0 | 25 | 2 | 0 | 0 |
| 7/29/1995 | 33.13 |  | 68 | 0 | 61 | 1 | 2 | 0 |
| 7/30/1995 | 30.30 |  | 17 | 0 | 41 | 2 | 0 | 0 |
| 7/31/1995 | 30.02 | 21.67 | 10 | 0 | 25 | 0 | 0 | 0 |
| 8/1/1995 | 29.45 | 22.22 | 30 | 0 | 60 | 1 | 0 | 0 |
| 8/2/1995 | 29.17 | 22.22 | 29 | 0 | 37 | 1 | 0 | 0 |
| 8/3/1995 | 30.58 | 22.22 | 15 | 0 | 29 | 2 | 1 | 0 |
| 8/4/1995 | 31.15 | 22.78 | 7 | 0 | 4 | 1 | 1 | 0 |
| 8/5/1995 | 30.58 | 22.78 | 15 | 0 | 17 | 0 | 1 | 0 |
| 8/6/1995 | 31.71 | 22.22 | 6 | 0 | 7 | 1 | 2 | 0 |
| 8/7/1995 | 31.43 | 20.56 | 3 | 0 | 4 | 1 | 0 | 0 |
| 8/8/1995 | 31.43 | 20.00 | 0 | 0 | 2 | 1 | 0 | 0 |
| 8/9/1995 | 29.45 | 20.56 | 0 | 0 | 3 | 0 | 0 | 0 |
| 8/10/1995 | 27.61 | 18.89 | 2 | 0 | 5 | 1 | 0 | 0 |
| 8/11/1995 | 31.43 | 19.44 | 3 | 0 | 1 | 0 | 0 | 0 |
| 8/12/1995 | 29.45 | 19.44 | 5 | 0 | 3 | 0 | 0 | 0 |
| 8/13/1995 | 29.17 | 20.00 | 8 | 0 | 3 | 0 | 0 | 0 |
| 8/14/1995 | 28.88 | 20.00 | 34 | 0 | 35 | 1 | 0 | 0 |
| 8/15/1995 | 27.95 | 19.44 | 44 | 0 | 54 | 1 | 0 | 0 |
| 8/16/1995 | 27.81 | 17.78 | 27 | 0 | 23 | 1 | 0 | 0 |
| 8/17/1995 | 28.88 | 17.78 | 5 | 0 | 15 | 0 | 0 | 0 |
| 8/18/1995 | 30.02 | 18.33 | 2 | 0 | 6 | 0 | 0 | 0 |

| Date | Discharge (cms) | Temp (C°) | Chinook | Coho | Steelhead | Cutthroat | Lamprey | Sucker |
|---|---|---|---|---|---|---|---|---|
| 8/19/1995 | 29.17 | 18.89 | 12 | 0 | 39 | 0 | 0 | 0 |
| 8/20/1995 | 28.60 | 19.44 | 45 | 0 | 31 | 1 | 0 | 0 |
| 8/21/1995 | 28.12 | 20.00 | 38 | 0 | 50 | 1 | 0 | 0 |
| 8/22/1995 | 26.99 | 20.00 | 32 | 0 | 27 | 0 | 0 | 0 |
| 8/23/1995 | 26.76 | 19.44 | 8 | 0 | 16 | 1 | 0 | 0 |
| 8/24/1995 | 26.65 | 18.89 | 12 | 0 | 42 | 2 | 0 | 0 |
| 8/25/1995 | 26.42 | 17.78 | 11 | 0 | 12 | 0 | 0 | 0 |
| 8/26/1995 | 28.26 | 17.78 | 6 | 0 | 14 | 0 | 0 | 0 |
| 8/27/1995 | 26.96 | 17.78 | 9 | 0 | 17 | 0 | 0 | 0 |
| 8/28/1995 | 25.77 | 18.89 | 4 | 0 | 11 | 0 | 0 | 0 |
| 8/29/1995 | 26.90 | 18.89 | 3 | 0 | 14 | 0 | 0 | 0 |
| 8/30/1995 | 25.09 | 18.89 | 4 | 0 | 26 | 0 | 0 | 0 |
| 8/31/1995 | 25.80 | 18.89 | 6 | 0 | 22 | 0 | 0 | 0 |
| 9/1/1995 | 25.34 | 20.00 | 24 | 0 | 21 | 0 | 0 | 0 |
| 9/2/1995 | 25.29 | 19.44 | 16 | 0 | 35 | 0 | 1 | 0 |
| 9/3/1995 | 23.98 | 18.33 | 14 | 0 | 16 | 0 | 0 | 0 |
| 9/4/1995 | 24.58 | 18.33 | 8 | 0 | 13 | 0 | 1 | 0 |
| 9/5/1995 | 24.81 | 18.33 | 3 | 0 | 13 | 0 | 0 | 0 |
| 9/6/1995 | 24.61 | 17.78 | 2 | 1 | 5 | 0 | 0 | 0 |
| 9/7/1995 | 27.33 | 17.78 | 10 | 0 | 9 | 0 | 0 | 0 |
| 9/8/1995 | 25.80 | 17.78 | 5 | 0 | 6 | 1 | 0 | 0 |
| 9/9/1995 | 27.10 | 18.33 | 7 | 1 | 8 | 1 | 0 | 0 |
| 9/10/1995 | 27.01 | 18.33 | 17 | 0 | 5 | 0 | 0 | 0 |
| 9/11/1995 | 25.97 | 18.89 | 8 | 0 | 4 | 0 | 0 | 0 |
| 9/12/1995 | 25.20 | 18.89 | 3 | 0 | 16 | 0 | 0 | 0 |
| 9/13/1995 | 25.82 | 18.89 | 6 | 3 | 5 | 1 | 0 | 0 |
| 9/14/1995 | 23.76 | 18.89 | 6 | 3 | 9 | 0 | 0 | 0 |
| 9/15/1995 | 25.80 | 17.78 | 9 | 1 | 11 | 1 | 0 | 0 |
| 9/16/1995 | 25.80 | 16.67 | 3 | 0 | 17 | 1 | 0 | 0 |
| 9/17/1995 | 24.83 | 16.67 | 0 | 4 | 3 | 0 | 0 | 0 |
| 9/18/1995 | 25.15 | 18.33 | 0 | 0 | 0 | 0 | 0 | 0 |
| 9/19/1995 | 22.54 | 18.33 | 3 | 2 | 10 | 0 | 0 | 0 |
| 9/20/1995 | 25.34 | 18.33 | 5 | 1 | 5 | 1 | 0 | 0 |
| 9/21/1995 | 24.66 | 17.78 | 4 | 3 | 5 | 0 | 0 | 0 |
| 9/22/1995 | 23.45 | 17.78 | 2 | 2 | 7 | 0 | 0 | 0 |
| 9/23/1995 | 23.39 | 17.22 | 2 | 1 | 8 | 0 | 0 | 0 |
| 9/24/1995 | 23.56 | 17.22 | 1 | 2 | 4 | 0 | 0 | 0 |
| 9/25/1995 | 24.72 | 16.67 | 3 | 2 | 0 | 0 | 0 | 0 |
| 9/26/1995 | 26.56 | 15.56 | 4 | 8 | 6 | 0 | 0 | 0 |
| 9/27/1995 | 29.17 | 15.56 | 4 | 25 | 3 | 0 | 0 | 0 |
| 9/28/1995 | 28.60 | 15.00 | 3 | 7 | 6 | 0 | 0 | 0 |
| 9/29/1995 | 32.56 | 13.89 | 2 | 9 | 4 | 0 | 0 | 0 |
| 9/30/1995 | 34.83 | 14.44 | 12 | 18 | 2 | 0 | 0 | 0 |
| 10/1/1995 | 34.55 | 14.44 | 0 | 0 | 0 | 0 | 0 | 0 |
| 10/2/1995 | 31.43 | 14.44 | 1 | 0 | 0 | 0 | 0 | 0 |

| Date | Discharge (cms) | Temp (C°) | Chinook | Coho | Steelhead | Cutthroat | Lamprey | Sucker |
|---|---|---|---|---|---|---|---|---|
| 10/3/1995 | 27.64 | 13.89 | 5 | 1 | 0 | 0 | 0 | 0 |
| 10/4/1995 | 30.58 | 13.33 | 6 | 46 | 11 | 0 | 0 | 0 |
| 10/5/1995 | 33.98 | 13.33 | 4 | 34 | 2 | 0 | 0 | 0 |
| 10/6/1995 | 29.73 | 12.78 | 7 | 25 | 5 | 0 | 0 | 0 |
| 10/7/1995 | 28.32 | 12.22 | 2 | 20 | 6 | 0 | 0 | 0 |
| 10/8/1995 | 27.72 | 11.67 | 2 | 4 | 0 | 0 | 0 | 0 |
| 10/9/1995 | 26.65 | 12.78 | 0 | 0 | 0 | 0 | 0 | 0 |
| 10/10/1995 | 27.16 | 12.78 | 9 | 21 | 7 | 0 | 0 | 0 |
| 10/11/1995 | 28.23 | 12.22 | 13 | 3 | 5 | 0 | 0 | 0 |
| 10/12/1995 | 43.32 | 11.67 | 22 | 37 | 10 | 0 | 0 | 0 |
| 10/13/1995 | 43.89 | 11.11 | 42 | 115 | 10 | 1 | 0 | 0 |
| 10/14/1995 | 35.40 | 11.67 | 7 | 58 | 8 | 0 | 0 | 0 |
| 10/15/1995 | 31.15 | 11.11 | 2 | 20 | 5 | 0 | 0 | 0 |
| 10/16/1995 | 29.45 | 12.22 | 124 | 8 | 7 | 0 | 0 | 0 |
| 10/17/1995 | 26.59 | 11.11 | 4 | 25 | 3 | 0 | 0 | 0 |
| 10/18/1995 | 25.57 | 11.67 | 3 | 5 | 0 | 0 | 0 | 0 |
| 10/19/1995 | 29.45 | 11.67 | 2 | 0 | 1 | 0 | 0 | 0 |
| 10/20/1995 | 28.23 | 11.11 | 11 | 47 | 4 | 0 | 0 | 0 |
| 10/21/1995 | 27.27 | 10.00 | 1 | 10 | 5 | 0 | 0 | 0 |
| 10/22/1995 | 27.35 | 10.00 | 1 | 17 | 2 | 0 | 0 | 0 |
| 10/23/1995 | 27.35 | 10.56 | 1 | 22 | 3 | 0 | 0 | 0 |
| 10/24/1995 | 26.67 | 10.00 | 1 | 8 | 1 | 0 | 0 | 0 |
| 10/25/1995 | 25.77 | 10.00 | 1 | 18 | 2 | 0 | 0 | 0 |
| 10/26/1995 | 26.53 | 10.00 | 0 | 0 | 0 | 0 | 0 | 0 |
| 10/27/1995 | 38.79 | 9.44 | 3 | 14 | 0 | 0 | 0 | 0 |
| 10/28/1995 | 36.25 | 10.00 | 6 | 75 | 18 | 0 | 0 | 0 |
| 10/29/1995 | 34.26 | 10.00 | 3 | 34 | 2 | 0 | 0 | 0 |
| 10/30/1995 | 32.56 | 9.44 | 0 | 5 | 0 | 0 | 0 | 0 |
| 10/31/1995 | 30.02 | 9.44 | 0 | 0 | 0 | 0 | 0 | 0 |
| 11/1/1995 | 30.87 | 9.44 | 6 | 13 | 3 | 0 | 0 | 0 |
| 11/2/1995 | 30.87 | 8.33 | 1 | 1 | 1 | 0 | 0 | 0 |
| 11/3/1995 | 32.56 | 6.67 | 2 | 2 | 1 | 0 | 0 | 0 |
| 11/4/1995 | 30.02 | 5.56 | 0 | 0 | 2 | 0 | 0 | 0 |
| 11/5/1995 | 28.60 | 6.11 | 0 | 0 | 0 | 0 | 0 | 0 |
| 11/6/1995 | 28.88 | 6.67 | 0 | 0 | 0 | 0 | 0 | 0 |
| 11/7/1995 | 32.00 | 8.33 | 0 | 0 | 0 | 0 | 0 | 0 |
| 11/8/1995 | 36.53 | 8.33 | 1 | 0 | 1 | 0 | 0 | 0 |
| 11/9/1995 | 59.18 | 8.33 | 0 | 50 | 13 | 0 | 0 | 0 |
| 11/10/1995 | 69.66 | 8.89 | 4 | 75 | 7 | 0 | 0 | 0 |
| 11/11/1995 | 64.28 | 8.89 | 1 | 68 | 7 | 0 | 0 | 0 |
| 11/12/1995 | 236.73 | 10.00 | 3 | 196 | 9 | 0 | 0 | 0 |
| 11/13/1995 | 196.24 | 10.56 | 5 | 109 | 17 | 0 | 0 | 0 |
| 11/14/1995 | 131.67 | 10.56 | 8 | 194 | 39 | 1 | 0 | 9 |
| 11/15/1995 | 90.05 | 10.00 | 1 | 50 | 9 | 1 | 0 | 0 |
| 11/16/1995 | 73.06 | 10.00 | 2 | 23 | 9 | 0 | 0 | 0 |

| Date | Discharge (cms) | Temp (C°) | Chinook | Coho | Steelhead | Cutthroat | Lamprey | Sucker |
|---|---|---|---|---|---|---|---|---|
| 11/17/1995 | 60.88 | 9.44 | 0 | 7 | 19 | 0 | 0 | 0 |
| 11/18/1995 | 64.56 | 9.44 | 0 | 6 | 12 | 1 | 0 | 0 |
| 11/19/1995 | 70.23 | 8.89 | 1 | 4 | 4 | 0 | 0 | 0 |
| 11/20/1995 | 60.60 | 8.33 | 0 | 3 | 8 | 1 | 0 | 0 |
| 11/21/1995 | 53.52 | 7.78 | 0 | 1 | 4 | 0 | 0 | 0 |
| 11/22/1995 | 51.82 | 8.33 | 0 | 0 | 5 | 0 | 0 | 0 |
| 11/23/1995 | 47.57 | 8.89 | 0 | 2 | 13 | 0 | 0 | 0 |
| 11/24/1995 | 48.99 | 10.00 | 1 | 2 | 7 | 0 | 0 | 0 |
| 11/25/1995 | 173.30 | 11.11 | 1 | 2 | 30 | 0 | 0 | 0 |
| 11/26/1995 | 356.79 | 12.78 | 0 | 16 | 5 | 0 | 0 | 0 |
| 11/27/1995 | 213.51 | 11.11 | 0 | 10 | 2 | 0 | 0 | 0 |
| 11/28/1995 | 385.11 | 12.22 | 0 | 0 | 0 | 0 | 0 | 0 |
| 11/29/1995 | 252.87 | 12.22 | 1 | 4 | 17 | 0 | 0 | 0 |
| 11/30/1995 | 419.09 | 11.67 | 0 | 0 | 1 | 0 | 0 | 0 |
| 12/1/1995 | 651.29 | 13.89 | 0 | 0 | 0 | 0 | 0 | 0 |
| 12/2/1995 | 410.59 | 14.44 | 0 | 0 | 7 | 0 | 0 | 0 |
| 12/3/1995 | 256.83 | 11.67 | 0 | 1 | 17 | 0 | 0 | 0 |
| 12/4/1995 | 270.43 | 8.33 | 0 | 0 | 20 | 0 | 0 | 0 |
| 12/5/1995 | 353.96 | 11.67 | 0 | 0 | 7 | 0 | 0 | 0 |
| 12/6/1995 | 447.41 | 12.78 | 0 | 1 | 8 | 0 | 0 | 0 |
| 12/7/1995 | 334.14 | 13.89 | 0 | 1 | 15 | 0 | 0 | 0 |
| 12/8/1995 | 288.83 | 14.44 | 0 | 0 | 18 | 0 | 0 | 0 |
| 12/9/1995 | 521.03 | 13.89 | 0 | 0 | 0 | 0 | 0 | 0 |
| 12/10/1995 | 427.58 | 13.89 | 0 | 1 | 20 | 0 | 0 | 0 |
| 12/11/1995 | 348.30 | 14.44 | 0 | 0 | 20 | 0 | 0 | 0 |
| 12/12/1995 | 790.04 | 15.00 | 0 | 0 | 0 | 0 | 0 | 0 |
| 12/13/1995 | 923.13 | 13.33 | 0 | 0 | 0 | 0 | 0 | 0 |
| 12/14/1995 | 586.16 | 13.33 | 0 | 0 | 0 | 0 | 0 | 0 |
| 12/15/1995 | 631.47 | 13.33 | 0 | 0 | 0 | 0 | 0 | 0 |
| 12/16/1995 | 407.76 | 12.22 | 0 | 0 | 9 | 0 | 0 | 0 |
| 12/17/1995 | 291.66 | 14.44 | 0 | 0 | 17 | 0 | 0 | 0 |
| 12/18/1995 | 238.71 | 14.44 | 0 | 0 | 25 | 0 | 0 | 0 |
| 12/19/1995 | 194.25 | 13.33 | 0 | 0 | 0 | 0 | 0 | 0 |
| 12/20/1995 | 166.50 | 11.11 | 0 | 0 | 0 | 0 | 0 | 0 |
| 12/21/1995 | 145.55 | 7.22 | 0 | 0 | 0 | 0 | 0 | 0 |
| 12/22/1995 | 130.26 | 6.11 | 0 | 0 | 5 | 0 | 0 | 0 |
| 12/23/1995 | 113.83 | | 0 | 0 | 2 | 0 | 0 | 0 |
| 12/24/1995 | 103.92 | | 0 | 0 | 1 | 0 | 0 | 1 |
| 12/25/1995 | 95.71 | | 0 | 0 | 0 | 0 | 0 | 0 |
| 12/26/1995 | 90.05 | | 0 | 0 | 0 | 0 | 0 | 0 |
| 12/27/1995 | 82.97 | 3.33 | 0 | 0 | 2 | 0 | 0 | 0 |
| 12/28/1995 | 84.95 | 4.44 | 0 | 0 | 7 | 0 | 0 | 0 |
| 12/29/1995 | 241.83 | 6.67 | 0 | 0 | 22 | 0 | 0 | 0 |
| 12/30/1995 | 577.66 | 7.78 | 0 | 0 | 5 | 0 | 0 | 0 |
| 12/31/1995 | 722.08 | | 0 | 0 | 0 | 0 | 0 | 0 |

| Date | Discharge (cms) | Temp (C°) | Chinook | Coho | Steelhead | Cutthroat | Lamprey | Sucker |
|---|---|---|---|---|---|---|---|---|
| 1/1/1996 | 373.78 | 6.67 | 0 | 0 | 28 | 0 | 0 | 0 |
| 1/2/1996 | 256.27 | 6.11 | 0 | 0 | 1 | 0 | 0 | 0 |
| 1/3/1996 | 206.43 | 7.22 | 0 | 0 | 31 | 0 | 0 | 0 |
| 1/4/1996 | 197.93 | 7.22 | 0 | 0 | 35 | 0 | 0 | 0 |
| 1/5/1996 | 177.83 | 6.11 | 0 | 0 | 15 | 0 | 0 | 0 |
| 1/6/1996 | 163.11 | 6.11 | 0 | 0 | 23 | 0 | 0 | 0 |
| 1/7/1996 | 153.19 | 6.67 | 0 | 0 | 41 | 0 | 0 | 0 |
| 1/8/1996 | 294.50 | 7.22 | 0 | 0 | 66 | 0 | 0 | 0 |
| 1/9/1996 | 376.61 | 7.78 | 0 | 0 | 75 | 0 | 0 | 0 |
| 1/10/1996 | 498.38 | 7.78 | 0 | 0 | 13 | 0 | 0 | 0 |
| 1/11/1996 | 317.15 | 6.67 | 0 | 0 | 22 | 0 | 0 | 0 |
| 1/12/1996 | 234.46 | 6.11 | 0 | 0 | 26 | 0 | 0 | 0 |
| 1/13/1996 | 188.87 | 6.11 | 0 | 0 | 20 | 0 | 0 | 0 |
| 1/14/1996 | 161.12 | 5.56 | 0 | 0 | 13 | 0 | 0 | 0 |
| 1/15/1996 | 167.92 | 7.22 | 0 | 0 | 61 | 0 | 0 | 0 |
| 1/16/1996 | 202.47 | 7.22 | 0 | 0 | 74 | 0 | 0 | 0 |
| 1/17/1996 | 207.85 | 6.11 | 0 | 0 | 6 | 0 | 0 | 0 |
| 1/18/1996 | 195.39 | 5.00 | 0 | 0 | 0 | 0 | 0 | 0 |
| 1/19/1996 | 331.31 | 6.11 | 0 | 0 | 3 | 0 | 0 | 0 |
| 1/20/1996 | 560.67 | 6.11 | 0 | 0 | 0 | 0 | 0 | 0 |
| 1/21/1996 | 577.66 | 6.11 | 0 | 0 | 0 | 0 | 0 | 0 |
| 1/22/1996 | 325.64 | 5.56 | 0 | 0 | 6 | 0 | 0 | 0 |
| 1/23/1996 | 255.70 | 5.00 | 0 | 0 | 10 | 0 | 0 | 0 |
| 1/24/1996 | 351.13 | 5.00 | 0 | 0 | 0 | 0 | 0 | 0 |
| 1/25/1996 | 263.35 | 4.44 | 0 | 0 | 2 | 0 | 0 | 0 |
| 1/26/1996 | 213.23 | 5.00 | 0 | 0 | 10 | 0 | 0 | 0 |
| 1/27/1996 | 302.99 | 5.00 | 0 | 0 | 2 | 0 | 0 | 0 |
| 1/28/1996 | 345.47 | 5.00 | 0 | 0 | 5 | 0 | 0 | 0 |
| 1/29/1996 | 256.83 | 5.00 | 0 | 0 | 0 | 0 | 0 | 0 |
| 1/30/1996 | 205.30 | 5.00 | 0 | 0 | 0 | 0 | 0 | 0 |
| 1/31/1996 | 178.96 | 4.44 | 0 | 0 | 20 | 0 | 0 | 0 |
| 2/1/1996 | 169.33 | 3.89 | 0 | 0 | 4 | 0 | 0 | 0 |
| 2/2/1996 | 154.61 | 3.89 | 0 | 0 | 6 | 0 | 0 | 0 |
| 2/3/1996 | 139.04 | 5.00 | 0 | 0 | 25 | 0 | 0 | 0 |
| 2/4/1996 | 164.52 | 5.56 | 0 | 0 | 57 | 0 | 0 | 0 |
| 2/5/1996 | 286.00 | 6.11 | 0 | 0 | 57 | 0 | 0 | 0 |
| 2/6/1996 | 603.15 | 6.11 | 0 | 0 | 0 | 0 | 0 | 0 |
| 2/7/1996 | 838.18 | 7.22 | 0 | 0 | 0 | 0 | 0 | 0 |
| 2/8/1996 | 676.77 | 7.22 | 0 | 0 | 0 | 0 | 0 | 0 |
| 2/9/1996 | 1016.57 | 7.22 | 0 | 0 | 0 | 0 | 0 | 0 |
| 2/10/1996 | 642.79 | 6.67 | 0 | 0 | 0 | 0 | 0 | 0 |
| 2/11/1996 | 433.25 | 6.67 | 0 | 0 | 36 | 0 | 0 | 0 |
| 2/12/1996 | 351.13 | 6.67 | 0 | 0 | 145 | 0 | 0 | 0 |
| 2/13/1996 | 314.32 | 6.67 | 0 | 0 | 76 | 0 | 0 | 0 |
| 2/14/1996 | 286.00 | 6.67 | 0 | 0 | 73 | 0 | 0 | 0 |

| Date | Discharge (cms) | Temp (C°) | Chinook | Coho | Steelhead | Cutthroat | Lamprey | Sucker |
|---|---|---|---|---|---|---|---|---|
| 2/15/1996 | 261.08 | 6.67 | 0 | 0 | 85 | 0 | 0 | 0 |
| 2/16/1996 | 244.66 | 7.22 | 0 | 0 | 73 | 0 | 0 | 0 |
| 2/17/1996 | 254.57 | 7.78 | 0 | 0 | 159 | 0 | 0 | 0 |
| 2/18/1996 | 294.50 | 7.22 | 0 | 0 | 118 | 0 | 0 | 0 |
| 2/19/1996 | 421.92 | 6.67 | 0 | 0 | 1 | 0 | 0 | 0 |
| 2/20/1996 | 351.13 | 7.22 | 0 | 0 | 44 | 0 | 0 | 0 |
| 2/21/1996 | 342.63 | 5.56 | 0 | 0 | 0 | 0 | 0 | 0 |
| 2/22/1996 | 276.66 | 5.56 | 0 | 0 | 4 | 0 | 0 | 0 |
| 2/23/1996 | 264.48 | 5.00 | 0 | 0 | 0 | 0 | 0 | 0 |
| 2/24/1996 | 248.91 | 5.00 | 0 | 0 | 1 | 0 | 0 | 0 |
| 2/25/1996 | 200.48 | 5.00 | 0 | 0 | 1 | 0 | 0 | 0 |
| 2/26/1996 | 172.45 | 3.89 | 0 | 0 | 2 | 0 | 0 | 0 |
| 2/27/1996 | 154.33 | 3.89 | 0 | 0 | 0 | 0 | 0 | 0 |
| 2/28/1996 | 154.33 | 5.00 | 0 | 0 | 9 | 0 | 0 | 0 |
| 2/29/1996 | 141.30 | 6.11 | 0 | 0 | 45 | 0 | 0 | 0 |
| 3/1/1996 | 141.30 | 5.56 | 0 | 0 | 7 | 0 | 0 | 0 |
| 3/2/1996 | 147.53 | 5.00 | 0 | 0 | 10 | 0 | 0 | 0 |
| 3/3/1996 | 164.80 | 6.67 | 0 | 0 | 70 | 0 | 0 | 0 |
| 3/4/1996 | 219.46 | 6.11 | 0 | 0 | 271 | 0 | 0 | 0 |
| 3/5/1996 | 245.22 | 6.11 | 0 | 0 | 69 | 0 | 0 | 0 |
| 3/6/1996 | 212.38 | 6.67 | 0 | 0 | 24 | 0 | 0 | 0 |
| 3/7/1996 | 177.83 | 7.22 | 0 | 0 | 92 | 0 | 0 | 0 |
| 3/8/1996 | 176.98 | 7.78 | 0 | 0 | 123 | 1 | 0 | 0 |
| 3/9/1996 | 194.82 | 7.78 | 0 | 0 | 104 | 0 | 0 | 0 |
| 3/10/1996 | 196.52 | 7.78 | 0 | 0 | 84 | 0 | 0 | 2 |
| 3/11/1996 | 209.83 | 7.78 | 0 | 0 | 82 | 0 | 0 | 1 |
| 3/12/1996 | 228.52 | 7.78 | 0 | 0 | 18 | 0 | 0 | 0 |
| 3/13/1996 | 200.48 | 7.78 | 0 | 0 | 14 | 0 | 0 | 0 |
| 3/14/1996 | 173.30 | 7.78 | 0 | 0 | 8 | 0 | 0 | 0 |
| 3/15/1996 | 150.93 | 7.22 | 0 | 0 | 14 | 0 | 0 | 0 |
| 3/16/1996 | 135.64 | 7.78 | 0 | 0 | 13 | 0 | 0 | 0 |
| 3/17/1996 | 125.16 | 8.33 | 0 | 0 | 19 | 0 | 0 | 0 |
| 3/18/1996 | 118.93 | 8.89 | 0 | 0 | 72 | 0 | 0 | 1 |
| 3/19/1996 | 115.82 | 8.89 | 0 | 0 | 87 | 0 | 0 | 4 |
| 3/20/1996 | 111.00 | 8.89 | 0 | 0 | 33 | 0 | 0 | 1 |
| 3/21/1996 | 106.75 | 8.89 | 0 | 0 | 21 | 1 | 0 | 1 |
| 3/22/1996 | 108.17 | 7.22 | 0 | 0 | 10 | 1 | 0 | 0 |
| 3/23/1996 | 109.59 | 7.78 | 0 | 0 | 16 | 0 | 0 | 0 |
| 3/24/1996 | 100.24 | 7.78 | 0 | 0 | 14 | 0 | 0 | 0 |
| 3/25/1996 | 94.01 | 7.78 | 2 | 0 | 5 | 0 | 0 | 0 |
| 3/26/1996 | 88.35 | 7.78 | 0 | 0 | 0 | 0 | 0 | 0 |
| 3/27/1996 | 92.60 | 6.11 | 0 | 0 | 0 | 0 | 0 | 0 |
| 3/28/1996 | 99.39 | 6.67 | 0 | 0 | 1 | 0 | 0 | 0 |
| 3/29/1996 | 92.60 | 6.11 | 1 | 0 | 16 | 0 | 0 | 0 |
| 3/30/1996 | 89.20 | 6.67 | 1 | 0 | 11 | 0 | 0 | 0 |

| Date | Discharge (cms) | Temp (C°) | Chinook | Coho | Steelhead | Cutthroat | Lamprey | Sucker |
|---|---|---|---|---|---|---|---|---|
| 3/31/1996 | 109.87 | 7.22 | 13 | 0 | 85 | 0 | 0 | 0 |
| 4/1/1996 | 291.66 | 7.78 | 1 | 0 | 96 | 0 | 0 | 0 |
| 4/2/1996 | 274.39 | 7.78 | 4 | 0 | 200 | 0 | 0 | 0 |
| 4/3/1996 | 219.46 | 8.89 | 5 | 0 | 196 | 0 | 0 | 10 |
| 4/4/1996 | 178.40 | 8.89 | 5 | 0 | 151 | 0 | 0 | 12 |
| 4/5/1996 | 152.63 | 9.44 | 3 | 0 | 132 | 0 | 0 | 137 |
| 4/6/1996 | 136.49 | 11.11 | 3 | 0 | 48 | 0 | 0 | 960 |
| 4/7/1996 | 131.67 | 11.67 | 1 | 0 | 11 | 0 | 0 | 480 |
| 4/8/1996 | 130.82 | 12.22 | 8 | 0 | 61 | 0 | 0 | 735 |
| 4/9/1996 | 129.12 | 11.11 | 4 | 0 | 15 | 0 | 0 | 118 |
| 4/10/1996 | 134.51 | 10.00 | 10 | 0 | 9 | 0 | 0 | 0 |
| 4/11/1996 | 125.44 | 8.33 | 1 | 0 | 5 | 0 | 0 | 0 |
| 4/12/1996 | 144.98 | 7.78 | 3 | 0 | 3 | 0 | 0 | 0 |
| 4/13/1996 | 157.44 | 7.78 | 0 | 0 | 2 | 0 | 0 | 0 |
| 4/14/1996 | 149.23 | 8.33 | 0 | 0 | 9 | 0 | 0 | 0 |
| 4/15/1996 | 140.45 | 8.33 | 4 | 0 | 18 | 0 | 0 | 0 |
| 4/16/1996 | 145.27 | 8.89 | 0 | 0 | 8 | 0 | 0 | 0 |
| 4/17/1996 | 146.68 | 7.78 | 0 | 0 | 0 | 0 | 0 | 0 |
| 4/18/1996 | 142.43 | 7.78 | 1 | 0 | 1 | 0 | 0 | 0 |
| 4/19/1996 | 136.77 | 7.78 | 4 | 0 | 1 | 0 | 0 | 0 |
| 4/20/1996 | 139.04 | 7.22 | 2 | 0 | 3 | 0 | 0 | 0 |
| 4/21/1996 | 146.11 | 8.33 | 23 | 0 | 5 | 0 | 0 | 0 |
| 4/22/1996 | 162.82 | 8.33 | 0 | 0 | 0 | 0 | 0 | 0 |
| 4/23/1996 | 334.14 | 7.78 | 1 | 0 | 36 | 0 | 0 | 0 |
| 4/24/1996 | 696.59 | 8.33 | 0 | 0 | 0 | 0 | 0 | 0 |
| 4/25/1996 | 538.02 | 7.78 | 0 | 0 | 9 | 0 | 0 | 0 |
| 4/26/1996 | 342.63 | 10.00 | 8 | 0 | 111 | 0 | 0 | 0 |
| 4/27/1996 | 255.70 | 10.00 | 7 | 0 | 14 | 0 | 0 | 3 |
| 4/28/1996 | 205.86 | 9.44 | 0 | 0 | 0 | 0 | 0 | 0 |
| 4/29/1996 | 175.00 | 10.56 | 0 | 0 | 20 | 0 | 0 | 21 |
| 4/30/1996 | 156.31 | 11.11 | 6 | 0 | 12 | 0 | 0 | 0 |
| 5/1/1996 | 143.85 | 12.22 | 35 | 0 | 18 | 0 | 0 | 215 |
| 5/2/1996 | 133.66 | 11.67 | 25 | 0 | 5 | 0 | 0 | 88 |
| 5/3/1996 | 125.44 | 10.56 | 49 | 0 | 1 | 0 | 0 | 8 |
| 5/4/1996 | 115.53 | 10.00 | 4 | 0 | 0 | 1 | 0 | 0 |
| 5/5/1996 | 106.19 | 10.56 | 26 | 0 | 0 | 0 | 0 | 1 |
| 5/6/1996 | 101.09 | 11.67 | 56 | 0 | 4 | 0 | 0 | 0 |
| 5/7/1996 | 95.99 | 11.67 | 341 | 0 | 18 | 0 | 0 | 30 |
| 5/8/1996 | 90.61 | 11.67 | 341 | 0 | 10 | 0 | 1 | 79 |
| 5/9/1996 | 86.08 | 11.11 | 135 | 0 | 5 | 0 | 0 | 119 |
| 5/10/1996 | 80.99 | 11.11 | 134 | 0 | 3 | 0 | 0 | 109 |
| 5/11/1996 | 79.29 | 12.22 | 142 | 0 | 3 | 0 | 0 | 834 |
| 5/12/1996 | 77.87 | 13.33 | 199 | 0 | 7 | 0 | 0 | 541 |
| 5/13/1996 | 80.14 | 12.78 | 130 | 0 | 3 | 1 | 0 | 739 |
| 5/14/1996 | 116.67 | 13.33 | 269 | 0 | 6 | 0 | 0 | 174 |

| Date | Discharge (cms) | Temp (C°) | Chinook | Coho | Steelhead | Cutthroat | Lamprey | Sucker |
|---|---|---|---|---|---|---|---|---|
| 5/15/1996 | 250.32 | 11.11 | 15 | 0 | 3 | 0 | 0 | 2 |
| 5/16/1996 | 336.97 | 10.56 | 3 | 0 | 0 | 0 | 0 | 0 |
| 5/17/1996 | 308.65 | 10.56 | 5 | 0 | 2 | 0 | 0 | 0 |
| 5/18/1996 | 322.81 | 10.00 | 0 | 0 | 3 | 0 | 0 | 0 |
| 5/19/1996 | 345.47 | 9.44 | 0 | 0 | 2 | 0 | 0 | 0 |
| 5/20/1996 | 311.49 | 10.56 | 7 | 0 | 10 | 0 | 0 | 0 |
| 5/21/1996 | 271.28 | 10.00 | 12 | 0 | 3 | 0 | 0 | 0 |
| 5/22/1996 | 410.59 | 9.44 | 0 | 0 | 0 | 0 | 0 | 0 |
| 5/23/1996 | 373.78 | 9.44 | 2 | 0 | 2 | 0 | 0 | 0 |
| 5/24/1996 | 286.00 | 10.56 | 10 | 0 | 4 | 0 | 0 | 0 |
| 5/25/1996 | 227.67 | 12.22 | 21 | 0 | 1 | 0 | 0 | 53 |
| 5/26/1996 | 189.72 | 11.11 | 129 | 0 | 14 | 0 | 0 | 15 |
| 5/27/1996 | 164.80 | 11.67 | 98 | 0 | 4 | 0 | 0 | 108 |
| 5/28/1996 | 145.83 | 11.67 | 281 | 0 | 5 | 0 | 0 | 28 |
| 5/29/1996 | 132.81 | 11.67 | 126 | 0 | 3 | 0 | 0 | 1 |
| 5/30/1996 | 120.63 | 13.33 | 102 | 0 | 7 | 0 | 1 | 88 |
| 5/31/1996 | 110.72 | 13.89 | 144 | 0 | 2 | 0 | 2 | 266 |
| 6/1/1996 | 102.79 | 15.00 | 92 | 0 | 4 | 0 | 2 | 261 |
| 6/2/1996 | 96.56 | 16.11 | 290 | 0 | 8 | 0 | 1 | 454 |
| 6/3/1996 | 92.03 | 17.22 | 160 | 0 | 9 | 0 | 2 | 393 |
| 6/4/1996 | 86.08 | 17.22 | 132 | 0 | 3 | 0 | 0 | 243 |
| 6/5/1996 | 84.67 | 17.78 | 126 | 0 | 12 | 0 | 1 | 274 |
| 6/6/1996 | 76.74 | 16.67 | 56 | 0 | 6 | 0 | 0 | 247 |
| 6/7/1996 | 69.66 | 16.67 | 118 | 0 | 14 | 0 | 0 | 148 |
| 6/8/1996 | 71.92 | 16.67 | 88 | 0 | 18 | 0 | 1 | 127 |
| 6/9/1996 | 70.79 | 16.67 | 59 | 0 | 13 | 0 | 1 | 57 |
| 6/10/1996 | 68.53 | 16.67 | 53 | 0 | 11 | 0 | 2 | 118 |
| 6/11/1996 | 65.98 | 17.22 | 82 | 0 | 30 | 0 | 0 | 101 |
| 6/12/1996 | 64.56 | 16.11 | 58 | 0 | 14 | 0 | 0 | 121 |
| 6/13/1996 | 62.01 | 16.67 | 85 | 0 | 22 | 0 | 0 | 73 |
| 6/14/1996 | 61.16 | 17.22 | 33 | 0 | 10 | 0 | 1 | 50 |
| 6/15/1996 | 58.90 | 16.67 | 29 | 0 | 11 | 0 | 1 | 189 |
| 6/16/1996 | 56.63 | 15.56 | 56 | 0 | 20 | 1 | 1 | 36 |
| 6/17/1996 | 56.92 | 15.00 | 19 | 0 | 17 | 0 | 0 | 8 |
| 6/18/1996 | 56.63 | 15.56 | 14 | 0 | 12 | 0 | 0 | 4 |
| 6/19/1996 | 56.07 | 15.00 | 26 | 0 | 31 | 0 | 0 | 10 |
| 6/20/1996 | 54.65 | 16.11 | 13 | 0 | 25 | 0 | 1 | 379 |
| 6/21/1996 | 53.80 | 15.56 | 40 | 0 | 29 | 0 | 0 | 13 |
| 6/22/1996 | 52.10 | 16.11 | 28 | 0 | 18 | 0 | 0 | 33 |
| 6/23/1996 | 53.24 | 16.11 | 6 | 0 | 4 | 0 | 0 | 227 |
| 6/24/1996 | 58.62 | 15.56 | 7 | 0 | 4 | 0 | 0 | 170 |
| 6/25/1996 | 58.33 | 16.67 | 16 | 0 | 46 | 0 | 0 | 117 |
| 6/26/1996 | 56.35 | 16.67 | 25 | 0 | 36 | 0 | 0 | 192 |
| 6/27/1996 | 61.45 | 15.56 | 40 | 0 | 55 | 0 | 1 | 583 |
| 6/28/1996 | 62.01 | 16.11 | 23 | 0 | 35 | 0 | 1 | 269 |

| Date | Discharge (cms) | Temp (C°) | Chinook | Coho | Steelhead | Cutthroat | Lamprey | Sucker |
|---|---|---|---|---|---|---|---|---|
| 6/29/1996 | 54.37 | 17.22 | 2 | 0 | 26 | 0 | 0 | 68 |
| 6/30/1996 | 51.25 | 18.33 | 25 | 0 | 46 | 0 | 2 | 160 |
| 7/1/1996 | 50.40 | 19.44 | 6 | 0 | 3 | 0 | 0 | 744 |
| 7/2/1996 | 48.70 | 20.00 | 2 | 0 | 2 | 0 | 0 | 603 |
| 7/3/1996 | 47.57 | 20.00 | 57 | 0 | 134 | 0 | 1 | 868 |
| 7/4/1996 | 47.01 | 18.89 | 49 | 0 | 105 | 3 | 0 | 1074 |
| 7/5/1996 | 46.44 | 18.89 | 15 | 0 | 44 | 0 | 0 | 143 |
| 7/6/1996 | 45.02 | 19.44 | 17 | 0 | 58 | 0 | 0 | 298 |
| 7/7/1996 | 44.74 | 20.00 | 6 | 0 | 43 | 0 | 0 | 79 |
| 7/8/1996 | 44.46 | 20.56 | 0 | 0 | 0 | 1 | 1 | 43 |
| 7/9/1996 | 42.76 | 19.44 | 45 | 0 | 85 | 4 | 3 | 98 |
| 7/10/1996 | 41.06 | 20.00 | 20 | 0 | 63 | 3 | 3 | 63 |
| 7/11/1996 | 40.78 | 21.11 | 21 | 0 | 46 | 3 | 5 | 452 |
| 7/12/1996 | 39.08 | 22.22 | 22 | 0 | 66 | 5 | 12 | 274 |
| 7/13/1996 | 39.93 | 22.78 | 21 | 0 | 49 | 5 | 6 | 42 |
| 7/14/1996 | 39.08 | 21.67 | 17 | 0 | 18 | 7 | 6 | 1 |
| 7/15/1996 | 39.64 | 22.22 | 15 | 0 | 25 | 3 | 5 | 2 |
| 7/16/1996 | 40.21 | 20.56 | 3 | 0 | 19 | 2 | 0 | 1 |
| 7/17/1996 | 38.51 | 18.89 | 2 | 0 | 4 | 0 | 0 | 0 |
| 7/18/1996 | 43.61 | 16.67 | 0 | 0 | 0 | 0 | 0 | 0 |
| 7/19/1996 | 43.04 | 17.78 | 0 | 0 | 0 | 0 | 0 | 0 |
| 7/20/1996 | 39.36 | 18.33 | 0 | 0 | 0 | 0 | 0 | 0 |
| 7/21/1996 | 39.36 | 19.44 | 7 | 0 | 12 | 2 | 0 | 33 |
| 7/22/1996 | 38.79 | 21.11 | 1 | 0 | 12 | 1 | 0 | 1 |
| 7/23/1996 | 36.53 | 22.22 | 18 | 0 | 69 | 1 | 1 | 7 |
| 7/24/1996 | 37.66 | 22.78 | 55 | 0 | 153 | 1 | 5 | 15 |
| 7/25/1996 | 36.53 | 23.33 | 18 | 0 | 114 | 2 | 4 | 4 |
| 7/26/1996 | 37.10 | 23.33 | 9 | 0 | 49 | 0 | 0 | 22 |
| 7/27/1996 | 35.40 | 22.78 | 10 | 0 | 26 | 0 | 0 | 12 |
| 7/28/1996 | 37.10 | 21.67 | 3 | 0 | 8 | 0 | 1 | 6 |
| 7/29/1996 | 38.23 | 22.22 | 0 | 0 | 1 | 0 | 0 | 1 |
| 7/30/1996 | 36.81 | 21.67 | 0 | 0 | 0 | 0 | 0 | 0 |
| 7/31/1996 | 35.96 | 21.67 | 0 | 0 | 0 | 0 | 0 | 0 |
| 8/1/1996 | 31.71 | 21.11 | 0 | 0 | 2 | 0 | 0 | 0 |
| 8/2/1996 | 34.26 | 18.89 | 1 | 0 | 2 | 0 | 0 | 0 |
| 8/3/1996 | 34.26 | 19.44 | 0 | 0 | 0 | 0 | 0 | 0 |
| 8/4/1996 | 33.98 | 19.44 | 0 | 0 | 1 | 0 | 0 | 1 |
| 8/5/1996 | 33.98 | 18.89 | 1 | 0 | 0 | 0 | 0 | 0 |
| 8/6/1996 | 33.98 | 18.89 | 2 | 0 | 3 | 0 | 0 | 0 |
| 8/7/1996 | 32.85 | 20.00 | 7 | 0 | 16 | 0 | 0 | 2 |
| 8/8/1996 | 32.56 | 21.11 | 8 | 0 | 24 | 0 | 0 | 0 |
| 8/9/1996 | 32.56 | 21.67 | 44 | 0 | 110 | 0 | 2 | 2 |
| 8/10/1996 | 32.56 | 22.22 | 31 | 0 | 62 | 1 | 1 | 1 |
| 8/11/1996 | 32.00 | 21.11 | 23 | 0 | 53 | 1 | 0 | 0 |
| 8/12/1996 | 30.58 | 21.11 | 4 | 0 | 28 | 0 | 0 | 0 |

| Date | Discharge (cms) | Temp (C°) | Chinook | Coho | Steelhead | Cutthroat | Lamprey | Sucker |
|---|---|---|---|---|---|---|---|---|
| 8/13/1996 | 26.02 | 22.22 | 5 | 0 | 16 | 0 | 0 | 0 |
| 8/14/1996 | 32.56 | 21.67 | 2 | 0 | 7 | 0 | 0 | 2 |
| 8/15/1996 | 28.60 | 21.67 | 2 | 0 | 18 | 0 | 1 | 0 |
| 8/16/1996 | 28.88 | 21.11 | 2 | 0 | 9 | 1 | 0 | 0 |
| 8/17/1996 | 30.02 | 20.00 | 0 | 0 | 4 | 0 | 0 | 0 |
| 8/18/1996 | 30.87 | 18.33 | 1 | 0 | 1 | 0 | 0 | 0 |
| 8/19/1996 | 29.45 | 17.78 | 0 | 0 | 2 | 0 | 0 | 0 |
| 8/20/1996 | 28.01 | 17.78 | 0 | 0 | 0 | 0 | 0 | 0 |
| 8/21/1996 | 28.88 | 18.33 | 2 | 0 | 0 | 0 | 0 | 0 |
| 8/22/1996 | 29.45 | 18.33 | 16 | 0 | 10 | 0 | 0 | 0 |
| 8/23/1996 | 29.17 | 18.33 | 16 | 0 | 9 | 0 | 0 | 0 |
| 8/24/1996 | 29.45 | 18.33 | 18 | 0 | 25 | 0 | 0 | 0 |
| 8/25/1996 | 26.45 | 18.89 | 36 | 0 | 30 | 0 | 0 | 0 |
| 8/26/1996 | 24.61 | 18.89 | 14 | 0 | 12 | 0 | 0 | 0 |
| 8/27/1996 | 29.45 | 18.33 | 20 | 0 | 40 | 0 | 0 | 0 |
| 8/28/1996 | 30.30 | 17.78 | 2 | 0 | 9 | 0 | 0 | 0 |
| 8/29/1996 | 29.45 | 17.78 | 0 | 0 | 7 | 0 | 0 | 0 |
| 8/30/1996 | 28.09 | 18.33 | 13 | 0 | 29 | 0 | 0 | 0 |
| 8/31/1996 | 28.60 | 17.78 | 6 | 0 | 20 | 0 | 1 | 0 |
| 9/1/1996 | 26.99 | 18.89 | 9 | 0 | 21 | 1 | 0 | 0 |
| 9/2/1996 | 27.04 | 18.33 | 7 | 0 | 24 | 1 | 0 | 0 |
| 9/3/1996 | 30.30 | 17.22 | 4 | 0 | 29 | 1 | 0 | 0 |
| 9/4/1996 | 29.45 | 16.11 | 2 | 0 | 13 | 0 | 0 | 0 |
| 9/5/1996 | 28.60 | 16.11 | 3 | 0 | 8 | 1 | 0 | 0 |
| 9/6/1996 | 26.99 | 16.11 | 8 | 0 | 10 | 1 | 0 | 0 |
| 9/7/1996 | 26.73 | 16.67 | 3 | 0 | 8 | 0 | 0 | 0 |
| 9/8/1996 | 25.23 | 16.67 | 8 | 0 | 16 | 0 | 0 | 0 |
| 9/9/1996 | 25.34 | 16.67 | 11 | 0 | 12 | 0 | 0 | 1 |
| 9/10/1996 | 25.60 | 17.78 | 13 | 0 | 14 | 0 | 0 | 0 |
| 9/11/1996 | 29.45 | 17.22 | 10 | 0 | 18 | 0 | 0 | 0 |
| 9/12/1996 | 27.61 | 16.67 | 13 | 0 | 18 | 0 | 0 | 1 |
| 9/13/1996 | 27.72 | 15.56 | 3 | 0 | 8 | 1 | 0 | 0 |
| 9/14/1996 | 29.17 | 15.00 | 4 | 0 | 9 | 0 | 0 | 0 |
| 9/15/1996 | 32.85 | 14.44 | 3 | 0 | 2 | 0 | 0 | 0 |
| 9/16/1996 | 51.25 | 13.33 | 47 | 0 | 26 | 1 | 0 | 16 |
| 9/17/1996 | 54.09 | 12.78 | 22 | 1 | 15 | 2 | 0 | 6 |
| 9/18/1996 | 40.21 | 12.22 | 5 | 0 | 8 | 0 | 0 | 1 |
| 9/19/1996 | 36.25 | 13.33 | 5 | 1 | 31 | 0 | 0 | 0 |
| 9/20/1996 | 32.28 | 12.78 | 3 | 3 | 8 | 0 | 0 | 0 |
| 9/21/1996 | 34.26 | 12.22 | 3 | 1 | 3 | 0 | 0 | 0 |
| 9/22/1996 | 32.28 | 12.22 | 2 | 2 | 6 | 0 | 0 | 0 |
| 9/23/1996 | 31.71 | 12.22 | 0 | 0 | 1 | 0 | 0 | 0 |
| 9/24/1996 | 31.15 | 11.67 | 0 | 0 | 2 | 0 | 0 | 0 |
| 9/25/1996 | 30.87 | 11.67 | 0 | 2 | 2 | 0 | 0 | 0 |
| 9/26/1996 | 30.87 | 12.22 | 0 | 3 | 0 | 0 | 0 | 0 |

| Date | Discharge (cms) | Temp (C°) | Chinook | Coho | Steelhead | Cutthroat | Lamprey | Sucker |
|---|---|---|---|---|---|---|---|---|
| 9/27/1996 | 30.87 | 12.22 | 0 | 0 | 2 | 0 | 0 | 0 |
| 9/28/1996 | 29.45 | 13.33 | 1 | 0 | 1 | 2 | 0 | 0 |
| 9/29/1996 | 30.02 | 13.89 | 13 | 46 | 8 | 0 | 0 | 0 |
| 9/30/1996 | 29.73 | 14.44 | 16 | 31 | 34 | 0 | 0 | 0 |
| 10/1/1996 | 29.73 | 13.89 | 6 | 28 | 20 | 0 | 0 | 0 |
| 10/2/1996 | 30.02 | 13.89 | 1 | 5 | 8 | 0 | 0 | 0 |
| 10/3/1996 | 30.30 | 14.44 | 15 | 33 | 23 | 1 | 0 | 0 |
| 10/4/1996 | 30.02 | 14.44 | 5 | 64 | 21 | 0 | 0 | 0 |
| 10/5/1996 | 28.60 | 14.44 | 9 | 57 | 5 | 3 | 0 | 0 |
| 10/6/1996 | 28.60 | 15.00 | 20 | 16 | 7 | 0 | 0 | 0 |
| 10/7/1996 | 30.02 | 15.00 | 3 | 2 | 6 | 0 | 0 | 0 |
| 10/8/1996 | 29.73 | 15.00 | 21 | 79 | 19 | 0 | 0 | 0 |
| 10/9/1996 | 28.60 | 14.44 | 6 | 25 | 4 | 1 | 0 | 0 |
| 10/10/1996 | 29.17 | 14.44 | 0 | 0 | 0 | 0 | 0 | 0 |
| 10/11/1996 | 28.88 | 13.89 | 5 | 31 | 4 | 1 | 0 | 0 |
| 10/12/1996 | 28.60 | 12.78 | 4 | 11 | 3 | 0 | 0 | 1 |
| 10/13/1996 | 30.30 | 12.22 | 12 | 1 | 1 | 0 | 0 | 0 |
| 10/14/1996 | 33.98 | 11.67 | 2 | 5 | 4 | 0 | 0 | 0 |
| 10/15/1996 | 32.28 | 11.67 | 10 | 13 | 9 | 1 | 0 | 0 |
| 10/16/1996 | 32.28 | 11.11 | 4 | 20 | 0 | 0 | 0 | 0 |
| 10/17/1996 | 37.10 | 10.00 | 2 | 26 | 5 | 1 | 0 | 0 |
| 10/18/1996 | 37.66 | 9.44 | 3 | 4 | 0 | 0 | 0 | 0 |
| 10/19/1996 | 74.19 | 8.89 | 7 | 13 | 5 | 0 | 0 | 0 |
| 10/20/1996 | 53.24 | 8.33 | 3 | 2 | 3 | 0 | 0 | 0 |
| 10/21/1996 | 43.04 | 7.78 | 1 | 3 | 1 | 0 | 0 | 0 |
| 10/22/1996 | 39.93 | 7.78 | 2 | 13 | 3 | 0 | 0 | 0 |
| 10/23/1996 | 60.60 | 8.33 | 4 | 79 | 15 | 0 | 0 | 0 |
| 10/24/1996 | 101.66 | 8.89 | 23 | 228 | 58 | 1 | 0 | 0 |
| 10/25/1996 | 236.73 | 8.33 | 4 | 23 | 5 | 0 | 0 | 0 |
| 10/26/1996 | 184.06 | 7.22 | 11 | 79 | 6 | 0 | 0 | 0 |
| 10/27/1996 | 116.10 | 7.22 | 1 | 59 | 10 | 0 | 0 | 0 |
| 10/28/1996 | 93.73 | 6.67 | 1 | 26 | 3 | 1 | 0 | 0 |
| 10/29/1996 | 176.98 | 7.22 | 2 | 19 | 7 | 0 | 0 | 0 |
| 10/30/1996 | 141.30 | 7.78 | 7 | 50 | 24 | 0 | 0 | 0 |
| 10/31/1996 | 102.22 | 7.78 | 0 | 36 | 8 | 0 | 0 | 0 |
| 11/1/1996 | 83.53 | 6.67 | 0 | 9 | 4 | 0 | 0 | 0 |
| 11/2/1996 | 69.94 | 6.11 | 1 | 4 | 7 | 0 | 0 | 0 |
| 11/3/1996 | 62.86 | 6.67 | 0 | 13 | 2 | 0 | 0 | 0 |
| 11/4/1996 | 61.73 | 7.22 | 1 | 8 | 13 | 0 | 0 | 0 |
| 11/5/1996 | 59.75 | 6.67 | 0 | 15 | 8 | 0 | 0 | 0 |
| 11/6/1996 | 55.78 | 6.11 | 0 | 7 | 4 | 0 | 0 | 0 |
| 11/7/1996 | 62.01 | 7.22 | 1 | 12 | 9 | 0 | 0 | 0 |
| 11/8/1996 | 65.98 | 7.22 | 0 | 12 | 8 | 0 | 0 | 0 |
| 11/9/1996 | 61.73 | 7.22 | 0 | 9 | 6 | 0 | 0 | 0 |
| 11/10/1996 | 58.33 | 6.11 | 0 | 5 | 6 | 0 | 0 | 0 |

| Date | Discharge (cms) | Temp (C°) | Chinook | Coho | Steelhead | Cutthroat | Lamprey | Sucker |
|---|---|---|---|---|---|---|---|---|
| 11/11/1996 | 55.50 | 7.22 | 0 | 11 | 4 | 0 | 0 | 0 |
| 11/12/1996 | 52.10 | 7.78 | 0 | 11 | 11 | 0 | 0 | 0 |
| 11/13/1996 | 52.95 | 7.78 | 0 | 11 | 19 | 0 | 0 | 0 |
| 11/14/1996 | 67.39 | 7.78 | 1 | 7 | 9 | 0 | 0 | 0 |
| 11/15/1996 | 102.51 | 7.78 | 0 | 12 | 22 | 1 | 0 | 0 |
| 11/16/1996 | 116.38 | 7.22 | 3 | 3 | 12 | 0 | 0 | 0 |
| 11/17/1996 | 135.64 | 6.67 | 1 | 6 | 10 | 1 | 0 | 0 |
| 11/18/1996 | 872.16 | 7.78 | 0 | 3 | 3 | 0 | 0 | 0 |
| 11/19/1996 | 1631.05 | | 0 | 18 | 0 | 0 | 0 | 0 |
| 11/20/1996 | 756.06 | | 0 | 0 | 0 | 0 | 0 | 0 |
| 11/21/1996 | 387.94 | | 0 | 0 | 0 | 0 | 0 | 0 |
| 11/22/1996 | 291.66 | | 0 | 0 | 0 | 0 | 0 | 0 |
| 11/23/1996 | 236.73 | | 0 | 0 | 0 | 0 | 0 | 0 |
| 11/24/1996 | 227.38 | | 0 | 0 | 38 | 1 | 0 | 0 |
| 11/25/1996 | 302.99 | | 0 | 0 | 0 | 0 | 0 | 0 |
| 11/26/1996 | 237.86 | | 0 | 0 | 0 | 0 | 0 | 0 |
| 11/27/1996 | 186.89 | 7.22 | 0 | 4 | 9 | 0 | 0 | 0 |
| 11/28/1996 | 314.32 | 7.78 | 0 | 2 | 15 | 0 | 0 | 0 |
| 11/29/1996 | 362.46 | 7.22 | 0 | 0 | 0 | 0 | 0 | 0 |
| 11/30/1996 | 262.78 | 7.22 | 0 | 7 | 4 | 1 | 0 | 0 |
| 12/1/1996 | 543.68 | 7.22 | 0 | 0 | 0 | 0 | 0 | 0 |
| 12/2/1996 | 416.26 | 6.11 | 0 | 0 | 0 | 0 | 0 | 0 |
| 12/3/1996 | 319.98 | 6.11 | 0 | 0 | 0 | 0 | 0 | 0 |
| 12/4/1996 | 376.61 | 7.22 | 0 | 0 | 8 | 0 | 0 | 0 |
| 12/5/1996 | 1149.66 | 7.22 | 0 | 0 | 0 | 0 | 0 | 0 |
| 12/6/1996 | 617.31 | 6.11 | 0 | 0 | 0 | 0 | 0 | 0 |
| 12/7/1996 | 498.38 | 6.67 | 0 | 0 | 0 | 0 | 0 | 0 |
| 12/8/1996 | 1370.54 | 7.22 | 0 | 0 | 0 | 0 | 0 | 0 |
| 12/9/1996 | 1180.81 | | 0 | 0 | 0 | 0 | 0 | 0 |
| 12/10/1996 | 753.23 | | 0 | 0 | 0 | 0 | 0 | 0 |
| 12/11/1996 | 586.16 | | 0 | 0 | 0 | 0 | 0 | 0 |
| 12/12/1996 | 438.91 | | 0 | 0 | 0 | 0 | 0 | 0 |
| 12/13/1996 | 427.58 | | 0 | 0 | 0 | 0 | 0 | 0 |
| 12/14/1996 | 351.13 | | 0 | 0 | 0 | 0 | 0 | 0 |
| 12/15/1996 | 261.65 | | 0 | 0 | 0 | 0 | 0 | 0 |
| 12/16/1996 | 208.98 | | 0 | 0 | 1 | 0 | 0 | 0 |
| 12/17/1996 | 178.11 | | 0 | 0 | 6 | 0 | 0 | 0 |
| 12/18/1996 | 157.44 | 4.44 | 0 | 0 | 4 | 0 | 0 | 0 |
| 12/19/1996 | 140.17 | 3.89 | 0 | 0 | 0 | 0 | 0 | 0 |
| 12/20/1996 | 156.59 | 5.00 | 0 | 0 | 3 | 0 | 0 | 0 |
| 12/21/1996 | 251.74 | 5.56 | 0 | 0 | 15 | 1 | 0 | 0 |
| 12/22/1996 | 213.23 | 5.00 | 0 | 0 | 0 | 0 | 0 | 0 |
| 12/23/1996 | 187.17 | 5.56 | 0 | 0 | 20 | 0 | 0 | 0 |
| 12/24/1996 | 189.16 | 6.11 | 0 | 0 | 19 | 0 | 0 | 0 |
| 12/25/1996 | 580.50 | 7.22 | 0 | 0 | 0 | 0 | 0 | 0 |

| Date | Discharge (cms) | Temp (C°) | Chinook | Coho | Steelhead | Cutthroat | Lamprey | Sucker |
|---|---|---|---|---|---|---|---|---|
| 12/26/1996 | 1118.52 | 7.78 | 0 | 0 | 0 | 0 | 0 | 0 |
| 12/27/1996 | 775.88 | 7.78 | 0 | 0 | 0 | 0 | 0 | 0 |
| 12/28/1996 | 538.02 | | 0 | 0 | 0 | 0 | 0 | 0 |
| 12/29/1996 | 597.49 | 7.78 | 0 | 0 | 0 | 0 | 0 | 0 |
| 12/30/1996 | 756.06 | 7.78 | 0 | 0 | 0 | 0 | 0 | 0 |
| 12/31/1996 | 866.50 | 7.78 | 0 | 0 | 0 | 0 | 0 | 0 |
| 1/1/1997 | 1141.17 | 8.33 | 0 | 0 | 0 | 0 | 0 | 0 |
| 1/2/1997 | 968.44 | | 0 | 0 | 0 | 0 | 0 | 0 |
| 1/3/1997 | 775.88 | 7.22 | 0 | 0 | 0 | 0 | 0 | 0 |
| 1/4/1997 | 515.37 | 6.11 | 0 | 0 | 0 | 0 | 0 | 0 |
| 1/5/1997 | 362.46 | 5.00 | 0 | 0 | 0 | 0 | 0 | 0 |
| 1/6/1997 | 273.26 | 5.00 | 0 | 0 | 16 | 0 | 0 | 0 |
| 1/7/1997 | 222.29 | 5.56 | 0 | 0 | 1 | 0 | 0 | 0 |
| 1/8/1997 | 193.40 | 5.56 | 0 | 0 | 42 | 0 | 0 | 0 |
| 1/9/1997 | 171.88 | 5.56 | 0 | 0 | 21 | 0 | 0 | 0 |
| 1/10/1997 | 155.46 | 6.67 | 0 | 0 | 19 | 0 | 0 | 0 |
| 1/11/1997 | 144.13 | 5.56 | 0 | 0 | 6 | 0 | 0 | 0 |
| 1/12/1997 | 133.37 | 5.00 | 0 | 0 | 1 | 0 | 0 | 0 |
| 1/13/1997 | 120.63 | 3.33 | 0 | 0 | 0 | 0 | 0 | 0 |
| 1/14/1997 | 110.44 | 2.22 | 0 | 0 | 0 | 0 | 0 | 0 |
| 1/15/1997 | 106.75 | 2.78 | 0 | 0 | 0 | 0 | 0 | 0 |
| 1/16/1997 | 101.37 | 3.89 | 0 | 0 | 0 | 0 | 0 | 0 |
| 1/17/1997 | 103.36 | 5.00 | 0 | 0 | 2 | 0 | 0 | 0 |
| 1/18/1997 | 107.89 | 5.56 | 0 | 0 | 13 | 0 | 0 | 0 |
| 1/19/1997 | 107.04 | 5.56 | 0 | 0 | 69 | 0 | 0 | 0 |
| 1/20/1997 | 111.85 | 6.11 | 0 | 0 | 175 | 0 | 0 | 0 |
| 1/21/1997 | 129.97 | 5.56 | 0 | 0 | 28 | 0 | 0 | 0 |
| 1/22/1997 | 135.92 | 5.00 | 0 | 0 | 0 | 0 | 0 | 0 |
| 1/23/1997 | 123.74 | 4.44 | 0 | 0 | 1 | 0 | 0 | 0 |
| 1/24/1997 | 114.12 | 3.89 | 0 | 0 | 0 | 0 | 0 | 0 |
| 1/25/1997 | 143.57 | 4.44 | 0 | 0 | 1 | 0 | 0 | 0 |
| 1/26/1997 | 334.14 | 5.56 | 0 | 0 | 0 | 0 | 0 | 0 |
| 1/27/1997 | 269.86 | 6.11 | 0 | 0 | 85 | 0 | 0 | 0 |
| 1/28/1997 | 272.12 | 7.22 | 0 | 0 | 54 | 0 | 0 | 0 |
| 1/29/1997 | 297.33 | 7.22 | 0 | 0 | 49 | 0 | 0 | 0 |
| 1/30/1997 | 233.05 | 6.67 | 0 | 0 | 82 | 0 | 0 | 0 |
| 1/31/1997 | 540.85 | 7.78 | 0 | 0 | 0 | 0 | 0 | 0 |
| 2/1/1997 | 719.25 | 7.78 | 0 | 0 | 65 | 0 | 0 | 0 |
| 2/2/1997 | 419.09 | 7.22 | 0 | 0 | 0 | 0 | 0 | 0 |
| 2/3/1997 | 291.66 | 6.11 | 0 | 0 | 2 | 0 | 0 | 0 |
| 2/4/1997 | 228.80 | 6.67 | 0 | 0 | 24 | 0 | 0 | 0 |
| 2/5/1997 | 188.87 | 6.67 | 0 | 0 | 40 | 0 | 0 | 0 |
| 2/6/1997 | 163.39 | 5.00 | 0 | 0 | 0 | 0 | 0 | 0 |
| 2/7/1997 | 160.84 | 6.11 | 0 | 0 | 9 | 0 | 0 | 0 |
| 2/8/1997 | 160.27 | 6.67 | 0 | 0 | 33 | 0 | 0 | 0 |

| Date | Discharge (cms) | Temp (C°) | Chinook | Coho | Steelhead | Cutthroat | Lamprey | Sucker |
|---|---|---|---|---|---|---|---|---|
| 2/9/1997 | 143.57 | 6.11 | 0 | 0 | 19 | 0 | 0 | 0 |
| 2/10/1997 | 131.96 | 6.11 | 0 | 0 | 69 | 0 | 0 | 0 |
| 2/11/1997 | 122.33 | 6.11 | 0 | 0 | 30 | 0 | 0 | 0 |
| 2/12/1997 | 141.02 | 6.67 | 0 | 0 | 29 | 0 | 0 | 0 |
| 2/13/1997 | 148.66 | 6.11 | 0 | 0 | 11 | 0 | 0 | 0 |
| 2/14/1997 | 143.28 | 7.22 | 0 | 0 | 61 | 0 | 0 | 0 |
| 2/15/1997 | 141.87 | 7.78 | 0 | 0 | 54 | 0 | 0 | 0 |
| 2/16/1997 | 131.96 | 6.11 | 0 | 0 | 2 | 0 | 0 | 0 |
| 2/17/1997 | 133.94 | 7.78 | 0 | 0 | 102 | 1 | 0 | 0 |
| 2/18/1997 | 143.00 | 7.22 | 0 | 0 | 155 | 0 | 0 | 0 |
| 2/19/1997 | 201.62 | 7.22 | 0 | 0 | 208 | 1 | 0 | 0 |
| 2/20/1997 | 286.00 | 6.67 | 0 | 0 | 20 | 0 | 0 | 0 |
| 2/21/1997 | 214.92 | 6.67 | 0 | 0 | 21 | 0 | 0 | 0 |
| 2/22/1997 | 172.73 | 6.11 | 0 | 0 | 6 | 0 | 0 | 0 |
| 2/23/1997 | 148.95 | 6.11 | 0 | 0 | 2 | 0 | 0 | 0 |
| 2/24/1997 | 129.12 | 6.11 | 0 | 0 | 1 | 0 | 0 | 0 |
| 2/25/1997 | 115.25 | 5.56 | 0 | 0 | 1 | 0 | 0 | 0 |
| 2/26/1997 | 111.00 | 6.11 | 0 | 0 | 10 | 0 | 0 | 0 |
| 2/27/1997 | 130.82 | 6.67 | 0 | 0 | 81 | 0 | 0 | 0 |
| 2/28/1997 | 135.92 | 6.11 | 0 | 0 | 5 | 0 | 0 | 0 |
| 3/1/1997 | 140.73 | 5.56 | 0 | 0 | 12 | 0 | 0 | 0 |
| 3/2/1997 | 410.59 | 6.11 | 0 | 0 | 1 | 0 | 0 | 0 |
| 3/3/1997 | 305.82 | 6.11 | 0 | 0 | 0 | 0 | 0 | 0 |
| 3/4/1997 | 220.02 | 6.67 | 0 | 0 | 61 | 0 | 0 | 0 |
| 3/5/1997 | 179.53 | 5.56 | 0 | 0 | 63 | 0 | 0 | 0 |
| 3/6/1997 | 166.22 | 7.22 | 0 | 0 | 360 | 0 | 0 | 0 |
| 3/7/1997 | 192.55 | 7.22 | 0 | 0 | 303 | 1 | 0 | 0 |
| 3/8/1997 | 233.61 | 7.78 | 0 | 0 | 187 | 0 | 0 | 0 |
| 3/9/1997 | 203.60 | 7.78 | 0 | 0 | 118 | 0 | 0 | 0 |
| 3/10/1997 | 209.83 | 7.78 | 0 | 0 | 196 | 0 | 0 | 0 |
| 3/11/1997 | 275.24 | 7.78 | 0 | 0 | 138 | 0 | 0 | 0 |
| 3/12/1997 | 248.62 | 6.67 | 0 | 0 | 10 | 0 | 0 | 0 |
| 3/13/1997 | 200.20 | 6.67 | 0 | 0 | 2 | 0 | 0 | 0 |
| 3/14/1997 | 170.47 | 6.67 | 0 | 0 | 7 | 0 | 0 | 0 |
| 3/15/1997 | 163.95 | 7.78 | 1 | 0 | 63 | 0 | 0 | 0 |
| 3/16/1997 | 191.42 | 7.78 | 0 | 0 | 146 | 0 | 0 | 0 |
| 3/17/1997 | 254.85 | 7.78 | 0 | 0 | 125 | 0 | 0 | 0 |
| 3/18/1997 | 214.64 | 8.33 | 0 | 0 | 119 | 1 | 0 | 0 |
| 3/19/1997 | 182.36 | 8.89 | 0 | 0 | 128 | 0 | 0 | 0 |
| 3/20/1997 | 192.55 | 10.00 | 0 | 0 | 114 | 1 | 0 | 1 |
| 3/21/1997 | 190.01 | 8.89 | 0 | 0 | 17 | 0 | 0 | 0 |
| 3/22/1997 | 165.37 | 8.33 | 0 | 0 | 8 | 0 | 0 | 0 |
| 3/23/1997 | 147.25 | 8.89 | 0 | 0 | 23 | 0 | 0 | 0 |
| 3/24/1997 | 134.51 | 10.00 | 0 | 0 | 69 | 0 | 0 | 0 |
| 3/25/1997 | 125.73 | 10.56 | 0 | 0 | 172 | 1 | 0 | 160 |

| Date | Discharge (cms) | Temp (C°) | Chinook | Coho | Steelhead | Cutthroat | Lamprey | Sucker |
|---|---|---|---|---|---|---|---|---|
| 3/26/1997 | 122.05 | 9.44 | 2 | 0 | 47 | 0 | 0 | 2 |
| 3/27/1997 | 123.74 | 9.44 | 0 | 0 | 30 | 0 | 0 | 0 |
| 3/28/1997 | 125.16 | 9.44 | 0 | 0 | 8 | 0 | 0 | 0 |
| 3/29/1997 | 116.38 | 8.33 | 0 | 0 | 4 | 0 | 0 | 0 |
| 3/30/1997 | 109.30 | 7.22 | 0 | 0 | 1 | 0 | 0 | 0 |
| 3/31/1997 | 114.68 | 7.22 | 0 | 0 | 4 | 0 | 0 | 0 |
| 4/1/1997 | 115.82 | 7.22 | 0 | 0 | 0 | 0 | 0 | 0 |
| 4/2/1997 | 111.57 | 7.78 | 0 | 0 | 0 | 0 | 0 | 0 |
| 4/3/1997 | 107.32 | 8.33 | 1 | 0 | 16 | 0 | 0 | 0 |
| 4/4/1997 | 105.62 | 8.89 | 0 | 0 | 10 | 0 | 0 | 0 |
| 4/5/1997 | 103.64 | 8.89 | 0 | 0 | 6 | 1 | 0 | 0 |
| 4/6/1997 | 96.56 | 8.33 | 1 | 0 | 24 | 0 | 0 | 0 |
| 4/7/1997 | 94.30 | 8.33 | 4 | 0 | 24 | 0 | 0 | 0 |
| 4/8/1997 | 97.98 | 9.44 | 4 | 0 | 126 | 0 | 0 | 13 |
| 4/9/1997 | 106.75 | 8.89 | 5 | 0 | 29 | 0 | 0 | 0 |
| 4/10/1997 | 103.07 | 8.89 | 0 | 0 | 3 | 0 | 0 | 0 |
| 4/11/1997 | 96.28 | 10.00 | 2 | 0 | 35 | 0 | 0 | 52 |
| 4/12/1997 | 88.07 | 10.00 | 4 | 0 | 37 | 0 | 0 | 8 |
| 4/13/1997 | 86.65 | 8.89 | 12 | 0 | 23 | 0 | 0 | 0 |
| 4/14/1997 | 94.86 | 8.89 | 13 | 0 | 39 | 0 | 0 | 89 |
| 4/15/1997 | 109.30 | 11.11 | 7 | 0 | 131 | 0 | 0 | 120 |
| 4/16/1997 | 104.77 | 11.67 | 6 | 0 | 100 | 0 | 0 | 253 |
| 4/17/1997 | 102.79 | 12.22 | 7 | 0 | 30 | 1 | 0 | 547 |
| 4/18/1997 | 99.96 | 11.67 | 55 | 0 | 47 | 0 | 0 | 638 |
| 4/19/1997 | 126.58 | 10.56 | 20 | 0 | 47 | 0 | 0 | 1 |
| 4/20/1997 | 242.11 | 10.00 | 10 | 0 | 17 | 0 | 1 | 0 |
| 4/21/1997 | 252.30 | 9.44 | 8 | 0 | 12 | 0 | 0 | 0 |
| 4/22/1997 | 198.22 | 9.44 | 4 | 0 | 13 | 0 | 0 | 0 |
| 4/23/1997 | 256.55 | 10.00 | 25 | 0 | 25 | 0 | 0 | 0 |
| 4/24/1997 | 331.31 | 8.89 | 0 | 0 | 2 | 0 | 0 | 0 |
| 4/25/1997 | 254.57 | 10.00 | 2 | 0 | 10 | 0 | 0 | 0 |
| 4/26/1997 | 200.20 | 11.11 | 6 | 0 | 14 | 0 | 0 | 43 |
| 4/27/1997 | 179.81 | 11.67 | 5 | 0 | 6 | 0 | 0 | 41 |
| 4/28/1997 | 161.97 | 10.00 | 4 | 0 | 4 | 0 | 0 | 1 |
| 4/29/1997 | 175.28 | 10.56 | 24 | 0 | 1 | 0 | 0 | 0 |
| 4/30/1997 | 209.83 | 10.00 | 77 | 0 | 8 | 0 | 0 | 0 |
| 5/1/1997 | 245.22 | 10.00 | 1 | 0 | 1 | 0 | 0 | 0 |
| 5/2/1997 | 199.63 | 9.44 | 5 | 0 | 1 | 0 | 0 | 0 |
| 5/3/1997 | 173.02 | 10.56 | 13 | 0 | 3 | 0 | 0 | 0 |
| 5/4/1997 | 154.89 | 11.11 | 0 | 0 | 0 | 0 | 0 | 0 |
| 5/5/1997 | 137.90 | 12.22 | 2 | 0 | 5 | 0 | 0 | 251 |
| 5/6/1997 | 126.86 | 12.78 | 87 | 0 | 11 | 0 | 0 | 279 |
| 5/7/1997 | 117.80 | 12.78 | 36 | 0 | 5 | 0 | 0 | 383 |
| 5/8/1997 | 111.29 | 13.89 | 154 | 0 | 5 | 0 | 0 | 788 |
| 5/9/1997 | 105.34 | 14.44 | 80 | 0 | 0 | 0 | 0 | 791 |

| Date | Discharge (cms) | Temp (C°) | Chinook | Coho | Steelhead | Cutthroat | Lamprey | Sucker |
|---|---|---|---|---|---|---|---|---|
| 5/10/1997 | 105.06 | 15.00 | 53 | 0 | 4 | 0 | 0 | 871 |
| 5/11/1997 | 107.32 | 15.56 | 60 | 0 | 1 | 2 | 0 | 573 |
| 5/12/1997 | 110.15 | 15.56 | 307 | 0 | 3 | 0 | 0 | 769 |
| 5/13/1997 | 109.59 | 15.56 | 176 | 0 | 6 | 0 | 0 | 272 |
| 5/14/1997 | 105.06 | 16.11 | 61 | 0 | 2 | 0 | 0 | 411 |
| 5/15/1997 | 100.81 | 16.67 | 84 | 0 | 0 | 1 | 0 | 641 |
| 5/16/1997 | 96.56 | 16.11 | 135 | 0 | 1 | 0 | 1 | 323 |
| 5/17/1997 | 93.73 | 17.22 | 50 | 0 | 0 | 1 | 0 | 397 |
| 5/18/1997 | 90.33 | 17.22 | 95 | 0 | 0 | 1 | 0 | 440 |
| 5/19/1997 | 84.95 | 16.67 | 52 | 0 | 5 | 0 | 0 | 273 |
| 5/20/1997 | 80.99 | 16.11 | 27 | 0 | 2 | 0 | 0 | 52 |
| 5/21/1997 | 79.00 | 16.11 | 41 | 0 | 0 | 1 | 0 | 94 |
| 5/22/1997 | 77.02 | 15.00 | 43 | 0 | 1 | 1 | 0 | 5 |
| 5/23/1997 | 76.17 | 14.44 | 105 | 0 | 14 | 0 | 0 | 0 |
| 5/24/1997 | 79.29 | 13.33 | 24 | 0 | 4 | 0 | 0 | 0 |
| 5/25/1997 | 82.12 | 14.44 | 0 | 0 | 2 | 0 | 0 | 1 |
| 5/26/1997 | 75.32 | 13.89 | 94 | 0 | 1 | 0 | 0 | 32 |
| 5/27/1997 | 71.92 | 14.44 | 32 | 0 | 7 | 0 | 0 | 22 |
| 5/28/1997 | 72.77 | 14.44 | 291 | 0 | 12 | 1 | 0 | 429 |
| 5/29/1997 | 80.99 | 16.11 | 178 | 0 | 13 | 0 | 0 | 562 |
| 5/30/1997 | 80.42 | 17.78 | 80 | 0 | 7 | 0 | 2 | 789 |
| 5/31/1997 | 76.17 | 17.78 | 125 | 0 | 12 | 1 | 0 | 861 |
| 6/1/1997 | 78.15 | 16.11 | 48 | 0 | 6 | 1 | 0 | 134 |
| 6/2/1997 | 73.34 | 16.11 | 52 | 0 | 4 | 0 | 0 | 49 |
| 6/3/1997 | 72.49 | 15.00 | 69 | 0 | 9 | 0 | 0 | 4 |
| 6/4/1997 | 87.78 | 15.00 | 38 | 0 | 3 | 0 | 0 | 10 |
| 6/5/1997 | 95.43 | 15.56 | 31 | 0 | 3 | 0 | 0 | 119 |
| 6/6/1997 | 85.23 | 16.67 | 15 | 0 | 3 | 0 | 0 | 852 |
| 6/7/1997 | 77.30 | 15.56 | 129 | 0 | 3 | 0 | 0 | 58 |
| 6/8/1997 | 72.49 | 16.11 | 6 | 0 | 2 | 0 | 0 | 3 |
| 6/9/1997 | 70.51 | 17.22 | 53 | 0 | 7 | 0 | 0 | 425 |
| 6/10/1997 | 67.39 | 17.78 | 106 | 0 | 15 | 1 | 0 | 721 |
| 6/11/1997 | 64.00 | 16.11 | 169 | 0 | 7 | 0 | 0 | 401 |
| 6/12/1997 | 61.73 | 15.56 | 83 | 0 | 3 | 0 | 0 | 27 |
| 6/13/1997 | 62.58 | 16.11 | 0 | 0 | 1 | 0 | 0 | 0 |
| 6/14/1997 | 60.88 | 16.67 | 11 | 0 | 0 | 0 | 0 | 675 |
| 6/15/1997 | 58.62 | 18.33 | 0 | 0 | 0 | 0 | 0 | 0 |
| 6/16/1997 | 57.20 | 19.44 | 130 | 0 | 4 | 0 | 1 | 270 |
| 6/17/1997 | 55.78 | 18.33 | 24 | 0 | 26 | 0 | 0 | 355 |
| 6/18/1997 | 55.22 | 18.89 | 1 | 0 | 0 | 0 | 0 | 101 |
| 6/19/1997 | 53.52 | 18.89 | 52 | 0 | 16 | 0 | 0 | 88 |
| 6/20/1997 | 53.80 | 18.33 | 110 | 0 | 22 | 1 | 0 | 49 |
| 6/21/1997 | 53.24 | 17.22 | 58 | 0 | 43 | 0 | 0 | 58 |
| 6/22/1997 | 52.10 | 16.11 | 17 | 0 | 13 | 1 | 0 | 1 |
| 6/23/1997 | 51.54 | 16.11 | 15 | 0 | 25 | 1 | 0 | 0 |

| Date | Discharge (cms) | Temp (C°) | Chinook | Coho | Steelhead | Cutthroat | Lamprey | Sucker |
|---|---|---|---|---|---|---|---|---|
| 6/24/1997 | 50.69 | 16.67 | 12 | 0 | 19 | 1 | 0 | 11 |
| 6/25/1997 | 49.84 | 17.22 | 40 | 0 | 33 | 0 | 0 | 1106 |
| 6/26/1997 | 48.42 | 18.33 | 32 | 0 | 30 | 1 | 0 | 1118 |
| 6/27/1997 | 42.76 | 18.33 | 66 | 0 | 36 | 1 | 0 | 751 |
| 6/28/1997 | 46.16 | 17.22 | 25 | 0 | 40 | 0 | 0 | 103 |
| 6/29/1997 | 49.27 | 16.67 | 10 | 0 | 19 | 0 | 0 | 1 |
| 6/30/1997 | 63.71 | 16.11 | 21 | 0 | 18 | 0 | 0 | 106 |
| 7/1/1997 | 78.44 | 16.11 | 0 | 0 | 0 | 0 | 0 | 0 |
| 7/2/1997 | 65.98 | 17.22 | 1 | 0 | 3 | 1 | 0 | 335 |
| 7/3/1997 | 56.63 | 18.89 | 8 | 0 | 38 | 2 | 1 | 795 |
| 7/4/1997 | 52.67 | 20.00 | 41 | 0 | 50 | 1 | 1 | 857 |
| 7/5/1997 | 49.55 | 20.56 | 41 | 0 | 70 | 4 | 0 | 415 |
| 7/6/1997 | 48.42 | 21.11 | 26 | 0 | 51 | 3 | 0 | 126 |
| 7/7/1997 | 46.72 | 20.56 | 31 | 0 | 50 | 5 | 1 | 13 |
| 7/8/1997 | 47.01 | 20.00 | 9 | 0 | 9 | 0 | 0 | 24 |
| 7/9/1997 | 47.01 | 19.44 | 5 | 0 | 2 | 1 | 0 | 36 |
| 7/10/1997 | 47.29 | 17.78 | 6 | 0 | 52 | 2 | 0 | 1 |
| 7/11/1997 | 47.86 | 17.22 | 10 | 0 | 28 | 1 | 0 | 0 |
| 7/12/1997 | 44.74 | 18.33 | 8 | 0 | 19 | 1 | 0 | 0 |
| 7/13/1997 | 41.34 | 19.44 | 5 | 0 | 24 | 1 | 0 | 52 |
| 7/14/1997 | 42.19 | 20.56 | 11 | 0 | 26 | 0 | 0 | 46 |
| 7/15/1997 | 40.49 | 21.11 | 15 | 0 | 32 | 0 | 0 | 2 |
| 7/16/1997 | 40.49 | 21.67 | 16 | 0 | 7 | 1 | 0 | 4 |
| 7/17/1997 | 41.63 | 20.00 | 40 | 0 | 102 | 1 | 0 | 2 |
| 7/18/1997 | 41.91 | 19.44 | 3 | 0 | 21 | 1 | 0 | 0 |
| 7/19/1997 | 40.49 | 20.00 | 0 | 0 | 1 | 1 | 0 | 2 |
| 7/20/1997 | 40.21 | 21.67 | 1 | 0 | 0 | 0 | 0 | 0 |
| 7/21/1997 | 39.64 | 21.11 | 15 | 0 | 40 | 1 | 1 | 8 |
| 7/22/1997 | 39.08 | 21.11 | 11 | 0 | 42 | 5 | 0 | 2 |
| 7/23/1997 | 39.36 | 21.11 | 3 | 0 | 10 | 1 | 0 | 0 |
| 7/24/1997 | 39.08 | 21.11 | 13 | 0 | 27 | 0 | 0 | 5 |
| 7/25/1997 | 37.38 | 21.11 | 13 | 0 | 35 | 0 | 0 | 1 |
| 7/26/1997 | 36.81 | 21.11 | 6 | 0 | 30 | 1 | 1 | 4 |
| 7/27/1997 | 38.23 | 21.11 | 8 | 0 | 12 | 3 | 1 | 0 |
| 7/28/1997 | 35.96 | 21.67 | 3 | 0 | 5 | 0 | 0 | 0 |
| 7/29/1997 | 35.40 | 20.56 | 3 | 0 | 1 | 0 | 0 | 6 |
| 7/30/1997 | 35.11 | 20.00 | 0 | 0 | 37 | 0 | 0 | 0 |
| 7/31/1997 | 37.10 | 20.56 | 0 | 0 | 0 | 0 | 0 | 0 |
| 8/1/1997 | 35.68 | 21.11 | 7 | 0 | 5 | 0 | 0 | 0 |
| 8/2/1997 | 34.83 | 21.11 | 0 | 0 | 9 | 0 | 0 | 2 |
| 8/3/1997 | 33.98 | 21.67 | 7 | 0 | 11 | 0 | 1 | 0 |
| 8/4/1997 | 35.68 | 21.67 | 10 | 0 | 28 | 0 | 1 | 2 |
| 8/5/1997 | 35.40 | 22.22 | 13 | 0 | 20 | 0 | 1 | 0 |
| 8/6/1997 | 32.00 | 22.78 | 17 | 0 | 25 | 1 | 0 | 0 |
| 8/7/1997 | 30.30 | 22.78 | 11 | 0 | 20 | 0 | 0 | 2 |

| Date | Discharge (cms) | Temp (C°) | Chinook | Coho | Steelhead | Cutthroat | Lamprey | Sucker |
|---|---|---|---|---|---|---|---|---|
| 8/8/1997 | 33.13 | 21.67 | 5 | 0 | 18 | 1 | 1 | 0 |
| 8/9/1997 | 33.41 | 21.11 | 3 | 0 | 12 | 1 | 0 | 1 |
| 8/10/1997 | 33.41 | 21.67 | 1 | 0 | 9 | 1 | 0 | 2 |
| 8/11/1997 | 34.55 | 21.11 | 0 | 0 | 17 | 0 | 0 | 1 |
| 8/12/1997 | 35.68 | 21.67 | 2 | 0 | 4 | 1 | 0 | 0 |
| 8/13/1997 | 31.43 | 21.67 | 0 | 0 | 0 | 0 | 0 | 0 |
| 8/14/1997 | 33.70 | 21.67 | 0 | 0 | 0 | 0 | 0 | 0 |
| 8/15/1997 | 32.00 | 21.11 | 2 | 0 | 5 | 0 | 0 | 2 |
| 8/16/1997 | 32.56 | 21.11 | 2 | 0 | 5 | 0 | 0 | 0 |
| 8/17/1997 | 31.15 | 19.44 | 0 | 0 | 0 | 0 | 0 | 0 |
| 8/18/1997 | 33.13 | 18.89 | 0 | 0 | 1 | 0 | 0 | 1 |
| 8/19/1997 | 30.87 | 20.56 | 0 | 0 | 3 | 0 | 0 | 0 |
| 8/20/1997 | 30.02 | 20.00 | 3 | 0 | 5 | 1 | 0 | 18 |
| 8/21/1997 | 36.53 | 20.00 | 3 | 0 | 5 | 0 | 0 | 2 |
| 8/22/1997 | 34.83 | 20.00 | 6 | 0 | 8 | 1 | 0 | 0 |
| 8/23/1997 | 31.71 | 20.00 | 6 | 0 | 5 | 0 | 0 | 0 |
| 8/24/1997 | 35.40 | 20.00 | 13 | 0 | 2 | 0 | 0 | 0 |
| 8/25/1997 | 46.16 | 18.89 | 18 | 0 | 62 | 3 | 0 | 19 |
| 8/26/1997 | 38.51 | 17.78 | 7 | 0 | 19 | 0 | 0 | 0 |
| 8/27/1997 | 35.68 | 17.78 | 13 | 0 | 1 | 1 | 0 | 0 |
| 8/28/1997 | 36.25 | 17.22 | 0 | 0 | 0 | 0 | 0 | 0 |
| 8/29/1997 | 34.83 | 17.78 | 13 | 0 | 57 | 2 | 0 | 1 |
| 8/30/1997 | 33.41 | 18.33 | 6 | 0 | 26 | 0 | 0 | 0 |
| 8/31/1997 | 35.11 | 18.89 | 10 | 0 | 19 | 0 | 0 | 2 |
| 9/1/1997 | 35.11 | 18.89 | 22 | 0 | 15 | 1 | 0 | 5 |
| 9/2/1997 | 33.70 | 18.89 | 12 | 0 | 12 | 1 | 0 | 0 |
| 9/3/1997 | 34.26 | 19.44 | 12 | 0 | 15 | 0 | 0 | 0 |
| 9/4/1997 | 35.68 | | 0 | 0 | 0 | 0 | 0 | 0 |
| 9/5/1997 | 36.25 | | 0 | 0 | 0 | 0 | 0 | 0 |
| 9/6/1997 | 32.56 | | 0 | 0 | 0 | 0 | 0 | 0 |
| 9/7/1997 | 33.41 | | 0 | 0 | 0 | 0 | 0 | 0 |
| 9/8/1997 | 33.98 | | 0 | 0 | 0 | 0 | 0 | 0 |
| 9/9/1997 | 32.85 | | 0 | 0 | 0 | 0 | 0 | 0 |
| 9/10/1997 | 33.13 | | 0 | 0 | 0 | 0 | 0 | 0 |
| 9/11/1997 | 33.70 | | 0 | 0 | 0 | 0 | 0 | 0 |
| 9/12/1997 | 33.70 | | 0 | 0 | 0 | 0 | 0 | 0 |
| 9/13/1997 | 33.98 | | 0 | 0 | 0 | 0 | 0 | 0 |
| 9/14/1997 | 36.81 | | 0 | 0 | 0 | 0 | 0 | 0 |
| 9/15/1997 | 41.63 | | 0 | 0 | 0 | 0 | 0 | 0 |
| 9/16/1997 | 47.01 | | 0 | 0 | 0 | 0 | 0 | 0 |
| 9/17/1997 | 55.22 | 13.89 | 16 | 12 | 47 | 1 | 0 | 12 |
| 9/18/1997 | 77.02 | 12.78 | 8 | 8 | 4 | 0 | 0 | 5 |
| 9/19/1997 | 55.78 | 13.89 | 2 | 2 | 11 | 0 | 0 | 2 |
| 9/20/1997 | 45.59 | 14.44 | 3 | 0 | 1 | 1 | 0 | 0 |
| 9/21/1997 | 41.91 | 15.00 | 1 | 4 | 0 | 2 | 0 | 0 |

| Date | Discharge (cms) | Temp (C°) | Chinook | Coho | Steelhead | Cutthroat | Lamprey | Sucker |
|---|---|---|---|---|---|---|---|---|
| 9/22/1997 | 38.79 | 15.56 | 1 | 14 | 10 | 0 | 0 | 0 |
| 9/23/1997 | 34.55 | 15.00 | 7 | 43 | 10 | 1 | 0 | 0 |
| 9/24/1997 | 38.79 | 16.11 | 0 | 52 | 9 | 0 | 0 | 0 |
| 9/25/1997 | 35.96 | 15.56 | 6 | 83 | 10 | 1 | 0 | 1 |
| 9/26/1997 | 35.11 | 15.00 | 2 | 26 | 4 | 0 | 0 | 1 |
| 9/27/1997 | 35.40 | 15.00 | 1 | 16 | 0 | 0 | 0 | 0 |
| 9/28/1997 | 35.68 | 15.00 | 0 | 6 | 3 | 0 | 0 | 0 |
| 9/29/1997 | 35.40 | 15.00 | 3 | 15 | 8 | 1 | 0 | 0 |
| 9/30/1997 | 35.40 | 13.89 | 5 | 43 | 19 | 2 | 0 | 0 |
| 10/1/1997 | 37.10 | 13.33 | 3 | 4 | 1 | 0 | 0 | 0 |
| 10/2/1997 | 55.78 | 12.22 | 11 | 45 | 6 | 0 | 0 | 4 |
| 10/3/1997 | 60.03 | 12.78 | 27 | 79 | 9 | 2 | 0 | 47 |
| 10/4/1997 | 55.50 | 12.78 | 10 | 28 | 3 | 0 | 0 | 16 |
| 10/5/1997 | 73.34 | 13.33 | 3 | 2 | 0 | 0 | 0 | 3 |
| 10/6/1997 | 81.84 | 12.22 | 11 | 65 | 6 | 0 | 0 | 6 |
| 10/7/1997 | 60.88 | 11.11 | 1 | 50 | 4 | 0 | 0 | 0 |
| 10/8/1997 | 52.39 | 11.11 | 1 | 11 | 1 | 0 | 0 | 0 |
| 10/9/1997 | 96.28 | 10.56 | 0 | 3 | 0 | 0 | 0 | 0 |
| 10/10/1997 | 121.76 | 10.00 | 4 | 32 | 3 | 0 | 0 | 0 |
| 10/11/1997 | 91.46 | 10.00 | 6 | 71 | 5 | 1 | 0 | 0 |
| 10/12/1997 | 96.56 | 10.00 | 1 | 1 | 0 | 0 | 0 | 0 |
| 10/13/1997 | 91.75 | 10.00 | 0 | 20 | 3 | 0 | 0 | 0 |
| 10/14/1997 | 74.47 | 10.00 | 0 | 50 | 3 | 0 | 0 | 0 |
| 10/15/1997 | 65.13 | 11.11 | 1 | 42 | 2 | 0 | 0 | 0 |
| 10/16/1997 | 59.18 | 11.11 | 4 | 22 | 5 | 0 | 0 | 1 |
| 10/17/1997 | 54.65 | 10.56 | 0 | 14 | 5 | 0 | 0 | 0 |
| 10/18/1997 | 50.69 | 10.56 | 2 | 10 | 3 | 0 | 0 | 0 |
| 10/19/1997 | 47.57 | 11.11 | 0 | 1 | 0 | 0 | 0 | 0 |
| 10/20/1997 | 45.59 | 11.11 | 4 | 0 | 0 | 0 | 0 | 0 |
| 10/21/1997 | 43.32 | 10.56 | 0 | 0 | 0 | 0 | 0 | 0 |
| 10/22/1997 | 41.34 | 9.44 | 0 | 16 | 7 | 0 | 0 | 0 |
| 10/23/1997 | 40.49 | 10.00 | 0 | 12 | 2 | 1 | 0 | 0 |
| 10/24/1997 | 39.64 | 9.44 | 0 | 2 | 1 | 0 | 0 | 0 |
| 10/25/1997 | 39.93 | 8.89 | 1 | 4 | 0 | 0 | 0 | 0 |
| 10/26/1997 | 38.79 | 8.33 | 1 | 0 | 2 | 0 | 0 | 0 |
| 10/27/1997 | 38.23 | 8.89 | 0 | 2 | 0 | 0 | 0 | 0 |
| 10/28/1997 | 37.94 | 8.89 | 1 | 1 | 0 | 1 | 0 | 0 |
| 10/29/1997 | 39.36 | 9.44 | 0 | 0 | 0 | 0 | 0 | 0 |
| 10/30/1997 | 74.76 | 10.00 | 0 | 0 | 0 | 0 | 0 | 0 |
| 10/31/1997 | 280.05 | 11.11 | 0 | 6 | 2 | 0 | 0 | 0 |
| 11/1/1997 | 152.63 | 10.56 | 4 | 42 | 16 | 0 | 0 | 0 |
| 11/2/1997 | 103.64 | 9.44 | 0 | 10 | 0 | 0 | 0 | 0 |
| 11/3/1997 | 81.84 | 9.44 | 0 | 2 | 0 | 1 | 0 | 0 |
| 11/4/1997 | 69.38 | 10.00 | 0 | 8 | 7 | 0 | 0 | 0 |
| 11/5/1997 | 61.73 | 10.00 | 0 | 2 | 1 | 0 | 0 | 0 |

| Date | Discharge (cms) | Temp (C°) | Chinook | Coho | Steelhead | Cutthroat | Lamprey | Sucker |
|---|---|---|---|---|---|---|---|---|
| 11/6/1997 | 62.86 | 10.00 | 2 | 5 | 2 | 0 | 0 | 0 |
| 11/7/1997 | 69.38 | 10.00 | 0 | 39 | 33 | 1 | 0 | 0 |
| 11/8/1997 | 76.17 | 9.44 | 0 | 11 | 10 | 0 | 0 | 0 |
| 11/9/1997 | 70.79 | 8.89 | 1 | 3 | 2 | 0 | 0 | 0 |
| 11/10/1997 | 65.41 | 7.78 | 0 | 0 | 0 | 0 | 0 | 0 |
| 11/11/1997 | 62.30 | 8.33 | 0 | 0 | 0 | 0 | 0 | 0 |
| 11/12/1997 | 58.62 | 8.89 | 0 | 1 | 0 | 0 | 0 | 0 |
| 11/13/1997 | 55.78 | 8.33 | 0 | 6 | 4 | 0 | 0 | 0 |
| 11/14/1997 | 54.09 | 7.78 | 0 | 0 | 0 | 0 | 0 | 0 |
| 11/15/1997 | 52.67 | 7.78 | 0 | 1 | 1 | 0 | 0 | 0 |
| 11/16/1997 | 52.95 | 6.67 | 0 | 0 | 0 | 0 | 0 | 0 |
| 11/17/1997 | 53.80 | 7.22 | 0 | 0 | 0 | 0 | 0 | 0 |
| 11/18/1997 | 55.50 | 7.22 | 0 | 0 | 0 | 0 | 0 | 0 |
| 11/19/1997 | 69.66 | 7.78 | 0 | 3 | 14 | 0 | 0 | 0 |
| 11/20/1997 | 97.98 | 7.78 | 0 | 4 | 4 | 0 | 0 | 0 |
| 11/21/1997 | 105.91 | 7.78 | 0 | 5 | 1 | 0 | 0 | 0 |
| 11/22/1997 | 87.22 | 7.22 | 1 | 9 | 6 | 0 | 0 | 0 |
| 11/23/1997 | 77.59 | 7.78 | 0 | 5 | 8 | 0 | 0 | 0 |
| 11/24/1997 | 77.59 | 8.33 | 0 | 8 | 0 | 0 | 0 | 0 |
| 11/25/1997 | 127.43 | 7.78 | 0 | 2 | 6 | 0 | 0 | 0 |
| 11/26/1997 | 125.16 | 7.22 | 0 | 1 | 1 | 0 | 0 | 0 |
| 11/27/1997 | 108.45 | 7.78 | 0 | 4 | 3 | 0 | 0 | 0 |
| 11/28/1997 | 94.01 | 8.33 | 0 | 4 | 3 | 0 | 0 | 0 |
| 11/29/1997 | 84.67 | 7.78 | 0 | 1 | 7 | 0 | 0 | 0 |
| 11/30/1997 | 90.61 | 7.22 | 0 | 0 | 3 | 0 | 0 | 0 |
| 12/1/1997 | 85.80 | 6.67 | 0 | 0 | 2 | 0 | 0 | 0 |
| 12/2/1997 | 78.15 | 5.56 | 0 | 0 | 2 | 0 | 0 | 0 |
| 12/3/1997 | 69.94 | 4.44 | 0 | 0 | 0 | 0 | 0 | 0 |
| 12/4/1997 | 68.24 | 5.00 | 0 | 0 | 0 | 0 | 0 | 0 |
| 12/5/1997 | 65.41 | 5.56 | 0 | 0 | 0 | 0 | 0 | 0 |
| 12/6/1997 | 61.16 | 6.11 | 0 | 0 | 1 | 0 | 0 | 0 |
| 12/7/1997 | 62.30 | 6.11 | 0 | 0 | 5 | 0 | 0 | 0 |
| 12/8/1997 | 78.15 | 5.56 | 0 | 0 | 0 | 0 | 0 | 0 |
| 12/9/1997 | 77.59 | 5.56 | 0 | 0 | 0 | 0 | 0 | 0 |
| 12/10/1997 | 71.36 | 5.00 | 0 | 0 | 1 | 0 | 0 | 0 |
| 12/11/1997 | 65.98 | 4.44 | 0 | 0 | 0 | 0 | 0 | 0 |
| 12/12/1997 | 62.30 | 3.89 | 0 | 0 | 0 | 0 | 0 | 0 |
| 12/13/1997 | 59.47 | 3.89 | 0 | 0 | 0 | 0 | 0 | 0 |
| 12/14/1997 | 58.90 | 3.89 | 0 | 0 | 0 | 0 | 0 | 0 |
| 12/15/1997 | 63.15 | 4.44 | 0 | 0 | 0 | 0 | 0 | 0 |
| 12/16/1997 | 102.51 | 5.56 | 0 | 0 | 11 | 0 | 0 | 0 |
| 12/17/1997 | 345.47 | 6.67 | 0 | 0 | 1 | 0 | 0 | 0 |
| 12/18/1997 | 235.60 | 6.11 | 0 | 0 | 4 | 0 | 0 | 0 |
| 12/19/1997 | 153.76 | 5.00 | 0 | 0 | 4 | 0 | 0 | 0 |
| 12/20/1997 | 125.16 | 4.44 | 0 | 0 | 1 | 0 | 0 | 0 |

| Date | Discharge (cms) | Temp (C°) | Chinook | Coho | Steelhead | Cutthroat | Lamprey | Sucker |
|---|---|---|---|---|---|---|---|---|
| 12/21/1997 | 112.42 | 5.00 | 0 | 0 | 4 | 0 | 0 | 0 |
| 12/22/1997 | 97.13 | 4.44 | 0 | 0 | 0 | 0 | 0 | 0 |
| 12/23/1997 | 89.20 | 3.89 | 0 | 0 | 0 | 0 | 0 | 0 |
| 12/24/1997 | 91.75 | 4.44 | 0 | 0 | 2 | 0 | 0 | 0 |
| 12/25/1997 | 88.07 | 4.44 | 0 | 0 | 1 | 0 | 0 | 0 |
| 12/26/1997 | 81.84 | 3.33 | 0 | 0 | 0 | 0 | 0 | 0 |
| 12/27/1997 | 76.17 | 3.89 | 0 | 0 | 0 | 0 | 0 | 0 |
| 12/28/1997 | 71.08 | 4.44 | 0 | 0 | 0 | 0 | 0 | 0 |
| 12/29/1997 | 69.66 | 4.44 | 0 | 0 | 1 | 0 | 0 | 0 |
| 12/30/1997 | 73.91 | 4.44 | 0 | 0 | 2 | 0 | 0 | 0 |
| 12/31/1997 | 74.19 | 4.44 | 0 | 0 | 0 | 0 | 0 | 0 |
| 1/1/1998 | 74.19 | 3.89 | 0 | 0 | 0 | 0 | 0 | 0 |
| 1/2/1998 | 112.13 | 5.00 | 0 | 0 | 0 | 0 | 0 | 0 |
| 1/3/1998 | 135.35 | 5.00 | 0 | 0 | 0 | 0 | 0 | 0 |
| 1/4/1998 | 159.99 | 5.00 | 0 | 0 | 0 | 0 | 0 | 0 |
| 1/5/1998 | 135.64 | 5.00 | 0 | 0 | 0 | 0 | 0 | 0 |
| 1/6/1998 | 115.25 | 5.56 | 0 | 0 | 0 | 0 | 0 | 0 |
| 1/7/1998 | 131.96 | 5.56 | 0 | 0 | 0 | 0 | 0 | 0 |
| 1/8/1998 | 193.97 | 6.67 | 0 | 0 | 0 | 0 | 0 | 0 |
| 1/9/1998 | 160.56 | 6.11 | 0 | 0 | 0 | 0 | 0 | 0 |
| 1/10/1998 | 157.72 | 6.67 | 0 | 0 | 0 | 0 | 0 | 0 |
| 1/11/1998 | 413.43 | 7.22 | 0 | 0 | 0 | 0 | 0 | 0 |
| 1/12/1998 | 438.91 | 7.22 | 0 | 0 | 0 | 0 | 0 | 0 |
| 1/13/1998 | 447.41 | 7.22 | 0 | 0 | 0 | 0 | 0 | 0 |
| 1/14/1998 | 339.80 | 7.78 | 0 | 0 | 0 | 0 | 0 | 0 |
| 1/15/1998 | 430.42 | 7.78 | 0 | 0 | 0 | 0 | 0 | 0 |
| 1/16/1998 | 444.57 | 8.33 | 0 | 0 | 0 | 0 | 0 | 0 |
| 1/17/1998 | 688.10 | 8.33 | 0 | 0 | 0 | 0 | 0 | 0 |
| 1/18/1998 | 438.91 | 7.78 | 0 | 0 | 0 | 0 | 0 | 0 |
| 1/19/1998 | 348.30 | 7.22 | 0 | 0 | 0 | 0 | 0 | 0 |
| 1/20/1998 | 283.17 | 7.22 | 0 | 0 | 0 | 0 | 0 | 0 |
| 1/21/1998 | 230.22 | 6.67 | 0 | 0 | 0 | 0 | 0 | 0 |
| 1/22/1998 | 231.07 | 6.67 | 0 | 0 | 0 | 0 | 0 | 0 |
| 1/23/1998 | 269.86 | 7.22 | 0 | 0 | 0 | 0 | 0 | 0 |
| 1/24/1998 | 264.76 | 7.22 | 0 | 0 | 0 | 0 | 0 | 0 |
| 1/25/1998 | 250.32 | 7.22 | 0 | 0 | 0 | 0 | 0 | 0 |
| 1/26/1998 | 247.21 | 7.78 | 0 | 0 | 0 | 0 | 0 | 0 |
| 1/27/1998 | 225.12 | 7.78 | 0 | 0 | 0 | 0 | 0 | 0 |
| 1/28/1998 | 192.55 | 7.78 | 0 | 0 | 0 | 0 | 0 | 0 |
| 1/29/1998 | 183.49 | 7.78 | 0 | 0 | 0 | 0 | 0 | 0 |
| 1/30/1998 | 188.02 | 7.22 | 0 | 0 | 0 | 0 | 0 | 0 |
| 1/31/1998 | 171.60 | 6.67 | 0 | 0 | 0 | 0 | 0 | 0 |
| 2/1/1998 | 152.63 | 6.67 | 0 | 0 | 0 | 0 | 0 | 0 |
| 2/2/1998 | 141.02 | 7.22 | 0 | 0 | 0 | 0 | 0 | 0 |
| 2/3/1998 | 127.99 | 6.67 | 0 | 0 | 0 | 0 | 0 | 0 |

| Date | Discharge (cms) | Temp (C°) | Chinook | Coho | Steelhead | Cutthroat | Lamprey | Sucker |
|---|---|---|---|---|---|---|---|---|
| 2/4/1998 | 127.14 | 7.22 | 0 | 0 | 0 | 0 | 0 | 0 |
| 2/5/1998 | 115.82 | 7.22 | 0 | 0 | 0 | 0 | 0 | 0 |
| 2/6/1998 | 111.29 | 7.78 | 0 | 0 | 0 | 0 | 0 | 0 |
| 2/7/1998 | 100.52 | 7.22 | 0 | 0 | 0 | 0 | 0 | 0 |
| 2/8/1998 | 101.37 | 7.22 | 0 | 0 | 0 | 0 | 0 | 0 |
| 2/9/1998 | 95.99 | 7.22 | 0 | 0 | 0 | 0 | 0 | 0 |
| 2/10/1998 | 91.75 | 6.67 | 0 | 0 | 0 | 0 | 0 | 0 |
| 2/11/1998 | 96.56 | 6.67 | 0 | 0 | 0 | 0 | 0 | 0 |
| 2/12/1998 | 105.34 | 7.22 | 0 | 0 | 0 | 0 | 0 | 0 |
| 2/13/1998 | 118.65 | 7.78 | 0 | 0 | 0 | 0 | 0 | 0 |
| 2/14/1998 | 129.69 | 6.67 | 0 | 0 | 0 | 0 | 0 | 0 |
| 2/15/1998 | 175.85 | 7.22 | 0 | 0 | 0 | 0 | 0 | 0 |
| 2/16/1998 | 163.95 | 6.11 | 0 | 0 | 0 | 0 | 0 | 0 |
| 2/17/1998 | 138.75 | 6.67 | 0 | 0 | 0 | 0 | 0 | 0 |
| 2/18/1998 | 121.48 | 6.67 | 0 | 0 | 0 | 0 | 0 | 0 |
| 2/19/1998 | 127.71 | | 0 | 0 | 0 | 0 | 0 | 0 |
| 2/20/1998 | 135.64 | 6.67 | 0 | 0 | 0 | 0 | 0 | 0 |
| 2/21/1998 | 339.80 | 6.67 | 0 | 0 | 0 | 0 | 0 | 0 |
| 2/22/1998 | 325.64 | 6.67 | 0 | 0 | 0 | 0 | 0 | 0 |
| 2/23/1998 | 232.48 | 6.11 | 0 | 0 | 0 | 0 | 0 | 0 |
| 2/24/1998 | 182.36 | 7.22 | 0 | 0 | 0 | 0 | 0 | 0 |
| 2/25/1998 | 159.14 | 6.67 | 0 | 0 | 0 | 0 | 0 | 0 |
| 2/26/1998 | 143.28 | 6.67 | 0 | 0 | 0 | 0 | 0 | 0 |
| 2/27/1998 | 127.71 | 6.67 | 0 | 0 | 0 | 0 | 0 | 0 |
| 2/28/1998 | 130.82 | 7.22 | 0 | 0 | 0 | 0 | 0 | 0 |
| 3/1/1998 | 196.24 | 7.78 | 0 | 0 | 0 | 0 | 0 | 0 |
| 3/2/1998 | 252.02 | 7.78 | 0 | 0 | 0 | 0 | 0 | 0 |
| 3/3/1998 | 286.00 | 6.67 | 0 | 0 | 0 | 0 | 0 | 0 |
| 3/4/1998 | 251.17 | 6.67 | 0 | 0 | 0 | 0 | 0 | 0 |
| 3/5/1998 | 201.62 | 6.11 | 0 | 0 | 0 | 0 | 0 | 0 |
| 3/6/1998 | 172.17 | 6.67 | 0 | 0 | 0 | 0 | 0 | 0 |
| 3/7/1998 | 148.66 | 5.56 | 0 | 0 | 0 | 0 | 0 | 0 |
| 3/8/1998 | 141.02 | 6.67 | 0 | 0 | 0 | 0 | 0 | 0 |
| 3/9/1998 | 133.94 | 7.22 | 0 | 0 | 0 | 0 | 0 | 0 |
| 3/10/1998 | 127.99 | 6.11 | 0 | 0 | 0 | 0 | 0 | 0 |
| 3/11/1998 | 128.56 | 8.89 | 0 | 0 | 0 | 0 | 0 | 0 |
| 3/12/1998 | 153.19 | 8.33 | 0 | 0 | 0 | 0 | 0 | 0 |
| 3/13/1998 | 176.41 | 9.44 | 0 | 0 | 0 | 0 | 0 | 0 |
| 3/14/1998 | 154.89 | 10.00 | 0 | 0 | 0 | 0 | 0 | 0 |
| 3/15/1998 | 144.70 | 9.44 | 0 | 0 | 0 | 0 | 0 | 0 |
| 3/16/1998 | 145.27 | 10.00 | 0 | 0 | 0 | 0 | 0 | 0 |
| 3/17/1998 | 137.62 | 9.44 | 0 | 0 | 0 | 0 | 0 | 0 |
| 3/18/1998 | 123.18 | 8.89 | 0 | 0 | 0 | 0 | 0 | 0 |
| 3/19/1998 | 109.30 | 9.44 | 0 | 0 | 0 | 0 | 0 | 0 |
| 3/20/1998 | 103.64 | 10.00 | 0 | 0 | 0 | 0 | 0 | 0 |

| Date | Discharge (cms) | Temp (C°) | Chinook | Coho | Steelhead | Cutthroat | Lamprey | Sucker |
|---|---|---|---|---|---|---|---|---|
| 3/21/1998 | 102.22 | 9.44 | 0 | 0 | 0 | 0 | 0 | 0 |
| 3/22/1998 | 154.89 | 10.00 | 0 | 0 | 0 | 0 | 0 | 0 |
| 3/23/1998 | 339.80 | 9.44 | 0 | 0 | 0 | 0 | 0 | 0 |
| 3/24/1998 | 314.32 | 8.33 | 0 | 0 | 0 | 0 | 0 | 0 |
| 3/25/1998 | 245.51 | 9.44 | 0 | 0 | 0 | 0 | 0 | 0 |
| 3/26/1998 | 217.47 | 8.33 | 0 | 0 | 0 | 0 | 0 | 0 |
| 3/27/1998 | 205.01 | 7.22 | 0 | 0 | 0 | 0 | 0 | 0 |
| 3/28/1998 | 186.89 | 7.22 | 0 | 0 | 0 | 0 | 0 | 0 |
| 3/29/1998 | 165.37 | 7.22 | 0 | 0 | 0 | 0 | 0 | 0 |
| 3/30/1998 | 146.40 | 6.11 | 0 | 0 | 0 | 0 | 0 | 0 |
| 3/31/1998 | 143.85 | 7.22 | 0 | 0 | 0 | 0 | 0 | 0 |
| 4/1/1998 | 135.64 | 7.78 | 0 | 0 | 0 | 0 | 0 | 0 |
| 4/2/1998 | 127.14 | 7.78 | 0 | 0 | 0 | 0 | 0 | 0 |
| 4/3/1998 | 121.20 | 8.89 | 0 | 0 | 0 | 0 | 0 | 0 |
| 4/4/1998 | 118.08 | 8.89 | 0 | 0 | 0 | 0 | 0 | 0 |
| 4/5/1998 | 114.97 | 8.89 | 0 | 0 | 0 | 0 | 0 | 0 |
| 4/6/1998 | 116.38 | 9.44 | 0 | 0 | 0 | 0 | 0 | 0 |
| 4/7/1998 | 122.90 | 9.44 | 0 | 0 | 0 | 0 | 0 | 0 |
| 4/8/1998 | 121.48 | 8.89 | 0 | 0 | 0 | 0 | 0 | 0 |
| 4/9/1998 | 118.08 | 8.89 | 0 | 0 | 0 | 0 | 0 | 0 |
| 4/10/1998 | 138.75 | 9.44 | 0 | 0 | 0 | 0 | 0 | 0 |
| 4/11/1998 | 177.83 | 8.89 | 0 | 0 | 0 | 0 | 0 | 0 |
| 4/12/1998 | 173.30 | 7.78 | 0 | 0 | 0 | 0 | 0 | 0 |
| 4/13/1998 | 157.72 | 8.33 | 0 | 0 | 0 | 0 | 0 | 0 |
| 4/14/1998 | 138.47 | 8.89 | 0 | 0 | 0 | 0 | 0 | 0 |
| 4/15/1998 | 127.99 | 8.89 | 0 | 0 | 0 | 0 | 0 | 0 |
| 4/16/1998 | 116.10 | 10.00 | 0 | 0 | 0 | 0 | 0 | 0 |
| 4/17/1998 | 105.91 | 10.56 | 0 | 0 | 0 | 0 | 0 | 0 |
| 4/18/1998 | 97.41 | 11.11 | 0 | 0 | 0 | 0 | 0 | 0 |
| 4/19/1998 | 94.86 | 11.67 | 0 | 0 | 0 | 0 | 0 | 0 |
| 4/20/1998 | 93.45 | 12.78 | 0 | 0 | 0 | 0 | 0 | 0 |
| 4/21/1998 | 92.88 | 12.78 | 0 | 0 | 0 | 0 | 0 | 0 |
| 4/22/1998 | 90.90 | 12.78 | 0 | 0 | 0 | 0 | 0 | 0 |
| 4/23/1998 | 101.94 | 12.22 | 0 | 0 | 0 | 0 | 0 | 0 |
| 4/24/1998 | 151.50 | 10.56 | 0 | 0 | 0 | 0 | 0 | 0 |
| 4/25/1998 | 154.33 | 10.56 | 0 | 0 | 0 | 0 | 0 | 0 |
| 4/26/1998 | 131.96 | 11.11 | 0 | 0 | 0 | 0 | 0 | 0 |
| 4/27/1998 | 121.76 | 12.22 | 0 | 0 | 0 | 0 | 0 | 0 |
| 4/28/1998 | 113.83 | 13.89 | 0 | 0 | 0 | 0 | 0 | 0 |
| 4/29/1998 | 114.12 | 14.44 | 0 | 0 | 0 | 0 | 0 | 0 |
| 4/30/1998 | 116.10 | 14.44 | 0 | 0 | 0 | 0 | 0 | 0 |
| 5/1/1998 | 114.97 | 14.44 | 0 | 0 | 0 | 0 | 0 | 0 |
| 5/2/1998 | 115.25 | 12.78 | 0 | 0 | 0 | 0 | 0 | 0 |
| 5/3/1998 | 115.25 | 13.89 | 0 | 0 | 0 | 0 | 0 | 0 |
| 5/4/1998 | 105.34 | 12.78 | 0 | 0 | 0 | 0 | 0 | 0 |

| Date | Discharge (cms) | Temp (C°) | Chinook | Coho | Steelhead | Cutthroat | Lamprey | Sucker |
|---|---|---|---|---|---|---|---|---|
| 5/5/1998 | 107.04 | 13.89 | 0 | 0 | 0 | 0 | 0 | 0 |
| 5/6/1998 | 106.75 | 13.89 | 0 | 0 | 0 | 0 | 0 | 0 |
| 5/7/1998 | 102.51 | 11.67 | 0 | 0 | 0 | 0 | 0 | 0 |
| 5/8/1998 | 100.24 | 12.22 | 0 | 0 | 0 | 0 | 0 | 0 |
| 5/9/1998 | 104.49 | 11.11 | 0 | 0 | 0 | 0 | 0 | 0 |
| 5/10/1998 | 101.09 | 10.00 | 0 | 0 | 0 | 0 | 0 | 0 |
| 5/11/1998 | 96.84 | 10.00 | 0 | 0 | 0 | 0 | 0 | 0 |
| 5/12/1998 | 103.36 | 10.00 | 0 | 0 | 0 | 0 | 0 | 0 |
| 5/13/1998 | 129.12 | 10.00 | 0 | 0 | 0 | 0 | 0 | 0 |
| 5/14/1998 | 126.01 | 9.44 | 0 | 0 | 0 | 0 | 0 | 0 |
| 5/15/1998 | 168.77 | 9.44 | 0 | 0 | 0 | 0 | 0 | 0 |
| 5/16/1998 | 186.32 | 9.44 | 0 | 0 | 0 | 0 | 0 | 0 |
| 5/17/1998 | 170.47 | 9.44 | 0 | 0 | 0 | 0 | 0 | 0 |
| 5/18/1998 | 159.42 | 11.11 | 0 | 0 | 0 | 0 | 0 | 0 |
| 5/19/1998 | 151.50 | 11.11 | 0 | 0 | 0 | 0 | 0 | 0 |
| 5/20/1998 | 244.66 | 10.56 | 0 | 0 | 0 | 0 | 0 | 0 |
| 5/21/1998 | 351.13 | 10.00 | 0 | 0 | 0 | 0 | 0 | 0 |
| 5/22/1998 | 242.11 | 11.67 | 0 | 0 | 0 | 0 | 0 | 0 |
| 5/23/1998 | 186.32 | 11.67 | 0 | 0 | 0 | 0 | 0 | 0 |
| 5/24/1998 | 164.52 | 10.56 | 0 | 0 | 0 | 0 | 0 | 0 |
| 5/25/1998 | 249.75 | 10.56 | 0 | 0 | 0 | 0 | 0 | 0 |
| 5/26/1998 | 243.52 | 10.00 | 0 | 0 | 0 | 0 | 0 | 0 |
| 5/27/1998 | 209.26 | 11.11 | 0 | 0 | 0 | 0 | 0 | 0 |
| 5/28/1998 | 178.40 | 10.00 | 0 | 0 | 0 | 0 | 0 | 0 |
| 5/29/1998 | 291.66 | 10.56 | 0 | 0 | 0 | 0 | 0 | 0 |
| 5/30/1998 | 402.10 | 11.11 | 0 | 0 | 0 | 0 | 0 | 0 |
| 5/31/1998 | 297.33 | 12.22 | 0 | 0 | 0 | 0 | 0 | 0 |
| 6/1/1998 | 222.00 | 13.89 | 0 | 0 | 0 | 0 | 0 | 0 |
| 6/2/1998 | 179.81 | 14.44 | 0 | 0 | 0 | 0 | 0 | 0 |
| 6/3/1998 | 154.89 | 14.44 | 0 | 0 | 0 | 0 | 0 | 0 |
| 6/4/1998 | 140.73 | 12.78 | 0 | 0 | 0 | 0 | 0 | 0 |
| 6/5/1998 | 127.71 | 15.56 | 0 | 0 | 0 | 0 | 0 | 0 |
| 6/6/1998 | 117.23 | 16.67 | 0 | 0 | 0 | 0 | 0 | 0 |
| 6/7/1998 | 109.02 | 16.11 | 0 | 0 | 0 | 0 | 0 | 0 |
| 6/8/1998 | 107.60 | 15.00 | 0 | 0 | 0 | 0 | 0 | 0 |
| 6/9/1998 | 104.49 | 16.11 | 0 | 0 | 0 | 0 | 0 | 0 |
| 6/10/1998 | 101.94 | 15.00 | 0 | 0 | 0 | 0 | 0 | 0 |
| 6/11/1998 | 107.60 | 15.56 | 0 | 0 | 0 | 0 | 0 | 0 |
| 6/12/1998 | 103.92 | 16.67 | 0 | 0 | 0 | 0 | 0 | 0 |
| 6/13/1998 | 96.28 | 16.67 | 0 | 0 | 0 | 0 | 0 | 0 |
| 6/14/1998 | 89.48 | 17.22 | 0 | 0 | 0 | 0 | 0 | 0 |
| 6/15/1998 | 82.40 | 16.67 | 0 | 0 | 0 | 0 | 0 | 0 |
| 6/16/1998 | 79.00 | 16.11 | 0 | 0 | 0 | 0 | 0 | 0 |
| 6/17/1998 | 77.30 | 16.67 | 0 | 0 | 0 | 0 | 0 | 0 |
| 6/18/1998 | 74.19 | 15.56 | 0 | 0 | 0 | 0 | 0 | 0 |

| Date | Discharge (cms) | Temp (C°) | Chinook | Coho | Steelhead | Cutthroat | Lamprey | Sucker |
|---|---|---|---|---|---|---|---|---|
| 6/19/1998 | 69.94 | 17.22 | 0 | 0 | 0 | 0 | 0 | 0 |
| 6/20/1998 | 66.83 | 17.78 | 0 | 0 | 0 | 0 | 0 | 0 |
| 6/21/1998 | 70.23 | 18.89 | 0 | 0 | 0 | 0 | 0 | 0 |
| 6/22/1998 | 67.11 | 17.78 | 0 | 0 | 0 | 0 | 0 | 0 |
| 6/23/1998 | 66.54 | 17.78 | 0 | 0 | 0 | 0 | 0 | 0 |
| 6/24/1998 | 67.11 | 17.22 | 0 | 0 | 0 | 0 | 0 | 0 |
| 6/25/1998 | 69.38 | 15.56 | 0 | 0 | 0 | 0 | 0 | 0 |
| 6/26/1998 | 72.21 | 15.56 | 0 | 0 | 0 | 0 | 0 | 0 |
| 6/27/1998 | 71.36 | 16.11 | 0 | 0 | 0 | 0 | 0 | 0 |
| 6/28/1998 | 65.70 | 17.78 | 0 | 0 | 0 | 0 | 0 | 0 |
| 6/29/1998 | 61.73 | 17.22 | 0 | 0 | 0 | 0 | 0 | 0 |
| 6/30/1998 | 60.31 | 17.22 | 0 | 0 | 0 | 0 | 0 | 0 |
| 7/1/1998 | 58.62 | 16.11 | 0 | 0 | 0 | 0 | 0 | 0 |
| 7/2/1998 | 57.20 | 16.67 | 0 | 0 | 0 | 0 | 0 | 0 |
| 7/3/1998 | 54.93 | 17.22 | 0 | 0 | 0 | 0 | 0 | 0 |
| 7/4/1998 | 54.65 | 17.78 | 0 | 0 | 0 | 0 | 0 | 0 |
| 7/5/1998 | 53.80 | 19.44 | 0 | 0 | 0 | 0 | 0 | 0 |
| 7/6/1998 | 51.25 | 21.11 | 0 | 0 | 0 | 0 | 0 | 0 |
| 7/7/1998 | 47.57 | 20.00 | 0 | 0 | 0 | 0 | 0 | 0 |
| 7/8/1998 | 47.01 | 20.56 | 0 | 0 | 0 | 0 | 0 | 0 |
| 7/9/1998 | 46.44 | 21.11 | 0 | 0 | 0 | 0 | 0 | 0 |
| 7/10/1998 | 49.55 | 20.56 | 0 | 0 | 0 | 0 | 0 | 0 |
| 7/11/1998 | 48.42 | 18.33 | 0 | 0 | 0 | 0 | 0 | 0 |
| 7/12/1998 | 46.72 | 18.89 | 0 | 0 | 0 | 0 | 0 | 0 |
| 7/13/1998 | 43.89 | 21.11 | 0 | 0 | 0 | 0 | 0 | 0 |
| 7/14/1998 | 43.89 | 21.11 | 0 | 0 | 0 | 0 | 0 | 0 |
| 7/15/1998 | 39.93 | 22.22 | 0 | 0 | 0 | 0 | 0 | 0 |
| 7/16/1998 | 39.93 | 22.22 | 0 | 0 | 0 | 0 | 0 | 0 |
| 7/17/1998 | 42.19 | 22.22 | 0 | 0 | 0 | 0 | 0 | 0 |
| 7/18/1998 | 42.76 | 22.22 | 0 | 0 | 0 | 0 | 0 | 0 |
| 7/19/1998 | 41.34 | 21.67 | 0 | 0 | 0 | 0 | 0 | 0 |
| 7/20/1998 | 38.23 | 21.67 | 0 | 0 | 0 | 0 | 0 | 0 |
| 7/21/1998 | 38.79 | 22.78 | 0 | 0 | 0 | 0 | 0 | 0 |
| 7/22/1998 | 39.64 | 22.78 | 0 | 0 | 0 | 0 | 0 | 0 |
| 7/23/1998 | 38.51 | 22.78 | 0 | 0 | 0 | 0 | 0 | 0 |
| 7/24/1998 | 36.81 | 22.22 | 0 | 0 | 0 | 0 | 0 | 0 |
| 7/25/1998 | 37.66 | 22.78 | 0 | 0 | 0 | 0 | 0 | 0 |
| 7/26/1998 | 38.51 | 23.89 | 0 | 0 | 0 | 0 | 0 | 0 |
| 7/27/1998 | 38.23 | 23.89 | 0 | 0 | 0 | 0 | 0 | 0 |
| 7/28/1998 | 34.55 | 24.44 | 0 | 0 | 0 | 0 | 0 | 0 |
| 7/29/1998 | 32.85 | 22.78 | 0 | 0 | 0 | 0 | 0 | 0 |
| 7/30/1998 | 31.71 | 21.67 | 0 | 0 | 0 | 0 | 0 | 0 |
| 7/31/1998 | 33.70 | 21.11 | 0 | 0 | 0 | 0 | 0 | 0 |
| 8/1/1998 | 33.98 | 21.11 | 0 | 0 | 0 | 0 | 0 | 0 |
| 8/2/1998 | 31.43 | 22.78 | 0 | 0 | 0 | 0 | 0 | 0 |

| Date | Discharge (cms) | Temp (C°) | Chinook | Coho | Steelhead | Cutthroat | Lamprey | Sucker |
|---|---|---|---|---|---|---|---|---|
| 8/3/1998 | 33.41 | 22.78 | 0 | 0 | 0 | 0 | 0 | 0 |
| 8/4/1998 | 30.58 | 23.33 | 0 | 0 | 0 | 0 | 0 | 0 |
| 8/5/1998 | 31.15 | 23.33 | 0 | 0 | 0 | 0 | 0 | 0 |
| 8/6/1998 | 35.96 | 21.67 | 0 | 0 | 0 | 0 | 0 | 0 |
| 8/7/1998 | 32.00 | 20.56 | 0 | 0 | 0 | 0 | 0 | 0 |
| 8/8/1998 | 29.45 | 21.11 | 0 | 0 | 0 | 0 | 0 | 0 |
| 8/9/1998 | 30.58 | 21.11 | 0 | 0 | 0 | 0 | 0 | 0 |
| 8/10/1998 | 30.30 | 21.67 | 0 | 0 | 0 | 0 | 0 | 0 |
| 8/11/1998 | 30.30 | 22.22 | 0 | 0 | 0 | 0 | 0 | 0 |
| 8/12/1998 | 30.02 | 22.22 | 0 | 0 | 0 | 0 | 0 | 0 |
| 8/13/1998 | 29.17 | 22.78 | 0 | 0 | 0 | 0 | 0 | 0 |
| 8/14/1998 | 29.45 | 23.33 | 0 | 0 | 0 | 0 | 0 | 0 |
| 8/15/1998 | 28.88 | 22.22 | 0 | 0 | 0 | 0 | 0 | 0 |
| 8/16/1998 | 29.17 | 20.00 | 0 | 0 | 0 | 0 | 0 | 0 |
| 8/17/1998 | 28.60 | 20.00 | 0 | 0 | 0 | 0 | 0 | 0 |
| 8/18/1998 | 27.89 | 20.00 | 0 | 0 | 0 | 0 | 0 | 0 |
| 8/19/1998 | 27.81 | 20.00 | 0 | 0 | 0 | 0 | 0 | 0 |
| 8/20/1998 | 29.17 | 19.44 | 0 | 0 | 0 | 0 | 0 | 0 |
| 8/21/1998 | 28.60 | 19.44 | 0 | 0 | 0 | 0 | 0 | 0 |
| 8/22/1998 | 28.88 | 19.44 | 0 | 0 | 0 | 0 | 0 | 0 |
| 8/23/1998 | 28.88 | 18.89 | 0 | 0 | 0 | 0 | 0 | 0 |
| 8/24/1998 | 28.88 | 18.89 | 0 | 0 | 0 | 0 | 0 | 0 |
| 8/25/1998 | 29.17 | 19.44 | 0 | 0 | 0 | 0 | 0 | 0 |
| 8/26/1998 | 28.60 | 20.00 | 0 | 0 | 0 | 0 | 0 | 0 |
| 8/27/1998 | 28.32 | 20.00 | 0 | 0 | 0 | 0 | 0 | 0 |
| 8/28/1998 | 27.75 | 20.00 | 0 | 0 | 0 | 0 | 0 | 0 |
| 8/29/1998 | 26.62 | 20.00 | 0 | 0 | 0 | 0 | 0 | 0 |
| 8/30/1998 | 25.77 | 20.56 | 0 | 0 | 0 | 0 | 0 | 0 |
| 8/31/1998 | 24.64 | 21.11 | 0 | 0 | 0 | 0 | 0 | 0 |
| 9/1/1998 | 24.07 | 21.11 | 0 | 0 | 0 | 0 | 0 | 0 |
| 9/2/1998 | 23.79 | 21.11 | 0 | 0 | 0 | 0 | 0 | 0 |
| 9/3/1998 | 23.22 | 21.11 | 0 | 0 | 0 | 0 | 0 | 0 |
| 9/4/1998 | 22.94 | 21.11 | 0 | 0 | 0 | 0 | 0 | 0 |
| 9/5/1998 | 22.37 | 20.56 | 0 | 0 | 0 | 0 | 0 | 0 |
| 9/6/1998 | 22.37 | 20.00 | 0 | 0 | 0 | 0 | 0 | 0 |
| 9/7/1998 | 22.65 | 19.44 | 0 | 0 | 0 | 0 | 0 | 0 |
| 9/8/1998 | 22.65 | 18.33 | 0 | 0 | 0 | 0 | 0 | 0 |
| 9/9/1998 | 22.37 | 17.22 | 0 | 0 | 0 | 0 | 0 | 0 |
| 9/10/1998 | 22.09 | 16.67 | 0 | 0 | 0 | 0 | 0 | 0 |
| 9/11/1998 | 22.37 | 17.22 | 0 | 0 | 0 | 0 | 0 | 0 |
| 9/12/1998 | 22.65 | 17.22 | 0 | 0 | 0 | 0 | 0 | 0 |
| 9/13/1998 | 23.50 | 17.78 | 0 | 0 | 0 | 0 | 0 | 0 |
| 9/14/1998 | 23.50 | 17.78 | 0 | 0 | 0 | 0 | 0 | 0 |
| 9/15/1998 | 23.22 | 18.33 | 0 | 0 | 0 | 0 | 0 | 0 |
| 9/16/1998 | 22.94 | 18.33 | 0 | 0 | 0 | 0 | 0 | 0 |

| Date | Discharge (cms) | Temp (C°) | Chinook | Coho | Steelhead | Cutthroat | Lamprey | Sucker |
|---|---|---|---|---|---|---|---|---|
| 9/17/1998 | 22.65 | 17.22 | 0 | 0 | 0 | 0 | 0 | 0 |
| 9/18/1998 | 22.65 | 16.67 | 0 | 0 | 0 | 0 | 0 | 0 |
| 9/19/1998 | 22.37 | 16.11 | 0 | 0 | 0 | 0 | 0 | 0 |
| 9/20/1998 | 22.65 | 15.56 | 0 | 0 | 0 | 0 | 0 | 0 |
| 9/21/1998 | 24.07 | 15.56 | 0 | 0 | 0 | 0 | 0 | 0 |
| 9/22/1998 | 24.92 | 15.56 | 0 | 0 | 0 | 0 | 0 | 0 |
| 9/23/1998 | 25.99 | 15.56 | 0 | 0 | 0 | 0 | 0 | 0 |
| 9/24/1998 | 26.11 | 15.00 | 0 | 0 | 0 | 0 | 0 | 0 |
| 9/25/1998 | 25.97 | 14.44 | 0 | 0 | 0 | 0 | 0 | 0 |
| 9/26/1998 | 26.59 | 15.00 | 0 | 0 | 0 | 0 | 0 | 0 |
| 9/27/1998 | 27.69 | 15.00 | 0 | 0 | 0 | 0 | 0 | 0 |
| 9/28/1998 | 27.16 | 15.56 | 0 | 0 | 0 | 0 | 0 | 0 |
| 9/29/1998 | 27.86 | 14.44 | 0 | 0 | 0 | 0 | 0 | 0 |
| 9/30/1998 | 28.01 | 13.89 | 0 | 0 | 0 | 0 | 0 | 0 |
| 10/1/1998 | 28.60 | 13.89 | 0 | 0 | 0 | 0 | 0 | 0 |
| 10/2/1998 | 30.02 | 13.33 | 0 | 0 | 0 | 0 | 0 | 0 |
| 10/3/1998 | 32.56 | 13.33 | 0 | 0 | 0 | 0 | 0 | 0 |
| 10/4/1998 | 33.13 | 13.33 | 0 | 0 | 0 | 0 | 0 | 0 |
| 10/5/1998 | 32.85 | 13.33 | 0 | 0 | 0 | 0 | 0 | 0 |
| 10/6/1998 | 30.02 | 12.78 | 0 | 0 | 0 | 0 | 0 | 0 |
| 10/7/1998 | 27.75 | 12.78 | 0 | 0 | 0 | 0 | 0 | 0 |
| 10/8/1998 | 29.17 | 12.78 | 0 | 0 | 0 | 0 | 0 | 0 |
| 10/9/1998 | 30.02 | 12.22 | 0 | 0 | 0 | 0 | 0 | 0 |
| 10/10/1998 | 31.71 | 12.22 | 0 | 0 | 0 | 0 | 0 | 0 |
| 10/11/1998 | 32.85 | 12.22 | 0 | 0 | 0 | 0 | 0 | 0 |
| 10/12/1998 | 30.87 | 12.22 | 0 | 0 | 0 | 0 | 0 | 0 |
| 10/13/1998 | 31.43 | 12.22 | 0 | 0 | 0 | 0 | 0 | 0 |
| 10/14/1998 | 37.38 | 11.67 | 0 | 0 | 0 | 0 | 0 | 0 |
| 10/15/1998 | 36.53 | 11.11 | 0 | 0 | 0 | 0 | 0 | 0 |
| 10/16/1998 | 36.25 | 11.11 | 0 | 0 | 0 | 0 | 0 | 0 |
| 10/17/1998 | 36.81 | 10.56 | 0 | 0 | 0 | 0 | 0 | 0 |
| 10/18/1998 | 33.70 | 10.56 | 0 | 0 | 0 | 0 | 0 | 0 |
| 10/19/1998 | 30.87 | 10.56 | 0 | 0 | 0 | 0 | 0 | 0 |
| 10/20/1998 | 30.30 | 10.56 | 0 | 0 | 0 | 0 | 0 | 0 |
| 10/21/1998 | 31.15 | 10.00 | 0 | 0 | 0 | 0 | 0 | 0 |
| 10/22/1998 | 30.58 | 10.00 | 0 | 0 | 0 | 0 | 0 | 0 |
| 10/23/1998 | 28.32 | 10.00 | 0 | 0 | 0 | 0 | 0 | 0 |
| 10/24/1998 | 28.32 | 10.56 | 0 | 0 | 0 | 0 | 0 | 0 |
| 10/25/1998 | 30.87 | 11.11 | 0 | 0 | 0 | 0 | 0 | 0 |
| 10/26/1998 | 32.56 | 10.56 | 0 | 0 | 0 | 0 | 0 | 0 |
| 10/27/1998 | 32.56 | 10.56 | 0 | 0 | 0 | 0 | 0 | 0 |
| 10/28/1998 | 33.41 | 10.56 | 0 | 0 | 0 | 0 | 0 | 0 |
| 10/29/1998 | 34.26 | 10.00 | 0 | 0 | 0 | 0 | 0 | 0 |
| 10/30/1998 | 32.00 | 8.89 | 0 | 0 | 0 | 0 | 0 | 0 |
| 10/31/1998 | 31.43 | 7.78 | 0 | 0 | 0 | 0 | 0 | 0 |

| Date | Discharge (cms) | Temp (C°) | Chinook | Coho | Steelhead | Cutthroat | Lamprey | Sucker |
|---|---|---|---|---|---|---|---|---|
| 11/1/1998 | 32.85 | 8.50 | 1 | 1 | 0 | 0 | 0 | 0 |
| 11/2/1998 | 37.10 | 8.61 | 0 | 2 | 0 | 0 | 0 | 0 |
| 11/3/1998 | 35.68 | 8.39 | 1 | 6 | 0 | 0 | 0 | 0 |
| 11/4/1998 | 36.25 | 9.28 | 0 | 0 | 0 | 0 | 0 | 0 |
| 11/5/1998 | 52.67 | 8.99 | 2 | 103 | 35 | 0 | 0 | 0 |
| 11/6/1998 | 69.66 | 8.78 | 0 | 331 | 25 | 3 | 0 | 0 |
| 11/7/1998 | 62.86 | 8.24 | 0 | 226 | 18 | 0 | 0 | 0 |
| 11/8/1998 | 86.37 | 7.70 | 0 | 166 | 13 | 0 | 0 | 0 |
| 11/9/1998 | 74.76 | 7.78 | 0 | 47 | 1 | 0 | 0 | 0 |
| 11/10/1998 | 59.75 | 7.61 | 0 | 100 | 14 | 0 | 0 | 0 |
| 11/11/1998 | 79.00 | 6.89 | 0 | 3 | 1 | 0 | 0 | 0 |
| 11/12/1998 | 65.13 | 6.72 | 0 | 19 | 3 | 0 | 0 | 0 |
| 11/13/1998 | 53.24 | 7.28 | 0 | 19 | 1 | 0 | 0 | 0 |
| 11/14/1998 | 48.70 | 7.33 | 0 | 44 | 2 | 1 | 0 | 0 |
| 11/15/1998 | 47.86 | 8.11 | 0 | 44 | 11 | 0 | 0 | 0 |
| 11/16/1998 | 67.11 | 8.03 | 0 | 29 | 5 | 0 | 0 | 0 |
| 11/17/1998 | 82.12 | 8.37 | 0 | 272 | 41 | 2 | 0 | 0 |
| 11/18/1998 | 97.41 | 8.45 | 0 | 17 | 1 | 0 | 0 | 0 |
| 11/19/1998 | 96.28 | 7.83 | 0 | 79 | 23 | 0 | 0 | 0 |
| 11/20/1998 | 83.82 | 7.84 | 0 | 40 | 3 | 1 | 0 | 0 |
| 11/21/1998 | 1138.34 | 9.18 | 0 | 2 | 2 | 0 | 0 | 0 |
| 11/22/1998 | 835.35 | 9.02 | 0 | 0 | 0 | 0 | 0 | 0 |
| 11/23/1998 | 455.90 | 8.77 | 0 | 0 | 0 | 0 | 0 | 0 |
| 11/24/1998 | 535.19 | 8.50 | 0 | 0 | 0 | 0 | 0 | 0 |
| 11/25/1998 | 351.13 | 8.70 | 0 | 0 | 0 | 0 | 0 | 0 |
| 11/26/1998 | 379.45 | 9.14 | 0 | 0 | 0 | 0 | 0 | 0 |
| 11/27/1998 | 339.80 | 8.71 | 0 | 0 | 0 | 0 | 0 | 0 |
| 11/28/1998 | 219.74 | 8.00 | 0 | 6 | 6 | 0 | 0 | 0 |
| 11/29/1998 | 180.38 | 7.16 | 0 | 1 | 2 | 0 | 0 | 0 |
| 11/30/1998 | 253.44 | 7.78 | 0 | 0 | 0 | 0 | 0 | 0 |
| 12/1/1998 | 351.13 | 8.11 | 0 | 0 | 0 | 0 | 0 | 0 |
| 12/2/1998 | 722.08 | 8.45 | 0 | 0 | 0 | 0 | 0 | 0 |
| 12/3/1998 | 750.40 | 8.36 | 0 | 0 | 0 | 0 | 0 | 0 |
| 12/4/1998 | 362.46 | 7.06 | 0 | 0 | 1 | 0 | 0 | 0 |
| 12/5/1998 | 244.94 | 6.22 | 0 | 2 | 1 | 0 | 0 | 0 |
| 12/6/1998 | 198.50 | 5.11 | 0 | 0 | 1 | 0 | 0 | 0 |
| 12/7/1998 | 161.97 | 5.43 | 0 | 2 | 2 | 0 | 0 | 0 |
| 12/8/1998 | 200.77 | 5.89 | 0 | 1 | 13 | 1 | 0 | 0 |
| 12/9/1998 | 178.96 | 5.65 | 0 | 2 | 3 | 0 | 0 | 0 |
| 12/10/1998 | 148.38 | 5.56 | 0 | 5 | 8 | 0 | 0 | 0 |
| 12/11/1998 | 159.99 | 6.61 | 0 | 4 | 45 | 0 | 0 | 0 |
| 12/12/1998 | 217.19 | 7.72 | 0 | 9 | 39 | 0 | 0 | 0 |
| 12/13/1998 | 246.64 | 7.78 | 0 | 3 | 60 | 0 | 0 | 0 |
| 12/14/1998 | 215.21 | 7.15 | 0 | 0 | 1 | 0 | 0 | 0 |
| 12/15/1998 | 172.17 | 6.22 | 0 | 0 | 9 | 0 | 0 | 0 |

| Date | Discharge (cms) | Temp (C°) | Chinook | Coho | Steelhead | Cutthroat | Lamprey | Sucker |
|---|---|---|---|---|---|---|---|---|
| 12/16/1998 | 146.68 | 5.50 | 0 | 0 | 0 | 0 | 0 | 0 |
| 12/17/1998 | 131.11 | 5.44 | 0 | 0 | 1 | 1 | 0 | 0 |
| 12/18/1998 | 122.33 | 5.42 | 0 | 0 | 1 | 0 | 0 | 0 |
| 12/19/1998 | 115.82 | 4.78 | 0 | 0 | 1 | 0 | 0 | 0 |
| 12/20/1998 | 104.21 | 3.14 | 0 | 0 | 0 | 0 | 0 | 0 |
| 12/21/1998 | 89.20 | 1.14 | 0 | 0 | 0 | 0 | 0 | 0 |
| 12/22/1998 | 82.40 | 0.58 | 0 | 0 | 0 | 0 | 0 | 0 |
| 12/23/1998 | 82.12 | 0.53 | 0 | 0 | 0 | 0 | 0 | 0 |
| 12/24/1998 | 77.87 | 0.60 | 0 | 0 | 0 | 0 | 0 | 0 |
| 12/25/1998 | 92.60 | 1.98 | 0 | 0 | 0 | 0 | 0 | 0 |
| 12/26/1998 | 152.63 | 3.59 | 0 | 0 | 0 | 0 | 0 | 0 |
| 12/27/1998 | 182.08 | 5.89 | 0 | 0 | 10 | 0 | 0 | 0 |
| 12/28/1998 | 1053.39 | 6.72 | 0 | 0 | 1 | 0 | 0 | 0 |
| 12/29/1998 | 770.22 | 7.72 | 0 | 0 | 0 | 0 | 0 | 0 |
| 12/30/1998 | 402.10 | 7.89 | 0 | 0 | 0 | 0 | 0 | 0 |
| 12/31/1998 | 322.81 | 7.72 | 0 | 0 | 18 | 0 | 0 | 0 |
| 1/1/1999 | 311.49 | 6.87 | 0 | 0 | 51 | 0 | 0 | 0 |
| 1/2/1999 | 234.46 | 6.18 | 0 | 0 | 4 | 0 | 0 | 0 |
| 1/3/1999 | 186.32 | 5.47 | 0 | 0 | 0 | 0 | 0 | 0 |
| 1/4/1999 | 155.74 | 4.73 | 0 | 0 | 1 | 1 | 0 | 0 |
| 1/5/1999 | 134.79 | 4.89 | 0 | 0 | 7 | 0 | 0 | 0 |
| 1/6/1999 | 118.08 | 4.99 | 0 | 0 | 13 | 0 | 0 | 0 |
| 1/7/1999 | 111.00 | 5.44 | 0 | 0 | 24 | 0 | 0 | 0 |
| 1/8/1999 | 101.09 | 5.03 | 0 | 0 | 3 | 0 | 0 | 0 |
| 1/9/1999 | 93.73 | 4.48 | 0 | 0 | 1 | 0 | 0 | 0 |
| 1/10/1999 | 89.20 | 3.98 | 0 | 0 | 0 | 0 | 0 | 0 |
| 1/11/1999 | 82.40 | 3.98 | 0 | 0 | 0 | 0 | 0 | 0 |
| 1/12/1999 | 85.23 | 5.32 | 0 | 0 | 10 | 0 | 0 | 0 |
| 1/13/1999 | 86.08 | 6.20 | 0 | 0 | 63 | 1 | 0 | 0 |
| 1/14/1999 | 90.61 | 6.19 | 0 | 0 | 86 | 1 | 0 | 0 |
| 1/15/1999 | 427.58 | 7.24 | 0 | 0 | 4 | 0 | 0 | 0 |
| 1/16/1999 | 441.74 | 7.26 | 0 | 0 | 49 | 0 | 0 | 0 |
| 1/17/1999 | 393.60 | 7.00 | 0 | 0 | 6 | 0 | 0 | 0 |
| 1/18/1999 | 639.96 | 7.42 | 0 | 0 | 0 | 0 | 0 | 0 |
| 1/19/1999 | 421.92 | 7.51 | 0 | 0 | 14 | 0 | 0 | 0 |
| 1/20/1999 | 348.30 | 7.28 | 0 | 0 | 0 | 0 | 0 | 0 |
| 1/21/1999 | 532.36 | 6.95 | 0 | 0 | 1 | 0 | 0 | 0 |
| 1/22/1999 | 484.22 | 6.73 | 0 | 0 | 8 | 0 | 0 | 0 |
| 1/23/1999 | 535.19 | 6.71 | 0 | 0 | 0 | 0 | 0 | 0 |
| 1/24/1999 | 334.14 | 6.01 | 0 | 0 | 0 | 0 | 0 | 0 |
| 1/25/1999 | 238.71 | 5.51 | 0 | 0 | 3 | 0 | 0 | 0 |
| 1/26/1999 | 194.54 | 5.10 | 0 | 0 | 0 | 0 | 0 | 0 |
| 1/27/1999 | 161.12 | 5.32 | 0 | 0 | 0 | 0 | 0 | 0 |
| 1/28/1999 | 141.30 | 5.64 | 0 | 0 | 9 | 1 | 0 | 0 |
| 1/29/1999 | 127.99 | 5.92 | 0 | 0 | 14 | 0 | 0 | 0 |

| Date | Discharge (cms) | Temp (C°) | Chinook | Coho | Steelhead | Cutthroat | Lamprey | Sucker |
|---|---|---|---|---|---|---|---|---|
| 1/30/1999 | 120.06 | 5.98 | 0 | 0 | 57 | 0 | 0 | 0 |
| 1/31/1999 | 114.97 | 5.63 | 0 | 0 | 5 | 0 | 0 | 0 |
| 2/1/1999 | 107.89 | 5.17 | 0 | 0 | 1 | 0 | 0 | 0 |
| 2/2/1999 | 111.29 | 4.96 | 0 | 0 | 3 | 0 | 0 | 0 |
| 2/3/1999 | 124.59 | 4.97 | 0 | 0 | 2 | 0 | 0 | 0 |
| 2/4/1999 | 138.19 | 5.85 | 0 | 0 | 37 | 1 | 0 | 0 |
| 2/5/1999 | 140.73 | 5.22 | 0 | 0 | 1 | 0 | 0 | 0 |
| 2/6/1999 | 216.34 | 5.46 | 0 | 0 | 17 | 0 | 0 | 0 |
| 2/7/1999 | 509.70 | 6.34 | 0 | 0 | 4 | 0 | 0 | 0 |
| 2/8/1999 | 382.28 | 5.85 | 0 | 0 | 0 | 0 | 0 | 0 |
| 2/9/1999 | 250.32 | 5.33 | 0 | 0 | 8 | 0 | 0 | 0 |
| 2/10/1999 | 193.69 | 4.79 | 0 | 0 | 0 | 0 | 0 | 0 |
| 2/11/1999 | 156.31 | 4.75 | 0 | 0 | 1 | 0 | 0 | 0 |
| 2/12/1999 | 138.47 | 5.33 | 0 | 0 | 14 | 0 | 0 | 0 |
| 2/13/1999 | 134.51 | 5.72 | 0 | 0 | 55 | 0 | 0 | 0 |
| 2/14/1999 | 152.63 | 6.52 | 0 | 0 | 147 | 2 | 0 | 0 |
| 2/15/1999 | 159.99 | 6.18 | 0 | 0 | 53 | 0 | 0 | 0 |
| 2/16/1999 | 167.07 | 6.42 | 0 | 0 | 132 | 0 | 0 | 0 |
| 2/17/1999 | 351.13 | 6.72 | 0 | 0 | 66 | 0 | 0 | 0 |
| 2/18/1999 | 461.56 | 6.75 | 0 | 0 | 2 | 0 | 0 | 0 |
| 2/19/1999 | 515.37 | 7.13 | 0 | 0 | 33 | 0 | 0 | 0 |
| 2/20/1999 | 314.32 | 6.13 | 0 | 0 | 16 | 0 | 0 | 0 |
| 2/21/1999 | 231.07 | 6.74 | 0 | 0 | 107 | 0 | 0 | 0 |
| 2/22/1999 | 189.16 | 6.67 | 0 | 0 | 114 | 1 | 0 | 0 |
| 2/23/1999 | 275.81 | 7.17 | 0 | 0 | 147 | 0 | 0 | 0 |
| 2/24/1999 | 328.48 | 7.44 | 0 | 0 | 366 | 0 | 0 | 0 |
| 2/25/1999 | 305.82 | 7.03 | 0 | 0 | 41 | 0 | 0 | 0 |
| 2/26/1999 | 259.10 | 6.12 | 0 | 0 | 4 | 0 | 0 | 0 |
| 2/27/1999 | 302.99 | 6.72 | 0 | 0 | 17 | 0 | 0 | 0 |
| 2/28/1999 | 696.59 | 7.42 | 0 | 0 | 0 | 0 | 0 | 0 |
| 3/1/1999 | 577.66 | 7.05 | 0 | 0 | 2 | 0 | 0 | 0 |
| 3/2/1999 | 359.62 | 6.73 | 0 | 0 | 9 | 0 | 0 | 0 |
| 3/3/1999 | 322.81 | 6.84 | 0 | 0 | 45 | 0 | 0 | 0 |
| 3/4/1999 | 288.83 | 6.00 | 0 | 0 | 0 | 0 | 0 | 0 |
| 3/5/1999 | 233.61 | 5.43 | 0 | 0 | 10 | 0 | 0 | 0 |
| 3/6/1999 | 197.09 | 5.14 | 0 | 0 | 4 | 0 | 0 | 0 |
| 3/7/1999 | 169.33 | | 0 | 0 | 20 | 0 | 0 | 0 |
| 3/8/1999 | 151.50 | | 0 | 0 | 53 | 0 | 0 | 0 |
| 3/9/1999 | 146.68 | 6.65 | 0 | 0 | 49 | 0 | 0 | 0 |
| 3/10/1999 | 130.26 | 6.38 | 0 | 0 | 35 | 0 | 0 | 0 |
| 3/11/1999 | 120.91 | 6.45 | 0 | 0 | 34 | 0 | 0 | 0 |
| 3/12/1999 | 112.42 | 5.83 | 0 | 0 | 26 | 0 | 0 | 0 |
| 3/13/1999 | 137.34 | 6.63 | 0 | 0 | 67 | 0 | 0 | 0 |
| 3/14/1999 | 173.30 | 6.86 | 0 | 0 | 266 | 0 | 0 | 0 |
| 3/15/1999 | 161.97 | 7.15 | 0 | 0 | 41 | 0 | 0 | 0 |

| Date | Discharge (cms) | Temp (C°) | Chinook | Coho | Steelhead | Cutthroat | Lamprey | Sucker |
|---|---|---|---|---|---|---|---|---|
| 3/16/1999 | 148.38 | 8.16 | 0 | 0 | 248 | 1 | 0 | 0 |
| 3/17/1999 | 138.19 | 7.63 | 0 | 0 | 92 | 0 | 0 | 0 |
| 3/18/1999 | 131.11 | 7.65 | 0 | 0 | 140 | 1 | 0 | 0 |
| 3/19/1999 | 131.39 | 8.07 | 0 | 0 | 82 | 3 | 0 | 1 |
| 3/20/1999 | 151.78 | 7.76 | 0 | 0 | 56 | 1 | 0 | 0 |
| 3/21/1999 | 157.72 | 8.63 | 0 | 0 | 155 | 4 | 0 | 0 |
| 3/22/1999 | 146.40 | 8.04 | 0 | 0 | 30 | 1 | 0 | 0 |
| 3/23/1999 | 145.83 | 8.34 | 0 | 0 | 52 | 0 | 0 | 0 |
| 3/24/1999 | 140.17 | 7.82 | 0 | 0 | 59 | 0 | 0 | 0 |
| 3/25/1999 | 148.66 | 7.72 | 0 | 0 | 66 | 0 | 0 | 0 |
| 3/26/1999 | 146.96 | 7.94 | 0 | 0 | 30 | 0 | 0 | 0 |
| 3/27/1999 | 146.96 | 7.31 | 0 | 0 | 6 | 0 | 0 | 0 |
| 3/28/1999 | 134.79 | 6.67 | 0 | 0 | 2 | 0 | 0 | 0 |
| 3/29/1999 | 135.92 | 7.33 | 0 | 0 | 8 | 0 | 0 | 0 |
| 3/30/1999 | 157.44 | 6.31 | 0 | 0 | 3 | 0 | 0 | 0 |
| 3/31/1999 | 147.25 | 6.13 | 0 | 0 | 3 | 0 | 0 | 0 |
| 4/1/1999 | 139.04 | 6.14 | 1 | 0 | 3 | 0 | 0 | 0 |
| 4/2/1999 | 133.66 | 7.57 | 0 | 0 | 24 | 0 | 0 | 0 |
| 4/3/1999 | 133.37 | 7.12 | 0 | 0 | 15 | 0 | 0 | 0 |
| 4/4/1999 | 128.28 | 6.31 | 1 | 0 | 0 | 0 | 0 | 0 |
| 4/5/1999 | 122.61 | 6.30 | 0 | 0 | 0 | 0 | 0 | 0 |
| 4/6/1999 | 124.03 | 8.01 | 0 | 0 | 27 | 0 | 0 | 0 |
| 4/7/1999 | 123.74 | 7.93 | 1 | 0 | 142 | 1 | 0 | 1 |
| 4/8/1999 | 137.90 | 7.05 | 0 | 0 | 14 | 0 | 0 | 0 |
| 4/9/1999 | 141.58 | 6.79 | 0 | 0 | 20 | 0 | 0 | 0 |
| 4/10/1999 | 130.82 | 6.80 | 1 | 0 | 3 | 0 | 0 | 0 |
| 4/11/1999 | 130.82 | 8.89 | 1 | 0 | 63 | 0 | 0 | 2 |
| 4/12/1999 | 144.70 | 8.80 | 4 | 0 | 183 | 0 | 0 | 10 |
| 4/13/1999 | 167.64 | 9.25 | 6 | 0 | 275 | 0 | 0 | 5 |
| 4/14/1999 | 167.64 | 9.62 | 3 | 0 | 117 | 1 | 0 | 10 |
| 4/15/1999 | 157.72 | 10.25 | 0 | 0 | 66 | 0 | 0 | 27 |
| 4/16/1999 | 171.32 | 10.23 | 1 | 0 | 114 | 0 | 0 | 156 |
| 4/17/1999 | 188.02 | 10.40 | 10 | 0 | 133 | 0 | 0 | 139 |
| 4/18/1999 | 205.01 | 9.97 | 10 | 0 | 79 | 0 | 0 | 102 |
| 4/19/1999 | 199.63 | 9.48 | 1 | 0 | 17 | 0 | 0 | 3 |
| 4/20/1999 | 183.21 | 9.14 | 0 | 0 | 8 | 0 | 0 | 0 |
| 4/21/1999 | 166.22 | 8.97 | 4 | 0 | 16 | 0 | 0 | 0 |
| 4/22/1999 | 150.93 | 8.54 | 1 | 0 | 6 | 0 | 0 | 0 |
| 4/23/1999 | 132.52 | 9.92 | 2 | 0 | 13 | 0 | 0 | 0 |
| 4/24/1999 | 131.11 | 10.72 | 5 | 0 | 27 | 0 | 0 | 85 |
| 4/25/1999 | 146.68 | 10.11 | 2 | 0 | 12 | 0 | 0 | 13 |
| 4/26/1999 | 149.80 | 9.57 | 1 | 0 | 9 | 0 | 0 | 0 |
| 4/27/1999 | 137.05 | 8.56 | 0 | 0 | 4 | 0 | 0 | 0 |
| 4/28/1999 | 122.90 | 8.01 | 1 | 0 | 3 | 0 | 0 | 0 |
| 4/29/1999 | 109.30 | 9.38 | 8 | 0 | 10 | 0 | 0 | 0 |

| Date | Discharge (cms) | Temp (C°) | Chinook | Coho | Steelhead | Cutthroat | Lamprey | Sucker |
|---|---|---|---|---|---|---|---|---|
| 4/30/1999 | 101.94 | 10.96 | 95 | 0 | 40 | 0 | 0 | 11 |
| 5/1/1999 | 104.21 | 10.91 | 104 | 0 | 50 | 0 | 0 | 150 |
| 5/2/1999 | 129.41 | 9.64 | 1 | 0 | 0 | 0 | 0 | 13 |
| 5/3/1999 | 193.40 | 8.17 | 3 | 0 | 18 | 0 | 0 | 0 |
| 5/4/1999 | 234.46 | 7.55 | 0 | 0 | 0 | 0 | 0 | 0 |
| 5/5/1999 | 194.25 | 8.19 | 0 | 0 | 0 | 0 | 0 | 0 |
| 5/6/1999 | 178.11 | 9.53 | 9 | 0 | 0 | 0 | 0 | 0 |
| 5/7/1999 | 183.49 | 8.91 | 1 | 0 | 2 | 0 | 0 | 0 |
| 5/8/1999 | 163.39 | 8.08 | 0 | 0 | 0 | 0 | 0 | 0 |
| 5/9/1999 | 141.02 | 8.07 | 0 | 0 | 0 | 0 | 0 | 0 |
| 5/10/1999 | 122.61 | 8.17 | 0 | 0 | 1 | 0 | 0 | 0 |
| 5/11/1999 | 117.23 | 7.94 | 0 | 0 | 0 | 0 | 0 | 0 |
| 5/12/1999 | 132.52 | 8.42 | 1 | 0 | 3 | 0 | 0 | 0 |
| 5/13/1999 | 147.25 | 10.10 | 80 | 0 | 8 | 0 | 0 | 0 |
| 5/14/1999 | 143.85 | 9.18 | 5 | 0 | 0 | 0 | 0 | 0 |
| 5/15/1999 | 145.27 | 9.68 | 3 | 0 | 4 | 0 | 0 | 0 |
| 5/16/1999 | 137.34 | 10.13 | 6 | 0 | 11 | 0 | 0 | 0 |
| 5/17/1999 | 132.52 | 10.29 | 86 | 0 | 6 | 0 | 0 | 10 |
| 5/18/1999 | 179.53 | 10.44 | 122 | 0 | 22 | 0 | 0 | 133 |
| 5/19/1999 | 186.32 | 9.98 | 3 | 0 | 0 | 0 | 1 | 149 |
| 5/20/1999 | 173.30 | 10.41 | 0 | 0 | 0 | 0 | 0 | 0 |
| 5/21/1999 | 156.59 | 10.36 | 16 | 0 | 1 | 0 | 0 | 0 |
| 5/22/1999 | 145.27 | 12.43 | 2 | 0 | 1 | 0 | 0 | 153 |
| 5/23/1999 | 152.63 | 12.68 | 2 | 0 | 2 | 0 | 0 | 1129 |
| 5/24/1999 | 175.00 | 12.42 | 0 | 0 | 0 | 0 | 0 | 0 |
| 5/25/1999 | 196.52 | 12.18 | 110 | 0 | 4 | 0 | 0 | 102 |
| 5/26/1999 | 182.36 | 11.24 | 1 | 0 | 0 | 0 | 0 | 0 |
| 5/27/1999 | 172.17 | 12.47 | 47 | 0 | 0 | 0 | 0 | 0 |
| 5/28/1999 | 173.87 | 12.92 | 65 | 0 | 3 | 0 | 0 | 0 |
| 5/29/1999 | 168.20 | 12.09 | 4 | 0 | 0 | 1 | 1 | 0 |
| 5/30/1999 | 156.88 | 11.89 | 3 | 0 | 0 | 0 | 0 | 0 |
| 5/31/1999 | 143.85 | 12.35 | 2 | 0 | 0 | 0 | 0 | 0 |
| 6/1/1999 | 142.72 | 12.67 | 118 | 0 | 4 | 0 | 0 | 1 |
| 6/2/1999 | 143.00 | 11.78 | 93 | 0 | 2 | 0 | 0 | 0 |
| 6/3/1999 | 127.99 | 10.69 | 3 | 0 | 0 | 0 | 1 | 0 |
| 6/4/1999 | 112.98 | 12.19 | 81 | 0 | 5 | 0 | 0 | 0 |
| 6/5/1999 | 105.34 | 11.92 | 324 | 0 | 10 | 0 | 0 | 4 |
| 6/6/1999 | 106.19 | 12.22 | 120 | 0 | 9 | 0 | 0 | 0 |
| 6/7/1999 | 101.94 | 11.66 | 124 | 0 | 5 | 0 | 0 | 0 |
| 6/8/1999 | 95.71 | 11.82 | 118 | 0 | 6 | 0 | 0 | 0 |
| 6/9/1999 | 89.20 | 13.03 | 171 | 0 | 11 | 0 | 0 | 2 |
| 6/10/1999 | 86.65 | 13.36 | 229 | 0 | 12 | 0 | 0 | 37 |
| 6/11/1999 | 85.52 | 13.90 | 234 | 0 | 13 | 0 | 1 | 87 |
| 6/12/1999 | 89.20 | 14.72 | 61 | 0 | 12 | 1 | 0 | 394 |
| 6/13/1999 | 97.13 | 14.92 | 39 | 0 | 5 | 0 | 0 | 678 |

| Date | Discharge (cms) | Temp (C°) | Chinook | Coho | Steelhead | Cutthroat | Lamprey | Sucker |
|---|---|---|---|---|---|---|---|---|
| 6/14/1999 | 107.04 | 15.51 | 29 | 0 | 7 | 0 | 5 | 281 |
| 6/15/1999 | 118.36 | 15.11 | 69 | 0 | 15 | 0 | 1 | 986 |
| 6/16/1999 | 117.51 | 14.03 | 37 | 0 | 3 | 0 | 0 | 9 |
| 6/17/1999 | 116.67 | 14.08 | 16 | 0 | 18 | 0 | 0 | 18 |
| 6/18/1999 | 112.13 | 15.11 | 18 | 0 | 29 | 0 | 0 | 21 |
| 6/19/1999 | 106.47 | 14.51 | 97 | 0 | 57 | 0 | 0 | 59 |
| 6/20/1999 | 105.06 | 13.95 | 17 | 0 | 19 | 0 | 0 | 1 |
| 6/21/1999 | 101.09 | 13.60 | 5 | 0 | 2 | 0 | 0 | 5 |
| 6/22/1999 | 97.98 | 15.19 | 45 | 0 | 21 | 0 | 0 | 0 |
| 6/23/1999 | 94.86 | 15.63 | 84 | 0 | 43 | 0 | 1 | 0 |
| 6/24/1999 | 91.75 | 15.43 | 187 | 0 | 63 | 0 | 3 | 0 |
| 6/25/1999 | 95.71 | 13.93 | 25 | 0 | 9 | 0 | 0 | 0 |
| 6/26/1999 | 91.46 | 14.62 | 37 | 0 | 25 | 0 | 0 | 0 |
| 6/27/1999 | 85.52 | 14.40 | 18 | 0 | 8 | 0 | 1 | 0 |
| 6/28/1999 | 80.99 | 15.24 | 108 | 0 | 62 | 0 | 0 | 0 |
| 6/29/1999 | 78.15 | 15.99 | 62 | 0 | 29 | 0 | 0 | 3 |
| 6/30/1999 | 77.87 | 16.49 | 65 | 0 | 34 | 0 | 2 | 0 |
| 7/1/1999 | 75.32 | 16.70 | 104 | 0 | 43 | 0 | 0 | 0 |
| 7/2/1999 | 75.61 | 16.26 | 77 | 0 | 36 | 0 | 0 | 0 |
| 7/3/1999 | 70.79 | 14.87 | 27 | 0 | 13 | 0 | 0 | 0 |
| 7/4/1999 | 69.38 | 14.52 | 21 | 0 | 10 | 0 | 0 | 0 |
| 7/5/1999 | 64.85 | 14.64 | 15 | 0 | 22 | 1 | 0 | 0 |
| 7/6/1999 | 62.58 | 15.70 | 19 | 0 | 65 | 0 | 0 | 0 |
| 7/7/1999 | 62.86 | 14.95 | 43 | 0 | 34 | 0 | 0 | 0 |
| 7/8/1999 | 62.01 | 16.89 | 74 | 0 | 80 | 1 | 1 | 10 |
| 7/9/1999 | 60.88 | 17.13 | 75 | 0 | 51 | 0 | 0 | 8 |
| 7/10/1999 | 56.63 | 17.82 | 82 | 0 | 45 | 0 | 1 | 11 |
| 7/11/1999 | 55.78 | 18.63 | 85 | 0 | 60 | 0 | 1 | 3 |
| 7/12/1999 | 57.77 | 19.32 | 65 | 0 | 44 | 0 | 4 | 9 |
| 7/13/1999 | 58.05 | 19.66 | 64 | 0 | 54 | 0 | 0 | 10 |
| 7/14/1999 | 55.22 | 18.99 | 39 | 0 | 51 | 2 | 0 | 1 |
| 7/15/1999 | 54.09 | 18.35 | 22 | 0 | 64 | 2 | 0 | 1 |
| 7/16/1999 | 51.54 | 17.76 | 18 | 0 | 44 | 3 | 0 | 0 |
| 7/17/1999 | 49.27 | 17.28 | 7 | 0 | 16 | 2 | 0 | 0 |
| 7/18/1999 | 47.86 | 17.54 | 3 | 0 | 9 | 1 | 1 | 0 |
| 7/19/1999 | 47.01 | 18.26 | 24 | 0 | 33 | 1 | 0 | 0 |
| 7/20/1999 | 45.02 | 18.68 | 31 | 0 | 34 | 3 | 0 | 1 |
| 7/21/1999 | 47.57 | 18.69 | 13 | 0 | 9 | 3 | 0 | 0 |
| 7/22/1999 | 50.40 | 18.61 | 10 | 0 | 23 | 2 | 0 | 0 |
| 7/23/1999 | 44.46 | 18.72 | 16 | 0 | 32 | 0 | 0 | 0 |
| 7/24/1999 | 44.46 | 18.80 | 12 | 0 | 63 | 0 | 0 | 0 |
| 7/25/1999 | 43.61 | 18.41 | 29 | 0 | 40 | 1 | 1 | 50 |
| 7/26/1999 | 43.32 | 19.18 | 1 | 0 | 0 | 0 | 0 | 18 |
| 7/27/1999 | 43.04 | 20.07 | 7 | 0 | 52 | 0 | 0 | 1 |
| 7/28/1999 | 41.91 | 19.39 | 8 | 0 | 33 | 0 | 0 | 8 |

| Date | Discharge (cms) | Temp (C°) | Chinook | Coho | Steelhead | Cutthroat | Lamprey | Sucker |
|---|---|---|---|---|---|---|---|---|
| 7/29/1999 | 40.78 | 20.03 | 6 | 0 | 40 | 2 | 0 | 11 |
| 7/30/1999 | 38.23 | 19.31 | 12 | 0 | 33 | 4 | 0 | 0 |
| 7/31/1999 | 36.81 | 19.41 | 14 | 0 | 42 | 2 | 0 | 1 |
| 8/1/1999 | 38.79 | 19.14 | 9 | 0 | 41 | 3 | 0 | 0 |
| 8/2/1999 | 39.08 | 18.90 | 4 | 0 | 13 | 2 | 0 | 0 |
| 8/3/1999 | 38.51 | 19.64 | 7 | 0 | 8 | 0 | 0 | 0 |
| 8/4/1999 | 37.38 | 19.47 | 6 | 0 | 38 | 2 | 0 | 1 |
| 8/5/1999 | 36.25 | 19.45 | 0 | 0 | 0 | 0 | 0 | 0 |
| 8/6/1999 | 38.23 | 18.15 | 4 | 0 | 57 | 3 | 0 | 0 |
| 8/7/1999 | 41.91 | 17.32 | 12 | 0 | 44 | 0 | 0 | 25 |
| 8/8/1999 | 44.46 | 16.20 | 4 | 0 | 24 | 0 | 0 | 0 |
| 8/9/1999 | 41.34 | 17.71 | 0 | 0 | 0 | 0 | 0 | 0 |
| 8/10/1999 | 37.10 | 19.10 | 5 | 0 | 45 | 0 | 0 | 1 |
| 8/11/1999 | 36.81 | 19.47 | 6 | 0 | 24 | 2 | 0 | 0 |
| 8/12/1999 | 40.78 | 17.95 | 3 | 0 | 6 | 3 | 0 | 0 |
| 8/13/1999 | 39.64 | 18.03 | 4 | 0 | 22 | 1 | 0 | 0 |
| 8/14/1999 | 38.23 | 17.11 | 0 | 0 | 9 | 2 | 0 | 0 |
| 8/15/1999 | 35.96 | 16.94 | 1 | 0 | 1 | 2 | 0 | 0 |
| 8/16/1999 | 35.40 | 17.47 | 0 | 0 | 4 | 0 | 0 | 0 |
| 8/17/1999 | 37.10 | 18.14 | 12 | 0 | 36 | 0 | 0 | 0 |
| 8/18/1999 | 35.68 | 18.58 | 13 | 0 | 17 | 2 | 0 | 0 |
| 8/19/1999 | 33.98 | 18.14 | 5 | 0 | 15 | 1 | 0 | 0 |
| 8/20/1999 | 33.41 | 19.31 | 7 | 0 | 18 | 4 | 0 | 0 |
| 8/21/1999 | 34.55 | 18.96 | 7 | 0 | 14 | 6 | 0 | 0 |
| 8/22/1999 | 34.83 | 19.13 | 2 | 0 | 1 | 0 | 0 | 0 |
| 8/23/1999 | 30.87 | 19.51 | 7 | 0 | 2 | 0 | 0 | 0 |
| 8/24/1999 | 31.71 | 18.84 | 5 | 0 | 38 | 1 | 0 | 0 |
| 8/25/1999 | 27.84 | 19.70 | 8 | 0 | 11 | 1 | 0 | 0 |
| 8/26/1999 | 29.73 | 20.26 | 9 | 0 | 15 | 2 | 0 | 0 |
| 8/27/1999 | 29.17 | 20.16 | 6 | 0 | 10 | 2 | 0 | 0 |
| 8/28/1999 | 31.43 | 20.04 | 8 | 0 | 4 | 2 | 0 | 1 |
| 8/29/1999 | 31.15 | 19.47 | 3 | 0 | 5 | 0 | 0 | 0 |
| 8/30/1999 | 36.53 | | 0 | 0 | 0 | 0 | 0 | 0 |
| 8/31/1999 | 36.25 | | 0 | 0 | 0 | 0 | 0 | 0 |
| 9/1/1999 | 35.40 | | 0 | 0 | 0 | 0 | 0 | 0 |
| 9/2/1999 | 32.00 | | 0 | 0 | 0 | 0 | 0 | 0 |
| 9/3/1999 | 33.70 | | 0 | 0 | 0 | 0 | 0 | 0 |
| 9/4/1999 | 33.13 | | 0 | 0 | 0 | 0 | 0 | 0 |
| 9/5/1999 | 32.56 | | 0 | 0 | 0 | 0 | 0 | 0 |
| 9/6/1999 | 32.00 | | 0 | 0 | 0 | 0 | 0 | 0 |
| 9/7/1999 | 32.00 | | 0 | 0 | 0 | 0 | 0 | 0 |
| 9/8/1999 | 28.88 | | 0 | 0 | 0 | 0 | 0 | 0 |
| 9/9/1999 | 28.60 | | 0 | 0 | 0 | 0 | 0 | 0 |
| 9/10/1999 | 23.76 | | 0 | 0 | 0 | 0 | 0 | 0 |
| 9/11/1999 | 28.88 | 15.77 | 23 | 0 | 16 | 4 | 0 | 12 |

| Date | Discharge (cms) | Temp (C°) | Chinook | Coho | Steelhead | Cutthroat | Lamprey | Sucker |
|---|---|---|---|---|---|---|---|---|
| 9/12/1999 | 30.87 | 16.27 | 2 | 0 | 7 | 1 | 0 | 5 |
| 9/13/1999 | 28.88 | 16.27 | 3 | 0 | 7 | 1 | 0 | 1 |
| 9/14/1999 | 27.55 | 16.51 | 0 | 0 | 1 | 0 | 0 | 0 |
| 9/15/1999 | 28.01 | 16.99 | 0 | 0 | 0 | 0 | 0 | 0 |
| 9/16/1999 | 30.87 | 16.03 | 2 | 1 | 10 | 0 | 0 | 0 |
| 9/17/1999 | 32.00 | 15.19 | 0 | 0 | 1 | 0 | 0 | 0 |
| 9/18/1999 | 30.58 | 15.56 | 1 | 1 | 6 | 0 | 0 | 0 |
| 9/19/1999 | 27.98 | 15.86 | 4 | 1 | 3 | 0 | 0 | 0 |
| 9/20/1999 | 28.18 | 16.08 | 2 | 1 | 5 | 0 | 0 | 0 |
| 9/21/1999 | 29.73 | 16.26 | 0 | 2 | 0 | 0 | 0 | 0 |
| 9/22/1999 | 29.45 | 16.55 | 1 | 0 | 4 | 0 | 0 | 0 |
| 9/23/1999 | 29.45 | 16.19 | 0 | 0 | 0 | 0 | 0 | 0 |
| 9/24/1999 | 29.45 | 16.01 | 0 | 10 | 6 | 2 | 0 | 0 |
| 9/25/1999 | 28.60 | 16.10 | 4 | 10 | 13 | 0 | 0 | 0 |
| 9/26/1999 | 27.64 | 15.36 | 0 | 2 | 2 | 0 | 0 | 0 |
| 9/27/1999 | 28.32 | 13.57 | 1 | 0 | 0 | 1 | 0 | 0 |
| 9/28/1999 | 28.88 | 12.92 | 0 | 0 | 0 | 0 | 0 | 0 |
| 9/29/1999 | 28.18 | 12.61 | 0 | 0 | 0 | 0 | 0 | 0 |
| 9/30/1999 | 28.01 | 12.88 | 0 | 0 | 0 | 0 | 0 | 0 |
| 10/1/1999 | 26.53 | 12.69 | 1 | 4 | 0 | 0 | 0 | 0 |
| 10/2/1999 | 26.22 | 12.70 | 0 | 0 | 0 | 0 | 0 | 0 |
| 10/3/1999 | 27.92 | 12.62 | 0 | 0 | 0 | 0 | 0 | 0 |
| 10/4/1999 | 27.75 | 12.66 | 2 | 2 | 0 | 0 | 0 | 0 |
| 10/5/1999 | 27.27 | 12.45 | 0 | 0 | 0 | 0 | 0 | 0 |
| 10/6/1999 | 27.81 | 12.57 | 3 | 17 | 0 | 0 | 0 | 0 |
| 10/7/1999 | 29.73 | 12.49 | 0 | 0 | 0 | 1 | 0 | 0 |
| 10/8/1999 | 29.73 | 13.14 | 0 | 0 | 0 | 0 | 0 | 0 |
| 10/9/1999 | 28.88 | 13.60 | 0 | 0 | 0 | 0 | 0 | 0 |
| 10/10/1999 | 28.60 | 12.86 | 0 | 0 | 0 | 0 | 0 | 0 |
| 10/11/1999 | 28.60 | 12.70 | 1 | 43 | 17 | 0 | 0 | 0 |
| 10/12/1999 | 27.58 | 12.34 | 0 | 0 | 0 | 0 | 0 | 0 |
| 10/13/1999 | 26.39 | 12.00 | 1 | 15 | 0 | 0 | 0 | 0 |
| 10/14/1999 | 26.76 | 12.49 | 0 | 0 | 0 | 0 | 0 | 0 |
| 10/15/1999 | 26.56 | 11.23 | 2 | 25 | 5 | 0 | 0 | 0 |
| 10/16/1999 | 27.27 | 10.38 | 1 | 0 | 2 | 0 | 0 | 0 |
| 10/17/1999 | 27.78 | 9.92 | 0 | 3 | 0 | 0 | 0 | 0 |
| 10/18/1999 | 27.67 | 9.50 | 0 | 0 | 0 | 0 | 0 | 0 |
| 10/19/1999 | 27.44 | 9.39 | 0 | 1 | 1 | 0 | 0 | 0 |
| 10/20/1999 | 27.47 | 9.60 | 1 | 4 | 0 | 0 | 0 | 0 |
| 10/21/1999 | 27.41 | 9.99 | 1 | 12 | 3 | 0 | 0 | 0 |
| 10/22/1999 | 25.71 | 10.07 | 2 | 44 | 11 | 0 | 0 | 0 |
| 10/23/1999 | 27.69 | 9.98 | 2 | 9 | 3 | 0 | 0 | 0 |
| 10/24/1999 | 28.60 | 10.23 | 0 | 3 | 0 | 0 | 0 | 0 |
| 10/25/1999 | 28.60 | 10.42 | 0 | 0 | 0 | 0 | 0 | 0 |
| 10/26/1999 | 31.43 | 10.33 | 0 | 0 | 0 | 0 | 0 | 0 |

| Date | Discharge (cms) | Temp (C°) | Chinook | Coho | Steelhead | Cutthroat | Lamprey | Sucker |
|---|---|---|---|---|---|---|---|---|
| 10/27/1999 | 37.38 | 9.28 | 2 | 75 | 0 | 0 | 0 | 0 |
| 10/28/1999 | 45.87 | 9.33 | 1 | 97 | 12 | 0 | 0 | 0 |
| 10/29/1999 | 88.91 | 9.22 | 1 | 113 | 10 | 0 | 0 | 0 |
| 10/30/1999 | 50.40 | 9.24 | 0 | 58 | 4 | 0 | 0 | 0 |
| 10/31/1999 | 38.23 | 9.63 | 0 | 0 | 0 | 0 | 0 | 0 |
| 11/1/1999 | 38.23 | 8.42 | 0 | 34 | 2 | 0 | 0 | 0 |
| 11/2/1999 | 36.25 | 7.94 | 0 | 25 | 7 | 0 | 0 | 0 |
| 11/3/1999 | 33.70 | 7.68 | 0 | 11 | 2 | 0 | 0 | 0 |
| 11/4/1999 | 32.56 | 8.18 | 0 | 39 | 3 | 0 | 0 | 0 |
| 11/5/1999 | 33.41 | 8.37 | 0 | 17 | 1 | 0 | 0 | 0 |
| 11/6/1999 | 35.40 | 8.74 | 0 | 81 | 4 | 0 | 0 | 0 |
| 11/7/1999 | 38.79 | 9.67 | 0 | 0 | 0 | 0 | 0 | 0 |
| 11/8/1999 | 35.68 | 9.66 | 0 | 54 | 15 | 0 | 0 | 0 |
| 11/9/1999 | 35.96 | 9.71 | 1 | 589 | 16 | 0 | 0 | 0 |
| 11/10/1999 | 37.66 | 9.40 | 0 | 480 | 20 | 0 | 0 | 0 |
| 11/11/1999 | 53.24 | 9.71 | 4 | 251 | 37 | 3 | 0 | 0 |
| 11/12/1999 | 45.02 | 10.55 | 1 | 88 | 5 | 0 | 0 | 0 |
| 11/13/1999 | 38.51 | 10.80 | 0 | 30 | 2 | 1 | 0 | 0 |
| 11/14/1999 | 35.68 | 10.41 | 0 | 17 | 1 | 1 | 0 | 0 |
| 11/15/1999 | 34.83 | 10.33 | 0 | 11 | 6 | 0 | 0 | 0 |
| 11/16/1999 | 35.40 | 10.16 | 0 | 22 | 6 | 0 | 0 | 0 |
| 11/17/1999 | 41.63 | 9.10 | 0 | 23 | 3 | 0 | 0 | 0 |
| 11/18/1999 | 44.17 | 8.50 | 1 | 12 | 2 | 0 | 0 | 0 |
| 11/19/1999 | 40.49 | 8.06 | 0 | 4 | 0 | 0 | 0 | 0 |
| 11/20/1999 | 47.57 | 8.11 | 0 | 0 | 0 | 0 | 0 | 0 |
| 11/21/1999 | 73.34 | 8.00 | 0 | 0 | 0 | 0 | 0 | 0 |
| 11/22/1999 | 70.23 | 7.34 | 0 | 0 | 0 | 0 | 0 | 0 |
| 11/23/1999 | 62.58 | 7.03 | 0 | 4 | 6 | 0 | 0 | 0 |
| 11/24/1999 | 69.09 | 6.94 | 0 | 29 | 4 | 0 | 0 | 0 |
| 11/25/1999 | 200.20 | 7.73 | 0 | 1 | 0 | 0 | 0 | 0 |
| 11/26/1999 | 365.29 | 9.15 | 0 | 0 | 1 | 0 | 0 | 0 |
| 11/27/1999 | 243.52 | 9.18 | 1 | 20 | 16 | 0 | 0 | 0 |
| 11/28/1999 | 135.35 | 8.08 | 0 | 2 | 1 | 0 | 0 | 0 |
| 11/29/1999 | 96.56 | 7.41 | 0 | 0 | 0 | 0 | 0 | 0 |
| 11/30/1999 | 88.35 | 7.32 | 0 | 6 | 10 | 0 | 0 | 0 |
| 12/1/1999 | 93.45 | 7.28 | 0 | 0 | 0 | 0 | 0 | 0 |
| 12/2/1999 | 126.86 | 7.35 | 0 | 0 | 2 | 1 | 0 | 0 |
| 12/3/1999 | 183.78 | 6.95 | 0 | 2 | 3 | 0 | 0 | 0 |
| 12/4/1999 | 122.90 | 6.19 | 0 | 0 | 3 | 0 | 0 | 0 |
| 12/5/1999 | 105.91 | 6.17 | 0 | 2 | 4 | 0 | 0 | 0 |
| 12/6/1999 | 107.32 | 6.79 | 0 | 2 | 5 | 0 | 0 | 0 |
| 12/7/1999 | 209.54 | 6.92 | 0 | 0 | 2 | 0 | 0 | 0 |
| 12/8/1999 | 183.49 | 6.33 | 0 | 0 | 0 | 0 | 0 | 0 |
| 12/9/1999 | 141.87 | 6.26 | 0 | 0 | 8 | 0 | 0 | 0 |
| 12/10/1999 | 142.43 | 6.13 | 0 | 0 | 6 | 0 | 0 | 0 |

| Date | Discharge (cms) | Temp (C°) | Chinook | Coho | Steelhead | Cutthroat | Lamprey | Sucker |
|---|---|---|---|---|---|---|---|---|
| 12/11/1999 | 119.50 | 6.41 | 0 | 0 | 2 | 0 | 0 | 0 |
| 12/12/1999 | 137.62 | 6.61 | 0 | 1 | 4 | 0 | 0 | 0 |
| 12/13/1999 | 223.99 | 6.54 | 0 | 0 | 4 | 0 | 0 | 0 |
| 12/14/1999 | 175.56 | 6.21 | 0 | 0 | 2 | 0 | 0 | 0 |
| 12/15/1999 | 145.83 | 6.22 | 0 | 0 | 0 | 0 | 0 | 0 |
| 12/16/1999 | 174.43 | 6.85 | 0 | 0 | 0 | 0 | 0 | 0 |
| 12/17/1999 | 264.48 | 7.47 | 0 | 0 | 0 | 0 | 0 | 0 |
| 12/18/1999 | 322.81 | 7.81 | 0 | 0 | 7 | 0 | 0 | 0 |
| 12/19/1999 | 233.90 | 7.66 | 0 | 0 | 5 | 0 | 0 | 0 |
| 12/20/1999 | 173.30 | 7.12 | 0 | 0 | 7 | 0 | 0 | 0 |
| 12/21/1999 | 144.70 | 6.83 | 0 | 0 | 7 | 0 | 0 | 0 |
| 12/22/1999 | 125.44 | 5.84 | 0 | 0 | 2 | 0 | 0 | 0 |
| 12/23/1999 | 109.87 | 4.90 | 0 | 0 | 0 | 0 | 0 | 0 |
| 12/24/1999 | 101.09 | 4.79 | 0 | 0 | 2 | 0 | 0 | 0 |
| 12/25/1999 | 96.84 | 4.66 | 0 | 0 | 1 | 0 | 0 | 0 |
| 12/26/1999 | 90.61 | 4.43 | 0 | 0 | 0 | 0 | 0 | 0 |
| 12/27/1999 | 84.10 | 4.22 | 0 | 0 | 1 | 0 | 0 | 0 |
| 12/28/1999 | 79.85 | 4.08 | 0 | 0 | 0 | 0 | 0 | 0 |
| 12/29/1999 | 73.06 | 3.90 | 0 | 0 | 0 | 0 | 0 | 0 |
| 12/30/1999 | 68.24 | 3.76 | 0 | 0 | 0 | 0 | 0 | 0 |
| 12/31/1999 | 60.03 | 3.82 | 0 | 0 | 0 | 0 | 0 | 0 |
| 1/1/2000 | 64.00 | 4.24 | 0 | 0 | 2 | 0 | 0 | 0 |
| 1/2/2000 | 77.02 | 4.65 | 0 | 0 | 2 | 0 | 0 | 0 |
| 1/3/2000 | 94.01 | 4.88 | 0 | 0 | 9 | 0 | 0 | 0 |
| 1/4/2000 | 106.47 | 5.30 | 0 | 0 | 10 | 0 | 0 | 0 |
| 1/5/2000 | 180.94 | 5.78 | 0 | 0 | 29 | 0 | 0 | 0 |
| 1/6/2000 | 134.51 | 5.26 | 0 | 0 | 15 | 0 | 0 | 0 |
| 1/7/2000 | 110.72 | 5.70 | 0 | 0 | 32 | 0 | 0 | 0 |
| 1/8/2000 | 120.91 | 6.10 | 0 | 0 | 22 | 0 | 0 | 0 |
| 1/9/2000 | 193.69 | 6.53 | 0 | 0 | 71 | 0 | 0 | 0 |
| 1/10/2000 | 438.91 | 6.46 | 0 | 0 | 5 | 0 | 0 | 0 |
| 1/11/2000 | 996.75 | 7.05 | 0 | 0 | 0 | 0 | 0 | 0 |
| 1/12/2000 | 464.40 | 6.16 | 0 | 0 | 0 | 0 | 0 | 0 |
| 1/13/2000 | 404.93 | 6.06 | 0 | 0 | 0 | 0 | 0 | 0 |
| 1/14/2000 | 775.88 | 6.65 | 0 | 0 | 0 | 0 | 0 | 0 |
| 1/15/2000 | 600.32 | 6.69 | 0 | 0 | 0 | 0 | 0 | 0 |
| 1/16/2000 | 438.91 | 6.73 | 0 | 0 | 1 | 0 | 0 | 0 |
| 1/17/2000 | 379.45 | 6.27 | 0 | 0 | 1 | 0 | 0 | 0 |
| 1/18/2000 | 273.82 | 5.67 | 0 | 0 | 3 | 0 | 0 | 0 |
| 1/19/2000 | 206.15 | 6.12 | 0 | 0 | 45 | 0 | 0 | 0 |
| 1/20/2000 | 240.69 | 6.72 | 0 | 0 | 48 | 1 | 0 | 0 |
| 1/21/2000 | 224.84 | 6.33 | 0 | 0 | 21 | 0 | 0 | 0 |
| 1/22/2000 | 186.61 | 6.64 | 0 | 0 | 75 | 0 | 0 | 0 |
| 1/23/2000 | 161.41 | 6.12 | 0 | 0 | 23 | 0 | 0 | 0 |
| 1/24/2000 | 170.47 | 6.49 | 0 | 0 | 13 | 0 | 0 | 0 |

| Date | Discharge (cms) | Temp (C°) | Chinook | Coho | Steelhead | Cutthroat | Lamprey | Sucker |
|---|---|---|---|---|---|---|---|---|
| 1/25/2000 | 210.68 | 6.87 | 0 | 0 | 86 | 0 | 0 | 0 |
| 1/26/2000 | 194.54 | 7.01 | 0 | 0 | 92 | 0 | 0 | 0 |
| 1/27/2000 | 167.64 | 5.68 | 0 | 0 | 27 | 0 | 0 | 0 |
| 1/28/2000 | 144.13 | 5.05 | 0 | 0 | 5 | 0 | 0 | 0 |
| 1/29/2000 | 123.46 | 4.28 | 0 | 0 | 0 | 0 | 0 | 0 |
| 1/30/2000 | 110.44 | 4.31 | 0 | 0 | 2 | 0 | 0 | 0 |
| 1/31/2000 | 101.66 | 5.21 | 0 | 0 | 35 | 0 | 0 | 0 |
| 2/1/2000 | 120.06 | 6.35 | 0 | 0 | 129 | 0 | 0 | 0 |
| 2/2/2000 | 407.76 | 7.14 | 0 | 0 | 30 | 0 | 0 | 0 |
| 2/3/2000 | 297.33 | 6.96 | 0 | 0 | 8 | 0 | 0 | 0 |
| 2/4/2000 | 211.53 | 7.27 | 0 | 0 | 67 | 0 | 0 | 0 |
| 2/5/2000 | 182.93 | 7.22 | 0 | 0 | 131 | 1 | 0 | 0 |
| 2/6/2000 | 165.09 | 7.54 | 0 | 0 | 120 | 0 | 0 | 0 |
| 2/7/2000 | 150.65 | 7.33 | 0 | 0 | 155 | 0 | 0 | 0 |
| 2/8/2000 | 142.72 | 7.29 | 0 | 0 | 74 | 0 | 0 | 0 |
| 2/9/2000 | 141.87 | 7.44 | 0 | 0 | 124 | 0 | 0 | 0 |
| 2/10/2000 | 137.62 | 7.71 | 0 | 0 | 126 | 0 | 0 | 0 |
| 2/11/2000 | 138.75 | 7.21 | 0 | 0 | 23 | 0 | 0 | 0 |
| 2/12/2000 | 162.54 | 7.42 | 0 | 0 | 17 | 0 | 0 | 0 |
| 2/13/2000 | 193.40 | 7.10 | 0 | 0 | 0 | 0 | 0 | 0 |
| 2/14/2000 | 342.63 | 7.24 | 0 | 0 | 0 | 0 | 0 | 0 |
| 2/15/2000 | 464.40 | 7.23 | 0 | 0 | 0 | 0 | 0 | 0 |
| 2/16/2000 | 302.99 | 7.13 | 0 | 0 | 0 | 0 | 0 | 0 |
| 2/17/2000 | 207.56 | 7.33 | 0 | 0 | 7 | 0 | 0 | 0 |
| 2/18/2000 | 169.62 | 7.02 | 0 | 0 | 33 | 0 | 0 | 0 |
| 2/19/2000 | 143.57 | 6.42 | 0 | 0 | 8 | 0 | 0 | 0 |
| 2/20/2000 | 127.71 | 5.91 | 0 | 0 | 11 | 0 | 0 | 0 |
| 2/21/2000 | 118.65 | 7.01 | 0 | 0 | 43 | 0 | 0 | 0 |
| 2/22/2000 | 117.23 | 7.36 | 0 | 0 | 108 | 0 | 0 | 0 |
| 2/23/2000 | 171.88 | 6.89 | 0 | 0 | 25 | 0 | 0 | 0 |
| 2/24/2000 | 157.72 | 6.80 | 0 | 0 | 16 | 0 | 0 | 0 |
| 2/25/2000 | 138.19 | 6.33 | 0 | 0 | 16 | 0 | 0 | 0 |
| 2/26/2000 | 170.75 | 6.71 | 0 | 0 | 27 | 0 | 0 | 0 |
| 2/27/2000 | 297.33 | 7.61 | 0 | 0 | 8 | 0 | 0 | 0 |
| 2/28/2000 | 258.82 | 7.60 | 0 | 0 | 8 | 0 | 0 | 0 |
| 2/29/2000 | 241.83 | 7.66 | 0 | 0 | 0 | 0 | 0 | 0 |
| 3/1/2000 | 222.57 | 7.01 | 0 | 0 | 3 | 0 | 0 | 0 |
| 3/2/2000 | 191.71 | 7.50 | 0 | 0 | 32 | 0 | 0 | 0 |
| 3/3/2000 | 168.77 | 8.25 | 0 | 0 | 255 | 0 | 0 | 0 |
| 3/4/2000 | 163.67 | 7.98 | 0 | 0 | 132 | 1 | 0 | 0 |
| 3/5/2000 | 173.30 | 7.08 | 0 | 0 | 3 | 0 | 0 | 0 |
| 3/6/2000 | 157.16 | 6.99 | 0 | 0 | 5 | 0 | 0 | 0 |
| 3/7/2000 | 136.20 | 6.23 | 0 | 0 | 5 | 0 | 0 | 0 |
| 3/8/2000 | 124.31 | 7.03 | 0 | 0 | 2 | 0 | 0 | 0 |
| 3/9/2000 | 109.87 | 7.40 | 0 | 0 | 56 | 0 | 0 | 0 |

| Date | Discharge (cms) | Temp (C°) | Chinook | Coho | Steelhead | Cutthroat | Lamprey | Sucker |
|---|---|---|---|---|---|---|---|---|
| 3/10/2000 | 102.51 | 7.12 | 0 | 0 | 16 | 0 | 0 | 0 |
| 3/11/2000 | 105.06 | 7.42 | 0 | 0 | 16 | 0 | 0 | 0 |
| 3/12/2000 | 104.77 | 7.59 | 0 | 0 | 20 | 0 | 0 | 0 |
| 3/13/2000 | 96.84 | 6.96 | 0 | 0 | 14 | 0 | 0 | 0 |
| 3/14/2000 | 121.20 | 8.45 | 0 | 0 | 157 | 0 | 0 | 0 |
| 3/15/2000 | 136.49 | 8.09 | 0 | 0 | 72 | 0 | 0 | 0 |
| 3/16/2000 | 140.73 | 8.59 | 0 | 0 | 211 | 0 | 0 | 0 |
| 3/17/2000 | 188.87 | 7.59 | 0 | 0 | 37 | 0 | 0 | 0 |
| 3/18/2000 | 166.22 | 7.25 | 0 | 0 | 36 | 0 | 0 | 0 |
| 3/19/2000 | 246.64 | 7.46 | 0 | 0 | 10 | 0 | 0 | 0 |
| 3/20/2000 | 248.06 | 7.69 | 0 | 0 | 13 | 0 | 0 | 0 |
| 3/21/2000 | 205.58 | 7.50 | 0 | 0 | 4 | 0 | 0 | 0 |
| 3/22/2000 | 177.26 | 7.69 | 0 | 0 | 25 | 0 | 0 | 0 |
| 3/23/2000 | 173.02 | 9.20 | 0 | 0 | 202 | 0 | 0 | 0 |
| 3/24/2000 | 162.54 | 7.83 | 0 | 0 | 22 | 0 | 0 | 0 |
| 3/25/2000 | 142.72 | 7.94 | 0 | 0 | 110 | 0 | 0 | 0 |
| 3/26/2000 | 127.99 | 9.28 | 0 | 0 | 152 | 0 | 0 | 0 |
| 3/27/2000 | 120.06 | 8.88 | 0 | 0 | 225 | 0 | 0 | 0 |
| 3/28/2000 | 118.08 | 8.86 | 0 | 0 | 54 | 0 | 0 | 0 |
| 3/29/2000 | 108.45 | 8.19 | 0 | 0 | 4 | 0 | 0 | 0 |
| 3/30/2000 | 96.84 | 8.72 | 0 | 0 | 9 | 0 | 0 | 0 |
| 3/31/2000 | 91.75 | 9.78 | 0 | 0 | 45 | 1 | 0 | 0 |
| 4/1/2000 | 87.50 | 10.56 | 1 | 0 | 142 | 1 | 0 | 7 |
| 4/2/2000 | 88.07 | 11.28 | 1 | 0 | 219 | 2 | 0 | 33 |
| 4/3/2000 | 94.58 | 11.99 | 3 | 0 | 126 | 1 | 0 | 79 |
| 4/4/2000 | 105.06 | 10.83 | 5 | 0 | 137 | 0 | 0 | 14 |
| 4/5/2000 | 100.24 | 11.08 | 1 | 0 | 26 | 1 | 0 | 30 |
| 4/6/2000 | 93.73 | 11.05 | 10 | 0 | 38 | 0 | 0 | 92 |
| 4/7/2000 | 86.65 | 10.28 | 1 | 0 | 14 | 1 | 0 | 10 |
| 4/8/2000 | 84.95 | 10.70 | 9 | 0 | 44 | 0 | 0 | 2 |
| 4/9/2000 | 85.23 | 10.22 | 6 | 0 | 27 | 0 | 0 | 13 |
| 4/10/2000 | 84.38 | 11.29 | 5 | 0 | 52 | 0 | 0 | 527 |
| 4/11/2000 | 82.69 | 11.91 | 15 | 0 | 83 | 0 | 0 | 1145 |
| 4/12/2000 | 83.53 | 11.54 | 33 | 0 | 76 | 0 | 0 | 1204 |
| 4/13/2000 | 142.72 | 11.43 | 8 | 0 | 28 | 0 | 1 | 385 |
| 4/14/2000 | 164.24 | 10.70 | 2 | 0 | 17 | 0 | 0 | 32 |
| 4/15/2000 | 149.80 | 10.31 | 7 | 0 | 20 | 0 | 0 | 0 |
| 4/16/2000 | 178.11 | 9.79 | 1 | 0 | 7 | 0 | 0 | 0 |
| 4/17/2000 | 166.50 | 10.64 | 7 | 0 | 16 | 0 | 0 | 0 |
| 4/18/2000 | 305.82 | 10.28 | 0 | 0 | 1 | 0 | 0 | 0 |
| 4/19/2000 | 297.33 | 8.77 | 0 | 0 | 2 | 0 | 0 | 0 |
| 4/20/2000 | 210.68 | 10.10 | 0 | 0 | 7 | 0 | 0 | 0 |
| 4/21/2000 | 167.35 | 11.19 | 14 | 0 | 31 | 0 | 0 | 3 |
| 4/22/2000 | 148.95 | 11.57 | 18 | 0 | 11 | 0 | 0 | 94 |
| 4/23/2000 | 141.02 | 10.51 | 0 | 0 | 1 | 0 | 0 | 0 |

| Date | Discharge (cms) | Temp (C°) | Chinook | Coho | Steelhead | Cutthroat | Lamprey | Sucker |
|---|---|---|---|---|---|---|---|---|
| 4/24/2000 | 129.12 | 9.51 | 0 | 0 | 0 | 0 | 0 | 0 |
| 4/25/2000 | 123.18 | 9.74 | 0 | 0 | 0 | 0 | 0 | 0 |
| 4/26/2000 | 123.18 | 9.96 | 0 | 0 | 14 | 0 | 0 | 0 |
| 4/27/2000 | 117.23 | 10.86 | 65 | 0 | 17 | 0 | 0 | 16 |
| 4/28/2000 | 118.36 | 10.54 | 11 | 0 | 3 | 0 | 0 | 0 |
| 4/29/2000 | 111.57 | 10.90 | 32 | 0 | 3 | 0 | 0 | 0 |
| 4/30/2000 | 106.75 | 11.87 | 24 | 0 | 9 | 0 | 0 | 180 |
| 5/1/2000 | 100.81 | 11.50 | 7 | 0 | 10 | 0 | 0 | 1397 |
| 5/2/2000 | 97.69 | 13.53 | 129 | 0 | 7 | 1 | 0 | 808 |
| 5/3/2000 | 95.71 | 12.31 | 197 | 0 | 13 | 0 | 0 | 502 |
| 5/4/2000 | 99.68 | 12.37 | 51 | 0 | 4 | 1 | 0 | 23 |
| 5/5/2000 | 97.13 | 12.18 | 67 | 0 | 4 | 0 | 0 | 2 |
| 5/6/2000 | 95.43 | 12.33 | 29 | 0 | 3 | 0 | 0 | 2 |
| 5/7/2000 | 88.35 | 12.04 | 93 | 0 | 6 | 0 | 0 | 50 |
| 5/8/2000 | 88.35 | 12.51 | 91 | 0 | 6 | 0 | 0 | 43 |
| 5/9/2000 | 94.30 | 12.05 | 5 | 0 | 0 | 0 | 0 | 12 |
| 5/10/2000 | 133.94 | 10.77 | 6 | 0 | 2 | 0 | 0 | 11 |
| 5/11/2000 | 181.23 | 8.81 | 27 | 0 | 1 | 0 | 0 | 0 |
| 5/12/2000 | 205.30 | 8.84 | 1 | 0 | 0 | 0 | 0 | 0 |
| 5/13/2000 | 179.25 | 10.11 | 7 | 0 | 3 | 0 | 0 | 0 |
| 5/14/2000 | 159.42 | 10.36 | 0 | 0 | 0 | 0 | 0 | 0 |
| 5/15/2000 | 145.83 | 11.06 | 14 | 0 | 9 | 0 | 0 | 38 |
| 5/16/2000 | 160.56 | 11.54 | 0 | 0 | 0 | 0 | 0 | 0 |
| 5/17/2000 | 155.74 | 10.57 | 39 | 0 | 6 | 0 | 0 | 30 |
| 5/18/2000 | 132.81 | 12.92 | 167 | 0 | 5 | 0 | 1 | 1 |
| 5/19/2000 | 115.82 | 13.81 | 243 | 0 | 9 | 0 | 0 | 6 |
| 5/20/2000 | 108.17 | 14.20 | 231 | 0 | 17 | 1 | 0 | 1174 |
| 5/21/2000 | 102.79 | 14.63 | 151 | 0 | 5 | 0 | 2 | 684 |
| 5/22/2000 | 101.09 | 14.31 | 102 | 0 | 8 | 0 | 1 | 609 |
| 5/23/2000 | 98.26 | 14.70 | 112 | 0 | 10 | 0 | 1 | 163 |
| 5/24/2000 | 95.14 | 15.69 | 78 | 0 | 4 | 0 | 0 | 16 |
| 5/25/2000 | 92.03 | 14.39 | 100 | 0 | 7 | 0 | 0 | 50 |
| 5/26/2000 | 86.65 | 14.82 | 105 | 0 | 10 | 0 | 1 | 58 |
| 5/27/2000 | 81.84 | 14.17 | 75 | 0 | 3 | 0 | 0 | 24 |
| 5/28/2000 | 90.61 | 13.74 | 82 | 0 | 5 | 0 | 0 | 19 |
| 5/29/2000 | 81.27 | 13.37 | 15 | 0 | 0 | 0 | 0 | 1 |
| 5/30/2000 | 76.74 | 13.94 | 97 | 0 | 4 | 0 | 0 | 0 |
| 5/31/2000 | 74.76 | 13.04 | 42 | 0 | 12 | 0 | 0 | 1 |
| 6/1/2000 | 69.94 | 13.20 | 68 | 0 | 16 | 0 | 0 | 10 |
| 6/2/2000 | 66.54 | 15.00 | 112 | 0 | 37 | 0 | 0 | 803 |
| 6/3/2000 | 64.00 | 15.94 | 144 | 0 | 40 | 0 | 2 | 437 |
| 6/4/2000 | 63.71 | 16.69 | 49 | 0 | 6 | 0 | 0 | 101 |
| 6/5/2000 | 62.86 | 16.48 | 43 | 0 | 10 | 0 | 0 | 657 |
| 6/6/2000 | 62.30 | 16.42 | 22 | 0 | 5 | 0 | 0 | 0 |
| 6/7/2000 | 61.16 | 15.45 | 104 | 0 | 66 | 0 | 0 | 167 |

| Date | Discharge (cms) | Temp (C°) | Chinook | Coho | Steelhead | Cutthroat | Lamprey | Sucker |
|---|---|---|---|---|---|---|---|---|
| 6/8/2000 | 62.30 | 14.61 | 53 | 0 | 29 | 0 | 0 | 1 |
| 6/9/2000 | 61.73 | 14.01 | 25 | 0 | 30 | 0 | 0 | 0 |
| 6/10/2000 | 65.70 | 13.10 | 45 | 0 | 25 | 0 | 0 | 0 |
| 6/11/2000 | 65.13 | 12.43 | 33 | 0 | 24 | 0 | 0 | 0 |
| 6/12/2000 | 71.08 | 12.68 | 25 | 0 | 25 | 0 | 0 | 0 |
| 6/13/2000 | 107.60 | 12.42 | 18 | 0 | 18 | 0 | 0 | 25 |
| 6/14/2000 | 92.03 | 14.63 | 57 | 0 | 3 | 0 | 0 | 4612 |
| 6/15/2000 | 75.89 | 17.50 | 72 | 0 | 81 | 0 | 2 | 132 |
| 6/16/2000 | 68.81 | 17.38 | 89 | 0 | 52 | 0 | 2 | 7 |
| 6/17/2000 | 64.85 | 17.65 | 60 | 0 | 36 | 0 | 0 | 3 |
| 6/18/2000 | 59.75 | 17.89 | 16 | 0 | 10 | 0 | 0 | 0 |
| 6/19/2000 | 55.50 | 17.75 | 9 | 0 | 1 | 0 | 0 | 13 |
| 6/20/2000 | 56.92 | 17.67 | 5 | 0 | 65 | 0 | 0 | 21 |
| 6/21/2000 | 54.37 | 18.56 | 68 | 0 | 42 | 1 | 0 | 349 |
| 6/22/2000 | 52.10 | 19.04 | 52 | 0 | 103 | 0 | 3 | 114 |
| 6/23/2000 | 50.40 | 18.68 | 47 | 0 | 86 | 1 | 0 | 13 |
| 6/24/2000 | 48.42 | 18.87 | 14 | 0 | 74 | 1 | 1 | 18 |
| 6/25/2000 | 47.57 | 19.55 | 11 | 0 | 53 | 0 | 1 | 78 |
| 6/26/2000 | 46.72 | 19.88 | 11 | 0 | 26 | 0 | 2 | 2 |
| 6/27/2000 | 44.74 | 20.43 | 8 | 0 | 78 | 1 | 5 | 700 |
| 6/28/2000 | 42.19 | 21.53 | 0 | 0 | 0 | 2 | 0 | 0 |
| 6/29/2000 | 41.63 | 22.14 | 10 | 0 | 19 | 2 | 0 | 16 |
| 6/30/2000 | 40.78 | 20.99 | 16 | 0 | 104 | 0 | 0 | 3 |
| 7/1/2000 | 44.46 | 19.88 | 23 | 0 | 28 | 1 | 1 | 3 |
| 7/2/2000 | 44.74 | 17.93 | 1 | 0 | 3 | 0 | 0 | 0 |
| 7/3/2000 | 41.63 | 18.22 | 0 | 0 | 0 | 0 | 0 | 0 |
| 7/4/2000 | 41.91 | 17.08 | 17 | 0 | 60 | 0 | 0 | 45 |
| 7/5/2000 | 40.21 | 16.78 | 0 | 0 | 0 | 0 | 0 | 0 |
| 7/6/2000 | 38.51 | 17.06 | 0 | 0 | 3 | 0 | 0 | 0 |
| 7/7/2000 | 39.36 | 18.84 | 0 | 0 | 0 | 0 | 0 | 0 |
| 7/8/2000 | 44.17 | 18.84 | 0 | 0 | 0 | 0 | 0 | 0 |
| 7/9/2000 | 42.76 | 18.18 | 8 | 0 | 152 | 1 | 0 | 23 |
| 7/10/2000 | 39.08 | 18.60 | 13 | 0 | 341 | 0 | 0 | 3 |
| 7/11/2000 | 40.78 | 19.56 | 0 | 0 | 0 | 0 | 0 | 0 |
| 7/12/2000 | 41.91 | 19.30 | 7 | 0 | 227 | 0 | 1 | 2 |
| 7/13/2000 | 40.21 | 19.89 | 24 | 0 | 183 | 0 | 2 | 0 |
| 7/14/2000 | 38.79 | 19.44 | 13 | 0 | 145 | 0 | 0 | 1 |
| 7/15/2000 | 37.94 | 18.49 | 2 | 0 | 50 | 1 | 0 | 0 |
| 7/16/2000 | 35.11 | 19.04 | 8 | 0 | 33 | 0 | 0 | 0 |
| 7/17/2000 | 33.70 | 20.04 | 11 | 0 | 20 | 2 | 0 | 2 |
| 7/18/2000 | 35.96 | 20.14 | 0 | 0 | 34 | 2 | 1 | 0 |
| 7/19/2000 | 37.94 | 19.28 | 18 | 0 | 108 | 3 | 0 | 1 |
| 7/20/2000 | 37.66 | 19.71 | 4 | 0 | 48 | 1 | 0 | 0 |
| 7/21/2000 | 36.81 | 20.99 | 18 | 0 | 72 | 2 | 0 | 3 |
| 7/22/2000 | 37.10 | 20.85 | 12 | 0 | 43 | 2 | 3 | 2 |

| Date | Discharge (cms) | Temp (C°) | Chinook | Coho | Steelhead | Cutthroat | Lamprey | Sucker |
|---|---|---|---|---|---|---|---|---|
| 7/23/2000 | 33.70 | 19.80 | 9 | 0 | 51 | 1 | 0 | 0 |
| 7/24/2000 | 30.87 | 20.20 | 11 | 0 | 24 | 1 | 0 | 0 |
| 7/25/2000 | 34.55 | 21.05 | 0 | 0 | 6 | 0 | 1 | 0 |
| 7/26/2000 | 35.96 | 20.56 | 6 | 0 | 54 | 3 | 0 | 0 |
| 7/27/2000 | 36.53 | 20.16 | 8 | 0 | 22 | 1 | 0 | 0 |
| 7/28/2000 | 34.26 | 20.84 | 4 | 0 | 32 | 1 | 0 | 0 |
| 7/29/2000 | 31.43 | 21.26 | 1 | 0 | 17 | 4 | 0 | 0 |
| 7/30/2000 | 28.32 | 21.88 | 3 | 0 | 38 | 1 | 0 | 0 |
| 7/31/2000 | 31.43 | 22.66 | 4 | 0 | 33 | 1 | 0 | 0 |
| 8/1/2000 | 32.56 | 22.36 | 4 | 0 | 17 | 5 | 0 | 0 |
| 8/2/2000 | 32.00 | 21.69 | 4 | 0 | 20 | 1 | 0 | 0 |
| 8/3/2000 | 32.56 | 21.54 | 2 | 0 | 13 | 1 | 0 | 0 |
| 8/4/2000 | 30.87 | 21.62 | 2 | 0 | 9 | 0 | 0 | 0 |
| 8/5/2000 | 30.58 | 21.34 | 3 | 0 | 6 | 0 | 0 | 0 |
| 8/6/2000 | 26.42 | 21.85 | 0 | 0 | 1 | 0 | 0 | 0 |
| 8/7/2000 | 25.20 | 22.29 | 0 | 0 | 3 | 0 | 0 | 0 |
| 8/8/2000 | 27.38 | 22.73 | 1 | 0 | 1 | 0 | 0 | 0 |
| 8/9/2000 | 31.71 | 22.18 | 1 | 0 | 0 | 0 | 0 | 0 |
| 8/10/2000 | 30.30 | 21.18 | 0 | 0 | 0 | 0 | 0 | 0 |
| 8/11/2000 | 28.60 | 20.27 | 1 | 0 | 6 | 0 | 0 | 0 |
| 8/12/2000 | 28.15 | 19.97 | 0 | 0 | 2 | 1 | 0 | 0 |
| 8/13/2000 | 26.73 | 19.80 | 4 | 0 | 0 | 0 | 0 | 0 |
| 8/14/2000 | 25.20 | 19.71 | 13 | 0 | 7 | 0 | 0 | 0 |
| 8/15/2000 | 26.14 | 19.29 | 9 | 0 | 15 | 0 | 0 | 0 |
| 8/16/2000 | 27.92 | 19.39 | 11 | 0 | 26 | 0 | 0 | 0 |
| 8/17/2000 | 27.16 | 19.09 | 14 | 0 | 40 | 0 | 0 | 0 |
| 8/18/2000 | 26.33 | 18.74 | 9 | 0 | 45 | 3 | 0 | 0 |
| 8/19/2000 | 25.71 | 19.04 | 2 | 0 | 33 | 0 | 0 | 0 |
| 8/20/2000 | 25.85 | 18.71 | 3 | 0 | 38 | 0 | 0 | 0 |
| 8/21/2000 | 26.02 | 18.71 | 10 | 0 | 36 | 0 | 0 | 0 |
| 8/22/2000 | 26.48 | 18.97 | 5 | 0 | 38 | 1 | 0 | 0 |
| 8/23/2000 | 29.45 | 19.64 | 5 | 0 | 21 | 0 | 0 | 0 |
| 8/24/2000 | 29.17 | 19.57 | 5 | 0 | 13 | 1 | 1 | 0 |
| 8/25/2000 | 27.33 | 18.95 | 14 | 0 | 23 | 1 | 0 | 0 |
| 8/26/2000 | 27.78 | 19.39 | 4 | 0 | 18 | 0 | 0 | 0 |
| 8/27/2000 | 26.16 | 19.06 | 7 | 0 | 13 | 0 | 0 | 0 |
| 8/28/2000 | 25.23 | 18.29 | 1 | 0 | 7 | 0 | 0 | 0 |
| 8/29/2000 | 25.91 | | 0 | 0 | 0 | 0 | 0 | 0 |
| 8/30/2000 | 27.16 | | 0 | 0 | 0 | 0 | 0 | 0 |
| 8/31/2000 | 27.78 | 16.94 | 9 | 0 | 31 | 0 | 0 | 0 |
| 9/1/2000 | 27.81 | 15.80 | 5 | 0 | 8 | 0 | 0 | 0 |
| 9/2/2000 | 28.60 | 15.16 | 4 | 0 | 36 | 1 | 0 | 0 |
| 9/3/2000 | 29.73 | 15.76 | 2 | 1 | 16 | 0 | 0 | 1 |
| 9/4/2000 | 31.15 | 15.50 | 2 | 1 | 9 | 0 | 0 | 0 |
| 9/5/2000 | 29.17 | 15.59 | 2 | 1 | 11 | 1 | 0 | 0 |

| Date | Discharge (cms) | Temp (C°) | Chinook | Coho | Steelhead | Cutthroat | Lamprey | Sucker |
|---|---|---|---|---|---|---|---|---|
| 9/6/2000 | 28.60 | 15.92 | 2 | 0 | 2 | 0 | 0 | 0 |
| 9/7/2000 | 30.02 | | 0 | 0 | 0 | 0 | 0 | 0 |
| 9/8/2000 | 26.31 | | 0 | 0 | 0 | 0 | 0 | 0 |
| 9/9/2000 | 31.43 | 15.77 | 2 | 0 | 10 | 1 | 0 | 0 |
| 9/10/2000 | 32.85 | 15.59 | 9 | 3 | 34 | 2 | 0 | 0 |
| 9/11/2000 | 32.56 | 15.67 | 4 | 0 | 7 | 1 | 0 | 0 |
| 9/12/2000 | 32.56 | 16.84 | 0 | 0 | 0 | 0 | 0 | 0 |
| 9/13/2000 | 32.56 | 16.89 | 3 | 0 | 19 | 0 | 0 | 0 |
| 9/14/2000 | 31.71 | | 0 | 0 | 0 | 0 | 0 | 0 |
| 9/15/2000 | 31.15 | 17.20 | 13 | 16 | 26 | 3 | 0 | 0 |
| 9/16/2000 | 32.85 | 17.09 | 12 | 10 | 18 | 0 | 0 | 0 |
| 9/17/2000 | 29.45 | 17.25 | 8 | 17 | 19 | 1 | 0 | 0 |
| 9/18/2000 | 30.02 | 17.37 | 10 | 12 | 7 | 0 | 0 | 0 |
| 9/19/2000 | 30.30 | 18.45 | 3 | 11 | 11 | 0 | 0 | 0 |
| 9/20/2000 | 26.79 | 17.91 | 5 | 30 | 18 | 2 | 0 | 0 |
| 9/21/2000 | 32.85 | 17.25 | 10 | 33 | 36 | 0 | 0 | 0 |
| 9/22/2000 | 30.02 | 16.05 | 2 | 8 | 6 | 0 | 0 | 0 |
| 9/23/2000 | 24.58 | 15.41 | 3 | 5 | 0 | 1 | 0 | 0 |
| 9/24/2000 | 23.96 | 15.11 | 0 | 6 | 3 | 0 | 0 | 0 |
| 9/25/2000 | 23.87 | 14.65 | 3 | 6 | 5 | 1 | 0 | 0 |
| 9/26/2000 | 24.07 | 14.12 | 1 | 2 | 1 | 0 | 0 | 0 |
| 9/27/2000 | 24.15 | | 0 | 0 | 0 | 0 | 0 | 0 |
| 9/28/2000 | 24.21 | | 0 | 0 | 0 | 0 | 0 | 0 |
| 9/29/2000 | 24.04 | 14.95 | 12 | 71 | 19 | 0 | 0 | 0 |
| 9/30/2000 | 24.13 | 14.86 | 9 | 62 | 7 | 0 | 0 | 0 |
| 10/1/2000 | 24.21 | 15.58 | 4 | 42 | 0 | 0 | 0 | 0 |
| 10/2/2000 | 24.27 | 15.31 | 3 | 161 | 14 | 1 | 0 | 0 |
| 10/3/2000 | 24.30 | 14.15 | 5 | 91 | 0 | 0 | 0 | 0 |
| 10/4/2000 | 23.98 | 13.54 | 1 | 65 | 11 | 2 | 0 | 0 |
| 10/5/2000 | 24.07 | 13.18 | 0 | 57 | 3 | 0 | 0 | 0 |
| 10/6/2000 | 24.15 | 12.93 | 1 | 46 | 2 | 2 | 0 | 0 |
| 10/7/2000 | 24.10 | 13.01 | 3 | 32 | 1 | 0 | 0 | 0 |
| 10/8/2000 | 23.90 | 12.36 | 3 | 24 | 0 | 0 | 0 | 0 |
| 10/9/2000 | 25.37 | 12.44 | 1 | 11 | 4 | 0 | 0 | 0 |
| 10/10/2000 | 26.70 | 11.69 | 0 | 0 | 0 | 1 | 0 | 0 |
| 10/11/2000 | 27.64 | 11.31 | 7 | 57 | 14 | 1 | 0 | 0 |
| 10/12/2000 | 27.16 | 11.08 | 11 | 54 | 21 | 0 | 0 | 0 |
| 10/13/2000 | 26.56 | 11.36 | 3 | 34 | 8 | 0 | 0 | 0 |
| 10/14/2000 | 26.05 | 11.92 | 17 | 100 | 27 | 0 | 0 | 0 |
| 10/15/2000 | 25.94 | 12.14 | 9 | 72 | 7 | 1 | 0 | 0 |
| 10/16/2000 | 26.70 | 12.07 | 8 | 23 | 0 | 0 | 0 | 0 |
| 10/17/2000 | 26.90 | 11.71 | 1 | 14 | 1 | 0 | 0 | 0 |
| 10/18/2000 | 26.93 | 11.58 | 0 | 0 | 0 | 0 | 0 | 0 |
| 10/19/2000 | 26.79 | 11.65 | 8 | 100 | 9 | 0 | 0 | 0 |
| 10/20/2000 | 28.88 | 11.86 | 30 | 90 | 47 | 0 | 0 | 0 |

| Date | Discharge (cms) | Temp (C°) | Chinook | Coho | Steelhead | Cutthroat | Lamprey | Sucker |
|---|---|---|---|---|---|---|---|---|
| 10/21/2000 | 47.29 | 11.47 | 9 | 86 | 13 | 0 | 0 | 0 |
| 10/22/2000 | 45.31 | 10.61 | 1 | 34 | 0 | 0 | 0 | 0 |
| 10/23/2000 | 33.98 | 10.18 | 8 | 500 | 42 | 0 | 0 | 0 |
| 10/24/2000 | 30.02 | 9.36 | 0 | 112 | 8 | 0 | 0 | 0 |
| 10/25/2000 | 28.60 | 9.34 | 2 | 12 | 0 | 0 | 0 | 0 |
| 10/26/2000 | 27.72 | 9.36 | 0 | 19 | 4 | 0 | 0 | 0 |
| 10/27/2000 | 27.04 | 10.47 | 6 | 363 | 40 | 1 | 0 | 0 |
| 10/28/2000 | 29.73 | 10.57 | 26 | 242 | 26 | 1 | 0 | 0 |
| 10/29/2000 | 39.64 | 10.28 | 2 | 38 | 1 | 1 | 0 | 0 |
| 10/30/2000 | 38.79 | 9.92 | 0 | 95 | 0 | 0 | 0 | 0 |
| 10/31/2000 | 33.70 | 9.80 | 0 | 0 | 0 | 0 | 0 | 0 |
| 11/1/2000 | 32.85 | 8.94 | 0 | 106 | 18 | 1 | 0 | 0 |
| 11/2/2000 | 32.56 | 8.82 | 1 | 52 | 5 | 1 | 0 | 0 |
| 11/3/2000 | 32.28 | 9.37 | 2 | 58 | 4 | 0 | 0 | 0 |
| 11/4/2000 | 34.83 | 9.93 | 4 | 77 | 10 | 0 | 0 | 0 |
| 11/5/2000 | 35.96 | 8.88 | 0 | 71 | 4 | 0 | 0 | 0 |
| 11/6/2000 | 35.11 | 8.73 | 0 | 2 | 0 | 0 | 0 | 0 |
| 11/7/2000 | 32.28 | 8.26 | 0 | 0 | 0 | 0 | 0 | 0 |
| 11/8/2000 | 34.83 | 8.15 | 0 | 0 | 0 | 0 | 0 | 0 |
| 11/9/2000 | 60.31 | 7.50 | 1 | 103 | 21 | 0 | 0 | 0 |
| 11/10/2000 | 52.10 | 6.88 | 1 | 95 | 0 | 0 | 0 | 0 |
| 11/11/2000 | 44.17 | 6.25 | 0 | 0 | 0 | 0 | 0 | 0 |
| 11/12/2000 | 39.93 | 5.36 | 0 | 0 | 0 | 0 | 0 | 0 |
| 11/13/2000 | 35.96 | 4.66 | 0 | 4 | 1 | 0 | 0 | 0 |
| 11/14/2000 | 34.83 | 4.72 | 0 | 0 | 0 | 0 | 0 | 0 |
| 11/15/2000 | 35.11 | 5.04 | 0 | 0 | 0 | 0 | 0 | 0 |
| 11/16/2000 | 34.26 | 5.21 | 0 | 5 | 1 | 0 | 0 | 0 |
| 11/17/2000 | 32.85 | 4.86 | 0 | 2 | 0 | 0 | 0 | 0 |
| 11/18/2000 | 32.56 | 4.36 | 0 | 1 | 0 | 0 | 0 | 0 |
| 11/19/2000 | 32.85 | 3.87 | 0 | 0 | 0 | 0 | 0 | 0 |
| 11/20/2000 | 32.56 | 3.49 | 0 | 0 | 0 | 0 | 0 | 0 |
| 11/21/2000 | 32.56 | 3.96 | 0 | 0 | 0 | 0 | 0 | 0 |
| 11/22/2000 | 32.28 | 3.83 | 0 | 2 | 0 | 0 | 0 | 0 |
| 11/23/2000 | 32.00 | 3.74 | 0 | 0 | 0 | 0 | 0 | 0 |
| 11/24/2000 | 34.26 | 4.39 | 0 | 2 | 0 | 0 | 0 | 0 |
| 11/25/2000 | 41.34 | 5.25 | 0 | 33 | 3 | 0 | 0 | 0 |
| 11/26/2000 | 41.63 | 5.55 | 0 | 82 | 4 | 1 | 0 | 0 |
| 11/27/2000 | 44.17 | 6.07 | 1 | 131 | 18 | 0 | 0 | 0 |
| 11/28/2000 | 49.55 | 5.76 | 0 | 76 | 9 | 0 | 0 | 0 |
| 11/29/2000 | 47.29 | 5.81 | 1 | 16 | 0 | 0 | 0 | 0 |
| 11/30/2000 | 57.48 | 6.26 | 0 | 0 | 0 | 0 | 0 | 0 |
| 12/1/2000 | 56.35 | 5.93 | 0 | 200 | 18 | 0 | 0 | 0 |
| 12/2/2000 | 48.14 | 5.91 | 0 | 106 | 5 | 0 | 0 | 0 |
| 12/3/2000 | 43.89 | 6.32 | 0 | 75 | 10 | 0 | 0 | 0 |
| 12/4/2000 | 41.34 | 6.07 | 0 | 39 | 20 | 0 | 0 | 0 |

| Date | Discharge (cms) | Temp (C°) | Chinook | Coho | Steelhead | Cutthroat | Lamprey | Sucker |
|---|---|---|---|---|---|---|---|---|
| 12/5/2000 | 38.51 | 6.00 | 0 | 43 | 9 | 0 | 0 | 0 |
| 12/6/2000 | 36.81 | 5.72 | 0 | 20 | 2 | 0 | 0 | 0 |
| 12/7/2000 | 35.68 | 4.94 | 0 | 4 | 0 | 0 | 0 | 0 |
| 12/8/2000 | 34.83 | 4.58 | 0 | 4 | 0 | 0 | 0 | 0 |
| 12/9/2000 | 33.98 | 5.00 | 0 | 7 | 1 | 0 | 0 | 0 |
| 12/10/2000 | 33.70 | 5.17 | 0 | 3 | 1 | 0 | 0 | 0 |
| 12/11/2000 | 33.70 | 5.06 | 0 | 2 | 1 | 0 | 0 | 0 |
| 12/12/2000 | 34.83 | 5.19 | 0 | 1 | 2 | 0 | 0 | 0 |
| 12/13/2000 | 37.94 | 5.27 | 0 | 25 | 12 | 0 | 0 | 0 |
| 12/14/2000 | 80.42 | 5.68 | 0 | 44 | 34 | 0 | 0 | 0 |
| 12/15/2000 | 136.77 | 6.14 | 0 | 34 | 30 | 0 | 0 | 0 |
| 12/16/2000 | 111.00 | 6.35 | 1 | 35 | 28 | 0 | 0 | 0 |
| 12/17/2000 | 104.77 | 6.12 | 0 | 26 | 16 | 0 | 0 | 0 |
| 12/18/2000 | 86.93 | 5.49 | 0 | 29 | 5 | 0 | 0 | 0 |
| 12/19/2000 | 64.85 | 4.99 | 0 | 41 | 0 | 0 | 0 | 0 |
| 12/20/2000 | 57.48 | 5.05 | 0 | 26 | 9 | 1 | 0 | 0 |
| 12/21/2000 | 55.50 | 5.45 | 0 | 17 | 7 | 0 | 0 | 0 |
| 12/22/2000 | 113.27 | 6.13 | 0 | 9 | 20 | 0 | 0 | 0 |
| 12/23/2000 | 195.67 | 6.88 | 0 | 14 | 75 | 0 | 0 | 0 |
| 12/24/2000 | 165.37 | 6.88 | 0 | 1 | 43 | 0 | 0 | 0 |
| 12/25/2000 | 118.08 | 6.22 | 0 | 2 | 7 | 0 | 0 | 0 |
| 12/26/2000 | 84.10 | 5.36 | 0 | 1 | 0 | 0 | 0 | 0 |
| 12/27/2000 | 70.23 | 5.30 | 0 | 0 | 0 | 0 | 0 | 0 |
| 12/28/2000 | 64.56 | 5.22 | 0 | 1 | 0 | 0 | 0 | 0 |
| 12/29/2000 | 59.75 | 4.93 | 0 | 0 | 0 | 0 | 0 | 0 |
| 12/30/2000 | 58.05 | 4.49 | 0 | 0 | 0 | 0 | 0 | 0 |
| 12/31/2000 | 55.22 | 4.71 | 0 | 0 | 0 | 0 | 0 | 0 |
| 1/1/2001 | 52.39 | 4.71 | 0 | 0 | 0 | 0 | 0 | 0 |
| 1/2/2001 | 50.40 | 4.99 | 0 | 0 | 1 | 0 | 0 | 0 |
| 1/3/2001 | 48.99 | 4.85 | 0 | 0 | 0 | 0 | 0 | 0 |
| 1/4/2001 | 48.42 | 5.15 | 0 | 0 | 0 | 0 | 0 | 0 |
| 1/5/2001 | 47.57 | 5.76 | 0 | 2 | 1 | 0 | 0 | 0 |
| 1/6/2001 | 47.57 | 5.87 | 0 | 1 | 3 | 0 | 0 | 0 |
| 1/7/2001 | 46.44 | 5.50 | 0 | 0 | 0 | 0 | 0 | 0 |
| 1/8/2001 | 45.87 | 5.24 | 0 | 0 | 3 | 0 | 0 | 0 |
| 1/9/2001 | 47.29 | 5.18 | 0 | 0 | 2 | 0 | 0 | 0 |
| 1/10/2001 | 47.29 | 5.13 | 0 | 0 | 3 | 0 | 0 | 0 |
| 1/11/2001 | 46.44 | 4.92 | 0 | 0 | 0 | 0 | 0 | 0 |
| 1/12/2001 | 44.74 | 5.14 | 0 | 0 | 2 | 0 | 0 | 0 |
| 1/13/2001 | 44.46 | 5.46 | 0 | 0 | 6 | 0 | 0 | 0 |
| 1/14/2001 | 49.55 | 5.37 | 0 | 0 | 45 | 0 | 0 | 0 |
| 1/15/2001 | 52.95 | 5.44 | 0 | 0 | 21 | 0 | 0 | 0 |
| 1/16/2001 | 50.40 | 4.92 | 0 | 0 | 1 | 0 | 0 | 0 |
| 1/17/2001 | 47.29 | 3.96 | 0 | 0 | 0 | 0 | 0 | 0 |
| 1/18/2001 | 45.31 | 3.73 | 0 | 0 | 0 | 0 | 0 | 0 |

| Date | Discharge (cms) | Temp (C°) | Chinook | Coho | Steelhead | Cutthroat | Lamprey | Sucker |
|---|---|---|---|---|---|---|---|---|
| 1/19/2001 | 44.46 | 4.09 | 0 | 0 | 1 | 0 | 0 | 0 |
| 1/20/2001 | 46.72 | 4.28 | 0 | 0 | 0 | 0 | 0 | 0 |
| 1/21/2001 | 48.42 | 4.80 | 0 | 0 | 6 | 0 | 0 | 0 |
| 1/22/2001 | 50.40 | 5.52 | 0 | 0 | 7 | 0 | 0 | 0 |
| 1/23/2001 | 51.25 | 5.76 | 0 | 0 | 29 | 0 | 0 | 0 |
| 1/24/2001 | 54.65 | 5.68 | 0 | 1 | 54 | 0 | 0 | 0 |
| 1/25/2001 | 63.15 | 5.52 | 0 | 0 | 115 | 0 | 0 | 0 |
| 1/26/2001 | 60.88 | 5.62 | 0 | 1 | 11 | 0 | 0 | 0 |
| 1/27/2001 | 55.50 | 4.78 | 0 | 0 | 1 | 0 | 0 | 0 |
| 1/28/2001 | 51.82 | 3.90 | 0 | 0 | 1 | 0 | 0 | 0 |
| 1/29/2001 | 50.97 | 4.22 | 0 | 0 | 1 | 0 | 0 | 0 |
| 1/30/2001 | 51.82 | 4.44 | 0 | 0 | 0 | 0 | 0 | 0 |
| 1/31/2001 | 48.99 | 4.36 | 0 | 1 | 1 | 0 | 0 | 0 |
| 2/1/2001 | 48.42 | 4.18 | 0 | 0 | 0 | 0 | 0 | 0 |
| 2/2/2001 | 48.42 | 5.09 | 0 | 0 | 4 | 0 | 0 | 0 |
| 2/3/2001 | 52.39 | 5.31 | 0 | 0 | 32 | 0 | 0 | 0 |
| 2/4/2001 | 73.34 | 6.16 | 0 | 0 | 250 | 0 | 0 | 0 |
| 2/5/2001 | 83.25 | 6.79 | 0 | 0 | 200 | 0 | 0 | 0 |
| 2/6/2001 | 73.62 | 6.50 | 0 | 0 | 80 | 0 | 0 | 0 |
| 2/7/2001 | 65.13 | 5.64 | 0 | 0 | 0 | 0 | 0 | 0 |
| 2/8/2001 | 59.75 | 4.44 | 0 | 0 | 0 | 0 | 0 | 0 |
| 2/9/2001 | 56.63 | 3.85 | 0 | 0 | 0 | 0 | 0 | 0 |
| 2/10/2001 | 54.37 | 4.22 | 0 | 0 | 0 | 0 | 0 | 0 |
| 2/11/2001 | 54.65 | 4.58 | 0 | 0 | 0 | 0 | 0 | 0 |
| 2/12/2001 | 53.80 | 4.50 | 0 | 0 | 0 | 0 | 0 | 0 |
| 2/13/2001 | 50.69 | 4.61 | 0 | 0 | 0 | 0 | 0 | 0 |
| 2/14/2001 | 49.27 | 4.02 | 0 | 0 | 0 | 0 | 0 | 0 |
| 2/15/2001 | 48.14 | 3.89 | 0 | 0 | 0 | 0 | 0 | 0 |
| 2/16/2001 | 47.01 | 4.42 | 0 | 0 | 0 | 0 | 0 | 0 |
| 2/17/2001 | 47.01 | 5.03 | 0 | 0 | 5 | 0 | 0 | 0 |
| 2/18/2001 | 47.57 | 6.13 | 0 | 0 | 57 | 0 | 0 | 0 |
| 2/19/2001 | 47.01 | 6.29 | 0 | 0 | 27 | 1 | 0 | 0 |
| 2/20/2001 | 45.87 | 6.25 | 0 | 0 | 43 | 0 | 0 | 0 |
| 2/21/2001 | 46.72 | 7.03 | 0 | 0 | 114 | 0 | 0 | 0 |
| 2/22/2001 | 48.42 | 6.91 | 0 | 0 | 187 | 0 | 0 | 0 |
| 2/23/2001 | 50.69 | 7.04 | 0 | 0 | 88 | 0 | 0 | 0 |
| 2/24/2001 | 50.40 | 6.46 | 0 | 0 | 25 | 0 | 0 | 0 |
| 2/25/2001 | 51.25 | 6.58 | 0 | 0 | 8 | 0 | 0 | 0 |
| 2/26/2001 | 49.27 | 6.73 | 0 | 0 | 20 | 0 | 0 | 0 |
| 2/27/2001 | 47.57 | 6.20 | 0 | 0 | 8 | 0 | 0 | 0 |
| 2/28/2001 | 46.16 | 5.92 | 0 | 0 | 2 | 0 | 0 | 0 |
| 3/1/2001 | 45.02 | 5.35 | 0 | 0 | 0 | 0 | 0 | 0 |
| 3/2/2001 | 53.52 | 6.21 | 0 | 0 | 61 | 0 | 0 | 0 |
| 3/3/2001 | 63.43 | 5.84 | 0 | 0 | 227 | 0 | 0 | 0 |
| 3/4/2001 | 60.88 | 5.67 | 0 | 0 | 6 | 0 | 0 | 0 |

| Date | Discharge (cms) | Temp (C°) | Chinook | Coho | Steelhead | Cutthroat | Lamprey | Sucker |
|---|---|---|---|---|---|---|---|---|
| 3/5/2001 | 58.90 | 6.30 | 0 | 0 | 145 | 0 | 0 | 0 |
| 3/6/2001 | 58.33 | 6.82 | 0 | 0 | 158 | 0 | 0 | 0 |
| 3/7/2001 | 57.20 | 8.00 | 0 | 0 | 475 | 0 | 0 | 0 |
| 3/8/2001 | 55.78 | 8.49 | 0 | 0 | 744 | 0 | 0 | 8 |
| 3/9/2001 | 57.20 | 8.21 | 0 | 0 | 215 | 1 | 0 | 0 |
| 3/10/2001 | 56.92 | 7.77 | 0 | 0 | 267 | 0 | 0 | 0 |
| 3/11/2001 | 54.65 | 7.89 | 0 | 0 | 50 | 0 | 0 | 0 |
| 3/12/2001 | 52.39 | 8.10 | 0 | 0 | 116 | 0 | 0 | 0 |
| 3/13/2001 | 50.40 | 8.43 | 0 | 0 | 221 | 1 | 0 | 0 |
| 3/14/2001 | 48.99 | 8.43 | 0 | 0 | 68 | 0 | 0 | 0 |
| 3/15/2001 | 47.86 | 7.90 | 0 | 0 | 170 | 0 | 0 | 0 |
| 3/16/2001 | 50.97 | 7.12 | 0 | 0 | 22 | 0 | 0 | 0 |
| 3/17/2001 | 54.37 | 7.13 | 0 | 0 | 122 | 0 | 0 | 0 |
| 3/18/2001 | 87.22 | 7.71 | 0 | 0 | 524 | 1 | 0 | 0 |
| 3/19/2001 | 117.80 | 8.51 | 0 | 0 | 628 | 1 | 0 | 3 |
| 3/20/2001 | 113.83 | 9.11 | 0 | 0 | 318 | 0 | 0 | 0 |
| 3/21/2001 | 94.86 | 9.16 | 0 | 0 | 134 | 0 | 0 | 0 |
| 3/22/2001 | 80.14 | 10.11 | 0 | 0 | 130 | 0 | 0 | 51 |
| 3/23/2001 | 69.38 | 10.89 | 0 | 0 | 161 | 0 | 1 | 256 |
| 3/24/2001 | 67.11 | 10.80 | 0 | 0 | 161 | 0 | 0 | 130 |
| 3/25/2001 | 75.04 | 10.53 | 3 | 0 | 123 | 0 | 0 | 14 |
| 3/26/2001 | 80.42 | 9.94 | 0 | 0 | 66 | 0 | 0 | 0 |
| 3/27/2001 | 78.72 | 8.77 | 0 | 0 | 17 | 0 | 0 | 0 |
| 3/28/2001 | 222.00 | 8.45 | 0 | 0 | 31 | 0 | 0 | 0 |
| 3/29/2001 | 189.44 | 10.10 | 0 | 0 | 145 | 0 | 0 | 1 |
| 3/30/2001 | 138.75 | 10.38 | 0 | 0 | 218 | 0 | 0 | 6 |
| 3/31/2001 | 110.15 | 9.92 | 0 | 0 | 36 | 0 | 0 | 0 |
| 4/1/2001 | 95.71 | 9.75 | 0 | 0 | 31 | 0 | 0 | 0 |
| 4/2/2001 | 88.91 | 9.32 | 0 | 0 | 18 | 0 | 0 | 0 |
| 4/3/2001 | 84.38 | 8.15 | 0 | 0 | 2 | 0 | 0 | 0 |
| 4/4/2001 | 79.00 | 8.19 | 0 | 0 | 4 | 0 | 0 | 0 |
| 4/5/2001 | 77.30 | 8.18 | 1 | 0 | 1 | 0 | 0 | 0 |
| 4/6/2001 | 82.97 | 7.93 | 2 | 0 | 4 | 0 | 0 | 0 |
| 4/7/2001 | 99.11 | 7.85 | 2 | 0 | 7 | 0 | 0 | 0 |
| 4/8/2001 | 94.86 | 7.85 | 0 | 0 | 6 | 0 | 0 | 0 |
| 4/9/2001 | 91.75 | 7.53 | 1 | 0 | 3 | 0 | 0 | 0 |
| 4/10/2001 | 85.80 | 7.83 | 3 | 0 | 10 | 0 | 0 | 0 |
| 4/11/2001 | 99.96 | 8.58 | 1 | 0 | 23 | 0 | 0 | 0 |
| 4/12/2001 | 120.06 | 7.48 | 0 | 0 | 8 | 0 | 0 | 0 |
| 4/13/2001 | 105.91 | 8.05 | 1 | 0 | 11 | 0 | 0 | 0 |
| 4/14/2001 | 90.61 | 8.75 | 0 | 0 | 9 | 0 | 0 | 0 |
| 4/15/2001 | 81.55 | 8.80 | 5 | 0 | 12 | 0 | 0 | 0 |
| 4/16/2001 | 78.15 | 9.91 | 4 | 0 | 89 | 0 | 0 | 96 |
| 4/17/2001 | 85.52 | 10.82 | 9 | 0 | 89 | 0 | 0 | 151 |
| 4/18/2001 | 94.01 | 10.51 | 44 | 0 | 114 | 0 | 0 | 493 |

| Date | Discharge (cms) | Temp (C°) | Chinook | Coho | Steelhead | Cutthroat | Lamprey | Sucker |
|---|---|---|---|---|---|---|---|---|
| 4/19/2001 | 106.47 | 10.31 | 19 | 0 | 36 | 0 | 0 | 84 |
| 4/20/2001 | 107.60 | 10.17 | 3 | 0 | 15 | 0 | 0 | 0 |
| 4/21/2001 | 99.96 | 10.26 | 13 | 0 | 12 | 0 | 0 | 1 |
| 4/22/2001 | 89.48 | 9.92 | 9 | 0 | 6 | 0 | 0 | 0 |
| 4/23/2001 | 81.84 | 10.32 | 2 | 0 | 0 | 0 | 0 | 0 |
| 4/24/2001 | 76.17 | 12.28 | 15 | 0 | 49 | 0 | 0 | 1215 |
| 4/25/2001 | 74.47 | 13.80 | 108 | 0 | 25 | 0 | 1 | 1127 |
| 4/26/2001 | 76.46 | 14.56 | 77 | 0 | 27 | 0 | 0 | 1087 |
| 4/27/2001 | 74.47 | 13.81 | 112 | 0 | 16 | 0 | 1 | 433 |
| 4/28/2001 | 74.47 | 12.89 | 24 | 0 | 6 | 0 | 0 | 200 |
| 4/29/2001 | 75.32 | 11.68 | 9 | 0 | 2 | 0 | 0 | 2 |
| 4/30/2001 | 69.09 | 10.85 | 6 | 0 | 2 | 0 | 0 | 0 |
| 5/1/2001 | 94.01 | 10.29 | 0 | 0 | 0 | 0 | 0 | 0 |
| 5/2/2001 | 95.99 | 9.94 | 0 | 0 | 0 | 0 | 0 | 0 |
| 5/3/2001 | 77.87 | 11.10 | 18 | 0 | 7 | 0 | 0 | 3 |
| 5/4/2001 | 70.51 | 12.07 | 160 | 0 | 11 | 0 | 0 | 249 |
| 5/5/2001 | 66.54 | 12.90 | 44 | 0 | 12 | 0 | 1 | 63 |
| 5/6/2001 | 62.30 | 13.48 | 113 | 0 | 7 | 1 | 0 | 396 |
| 5/7/2001 | 60.60 | 13.64 | 237 | 0 | 14 | 1 | 0 | 260 |
| 5/8/2001 | 58.33 | 14.55 | 17 | 0 | 6 | 0 | 0 | 531 |
| 5/9/2001 | 56.92 | 14.83 | 0 | 0 | 0 | 0 | 0 | 0 |
| 5/10/2001 | 54.93 | 14.87 | 253 | 0 | 9 | 1 | 0 | 48 |
| 5/11/2001 | 52.67 | 15.34 | 180 | 0 | 5 | 0 | 2 | 106 |
| 5/12/2001 | 49.55 | 16.34 | 160 | 0 | 4 | 1 | 1 | 179 |
| 5/13/2001 | 48.70 | 16.35 | 79 | 0 | 1 | 0 | 1 | 92 |
| 5/14/2001 | 49.27 | 16.28 | 23 | 0 | 0 | 0 | 1 | 1 |
| 5/15/2001 | 94.86 | 13.96 | 9 | 0 | 2 | 0 | 0 | 215 |
| 5/16/2001 | 233.61 | 11.26 | 0 | 0 | 0 | 0 | 0 | 0 |
| 5/17/2001 | 174.71 | 11.57 | 37 | 0 | 1 | 0 | 3 | 0 |
| 5/18/2001 | 125.73 | 12.63 | 34 | 0 | 6 | 0 | 0 | 4 |
| 5/19/2001 | 101.66 | 13.61 | 113 | 0 | 10 | 0 | 1 | 201 |
| 5/20/2001 | 85.80 | 14.74 | 1 | 0 | 0 | 0 | 1 | 24 |
| 5/21/2001 | 74.76 | 14.43 | 35 | 0 | 4 | 0 | 0 | 1069 |
| 5/22/2001 | 68.81 | 16.40 | 86 | 0 | 2 | 0 | 0 | 125 |
| 5/23/2001 | 61.73 | 17.90 | 341 | 0 | 13 | 0 | 4 | 5 |
| 5/24/2001 | 57.20 | 18.26 | 298 | 0 | 15 | 2 | 0 | 6 |
| 5/25/2001 | 54.37 | 18.70 | 291 | 0 | 38 | 0 | 0 | 29 |
| 5/26/2001 | 51.82 | 18.45 | 133 | 0 | 21 | 0 | 0 | 4 |
| 5/27/2001 | 43.32 | 18.18 | 285 | 0 | 32 | 3 | 1 | 8 |
| 5/28/2001 | 41.34 | 17.15 | 197 | 0 | 22 | 0 | 0 | 1 |
| 5/29/2001 | 42.19 | 16.43 | 127 | 0 | 24 | 0 | 0 | 0 |
| 5/30/2001 | 41.91 | 16.87 | 108 | 0 | 12 | 0 | 0 | 0 |
| 5/31/2001 | 40.78 | 17.80 | 157 | 0 | 37 | 2 | 0 | 18 |
| 6/1/2001 | 39.08 | 18.27 | 138 | 0 | 35 | 1 | 0 | 28 |
| 6/2/2001 | 39.08 | 16.09 | 99 | 0 | 24 | 1 | 0 | 0 |

| Date | Discharge (cms) | Temp (C°) | Chinook | Coho | Steelhead | Cutthroat | Lamprey | Sucker |
|---|---|---|---|---|---|---|---|---|
| 6/3/2001 | 39.64 | 15.47 | 139 | 0 | 14 | 0 | 0 | 0 |
| 6/4/2001 | 39.93 | 15.35 | 137 | 0 | 21 | 0 | 0 | 0 |
| 6/5/2001 | 39.36 | 14.68 | 132 | 0 | 30 | 0 | 0 | 1 |
| 6/6/2001 | 41.06 | 15.02 | 157 | 0 | 37 | 0 | 0 | 0 |
| 6/7/2001 | 39.08 | 16.69 | 146 | 0 | 58 | 0 | 0 | 8 |
| 6/8/2001 | 37.10 | 18.14 | 85 | 0 | 42 | 1 | 0 | 90 |
| 6/9/2001 | 35.96 | 18.70 | 36 | 0 | 18 | 0 | 1 | 7 |
| 6/10/2001 | 35.11 | 17.66 | 63 | 0 | 34 | 0 | 1 | 0 |
| 6/11/2001 | 34.83 | 16.92 | 71 | 0 | 62 | 0 | 1 | 1 |
| 6/12/2001 | 35.68 | 16.25 | 39 | 0 | 65 | 0 | 0 | 0 |
| 6/13/2001 | 36.53 | 16.69 | 32 | 0 | 61 | 0 | 0 | 0 |
| 6/14/2001 | 34.26 | 17.30 | 83 | 0 | 116 | 0 | 0 | 10 |
| 6/15/2001 | 33.41 | 17.20 | 56 | 0 | 70 | 0 | 0 | 6 |
| 6/16/2001 | 32.85 | 17.31 | 38 | 0 | 74 | 0 | 1 | 0 |
| 6/17/2001 | 32.00 | 17.47 | 45 | 0 | 49 | 0 | 0 | 0 |
| 6/18/2001 | 30.87 | 18.50 | 0 | 0 | 0 | 0 | 0 | 0 |
| 6/19/2001 | 30.30 | 18.11 | 17 | 0 | 43 | 0 | 0 | 14 |
| 6/20/2001 | 29.17 | 19.33 | 47 | 0 | 79 | 1 | 0 | 3 |
| 6/21/2001 | 28.60 | 20.15 | 77 | 0 | 218 | 1 | 0 | 19 |
| 6/22/2001 | 28.18 | 20.56 | 90 | 0 | 159 | 1 | 2 | 33 |
| 6/23/2001 | 28.29 | 20.45 | 41 | 0 | 94 | 0 | 3 | 5 |
| 6/24/2001 | 28.23 | 18.77 | 59 | 0 | 103 | 0 | 0 | 0 |
| 6/25/2001 | 29.17 | 17.13 | 7 | 0 | 14 | 0 | 0 | 0 |
| 6/26/2001 | 29.73 | 17.41 | 36 | 0 | 3 | 0 | 0 | 0 |
| 6/27/2001 | 30.87 | 17.12 | 0 | 0 | 0 | 0 | 0 | 0 |
| 6/28/2001 | 32.28 | 17.92 | 46 | 0 | 156 | 0 | 1 | 0 |
| 6/29/2001 | 31.15 | 17.88 | 52 | 0 | 158 | 0 | 0 | 0 |
| 6/30/2001 | 30.87 | 18.14 | 33 | 0 | 94 | 0 | 0 | 0 |
| 7/1/2001 | 30.30 | 18.40 | 13 | 0 | 43 | 1 | 0 | 0 |
| 7/2/2001 | 28.88 | 19.74 | 23 | 0 | 86 | 1 | 0 | 0 |
| 7/3/2001 | 28.32 | 20.69 | 40 | 0 | 176 | 0 | 0 | 1 |
| 7/4/2001 | 27.58 | 21.69 | 57 | 0 | 139 | 0 | 0 | 0 |
| 7/5/2001 | 26.73 | 21.63 | 71 | 0 | 162 | 0 | 0 | 0 |
| 7/6/2001 | 25.97 | 20.71 | 24 | 0 | 51 | 1 | 1 | 0 |
| 7/7/2001 | 25.06 | 21.12 | 16 | 0 | 50 | 0 | 0 | 0 |
| 7/8/2001 | 24.78 | 21.48 | 9 | 0 | 63 | 3 | 0 | 0 |
| 7/9/2001 | 24.44 | 21.95 | 11 | 0 | 13 | 1 | 0 | 1 |
| 7/10/2001 | 24.30 | 22.69 | 0 | 0 | 0 | 0 | 0 | 0 |
| 7/11/2001 | 24.66 | 21.64 | 22 | 0 | 3 | 1 | 0 | 0 |
| 7/12/2001 | 26.96 | 21.81 | 15 | 0 | 84 | 0 | 0 | 0 |
| 7/13/2001 | 26.65 | 22.28 | 5 | 0 | 22 | 0 | 0 | 0 |
| 7/14/2001 | 24.83 | 21.82 | 4 | 0 | 30 | 1 | 0 | 0 |
| 7/15/2001 | 24.38 | 21.08 | 3 | 0 | 17 | 1 | 0 | 0 |
| 7/16/2001 | 24.24 | 20.19 | 4 | 0 | 12 | 0 | 0 | 0 |
| 7/17/2001 | 24.35 | 19.96 | 4 | 0 | 4 | 0 | 0 | 0 |

| Date | Discharge (cms) | Temp (C°) | Chinook | Coho | Steelhead | Cutthroat | Lamprey | Sucker |
|---|---|---|---|---|---|---|---|---|
| 7/18/2001 | 24.24 | 19.95 | 33 | 0 | 19 | 0 | 1 | 0 |
| 7/19/2001 | 24.13 | 20.54 | 49 | 0 | 62 | 0 | 0 | 0 |
| 7/20/2001 | 24.13 | 20.24 | 48 | 0 | 128 | 0 | 0 | 0 |
| 7/21/2001 | 24.18 | 19.45 | 50 | 0 | 154 | 1 | 0 | 0 |
| 7/22/2001 | 24.18 | 20.25 | 32 | 0 | 151 | 0 | 0 | 0 |
| 7/23/2001 | 23.73 | 21.27 | 18 | 0 | 103 | 0 | 1 | 0 |
| 7/24/2001 | 23.16 | 22.07 | 27 | 0 | 113 | 0 | 0 | 0 |
| 7/25/2001 | 22.63 | 22.38 | 16 | 0 | 87 | 1 | 0 | 0 |
| 7/26/2001 | 22.12 | 22.38 | 13 | 0 | 49 | 2 | 0 | 0 |
| 7/27/2001 | 21.69 | 22.09 | 17 | 0 | 29 | 2 | 0 | 0 |
| 7/28/2001 | 21.52 | 21.85 | 9 | 0 | 26 | 1 | 1 | 0 |
| 7/29/2001 | 21.61 | 21.23 | 5 | 0 | 19 | 0 | 0 | 0 |
| 7/30/2001 | 22.74 | 19.69 | 5 | 0 | 9 | 1 | 0 | 0 |
| 7/31/2001 | 23.56 | 19.71 | 3 | 0 | 9 | 0 | 0 | 0 |
| 8/1/2001 | 23.11 | 20.18 | 4 | 0 | 7 | 1 | 0 | 0 |
| 8/2/2001 | 22.23 | 20.79 | 5 | 0 | 10 | 0 | 0 | 0 |
| 8/3/2001 | 21.78 | 21.33 | 15 | 0 | 14 | 1 | 0 | 0 |
| 8/4/2001 | 21.66 | 21.13 | 17 | 0 | 28 | 1 | 0 | 0 |
| 8/5/2001 | 21.61 | 20.80 | 8 | 0 | 8 | 1 | 0 | 0 |
| 8/6/2001 | 21.32 | 21.60 | 0 | 0 | 0 | 0 | 0 | 0 |
| 8/7/2001 | 21.10 | 21.63 | 11 | 0 | 0 | 0 | 0 | 0 |
| 8/8/2001 | 21.10 | 21.74 | 20 | 0 | 59 | 0 | 0 | 0 |
| 8/9/2001 | 21.01 | 22.35 | 17 | 0 | 26 | 3 | 0 | 0 |
| 8/10/2001 | 20.33 | 23.21 | 10 | 0 | 14 | 3 | 0 | 0 |
| 8/11/2001 | 20.16 | 23.19 | 14 | 0 | 14 | 2 | 0 | 0 |
| 8/12/2001 | 19.96 | 23.26 | 6 | 0 | 10 | 2 | 0 | 0 |
| 8/13/2001 | 19.68 | 22.95 | 3 | 0 | 9 | 1 | 0 | 0 |
| 8/14/2001 | 19.82 | 22.25 | 4 | 0 | 2 | 0 | 1 | 0 |
| 8/15/2001 | 20.19 | 21.77 | 3 | 0 | 2 | 2 | 0 | 0 |
| 8/16/2001 | 20.30 | 21.33 | 1 | 0 | 0 | 0 | 0 | 0 |
| 8/17/2001 | 20.39 | 21.25 | 2 | 0 | 0 | 0 | 0 | 0 |
| 8/18/2001 | 20.33 | 21.26 | 11 | 0 | 0 | 0 | 0 | 0 |
| 8/19/2001 | 20.19 | 20.40 | 25 | 0 | 2 | 0 | 0 | 0 |
| 8/20/2001 | 20.33 | 20.63 | 32 | 0 | 4 | 0 | 0 | 0 |
| 8/21/2001 | 20.39 | 19.96 | 34 | 0 | 12 | 0 | 0 | 0 |
| 8/22/2001 | 20.76 | 19.50 | 29 | 0 | 24 | 1 | 0 | 0 |
| 8/23/2001 | 21.29 | 18.84 | 15 | 0 | 20 | 1 | 0 | 0 |
| 8/24/2001 | 21.38 | 19.20 | 15 | 0 | 21 | 0 | 0 | 0 |
| 8/25/2001 | 20.67 | 19.71 | 26 | 0 | 25 | 2 | 0 | 0 |
| 8/26/2001 | 19.94 | 19.96 | 19 | 0 | 24 | 1 | 0 | 0 |
| 8/27/2001 | 19.43 | 20.23 | 26 | 0 | 20 | 3 | 0 | 0 |
| 8/28/2001 | 19.26 | 20.49 | 20 | 0 | 37 | 2 | 0 | 0 |
| 8/29/2001 | 19.17 | 20.73 | 24 | 0 | 33 | 6 | 0 | 0 |
| 8/30/2001 | 19.11 | 20.80 | 17 | 0 | 21 | 3 | 0 | 0 |
| 8/31/2001 | 19.00 | 20.59 | 19 | 0 | 25 | 3 | 0 | 0 |

| Date | Discharge (cms) | Temp (C°) | Chinook | Coho | Steelhead | Cutthroat | Lamprey | Sucker |
|---|---|---|---|---|---|---|---|---|
| 9/1/2001 | 18.80 | 20.68 | 23 | 1 | 24 | 1 | 0 | 0 |
| 9/2/2001 | 18.77 | 20.93 | 14 | 0 | 24 | 2 | 0 | 0 |
| 9/3/2001 | 18.58 | 20.69 | 11 | 0 | 12 | 0 | 0 | 0 |
| 9/4/2001 | 18.49 | 20.57 | 5 | 0 | 5 | 0 | 0 | 0 |
| 9/5/2001 | 18.18 | 19.53 | 0 | 0 | 0 | 0 | 0 | 0 |
| 9/6/2001 | 18.07 | 18.15 | 0 | 0 | 0 | 0 | 0 | 0 |
| 9/7/2001 | 18.01 | 17.76 | 0 | 0 | 0 | 0 | 0 | 0 |
| 9/8/2001 | 18.04 | 17.86 | 0 | 0 | 0 | 0 | 0 | 0 |
| 9/9/2001 | 18.01 | 18.06 | 0 | 0 | 0 | 0 | 0 | 0 |
| 9/10/2001 | 18.15 | 18.38 | 0 | 0 | 0 | 0 | 0 | 0 |
| 9/11/2001 | 18.97 | 18.46 | 0 | 0 | 0 | 0 | 0 | 0 |
| 9/12/2001 | 19.26 | 18.39 | 0 | 0 | 0 | 0 | 0 | 0 |
| 9/13/2001 | 18.97 | 17.58 | 7 | 0 | 8 | 0 | 0 | 0 |
| 9/14/2001 | 19.45 | 19.10 | 15 | 1 | 13 | 1 | 0 | 0 |
| 9/15/2001 | 19.37 | 19.50 | 17 | 5 | 14 | 3 | 0 | 0 |
| 9/16/2001 | 19.91 | 19.74 | 16 | 4 | 21 | 3 | 0 | 0 |
| 9/17/2001 | 20.08 | 19.36 | 30 | 4 | 21 | 2 | 0 | 0 |
| 9/18/2001 | 19.74 | 18.41 | 4 | 4 | 6 | 0 | 0 | 0 |
| 9/19/2001 | 19.60 | 17.64 | 6 | 3 | 5 | 0 | 0 | 0 |
| 9/20/2001 | 19.54 | 16.92 | 3 | 0 | 4 | 0 | 0 | 0 |
| 9/21/2001 | 19.62 | 16.71 | 4 | 1 | 4 | 0 | 0 | 0 |
| 9/22/2001 | 19.57 | 16.42 | 4 | 4 | 5 | 0 | 0 | 0 |
| 9/23/2001 | 19.54 | 16.63 | 8 | 6 | 8 | 0 | 0 | 0 |
| 9/24/2001 | 19.54 | 16.53 | 5 | 4 | 11 | 1 | 0 | 0 |
| 9/25/2001 | 20.84 | 16.05 | 18 | 8 | 16 | 4 | 0 | 0 |
| 9/26/2001 | 21.89 | 15.15 | 9 | 18 | 16 | 0 | 0 | 0 |
| 9/27/2001 | 23.87 | 14.53 | 7 | 11 | 5 | 0 | 0 | 0 |
| 9/28/2001 | 23.53 | 14.23 | 4 | 14 | 4 | 1 | 0 | 0 |
| 9/29/2001 | 22.20 | 14.33 | 2 | 18 | 3 | 0 | 0 | 0 |
| 9/30/2001 | 21.69 | 14.76 | 0 | 0 | 0 | 0 | 0 | 0 |
| 10/1/2001 | 21.41 | 14.95 | 0 | 0 | 0 | 0 | 0 | 0 |
| 10/2/2001 | 21.29 | 15.11 | 2 | 11 | 0 | 1 | 0 | 0 |
| 10/3/2001 | 21.07 | 14.72 | 7 | 61 | 30 | 3 | 0 | 0 |
| 10/4/2001 | 21.07 | 14.68 | 7 | 35 | 6 | 0 | 0 | 0 |
| 10/5/2001 | 20.22 | 14.33 | 8 | 30 | 12 | 1 | 0 | 0 |
| 10/6/2001 | 20.39 | 13.93 | 9 | 21 | 7 | 2 | 0 | 0 |
| 10/7/2001 | 20.47 | 13.37 | 1 | 5 | 0 | 0 | 0 | 0 |
| 10/8/2001 | 20.61 | 12.18 | 0 | 5 | 0 | 0 | 0 | 0 |
| 10/9/2001 | 20.84 | 11.94 | 0 | 0 | 0 | 0 | 0 | 0 |
| 10/10/2001 | 20.90 | 11.51 | 1 | 5 | 2 | 0 | 0 | 0 |
| 10/11/2001 | 21.95 | 11.65 | 6 | 19 | 1 | 0 | 0 | 0 |
| 10/12/2001 | 28.03 | 11.62 | 9 | 22 | 2 | 0 | 0 | 0 |
| 10/13/2001 | 24.66 | 12.20 | 18 | 33 | 22 | 0 | 0 | 0 |
| 10/14/2001 | 23.28 | 12.28 | 9 | 21 | 3 | 0 | 0 | 0 |
| 10/15/2001 | 22.37 | 12.23 | 7 | 21 | 0 | 0 | 0 | 0 |

| Date | Discharge (cms) | Temp (C°) | Chinook | Coho | Steelhead | Cutthroat | Lamprey | Sucker |
|---|---|---|---|---|---|---|---|---|
| 10/16/2001 | 23.11 | 12.22 | 3 | 89 | 8 | 0 | 0 | 0 |
| 10/17/2001 | 23.87 | 11.68 | 11 | 78 | 10 | 1 | 0 | 0 |
| 10/18/2001 | 23.84 | 11.20 | 7 | 23 | 0 | 0 | 0 | 0 |
| 10/19/2001 | 23.93 | 10.90 | 2 | 27 | 4 | 1 | 0 | 0 |
| 10/20/2001 | 23.02 | 10.57 | 3 | 55 | 7 | 0 | 0 | 0 |
| 10/21/2001 | 23.05 | 10.16 | 4 | 8 | 0 | 0 | 0 | 0 |
| 10/22/2001 | 23.33 | 10.61 | 1 | 0 | 0 | 0 | 0 | 0 |
| 10/23/2001 | 47.01 | 10.57 | 24 | 154 | 14 | 0 | 0 | 0 |
| 10/24/2001 | 45.87 | 9.67 | 45 | 972 | 93 | 1 | 0 | 2 |
| 10/25/2001 | 32.28 | 9.91 | 24 | 366 | 49 | 1 | 0 | 1 |
| 10/26/2001 | 28.23 | 9.97 | 9 | 269 | 32 | 0 | 0 | 0 |
| 10/27/2001 | 26.76 | 9.72 | 5 | 128 | 15 | 0 | 0 | 0 |
| 10/28/2001 | 26.22 | 9.09 | 0 | 13 | 0 | 0 | 0 | 0 |
| 10/29/2001 | 26.59 | 9.10 | 0 | 0 | 0 | 0 | 0 | 0 |
| 10/30/2001 | 28.20 | 9.57 | 0 | 20 | 20 | 0 | 0 | 0 |
| 10/31/2001 | 51.25 | 10.04 | 1 | 259 | 12 | 0 | 0 | 0 |
| 11/1/2001 | 48.14 | 10.56 | 3 | 256 | 37 | 1 | 0 | 7 |
| 11/2/2001 | 40.78 | 11.37 | 4 | 802 | 33 | 0 | 0 | 0 |
| 11/3/2001 | 34.55 | 11.29 | 1 | 918 | 22 | 0 | 0 | 0 |
| 11/4/2001 | 31.43 | 10.51 | 0 | 139 | 8 | 0 | 0 | 0 |
| 11/5/2001 | 30.02 | 9.90 | 0 | 67 | 2 | 0 | 0 | 0 |
| 11/6/2001 | 29.45 | 9.19 | 0 | 28 | 6 | 0 | 0 | 0 |
| 11/7/2001 | 28.60 | 8.40 | 0 | 6 | 0 | 0 | 0 | 0 |
| 11/8/2001 | 28.20 | 7.41 | 0 | 2 | 1 | 0 | 0 | 0 |
| 11/9/2001 | 27.81 | 6.77 | 0 | 3 | 0 | 0 | 0 | 0 |
| 11/10/2001 | 27.64 | 6.90 | 0 | 8 | 0 | 0 | 0 | 0 |
| 11/11/2001 | 28.88 | 7.35 | 0 | 2 | 0 | 0 | 0 | 0 |
| 11/12/2001 | 29.17 | 8.28 | 0 | 0 | 0 | 0 | 0 | 0 |
| 11/13/2001 | 31.15 | 8.38 | 0 | 2 | 0 | 0 | 0 | 0 |
| 11/14/2001 | 37.94 | 8.93 | 0 | 33 | 0 | 0 | 0 | 0 |
| 11/15/2001 | 43.04 | 9.68 | 1 | 123 | 10 | 1 | 0 | 0 |
| 11/16/2001 | 43.61 | 9.75 | 1 | 495 | 37 | 1 | 0 | 0 |
| 11/17/2001 | 83.25 | 9.36 | 1 | 289 | 21 | 0 | 0 | 0 |
| 11/18/2001 | 58.33 | 8.64 | 0 | 86 | 16 | 0 | 0 | 0 |
| 11/19/2001 | 43.32 | 8.36 | 0 | 12 | 0 | 0 | 0 | 0 |
| 11/20/2001 | 40.21 | 8.48 | 0 | 9 | 2 | 0 | 0 | 0 |
| 11/21/2001 | 64.56 | 8.13 | 1 | 107 | 28 | 0 | 0 | 0 |
| 11/22/2001 | 187.17 | 8.42 | 0 | 72 | 11 | 0 | 0 | 0 |
| 11/23/2001 | 244.37 | 8.61 | 1 | 48 | 4 | 0 | 0 | 0 |
| 11/24/2001 | 112.70 | | 0 | 48 | 10 | 0 | 0 | 0 |
| 11/25/2001 | 117.23 | | 0 | 4 | 0 | 0 | 0 | 0 |
| 11/26/2001 | 127.14 | | 0 | 0 | 0 | 0 | 0 | 0 |
| 11/27/2001 | 95.71 | | 0 | 0 | 0 | 0 | 0 | 0 |
| 11/28/2001 | 118.65 | | 0 | 11 | 4 | 0 | 0 | 0 |
| 11/29/2001 | 317.15 | | 0 | 0 | 0 | 0 | 0 | 0 |

| Date | Discharge (cms) | Temp (C°) | Chinook | Coho | Steelhead | Cutthroat | Lamprey | Sucker |
|---|---|---|---|---|---|---|---|---|
| 11/30/2001 | 182.93 | | 0 | 63 | 12 | 0 | 0 | 0 |
| 12/1/2001 | 169.62 | | 0 | 7 | 13 | 0 | 0 | 0 |
| 12/2/2001 | 216.91 | | 0 | 3 | 2 | 0 | 0 | 0 |
| 12/3/2001 | 150.36 | | 0 | 4 | 10 | 0 | 0 | 0 |
| 12/4/2001 | 127.14 | | 0 | 0 | 0 | 0 | 0 | 0 |
| 12/5/2001 | 192.27 | | 0 | 0 | 0 | 0 | 0 | 0 |
| 12/6/2001 | 274.39 | | 0 | 25 | 5 | 0 | 0 | 0 |
| 12/7/2001 | 353.96 | 7.22 | 0 | 4 | 1 | 0 | 0 | 0 |
| 12/8/2001 | 196.24 | 6.66 | 0 | 1 | 4 | 0 | 0 | 0 |
| 12/9/2001 | 163.67 | 6.60 | 0 | 0 | 3 | 0 | 0 | 0 |
| 12/10/2001 | 139.04 | 5.98 | 0 | 0 | 0 | 0 | 0 | 0 |
| 12/11/2001 | 128.28 | 5.63 | 0 | 0 | 0 | 0 | 0 | 0 |
| 12/12/2001 | 145.27 | 5.76 | 0 | 0 | 0 | 0 | 0 | 0 |
| 12/13/2001 | 178.96 | 6.39 | 0 | 0 | 0 | 0 | 0 | 0 |
| 12/14/2001 | 719.25 | 7.14 | 0 | 0 | 0 | 0 | 0 | 0 |
| 12/15/2001 | 348.30 | 6.58 | 0 | 0 | 0 | 0 | 0 | 0 |
| 12/16/2001 | 336.97 | 6.85 | 0 | 0 | 0 | 0 | 0 | 0 |
| 12/17/2001 | 546.52 | 7.50 | 0 | 0 | 0 | 0 | 0 | 0 |
| 12/18/2001 | 356.79 | 6.92 | 0 | 1 | 11 | 0 | 0 | 0 |
| 12/19/2001 | 256.83 | 6.77 | 0 | 0 | 0 | 0 | 0 | 0 |
| 12/20/2001 | 214.92 | 6.74 | 0 | 0 | 0 | 0 | 0 | 0 |
| 12/21/2001 | 179.81 | 6.36 | 0 | 1 | 36 | 0 | 0 | 0 |
| 12/22/2001 | 148.95 | 5.70 | 0 | 0 | 7 | 0 | 0 | 0 |
| 12/23/2001 | 125.16 | 5.78 | 0 | 0 | 20 | 0 | 0 | 0 |
| 12/24/2001 | 104.21 | 4.80 | 0 | 1 | 7 | 0 | 0 | 0 |
| 12/25/2001 | 92.03 | 4.47 | 0 | 0 | 2 | 0 | 0 | 0 |
| 12/26/2001 | 86.37 | 4.51 | 0 | 0 | 6 | 0 | 0 | 1 |
| 12/27/2001 | 82.97 | 4.77 | 0 | 0 | 14 | 0 | 0 | 0 |
| 12/28/2001 | 108.74 | 5.74 | 0 | 2 | 90 | 0 | 0 | 0 |
| 12/29/2001 | 127.99 | 6.26 | 0 | 0 | 113 | 0 | 0 | 0 |
| 12/30/2001 | 138.47 | 6.73 | 0 | 0 | 103 | 0 | 0 | 0 |
| 12/31/2001 | 193.97 | 7.18 | 0 | 0 | 106 | 0 | 0 | 0 |
| 1/1/2002 | 207.56 | 7.40 | 0 | 1 | 116 | 0 | 0 | 0 |
| 1/2/2002 | 229.37 | 7.89 | 0 | 0 | 105 | 0 | 0 | 0 |
| 1/3/2002 | 225.40 | 7.28 | 0 | 0 | 57 | 0 | 0 | 1 |
| 1/4/2002 | 175.00 | 6.68 | 0 | 0 | 50 | 0 | 0 | 0 |
| 1/5/2002 | 141.30 | 6.29 | 0 | 0 | 40 | 0 | 0 | 0 |
| 1/6/2002 | 211.81 | 7.50 | 0 | 0 | 74 | 0 | 0 | 0 |
| 1/7/2002 | 308.65 | 8.20 | 0 | 0 | 79 | 0 | 0 | 0 |
| 1/8/2002 | 441.74 | 8.19 | 0 | 0 | 73 | 0 | 0 | 0 |
| 1/9/2002 | 356.79 | 7.68 | 0 | 0 | 19 | 0 | 0 | 0 |
| 1/10/2002 | 231.35 | 6.80 | 0 | 1 | 9 | 0 | 0 | 0 |
| 1/11/2002 | 173.58 | 6.69 | 0 | 1 | 54 | 0 | 0 | 0 |
| 1/12/2002 | 148.38 | 7.14 | 0 | 0 | 133 | 0 | 0 | 0 |
| 1/13/2002 | 137.05 | 6.84 | 0 | 2 | 61 | 0 | 0 | 0 |

| Date | Discharge (cms) | Temp (C°) | Chinook | Coho | Steelhead | Cutthroat | Lamprey | Sucker |
|---|---|---|---|---|---|---|---|---|
| 1/14/2002 | 125.16 | 6.08 | 0 | 0 | 9 | 0 | 0 | 0 |
| 1/15/2002 | 112.98 | 5.31 | 0 | 0 | 1 | 0 | 0 | 0 |
| 1/16/2002 | 97.98 | 4.67 | 0 | 0 | 1 | 0 | 0 | 0 |
| 1/17/2002 | 93.45 | 4.40 | 0 | 0 | 0 | 0 | 0 | 0 |
| 1/18/2002 | 88.07 | 4.40 | 0 | 0 | 0 | 0 | 0 | 0 |
| 1/19/2002 | 91.18 | 4.96 | 0 | 0 | 2 | 0 | 0 | 0 |
| 1/20/2002 | 101.94 | 4.93 | 0 | 0 | 4 | 0 | 0 | 0 |
| 1/21/2002 | 209.83 | 4.81 | 0 | 0 | 4 | 0 | 0 | 0 |
| 1/22/2002 | 202.18 | 4.84 | 0 | 0 | 0 | 0 | 0 | 0 |
| 1/23/2002 | 151.78 | 5.15 | 0 | 0 | 13 | 0 | 0 | 0 |
| 1/24/2002 | 124.88 | 5.06 | 0 | 0 | 10 | 0 | 0 | 0 |
| 1/25/2002 | 175.56 | 5.71 | 0 | 0 | 48 | 0 | 0 | 0 |
| 1/26/2002 | 410.59 | 6.11 | 0 | 0 | 18 | 0 | 0 | 0 |
| 1/27/2002 | 238.43 | 5.53 | 0 | 0 | 4 | 0 | 0 | 0 |
| 1/28/2002 | 169.33 | 5.23 | 0 | 0 | 17 | 0 | 0 | 0 |
| 1/29/2002 | 132.52 | 4.50 | 0 | 0 | 1 | 0 | 0 | 0 |
| 1/30/2002 | 110.15 | 3.93 | 0 | 0 | 0 | 0 | 0 | 0 |
| 1/31/2002 | 97.13 | 3.77 | 0 | 0 | 0 | 0 | 0 | 0 |
| 2/1/2002 | 99.96 | 4.82 | 0 | 0 | 13 | 0 | 0 | 2 |
| 2/2/2002 | 102.51 | 4.84 | 0 | 0 | 7 | 0 | 0 | 0 |
| 2/3/2002 | 101.09 | 5.06 | 0 | 0 | 2 | 0 | 0 | 0 |
| 2/4/2002 | 103.07 | 4.94 | 0 | 0 | 0 | 0 | 0 | 0 |
| 2/5/2002 | 102.51 | 4.36 | 0 | 0 | 14 | 0 | 0 | 0 |
| 2/6/2002 | 101.37 | 4.99 | 0 | 0 | 15 | 0 | 0 | 0 |
| 2/7/2002 | 158.29 | 5.62 | 0 | 0 | 75 | 0 | 0 | 0 |
| 2/8/2002 | 342.63 | 6.01 | 0 | 0 | 15 | 0 | 0 | 0 |
| 2/9/2002 | 238.99 | 6.15 | 0 | 0 | 114 | 0 | 0 | 0 |
| 2/10/2002 | 176.98 | 6.23 | 0 | 0 | 177 | 0 | 0 | 0 |
| 2/11/2002 | 153.48 | 6.42 | 0 | 0 | 253 | 0 | 0 | 1 |
| 2/12/2002 | 141.58 | 6.62 | 0 | 0 | 345 | 0 | 0 | 1 |
| 2/13/2002 | 128.28 | 5.98 | 0 | 0 | 1 | 0 | 0 | 0 |
| 2/14/2002 | 124.59 | 5.72 | 0 | 0 | 22 | 0 | 0 | 0 |
| 2/15/2002 | 117.80 | 5.18 | 0 | 0 | 3 | 0 | 0 | 0 |
| 2/16/2002 | 114.12 | 5.57 | 0 | 0 | 8 | 0 | 0 | 0 |
| 2/17/2002 | 116.95 | 6.62 | 0 | 0 | 103 | 0 | 0 | 0 |
| 2/18/2002 | 112.70 | 6.97 | 0 | 0 | 191 | 0 | 0 | 0 |
| 2/19/2002 | 119.21 | 6.93 | 0 | 0 | 270 | 0 | 0 | 0 |
| 2/20/2002 | 204.16 | 7.04 | 0 | 0 | 402 | 0 | 0 | 0 |
| 2/21/2002 | 203.03 | 7.68 | 0 | 0 | 542 | 2 | 0 | 0 |
| 2/22/2002 | 207.56 | 8.15 | 0 | 0 | 464 | 0 | 0 | 0 |
| 2/23/2002 | 228.52 | 7.84 | 0 | 0 | 208 | 0 | 0 | 0 |
| 2/24/2002 | 214.92 | 7.72 | 0 | 0 | 109 | 0 | 0 | 0 |
| 2/25/2002 | 175.85 | 6.58 | 0 | 0 | 7 | 0 | 0 | 0 |
| 2/26/2002 | 147.53 | 6.36 | 0 | 0 | 6 | 0 | 0 | 0 |
| 2/27/2002 | 129.41 | 6.12 | 0 | 0 | 5 | 0 | 0 | 0 |

| Date | Discharge (cms) | Temp (C°) | Chinook | Coho | Steelhead | Cutthroat | Lamprey | Sucker |
|---|---|---|---|---|---|---|---|---|
| 2/28/2002 | 117.23 | 6.00 | 0 | 0 | 4 | 0 | 0 | 0 |
| 3/1/2002 | 105.06 | 5.51 | 0 | 0 | 0 | 0 | 0 | 0 |
| 3/2/2002 | 93.16 | 4.97 | 0 | 0 | 0 | 0 | 0 | 0 |
| 3/3/2002 | 82.69 | 5.02 | 0 | 0 | 1 | 0 | 0 | 0 |
| 3/4/2002 | 77.87 | 5.96 | 0 | 0 | 10 | 0 | 0 | 0 |
| 3/5/2002 | 74.19 | 5.77 | 0 | 0 | 5 | 0 | 0 | 0 |
| 3/6/2002 | 78.15 | 6.15 | 0 | 0 | 55 | 0 | 0 | 0 |
| 3/7/2002 | 115.25 | 6.62 | 0 | 0 | 200 | 0 | 0 | 0 |
| 3/8/2002 | 109.87 | 5.56 | 0 | 0 | 6 | 0 | 0 | 0 |
| 3/9/2002 | 95.71 | 5.05 | 0 | 0 | 1 | 0 | 0 | 0 |
| 3/10/2002 | 86.65 | 5.72 | 0 | 0 | 8 | 0 | 0 | 0 |
| 3/11/2002 | 90.90 | 6.65 | 0 | 0 | 104 | 0 | 0 | 0 |
| 3/12/2002 | 194.25 | 7.24 | 0 | 0 | 173 | 0 | 0 | 0 |
| 3/13/2002 | 207.85 | 6.30 | 0 | 0 | 75 | 0 | 0 | 0 |
| 3/14/2002 | 180.38 | 6.34 | 0 | 0 | 55 | 0 | 0 | 0 |
| 3/15/2002 | 158.86 | 6.02 | 0 | 0 | 39 | 0 | 0 | 0 |
| 3/16/2002 | 147.25 | 5.49 | 0 | 0 | 3 | 0 | 0 | 0 |
| 3/17/2002 | 134.22 | 5.01 | 0 | 0 | 0 | 0 | 0 | 0 |
| 3/18/2002 | 115.53 | 5.09 | 0 | 0 | 3 | 0 | 0 | 0 |
| 3/19/2002 | 106.75 | 6.76 | 0 | 0 | 49 | 0 | 0 | 0 |
| 3/20/2002 | 114.68 | 7.21 | 0 | 0 | 368 | 0 | 0 | 0 |
| 3/21/2002 | 142.43 | 7.34 | 0 | 0 | 480 | 0 | 0 | 0 |
| 3/22/2002 | 173.30 | 7.56 | 0 | 0 | 378 | 1 | 0 | 0 |
| 3/23/2002 | 184.63 | 8.05 | 0 | 0 | 456 | 0 | 0 | 0 |
| 3/24/2002 | 237.86 | 7.70 | 0 | 0 | 93 | 0 | 0 | 0 |
| 3/25/2002 | 218.61 | 8.26 | 0 | 0 | 180 | 0 | 0 | 0 |
| 3/26/2002 | 178.68 | 8.52 | 0 | 0 | 157 | 0 | 0 | 0 |
| 3/27/2002 | 154.33 | 9.00 | 0 | 0 | 149 | 0 | 0 | 0 |
| 3/28/2002 | 134.51 | 9.05 | 0 | 0 | 110 | 1 | 0 | 0 |
| 3/29/2002 | 120.06 | 9.48 | 0 | 0 | 54 | 0 | 0 | 0 |
| 3/30/2002 | 112.98 | 9.33 | 0 | 0 | 33 | 0 | 1 | 0 |
| 3/31/2002 | 107.89 | 9.14 | 0 | 0 | 15 | 0 | 0 | 0 |
| 4/1/2002 | 105.91 | 10.22 | 0 | 0 | 182 | 0 | 0 | 0 |
| 4/2/2002 | 107.89 | 10.58 | 2 | 0 | 224 | 0 | 0 | 0 |
| 4/3/2002 | 114.40 | 10.50 | 1 | 0 | 193 | 0 | 0 | 0 |
| 4/4/2002 | 120.06 | 10.84 | 4 | 0 | 184 | 0 | 0 | 0 |
| 4/5/2002 | 126.58 | 9.81 | 2 | 0 | 145 | 0 | 0 | 0 |
| 4/6/2002 | 129.69 | 9.45 | 0 | 0 | 69 | 0 | 0 | 0 |
| 4/7/2002 | 122.05 | 10.46 | 1 | 0 | 37 | 0 | 0 | 0 |
| 4/8/2002 | 112.13 | 10.62 | 11 | 0 | 95 | 1 | 0 | 0 |
| 4/9/2002 | 107.04 | 10.43 | 8 | 0 | 87 | 0 | 0 | 0 |
| 4/10/2002 | 127.99 | 10.24 | 10 | 0 | 101 | 0 | 0 | 0 |
| 4/11/2002 | 140.73 | 10.18 | 0 | 0 | 35 | 0 | 0 | 0 |
| 4/12/2002 | 143.00 | 10.80 | 1 | 0 | 27 | 0 | 0 | 1 |
| 4/13/2002 | 137.90 | 10.46 | 10 | 0 | 52 | 0 | 0 | 0 |

| Date | Discharge (cms) | Temp (C°) | Chinook | Coho | Steelhead | Cutthroat | Lamprey | Sucker |
|---|---|---|---|---|---|---|---|---|
| 4/14/2002 | 410.59 | 9.74 | 0 | 0 | 4 | 0 | 0 | 0 |
| 4/15/2002 | 351.13 | 7.53 | 0 | 0 | 0 | 0 | 0 | 0 |
| 4/16/2002 | 240.41 | 6.94 | 0 | 0 | 0 | 0 | 0 | 0 |
| 4/17/2002 | 211.24 | 6.83 | 0 | 0 | 1 | 0 | 0 | 0 |
| 4/18/2002 | 201.33 | 7.05 | 0 | 0 | 1 | 0 | 0 | 0 |
| 4/19/2002 | 177.26 | 8.00 | 0 | 0 | 0 | 1 | 0 | 0 |
| 4/20/2002 | 158.01 | 8.97 | 0 | 0 | 7 | 1 | 0 | 0 |
| 4/21/2002 | 140.17 | 10.28 | 5 | 0 | 38 | 0 | 0 | 0 |
| 4/22/2002 | 125.16 | 9.53 | 0 | 0 | 2 | 0 | 0 | 0 |
| 4/23/2002 | 115.82 | 10.49 | 0 | 0 | 0 | 0 | 0 | 0 |
| 4/24/2002 | 107.60 | 10.75 | 44 | 0 | 34 | 0 | 0 | 2 |
| 4/25/2002 | 99.39 | 12.06 | 76 | 0 | 29 | 0 | 0 | 225 |
| 4/26/2002 | 94.86 | 11.02 | 121 | 0 | 31 | 0 | 0 | 258 |
| 4/27/2002 | 96.84 | 10.45 | 57 | 0 | 20 | 0 | 0 | 0 |
| 4/28/2002 | 92.88 | 11.09 | 41 | 0 | 12 | 0 | 0 | 0 |
| 4/29/2002 | 86.08 | 10.31 | 2 | 0 | 1 | 0 | 0 | 0 |
| 4/30/2002 | 82.69 | 10.23 | 39 | 0 | 28 | 0 | 0 | 0 |
| 5/1/2002 | 80.14 | 10.50 | 66 | 0 | 3 | 0 | 1 | 1 |
| 5/2/2002 | 78.72 | 12.14 | 116 | 0 | 19 | 0 | 0 | 250 |
| 5/3/2002 | 78.72 | 12.69 | 194 | 0 | 33 | 0 | 0 | 1570 |
| 5/4/2002 | 79.85 | 13.22 | 137 | 0 | 11 | 0 | 0 | 533 |
| 5/5/2002 | 78.44 | 12.49 | 3 | 0 | 0 | 0 | 0 | 69 |
| 5/6/2002 | 76.46 | 10.93 | 0 | 0 | 0 | 0 | 0 | 0 |
| 5/7/2002 | 73.34 | 11.18 | 0 | 0 | 0 | 0 | 0 | 0 |
| 5/8/2002 | 70.79 | 11.05 | 3 | 0 | 9 | 0 | 0 | 33 |
| 5/9/2002 | 68.24 | 10.69 | 129 | 0 | 8 | 0 | 0 | 47 |
| 5/10/2002 | 65.98 | 11.77 | 56 | 0 | 7 | 0 | 0 | 0 |
| 5/11/2002 | 62.86 | 12.03 | 205 | 0 | 8 | 0 | 0 | 251 |
| 5/12/2002 | 61.45 | 12.87 | 271 | 0 | 18 | 0 | 0 | 504 |
| 5/13/2002 | 62.86 | 13.03 | 268 | 0 | 8 | 0 | 2 | 360 |
| 5/14/2002 | 65.98 | 12.54 | 118 | 0 | 10 | 0 | 0 | 64 |
| 5/15/2002 | 66.83 | 13.02 | 130 | 0 | 4 | 0 | 1 | 147 |
| 5/16/2002 | 65.98 | 13.30 | 95 | 0 | 4 | 0 | 0 | 221 |
| 5/17/2002 | 65.13 | 13.48 | 87 | 0 | 7 | 0 | 0 | 42 |
| 5/18/2002 | 66.26 | 13.32 | 121 | 0 | 1 | 0 | 0 | 14 |
| 5/19/2002 | 67.68 | 13.22 | 4 | 0 | 0 | 0 | 0 | 0 |
| 5/20/2002 | 68.53 | 12.30 | 2 | 0 | 4 | 0 | 0 | 4 |
| 5/21/2002 | 67.68 | 11.91 | 37 | 0 | 8 | 0 | 0 | 2 |
| 5/22/2002 | 68.53 | 13.08 | 7 | 0 | 0 | 0 | 0 | 0 |
| 5/23/2002 | 64.85 | 12.97 | 126 | 0 | 4 | 0 | 0 | 0 |
| 5/24/2002 | 59.47 | 13.10 | 151 | 0 | 5 | 0 | 0 | 1 |
| 5/25/2002 | 55.50 | 14.25 | 191 | 0 | 4 | 0 | 0 | 3 |
| 5/26/2002 | 54.65 | 15.41 | 118 | 0 | 9 | 1 | 1 | 33 |
| 5/27/2002 | 56.92 | 15.54 | 199 | 0 | 17 | 0 | 0 | 28 |
| 5/28/2002 | 61.73 | 14.80 | 31 | 0 | 2 | 0 | 0 | 0 |

| Date | Discharge (cms) | Temp (C°) | Chinook | Coho | Steelhead | Cutthroat | Lamprey | Sucker |
|---|---|---|---|---|---|---|---|---|
| 5/29/2002 | 66.26 | 15.86 | 81 | 0 | 32 | 0 | 1 | 23 |
| 5/30/2002 | 76.17 | 15.16 | 245 | 0 | 27 | 0 | 0 | 40 |
| 5/31/2002 | 77.02 | 15.32 | 147 | 0 | 14 | 0 | 1 | 17 |
| 6/1/2002 | 69.09 | 15.66 | 133 | 0 | 21 | 0 | 0 | 2 |
| 6/2/2002 | 66.26 | 15.45 | 146 | 0 | 22 | 0 | 2 | 0 |
| 6/3/2002 | 59.75 | 15.58 | 36 | 0 | 8 | 0 | 0 | 0 |
| 6/4/2002 | 57.48 | 16.88 | 93 | 0 | 45 | 0 | 1 | 22 |
| 6/5/2002 | 54.93 | 17.02 | 250 | 0 | 51 | 0 | 1 | 59 |
| 6/6/2002 | 53.52 | 16.92 | 86 | 0 | 25 | 0 | 0 | 7 |
| 6/7/2002 | 52.10 | 16.51 | 132 | 0 | 28 | 0 | 1 | 1 |
| 6/8/2002 | 51.54 | 15.15 | 101 | 0 | 13 | 0 | 0 | 0 |
| 6/9/2002 | 50.12 | 14.24 | 45 | 0 | 13 | 0 | 0 | 0 |
| 6/10/2002 | 47.57 | 14.76 | 81 | 0 | 21 | 0 | 0 | 0 |
| 6/11/2002 | 44.46 | 16.16 | 123 | 0 | 20 | 0 | 0 | 5 |
| 6/12/2002 | 42.76 | 17.87 | 126 | 0 | 40 | 0 | 2 | 17 |
| 6/13/2002 | 42.76 | 19.17 | 98 | 0 | 31 | 3 | 2 | 1220 |
| 6/14/2002 | 41.63 | 19.09 | 93 | 0 | 20 | 1 | 1 | 201 |
| 6/15/2002 | 41.06 | 18.48 | 45 | 0 | 18 | 0 | 0 | 41 |
| 6/16/2002 | 41.91 | 18.83 | 76 | 0 | 54 | 0 | 1 | 12 |
| 6/17/2002 | 42.19 | 18.15 | 67 | 0 | 42 | 0 | 2 | 53 |
| 6/18/2002 | 47.57 | 16.54 | 4 | 0 | 3 | 0 | 0 | 0 |
| 6/19/2002 | 58.33 | 15.66 | 7 | 0 | 6 | 1 | 0 | 29 |
| 6/20/2002 | 51.82 | 16.54 | 59 | 0 | 22 | 0 | 0 | 40 |
| 6/21/2002 | 43.32 | 17.28 | 34 | 0 | 19 | 0 | 0 | 24 |
| 6/22/2002 | 39.64 | 17.15 | 49 | 0 | 55 | 0 | 1 | 0 |
| 6/23/2002 | 37.94 | 17.85 | 39 | 0 | 35 | 0 | 0 | 8 |
| 6/24/2002 | 36.53 | 18.95 | 8 | 0 | 11 | 2 | 0 | 1 |
| 6/25/2002 | 36.81 | 19.66 | 14 | 0 | 6 | 1 | 0 | 137 |
| 6/26/2002 | 36.25 | 21.38 | 0 | 0 | 63 | 1 | 0 | 0 |
| 6/27/2002 | 36.53 | 21.10 | 21 | 0 | 139 | 1 | 2 | 143 |
| 6/28/2002 | 35.40 | 20.43 | 59 | 0 | 51 | 1 | 0 | 0 |
| 6/29/2002 | 35.68 | 19.83 | 41 | 0 | 91 | 0 | 1 | 0 |
| 6/30/2002 | 35.40 | 20.21 | 38 | 0 | 65 | 1 | 0 | 3 |
| 7/1/2002 | 32.56 | 20.61 | 24 | 0 | 69 | 1 | 0 | 1 |
| 7/2/2002 | 30.87 | 20.59 | 24 | 0 | 42 | 0 | 2 | 0 |
| 7/3/2002 | 30.30 | 20.63 | 30 | 0 | 72 | 1 | 1 | 1 |
| 7/4/2002 | 30.30 | 20.10 | 20 | 0 | 64 | 2 | 0 | 1 |
| 7/5/2002 | 30.58 | 20.07 | 24 | 0 | 86 | 0 | 0 | 0 |
| 7/6/2002 | 31.43 | 20.15 | 32 | 0 | 109 | 0 | 0 | 1 |
| 7/7/2002 | 30.58 | 19.60 | 10 | 0 | 69 | 1 | 0 | 0 |
| 7/8/2002 | 28.88 | 19.39 | 8 | 0 | 21 | 0 | 1 | 0 |
| 7/9/2002 | 28.26 | 21.26 | 15 | 0 | 105 | 1 | 0 | 1 |
| 7/10/2002 | 28.01 | 22.32 | 18 | 0 | 107 | 2 | 0 | 3 |
| 7/11/2002 | 27.86 | 23.21 | 23 | 0 | 111 | 3 | 2 | 3 |
| 7/12/2002 | 28.32 | 23.48 | 22 | 0 | 106 | 4 | 1 | 2 |

| Date | Discharge (cms) | Temp (C°) | Chinook | Coho | Steelhead | Cutthroat | Lamprey | Sucker |
|---|---|---|---|---|---|---|---|---|
| 7/13/2002 | 28.60 | 23.56 | 10 | 0 | 32 | 5 | 1 | 1 |
| 7/14/2002 | 28.32 | 21.90 | 5 | 0 | 9 | 1 | 0 | 0 |
| 7/15/2002 | 27.69 | 21.83 | 8 | 0 | 15 | 0 | 0 | 0 |
| 7/16/2002 | 27.13 | 22.23 | 3 | 0 | 10 | 0 | 0 | 0 |
| 7/17/2002 | 26.76 | 22.58 | 0 | 0 | 1 | 0 | 0 | 0 |
| 7/18/2002 | 26.19 | 22.21 | 6 | 0 | 10 | 0 | 0 | 0 |
| 7/19/2002 | 26.45 | 22.03 | 1 | 0 | 4 | 0 | 0 | 0 |
| 7/20/2002 | 26.33 | 21.12 | 0 | 0 | 5 | 2 | 0 | 0 |
| 7/21/2002 | 25.51 | 22.25 | 1 | 0 | 1 | 0 | 0 | 0 |
| 7/22/2002 | 25.17 | 23.05 | 7 | 0 | 1 | 0 | 0 | 0 |
| 7/23/2002 | 25.20 | 22.91 | 3 | 0 | 6 | 1 | 0 | 0 |
| 7/24/2002 | 24.98 | 22.79 | 3 | 0 | 4 | 0 | 0 | 0 |
| 7/25/2002 | 24.83 | 22.58 | 2 | 0 | 3 | 0 | 0 | 0 |
| 7/26/2002 | 24.55 | 23.15 | 1 | 0 | 4 | 0 | 0 | 0 |
| 7/27/2002 | 24.30 | 22.68 | 4 | 0 | 0 | 0 | 0 | 0 |
| 7/28/2002 | 23.79 | 22.38 | 3 | 0 | 0 | 0 | 0 | 0 |
| 7/29/2002 | 23.47 | 22.89 | 4 | 0 | 1 | 0 | 0 | 0 |
| 7/30/2002 | 23.33 | 23.71 | 4 | 0 | 2 | 0 | 0 | 0 |
| 7/31/2002 | 23.08 | 22.89 | 3 | 0 | 1 | 0 | 1 | 0 |
| 8/1/2002 | 22.85 | 21.65 | 1 | 0 | 1 | 0 | 0 | 0 |
| 8/2/2002 | 22.68 | 21.48 | 1 | 0 | 0 | 0 | 0 | 0 |
| 8/3/2002 | 22.71 | 20.85 | 0 | 0 | 0 | 0 | 0 | 0 |
| 8/4/2002 | 22.80 | 20.22 | 0 | 0 | 0 | 0 | 0 | 0 |
| 8/5/2002 | 23.02 | 19.35 | 0 | 0 | 0 | 0 | 0 | 0 |
| 8/6/2002 | 23.08 | 18.58 | 0 | 0 | 0 | 0 | 0 | 0 |
| 8/7/2002 | 22.94 | 18.78 | 0 | 0 | 0 | 0 | 0 | 0 |
| 8/8/2002 | 22.85 | 18.99 | 0 | 0 | 0 | 0 | 0 | 0 |
| 8/9/2002 | 22.34 | 19.18 | 34 | 0 | 35 | 0 | 0 | 0 |
| 8/10/2002 | 21.46 | 20.51 | 48 | 0 | 41 | 1 | 0 | 0 |
| 8/11/2002 | 21.24 | 21.39 | 26 | 0 | 41 | 1 | 2 | 0 |
| 8/12/2002 | 20.93 | 21.40 | 42 | 0 | 91 | 1 | 1 | 0 |
| 8/13/2002 | 20.84 | 21.84 | 20 | 0 | 66 | 2 | 0 | 0 |
| 8/14/2002 | 20.70 | 22.50 | 8 | 0 | 42 | 3 | 0 | 0 |
| 8/15/2002 | 20.59 | 22.32 | 18 | 0 | 21 | 3 | 0 | 0 |
| 8/16/2002 | 20.53 | 21.73 | 9 | 0 | 28 | 1 | 0 | 0 |
| 8/17/2002 | 20.61 | 21.61 | 8 | 0 | 11 | 0 | 0 | 0 |
| 8/18/2002 | 20.64 | 21.44 | 0 | 0 | 0 | 0 | 0 | 0 |
| 8/19/2002 | 20.67 | 20.59 | 0 | 0 | 0 | 0 | 0 | 0 |
| 8/20/2002 | 20.98 | 19.77 | 0 | 0 | 0 | 0 | 0 | 0 |
| 8/21/2002 | 20.95 | 18.90 | 0 | 0 | 0 | 0 | 0 | 0 |
| 8/22/2002 | 21.01 | 19.06 | 0 | 0 | 0 | 0 | 0 | 0 |
| 8/23/2002 | 20.95 | 19.50 | 13 | 0 | 1 | 0 | 0 | 0 |
| 8/24/2002 | 20.61 | 19.59 | 14 | 0 | 4 | 1 | 0 | 0 |
| 8/25/2002 | 20.44 | 19.97 | 10 | 0 | 3 | 0 | 0 | 0 |
| 8/26/2002 | 20.42 | 19.82 | 54 | 0 | 53 | 0 | 0 | 0 |

| Date | Discharge (cms) | Temp (C°) | Chinook | Coho | Steelhead | Cutthroat | Lamprey | Sucker |
|---|---|---|---|---|---|---|---|---|
| 8/27/2002 | 20.33 | 20.18 | 27 | 0 | 25 | 2 | 1 | 0 |
| 8/28/2002 | 20.25 | 20.44 | 25 | 0 | 37 | 1 | 0 | 0 |
| 8/29/2002 | 20.42 | 20.87 | 34 | 0 | 29 | 3 | 0 | 0 |
| 8/30/2002 | 20.25 | 20.20 | 29 | 0 | 29 | 4 | 0 | 0 |
| 8/31/2002 | 19.85 | 19.84 | 22 | 0 | 13 | 1 | 0 | 0 |
| 9/1/2002 | 19.43 | 19.84 | 18 | 0 | 18 | 4 | 0 | 0 |
| 9/2/2002 | 19.31 | 20.34 | 12 | 0 | 21 | 3 | 0 | 0 |
| 9/3/2002 | 19.28 | 20.02 | 22 | 0 | 30 | 0 | 0 | 0 |
| 9/4/2002 | 19.40 | 18.67 | 3 | 0 | 4 | 0 | 0 | 0 |
| 9/5/2002 | 19.48 | 17.90 | 0 | 0 | 0 | 0 | 0 | 0 |
| 9/6/2002 | 19.57 | 17.29 | 0 | 0 | 0 | 0 | 0 | 0 |
| 9/7/2002 | 20.33 | 16.92 | 0 | 0 | 0 | 0 | 0 | 0 |
| 9/8/2002 | 21.15 | 16.59 | 0 | 0 | 0 | 0 | 0 | 0 |
| 9/9/2002 | 20.90 | 16.38 | 0 | 0 | 0 | 0 | 0 | 0 |
| 9/10/2002 | 20.44 | 16.77 | 0 | 0 | 0 | 0 | 0 | 0 |
| 9/11/2002 | 20.13 | 16.76 | 7 | 0 | 0 | 0 | 0 | 0 |
| 9/12/2002 | 19.82 | 17.83 | 7 | 0 | 40 | 0 | 0 | 0 |
| 9/13/2002 | 19.68 | 18.25 | 11 | 0 | 27 | 2 | 0 | 0 |
| 9/14/2002 | 19.77 | 18.39 | 23 | 3 | 12 | 3 | 0 | 0 |
| 9/15/2002 | 19.77 | 17.52 | 13 | 1 | 11 | 2 | 0 | 0 |
| 9/16/2002 | 19.68 | 17.17 | 11 | 0 | 8 | 0 | 0 | 0 |
| 9/17/2002 | 21.21 | 17.20 | 8 | 2 | 14 | 0 | 0 | 0 |
| 9/18/2002 | 28.60 | 17.13 | 31 | 2 | 28 | 0 | 0 | 0 |
| 9/19/2002 | 27.18 | 17.15 | 40 | 5 | 19 | 0 | 0 | 0 |
| 9/20/2002 | 23.19 | 17.44 | 17 | 6 | 8 | 0 | 0 | 0 |
| 9/21/2002 | 22.12 | 17.19 | 10 | 2 | 5 | 2 | 0 | 0 |
| 9/22/2002 | 21.78 | 16.71 | 3 | 1 | 3 | 0 | 0 | 0 |
| 9/23/2002 | 21.55 | 16.86 | 0 | 0 | 0 | 0 | 0 | 0 |
| 9/24/2002 | 21.35 | 16.64 | 0 | 0 | 0 | 2 | 0 | 0 |
| 9/25/2002 | 21.29 | 16.42 | 0 | 0 | 0 | 0 | 0 | 0 |
| 9/26/2002 | 21.29 | 15.76 | 7 | 18 | 3 | 1 | 0 | 0 |
| 9/27/2002 | 21.29 | 16.19 | 4 | 14 | 30 | 0 | 0 | 0 |
| 9/28/2002 | 21.27 | 16.14 | 6 | 14 | 10 | 3 | 0 | 0 |
| 9/29/2002 | 21.61 | 15.78 | 3 | 4 | 1 | 1 | 0 | 0 |
| 9/30/2002 | 22.03 | 14.19 | 0 | 0 | 0 | 0 | 0 | 0 |
| 10/1/2002 | 24.78 | 13.51 | 0 | 0 | 0 | 0 | 0 | 0 |
| 10/2/2002 | 26.82 | 12.81 | 0 | 0 | 0 | 0 | 0 | 0 |
| 10/3/2002 | 25.06 | 12.03 | 3 | 5 | 0 | 0 | 0 | 0 |
| 10/4/2002 | 25.23 | 11.89 | 9 | 20 | 5 | 0 | 0 | 0 |
| 10/5/2002 | 25.88 | 12.92 | 13 | 42 | 11 | 0 | 0 | 0 |
| 10/6/2002 | 24.89 | 13.22 | 3 | 49 | 12 | 0 | 0 | 1 |
| 10/7/2002 | 23.84 | 13.74 | 0 | 0 | 0 | 0 | 0 | 0 |
| 10/8/2002 | 23.50 | 13.70 | 0 | 0 | 0 | 0 | 0 | 0 |
| 10/9/2002 | 24.35 | 13.07 | 18 | 413 | 2 | 0 | 0 | 0 |
| 10/10/2002 | 23.96 | 12.39 | 1 | 104 | 36 | 0 | 0 | 0 |

| Date | Discharge (cms) | Temp (C°) | Chinook | Coho | Steelhead | Cutthroat | Lamprey | Sucker |
|---|---|---|---|---|---|---|---|---|
| 10/11/2002 | 23.33 | 12.29 | 2 | 18 | 1 | 0 | 0 | 0 |
| 10/12/2002 | 22.68 | 11.82 | 0 | 12 | 0 | 0 | 0 | 0 |
| 10/13/2002 | 22.60 | 11.26 | 0 | 10 | 3 | 0 | 0 | 0 |
| 10/14/2002 | 22.54 | 10.89 | 1 | 14 | 0 | 0 | 0 | 0 |
| 10/15/2002 | 22.99 | 10.61 | 0 | 17 | 9 | 0 | 0 | 0 |
| 10/16/2002 | 23.16 | 10.79 | 2 | 10 | 1 | 1 | 0 | 0 |
| 10/17/2002 | 23.22 | 11.05 | 8 | 21 | 7 | 0 | 0 | 0 |
| 10/18/2002 | 23.19 | 10.99 | 7 | 56 | 10 | 1 | 0 | 0 |
| 10/19/2002 | 23.05 | 10.99 | 5 | 23 | 1 | 0 | 0 | 0 |
| 10/20/2002 | 22.99 | 11.26 | 9 | 30 | 12 | 1 | 0 | 0 |
| 10/21/2002 | 23.05 | 11.28 | 1 | 14 | 0 | 0 | 0 | 0 |
| 10/22/2002 | 23.02 | 11.44 | 0 | 0 | 0 | 0 | 0 | 0 |
| 10/23/2002 | 23.05 | 11.44 | 0 | 0 | 0 | 0 | 0 | 0 |
| 10/24/2002 | 23.22 | 10.91 | 6 | 117 | 2 | 0 | 0 | 0 |
| 10/25/2002 | 23.08 | 10.06 | 1 | 87 | 16 | 0 | 0 | 0 |
| 10/26/2002 | 22.82 | 10.17 | 2 | 8 | 7 | 0 | 0 | 0 |
| 10/27/2002 | 22.96 | 9.61 | 0 | 10 | 1 | 0 | 0 | 0 |
| 10/28/2002 | 22.88 | 9.39 | 1 | 2 | 0 | 0 | 0 | 0 |
| 10/29/2002 | 22.94 | 9.61 | 0 | 0 | 0 | 0 | 0 | 0 |
| 10/30/2002 | 22.82 | 8.27 | 0 | 0 | 2 | 0 | 0 | 0 |
| 10/31/2002 | 22.68 | 7.06 | 0 | 3 | 0 | 0 | 0 | 0 |
| 11/1/2002 | 22.85 | 5.68 | 0 | 1 | 0 | 0 | 0 | 0 |
| 11/2/2002 | 23.02 | 4.73 | 0 | 0 | 0 | 0 | 0 | 0 |
| 11/3/2002 | 23.02 | 4.60 | 1 | 0 | 0 | 0 | 0 | 0 |
| 11/4/2002 | 23.67 | 4.57 | 0 | 0 | 0 | 0 | 0 | 0 |
| 11/5/2002 | 24.07 |  | 0 | 0 | 0 | 0 | 0 | 0 |
| 11/6/2002 | 24.58 | 5.71 | 1 | 55 | 1 | 0 | 0 | 0 |
| 11/7/2002 | 26.56 | 6.65 | 1 | 23 | 2 | 0 | 0 | 0 |
| 11/8/2002 | 30.58 | 7.26 | 3 | 451 | 23 | 0 | 0 | 0 |
| 11/9/2002 | 50.40 | 7.70 | 23 | 1285 | 77 | 0 | 0 | 0 |
| 11/10/2002 | 76.46 | 7.77 | 11 | 358 | 42 | 0 | 0 | 0 |
| 11/11/2002 | 71.36 | 7.75 | 1 | 59 | 2 | 0 | 0 | 0 |
| 11/12/2002 | 56.92 | 7.94 | 0 | 120 | 1 | 2 | 0 | 0 |
| 11/13/2002 | 49.27 | 8.18 | 0 | 1000 | 63 | 0 | 0 | 0 |
| 11/14/2002 | 44.17 | 8.45 | 1 | 923 | 42 | 4 | 0 | 0 |
| 11/15/2002 | 39.36 | 8.47 | 2 | 301 | 12 | 0 | 0 | 0 |
| 11/16/2002 | 35.68 | 7.39 | 0 | 250 | 9 | 0 | 0 | 0 |
| 11/17/2002 | 34.55 | 7.53 | 0 | 132 | 6 | 0 | 0 | 0 |
| 11/18/2002 | 42.19 | 7.26 | 0 | 35 | 1 | 0 | 0 | 0 |
| 11/19/2002 | 37.94 | 7.61 | 0 | 0 | 0 | 2 | 0 | 0 |
| 11/20/2002 | 33.41 | 7.81 | 0 | 0 | 0 | 0 | 0 | 0 |
| 11/21/2002 | 31.15 | 7.74 | 0 | 39 | 3 | 0 | 0 | 0 |
| 11/22/2002 | 30.58 | 7.74 | 0 | 112 | 9 | 1 | 0 | 0 |
| 11/23/2002 | 29.45 | 8.24 | 0 | 128 | 21 | 0 | 0 | 0 |
| 11/24/2002 | 29.45 | 8.24 | 0 | 13 | 1 | 0 | 0 | 0 |

| Date | Discharge (cms) | Temp (C°) | Chinook | Coho | Steelhead | Cutthroat | Lamprey | Sucker |
|---|---|---|---|---|---|---|---|---|
| 11/25/2002 | 29.17 | 7.94 | 0 | 0 | 0 | 0 | 0 | 0 |
| 11/26/2002 | 28.88 | 6.54 | 0 | 24 | 3 | 0 | 0 | 0 |
| 11/27/2002 | 27.95 | 5.54 | 0 | 2 | 0 | 0 | 0 | 0 |
| 11/28/2002 | 27.04 | 4.93 | 0 | 0 | 0 | 0 | 0 | 0 |
| 11/29/2002 | 26.33 | 4.78 | 0 | 0 | 0 | 0 | 0 | 0 |
| 11/30/2002 | 25.94 | 4.85 | 0 | 0 | 0 | 0 | 0 | 0 |
| 12/1/2002 | 25.74 | 5.29 | 0 | 1 | 1 | 0 | 0 | 0 |
| 12/2/2002 | 25.46 | 5.56 | 0 | 6 | 0 | 1 | 0 | 0 |
| 12/3/2002 | 25.20 | 5.57 | 0 | 7 | 0 | 0 | 0 | 0 |
| 12/4/2002 | 25.37 | 5.56 | 0 | 0 | 1 | 0 | 0 | 0 |
| 12/5/2002 | 25.43 | 5.64 | 0 | 0 | 0 | 0 | 0 | 0 |
| 12/6/2002 | 25.51 | 6.11 | 0 | 20 | 2 | 0 | 0 | 0 |
| 12/7/2002 | 25.09 | 6.24 | 0 | 13 | 1 | 0 | 0 | 0 |
| 12/8/2002 | 25.09 | 6.22 | 0 | 12 | 1 | 0 | 0 | 0 |
| 12/9/2002 | 25.03 | 5.89 | 0 | 0 | 0 | 0 | 0 | 0 |
| 12/10/2002 | 26.22 | 5.73 | 0 | 0 | 0 | 0 | 0 | 0 |
| 12/11/2002 | 29.73 | 5.57 | 0 | 36 | 7 | 0 | 0 | 0 |
| 12/12/2002 | 35.96 | 6.40 | 0 | 0 | 0 | 0 | 0 | 0 |
| 12/13/2002 | 48.14 | 6.73 | 0 | 148 | 37 | 3 | 0 | 0 |
| 12/14/2002 | 69.66 | 7.61 | 0 | 113 | 69 | 2 | 0 | 0 |
| 12/15/2002 | 100.24 | 7.78 | 0 | 49 | 38 | 0 | 0 | 0 |
| 12/16/2002 | 226.53 | 7.96 | 0 | 6 | 2 | 0 | 0 | 0 |
| 12/17/2002 | 162.54 | 7.61 | 0 | 5 | 17 | 0 | 0 | 0 |
| 12/18/2002 | 96.84 | 6.64 | 0 | 5 | 2 | 0 | 0 | 0 |
| 12/19/2002 | 75.04 | 6.07 | 0 | 8 | 3 | 0 | 0 | 0 |
| 12/20/2002 | 62.86 | 5.68 | 0 | 8 | 1 | 0 | 0 | 0 |
| 12/21/2002 | 59.47 | 6.02 | 0 | 12 | 6 | 0 | 0 | 0 |
| 12/22/2002 | 93.73 | 6.28 | 0 | 9 | 28 | 0 | 0 | 0 |
| 12/23/2002 | 70.79 | 6.31 | 0 | 9 | 13 | 0 | 0 | 0 |
| 12/24/2002 | 57.77 | 5.78 | 0 | 3 | 3 | 0 | 0 | 0 |
| 12/25/2002 | 50.97 | 5.44 | 0 | 0 | 0 | 0 | 0 | 0 |
| 12/26/2002 | 52.10 | 5.66 | 0 | 1 | 6 | 0 | 0 | 0 |
| 12/27/2002 | 245.51 | 6.59 | 0 | 1 | 28 | 0 | 0 | 0 |
| 12/28/2002 | 481.39 | 7.42 | 0 | 0 | 7 | 0 | 0 | 0 |
| 12/29/2002 | 379.45 | 7.04 | 0 | 6 | 6 | 0 | 0 | 0 |
| 12/30/2002 | 322.81 | 7.00 | 0 | 0 | 22 | 0 | 0 | 0 |
| 12/31/2002 | 546.52 | 7.75 | 0 | 0 | 5 | 0 | 0 | 0 |
| 1/1/2003 | 297.33 | 7.41 | 0 | 1 | 18 | 0 | 0 | 0 |
| 1/2/2003 | 227.10 | 7.43 | 0 | 0 | 52 | 1 | 0 | 0 |
| 1/3/2003 | 495.54 | 8.15 | 0 | 0 | 9 | 0 | 0 | 0 |
| 1/4/2003 | 342.63 | 8.70 | 0 | 0 | 13 | 0 | 0 | 0 |
| 1/5/2003 | 297.33 | 8.45 | 0 | 0 | 15 | 0 | 0 | 0 |
| 1/6/2003 | 206.71 | 6.99 | 0 | 0 | 34 | 0 | 0 | 0 |
| 1/7/2003 | 155.74 | 6.30 | 0 | 0 | 21 | 0 | 0 | 0 |
| 1/8/2003 | 127.71 | 5.59 | 0 | 0 | 20 | 0 | 0 | 0 |

| Date | Discharge (cms) | Temp (C°) | Chinook | Coho | Steelhead | Cutthroat | Lamprey | Sucker |
|---|---|---|---|---|---|---|---|---|
| 1/9/2003 | 111.85 | 5.20 | 0 | 0 | 12 | 0 | 0 | 0 |
| 1/10/2003 | 100.24 | 5.38 | 0 | 0 | 14 | 0 | 0 | 0 |
| 1/11/2003 | 91.46 | 5.67 | 0 | 0 | 21 | 0 | 0 | 0 |
| 1/12/2003 | 85.80 | 6.48 | 0 | 0 | 28 | 0 | 0 | 0 |
| 1/13/2003 | 96.84 | 6.89 | 0 | 0 | 54 | 0 | 0 | 0 |
| 1/14/2003 | 159.14 | 7.81 | 0 | 0 | 70 | 0 | 0 | 0 |
| 1/15/2003 | 156.31 | 7.63 | 0 | 0 | 46 | 0 | 0 | 0 |
| 1/16/2003 | 128.28 | 6.72 | 0 | 0 | 19 | 0 | 0 | 0 |
| 1/17/2003 | 106.19 | 5.93 | 0 | 0 | 9 | 0 | 0 | 0 |
| 1/18/2003 | 95.14 | 5.94 | 0 | 0 | 6 | 0 | 0 | 0 |
| 1/19/2003 | 89.48 | 6.23 | 0 | 0 | 11 | 0 | 0 | 0 |
| 1/20/2003 | 83.25 | 6.39 | 0 | 0 | 14 | 0 | 0 | 1 |
| 1/21/2003 | 78.15 | 6.49 | 0 | 0 | 8 | 0 | 0 | 0 |
| 1/22/2003 | 75.32 | 6.86 | 0 | 0 | 44 | 0 | 0 | 0 |
| 1/23/2003 | 83.25 | 7.53 | 0 | 0 | 52 | 0 | 0 | 0 |
| 1/24/2003 | 88.07 | 7.76 | 0 | 0 | 106 | 0 | 0 | 0 |
| 1/25/2003 | 160.27 | 7.98 | 0 | 0 | 159 | 0 | 0 | 0 |
| 1/26/2003 | 178.68 | 8.84 | 0 | 0 | 227 | 0 | 0 | 2 |
| 1/27/2003 | 444.57 | 9.30 | 0 | 0 | 34 | 0 | 0 | 0 |
| 1/28/2003 | 314.32 | 7.99 | 0 | 0 | 15 | 0 | 0 | 0 |
| 1/29/2003 | 209.26 | 7.43 | 0 | 0 | 12 | 0 | 0 | 0 |
| 1/30/2003 | 376.61 | 8.00 | 0 | 0 | 26 | 0 | 0 | 0 |
| 1/31/2003 | 532.36 | 9.39 | 0 | 0 | 28 | 0 | 0 | 0 |
| 2/1/2003 | 416.26 | 9.11 | 0 | 0 | 15 | 0 | 0 | 0 |
| 2/2/2003 | 328.48 | 8.12 | 0 | 0 | 12 | 0 | 0 | 0 |
| 2/3/2003 | 246.36 | 7.67 | 0 | 0 | 0 | 0 | 0 | 0 |
| 2/4/2003 | 195.67 | 6.72 | 0 | 0 | 0 | 0 | 0 | 0 |
| 2/5/2003 | 158.86 | 6.06 | 0 | 0 | 0 | 0 | 0 | 0 |
| 2/6/2003 | 135.07 | 5.11 | 0 | 0 | 1 | 0 | 0 | 0 |
| 2/7/2003 | 115.53 | 4.56 | 0 | 0 | 0 | 0 | 0 | 0 |
| 2/8/2003 | 104.49 | 4.22 | 0 | 0 | 1 | 0 | 0 | 0 |
| 2/9/2003 | 93.16 | 4.18 | 0 | 0 | 1 | 0 | 0 | 0 |
| 2/10/2003 | 84.38 | 4.40 | 0 | 0 | 0 | 0 | 0 | 0 |
| 2/11/2003 | 79.57 | 4.36 | 0 | 0 | 0 | 0 | 0 | 0 |
| 2/12/2003 | 73.34 | 4.36 | 0 | 0 | 0 | 0 | 0 | 0 |
| 2/13/2003 | 69.09 | 4.94 | 0 | 0 | 0 | 0 | 0 | 0 |
| 2/14/2003 | 69.38 | 6.93 | 0 | 0 | 51 | 0 | 0 | 0 |
| 2/15/2003 | 67.96 | 6.88 | 0 | 0 | 161 | 0 | 0 | 0 |
| 2/16/2003 | 92.03 | 7.13 | 0 | 0 | 125 | 0 | 0 | 0 |
| 2/17/2003 | 128.84 | 7.28 | 0 | 0 | 307 | 0 | 0 | 0 |
| 2/18/2003 | 150.08 | 7.52 | 0 | 0 | 291 | 0 | 0 | 0 |
| 2/19/2003 | 149.23 | 7.69 | 0 | 0 | 311 | 0 | 0 | 0 |
| 2/20/2003 | 135.92 | 7.66 | 0 | 0 | 223 | 0 | 0 | 0 |
| 2/21/2003 | 128.84 | 7.91 | 0 | 0 | 220 | 0 | 0 | 0 |
| 2/22/2003 | 146.11 | 8.31 | 0 | 0 | 288 | 1 | 0 | 0 |

| Date | Discharge (cms) | Temp (C°) | Chinook | Coho | Steelhead | Cutthroat | Lamprey | Sucker |
|---|---|---|---|---|---|---|---|---|
| 2/23/2003 | 144.98 | 7.44 | 0 | 0 | 34 | 0 | 0 | 0 |
| 2/24/2003 | 125.16 | 6.76 | 0 | 0 | 4 | 0 | 0 | 0 |
| 2/25/2003 | 108.45 | 5.99 | 0 | 0 | 3 | 0 | 0 | 0 |
| 2/26/2003 | 96.56 | 5.37 | 0 | 0 | 2 | 0 | 0 | 0 |
| 2/27/2003 | 86.93 | 5.83 | 0 | 0 | 1 | 0 | 0 | 4 |
| 2/28/2003 | 79.00 | 5.93 | 0 | 0 | 6 | 0 | 0 | 0 |
| 3/1/2003 | 75.89 | 6.74 | 0 | 0 | 37 | 0 | 0 | 1 |
| 3/2/2003 | 70.23 | 6.64 | 0 | 0 | 14 | 0 | 0 | 2 |
| 3/3/2003 | 71.92 | 7.18 | 0 | 0 | 61 | 0 | 0 | 0 |
| 3/4/2003 | 77.02 | 7.28 | 0 | 0 | 211 | 0 | 0 | 0 |
| 3/5/2003 | 76.17 | 7.47 | 0 | 0 | 128 | 0 | 0 | 0 |
| 3/6/2003 | 79.85 | 7.51 | 0 | 0 | 157 | 0 | 0 | 0 |
| 3/7/2003 | 186.89 | 7.30 | 0 | 0 | 335 | 0 | 0 | 0 |
| 3/8/2003 | 288.83 | 8.61 | 0 | 0 | 105 | 0 | 0 | 0 |
| 3/9/2003 | 221.44 | 8.75 | 0 | 0 | 527 | 0 | 0 | 1 |
| 3/10/2003 | 278.64 | 9.38 | 0 | 0 | 139 | 0 | 0 | 0 |
| 3/11/2003 | 214.92 | 9.59 | 0 | 0 | 359 | 0 | 0 | 18 |
| 3/12/2003 | 177.26 | 9.56 | 0 | 0 | 393 | 0 | 0 | 13 |
| 3/13/2003 | 157.72 | 9.18 | 0 | 0 | 278 | 0 | 0 | 6 |
| 3/14/2003 | 153.48 | 9.64 | 0 | 0 | 128 | 0 | 0 | 1 |
| 3/15/2003 | 158.29 | 9.36 | 0 | 0 | 47 | 0 | 0 | 0 |
| 3/16/2003 | 159.99 | 8.84 | 0 | 0 | 47 | 0 | 0 | 0 |
| 3/17/2003 | 138.75 | 9.01 | 0 | 0 | 24 | 0 | 0 | 0 |
| 3/18/2003 | 120.06 | 8.59 | 0 | 0 | 21 | 0 | 0 | 0 |
| 3/19/2003 | 105.62 | 7.47 | 0 | 0 | 15 | 0 | 0 | 0 |
| 3/20/2003 | 106.19 | 8.31 | 0 | 0 | 21 | 0 | 0 | 0 |
| 3/21/2003 | 120.35 | 8.66 | 0 | 0 | 90 | 0 | 0 | 0 |
| 3/22/2003 | 242.39 | 8.17 | 0 | 0 | 54 | 0 | 0 | 1 |
| 3/23/2003 | 342.63 | 7.92 | 0 | 0 | 1 | 0 | 0 | 0 |
| 3/24/2003 | 246.64 | 7.81 | 0 | 0 | 12 | 0 | 0 | 0 |
| 3/25/2003 | 222.85 | 7.90 | 0 | 0 | 83 | 0 | 0 | 0 |
| 3/26/2003 | 472.89 | 8.19 | 0 | 0 | 14 | 1 | 0 | 0 |
| 3/27/2003 | 376.61 | 8.00 | 0 | 0 | 4 | 0 | 0 | 0 |
| 3/28/2003 | 259.38 | 8.78 | 0 | 0 | 112 | 1 | 0 | 0 |
| 3/29/2003 | 197.93 | 9.64 | 0 | 0 | 201 | 0 | 0 | 0 |
| 3/30/2003 | 165.94 | 10.44 | 0 | 0 | 235 | 1 | 0 | 4 |
| 3/31/2003 | 149.51 | 10.08 | 0 | 0 | 222 | 0 | 0 | 6 |
| 4/1/2003 | 157.44 | 9.52 | 0 | 0 | 66 | 0 | 0 | 1 |
| 4/2/2003 | 158.86 | 8.14 | 0 | 0 | 12 | 0 | 0 | 0 |
| 4/3/2003 | 154.89 | 7.27 | 0 | 0 | 5 | 0 | 0 | 0 |
| 4/4/2003 | 169.05 | 7.04 | 0 | 0 | 6 | 0 | 0 | 0 |
| 4/5/2003 | 188.87 | 6.97 | 0 | 0 | 5 | 0 | 0 | 0 |
| 4/6/2003 | 221.44 | 7.36 | 0 | 0 | 3 | 0 | 0 | 0 |
| 4/7/2003 | 227.67 | 8.83 | 0 | 0 | 42 | 0 | 0 | 0 |
| 4/8/2003 | 226.25 | 9.37 | 0 | 0 | 104 | 0 | 0 | 0 |

| Date | Discharge (cms) | Temp (C°) | Chinook | Coho | Steelhead | Cutthroat | Lamprey | Sucker |
|---|---|---|---|---|---|---|---|---|
| 4/9/2003 | 235.03 | 10.22 | 0 | 0 | 124 | 0 | 0 | 5 |
| 4/10/2003 | 213.51 | 9.89 | 1 | 0 | 59 | 0 | 0 | 1 |
| 4/11/2003 | 238.99 | 9.57 | 0 | 0 | 15 | 0 | 0 | 0 |
| 4/12/2003 | 227.38 | 9.67 | 1 | 0 | 43 | 0 | 0 | 0 |
| 4/13/2003 | 210.39 | 9.28 | 0 | 0 | 16 | 0 | 0 | 0 |
| 4/14/2003 | 209.83 | 9.05 | 1 | 0 | 18 | 0 | 0 | 0 |
| 4/15/2003 | 184.06 | 9.05 | 3 | 0 | 16 | 0 | 0 | 0 |
| 4/16/2003 | 163.95 | 9.44 | 1 | 0 | 13 | 0 | 0 | 0 |
| 4/17/2003 | 171.32 | 9.83 | 18 | 0 | 66 | 0 | 0 | 0 |
| 4/18/2003 | 173.58 | 9.70 | 5 | 0 | 18 | 0 | 0 | 0 |
| 4/19/2003 | 156.59 | 9.80 | 4 | 0 | 10 | 0 | 0 | 0 |
| 4/20/2003 | 138.47 | 10.19 | 37 | 0 | 65 | 0 | 0 | 0 |
| 4/21/2003 | 150.65 | 9.60 | 43 | 0 | 50 | 0 | 0 | 2 |
| 4/22/2003 | 141.58 | 9.58 | 10 | 0 | 9 | 0 | 0 | 4 |
| 4/23/2003 | 143.85 | 9.50 | 13 | 0 | 9 | 0 | 0 | 2 |
| 4/24/2003 | 241.83 | 8.97 | 3 | 0 | 4 | 0 | 0 | 0 |
| 4/25/2003 | 237.86 | 7.94 | 2 | 0 | 2 | 0 | 0 | 1 |
| 4/26/2003 | 190.01 | 8.51 | 1 | 0 | 5 | 0 | 0 | 1 |
| 4/27/2003 | 157.72 | 9.60 | 12 | 0 | 16 | 0 | 0 | 0 |
| 4/28/2003 | 137.90 | 9.22 | 0 | 0 | 1 | 0 | 0 | 0 |
| 4/29/2003 | 124.59 | 9.70 | 0 | 0 | 0 | 0 | 0 | 0 |
| 4/30/2003 | 115.82 | 10.72 | 26 | 0 | 38 | 0 | 0 | 6 |
| 5/1/2003 | 105.06 | 12.09 | 186 | 0 | 35 | 0 | 0 | 46 |
| 5/2/2003 | 97.13 | 13.19 | 186 | 0 | 31 | 0 | 0 | 282 |
| 5/3/2003 | 94.86 | 11.23 | 206 | 0 | 10 | 0 | 1 | 134 |
| 5/4/2003 | 109.59 | 10.63 | 37 | 0 | 8 | 0 | 0 | 8 |
| 5/5/2003 | 145.27 | 9.41 | 1 | 0 | 0 | 0 | 0 | 2 |
| 5/6/2003 | 130.54 | 9.91 | 0 | 0 | 0 | 0 | 0 | 0 |
| 5/7/2003 | 115.82 | 9.98 | 6 | 0 | 6 | 0 | 1 | 0 |
| 5/8/2003 | 107.89 | 10.94 | 126 | 0 | 10 | 0 | 1 | 0 |
| 5/9/2003 | 101.66 | 10.32 | 83 | 0 | 4 | 0 | 0 | 0 |
| 5/10/2003 | 95.14 | 10.43 | 101 | 0 | 2 | 0 | 0 | 0 |
| 5/11/2003 | 91.46 | 10.82 | 196 | 0 | 8 | 0 | 0 | 0 |
| 5/12/2003 | 90.61 | 10.28 | 49 | 0 | 9 | 0 | 0 | 21 |
| 5/13/2003 | 87.50 | 12.61 | 0 | 0 | 0 | 0 | 0 | 0 |
| 5/14/2003 | 86.08 | 14.99 | 248 | 0 | 12 | 0 | 1 | 501 |
| 5/15/2003 | 86.37 | 13.66 | 436 | 0 | 19 | 0 | 2 | 145 |
| 5/16/2003 | 84.38 | 12.91 | 248 | 0 | 6 | 0 | 1 | 1 |
| 5/17/2003 | 79.85 | 12.27 | 165 | 0 | 0 | 0 | 0 | 0 |
| 5/18/2003 | 74.47 | 12.11 | 88 | 0 | 5 | 0 | 0 | 0 |
| 5/19/2003 | 69.94 | 11.58 | 8 | 0 | 0 | 0 | 0 | 0 |
| 5/20/2003 | 67.11 | 12.42 | 47 | 0 | 5 | 0 | 0 | 17 |
| 5/21/2003 | 66.26 | 14.34 | 214 | 0 | 11 | 0 | 1 | 223 |
| 5/22/2003 | 67.11 | 15.03 | 459 | 0 | 15 | 0 | 3 | 907 |
| 5/23/2003 | 68.53 | 15.53 | 95 | 0 | 2 | 0 | 0 | 491 |

| Date | Discharge (cms) | Temp (C°) | Chinook | Coho | Steelhead | Cutthroat | Lamprey | Sucker |
|---|---|---|---|---|---|---|---|---|
| 5/24/2003 | 71.92 | 16.34 | 145 | 0 | 10 | 0 | 0 | 552 |
| 5/25/2003 | 76.46 | 15.16 | 191 | 0 | 13 | 0 | 5 | 233 |
| 5/26/2003 | 75.89 | 14.53 | 94 | 0 | 4 | 0 | 0 | 39 |
| 5/27/2003 | 71.64 | 14.41 | 120 | 0 | 16 | 0 | 7 | 269 |
| 5/28/2003 | 68.53 | 16.73 | 30 | 0 | 5 | 0 | 0 | 94 |
| 5/29/2003 | 71.36 | 16.37 | 290 | 0 | 32 | 0 | 1 | 256 |
| 5/30/2003 | 71.64 | 15.53 | 293 | 0 | 39 | 0 | 3 | 64 |
| 5/31/2003 | 71.08 | 14.45 | 37 | 0 | 2 | 0 | 0 | 0 |
| 6/1/2003 | 67.39 | 16.03 | 0 | 0 | 0 | 0 | 0 | 0 |
| 6/2/2003 | 62.58 | 16.46 | 42 | 0 | 31 | 0 | 0 | 195 |
| 6/3/2003 | 58.33 | 16.92 | 120 | 0 | 26 | 0 | 0 | 103 |
| 6/4/2003 | 57.20 | 17.73 | 200 | 0 | 40 | 0 | 1 | 160 |
| 6/5/2003 | 58.62 | 18.77 | 154 | 0 | 35 | 0 | 2 | 201 |
| 6/6/2003 | 57.77 | 19.52 | 138 | 0 | 37 | 0 | 6 | 550 |
| 6/7/2003 | 57.48 | 19.77 | 104 | 0 | 41 | 2 | 1 | 533 |
| 6/8/2003 | 56.92 | 19.86 | 115 | 0 | 25 | 1 | 3 | 199 |
| 6/9/2003 | 55.78 | 19.46 | 74 | 0 | 27 | 0 | 4 | 103 |
| 6/10/2003 | 54.37 | 19.06 | 30 | 0 | 14 | 0 | 2 | 5 |
| 6/11/2003 | 52.10 | 17.74 | 0 | 0 | 0 | 0 | 0 | 0 |
| 6/12/2003 | 49.84 | 17.38 | 38 | 0 | 3 | 0 | 0 | 240 |
| 6/13/2003 | 47.57 | 16.77 | 39 | 0 | 59 | 1 | 0 | 21 |
| 6/14/2003 | 45.59 | 16.99 | 54 | 0 | 35 | 0 | 1 | 359 |
| 6/15/2003 | 43.61 | 17.63 | 91 | 0 | 47 | 0 | 1 | 799 |
| 6/16/2003 | 42.48 | 18.17 | 76 | 0 | 41 | 0 | 2 | 20 |
| 6/17/2003 | 41.63 | 20.22 | 0 | 0 | 0 | 0 | 0 | 0 |
| 6/18/2003 | 40.21 | 19.71 | 24 | 0 | 3 | 2 | 1 | 438 |
| 6/19/2003 | 39.93 | 18.06 | 62 | 0 | 121 | 1 | 0 | 29 |
| 6/20/2003 | 39.64 | 16.80 | 19 | 0 | 35 | 0 | 0 | 3 |
| 6/21/2003 | 39.64 | 17.91 | 0 | 0 | 0 | 0 | 0 | 0 |
| 6/22/2003 | 38.79 | 17.00 | 15 | 0 | 3 | 0 | 0 | 9 |
| 6/23/2003 | 38.51 | 16.13 | 27 | 0 | 37 | 0 | 0 | 0 |
| 6/24/2003 | 37.94 | 15.52 | 24 | 0 | 9 | 0 | 0 | 3 |
| 6/25/2003 | 36.81 | 18.51 | 50 | 0 | 114 | 0 | 0 | 158 |
| 6/26/2003 | 35.68 | 18.91 | 142 | 0 | 216 | 0 | 7 | 491 |
| 6/27/2003 | 34.55 | 20.33 | 56 | 0 | 141 | 0 | 10 | 23 |
| 6/28/2003 | 33.98 | 21.15 | 80 | 0 | 267 | 1 | 43 | 8 |
| 6/29/2003 | 33.41 | 22.28 | 88 | 0 | 209 | 1 | 24 | 15 |
| 6/30/2003 | 32.85 | 22.09 | 59 | 0 | 93 | 0 | 23 | 2 |
| 7/1/2003 | 32.28 | 21.06 | 0 | 0 | 0 | 2 | 0 | 0 |
| 7/2/2003 | 31.71 | 19.75 | 41 | 0 | 14 | 1 | 0 | 2 |
| 7/3/2003 | 31.43 | 19.90 | 13 | 0 | 17 | 0 | 0 | 1 |
| 7/4/2003 | 30.87 | 20.09 | 26 | 0 | 131 | 0 | 1 | 0 |
| 7/5/2003 | 30.58 | 20.73 | 23 | 0 | 48 | 1 | 5 | 1 |
| 7/6/2003 | 30.30 | 21.17 | 27 | 0 | 38 | 0 | 2 | 0 |
| 7/7/2003 | 30.02 | 21.59 | 31 | 0 | 40 | 0 | 6 | 2 |

| Date | Discharge (cms) | Temp (C°) | Chinook | Coho | Steelhead | Cutthroat | Lamprey | Sucker |
|---|---|---|---|---|---|---|---|---|
| 7/8/2003 | 29.73 | 21.37 | 22 | 0 | 31 | 1 | 2 | 0 |
| 7/9/2003 | 29.45 | 21.69 | 21 | 0 | 47 | 0 | 1 | 0 |
| 7/10/2003 | 29.17 | 22.45 | 29 | 0 | 45 | 0 | 0 | 0 |
| 7/11/2003 | 28.20 | 22.47 | 20 | 0 | 42 | 1 | 0 | 0 |
| 7/12/2003 | 27.07 | 22.71 | 13 | 0 | 24 | 0 | 3 | 0 |
| 7/13/2003 | 26.93 | 22.63 | 7 | 0 | 17 | 1 | 1 | 0 |
| 7/14/2003 | 26.53 | 22.84 | 2 | 0 | 6 | 0 | 0 | 0 |
| 7/15/2003 | 26.62 | 22.43 | 2 | 0 | 12 | 0 | 1 | 0 |
| 7/16/2003 | 26.70 | 22.12 | 5 | 0 | 2 | 0 | 1 | 2 |
| 7/17/2003 | 26.36 | 22.27 | 3 | 0 | 4 | 0 | 1 | 0 |
| 7/18/2003 | 26.22 | 22.94 | 1 | 0 | 4 | 0 | 1 | 0 |
| 7/19/2003 | 25.91 | 23.01 | 1 | 0 | 5 | 0 | 0 | 0 |
| 7/20/2003 | 25.68 | 23.21 | 7 | 0 | 3 | 0 | 1 | 0 |
| 7/21/2003 | 25.82 | 23.94 | 5 | 0 | 5 | 0 | 2 | 0 |
| 7/22/2003 | 25.49 | 24.01 | 1 | 0 | 0 | 0 | 0 | 0 |
| 7/23/2003 | 25.17 | 24.01 | 0 | 0 | 0 | 1 | 2 | 3 |
| 7/24/2003 | 25.09 | 23.45 | 1 | 0 | 1 | 0 | 2 | 1 |
| 7/25/2003 | 24.89 | 22.77 | 0 | 0 | 0 | 0 | 0 | 1 |
| 7/26/2003 | 24.75 | 22.77 | 0 | 0 | 0 | 1 | 2 | 1 |
| 7/27/2003 | 24.58 | 22.82 | 0 | 0 | 0 | 0 | 0 | 0 |
| 7/28/2003 | 23.87 | 23.77 | 2 | 0 | 0 | 1 | 3 | 0 |
| 7/29/2003 | 23.33 | 24.38 | 1 | 0 | 0 | 0 | 0 | 1 |
| 7/30/2003 | 23.19 | 24.67 | 0 | 0 | 0 | 0 | 1 | 2 |
| 7/31/2003 | 23.25 | 24.52 | 0 | 0 | 0 | 0 | 2 | 2 |
| 8/1/2003 | 23.36 | 23.66 | 0 | 0 | 2 | 1 | 0 | 1 |
| 8/2/2003 | 23.22 | 22.85 | 0 | 0 | 0 | 0 | 0 | 1 |
| 8/3/2003 | 23.05 | 21.72 | 0 | 0 | 0 | 0 | 0 | 0 |
| 8/4/2003 | 22.40 | 22.10 | 1 | 0 | 0 | 0 | 0 | 0 |
| 8/5/2003 | 22.17 | 22.33 | 0 | 0 | 3 | 0 | 0 | 1 |
| 8/6/2003 | 22.96 | 21.91 | 1 | 0 | 4 | 0 | 0 | 0 |
| 8/7/2003 | 23.87 | 21.93 | 2 | 0 | 2 | 0 | 0 | 0 |
| 8/8/2003 | 27.27 | 21.84 | 16 | 0 | 2 | 0 | 0 | 0 |
| 8/9/2003 | 25.91 | 21.79 | 38 | 0 | 0 | 0 | 0 | 0 |
| 8/10/2003 | 24.58 | 21.80 | 30 | 0 | 2 | 0 | 0 | 1 |
| 8/11/2003 | 23.33 | 21.63 | 29 | 0 | 10 | 0 | 1 | 0 |
| 8/12/2003 | 22.88 | 21.39 | 35 | 0 | 23 | 0 | 0 | 0 |
| 8/13/2003 | 22.60 | 21.45 | 29 | 0 | 30 | 0 | 0 | 0 |
| 8/14/2003 | 22.17 | 21.56 | 39 | 0 | 33 | 0 | 0 | 0 |
| 8/15/2003 | 21.78 | 21.49 | 28 | 0 | 33 | 0 | 1 | 0 |
| 8/16/2003 | 21.63 | 21.17 | 13 | 0 | 19 | 0 | 2 | 0 |
| 8/17/2003 | 21.24 | 21.18 | 21 | 0 | 20 | 0 | 0 | 0 |
| 8/18/2003 | 21.21 | 21.88 | 12 | 0 | 14 | 0 | 1 | 0 |
| 8/19/2003 | 21.63 | 22.49 | 0 | 0 | 0 | 0 | 0 | 0 |
| 8/20/2003 | 21.89 | 21.96 | 0 | 0 | 0 | 0 | 0 | 0 |
| 8/21/2003 | 22.46 | 21.82 | 0 | 0 | 0 | 0 | 0 | 0 |

| Date | Discharge (cms) | Temp (C°) | Chinook | Coho | Steelhead | Cutthroat | Lamprey | Sucker |
|---|---|---|---|---|---|---|---|---|
| 8/22/2003 | 21.80 | 21.60 | 0 | 0 | 0 | 0 | 0 | 0 |
| 8/23/2003 | 21.63 | 20.85 | 0 | 0 | 0 | 0 | 0 | 0 |
| 8/24/2003 | 21.44 | 20.92 | 0 | 0 | 0 | 1 | 0 | 0 |
| 8/25/2003 | 20.70 | 20.99 | 0 | 0 | 0 | 0 | 0 | 0 |
| 8/26/2003 | 20.47 | 20.81 | 0 | 0 | 0 | 0 | 0 | 0 |
| 8/27/2003 | 20.16 | 20.03 | 23 | 0 | 34 | 0 | 0 | 0 |
| 8/28/2003 | 20.10 | 20.38 | 32 | 0 | 10 | 0 | 0 | 0 |
| 8/29/2003 | 20.05 | 20.36 | 30 | 0 | 14 | 0 | 0 | 0 |
| 8/30/2003 | 20.27 | 20.67 | 73 | 0 | 25 | 0 | 0 | 0 |
| 8/31/2003 | 20.95 | 20.80 | 62 | 0 | 26 | 0 | 0 | 0 |
| 9/1/2003 | 20.90 | 20.36 | 47 | 0 | 17 | 0 | 0 | 0 |
| 9/2/2003 | 21.01 | 20.32 | 30 | 0 | 33 | 0 | 0 | 0 |
| 9/3/2003 | 20.84 | 20.60 | 40 | 0 | 31 | 0 | 0 | 0 |
| 9/4/2003 | 21.32 | 20.29 | 26 | 0 | 23 | 1 | 0 | 0 |
| 9/5/2003 | 21.72 | 20.58 | 25 | 0 | 19 | 0 | 0 | 0 |
| 9/6/2003 | 21.86 | 20.66 | 35 | 0 | 10 | 0 | 0 | 0 |
| 9/7/2003 | 22.63 | 19.99 | 43 | 0 | 14 | 0 | 0 | 0 |
| 9/8/2003 | 23.53 | 18.61 | 3 | 0 | 0 | 0 | 0 | 0 |
| 9/9/2003 | 26.48 | 17.23 | 0 | 0 | 0 | 0 | 0 | 0 |
| 9/10/2003 | 34.55 | 16.66 | 13 | 0 | 1 | 0 | 0 | 12 |
| 9/11/2003 | 29.73 | 16.93 | 2 | 0 | 0 | 0 | 0 | 0 |
| 9/12/2003 | 25.80 | 18.12 | 20 | 0 | 47 | 0 | 0 | 0 |
| 9/13/2003 | 24.61 | 17.44 | 18 | 1 | 17 | 0 | 0 | 0 |
| 9/14/2003 | 23.50 | 17.48 | 9 | 1 | 10 | 0 | 0 | 0 |
| 9/15/2003 | 22.77 | 17.19 | 15 | 1 | 28 | 0 | 0 | 0 |
| 9/16/2003 | 22.71 | 16.25 | 24 | 2 | 24 | 0 | 0 | 0 |
| 9/17/2003 | 23.87 | 15.35 | 9 | 0 | 7 | 0 | 0 | 0 |
| 9/18/2003 | 25.23 | 15.84 | 3 | 0 | 5 | 0 | 0 | 0 |
| 9/19/2003 | 24.55 | 15.80 | 9 | 1 | 12 | 0 | 0 | 0 |
| 9/20/2003 | 24.07 | 15.66 | 9 | 3 | 16 | 0 | 0 | 0 |
| 9/21/2003 | 23.73 | 15.43 | 16 | 7 | 8 | 0 | 0 | 0 |
| 9/22/2003 | 23.62 | 15.97 | 10 | 5 | 13 | 0 | 0 | 0 |
| 9/23/2003 | 23.59 | 16.36 | 14 | 11 | 25 | 0 | 0 | 0 |
| 9/24/2003 | 23.64 | 16.38 | 19 | 22 | 15 | 0 | 0 | 0 |
| 9/25/2003 | 23.50 | 16.59 | 26 | 36 | 19 | 0 | 0 | 0 |
| 9/26/2003 | 23.25 | 16.54 | 16 | 19 | 18 | 0 | 0 | 0 |
| 9/27/2003 | 23.73 | 17.19 | 24 | 23 | 18 | 1 | 0 | 0 |
| 9/28/2003 | 23.79 | 17.31 | 27 | 30 | 19 | 0 | 0 | 0 |
| 9/29/2003 | 23.64 | 16.95 | 40 | 66 | 29 | 0 | 0 | 0 |
| 9/30/2003 | 23.56 | 16.01 | 11 | 27 | 11 | 0 | 0 | 0 |
| 10/1/2003 | 23.76 | 15.91 | 9 | 24 | 7 | 0 | 0 | 0 |
| 10/2/2003 | 23.79 | 15.73 | 9 | 48 | 1 | 0 | 0 | 0 |
| 10/3/2003 | 24.18 | 14.97 | 20 | 51 | 23 | 1 | 0 | 0 |
| 10/4/2003 | 24.49 | 15.16 | 19 | 74 | 13 | 0 | 0 | 0 |
| 10/5/2003 | 24.07 | 14.64 | 20 | 66 | 13 | 0 | 0 | 0 |

| Date | Discharge (cms) | Temp (C°) | Chinook | Coho | Steelhead | Cutthroat | Lamprey | Sucker |
|---|---|---|---|---|---|---|---|---|
| 10/6/2003 | 24.24 | 14.39 | 11 | 35 | 1 | 0 | 0 | 0 |
| 10/7/2003 | 24.52 | 16.29 | 24 | 61 | 6 | 0 | 0 | 1 |
| 10/8/2003 | 24.38 | 15.12 | 36 | 158 | 16 | 1 | 0 | 0 |
| 10/9/2003 | 24.72 | 13.82 | 14 | 26 | 2 | 0 | 0 | 0 |
| 10/10/2003 | 25.43 | 13.20 | 14 | 22 | 8 | 0 | 0 | 0 |
| 10/11/2003 | 26.25 | 12.64 | 12 | 33 | 2 | 0 | 0 | 0 |
| 10/12/2003 | 27.41 | 11.73 | 21 | 26 | 1 | 0 | 0 | 0 |
| 10/13/2003 | 28.32 | 11.70 | 14 | 50 | 8 | 0 | 0 | 0 |
| 10/14/2003 | 27.41 | 11.94 | 17 | 27 | 12 | 0 | 0 | 0 |
| 10/15/2003 | 26.87 | 11.77 | 19 | 24 | 14 | 0 | 0 | 0 |
| 10/16/2003 | 28.88 | 11.73 | 27 | 91 | 7 | 1 | 0 | 0 |
| 10/17/2003 | 29.73 | 12.34 | 60 | 190 | 21 | 0 | 0 | 0 |
| 10/18/2003 | 27.98 | 12.50 | 29 | 127 | 19 | 0 | 0 | 0 |
| 10/19/2003 | 26.25 | 12.63 | 7 | 13 | 1 | 0 | 0 | 0 |
| 10/20/2003 | 25.29 | 12.66 | 3 | 19 | 4 | 0 | 0 | 0 |
| 10/21/2003 | 24.98 | 13.50 | 13 | 19 | 0 | 0 | 0 | 0 |
| 10/22/2003 | 24.98 | 13.61 | 16 | 121 | 17 | 0 | 0 | 1 |
| 10/23/2003 | 25.26 | 13.10 | 13 | 83 | 22 | 0 | 0 | 0 |
| 10/24/2003 | 25.57 | 12.27 | 15 | 32 | 6 | 0 | 0 | 0 |
| 10/25/2003 | 25.26 | 11.39 | 4 | 9 | 1 | 0 | 0 | 0 |
| 10/26/2003 | 25.32 | 11.24 | 6 | 8 | 4 | 0 | 0 | 0 |
| 10/27/2003 | 24.83 | 11.08 | 5 | 6 | 1 | 0 | 0 | 0 |
| 10/28/2003 | 24.27 | 10.95 | 2 | 2 | 0 | 0 | 0 | 0 |
| 10/29/2003 | 23.70 | 11.47 | 0 | 0 | 0 | 0 | 0 | 0 |
| 10/30/2003 | 24.07 | 10.28 | 1 | 27 | 8 | 0 | 0 | 0 |
| 10/31/2003 | 25.49 | 8.94 | 5 | 7 | 0 | 0 | 0 | 0 |
| 11/1/2003 | 25.49 | 7.50 | 0 | 0 | 1 | 0 | 0 | 0 |
| 11/2/2003 | 25.49 | 6.75 | 0 | 0 | 0 | 0 | 0 | 0 |
| 11/3/2003 | 26.90 | 5.94 | 0 | 0 | 0 | 0 | 0 | 0 |
| 11/4/2003 | 28.32 | 5.52 | 0 | 0 | 0 | 0 | 0 | 0 |
| 11/5/2003 | 29.73 | 6.23 | 2 | 2 | 0 | 0 | 0 | 0 |
| 11/6/2003 | 31.15 | 6.30 | 3 | 7 | 0 | 0 | 0 | 0 |
| 11/7/2003 | 31.15 | 6.53 | 6 | 9 | 3 | 0 | 0 | 0 |
| 11/8/2003 | 29.73 | 7.01 | 11 | 17 | 3 | 0 | 0 | 0 |
| 11/9/2003 | 28.32 | 7.60 | 10 | 36 | 7 | 0 | 0 | 0 |
| 11/10/2003 | 28.32 | 7.79 | 2 | 23 | 0 | 0 | 0 | 0 |
| 11/11/2003 | 28.32 | 8.24 | 0 | 0 | 0 | 0 | 0 | 0 |
| 11/12/2003 | 29.73 | 8.22 | 0 | 0 | 0 | 0 | 0 | 0 |
| 11/13/2003 | 32.56 | 8.23 | 0 | 0 | 0 | 1 | 0 | 0 |
| 11/14/2003 | 31.15 | 7.85 | 4 | 217 | 22 | 0 | 0 | 0 |
| 11/15/2003 | 28.32 | 7.41 | 1 | 54 | 7 | 0 | 0 | 0 |
| 11/16/2003 | 32.56 | 6.23 | 1 | 28 | 17 | 0 | 0 | 0 |
| 11/17/2003 | 79.29 | 7.53 | 2 | 142 | 23 | 0 | 0 | 0 |
| 11/18/2003 | 82.12 | 8.35 | 14 | 527 | 85 | 0 | 0 | 0 |
| 11/19/2003 | 48.99 | 8.97 | 0 | 41 | 3 | 2 | 0 | 0 |

| Date | Discharge (cms) | Temp (C°) | Chinook | Coho | Steelhead | Cutthroat | Lamprey | Sucker |
|---|---|---|---|---|---|---|---|---|
| 11/20/2003 | 55.78 | 7.47 | 0 | 59 | 19 | 0 | 0 | 1 |
| 11/21/2003 | 51.54 | 6.67 | 1 | 46 | 12 | 0 | 0 | 0 |
| 11/22/2003 | 43.04 | 5.83 | 0 | 76 | 7 | 1 | 0 | 0 |
| 11/23/2003 | 37.38 | 5.13 | 0 | 3 | 0 | 0 | 0 | 0 |
| 11/24/2003 | 35.68 | 4.86 | 0 | 3 | 0 | 0 | 0 | 0 |
| 11/25/2003 | 40.21 | 4.67 | 0 | 0 | 0 | 0 | 0 | 0 |
| 11/26/2003 | 52.39 | 4.97 | 0 | 0 | 0 | 0 | 0 | 0 |
| 11/27/2003 | 59.75 | 4.98 | 0 | 0 | 0 | 0 | 0 | 0 |
| 11/28/2003 | 47.01 | 5.31 | 0 | 0 | 0 | 0 | 0 | 0 |
| 11/29/2003 | 105.91 | 5.94 | 0 | 0 | 0 | 0 | 0 | 0 |
| 11/30/2003 | 218.32 | 7.44 | 0 | 54 | 29 | 1 | 0 | 0 |
| 12/1/2003 | 122.90 | 7.95 | 2 | 32 | 37 | 1 | 0 | 0 |
| 12/2/2003 | 99.11 | 7.52 | 0 | 98 | 63 | 0 | 0 | 0 |
| 12/3/2003 | 86.08 | 7.78 | 0 | 72 | 53 | 0 | 0 | 0 |
| 12/4/2003 | 95.71 | 7.82 | 0 | 32 | 35 | 0 | 0 | 0 |
| 12/5/2003 | 93.16 | 7.90 | 0 | 20 | 34 | 0 | 0 | 0 |
| 12/6/2003 | 212.38 | 8.11 | 0 | 13 | 15 | 0 | 0 | 0 |
| 12/7/2003 | 190.57 | 7.78 | 0 | 8 | 28 | 0 | 0 | 0 |
| 12/8/2003 | 183.49 | 7.44 | 0 | 2 | 24 | 0 | 0 | 0 |
| 12/9/2003 | 140.17 | 6.79 | 0 | 2 | 34 | 0 | 0 | 0 |
| 12/10/2003 | 131.96 | 6.79 | 0 | 1 | 38 | 1 | 0 | 0 |
| 12/11/2003 | 108.17 | 6.58 | 0 | 4 | 18 | 0 | 0 | 0 |
| 12/12/2003 | 127.43 | 6.94 | 0 | 0 | 33 | 0 | 0 | 0 |
| 12/13/2003 | 838.18 | 8.09 | 0 | 0 | 0 | 0 | 0 | 0 |
| 12/14/2003 | 807.03 | 7.10 | 0 | 0 | 0 | 0 | 0 | 0 |
| 12/15/2003 | 336.97 | 6.40 | 0 | 0 | 0 | 0 | 0 | 0 |
| 12/16/2003 | 213.23 | 6.17 | 0 | 0 | 10 | 0 | 0 | 0 |
| 12/17/2003 | 176.13 | 6.61 | 0 | 0 | 45 | 0 | 0 | 0 |
| 12/18/2003 | 182.93 | 6.06 | 0 | 0 | 15 | 0 | 0 | 0 |
| 12/19/2003 | 182.08 | 5.98 | 0 | 0 | 19 | 0 | 0 | 0 |
| 12/20/2003 | 183.78 | 7.09 | 0 | 0 | 78 | 0 | 0 | 0 |
| 12/21/2003 | 168.49 | 7.52 | 0 | 0 | 79 | 0 | 0 | 0 |
| 12/22/2003 | 138.75 | 6.66 | 0 | 0 | 38 | 0 | 0 | 0 |
| 12/23/2003 | 118.93 | 6.35 | 0 | 1 | 21 | 0 | 0 | 0 |
| 12/24/2003 | 124.88 | 6.91 | 0 | 0 | 43 | 0 | 0 | 0 |
| 12/25/2003 | 179.25 | 6.87 | 0 | 0 | 21 | 0 | 0 | 0 |
| 12/26/2003 | 165.09 | 5.97 | 0 | 0 | 12 | 0 | 0 | 0 |
| 12/27/2003 | 134.22 | 5.56 | 0 | 0 | 9 | 0 | 0 | 0 |
| 12/28/2003 | 134.22 | 5.48 | 0 | 0 | 11 | 0 | 0 | 0 |
| 12/29/2003 | 250.04 | 6.04 | 0 | 0 | 5 | 0 | 0 | 0 |
| 12/30/2003 | 244.94 | 6.11 | 0 | 0 | 17 | 0 | 0 | 0 |
| 12/31/2003 | 176.41 | 5.98 | 0 | 0 | 35 | 0 | 0 | 0 |
| 1/1/2004 | 233.61 | 5.63 | 0 | 0 | 0 | 0 | 0 | 0 |
| 1/2/2004 | 241.54 | 5.45 | 0 | 0 | 5 | 0 | 0 | 0 |
| 1/3/2004 | 166.79 | 5.54 | 0 | 0 | 13 | 0 | 0 | 0 |

| Date | Discharge (cms) | Temp (C°) | Chinook | Coho | Steelhead | Cutthroat | Lamprey | Sucker |
|---|---|---|---|---|---|---|---|---|
| 1/4/2004 | 140.73 | 5.28 | 0 | 0 | 17 | 0 | 0 | 0 |
| 1/5/2004 | 119.50 | 5.07 | 0 | 0 | 0 | 0 | 0 | 0 |
| 1/6/2004 | 114.68 | 5.31 | 0 | 0 | 0 | 0 | 0 | 0 |
| 1/7/2004 | 173.58 | 6.34 | 0 | 0 | 181 | 0 | 0 | 0 |
| 1/8/2004 | 236.16 | 6.62 | 0 | 0 | 53 | 0 | 0 | 0 |
| 1/9/2004 | 300.16 | 7.10 | 0 | 0 | 50 | 0 | 0 | 0 |
| 1/10/2004 | 297.33 | 7.21 | 0 | 0 | 80 | 0 | 0 | 0 |
| 1/11/2004 | 228.23 | 6.91 | 0 | 0 | 136 | 0 | 0 | 0 |
| 1/12/2004 | 205.01 | 6.28 | 0 | 0 | 92 | 0 | 0 | 0 |
| 1/13/2004 | 203.88 | 6.95 | 0 | 0 | 170 | 0 | 0 | 0 |
| 1/14/2004 | 209.83 | 7.12 | 0 | 0 | 212 | 0 | 0 | 0 |
| 1/15/2004 | 251.74 | 7.57 | 0 | 0 | 74 | 0 | 0 | 0 |
| 1/16/2004 | 212.66 | 7.53 | 0 | 0 | 137 | 0 | 0 | 0 |
| 1/17/2004 | 173.02 | 6.67 | 0 | 0 | 45 | 0 | 0 | 0 |
| 1/18/2004 | 153.48 | 6.84 | 0 | 0 | 91 | 0 | 0 | 0 |
| 1/19/2004 | 168.20 | 7.44 | 0 | 0 | 171 | 0 | 0 | 0 |
| 1/20/2004 | 218.89 | 7.37 | 0 | 0 | 91 | 0 | 0 | 0 |
| 1/21/2004 | 178.68 | 6.37 | 0 | 0 | 25 | 0 | 0 | 0 |
| 1/22/2004 | 145.55 | 5.26 | 0 | 0 | 3 | 0 | 0 | 0 |
| 1/23/2004 | 153.48 | 5.40 | 0 | 0 | 13 | 0 | 0 | 0 |
| 1/24/2004 | 376.61 | 7.03 | 0 | 0 | 35 | 0 | 0 | 0 |
| 1/25/2004 | 322.81 | 6.49 | 0 | 0 | 9 | 0 | 0 | 0 |
| 1/26/2004 | 243.52 | 6.27 | 0 | 0 | 43 | 0 | 0 | 0 |
| 1/27/2004 | 334.14 | 6.62 | 0 | 0 | 8 | 0 | 0 | 0 |
| 1/28/2004 | 410.59 | 7.34 | 0 | 0 | 11 | 0 | 0 | 0 |
| 1/29/2004 | 433.25 | 7.95 | 0 | 0 | 138 | 0 | 0 | 0 |
| 1/30/2004 | 441.74 | 7.59 | 0 | 0 | 72 | 0 | 0 | 0 |
| 1/31/2004 | 356.79 | 6.47 | 0 | 0 | 14 | 0 | 0 | 0 |
| 2/1/2004 | 268.16 | 6.74 | 0 | 0 | 63 | 0 | 0 | 0 |
| 2/2/2004 | 255.98 | 7.11 | 0 | 0 | 118 | 0 | 0 | 0 |
| 2/3/2004 | 233.90 | 7.07 | 0 | 0 | 343 | 0 | 0 | 0 |
| 2/4/2004 | 196.24 | 7.37 | 0 | 0 | 317 | 0 | 0 | 0 |
| 2/5/2004 | 168.77 | 6.92 | 0 | 0 | 130 | 0 | 0 | 0 |
| 2/6/2004 | 153.48 | 6.65 | 0 | 0 | 57 | 0 | 0 | 0 |
| 2/7/2004 | 181.79 | 6.85 | 0 | 0 | 100 | 0 | 0 | 0 |
| 2/8/2004 | 176.41 | 6.63 | 0 | 0 | 56 | 0 | 0 | 0 |
| 2/9/2004 | 152.63 | 6.02 | 0 | 0 | 14 | 0 | 0 | 0 |
| 2/10/2004 | 134.22 | 5.91 | 0 | 0 | 6 | 0 | 0 | 0 |
| 2/11/2004 | 122.05 | 5.35 | 0 | 0 | 2 | 0 | 0 | 0 |
| 2/12/2004 | 115.53 | 5.62 | 0 | 0 | 3 | 0 | 0 | 0 |
| 2/13/2004 | 109.30 | 5.02 | 0 | 0 | 1 | 0 | 0 | 0 |
| 2/14/2004 | 104.77 | 5.52 | 0 | 0 | 7 | 0 | 0 | 0 |
| 2/15/2004 | 106.75 | 6.65 | 0 | 0 | 64 | 0 | 0 | 0 |
| 2/16/2004 | 122.90 | 6.95 | 0 | 0 | 324 | 0 | 0 | 0 |
| 2/17/2004 | 288.83 | 7.12 | 0 | 0 | 52 | 1 | 0 | 0 |

| Date | Discharge (cms) | Temp (C°) | Chinook | Coho | Steelhead | Cutthroat | Lamprey | Sucker |
|---|---|---|---|---|---|---|---|---|
| 2/18/2004 | 373.78 | 7.75 | 0 | 0 | 108 | 0 | 0 | 0 |
| 2/19/2004 | 300.16 | 7.77 | 0 | 0 | 110 | 0 | 0 | 0 |
| 2/20/2004 | 237.30 | 6.92 | 0 | 0 | 129 | 0 | 0 | 0 |
| 2/21/2004 | 188.87 | 6.74 | 0 | 0 | 238 | 0 | 0 | 0 |
| 2/22/2004 | 161.41 | 6.86 | 0 | 0 | 1 | 0 | 0 | 0 |
| 2/23/2004 | 148.10 | 7.72 | 0 | 0 | 620 | 0 | 0 | 0 |
| 2/24/2004 | 136.49 | 7.94 | 0 | 0 | 370 | 0 | 0 | 0 |
| 2/25/2004 | 128.28 | 7.71 | 0 | 0 | 169 | 0 | 0 | 0 |
| 2/26/2004 | 148.66 | 7.24 | 0 | 0 | 166 | 0 | 0 | 0 |
| 2/27/2004 | 161.97 | 7.36 | 0 | 0 | 144 | 0 | 0 | 0 |
| 2/28/2004 | 171.03 | 7.57 | 0 | 0 | 172 | 0 | 0 | 0 |
| 2/29/2004 | 159.99 | 7.26 | 0 | 0 | 2 | 0 | 0 | 0 |
| 3/1/2004 | 181.51 | 7.06 | 0 | 0 | 43 | 0 | 0 | 0 |
| 3/2/2004 | 199.92 | 7.87 | 0 | 0 | 76 | 0 | 0 | 0 |
| 3/3/2004 | 170.18 | 7.04 | 0 | 0 | 61 | 0 | 0 | 0 |
| 3/4/2004 | 154.04 | 7.32 | 0 | 0 | 62 | 0 | 0 | 0 |
| 3/5/2004 | 182.64 | 7.16 | 0 | 0 | 81 | 0 | 0 | 0 |
| 3/6/2004 | 215.77 | 8.24 | 0 | 0 | 248 | 0 | 0 | 0 |
| 3/7/2004 | 198.78 | 8.97 | 0 | 0 | 472 | 0 | 0 | 3 |
| 3/8/2004 | 174.71 | 9.35 | 0 | 0 | 484 | 0 | 0 | 9 |
| 3/9/2004 | 169.62 | 9.64 | 0 | 0 | 390 | 0 | 0 | 19 |
| 3/10/2004 | 163.11 | 9.29 | 0 | 0 | 188 | 0 | 0 | 9 |
| 3/11/2004 | 145.83 | 9.17 | 0 | 0 | 64 | 0 | 0 | 1 |
| 3/12/2004 | 133.09 | 9.20 | 0 | 0 | 30 | 0 | 0 | 0 |
| 3/13/2004 | 124.59 | 9.13 | 0 | 0 | 38 | 0 | 0 | 1 |
| 3/14/2004 | 116.67 | 9.91 | 0 | 0 | 111 | 0 | 0 | 0 |
| 3/15/2004 | 112.13 | 8.93 | 0 | 0 | 16 | 0 | 0 | 0 |
| 3/16/2004 | 106.75 | 9.16 | 0 | 0 | 0 | 0 | 0 | 0 |
| 3/17/2004 | 101.94 | 9.62 | 1 | 0 | 144 | 0 | 0 | 0 |
| 3/18/2004 | 101.37 | 9.47 | 0 | 0 | 240 | 0 | 0 | 0 |
| 3/19/2004 | 102.22 | 9.85 | 0 | 0 | 146 | 0 | 0 | 0 |
| 3/20/2004 | 96.56 | 9.54 | 0 | 0 | 120 | 0 | 0 | 0 |
| 3/21/2004 | 91.75 | 9.51 | 0 | 0 | 132 | 0 | 0 | 0 |
| 3/22/2004 | 92.88 | 10.48 | 0 | 0 | 354 | 0 | 0 | 54 |
| 3/23/2004 | 96.84 | 10.90 | 0 | 0 | 388 | 0 | 0 | 214 |
| 3/24/2004 | 99.96 | 10.67 | 1 | 0 | 355 | 0 | 0 | 181 |
| 3/25/2004 | 100.81 | 9.23 | 0 | 0 | 72 | 0 | 0 | 2 |
| 3/26/2004 | 104.21 | 8.05 | 0 | 0 | 8 | 0 | 0 | 0 |
| 3/27/2004 | 111.57 | 8.39 | 0 | 0 | 6 | 0 | 0 | 0 |
| 3/28/2004 | 118.65 | 9.36 | 0 | 0 | 40 | 0 | 0 | 0 |
| 3/29/2004 | 103.92 | 10.33 | 0 | 0 | 114 | 0 | 0 | 3 |
| 3/30/2004 | 103.36 | 9.65 | 0 | 0 | 107 | 0 | 0 | 8 |
| 3/31/2004 | 106.75 | 9.98 | 0 | 0 | 135 | 0 | 0 | 9 |
| 4/1/2004 | 97.69 | 9.55 | 0 | 0 | 19 | 0 | 0 | 4 |
| 4/2/2004 | 88.35 | 9.61 | 0 | 0 | 18 | 0 | 0 | 0 |

| Date | Discharge (cms) | Temp (C°) | Chinook | Coho | Steelhead | Cutthroat | Lamprey | Sucker |
|---|---|---|---|---|---|---|---|---|
| 4/3/2004 | 79.57 | 10.56 | 2 | 0 | 117 | 0 | 0 | 0 |
| 4/4/2004 | 75.32 | 10.68 | 2 | 0 | 241 | 0 | 0 | 246 |
| 4/5/2004 | 74.19 | 10.49 | 3 | 0 | 172 | 0 | 0 | 163 |
| 4/6/2004 | 74.76 | 10.61 | 0 | 0 | 98 | 0 | 0 | 22 |
| 4/7/2004 | 72.77 | 10.89 | 6 | 0 | 108 | 0 | 0 | 38 |
| 4/8/2004 | 71.08 | 10.93 | 10 | 0 | 67 | 0 | 0 | 43 |
| 4/9/2004 | 70.51 | 11.94 | 9 | 0 | 105 | 0 | 0 | 830 |
| 4/10/2004 | 69.09 | 12.42 | 11 | 0 | 67 | 0 | 0 | 1330 |
| 4/11/2004 | 68.81 | 12.17 | 11 | 0 | 22 | 0 | 0 | 153 |
| 4/12/2004 | 69.09 | 12.58 | 27 | 0 | 102 | 0 | 0 | 904 |
| 4/13/2004 | 72.21 | 11.72 | 6 | 0 | 36 | 0 | 0 | 17 |
| 4/14/2004 | 75.32 | 11.51 | 10 | 0 | 20 | 0 | 0 | 11 |
| 4/15/2004 | 80.42 | 10.66 | 6 | 0 | 11 | 0 | 0 | 0 |
| 4/16/2004 | 82.97 | 9.85 | 2 | 0 | 4 | 0 | 0 | 0 |
| 4/17/2004 | 79.00 | 10.03 | 0 | 0 | 6 | 0 | 0 | 0 |
| 4/18/2004 | 75.04 | 10.12 | 6 | 0 | 15 | 0 | 0 | 0 |
| 4/19/2004 | 73.06 | 9.46 | 0 | 0 | 3 | 0 | 0 | 0 |
| 4/20/2004 | 85.80 | 8.88 | 0 | 0 | 7 | 0 | 0 | 0 |
| 4/21/2004 | 199.07 | 8.18 | 1 | 0 | 16 | 0 | 0 | 0 |
| 4/22/2004 | 235.60 | 9.35 | 0 | 0 | 21 | 0 | 0 | 0 |
| 4/23/2004 | 164.52 | 10.11 | 12 | 0 | 49 | 0 | 0 | 2 |
| 4/24/2004 | 135.07 | 11.12 | 4 | 0 | 25 | 0 | 0 | 63 |
| 4/25/2004 | 116.95 | 10.99 | 0 | 0 | 0 | 0 | 0 | 7 |
| 4/26/2004 | 107.32 | 12.48 | 0 | 0 | 0 | 0 | 0 | 0 |
| 4/27/2004 | 101.94 | 13.24 | 5 | 0 | 0 | 0 | 0 | 1939 |
| 4/28/2004 | 99.11 | 12.91 | 8 | 0 | 56 | 0 | 0 | 0 |
| 4/29/2004 | 97.13 | 13.32 | 87 | 0 | 9 | 0 | 2 | 136 |
| 4/30/2004 | 91.18 | 12.27 | 8 | 0 | 4 | 0 | 0 | 16 |
| 5/1/2004 | 88.07 | 14.65 | 57 | 0 | 13 | 0 | 0 | 681 |
| 5/2/2004 | 88.63 | 13.85 | 66 | 0 | 15 | 0 | 1 | 402 |
| 5/3/2004 | 89.20 | 14.20 | 12 | 0 | 0 | 0 | 0 | 70 |
| 5/4/2004 | 89.48 | 13.69 | 6 | 0 | 2 | 0 | 0 | 149 |
| 5/5/2004 | 86.08 | 13.98 | 26 | 0 | 6 | 0 | 0 | 25 |
| 5/6/2004 | 80.99 | 12.88 | 115 | 0 | 5 | 0 | 0 | 16 |
| 5/7/2004 | 75.61 | 12.53 | 120 | 0 | 7 | 0 | 0 | 6 |
| 5/8/2004 | 75.32 | 13.17 | 168 | 0 | 12 | 0 | 0 | 27 |
| 5/9/2004 | 74.76 | 12.48 | 10 | 0 | 0 | 0 | 0 | 5 |
| 5/10/2004 | 71.36 | 11.97 | 9 | 0 | 7 | 0 | 0 | 0 |
| 5/11/2004 | 80.14 | 12.12 | 14 | 0 | 0 | 1 | 0 | 0 |
| 5/12/2004 | 93.16 | 12.27 | 135 | 0 | 5 | 0 | 0 | 43 |
| 5/13/2004 | 82.69 | 13.62 | 139 | 0 | 12 | 0 | 0 | 239 |
| 5/14/2004 | 76.46 | 13.14 | 201 | 0 | 20 | 0 | 2 | 212 |
| 5/15/2004 | 71.36 | 13.41 | 287 | 0 | 13 | 0 | 3 | 187 |
| 5/16/2004 | 69.38 | 13.56 | 3 | 0 | 0 | 0 | 1 | 3 |
| 5/17/2004 | 69.66 | 13.84 | 26 | 0 | 8 | 0 | 0 | 140 |

| Date | Discharge (cms) | Temp (C°) | Chinook | Coho | Steelhead | Cutthroat | Lamprey | Sucker |
|---|---|---|---|---|---|---|---|---|
| 5/18/2004 | 82.97 | 13.40 | 39 | 0 | 0 | 0 | 0 | 0 |
| 5/19/2004 | 158.86 | 11.74 | 0 | 0 | 0 | 0 | 0 | 0 |
| 5/20/2004 | 128.84 | 11.67 | 17 | 0 | 8 | 0 | 1 | 113 |
| 5/21/2004 | 110.44 | 13.36 | 310 | 0 | 16 | 0 | 0 | 318 |
| 5/22/2004 | 100.24 | 13.38 | 227 | 0 | 11 | 0 | 0 | 60 |
| 5/23/2004 | 97.13 | 13.13 | 149 | 0 | 5 | 0 | 1 | 5 |
| 5/24/2004 | 89.76 | 12.44 | 6 | 0 | 0 | 0 | 0 | 1 |
| 5/25/2004 | 79.85 | 13.81 | 15 | 0 | 5 | 0 | 0 | 375 |
| 5/26/2004 | 76.17 | 15.11 | 223 | 0 | 27 | 1 | 0 | 610 |
| 5/27/2004 | 75.89 | 14.30 | 192 | 0 | 27 | 0 | 0 | 179 |
| 5/28/2004 | 135.92 | 13.52 | 53 | 0 | 13 | 1 | 2 | 17 |
| 5/29/2004 | 189.72 | 11.74 | 2 | 0 | 2 | 0 | 1 | 0 |
| 5/30/2004 | 153.48 | 12.23 | 4 | 0 | 4 | 0 | 0 | 0 |
| 5/31/2004 | 119.78 | 12.19 | 66 | 0 | 33 | 0 | 0 | 717 |
| 6/1/2004 | 102.79 | 14.37 | 0 | 0 | 0 | 0 | 0 | 0 |
| 6/2/2004 | 92.88 | 15.22 | 59 | 0 | 6 | 1 | 0 | 345 |
| 6/3/2004 | 86.65 | 15.86 | 211 | 0 | 73 | 0 | 0 | 772 |
| 6/4/2004 | 81.55 | 16.12 | 103 | 0 | 52 | 0 | 2 | 643 |
| 6/5/2004 | 77.02 | 16.44 | 51 | 0 | 21 | 2 | 0 | 51 |
| 6/6/2004 | 76.17 | 14.89 | 178 | 0 | 59 | 1 | 0 | 147 |
| 6/7/2004 | 77.02 | 14.14 | 78 | 0 | 41 | 0 | 0 | 0 |
| 6/8/2004 | 80.99 | 12.79 | 61 | 0 | 35 | 0 | 0 | 0 |
| 6/9/2004 | 86.65 | 12.49 | 49 | 0 | 41 | 0 | 0 | 0 |
| 6/10/2004 | 102.79 | 12.11 | 45 | 0 | 61 | 0 | 0 | 11 |
| 6/11/2004 | 117.23 | 12.72 | 42 | 0 | 23 | 0 | 0 | 89 |
| 6/12/2004 | 100.81 | 13.13 | 29 | 0 | 36 | 0 | 0 | 2 |
| 6/13/2004 | 87.78 | 13.44 | 4 | 0 | 2 | 1 | 0 | 2 |
| 6/14/2004 | 79.57 | 14.88 | 25 | 0 | 5 | 1 | 0 | 23 |
| 6/15/2004 | 73.34 | 16.37 | 66 | 0 | 67 | 0 | 1 | 6 |
| 6/16/2004 | 68.24 | 16.67 | 107 | 0 | 100 | 1 | 0 | 18 |
| 6/17/2004 | 65.13 | 17.53 | 54 | 0 | 62 | 0 | 1 | 41 |
| 6/18/2004 | 62.58 | 18.27 | 74 | 0 | 85 | 1 | 0 | 8 |
| 6/19/2004 | 59.18 | 18.70 | 78 | 0 | 109 | 3 | 1 | 17 |
| 6/20/2004 | 57.48 | 19.39 | 67 | 0 | 79 | 1 | 1 | 74 |
| 6/21/2004 | 55.22 | 19.54 | 36 | 0 | 66 | 0 | 0 | 10 |
| 6/22/2004 | 53.52 | 19.46 | 5 | 0 | 45 | 3 | 6 | 16 |
| 6/23/2004 | 52.67 | 16.84 | 44 | 0 | 100 | 0 | 0 | 0 |
| 6/24/2004 | 50.69 | 17.25 | 25 | 0 | 122 | 2 | 0 | 1 |
| 6/25/2004 | 49.27 | 18.74 | 29 | 0 | 73 | 1 | 0 | 18 |
| 6/26/2004 | 47.01 | 20.11 | 35 | 0 | 47 | 3 | 0 | 2 |
| 6/27/2004 | 45.59 | 20.05 | 19 | 0 | 54 | 1 | 0 | 0 |
| 6/28/2004 | 44.46 | 20.29 | 8 | 0 | 0 | 0 | 1 | 35 |
| 6/29/2004 | 43.04 | 20.99 | 5 | 0 | 83 | 2 | 0 | 0 |
| 6/30/2004 | 43.04 | 21.27 | 51 | 0 | 199 | 0 | 0 | 6 |
| 7/1/2004 | 42.19 | 21.29 | 40 | 0 | 205 | 0 | 1 | 27 |

| Date | Discharge (cms) | Temp (C°) | Chinook | Coho | Steelhead | Cutthroat | Lamprey | Sucker |
|---|---|---|---|---|---|---|---|---|
| 7/2/2004 | 41.63 | 20.85 | 16 | 0 | 93 | 0 | 1 | 6 |
| 7/3/2004 | 41.34 | 20.89 | 36 | 0 | 118 | 1 | 0 | 15 |
| 7/4/2004 | 41.06 | 20.03 | 7 | 0 | 38 | 0 | 0 | 0 |
| 7/5/2004 | 40.21 | 20.60 | 8 | 0 | 26 | 0 | 1 | 2 |
| 7/6/2004 | 39.08 | 20.94 | 6 | 0 | 11 | 0 | 0 | 1 |
| 7/7/2004 | 37.38 | 21.78 | 0 | 0 | 0 | 0 | 0 | 0 |
| 7/8/2004 | 36.53 | 20.74 | 17 | 0 | 60 | 2 | 0 | 2 |
| 7/9/2004 | 35.68 | 20.59 | 6 | 0 | 45 | 1 | 0 | 0 |
| 7/10/2004 | 34.83 | 20.50 | 8 | 0 | 31 | 2 | 1 | 0 |
| 7/11/2004 | 34.83 | 20.42 | 7 | 0 | 25 | 1 | 0 | 0 |
| 7/12/2004 | 33.41 | 21.05 | 5 | 0 | 16 | 0 | 0 | 0 |
| 7/13/2004 | 33.70 | 21.67 | 7 | 0 | 12 | 0 | 1 | 2 |
| 7/14/2004 | 32.85 | 21.73 | 17 | 0 | 21 | 1 | 1 | 0 |
| 7/15/2004 | 31.71 | 22.09 | 16 | 0 | 36 | 0 | 0 | 0 |
| 7/16/2004 | 30.58 | 22.46 | 7 | 0 | 38 | 1 | 0 | 2 |
| 7/17/2004 | 30.87 | 22.59 | 9 | 0 | 47 | 1 | 0 | 1 |
| 7/18/2004 | 30.87 | 22.40 | 2 | 0 | 1 | 1 | 2 | 0 |
| 7/19/2004 | 31.15 | 22.65 | 0 | 0 | 0 | 0 | 0 | 0 |
| 7/20/2004 | 30.87 | 22.01 | 2 | 0 | 13 | 0 | 0 | 0 |
| 7/21/2004 | 30.87 | 22.24 | 0 | 0 | 7 | 0 | 0 | 0 |
| 7/22/2004 | 30.87 | 22.43 | 6 | 0 | 6 | 2 | 0 | 0 |
| 7/23/2004 | 30.87 | 23.10 | 1 | 0 | 6 | 0 | 1 | 0 |
| 7/24/2004 | 30.02 | 23.73 | 0 | 0 | 3 | 1 | 0 | 0 |
| 7/25/2004 | 29.17 | 23.87 | 2 | 0 | 1 | 4 | 0 | 0 |
| 7/26/2004 | 28.60 | 23.43 | 0 | 0 | 2 | 1 | 0 | 1 |
| 7/27/2004 | 27.95 | 23.37 | 1 | 0 | 0 | 1 | 0 | 0 |
| 7/28/2004 | 28.32 | 23.64 | 0 | 0 | 1 | 1 | 0 | 0 |
| 7/29/2004 | 28.23 | 23.73 | 0 | 0 | 0 | 0 | 0 | 0 |
| 7/30/2004 | 27.52 | 23.63 | 0 | 0 | 0 | 0 | 0 | 1 |
| 7/31/2004 | 26.99 | 23.17 | 0 | 0 | 0 | 0 | 0 | 0 |
| 8/1/2004 | 26.84 | 22.69 | 0 | 0 | 1 | 0 | 0 | 0 |
| 8/2/2004 | 26.56 | 22.68 | 0 | 0 | 1 | 1 | 0 | 0 |
| 8/3/2004 | 26.93 | 21.93 | 1 | 0 | 0 | 0 | 0 | 0 |
| 8/4/2004 | 27.18 | 22.25 | 3 | 0 | 1 | 0 | 0 | 0 |
| 8/5/2004 | 27.24 | 21.04 | 10 | 0 | 2 | 0 | 0 | 0 |
| 8/6/2004 | 27.13 | 20.54 | 17 | 0 | 9 | 0 | 0 | 0 |
| 8/7/2004 | 27.27 | 19.73 | 23 | 0 | 4 | 0 | 0 | 0 |
| 8/8/2004 | 27.07 | 21.11 | 0 | 0 | 0 | 0 | 0 | 0 |
| 8/9/2004 | 26.33 | 21.47 | 41 | 0 | 101 | 0 | 0 | 0 |
| 8/10/2004 | 25.63 | 22.15 | 38 | 0 | 110 | 0 | 0 | 0 |
| 8/11/2004 | 24.92 | 22.55 | 30 | 0 | 106 | 2 | 0 | 0 |
| 8/12/2004 | 24.15 | 23.00 | 8 | 0 | 87 | 0 | 2 | 0 |
| 8/13/2004 | 23.53 | 23.21 | 19 | 0 | 39 | 2 | 0 | 0 |
| 8/14/2004 | 23.50 | 23.15 | 9 | 0 | 21 | 0 | 0 | 0 |
| 8/15/2004 | 23.96 | 23.00 | 0 | 0 | 5 | 0 | 1 | 0 |

| Date | Discharge (cms) | Temp (C°) | Chinook | Coho | Steelhead | Cutthroat | Lamprey | Sucker |
|---|---|---|---|---|---|---|---|---|
| 8/16/2004 | 24.61 | 22.60 | 3 | 0 | 5 | 2 | 1 | 0 |
| 8/17/2004 | 24.86 | 22.45 | 1 | 0 | 4 | 0 | 0 | 0 |
| 8/18/2004 | 24.92 | 22.59 | 1 | 0 | 3 | 0 | 1 | 0 |
| 8/19/2004 | 24.49 | 22.82 | 2 | 0 | 3 | 0 | 0 | 0 |
| 8/20/2004 | 24.32 | 22.87 | 1 | 0 | 3 | 0 | 0 | 0 |
| 8/21/2004 | 23.93 | 22.77 | 1 | 0 | 0 | 0 | 0 | 0 |
| 8/22/2004 | 24.72 | 21.81 | 0 | 0 | 0 | 0 | 0 | 0 |
| 8/23/2004 | 29.17 | 20.10 | 0 | 0 | 0 | 0 | 0 | 0 |
| 8/24/2004 | 30.58 | 18.85 | 0 | 0 | 0 | 0 | 0 | 0 |
| 8/25/2004 | 29.73 | 18.29 | 2 | 0 | 2 | 0 | 0 | 0 |
| 8/26/2004 | 31.71 | 18.06 | 3 | 0 | 17 | 0 | 0 | 0 |
| 8/27/2004 | 39.36 | 17.83 | 7 | 0 | 11 | 0 | 0 | 0 |
| 8/28/2004 | 33.13 | 18.47 | 10 | 0 | 7 | 0 | 0 | 0 |
| 8/29/2004 | 29.17 | 18.80 | 8 | 0 | 10 | 0 | 0 | 0 |
| 8/30/2004 | 26.73 | 20.16 | 0 | 0 | 0 | 0 | 0 | 0 |
| 8/31/2004 | 25.80 | 20.73 | 0 | 0 | 0 | 0 | 0 | 0 |
| 9/1/2004 | 25.82 | 20.72 | 39 | 2 | 7 | 0 | 0 | 0 |
| 9/2/2004 | 26.22 | 18.54 | 13 | 0 | 55 | 0 | 0 | 0 |
| 9/3/2004 | 26.79 | 17.44 | 7 | 0 | 29 | 1 | 0 | 0 |
| 9/4/2004 | 27.27 | 17.77 | 11 | 0 | 29 | 0 | 0 | 0 |
| 9/5/2004 | 27.07 | 17.92 | 12 | 1 | 20 | 1 | 0 | 0 |
| 9/6/2004 | 26.87 | 17.86 | 16 | 0 | 11 | 0 | 0 | 0 |
| 9/7/2004 | 26.65 | 17.81 | 14 | 0 | 18 | 0 | 0 | 0 |
| 9/8/2004 | 26.50 | 17.99 | 7 | 0 | 14 | 0 | 0 | 0 |
| 9/9/2004 | 26.48 | 18.26 | 17 | 0 | 23 | 0 | 0 | 0 |
| 9/10/2004 | 26.19 | 18.16 | 8 | 2 | 12 | 0 | 0 | 0 |
| 9/11/2004 | 26.33 | 18.40 | 15 | 2 | 31 | 0 | 0 | 0 |
| 9/12/2004 | 26.45 | 17.65 | 9 | 2 | 15 | 0 | 0 | 0 |
| 9/13/2004 | 27.10 | 17.21 | 3 | 0 | 3 | 0 | 0 | 0 |
| 9/14/2004 | 28.88 | 17.12 | 1 | 0 | 6 | 0 | 0 | 0 |
| 9/15/2004 | 30.02 | 17.30 | 6 | 2 | 5 | 1 | 0 | 0 |
| 9/16/2004 | 31.71 | 16.74 | 5 | 0 | 10 | 0 | 0 | 0 |
| 9/17/2004 | 32.00 | 16.27 | 3 | 2 | 9 | 0 | 0 | 0 |
| 9/18/2004 | 32.56 | 15.13 | 12 | 0 | 14 | 0 | 0 | 0 |
| 9/19/2004 | 41.63 | 14.37 | 18 | 8 | 14 | 0 | 0 | 0 |
| 9/20/2004 | 37.38 | 14.36 | 10 | 9 | 9 | 0 | 0 | 0 |
| 9/21/2004 | 37.66 | 14.10 | 5 | 9 | 12 | 0 | 0 | 0 |
| 9/22/2004 | 34.26 | 14.26 | 9 | 23 | 11 | 0 | 0 | 0 |
| 9/23/2004 | 31.71 | 14.76 | 8 | 21 | 11 | 0 | 0 | 0 |
| 9/24/2004 | 30.30 | 15.00 | 14 | 34 | 9 | 0 | 0 | 0 |
| 9/25/2004 | 29.73 | 15.08 | 7 | 73 | 20 | 1 | 0 | 0 |
| 9/26/2004 | 28.32 | 14.13 | 10 | 29 | 7 | 0 | 0 | 0 |
| 9/27/2004 | 27.67 | 14.82 | 5 | 14 | 7 | 0 | 0 | 0 |
| 9/28/2004 | 27.35 | 16.48 | 11 | 35 | 9 | 0 | 0 | 0 |
| 9/29/2004 | 27.69 | 16.29 | 12 | 142 | 21 | 0 | 0 | 0 |

| Date | Discharge (cms) | Temp (C°) | Chinook | Coho | Steelhead | Cutthroat | Lamprey | Sucker |
|---|---|---|---|---|---|---|---|---|
| 9/30/2004 | 28.06 | 15.10 | 16 | 156 | 8 | 1 | 0 | 0 |
| 10/1/2004 | 27.72 | 15.18 | 5 | 32 | 1 | 0 | 0 | 0 |
| 10/2/2004 | 27.41 | 15.49 | 4 | 34 | 9 | 0 | 0 | 0 |
| 10/3/2004 | 26.67 | 15.46 | 7 | 29 | 3 | 0 | 0 | 0 |
| 10/4/2004 | 26.31 | 15.37 | 7 | 12 | 4 | 0 | 0 | 0 |
| 10/5/2004 | 26.73 | 15.08 | 5 | 28 | 15 | 0 | 0 | 0 |
| 10/6/2004 | 26.65 | 15.05 | 6 | 36 | 10 | 0 | 0 | 0 |
| 10/7/2004 | 26.90 | 15.02 | 12 | 24 | 11 | 0 | 0 | 0 |
| 10/8/2004 | 27.35 | 14.80 | 21 | 50 | 10 | 0 | 0 | 0 |
| 10/9/2004 | 30.30 | 13.59 | 33 | 69 | 16 | 0 | 0 | 0 |
| 10/10/2004 | 33.98 | 13.26 | 1 | 11 | 1 | 0 | 0 | 0 |
| 10/11/2004 | 30.87 | 13.03 | 2 | 5 | 1 | 0 | 0 | 0 |
| 10/12/2004 | 29.17 | 13.10 | 0 | 0 | 0 | 0 | 0 | 0 |
| 10/13/2004 | 28.06 | 12.77 | 5 | 57 | 17 | 0 | 0 | 1 |
| 10/14/2004 | 27.58 | 12.98 | 3 | 51 | 6 | 0 | 0 | 0 |
| 10/15/2004 | 27.01 | 12.98 | 3 | 16 | 3 | 0 | 0 | 0 |
| 10/16/2004 | 26.73 | 12.82 | 7 | 29 | 3 | 0 | 0 | 0 |
| 10/17/2004 | 27.72 | 12.77 | 0 | 9 | 0 | 0 | 0 | 0 |
| 10/18/2004 | 42.48 | 11.24 | 19 | 90 | 8 | 0 | 0 | 0 |
| 10/19/2004 | 56.35 | 10.55 | 26 | 365 | 27 | 0 | 0 | 1 |
| 10/20/2004 | 44.17 | 10.29 | 7 | 384 | 19 | 0 | 0 | 1 |
| 10/21/2004 | 38.23 | 10.36 | 9 | 150 | 17 | 0 | 0 | 0 |
| 10/22/2004 | 36.81 | 10.47 | 4 | 37 | 3 | 0 | 0 | 0 |
| 10/23/2004 | 86.65 | 10.55 | 22 | 54 | 17 | 0 | 0 | 1 |
| 10/24/2004 | 107.04 | 9.82 | 19 | 332 | 20 | 0 | 0 | 0 |
| 10/25/2004 | 69.09 | 9.05 | 1 | 168 | 1 | 0 | 0 | 0 |
| 10/26/2004 | 51.54 | 8.81 | 0 | 0 | 0 | 0 | 0 | 0 |
| 10/27/2004 | 48.14 | 8.92 | 1 | 5 | 0 | 0 | 0 | 0 |
| 10/28/2004 | 50.12 | 8.64 | 3 | 188 | 25 | 0 | 0 | 0 |
| 10/29/2004 | 48.14 | 8.98 | 2 | 138 | 12 | 0 | 0 | 0 |
| 10/30/2004 | 45.59 | 9.34 | 3 | 119 | 13 | 0 | 0 | 0 |
| 10/31/2004 | 51.82 | 9.22 | 0 | 15 | 0 | 0 | 0 | 0 |
| 11/1/2004 | 52.39 | 8.85 | 0 | 0 | 0 | 0 | 0 | 0 |
| 11/2/2004 | 48.70 | 8.76 | 0 | 0 | 0 | 0 | 0 | 0 |
| 11/3/2004 | 75.32 | 8.56 | 2 | 79 | 1 | 0 | 0 | 0 |
| 11/4/2004 | 82.97 | 7.93 | 0 | 95 | 25 | 0 | 0 | 0 |
| 11/5/2004 | 65.13 | 6.91 | 0 | 52 | 4 | 0 | 0 | 0 |
| 11/6/2004 | 54.65 | 6.59 | 0 | 6 | 0 | 0 | 0 | 0 |
| 11/7/2004 | 46.72 | 6.42 | 1 | 4 | 0 | 0 | 0 | 0 |
| 11/8/2004 | 41.63 | 6.41 | 0 | 5 | 2 | 0 | 0 | 0 |
| 11/9/2004 | 38.51 | 6.87 | 1 | 19 | 7 | 0 | 0 | 0 |
| 11/10/2004 | 36.53 | 7.39 | 1 | 31 | 4 | 0 | 0 | 0 |
| 11/11/2004 | 35.11 | 7.78 | 1 | 46 | 8 | 0 | 0 | 0 |
| 11/12/2004 | 33.98 | 8.46 | 0 | 73 | 13 | 0 | 0 | 0 |
| 11/13/2004 | 33.41 | 8.32 | 0 | 40 | 9 | 0 | 0 | 0 |

| Date | Discharge (cms) | Temp (C°) | Chinook | Coho | Steelhead | Cutthroat | Lamprey | Sucker |
|---|---|---|---|---|---|---|---|---|
| 11/14/2004 | 33.13 | 8.04 | 0 | 22 | 8 | 0 | 0 | 0 |
| 11/15/2004 | 32.56 | 7.87 | 0 | 6 | 7 | 0 | 0 | 0 |
| 11/16/2004 | 32.28 | 8.31 | 0 | 9 | 5 | 0 | 0 | 0 |
| 11/17/2004 | 32.00 | 8.25 | 0 | 10 | 6 | 0 | 0 | 0 |
| 11/18/2004 | 31.71 | 7.77 | 0 | 16 | 6 | 0 | 0 | 0 |
| 11/19/2004 | 32.00 | 7.35 | 0 | 3 | 4 | 0 | 0 | 0 |
| 11/20/2004 | 31.43 | 6.26 | 0 | 0 | 2 | 0 | 0 | 0 |
| 11/21/2004 | 30.58 | 6.00 | 0 | 0 | 1 | 0 | 0 | 0 |
| 11/22/2004 | 29.73 | 5.35 | 0 | 0 | 0 | 0 | 0 | 0 |
| 11/23/2004 | 29.17 | 5.12 | 0 | 0 | 0 | 0 | 0 | 0 |
| 11/24/2004 | 29.17 | 5.70 | 0 | 0 | 0 | 0 | 0 | 0 |
| 11/25/2004 | 30.30 | 6.97 | 0 | 6 | 2 | 0 | 0 | 0 |
| 11/26/2004 | 43.61 | 6.88 | 0 | 22 | 28 | 0 | 0 | 0 |
| 11/27/2004 | 46.16 | 6.94 | 0 | 65 | 46 | 0 | 0 | 0 |
| 11/28/2004 | 41.34 | 6.43 | 0 | 15 | 4 | 0 | 0 | 0 |
| 11/29/2004 | 37.66 | 5.76 | 0 | 4 | 1 | 0 | 0 | 0 |
| 11/30/2004 | 35.68 | 5.26 | 0 | 0 | 1 | 0 | 0 | 0 |
| 12/1/2004 | 35.11 | 5.10 | 0 | 2 | 0 | 0 | 0 | 0 |
| 12/2/2004 | 35.40 | 4.96 | 0 | 0 | 0 | 0 | 0 | 0 |
| 12/3/2004 | 35.96 | 5.13 | 0 | 0 | 1 | 0 | 0 | 0 |
| 12/4/2004 | 35.40 | 5.14 | 0 | 2 | 0 | 0 | 0 | 0 |
| 12/5/2004 | 34.55 | 5.08 | 0 | 5 | 0 | 0 | 0 | 0 |
| 12/6/2004 | 37.66 | 4.83 | 0 | 8 | 2 | 0 | 0 | 0 |
| 12/7/2004 | 126.86 | 5.26 | 0 | 53 | 3 | 0 | 0 | 0 |
| 12/8/2004 | 317.15 | 6.13 | 0 | 36 | 31 | 0 | 0 | 0 |
| 12/9/2004 | 1042.06 | 8.53 | 0 | 0 | 0 | 0 | 0 | 0 |
| 12/10/2004 | 662.61 | 8.89 | 0 | 4 | 3 | 0 | 0 | 0 |
| 12/11/2004 | 308.65 | 8.92 | 0 | 72 | 7 | 0 | 0 | 0 |
| 12/12/2004 | 198.78 | 8.82 | 0 | 56 | 55 | 0 | 0 | 0 |
| 12/13/2004 | 152.63 | 8.39 | 0 | 17 | 57 | 1 | 0 | 0 |
| 12/14/2004 | 140.17 | 8.43 | 0 | 13 | 98 | 0 | 0 | 1 |
| 12/15/2004 | 131.39 | 7.89 | 0 | 3 | 53 | 1 | 0 | 0 |
| 12/16/2004 | 113.83 | 6.89 | 0 | 11 | 16 | 0 | 0 | 0 |
| 12/17/2004 | 98.26 | 5.70 | 0 | 2 | 10 | 0 | 0 | 0 |
| 12/18/2004 | 84.95 | 5.86 | 0 | 1 | 44 | 0 | 0 | 0 |
| 12/19/2004 | 76.74 | 6.04 | 0 | 3 | 33 | 0 | 0 | 0 |
| 12/20/2004 | 71.64 | 6.04 | 0 | 0 | 16 | 0 | 0 | 0 |
| 12/21/2004 | 65.98 | 5.63 | 0 | 0 | 2 | 0 | 0 | 0 |
| 12/22/2004 | 61.45 | 5.52 | 0 | 0 | 5 | 0 | 0 | 0 |
| 12/23/2004 | 57.77 | 5.46 | 0 | 0 | 3 | 0 | 0 | 0 |
| 12/24/2004 | 54.09 | 4.89 | 0 | 0 | 1 | 0 | 0 | 0 |
| 12/25/2004 | 51.82 | 3.85 | 0 | 0 | 0 | 0 | 0 | 0 |
| 12/26/2004 | 57.20 | 4.00 | 0 | 0 | 1 | 0 | 0 | 0 |
| 12/27/2004 | 58.05 | 4.50 | 0 | 1 | 4 | 0 | 0 | 0 |
| 12/28/2004 | 54.65 | 5.08 | 0 | 0 | 7 | 0 | 0 | 0 |

| Date | Discharge (cms) | Temp (C°) | Chinook | Coho | Steelhead | Cutthroat | Lamprey | Sucker |
|---|---|---|---|---|---|---|---|---|
| 12/29/2004 | 55.78 | 5.29 | 0 | 2 | 7 | 0 | 0 | 0 |
| 12/30/2004 | 59.75 | 5.27 | 0 | 0 | 9 | 0 | 0 | 0 |
| 12/31/2004 | 60.03 | 5.08 | 0 | 0 | 4 | 0 | 0 | 0 |
| 1/1/2005 | 60.88 | 5.27 | 0 | 1 | 3 | 0 | 0 | 0 |
| 1/2/2005 | 65.13 | 4.70 | 0 | 0 | 2 | 0 | 0 | 0 |
| 1/3/2005 | 62.01 | 4.49 | 0 | 0 | 0 | 0 | 0 | 0 |
| 1/4/2005 | 58.62 | 4.20 | 0 | 0 | 0 | 0 | 0 | 0 |
| 1/5/2005 | 55.50 | 3.38 | 0 | 0 | 0 | 0 | 0 | 0 |
| 1/6/2005 | 52.39 | 3.19 | 0 | 0 | 0 | 0 | 0 | 0 |
| 1/7/2005 | 52.39 | 3.46 | 0 | 0 | 0 | 0 | 0 | 0 |
| 1/8/2005 | 61.73 | 3.57 | 0 | 0 | 0 | 0 | 0 | 0 |
| 1/9/2005 | 75.04 | 3.58 | 0 | 0 | 0 | 0 | 0 | 0 |
| 1/10/2005 | 69.66 | 3.81 | 0 | 0 | 0 | 0 | 0 | 0 |
| 1/11/2005 | 65.98 | 4.15 | 0 | 0 | 10 | 0 | 0 | 0 |
| 1/12/2005 | 61.73 | 4.16 | 0 | 0 | 3 | 0 | 0 | 0 |
| 1/13/2005 | 57.48 | 4.44 | 0 | 0 | 4 | 0 | 0 | 0 |
| 1/14/2005 | 54.65 | 4.15 | 0 | 0 | 0 | 0 | 0 | 0 |
| 1/15/2005 | 54.93 | 4.50 | 0 | 0 | 6 | 0 | 0 | 0 |
| 1/16/2005 | 72.49 | 5.69 | 0 | 0 | 128 | 0 | 0 | 0 |
| 1/17/2005 | 110.44 | 6.05 | 0 | 1 | 252 | 0 | 0 | 0 |
| 1/18/2005 | 159.71 | 7.10 | 0 | 0 | 392 | 0 | 0 | 0 |
| 1/19/2005 | 176.41 | 7.19 | 0 | 0 | 2 | 0 | 0 | 0 |
| 1/20/2005 | 137.34 | 7.22 | 0 | 0 | 0 | 0 | 0 | 0 |
| 1/21/2005 | 113.55 | 6.65 | 0 | 1 | 64 | 0 | 0 | 0 |
| 1/22/2005 | 97.13 | 5.95 | 0 | 0 | 17 | 0 | 0 | 0 |
| 1/23/2005 | 87.22 | 6.07 | 0 | 0 | 23 | 0 | 0 | 0 |
| 1/24/2005 | 81.55 | 6.21 | 0 | 0 | 19 | 0 | 0 | 0 |
| 1/25/2005 | 75.61 | 6.11 | 0 | 0 | 14 | 0 | 0 | 0 |
| 1/26/2005 | 71.64 | 6.47 | 0 | 0 | 91 | 0 | 0 | 0 |
| 1/27/2005 | 70.23 | 7.12 | 0 | 0 | 128 | 0 | 0 | 0 |
| 1/28/2005 | 65.98 | 7.14 | 0 | 0 | 48 | 0 | 0 | 0 |
| 1/29/2005 | 62.30 | 7.10 | 0 | 0 | 0 | 0 | 0 | 0 |
| 1/30/2005 | 58.62 | 6.41 | 0 | 0 | 0 | 0 | 0 | 0 |
| 1/31/2005 | 55.50 | 5.88 | 0 | 0 | 0 | 0 | 0 | 0 |
| 2/1/2005 | 52.67 | 5.81 | 0 | 0 | 0 | 0 | 0 | 0 |
| 2/2/2005 | 50.97 | 5.01 | 0 | 0 | 0 | 0 | 0 | 0 |
| 2/3/2005 | 48.99 | 4.70 | 0 | 0 | 0 | 0 | 0 | 0 |
| 2/4/2005 | 47.57 | 4.68 | 0 | 0 | 0 | 0 | 0 | 0 |
| 2/5/2005 | 46.72 | 5.28 | 0 | 0 | 1 | 0 | 0 | 0 |
| 2/6/2005 | 45.59 | 5.69 | 0 | 0 | 7 | 0 | 0 | 0 |
| 2/7/2005 | 47.01 | 5.72 | 0 | 0 | 2 | 0 | 0 | 0 |
| 2/8/2005 | 45.59 | 5.77 | 0 | 0 | 4 | 0 | 0 | 0 |
| 2/9/2005 | 44.46 | 4.99 | 0 | 0 | 0 | 0 | 0 | 0 |
| 2/10/2005 | 43.61 | 4.57 | 0 | 0 | 0 | 0 | 0 | 0 |
| 2/11/2005 | 43.04 | 4.42 | 0 | 0 | 0 | 0 | 0 | 0 |

| Date | Discharge (cms) | Temp (C°) | Chinook | Coho | Steelhead | Cutthroat | Lamprey | Sucker |
|---|---|---|---|---|---|---|---|---|
| 2/12/2005 | 42.76 | 4.50 | 0 | 0 | 1 | 0 | 0 | 0 |
| 2/13/2005 | 47.29 | 4.88 | 0 | 0 | 0 | 0 | 0 | 0 |
| 2/14/2005 | 50.12 | 5.17 | 0 | 0 | 2 | 0 | 0 | 0 |
| 2/15/2005 | 48.14 | 4.90 | 0 | 0 | 0 | 0 | 0 | 0 |
| 2/16/2005 | 45.59 | 4.44 | 0 | 0 | 0 | 0 | 0 | 0 |
| 2/17/2005 | 43.89 | | 0 | 0 | 0 | 0 | 0 | 0 |
| 2/18/2005 | 41.63 | 4.43 | 0 | 0 | 0 | 0 | 0 | 0 |
| 2/19/2005 | 41.06 | 5.29 | 0 | 0 | 0 | 0 | 0 | 0 |
| 2/20/2005 | 49.27 | 6.68 | 0 | 0 | 226 | 0 | 0 | 0 |
| 2/21/2005 | 51.54 | 7.22 | 0 | 0 | 397 | 0 | 0 | 0 |
| 2/22/2005 | 48.99 | 7.34 | 0 | 0 | 175 | 0 | 0 | 0 |
| 2/23/2005 | 46.44 | 7.07 | 0 | 0 | 39 | 0 | 0 | 0 |
| 2/24/2005 | 44.17 | 7.31 | 0 | 0 | 71 | 0 | 0 | 0 |
| 2/25/2005 | 42.48 | 7.27 | 0 | 0 | 192 | 0 | 0 | 0 |
| 2/26/2005 | 41.34 | | 0 | 0 | 187 | 0 | 0 | 0 |
| 2/27/2005 | 40.21 | | 0 | 0 | 187 | 0 | 0 | 0 |
| 2/28/2005 | 39.93 | 8.86 | 0 | 0 | 189 | 0 | 0 | 0 |
| 3/1/2005 | 39.93 | 8.04 | 0 | 0 | 66 | 0 | 0 | 0 |
| 3/2/2005 | 39.08 | 8.65 | 0 | 0 | 49 | 0 | 0 | 0 |
| 3/3/2005 | 38.23 | | 0 | 0 | 105 | 0 | 0 | 0 |
| 3/4/2005 | 37.66 | 8.97 | 0 | 0 | 169 | 0 | 0 | 0 |
| 3/5/2005 | 37.10 | 8.85 | 0 | 0 | 30 | 0 | 0 | 0 |
| 3/6/2005 | 36.53 | 9.51 | 0 | 0 | 97 | 0 | 0 | 0 |
| 3/7/2005 | 35.96 | 9.80 | 0 | 0 | 140 | 0 | 0 | 0 |
| 3/8/2005 | 35.40 | 10.13 | 0 | 0 | 122 | 0 | 0 | 0 |
| 3/9/2005 | 34.83 | 10.41 | 0 | 0 | 193 | 0 | 0 | 12 |
| 3/10/2005 | 34.83 | 10.87 | 0 | 0 | 106 | 0 | 0 | 12 |
| 3/11/2005 | 33.98 | 11.08 | 0 | 0 | 154 | 0 | 0 | 11 |
| 3/12/2005 | 33.70 | 11.60 | 0 | 0 | 96 | 0 | 0 | 16 |
| 3/13/2005 | 33.41 | 11.24 | 0 | 0 | 122 | 0 | 0 | 0 |
| 3/14/2005 | 33.13 | 10.37 | 1 | 0 | 2 | 0 | 0 | 0 |
| 3/15/2005 | 32.56 | | 0 | 0 | 123 | 0 | 0 | 0 |
| 3/16/2005 | 32.56 | 9.77 | 0 | 0 | 24 | 0 | 0 | 0 |
| 3/17/2005 | 33.41 | 9.21 | 0 | 0 | 37 | 0 | 0 | 0 |
| 3/18/2005 | 33.98 | 9.39 | 0 | 0 | 35 | 0 | 0 | 0 |
| 3/19/2005 | 34.26 | 10.13 | 0 | 0 | 150 | 0 | 0 | 5 |
| 3/20/2005 | 35.40 | 9.88 | 0 | 0 | 70 | 0 | 0 | 0 |
| 3/21/2005 | 36.81 | 9.51 | 1 | 0 | 90 | 0 | 0 | 0 |
| 3/22/2005 | 39.93 | 9.63 | 1 | 0 | 37 | 0 | 0 | 0 |
| 3/23/2005 | 41.91 | 9.99 | 0 | 0 | 148 | 0 | 0 | 53 |
| 3/24/2005 | 45.87 | 9.74 | 1 | 0 | 93 | 0 | 0 | 25 |
| 3/25/2005 | 46.72 | 9.33 | 5 | 0 | 26 | 0 | 0 | 0 |
| 3/26/2005 | 45.59 | 9.15 | 0 | 0 | 22 | 0 | 0 | 0 |
| 3/27/2005 | 111.00 | 8.65 | 1 | 0 | 228 | 0 | 0 | 9 |
| 3/28/2005 | 359.62 | 8.17 | 0 | 0 | 9 | 0 | 0 | 0 |

| Date | Discharge (cms) | Temp (C°) | Chinook | Coho | Steelhead | Cutthroat | Lamprey | Sucker |
|---|---|---|---|---|---|---|---|---|
| 3/29/2005 | 427.58 | 7.59 | 0 | 0 | 1 | 0 | 0 | 0 |
| 3/30/2005 | 305.82 | 7.65 | 0 | 0 | 9 | 0 | 0 | 0 |
| 3/31/2005 | 184.34 | 8.77 | 0 | 0 | 77 | 0 | 0 | 0 |
| 4/1/2005 | 171.32 | 8.65 | 1 | 0 | 97 | 0 | 0 | 2 |
| 4/2/2005 | 179.81 | 8.73 | 0 | 0 | 22 | 0 | 0 | 0 |
| 4/3/2005 | 157.72 | 8.33 | 0 | 0 | 31 | 1 | 0 | 0 |
| 4/4/2005 | 180.38 | 8.24 | 1 | 0 | 12 | 0 | 0 | 0 |
| 4/5/2005 | 168.20 | 8.54 | 0 | 0 | 21 | 0 | 0 | 0 |
| 4/6/2005 | 148.10 | 9.92 | 1 | 0 | 56 | 0 | 0 | 17 |
| 4/7/2005 | 167.92 | 9.73 | 0 | 0 | 79 | 0 | 0 | 41 |
| 4/8/2005 | 222.57 | 8.29 | 1 | 0 | 5 | 0 | 0 | 0 |
| 4/9/2005 | 217.76 | 8.60 | 1 | 0 | 3 | 0 | 0 | 0 |
| 4/10/2005 | 185.19 | 8.95 | 1 | 0 | 19 | 1 | 0 | 2 |
| 4/11/2005 | 165.65 | 10.22 | 1 | 0 | 52 | 0 | 0 | 0 |
| 4/12/2005 | 172.45 | 9.44 | 0 | 0 | 18 | 0 | 0 | 0 |
| 4/13/2005 | 156.59 | 8.18 | 0 | 0 | 5 | 1 | 0 | 0 |
| 4/14/2005 | 140.45 | 8.68 | 0 | 0 | 9 | 0 | 0 | 0 |
| 4/15/2005 | 127.71 | 8.33 | 0 | 0 | 16 | 1 | 0 | 0 |
| 4/16/2005 | 121.20 | 8.66 | 4 | 0 | 15 | 0 | 0 | 0 |
| 4/17/2005 | 162.26 | 8.88 | 2 | 0 | 19 | 0 | 0 | 1 |
| 4/18/2005 | 184.06 | 8.12 | 0 | 0 | 0 | 0 | 0 | 0 |
| 4/19/2005 | 163.39 | 8.54 | 0 | 0 | 0 | 0 | 0 | 0 |
| 4/20/2005 | 140.73 | 10.56 | 6 | 0 | 32 | 0 | 0 | 394 |
| 4/21/2005 | 126.01 | 11.42 | 10 | 0 | 53 | 1 | 0 | 807 |
| 4/22/2005 | 111.57 | 12.53 | 13 | 0 | 27 | 0 | 0 | 920 |
| 4/23/2005 | 110.44 | 11.62 | 53 | 0 | 24 | 0 | 0 | 976 |
| 4/24/2005 | 113.83 | 10.75 | 0 | 0 | 0 | 0 | 0 | 638 |
| 4/25/2005 | 108.17 | 11.97 | 0 | 0 | 0 | 0 | 0 | 0 |
| 4/26/2005 | 100.52 | 11.65 | 6 | 0 | 28 | 0 | 0 | 757 |
| 4/27/2005 | 95.14 | 13.50 | 115 | 0 | 19 | 1 | 0 | 847 |
| 4/28/2005 | 92.03 | 13.42 | 48 | 0 | 17 | 0 | 0 | 317 |
| 4/29/2005 | 90.61 | 12.62 | 43 | 0 | 11 | 0 | 0 | 36 |
| 4/30/2005 | 84.67 | 13.69 | 59 | 0 | 13 | 0 | 0 | 48 |
| 5/1/2005 | 79.57 | 13.61 | 75 | 0 | 19 | 0 | 0 | 232 |
| 5/2/2005 | 77.59 | 13.58 | 0 | 0 | 0 | 0 | 0 | 0 |
| 5/3/2005 | 75.32 | 13.60 | 13 | 0 | 2 | 0 | 0 | 158 |
| 5/4/2005 | 74.76 | 14.48 | 169 | 0 | 8 | 1 | 0 | 452 |
| 5/5/2005 | 78.72 | 13.54 | 80 | 0 | 7 | 1 | 1 | 139 |
| 5/6/2005 | 89.76 | 12.87 | 70 | 0 | 6 | 0 | 0 | 16 |
| 5/7/2005 | 113.27 | 12.53 | 32 | 0 | 2 | 0 | 0 | 9 |
| 5/8/2005 | 126.58 | 12.60 | 21 | 0 | 3 | 0 | 0 | 0 |
| 5/9/2005 | 236.16 | 11.09 | 1 | 0 | 0 | 0 | 1 | 0 |
| 5/10/2005 | 430.42 | 9.62 | 0 | 0 | 0 | 0 | 0 | 0 |
| 5/11/2005 | 376.61 | 10.03 | 0 | 0 | 0 | 0 | 0 | 0 |
| 5/12/2005 | 246.64 | 11.25 | 2 | 0 | 0 | 0 | 0 | 1 |

| Date | Discharge (cms) | Temp (C°) | Chinook | Coho | Steelhead | Cutthroat | Lamprey | Sucker |
|---|---|---|---|---|---|---|---|---|
| 5/13/2005 | 183.21 | 12.49 | 24 | 0 | 1 | 0 | 0 | 50 |
| 5/14/2005 | 150.65 | 13.57 | 66 | 0 | 4 | 0 | 0 | 155 |
| 5/15/2005 | 129.97 | 13.61 | 84 | 0 | 5 | 0 | 0 | 287 |
| 5/16/2005 | 135.64 | 12.97 | 0 | 0 | 0 | 0 | 0 | 0 |
| 5/17/2005 | 164.80 | 11.72 | 0 | 0 | 0 | 0 | 0 | 0 |
| 5/18/2005 | 195.67 | 10.72 | 3 | 0 | 0 | 0 | 0 | 19 |
| 5/19/2005 | 255.42 | 11.19 | 11 | 0 | 0 | 0 | 0 | 0 |
| 5/20/2005 | 201.05 | 11.31 | 14 | 0 | 2 | 0 | 0 | 0 |
| 5/21/2005 | 179.81 | 11.22 | 15 | 0 | 1 | 0 | 0 | 0 |
| 5/22/2005 | 152.91 | 11.73 | 0 | 0 | 0 | 0 | 0 | 0 |
| 5/23/2005 | 131.67 | 12.04 | 12 | 0 | 4 | 0 | 0 | 29 |
| 5/24/2005 | 114.97 | 12.64 | 31 | 0 | 0 | 0 | 0 | 0 |
| 5/25/2005 | 102.79 | 14.94 | 123 | 0 | 6 | 0 | 0 | 577 |
| 5/26/2005 | 92.03 | 15.19 | 197 | 0 | 6 | 2 | 2 | 528 |
| 5/27/2005 | 84.67 | 16.53 | 113 | 0 | 6 | 1 | 1 | 444 |
| 5/28/2005 | 84.67 | 17.73 | 151 | 0 | 5 | 0 | 0 | 84 |
| 5/29/2005 | 118.36 | 14.19 | 27 | 0 | 2 | 0 | 0 | 0 |
| 5/30/2005 | 124.03 | 13.85 | 0 | 0 | 0 | 0 | 0 | 0 |
| 5/31/2005 | 105.34 | 13.74 | 21 | 0 | 19 | 1 | 0 | 69 |
| 6/1/2005 | 91.75 | 13.90 | 55 | 0 | 25 | 0 | 0 | 54 |
| 6/2/2005 | 88.35 | 14.68 | 67 | 0 | 32 | 1 | 0 | 19 |
| 6/3/2005 | 82.40 | 14.20 | 135 | 0 | 19 | 0 | 0 | 51 |
| 6/4/2005 | 75.89 | 14.52 | 197 | 0 | 78 | 0 | 0 | 246 |
| 6/5/2005 | 71.36 | 14.41 | 138 | 0 | 70 | 0 | 0 | 201 |
| 6/6/2005 | 84.10 | 13.84 | 4 | 0 | 5 | 0 | 0 | 0 |
| 6/7/2005 | 119.21 | 12.19 | 33 | 0 | 48 | 0 | 0 | 2 |
| 6/8/2005 | 117.80 | 12.89 | 12 | 0 | 20 | 0 | 0 | 3 |
| 6/9/2005 | 106.75 | 14.06 | 35 | 0 | 35 | 0 | 0 | 95 |
| 6/10/2005 | 91.46 | 14.32 | 78 | 0 | 72 | 0 | 1 | 132 |
| 6/11/2005 | 81.84 | 14.67 | 50 | 0 | 64 | 0 | 0 | 71 |
| 6/12/2005 | 78.15 | 14.39 | 20 | 0 | 16 | 0 | 2 | 29 |
| 6/13/2005 | 73.34 | 15.38 | 10 | 0 | 27 | 0 | 1 | 16 |
| 6/14/2005 | 67.96 | 15.59 | 12 | 0 | 13 | 0 | 0 | 113 |
| 6/15/2005 | 63.15 | 15.65 | 47 | 0 | 117 | 1 | 0 | 114 |
| 6/16/2005 | 59.18 | 16.05 | 49 | 0 | 80 | 0 | 0 | 155 |
| 6/17/2005 | 60.60 | 14.66 | 39 | 0 | 77 | 0 | 0 | 25 |
| 6/18/2005 | 61.73 | 14.79 | 27 | 0 | 74 | 0 | 0 | 2 |
| 6/19/2005 | 60.60 | 14.95 | 28 | 0 | 67 | 0 | 0 | 57 |
| 6/20/2005 | 56.92 | 15.59 | 17 | 0 | 27 | 0 | 1 | 8 |
| 6/21/2005 | 50.97 | 17.67 | 11 | 0 | 14 | 1 | 0 | 194 |
| 6/22/2005 | 49.55 | 18.09 | 12 | 0 | 87 | 1 | 0 | 135 |
| 6/23/2005 | 48.42 | 17.77 | 34 | 0 | 107 | 0 | 3 | 131 |
| 6/24/2005 | 47.86 | 18.03 | 16 | 0 | 47 | 1 | 2 | 161 |
| 6/25/2005 | 47.01 | 19.19 | 26 | 0 | 85 | 0 | 0 | 406 |
| 6/26/2005 | 45.02 | 19.17 | 49 | 0 | 94 | 0 | 1 | 84 |

| Date | Discharge (cms) | Temp (C°) | Chinook | Coho | Steelhead | Cutthroat | Lamprey | Sucker |
|---|---|---|---|---|---|---|---|---|
| 6/27/2005 | 43.32 | 17.99 | 20 | 0 | 35 | 0 | 0 | 0 |
| 6/28/2005 | 42.48 | 17.98 | 0 | 0 | 0 | 0 | 0 | 0 |
| 6/29/2005 | 41.91 | 18.14 | 7 | 0 | 20 | 0 | 0 | 75 |
| 6/30/2005 | 40.78 | 19.84 | 21 | 0 | 101 | 1 | 0 | 86 |
| 7/1/2005 | 40.49 | 20.21 | 35 | 0 | 179 | 3 | 5 | 190 |
| 7/2/2005 | 40.49 | 19.01 | 16 | 0 | 78 | 4 | 3 | 17 |
| 7/3/2005 | 39.64 | 19.11 | 10 | 0 | 52 | 1 | 2 | 64 |
| 7/4/2005 | 38.23 | 20.30 | 19 | 0 | 64 | 2 | 4 | 262 |
| 7/5/2005 | 37.38 | 20.75 | 15 | 0 | 80 | 0 | 4 | 46 |
| 7/6/2005 | 36.25 | 21.39 | 23 | 0 | 96 | 1 | 0 | 27 |
| 7/7/2005 | 35.96 | 20.61 | 6 | 0 | 23 | 2 | 0 | 14 |
| 7/8/2005 | 35.68 | 20.60 | 4 | 0 | 35 | 0 | 2 | 34 |
| 7/9/2005 | 35.68 | 20.10 | 9 | 0 | 26 | 0 | 2 | 16 |
| 7/10/2005 | 35.96 | 19.62 | 3 | 0 | 29 | 0 | 1 | 36 |
| 7/11/2005 | 35.96 | 19.97 | 1 | 0 | 6 | 0 | 1 | 7 |
| 7/12/2005 | 34.83 | 20.90 | 1 | 0 | 0 | 0 | 1 | 1 |
| 7/13/2005 | 34.26 | 20.70 | 18 | 0 | 101 | 0 | 1 | 0 |
| 7/14/2005 | 33.70 | 21.02 | 12 | 0 | 116 | 0 | 0 | 1 |
| 7/15/2005 | 32.56 | 22.09 | 17 | 0 | 92 | 1 | 0 | 5 |
| 7/16/2005 | 31.71 | 22.51 | 6 | 0 | 86 | 3 | 2 | 1 |
| 7/17/2005 | 31.43 | 22.67 | 19 | 0 | 47 | 0 | 0 | 1 |
| 7/18/2005 | 31.15 | 23.46 | 9 | 0 | 34 | 3 | 0 | 1 |
| 7/19/2005 | 30.30 | 24.00 | 5 | 0 | 19 | 2 | 0 | 3 |
| 7/20/2005 | 29.73 | 23.46 | 3 | 0 | 1 | 1 | 2 | 0 |
| 7/21/2005 | 29.45 | 23.15 | 1 | 0 | 5 | 1 | 0 | 0 |
| 7/22/2005 | 28.88 | 22.94 | 0 | 0 | 1 | 1 | 0 | 0 |
| 7/23/2005 | 28.88 | 22.72 | 0 | 0 | 3 | 3 | 0 | 0 |
| 7/24/2005 | 28.32 | 22.81 | 0 | 0 | 0 | 1 | 0 | 0 |
| 7/25/2005 | 27.69 | 22.41 | 0 | 0 | 0 | 0 | 0 | 0 |
| 7/26/2005 | 27.30 | 22.46 | 0 | 0 | 4 | 0 | 0 | 0 |
| 7/27/2005 | 27.30 | 23.07 | 1 | 0 | 0 | 0 | 1 | 0 |
| 7/28/2005 | 26.84 | 23.81 | 0 | 0 | 2 | 0 | 0 | 0 |
| 7/29/2005 | 26.25 | 23.86 | 0 | 0 | 0 | 0 | 0 | 0 |
| 7/30/2005 | 26.11 | 23.59 | 0 | 0 | 1 | 0 | 0 | 0 |
| 7/31/2005 | 25.74 | 23.69 | 0 | 0 | 1 | 0 | 0 | 2 |
| 8/1/2005 | 25.37 | 23.32 | 0 | 0 | 1 | 0 | 0 | 0 |
| 8/2/2005 | 24.89 | | 0 | 0 | 0 | 0 | 0 | 0 |
| 8/3/2005 | 24.75 | 22.22 | 0 | 0 | 0 | 0 | 0 | 0 |
| 8/4/2005 | 24.52 | 22.85 | 0 | 0 | 1 | 0 | 0 | 0 |
| 8/5/2005 | 24.44 | 23.51 | 1 | 0 | 0 | 0 | 1 | 0 |
| 8/6/2005 | 24.41 | 23.80 | 0 | 0 | 1 | 1 | 0 | 0 |
| 8/7/2005 | 24.18 | 23.72 | 0 | 0 | 0 | 0 | 0 | 0 |
| 8/8/2005 | 23.64 | 23.22 | 0 | 0 | 1 | 0 | 0 | 0 |
| 8/9/2005 | 22.85 | 23.44 | 0 | 0 | 0 | 0 | 0 | 0 |
| 8/10/2005 | 22.57 | 23.10 | 0 | 0 | 0 | 0 | 0 | 0 |

| Date | Discharge (cms) | Temp (C°) | Chinook | Coho | Steelhead | Cutthroat | Lamprey | Sucker |
|---|---|---|---|---|---|---|---|---|
| 8/11/2005 | 22.54 | 22.64 | 0 | 0 | 0 | 0 | 0 | 0 |
| 8/12/2005 | 22.46 | 22.43 | 1 | 0 | 0 | 0 | 0 | 0 |
| 8/13/2005 | 22.29 | 22.55 | 0 | 0 | 0 | 0 | 0 | 0 |
| 8/14/2005 | 22.14 | 22.80 | 0 | 0 | 0 | 0 | 0 | 0 |
| 8/15/2005 | 21.92 | 22.70 | 0 | 0 | 0 | 0 | 0 | 0 |
| 8/16/2005 | 21.83 | 22.40 | 0 | 0 | 1 | 0 | 0 | 0 |
| 8/17/2005 | 21.78 | 22.36 | 0 | 0 | 2 | 0 | 0 | 0 |
| 8/18/2005 | 21.80 | 22.02 | 0 | 0 | 0 | 1 | 0 | 0 |
| 8/19/2005 | 21.69 | 21.73 | 1 | 0 | 0 | 0 | 1 | 0 |
| 8/20/2005 | 21.69 | 22.05 | 2 | 0 | 0 | 0 | 0 | 0 |
| 8/21/2005 | 21.44 | 22.24 | 3 | 0 | 1 | 0 | 0 | 0 |
| 8/22/2005 | 21.15 | 22.05 | 8 | 0 | 1 | 0 | 0 | 0 |
| 8/23/2005 | 20.98 | 21.50 | 5 | 0 | 5 | 0 | 0 | 0 |
| 8/24/2005 | 20.56 | 20.65 | 9 | 0 | 7 | 0 | 0 | 0 |
| 8/25/2005 | 20.64 | 20.87 | 5 | 0 | 10 | 0 | 0 | 0 |
| 8/26/2005 | 20.44 | 21.49 | 9 | 0 | 11 | 0 | 0 | 0 |
| 8/27/2005 | 20.47 | 20.82 | 8 | 0 | 15 | 0 | 0 | 0 |
| 8/28/2005 | 20.25 | 20.82 | 10 | 0 | 13 | 0 | 0 | 0 |
| 8/29/2005 | 20.36 | 20.42 | 12 | 0 | 16 | 0 | 0 | 0 |
| 8/30/2005 | 20.47 | 19.61 | 1 | 0 | 2 | 0 | 0 | 0 |
| 8/31/2005 | 20.64 | 19.42 | 10 | 0 | 26 | 0 | 0 | 0 |
| 9/1/2005 | 20.73 | 19.64 | 11 | 0 | 11 | 0 | 0 | 0 |
| 9/2/2005 | 20.76 | 19.85 | 12 | 0 | 22 | 0 | 0 | 0 |
| 9/3/2005 | 20.70 | 20.06 | 14 | 0 | 14 | 1 | 0 | 0 |
| 9/4/2005 | 20.93 | 19.38 | 30 | 0 | 17 | 0 | 0 | 0 |
| 9/5/2005 | 20.87 | 19.08 | 15 | 0 | 9 | 1 | 0 | 0 |
| 9/6/2005 | 20.70 | 18.66 | 26 | 1 | 19 | 0 | 0 | 0 |
| 9/7/2005 | 21.12 | 18.54 | 10 | 1 | 12 | 0 | 0 | 0 |
| 9/8/2005 | 21.89 | 18.65 | 7 | 4 | 4 | 0 | 0 | 0 |
| 9/9/2005 | 22.65 | 18.42 | 9 | 0 | 13 | 1 | 0 | 0 |
| 9/10/2005 | 23.76 | 17.44 | 18 | 0 | 14 | 0 | 0 | 0 |
| 9/11/2005 | 25.03 | 16.53 | 5 | 1 | 5 | 1 | 0 | 0 |
| 9/12/2005 | 25.12 | 16.30 | 2 | 1 | 10 | 0 | 0 | 0 |
| 9/13/2005 | 24.13 | 16.33 | 5 | 1 | 16 | 0 | 0 | 0 |
| 9/14/2005 | 23.70 | 16.59 | 2 | 0 | 8 | 0 | 0 | 0 |
| 9/15/2005 | 23.53 | 16.75 | 4 | 0 | 5 | 1 | 0 | 0 |
| 9/16/2005 | 23.56 | 16.05 | 7 | 1 | 24 | 0 | 0 | 0 |
| 9/17/2005 | 23.93 | 16.11 | 9 | 0 | 30 | 0 | 0 | 0 |
| 9/18/2005 | 24.66 | 16.03 | 6 | 0 | 11 | 0 | 0 | 0 |
| 9/19/2005 | 24.18 | 15.99 | 1 | 0 | 3 | 0 | 0 | 0 |
| 9/20/2005 | 23.64 | 16.07 | 0 | 0 | 0 | 0 | 0 | 0 |
| 9/21/2005 | 23.96 | 15.67 | 5 | 1 | 15 | 1 | 0 | 0 |
| 9/22/2005 | 24.04 | 15.39 | 1 | 3 | 8 | 1 | 0 | 0 |
| 9/23/2005 | 24.15 | 15.09 | 3 | 5 | 7 | 0 | 0 | 0 |
| 9/24/2005 | 24.01 | 14.69 | 5 | 9 | 3 | 0 | 0 | 0 |

| Date | Discharge (cms) | Temp (C°) | Chinook | Coho | Steelhead | Cutthroat | Lamprey | Sucker |
|---|---|---|---|---|---|---|---|---|
| 9/25/2005 | 24.07 | 14.27 | 1 | 4 | 6 | 0 | 0 | 0 |
| 9/26/2005 | 24.04 | 14.11 | 4 | 7 | 13 | 0 | 0 | 0 |
| 9/27/2005 | 24.32 | 14.40 | 5 | 10 | 18 | 0 | 0 | 0 |
| 9/28/2005 | 24.95 | 14.59 | 7 | 14 | 11 | 0 | 0 | 0 |
| 9/29/2005 | 25.15 | 14.60 | 13 | 18 | 9 | 0 | 0 | 0 |
| 9/30/2005 | 25.17 | 15.00 | 15 | 16 | 31 | 1 | 0 | 0 |
| 10/1/2005 | 27.81 | 15.00 | 48 | 43 | 39 | 0 | 0 | 0 |
| 10/2/2005 | 31.43 | 13.43 | 30 | 44 | 28 | 0 | 0 | 0 |
| 10/3/2005 | 30.58 | 12.61 | 2 | 6 | 3 | 0 | 0 | 0 |
| 10/4/2005 | 33.13 | 12.47 | 0 | 0 | 0 | 0 | 0 | 0 |
| 10/5/2005 | 33.13 | 12.52 | 2 | 9 | 2 | 0 | 0 | 0 |
| 10/6/2005 | 31.43 | 12.13 | 0 | 6 | 0 | 0 | 0 | 0 |
| 10/7/2005 | 30.30 | 12.97 | 2 | 70 | 52 | 1 | 0 | 0 |
| 10/8/2005 | 29.73 | 12.61 | 3 | 42 | 13 | 0 | 0 | 0 |
| 10/9/2005 | 28.88 | 13.26 | 3 | 22 | 5 | 0 | 0 | 0 |
| 10/10/2005 | 28.01 | 13.54 | 1 | 4 | 0 | 0 | 0 | 0 |
| 10/11/2005 | 26.53 | 13.18 | 5 | 21 | 3 | 0 | 0 | 0 |
| 10/12/2005 | 26.48 | 12.74 | 0 | 71 | 26 | 0 | 0 | 0 |
| 10/13/2005 | 26.50 | 12.88 | 0 | 28 | 4 | 0 | 0 | 0 |
| 10/14/2005 | 26.70 | 12.97 | 3 | 23 | 9 | 0 | 0 | 0 |
| 10/15/2005 | 29.17 | 12.61 | 8 | 75 | 23 | 0 | 0 | 0 |
| 10/16/2005 | 32.28 | 12.19 | 8 | 89 | 12 | 0 | 0 | 0 |
| 10/17/2005 | 32.56 | 12.35 | 1 | 19 | 4 | 0 | 0 | 0 |
| 10/18/2005 | 28.60 | 12.91 | 3 | 6 | 0 | 0 | 0 | 1 |
| 10/19/2005 | 26.84 | 13.25 | 4 | 121 | 19 | 0 | 0 | 0 |
| 10/20/2005 | 26.50 | 12.88 | 3 | 41 | 11 | 0 | 0 | 0 |
| 10/21/2005 | 26.31 | 12.91 | 1 | 15 | 4 | 0 | 0 | 0 |
| 10/22/2005 | 26.19 | 12.70 | 0 | 9 | 4 | 0 | 0 | 0 |
| 10/23/2005 | 25.91 | 12.28 | 1 | 8 | 8 | 0 | 0 | 0 |
| 10/24/2005 | 25.80 | 12.15 | 0 | 3 | 1 | 0 | 0 | 0 |
| 10/25/2005 | 25.74 | 12.32 | 0 | 0 | 0 | 0 | 0 | 0 |
| 10/26/2005 | 26.31 | 11.87 | 0 | 0 | 0 | 0 | 0 | 0 |
| 10/27/2005 | 27.16 | 10.84 | 0 | 3 | 0 | 0 | 0 | 0 |
| 10/28/2005 | 27.61 | 10.49 | 3 | 39 | 11 | 0 | 0 | 0 |
| 10/29/2005 | 30.30 | 10.22 | 0 | 30 | 6 | 0 | 0 | 0 |
| 10/30/2005 | 30.02 | 10.25 | 2 | 24 | 16 | 0 | 0 | 0 |
| 10/31/2005 | 28.15 | 10.46 | 2 | 4 | 0 | 0 | 0 | 0 |
| 11/1/2005 | 28.06 | 11.25 | 0 | 3 | 5 | 1 | 0 | 0 |
| 11/2/2005 | 32.85 | 10.47 | 2 | 9 | 0 | 0 | 0 | 0 |
| 11/3/2005 | 35.11 | 9.78 | 7 | 109 | 38 | 0 | 0 | 0 |
| 11/4/2005 | 71.64 | 9.24 | 5 | 194 | 31 | 0 | 0 | 0 |
| 11/5/2005 | 78.44 | 8.61 | 1 | 261 | 14 | 0 | 0 | 0 |
| 11/6/2005 | 334.14 | 8.87 | 0 | 30 | 0 | 0 | 0 | 0 |
| 11/7/2005 | 211.81 | 8.88 | 0 | 0 | 0 | 0 | 0 | 0 |
| 11/8/2005 | 118.08 | 8.23 | 0 | 101 | 3 | 0 | 0 | 0 |

| Date | Discharge (cms) | Temp (C°) | Chinook | Coho | Steelhead | Cutthroat | Lamprey | Sucker |
|---|---|---|---|---|---|---|---|---|
| 11/9/2005 | 82.12 | 7.17 | 0 | 0 | 0 | 0 | 0 | 0 |
| 11/10/2005 | 64.00 | 6.70 | 1 | 28 | 11 | 0 | 0 | 0 |
| 11/11/2005 | 57.48 | 7.35 | 0 | 123 | 18 | 0 | 0 | 0 |
| 11/12/2005 | 62.30 | 7.79 | 0 | 126 | 18 | 0 | 0 | 0 |
| 11/13/2005 | 161.69 | 8.26 | 1 | 177 | 11 | 0 | 0 | 0 |
| 11/14/2005 | 237.30 | 9.30 | 0 | 112 | 15 | 0 | 0 | 0 |
| 11/15/2005 | 149.80 | 8.60 | 2 | 75 | 10 | 0 | 0 | 0 |
| 11/16/2005 | 102.51 | 7.48 | 1 | 15 | 3 | 0 | 0 | 0 |
| 11/17/2005 | 78.15 | 7.03 | 0 | 3 | 2 | 0 | 0 | 0 |
| 11/18/2005 | 65.98 | 6.89 | 0 | 1 | 4 | 0 | 0 | 0 |
| 11/19/2005 | 58.05 | 6.32 | 0 | 0 | 1 | 0 | 0 | 0 |
| 11/20/2005 | 52.95 | 5.89 | 0 | 0 | 2 | 0 | 0 | 0 |
| 11/21/2005 | 49.55 | 5.77 | 0 | 1 | 1 | 0 | 0 | 0 |
| 11/22/2005 | 47.01 | 5.80 | 0 | 3 | 0 | 0 | 0 | 0 |
| 11/23/2005 | 44.46 | 6.01 | 0 | 2 | 1 | 0 | 0 | 0 |
| 11/24/2005 | 42.76 | 6.00 | 0 | 0 | 1 | 0 | 0 | 0 |
| 11/25/2005 | 47.01 | 6.18 | 0 | 1 | 0 | 0 | 0 | 0 |
| 11/26/2005 | 96.28 | 6.67 | 0 | 3 | 8 | 0 | 0 | 0 |
| 11/27/2005 | 88.35 | 6.79 | 0 | 9 | 7 | 0 | 0 | 0 |
| 11/28/2005 | 82.69 | 5.91 | 0 | 6 | 2 | 0 | 0 | 0 |
| 11/29/2005 | 158.86 | 6.03 | 0 | 1 | 4 | 0 | 0 | 0 |
| 11/30/2005 | 163.11 | 6.48 | 0 | 11 | 26 | 0 | 0 | 0 |
| 12/1/2005 | 365.29 | 6.56 | 0 | 0 | 2 | 0 | 0 | 0 |
| 12/2/2005 | 461.56 | 7.09 | 0 | 0 | 0 | 0 | 0 | 0 |
| 12/3/2005 | 269.58 | 6.27 | 0 | 0 | 0 | 0 | 0 | 0 |
| 12/4/2005 | 181.23 | 6.21 | 0 | 0 | 0 | 0 | 0 | 0 |
| 12/5/2005 | 134.22 | 5.79 | 0 | 4 | 16 | 0 | 0 | 0 |
| 12/6/2005 | 112.98 | 4.91 | 0 | 2 | 3 | 0 | 0 | 0 |
| 12/7/2005 | 103.92 | 4.82 | 0 | 0 | 2 | 0 | 0 | 0 |
| 12/8/2005 | 127.43 | 5.31 | 0 | 1 | 20 | 0 | 0 | 0 |
| 12/9/2005 | 140.45 | 5.71 | 0 | 2 | 8 | 0 | 0 | 0 |
| 12/10/2005 | 134.51 | 5.58 | 0 | 0 | 11 | 0 | 0 | 0 |
| 12/11/2005 | 142.72 | 5.13 | 0 | 0 | 1 | 0 | 0 | 0 |
| 12/12/2005 | 136.49 | 5.14 | 0 | 0 | 2 | 0 | 0 | 0 |
| 12/13/2005 | 118.08 | 5.07 | 0 | 0 | 5 | 0 | 0 | 0 |
| 12/14/2005 | 102.22 | 4.79 | 0 | 0 | 0 | 0 | 0 | 0 |
| 12/15/2005 | 91.46 | 3.35 | 0 | 0 | 0 | 0 | 0 | 0 |
| 12/16/2005 | 82.69 | 2.83 | 0 | 0 | 0 | 0 | 0 | 0 |
| 12/17/2005 | 74.19 | 2.53 | 0 | 0 | 0 | 0 | 0 | 0 |
| 12/18/2005 | 68.53 | 2.32 | 0 | 0 | 1 | 0 | 0 | 0 |
| 12/19/2005 | 75.61 | 3.37 | 0 | 0 | 3 | 0 | 0 | 0 |
| 12/20/2005 | 175.00 | 5.59 | 0 | 0 | 3 | 0 | 0 | 0 |
| 12/21/2005 | 172.17 | 7.27 | 0 | 3 | 56 | 0 | 0 | 0 |
| 12/22/2005 | 233.61 | 7.46 | 0 | 0 | 29 | 1 | 0 | 0 |
| 12/23/2005 | 314.32 | 7.77 | 0 | 0 | 11 | 0 | 0 | 0 |

| Date | Discharge (cms) | Temp (C°) | Chinook | Coho | Steelhead | Cutthroat | Lamprey | Sucker |
|---|---|---|---|---|---|---|---|---|
| 12/24/2005 | 209.26 | 7.82 | 0 | 0 | 38 | 0 | 0 | 0 |
| 12/25/2005 | 168.20 | 7.97 | 0 | 0 | 48 | 1 | 0 | 0 |
| 12/26/2005 | 156.59 | 8.06 | 0 | 0 | 27 | 0 | 0 | 0 |
| 12/27/2005 | 264.20 | 7.81 | 0 | 0 | 23 | 0 | 0 | 0 |
| 12/28/2005 | 770.22 | 8.50 | 0 | 0 | 0 | 0 | 0 | 0 |
| 12/29/2005 | 648.46 | 7.94 | 0 | 0 | 1 | 0 | 0 | 0 |
| 12/30/2005 | 1098.69 | 8.24 | 0 | 0 | 1 | 0 | 0 | 0 |
| 12/31/2005 | 1438.50 | 8.78 | 0 | 0 | 0 | 0 | 0 | 0 |
| 1/1/2006 | 838.18 | 8.01 | 0 | 0 | 0 | 0 | 0 | 0 |
| 1/2/2006 | 492.71 | 7.85 | 0 | 0 | 0 | 0 | 0 | 0 |
| 1/3/2006 | 348.30 | 7.47 | 0 | 0 | 0 | 0 | 0 | 0 |
| 1/4/2006 | 273.54 | 6.79 | 0 | 0 | 0 | 0 | 0 | 0 |
| 1/5/2006 | 221.44 | 6.91 | 0 | 0 | 21 | 0 | 0 | 0 |
| 1/6/2006 | 192.27 | 7.19 | 0 | 0 | 49 | 0 | 0 | 0 |
| 1/7/2006 | 193.97 | 7.38 | 0 | 0 | 39 | 0 | 0 | 0 |
| 1/8/2006 | 274.39 | 7.06 | 0 | 0 | 3 | 0 | 0 | 0 |
| 1/9/2006 | 250.04 | 6.84 | 0 | 0 | 10 | 0 | 0 | 0 |
| 1/10/2006 | 605.98 | 7.68 | 0 | 0 | 1 | 0 | 0 | 0 |
| 1/11/2006 | 991.09 | 8.47 | 0 | 0 | 0 | 0 | 0 | 0 |
| 1/12/2006 | 546.52 | 7.48 | 0 | 0 | 0 | 0 | 0 | 0 |
| 1/13/2006 | 461.56 | 7.83 | 0 | 0 | 34 | 0 | 0 | 0 |
| 1/14/2006 | 569.17 | 8.05 | 0 | 0 | 21 | 0 | 0 | 0 |
| 1/15/2006 | 472.89 | 6.86 | 0 | 0 | 6 | 0 | 0 | 0 |
| 1/16/2006 | 351.13 | 6.46 | 0 | 0 | 5 | 0 | 0 | 0 |
| 1/17/2006 | 563.51 | 6.98 | 0 | 0 | 1 | 0 | 0 | 0 |
| 1/18/2006 | 883.49 | 7.33 | 0 | 0 | 0 | 0 | 0 | 0 |
| 1/19/2006 | 501.21 | 7.25 | 0 | 0 | 3 | 0 | 0 | 0 |
| 1/20/2006 | 385.11 | 7.24 | 0 | 0 | 16 | 0 | 0 | 0 |
| 1/21/2006 | 359.62 | 6.82 | 0 | 0 | 44 | 0 | 0 | 0 |
| 1/22/2006 | 275.52 | 6.75 | 0 | 0 | 22 | 0 | 0 | 0 |
| 1/23/2006 | 216.34 | 5.88 | 0 | 0 | 7 | 0 | 0 | 0 |
| 1/24/2006 | 191.99 | 6.10 | 0 | 0 | 40 | 0 | 0 | 0 |
| 1/25/2006 | 192.55 | 5.69 | 0 | 0 | 23 | 0 | 0 | 0 |
| 1/26/2006 | 184.06 | 6.01 | 0 | 0 | 40 | 0 | 0 | 0 |
| 1/27/2006 | 178.96 | 6.17 | 0 | 0 | 66 | 0 | 0 | 0 |
| 1/28/2006 | 256.27 | 6.23 | 0 | 0 | 7 | 0 | 0 | 0 |
| 1/29/2006 | 331.31 | 6.60 | 0 | 0 | 17 | 0 | 0 | 0 |
| 1/30/2006 | 470.06 | 7.32 | 0 | 0 | 26 | 0 | 0 | 0 |
| 1/31/2006 | 526.69 | 6.87 | 0 | 0 | 19 | 0 | 0 | 0 |
| 2/1/2006 | 685.27 | 6.74 | 0 | 0 | 2 | 0 | 0 | 0 |
| 2/2/2006 | 453.07 | | 0 | 0 | 53 | 0 | 0 | 0 |
| 2/3/2006 | 328.48 | 8.02 | 0 | 0 | 119 | 0 | 0 | 0 |
| 2/4/2006 | 382.28 | 7.79 | 0 | 0 | 106 | 0 | 0 | 0 |
| 2/5/2006 | 351.13 | 6.38 | 0 | 0 | 54 | 0 | 0 | 0 |
| 2/6/2006 | 267.03 | 6.31 | 0 | 0 | 8 | 0 | 0 | 0 |

| Date | Discharge (cms) | Temp (C°) | Chinook | Coho | Steelhead | Cutthroat | Lamprey | Sucker |
|---|---|---|---|---|---|---|---|---|
| 2/7/2006 | 217.47 | 6.63 | 0 | 0 | 53 | 0 | 0 | 0 |
| 2/8/2006 | 195.95 | 6.70 | 0 | 0 | 19 | 0 | 0 | 0 |
| 2/9/2006 | 184.63 | 6.88 | 0 | 0 | 90 | 0 | 0 | 0 |
| 2/10/2006 | 175.56 | 6.57 | 0 | 0 | 21 | 0 | 0 | 0 |
| 2/11/2006 | 159.71 | 6.41 | 0 | 0 | 11 | 0 | 0 | 0 |
| 2/12/2006 | 148.10 | 6.93 | 0 | 0 | 89 | 0 | 0 | 0 |
| 2/13/2006 | 137.62 | 6.26 | 0 | 0 | 54 | 0 | 0 | 0 |
| 2/14/2006 | 126.29 | 5.83 | 0 | 0 | 0 | 0 | 0 | 0 |
| 2/15/2006 | 120.63 | 5.37 | 0 | 0 | 5 | 0 | 0 | 0 |
| 2/16/2006 | 111.29 | 4.21 | 0 | 0 | 0 | 0 | 0 | 0 |
| 2/17/2006 | 103.36 | 3.49 | 0 | 0 | 0 | 0 | 0 | 0 |
| 2/18/2006 | 94.01 | 3.16 | 0 | 0 | 0 | 0 | 0 | 0 |
| 2/19/2006 | 88.63 | 2.87 | 0 | 0 | 0 | 0 | 0 | 0 |
| 2/20/2006 | 83.82 | 2.53 | 0 | 0 | 0 | 0 | 0 | 0 |
| 2/21/2006 | 79.29 | 3.33 | 0 | 0 | 0 | 0 | 0 | 0 |
| 2/22/2006 | 76.17 | 4.22 | 0 | 0 | 1 | 0 | 0 | 0 |
| 2/23/2006 | 74.76 | 4.83 | 0 | 0 | 3 | 0 | 0 | 0 |
| 2/24/2006 | 73.06 | 5.50 | 0 | 0 | 6 | 0 | 0 | 0 |
| 2/25/2006 | 67.96 | 5.44 | 0 | 0 | 8 | 0 | 0 | 0 |
| 2/26/2006 | 67.11 | 6.05 | 0 | 0 | 53 | 0 | 0 | 0 |
| 2/27/2006 | 84.38 | 6.72 | 0 | 0 | 756 | 0 | 0 | 0 |
| 2/28/2006 | 259.67 | 7.36 | 0 | 0 | 70 | 0 | 0 | 0 |
| 3/1/2006 | 250.04 | 7.23 | 0 | 0 | 54 | 0 | 0 | 0 |
| 3/2/2006 | 185.76 | 7.10 | 0 | 0 | 242 | 0 | 0 | 0 |
| 3/3/2006 | 147.53 | 6.46 | 0 | 0 | 100 | 0 | 0 | 0 |
| 3/4/2006 | 127.99 | 6.27 | 0 | 0 | 18 | 0 | 0 | 0 |
| 3/5/2006 | 115.25 | 7.04 | 0 | 0 | 102 | 0 | 0 | 0 |
| 3/6/2006 | 105.06 | 6.71 | 0 | 0 | 113 | 0 | 0 | 0 |
| 3/7/2006 | 107.04 | | 0 | 0 | 100 | 0 | 0 | 0 |
| 3/8/2006 | 121.48 | 6.50 | 0 | 0 | 48 | 0 | 0 | 0 |
| 3/9/2006 | 201.62 | 5.48 | 0 | 0 | 6 | 0 | 0 | 0 |
| 3/10/2006 | 183.78 | 4.88 | 0 | 0 | 0 | 0 | 0 | 0 |
| 3/11/2006 | 145.27 | 5.14 | 0 | 0 | 0 | 0 | 0 | 0 |
| 3/12/2006 | 123.46 | 5.04 | 0 | 0 | 0 | 0 | 0 | 0 |
| 3/13/2006 | 109.87 | 5.09 | 0 | 0 | 2 | 0 | 0 | 0 |
| 3/14/2006 | 103.07 | 5.32 | 0 | 0 | 6 | 0 | 0 | 0 |
| 3/15/2006 | 101.94 | 6.19 | 0 | 0 | 15 | 0 | 0 | 0 |
| 3/16/2006 | 112.70 | 6.57 | 0 | 0 | 71 | 0 | 0 | 0 |
| 3/17/2006 | 146.96 | 6.71 | 0 | 0 | 101 | 0 | 0 | 0 |
| 3/18/2006 | 143.00 | 7.41 | 0 | 0 | 253 | 0 | 0 | 0 |
| 3/19/2006 | 129.69 | 7.55 | 0 | 0 | 70 | 0 | 0 | 0 |
| 3/20/2006 | 119.21 | 6.35 | 0 | 0 | 9 | 0 | 0 | 0 |
| 3/21/2006 | 112.70 | 6.62 | 0 | 0 | 18 | 0 | 0 | 0 |
| 3/22/2006 | 121.48 | 7.41 | 1 | 0 | 222 | 0 | 0 | 1 |
| 3/23/2006 | 127.99 | 8.08 | 0 | 0 | 263 | 0 | 0 | 3 |

| Date | Discharge (cms) | Temp (C°) | Chinook | Coho | Steelhead | Cutthroat | Lamprey | Sucker |
|---|---|---|---|---|---|---|---|---|
| 3/24/2006 | 145.55 | 8.08 | 0 | 0 | 181 | 1 | 0 | 0 |
| 3/25/2006 | 162.82 | 8.05 | 0 | 0 | 213 | 0 | 0 | 4 |
| 3/26/2006 | 150.93 | 8.01 | 0 | 0 | 25 | 0 | 0 | 0 |
| 3/27/2006 | 133.94 | 7.59 | 0 | 0 | 26 | 0 | 0 | 0 |
| 3/28/2006 | 126.86 | 8.59 | 0 | 0 | 92 | 0 | 0 | 0 |
| 3/29/2006 | 146.11 | 8.77 | 0 | 0 | 177 | 0 | 0 | 3 |
| 3/30/2006 | 138.47 | 8.21 | 1 | 0 | 91 | 0 | 0 | 2 |
| 3/31/2006 | 136.49 | 8.17 | 0 | 0 | 62 | 0 | 0 | 0 |
| 4/1/2006 | 150.08 | 8.33 | 0 | 0 | 58 | 0 | 0 | 0 |
| 4/2/2006 | 142.15 | 8.00 | 0 | 0 | 36 | 0 | 0 | 0 |
| 4/3/2006 | 134.51 | 8.69 | 0 | 0 | 80 | 0 | 0 | 0 |
| 4/4/2006 | 138.75 | 9.42 | 0 | 0 | 114 | 0 | 0 | 3 |
| 4/5/2006 | 130.54 | 8.44 | 0 | 0 | 51 | 0 | 0 | 3 |
| 4/6/2006 | 127.71 | 9.06 | 0 | 0 | 36 | 1 | 0 | 0 |
| 4/7/2006 | 125.73 | 9.39 | 0 | 0 | 61 | 0 | 0 | 0 |
| 4/8/2006 | 119.78 | 9.95 | 0 | 0 | 130 | 1 | 0 | 114 |
| 4/9/2006 | 123.46 | 9.59 | 1 | 0 | 110 | 0 | 0 | 181 |
| 4/10/2006 | 136.49 | 9.67 | 0 | 0 | 85 | 0 | 0 | 32 |
| 4/11/2006 | 141.87 | 9.08 | 0 | 0 | 26 | 0 | 0 | 28 |
| 4/12/2006 | 131.11 | 9.92 | 0 | 0 | 31 | 0 | 0 | 32 |
| 4/13/2006 | 124.88 | 10.75 | 2 | 0 | 68 | 0 | 0 | 279 |
| 4/14/2006 | 132.24 | 10.24 | 4 | 0 | 63 | 0 | 0 | 50 |
| 4/15/2006 | 173.87 | 8.58 | 2 | 0 | 11 | 0 | 0 | 0 |
| 4/16/2006 | 198.50 | 7.56 | 0 | 0 | 3 | 0 | 0 | 0 |
| 4/17/2006 | 177.83 | 7.42 | 0 | 0 | 1 | 0 | 0 | 0 |
| 4/18/2006 | 156.31 | 8.91 | 1 | 0 | 17 | 0 | 0 | 0 |
| 4/19/2006 | 139.60 | 9.89 | 0 | 0 | 17 | 0 | 0 | 0 |
| 4/20/2006 | 134.22 | 11.01 | 2 | 0 | 43 | 0 | 0 | 170 |
| 4/21/2006 | 138.19 | 10.43 | 7 | 0 | 45 | 0 | 0 | 241 |
| 4/22/2006 | 144.70 | 9.96 | 0 | 0 | 2 | 0 | 0 | 41 |
| 4/23/2006 | 131.67 | 10.30 | 1 | 0 | 2 | 0 | 0 | 43 |
| 4/24/2006 | 121.20 | 11.68 | 2 | 0 | 31 | 0 | 0 | 330 |
| 4/25/2006 | 113.27 | 11.97 | 7 | 0 | 19 | 0 | 0 | 112 |
| 4/26/2006 | 111.00 | 10.87 | 4 | 0 | 17 | 0 | 0 | 45 |
| 4/27/2006 | 114.40 | 12.30 | 8 | 0 | 8 | 0 | 0 | 154 |
| 4/28/2006 | 116.38 | 12.98 | 0 | 0 | 23 | 0 | 0 | 270 |
| 4/29/2006 | 134.51 | 12.69 | 33 | 0 | 23 | 0 | 0 | 665 |
| 4/30/2006 | 142.43 | 11.76 | 3 | 0 | 5 | 0 | 0 | 92 |
| 5/1/2006 | 129.41 | 10.83 | 0 | 0 | 0 | 0 | 0 | 0 |
| 5/2/2006 | 113.83 | 10.72 | 0 | 0 | 0 | 0 | 0 | 0 |
| 5/3/2006 | 105.06 | 10.88 | 0 | 0 | 0 | 0 | 0 | 0 |
| 5/4/2006 | 99.96 | 12.68 | 12 | 0 | 13 | 0 | 0 | 198 |
| 5/5/2006 | 96.84 | 12.66 | 64 | 0 | 34 | 0 | 0 | 418 |
| 5/6/2006 | 95.43 | 12.28 | 14 | 0 | 10 | 0 | 0 | 293 |
| 5/7/2006 | 95.14 | 12.21 | 21 | 0 | 7 | 0 | 0 | 79 |

| Date | Discharge (cms) | Temp (C°) | Chinook | Coho | Steelhead | Cutthroat | Lamprey | Sucker |
|---|---|---|---|---|---|---|---|---|
| 5/8/2006 | 94.30 | 11.18 | 2 | 0 | 0 | 0 | 0 | 0 |
| 5/9/2006 | 92.31 | 11.58 | 0 | 0 | 0 | 0 | 0 | 0 |
| 5/10/2006 | 88.91 | 11.07 | 2 | 0 | 7 | 0 | 0 | 13 |
| 5/11/2006 | 87.22 | 13.18 | 57 | 0 | 15 | 0 | 0 | 800 |
| 5/12/2006 | 88.07 | 13.67 | 64 | 0 | 21 | 0 | 0 | 757 |
| 5/13/2006 | 88.91 | 13.38 | 23 | 0 | 7 | 0 | 0 | 200 |
| 5/14/2006 | 88.07 | 13.51 | 4 | 0 | 3 | 0 | 0 | 235 |
| 5/15/2006 | 88.63 | 13.31 | 10 | 0 | 3 | 0 | 0 | 9 |
| 5/16/2006 | 96.28 | 14.44 | 9 | 0 | 11 | 0 | 0 | 428 |
| 5/17/2006 | 103.07 | 14.55 | 44 | 0 | 1 | 0 | 0 | 45 |
| 5/18/2006 | 104.21 | 16.57 | 65 | 0 | 5 | 0 | 0 | 169 |
| 5/19/2006 | 105.34 | 15.83 | 103 | 0 | 12 | 1 | 0 | 100 |
| 5/20/2006 | 110.15 | 14.66 | 18 | 0 | 6 | 0 | 0 | 5 |
| 5/21/2006 | 100.81 | 14.41 | 4 | 0 | 0 | 0 | 0 | 1 |
| 5/22/2006 | 94.30 | 14.33 | 2 | 0 | 2 | 0 | 0 | 10 |
| 5/23/2006 | 89.76 | 14.75 | 42 | 0 | 12 | 0 | 0 | 17 |
| 5/24/2006 | 91.18 | 14.19 | 50 | 0 | 10 | 0 | 0 | 2 |
| 5/25/2006 | 89.48 | 13.62 | 12 | 0 | 6 | 0 | 0 | 0 |
| 5/26/2006 | 88.63 | 12.98 | 2 | 0 | 4 | 0 | 0 | 0 |
| 5/27/2006 | 116.38 | 11.60 | 5 | 0 | 1 | 0 | 0 | 0 |
| 5/28/2006 | 143.57 | 10.77 | 6 | 0 | 0 | 0 | 0 | 0 |
| 5/29/2006 | 135.35 | 12.04 | 0 | 0 | 0 | 0 | 0 | 0 |
| 5/30/2006 | 107.04 | 13.65 | 1 | 0 | 0 | 0 | 0 | 0 |
| 5/31/2006 | 92.31 | 14.32 | 29 | 0 | 2 | 0 | 0 | 45 |
| 6/1/2006 | 85.80 | 14.06 | 61 | 0 | 22 | 0 | 0 | 11 |
| 6/2/2006 | 94.30 | 15.28 | 98 | 0 | 77 | 0 | 0 | 19 |
| 6/3/2006 | 141.58 | 14.49 | 117 | 0 | 63 | 0 | 0 | 119 |
| 6/4/2006 | 128.56 | 15.38 | 90 | 0 | 58 | 0 | 0 | 60 |
| 6/5/2006 | 120.91 | 15.20 | 54 | 0 | 19 | 0 | 0 | 6 |
| 6/6/2006 | 109.59 | 15.80 | 86 | 0 | 42 | 0 | 0 | 0 |
| 6/7/2006 | 100.52 | 15.13 | 110 | 0 | 51 | 0 | 0 | 3 |
| 6/8/2006 | 87.78 | 15.81 | 113 | 0 | 94 | 0 | 0 | 3 |
| 6/9/2006 | 80.70 | 15.87 | 47 | 0 | 44 | 1 | 0 | 3 |
| 6/10/2006 | 77.30 | 15.82 | 94 | 0 | 62 | 0 | 0 | 12 |
| 6/11/2006 | 73.91 | 16.79 | 51 | 0 | 56 | 0 | 0 | 5 |
| 6/12/2006 | 72.49 | 17.72 | 29 | 0 | 34 | 3 | 1 | 0 |
| 6/13/2006 | 78.72 | 16.73 | 22 | 0 | 56 | 2 | 12 | 2193 |
| 6/14/2006 | 88.91 | 15.10 | 49 | 0 | 87 | 0 | 0 | 36 |
| 6/15/2006 | 90.61 | 14.98 | 24 | 0 | 67 | 1 | 0 | 14 |
| 6/16/2006 | 85.80 | 15.33 | 18 | 0 | 72 | 0 | 0 | 99 |
| 6/17/2006 | 79.00 | 15.82 | 17 | 0 | 31 | 0 | 0 | 85 |
| 6/18/2006 | 71.64 | 16.71 | 35 | 0 | 107 | 0 | 0 | 147 |
| 6/19/2006 | 65.70 | 17.01 | 10 | 0 | 11 | 0 | 0 | 24 |
| 6/20/2006 | 63.15 | 17.52 | 10 | 0 | 15 | 0 | 22 | 155 |
| 6/21/2006 | 60.88 | 17.84 | 38 | 0 | 96 | 0 | 0 | 59 |

| Date | Discharge (cms) | Temp (C°) | Chinook | Coho | Steelhead | Cutthroat | Lamprey | Sucker |
|---|---|---|---|---|---|---|---|---|
| 6/22/2006 | 57.48 | 17.89 | 41 | 0 | 79 | 1 | 0 | 53 |
| 6/23/2006 | 55.78 | 18.52 | 32 | 0 | 66 | 1 | 0 | 103 |
| 6/24/2006 | 53.52 | 19.33 | 29 | 0 | 52 | 1 | 0 | 105 |
| 6/25/2006 | 49.27 | 20.84 | 41 | 0 | 76 | 3 | 1 | 367 |
| 6/26/2006 | 47.86 | 22.59 | 35 | 0 | 107 | 8 | 2 | 818 |
| 6/27/2006 | 47.86 | 23.64 | 27 | 0 | 95 | 10 | 3 | 276 |
| 6/28/2006 | 48.70 | 23.06 | 16 | 0 | 66 | 4 | 1 | 161 |
| 6/29/2006 | 46.72 | 21.63 | 14 | 0 | 33 | 2 | 1 | 44 |
| 6/30/2006 | 45.59 | 22.32 | 9 | 0 | 20 | 1 | 0 | 129 |
| 7/1/2006 | 45.02 | 22.53 | 2 | 0 | 15 | 2 | 2 | 96 |
| 7/2/2006 | 43.61 | 22.56 | 3 | 0 | 11 | 1 | 0 | 20 |
| 7/3/2006 | 42.76 | 22.58 | 3 | 0 | 12 | 0 | 2 | 18 |
| 7/4/2006 | 42.48 | 22.47 | 1 | 0 | 8 | 0 | 0 | 12 |
| 7/5/2006 | 41.34 | 21.27 | 1 | 0 | 4 | 0 | 8 | 8 |
| 7/6/2006 | 40.78 | 19.23 | 1 | 0 | 2 | 0 | 0 | 0 |
| 7/7/2006 | 40.21 | 19.01 | 3 | 0 | 5 | 0 | 0 | 2 |
| 7/8/2006 | 39.08 | 20.09 | 11 | 0 | 30 | 0 | 1 | 27 |
| 7/9/2006 | 37.66 | 21.33 | 9 | 0 | 101 | 0 | 4 | 21 |
| 7/10/2006 | 35.68 | 21.85 | 9 | 0 | 109 | 0 | 3 | 0 |
| 7/11/2006 | 37.10 | 20.13 | 3 | 0 | 14 | 0 | 1 | 2 |
| 7/12/2006 | 36.25 | 19.72 | 12 | 0 | 181 | 2 | 0 | 2 |
| 7/13/2006 | 34.55 | 19.98 | 7 | 0 | 121 | 0 | 1 | 3 |
| 7/14/2006 | 34.26 | 21.32 | 5 | 0 | 108 | 0 | 0 | 0 |
| 7/15/2006 | 33.98 | 21.37 | 6 | 0 | 98 | 1 | 1 | 0 |
| 7/16/2006 | 33.41 | 21.63 | 2 | 0 | 45 | 1 | 1 | 0 |
| 7/17/2006 | 32.56 | 21.75 | 7 | 0 | 77 | 0 | 2 | 0 |
| 7/18/2006 | 32.28 | 22.17 | 7 | 0 | 65 | 0 | 3 | 0 |
| 7/19/2006 | 31.15 | 21.80 | 5 | 0 | 37 | 0 | 4 | 1 |
| 7/20/2006 | 32.00 | 21.46 | 4 | 0 | 32 | 0 | 4 | 0 |
| 7/21/2006 | 31.71 | 22.22 | 9 | 0 | 30 | 1 | 7 | 0 |
| 7/22/2006 | 31.15 | 22.69 | 0 | 0 | 22 | 1 | 5 | 4 |
| 7/23/2006 | 30.87 | 24.12 | 3 | 0 | 15 | 0 | 2 | 1 |
| 7/24/2006 | 30.30 | 25.27 | 0 | 0 | 3 | 0 | 3 | 0 |
| 7/25/2006 | 29.17 | 25.94 | 0 | 0 | 11 | 0 | 5 | 0 |
| 7/26/2006 | 29.73 | 25.07 | 0 | 0 | 2 | 0 | 2 | 0 |
| 7/27/2006 | 32.00 | 25.12 | 0 | 0 | 1 | 0 | 5 | 0 |
| 7/28/2006 | 31.71 | 24.53 | 0 | 0 | 0 | 0 | 3 | 0 |
| 7/29/2006 | 31.43 | 23.19 | 0 | 0 | 1 | 0 | 0 | 0 |
| 7/30/2006 | 32.28 | 22.09 | 0 | 0 | 0 | 0 | 1 | 0 |
| 7/31/2006 | 32.00 | 21.12 | 0 | 0 | 0 | 0 | 0 | 0 |
| 8/1/2006 | 31.43 | 21.04 | 0 | 0 | 0 | 0 | 0 | 0 |
| 8/2/2006 | 29.45 | 20.81 | 2 | 0 | 0 | 0 | 0 | 0 |
| 8/3/2006 | 29.45 | 20.37 | 3 | 0 | 0 | 0 | 1 | 0 |
| 8/4/2006 | 28.32 | 20.23 | 12 | 0 | 6 | 0 | 0 | 0 |
| 8/5/2006 | 28.18 | 19.88 | 10 | 0 | 15 | 0 | 0 | 0 |

| Date | Discharge (cms) | Temp (C°) | Chinook | Coho | Steelhead | Cutthroat | Lamprey | Sucker |
|---|---|---|---|---|---|---|---|---|
| 8/6/2006 | 28.88 | 20.40 | 11 | 0 | 37 | 0 | 2 | 0 |
| 8/7/2006 | 28.18 | 21.50 | 9 | 0 | 30 | 0 | 1 | 0 |
| 8/8/2006 | 27.33 | 21.06 | 17 | 0 | 29 | 0 | 0 | 0 |
| 8/9/2006 | 27.33 | 21.44 | 12 | 0 | 18 | 1 | 1 | 0 |
| 8/10/2006 | 26.90 | 21.80 | 15 | 0 | 28 | 0 | 4 | 0 |
| 8/11/2006 | 27.04 | 20.81 | 6 | 0 | 12 | 2 | 0 | 0 |
| 8/12/2006 | 26.19 | 20.30 | 7 | 0 | 14 | 0 | 0 | 0 |
| 8/13/2006 | 26.48 | 20.60 | 9 | 0 | 18 | 0 | 3 | 0 |
| 8/14/2006 | 27.04 | 20.85 | 12 | 0 | 27 | 0 | 4 | 0 |
| 8/15/2006 | 27.47 | 20.21 | 10 | 0 | 31 | 0 | 0 | 0 |
| 8/16/2006 | 27.18 | 19.13 | 0 | 0 | 7 | 0 | 0 | 0 |
| 8/17/2006 | 27.47 | 18.83 | 4 | 0 | 17 | 2 | 0 | 0 |
| 8/18/2006 | 27.47 | 19.68 | 4 | 0 | 14 | 1 | 0 | 0 |
| 8/19/2006 | 26.90 | 20.20 | 10 | 0 | 22 | 1 | 0 | 0 |
| 8/20/2006 | 26.62 | 20.51 | 17 | 0 | 31 | 2 | 1 | 0 |
| 8/21/2006 | 25.20 | 20.37 | 15 | 0 | 32 | 3 | 3 | 0 |
| 8/22/2006 | 24.64 | 19.72 | 7 | 0 | 50 | 4 | 0 | 0 |
| 8/23/2006 | 24.35 | 19.77 | 7 | 0 | 28 | 1 | 0 | 0 |
| 8/24/2006 | 23.79 | 20.09 | 7 | 0 | 28 | 0 | 0 | 0 |
| 8/25/2006 | 24.07 | 20.12 | 6 | 0 | 11 | 0 | 0 | 0 |
| 8/26/2006 | 24.21 | 20.25 | 4 | 0 | 19 | 0 | 0 | 0 |
| 8/27/2006 | 24.21 | 20.39 | 7 | 0 | 12 | 0 | 0 | 0 |
| 8/28/2006 | 24.07 | 20.83 | 9 | 0 | 22 | 0 | 0 | 0 |
| 8/29/2006 | 24.35 | 19.93 | 9 | 0 | 17 | 1 | 0 | 0 |
| 8/30/2006 | 23.79 | 19.00 | 1 | 0 | 7 | 1 | 0 | 0 |
| 8/31/2006 | 24.07 | 18.59 | 1 | 0 | 4 | 0 | 0 | 0 |
| 9/1/2006 | 23.93 | 18.73 | 2 | 0 | 8 | 0 | 0 | 0 |
| 9/2/2006 | 23.79 | 18.97 | 2 | 1 | 12 | 1 | 0 | 0 |
| 9/3/2006 | 23.79 | 19.07 | 6 | 0 | 14 | 0 | 0 | 0 |
| 9/4/2006 | 22.09 | 19.01 | 5 | 0 | 9 | 0 | 36 | 0 |
| 9/5/2006 | 27.04 | | 0 | 0 | 0 | 0 | 0 | 0 |
| 9/6/2006 | 26.33 | | 0 | 0 | 0 | 0 | 0 | 0 |
| 9/7/2006 | 23.79 | | 0 | 0 | 0 | 0 | 0 | 0 |
| 9/8/2006 | 23.36 | | 0 | 0 | 0 | 0 | 0 | 0 |
| 9/9/2006 | 23.08 | | 0 | 0 | 0 | 0 | 0 | 0 |
| 9/10/2006 | 22.09 | | 0 | 0 | 0 | 0 | 0 | 0 |
| 9/11/2006 | 22.51 | | 0 | 0 | 0 | 0 | 0 | 0 |
| 9/12/2006 | 22.09 | | 0 | 0 | 0 | 0 | 0 | 0 |
| 9/13/2006 | 22.23 | | 0 | 0 | 0 | 0 | 0 | 0 |
| 9/14/2006 | 22.23 | | 0 | 0 | 0 | 0 | 0 | 0 |
| 9/15/2006 | 22.09 | | 0 | 0 | 0 | 0 | 0 | 0 |
| 9/16/2006 | 20.10 | | 0 | 0 | 0 | 0 | 0 | 0 |
| 9/17/2006 | 21.52 | 17.27 | 5 | 12 | 9 | 0 | 0 | 0 |
| 9/18/2006 | 23.64 | 15.44 | 4 | 3 | 10 | 0 | 0 | 0 |
| 9/19/2006 | 23.93 | 14.58 | 0 | 6 | 3 | 0 | 0 | 0 |

| Date | Discharge (cms) | Temp (C°) | Chinook | Coho | Steelhead | Cutthroat | Lamprey | Sucker |
|---|---|---|---|---|---|---|---|---|
| 9/20/2006 | 25.77 | 14.44 | 3 | 4 | 11 | 1 | 0 | 0 |
| 9/21/2006 | 26.76 | 15.21 | 0 | 20 | 11 | 0 | 0 | 0 |
| 9/22/2006 | 27.47 | 15.20 | 2 | 35 | 9 | 0 | 0 | 0 |
| 9/23/2006 | 25.91 | 15.87 | 4 | 18 | 3 | 1 | 0 | 0 |
| 9/24/2006 | 24.21 | 16.02 | 3 | 26 | 11 | 0 | 0 | 0 |
| 9/25/2006 | 24.07 | 16.15 | 4 | 38 | 10 | 0 | 0 | 0 |
| 9/26/2006 | 23.64 | 16.11 | 6 | 32 | 12 | 1 | 0 | 0 |
| 9/27/2006 | 23.36 | 16.02 | 2 | 14 | 6 | 0 | 0 | 0 |
| 9/28/2006 | 23.79 | 15.90 | 0 | 22 | 3 | 0 | 0 | 0 |
| 9/29/2006 | 23.50 | 15.86 | 2 | 37 | 14 | 0 | 0 | 0 |
| 9/30/2006 | 23.50 | 15.79 | 6 | 47 | 9 | 0 | 0 | 0 |
| 10/1/2006 | 23.93 | 15.80 | 2 | 50 | 13 | 0 | 0 | 0 |
| 10/2/2006 | 23.79 | 15.56 | 0 | 21 | 9 | 0 | 0 | 0 |
| 10/3/2006 | 24.07 | 15.00 | 2 | 22 | 6 | 1 | 0 | 0 |
| 10/4/2006 | 23.79 | 14.02 | 1 | 7 | 1 | 0 | 0 | 0 |
| 10/5/2006 | 23.79 | 14.50 | 1 | 9 | 4 | 0 | 0 | 0 |
| 10/6/2006 | 23.50 | 14.50 | 0 | 9 | 7 | 0 | 0 | 0 |
| 10/7/2006 | 23.50 | 14.04 | 1 | 7 | 6 | 0 | 0 | 0 |
| 10/8/2006 | 23.22 | 13.79 | 1 | 5 | 3 | 0 | 0 | 0 |
| 10/9/2006 | 23.36 | 14.40 | 2 | 7 | 8 | 0 | 0 | 0 |
| 10/10/2006 | 23.08 | 13.09 | 1 | 41 | 11 | 0 | 0 | 0 |
| 10/11/2006 | 24.07 | 12.40 | 1 | 17 | 3 | 0 | 0 | 0 |
| 10/12/2006 | 23.50 | 12.14 | 3 | 8 | 1 | 0 | 0 | 0 |
| 10/13/2006 | 23.22 | 12.03 | 4 | 4 | 6 | 0 | 0 | 0 |
| 10/14/2006 | 23.08 | 11.87 | 2 | 6 | 5 | 0 | 0 | 0 |
| 10/15/2006 | 22.37 | 11.38 | 2 | 13 | 8 | 0 | 0 | 0 |
| 10/16/2006 | 26.19 | 10.75 | 1 | 2 | 3 | 0 | 0 | 0 |
| 10/17/2006 | 41.63 | 10.47 | 3 | 102 | 4 | 0 | 0 | 0 |
| 10/18/2006 | 32.00 | 11.00 | 5 | 456 | 50 | 0 | 0 | 0 |
| 10/19/2006 | 24.35 | 11.59 | 7 | 279 | 37 | 1 | 0 | 0 |
| 10/20/2006 | 22.37 | 12.27 | 1 | 75 | 16 | 0 | 0 | 0 |
| 10/21/2006 | 22.09 | 12.42 | 2 | 51 | 5 | 0 | 0 | 0 |
| 10/22/2006 | 21.95 | 12.18 | 1 | 18 | 2 | 0 | 0 | 0 |
| 10/23/2006 | 21.66 | 11.28 | 0 | 8 | 1 | 0 | 0 | 0 |
| 10/24/2006 | 21.38 | 10.17 | 0 | 6 | 1 | 0 | 0 | 0 |
| 10/25/2006 | 21.24 | 9.34 | 0 | 1 | 1 | 0 | 0 | 0 |
| 10/26/2006 | 21.24 | 9.40 | 0 | 3 | 4 | 0 | 0 | 0 |
| 10/27/2006 | 21.52 | 9.34 | 1 | 3 | 1 | 1 | 0 | 0 |
| 10/28/2006 | 21.52 | 9.17 | 0 | 0 | 2 | 0 | 0 | 0 |
| 10/29/2006 | 21.52 |  | 0 | 1 | 1 | 0 | 0 | 0 |
| 10/30/2006 | 21.80 | 8.07 | 0 | 1 | 1 | 0 | 0 | 0 |
| 10/31/2006 | 21.52 | 7.70 | 0 | 0 | 1 | 0 | 0 | 0 |
| 11/1/2006 | 21.15 | 7.13 | 0 | 0 | 0 | 0 | 0 | 0 |
| 11/2/2006 | 21.78 | 7.09 | 0 | 0 | 0 | 0 | 0 | 0 |
| 11/3/2006 | 22.26 | 7.78 | 1 | 2 | 1 | 0 | 0 | 0 |

| Date | Discharge (cms) | Temp (C°) | Chinook | Coho | Steelhead | Cutthroat | Lamprey | Sucker |
|---|---|---|---|---|---|---|---|---|
| 11/4/2006 | 27.95 | 8.87 | 0 | 50 | 15 | 0 | 0 | 0 |
| 11/5/2006 | 42.19 | 9.75 | 6 | 339 | 38 | 0 | 0 | 0 |
| 11/6/2006 | 76.46 | 10.32 | 2 | 460 | 36 | 0 | 0 | 1 |
| 11/7/2006 | 47.01 | 11.73 | 9 | 161 | 14 | 0 | 1 | 41 |
| 11/8/2006 | 121.76 | 11.81 | 6 | 192 | 20 | 0 | 1 | 0 |
| 11/9/2006 | 79.57 | 10.74 | 5 | 248 | 23 | 0 | 0 | 1 |
| 11/10/2006 | 63.43 | 9.24 | 0 | 86 | 6 | 0 | 0 | 0 |
| 11/11/2006 | 72.49 | 8.67 | 0 | 48 | 7 | 0 | 0 | 0 |
| 11/12/2006 | 70.51 | 7.87 | 0 | 30 | 6 | 0 | 0 | 0 |
| 11/13/2006 | 113.55 | 7.95 | 0 | 6 | 4 | 0 | 0 | 0 |
| 11/14/2006 | 220.31 | 8.88 | 0 | 11 | 7 | 0 | 0 | 0 |
| 11/15/2006 | 124.88 | 8.36 | 0 | 37 | 14 | 0 | 1 | 0 |
| 11/16/2006 | 137.34 | 8.45 | 0 | 21 | 5 | 1 | 0 | 0 |
| 11/17/2006 | 136.77 | 8.76 | 0 | 11 | 4 | 0 | 0 | 0 |
| 11/18/2006 | 87.22 | 8.39 | 0 | 8 | 20 | 0 | 0 | 0 |
| 11/19/2006 | 65.70 | 8.15 | 0 | 13 | 10 | 0 | 0 | 0 |
| 11/20/2006 | 129.69 | 8.49 | 0 | 19 | 5 | 0 | 0 | 0 |
| 11/21/2006 | 159.71 |  | 0 | 11 | 6 | 0 | 0 | 0 |
| 11/22/2006 | 208.70 | 8.78 | 0 | 1 | 6 | 0 | 0 | 0 |
| 11/23/2006 | 283.17 | 8.54 | 0 | 8 | 7 | 0 | 0 | 0 |
| 11/24/2006 | 201.90 | 8.45 | 0 | 4 | 8 | 0 | 0 | 0 |
| 11/25/2006 | 204.73 | 8.53 | 0 | 2 | 11 | 0 | 0 | 0 |
| 11/26/2006 | 185.19 | 8.22 | 0 | 1 | 9 | 1 | 0 | 0 |
| 11/27/2006 | 265.90 | 7.83 | 0 | 1 | 1 | 0 | 0 | 0 |
| 11/28/2006 | 176.98 | 6.92 | 0 | 0 | 2 | 0 | 0 | 0 |
| 11/29/2006 | 121.20 | 5.89 | 0 | 2 | 2 | 0 | 0 | 0 |
| 11/30/2006 | 93.73 | 5.27 | 0 | 1 | 6 | 0 | 0 | 0 |
| 12/1/2006 | 120.91 | 5.39 | 0 | 3 | 4 | 0 | 0 | 0 |
| 12/2/2006 | 112.70 | 5.56 | 0 | 1 | 4 | 0 | 0 | 0 |
| 12/3/2006 | 94.01 | 5.00 | 0 | 1 | 1 | 0 | 0 | 0 |
| 12/4/2006 | 84.38 | 4.42 | 0 | 0 | 2 | 0 | 0 | 0 |
| 12/5/2006 | 82.40 | 4.75 | 0 | 0 | 9 | 0 | 0 | 0 |
| 12/6/2006 | 88.07 | 4.89 | 0 | 0 | 4 | 0 | 0 | 0 |
| 12/7/2006 | 94.58 | 4.97 | 0 | 0 | 0 | 0 | 0 | 0 |
| 12/8/2006 | 94.30 | 5.13 | 0 | 0 | 5 | 0 | 0 | 0 |
| 12/9/2006 | 95.14 | 6.32 | 0 | 1 | 52 | 1 | 0 | 0 |
| 12/10/2006 | 96.56 | 6.83 | 0 | 4 | 33 | 0 | 0 | 0 |
| 12/11/2006 | 92.31 | 7.05 | 0 | 2 | 24 | 0 | 0 | 0 |
| 12/12/2006 | 149.51 | 7.22 | 0 | 3 | 22 | 0 | 0 | 0 |
| 12/13/2006 | 421.92 | 7.90 | 0 | 0 | 1 | 0 | 0 | 0 |
| 12/14/2006 | 747.56 | 8.43 | 0 | 0 | 0 | 0 | 0 | 0 |
| 12/15/2006 | 628.63 | 8.03 | 0 | 0 | 0 | 0 | 0 | 0 |
| 12/16/2006 | 362.46 | 6.09 | 0 | 0 | 3 | 0 | 0 | 0 |
| 12/17/2006 | 234.75 | 5.24 | 0 | 0 | 3 | 0 | 0 | 0 |
| 12/18/2006 | 170.75 | 4.41 | 0 | 0 | 5 | 0 | 0 | 0 |

| Date | Discharge (cms) | Temp (C°) | Chinook | Coho | Steelhead | Cutthroat | Lamprey | Sucker |
|---|---|---|---|---|---|---|---|---|
| 12/19/2006 | 140.17 | 3.64 | 0 | 0 | 5 | 0 | 0 | 0 |
| 12/20/2006 | 120.06 | 3.57 | 0 | 0 | 7 | 0 | 0 | 0 |
| 12/21/2006 | 133.09 | 4.91 | 0 | 0 | 72 | 0 | 0 | 0 |
| 12/22/2006 | 162.54 | 5.74 | 0 | 0 | 59 | 0 | 0 | 0 |
| 12/23/2006 | 143.28 | 6.15 | 0 | 2 | 93 | 1 | 0 | 0 |
| 12/24/2006 | 192.27 | 6.65 | 0 | 1 | 56 | 0 | 0 | 0 |
| 12/25/2006 | 481.39 | 7.32 | 0 | 0 | 12 | 0 | 0 | 0 |
| 12/26/2006 | 866.50 | 8.12 | 0 | 0 | 0 | 0 | 0 | 0 |
| 12/27/2006 | 611.64 | 7.88 | 0 | 0 | 1 | 0 | 0 | 0 |
| 12/28/2006 | 359.62 | 6.49 | 0 | 0 | 3 | 0 | 0 | 0 |
| 12/29/2006 | 247.49 | 5.55 | 0 | 0 | 5 | 0 | 0 | 0 |
| 12/30/2006 | 189.16 | 5.38 | 0 | 0 | 21 | 0 | 0 | 0 |
| 12/31/2006 | 157.44 | 5.66 | 0 | 0 | 52 | 0 | 0 | 0 |
| 1/1/2007 | 134.22 | 5.47 | 0 | 0 | 17 | 0 | 0 | 0 |
| 1/2/2007 | 118.65 | 5.94 | 0 | 0 | 90 | 0 | 0 | 0 |
| 1/3/2007 | 334.14 | | 0 | 0 | 62 | 0 | 0 | 0 |
| 1/4/2007 | 583.33 | | 0 | 0 | 0 | 0 | 0 | 0 |
| 1/5/2007 | 331.31 | 6.05 | 0 | 0 | 4 | 0 | 0 | 0 |
| 1/6/2007 | 305.82 | 5.91 | 0 | 0 | 6 | 0 | 0 | 0 |
| 1/7/2007 | 266.18 | 6.35 | 0 | 0 | 26 | 0 | 0 | 0 |
| 1/8/2007 | 252.87 | 6.88 | 0 | 0 | 27 | 0 | 0 | 0 |
| 1/9/2007 | 252.02 | 6.56 | 0 | 0 | 18 | 0 | 0 | 0 |
| 1/10/2007 | 246.36 | 6.42 | 0 | 0 | 41 | 0 | 0 | 0 |
| 1/11/2007 | 215.49 | 5.17 | 0 | 0 | 5 | 0 | 0 | 0 |
| 1/12/2007 | 173.87 | 3.82 | 0 | 0 | 0 | 0 | 0 | 0 |
| 1/13/2007 | 144.98 | 2.60 | 0 | 0 | 0 | 0 | 0 | 0 |
| 1/14/2007 | 126.01 | 1.97 | 0 | 0 | 0 | 0 | 0 | 1 |
| 1/15/2007 | 112.98 | 2.33 | 0 | 0 | 0 | 0 | 0 | 0 |
| 1/16/2007 | 104.49 | 1.67 | 0 | 0 | 0 | 0 | 0 | 0 |
| 1/17/2007 | 99.96 | 2.44 | 0 | 0 | 3 | 0 | 0 | 0 |
| 1/18/2007 | 92.03 | 3.47 | 0 | 0 | 11 | 0 | 0 | 0 |
| 1/19/2007 | 87.22 | 3.62 | 0 | 0 | 7 | 0 | 0 | 0 |
| 1/20/2007 | 88.07 | 4.26 | 0 | 0 | 9 | 0 | 0 | 0 |
| 1/21/2007 | 87.78 | 4.31 | 0 | 0 | 3 | 0 | 0 | 0 |
| 1/22/2007 | 83.53 | 3.91 | 0 | 0 | 2 | 0 | 0 | 0 |
| 1/23/2007 | 79.29 | 3.67 | 0 | 0 | 1 | 0 | 0 | 0 |
| 1/24/2007 | 77.59 | 3.35 | 0 | 0 | 1 | 0 | 0 | 0 |
| 1/25/2007 | 80.14 | 3.63 | 0 | 0 | 0 | 0 | 0 | 0 |
| 1/26/2007 | 80.99 | 3.81 | 0 | 0 | 1 | 0 | 0 | 0 |
| 1/27/2007 | 78.72 | 3.63 | 0 | 0 | 3 | 0 | 0 | 0 |
| 1/28/2007 | 77.02 | 3.61 | 0 | 0 | 1 | 0 | 0 | 0 |
| 1/29/2007 | 73.34 | 3.06 | 0 | 0 | 0 | 0 | 0 | 0 |
| 1/30/2007 | 69.66 | 3.10 | 0 | 0 | 0 | 0 | 0 | 0 |
| 1/31/2007 | 67.68 | 3.01 | 0 | 0 | 0 | 0 | 0 | 0 |
| 2/1/2007 | 65.13 | 2.69 | 0 | 0 | 0 | 0 | 0 | 0 |

| Date | Discharge (cms) | Temp (C°) | Chinook | Coho | Steelhead | Cutthroat | Lamprey | Sucker |
|---|---|---|---|---|---|---|---|---|
| 2/2/2007 | 61.16 | 2.61 | 0 | 0 | 0 | 0 | 0 | 0 |
| 2/3/2007 | 58.05 | 3.12 | 0 | 0 | 1 | 0 | 0 | 0 |
| 2/4/2007 | 55.50 | 4.13 | 0 | 0 | 2 | 0 | 0 | 0 |
| 2/5/2007 | 56.07 | 5.37 | 0 | 0 | 33 | 0 | 0 | 0 |
| 2/6/2007 | 63.43 | 5.95 | 0 | 0 | 156 | 0 | 0 | 0 |
| 2/7/2007 | 67.39 | 6.06 | 0 | 0 | 148 | 0 | 0 | 0 |
| 2/8/2007 | 80.42 | 5.95 | 0 | 0 | 351 | 0 | 0 | 0 |
| 2/9/2007 | 89.48 | 6.33 | 0 | 0 | 290 | 0 | 0 | 0 |
| 2/10/2007 | 94.58 | 6.30 | 0 | 0 | 135 | 0 | 0 | 0 |
| 2/11/2007 | 119.50 | 6.94 | 0 | 0 | 347 | 0 | 0 | 0 |
| 2/12/2007 | 147.81 | 6.90 | 0 | 0 | 13 | 0 | 0 | 0 |
| 2/13/2007 | 141.87 | 7.03 | 0 | 0 | 0 | 0 | 0 | 0 |
| 2/14/2007 | 130.26 | 6.68 | 0 | 0 | 16 | 0 | 0 | 0 |
| 2/15/2007 | 253.44 | 6.97 | 0 | 0 | 66 | 0 | 0 | 0 |
| 2/16/2007 | 484.22 | 7.99 | 0 | 0 | 90 | 0 | 0 | 0 |
| 2/17/2007 | 399.27 | 8.17 | 0 | 0 | 74 | 0 | 0 | 0 |
| 2/18/2007 | 265.90 | 7.96 | 0 | 0 | 99 | 0 | 0 | 0 |
| 2/19/2007 | 206.71 | 7.01 | 0 | 0 | 88 | 0 | 0 | 0 |
| 2/20/2007 | 184.06 | 6.81 | 0 | 0 | 92 | 0 | 0 | 0 |
| 2/21/2007 | 202.18 | 6.34 | 0 | 0 | 24 | 0 | 0 | 0 |
| 2/22/2007 | 210.68 | 5.91 | 0 | 0 | 2 | 0 | 0 | 0 |
| 2/23/2007 | 191.71 | 5.81 | 0 | 0 | 5 | 0 | 0 | 0 |
| 2/24/2007 | 178.68 | 6.09 | 0 | 0 | 21 | 0 | 0 | 0 |
| 2/25/2007 | 248.06 | 6.20 | 0 | 0 | 17 | 0 | 0 | 0 |
| 2/26/2007 | 246.07 | 6.02 | 0 | 0 | 9 | 0 | 0 | 0 |
| 2/27/2007 | 194.54 | 5.81 | 0 | 0 | 12 | 0 | 0 | 0 |
| 2/28/2007 | 177.83 | 5.56 | 0 | 0 | 9 | 0 | 0 | 0 |
| 3/1/2007 | 165.94 | 5.43 | 0 | 0 | 6 | 0 | 0 | 0 |
| 3/2/2007 | 178.11 | 5.86 | 0 | 0 | 31 | 0 | 0 | 0 |
| 3/3/2007 | 254.29 | 7.15 | 0 | 0 | 110 | 0 | 0 | 0 |
| 3/4/2007 | 282.04 | 7.50 | 0 | 0 | 228 | 0 | 0 | 0 |
| 3/5/2007 | 300.16 | 7.41 | 0 | 0 | 296 | 0 | 0 | 0 |
| 3/6/2007 | 283.17 | 7.38 | 0 | 0 | 164 | 0 | 0 | 0 |
| 3/7/2007 | 264.76 | 7.35 | 0 | 0 | 264 | 0 | 0 | 0 |
| 3/8/2007 | 260.80 | 7.54 | 0 | 0 | 227 | 1 | 0 | 0 |
| 3/9/2007 | 223.14 | 7.47 | 0 | 0 | 289 | 0 | 0 | 0 |
| 3/10/2007 | 217.47 | 7.40 | 0 | 0 | 151 | 1 | 0 | 0 |
| 3/11/2007 | 208.41 | 8.53 | 0 | 0 | 454 | 0 | 0 | 0 |
| 3/12/2007 | 246.64 | 8.37 | 0 | 0 | 253 | 0 | 0 | 0 |
| 3/13/2007 | 241.83 | 8.48 | 0 | 0 | 201 | 0 | 0 | 0 |
| 3/14/2007 | 211.81 | 8.66 | 0 | 0 | 152 | 0 | 0 | 0 |
| 3/15/2007 | 174.43 | 8.24 | 0 | 0 | 44 | 0 | 0 | 0 |
| 3/16/2007 | 150.93 | 8.53 | 0 | 0 | 46 | 1 | 0 | 0 |
| 3/17/2007 | 145.55 | 9.17 | 0 | 0 | 190 | 0 | 0 | 1 |
| 3/18/2007 | 145.55 | 9.69 | 0 | 0 | 212 | 0 | 0 | 65 |

| Date | Discharge (cms) | Temp (C°) | Chinook | Coho | Steelhead | Cutthroat | Lamprey | Sucker |
|---|---|---|---|---|---|---|---|---|
| 3/19/2007 | 145.27 | 9.07 | 0 | 0 | 162 | 0 | 0 | 45 |
| 3/20/2007 | 161.41 | 8.82 | 0 | 0 | 103 | 0 | 0 | 0 |
| 3/21/2007 | 160.27 | 7.98 | 0 | 0 | 6 | 0 | 0 | 0 |
| 3/22/2007 | 138.75 | 8.02 | 0 | 0 | 8 | 0 | 0 | 0 |
| 3/23/2007 | 125.44 | 9.05 | 0 | 0 | 71 | 0 | 0 | 0 |
| 3/24/2007 | 116.10 | 9.36 | 0 | 0 | 131 | 0 | 0 | 0 |
| 3/25/2007 | 126.29 | 9.79 | 1 | 0 | 248 | 0 | 0 | 64 |
| 3/26/2007 | 153.19 | 8.78 | 0 | 0 | 72 | 0 | 0 | 34 |
| 3/27/2007 | 175.56 | 7.18 | 0 | 0 | 2 | 0 | 0 | 0 |
| 3/28/2007 | 167.07 | 7.19 | 0 | 0 | 2 | 0 | 0 | 0 |
| 3/29/2007 | 147.25 | 8.01 | 0 | 0 | 14 | 0 | 0 | 0 |
| 3/30/2007 | 134.51 | 8.55 | 0 | 0 | 52 | 0 | 0 | 0 |
| 3/31/2007 | 125.44 | 8.93 | 3 | 0 | 124 | 0 | 0 | 0 |
| 4/1/2007 | 117.23 | 9.04 | 0 | 0 | 91 | 0 | 0 | 0 |
| 4/2/2007 | 109.30 | 9.33 | 0 | 0 | 35 | 0 | 0 | 0 |
| 4/3/2007 | 100.81 | 8.79 | 0 | 0 | 28 | 0 | 0 | 0 |
| 4/4/2007 | 92.88 | 9.67 | 0 | 0 | 80 | 0 | 0 | 0 |
| 4/5/2007 | 86.93 | 10.68 | 2 | 0 | 243 | 0 | 1 | 268 |
| 4/6/2007 | 84.38 | 11.58 | 3 | 0 | 163 | 0 | 0 | 819 |
| 4/7/2007 | 87.50 | 12.08 | 3 | 0 | 83 | 0 | 0 | 829 |
| 4/8/2007 | 95.14 | 11.38 | 3 | 0 | 49 | 0 | 0 | 132 |
| 4/9/2007 | 98.83 | 10.36 | 2 | 0 | 35 | 0 | 0 | 2 |
| 4/10/2007 | 111.00 | 9.97 | 0 | 0 | 7 | 0 | 0 | 0 |
| 4/11/2007 | 114.68 | 9.21 | 0 | 0 | 9 | 0 | 0 | 0 |
| 4/12/2007 | 122.90 | 8.49 | 0 | 0 | 6 | 0 | 0 | 0 |
| 4/13/2007 | 127.71 | 8.70 | 1 | 0 | 5 | 0 | 0 | 0 |
| 4/14/2007 | 127.71 | 8.97 | 0 | 0 | 17 | 0 | 0 | 0 |
| 4/15/2007 | 136.77 | 9.26 | 1 | 0 | 10 | 0 | 1 | 0 |
| 4/16/2007 | 131.39 | 8.56 | 0 | 0 | 6 | 0 | 0 | 3 |
| 4/17/2007 | 123.18 | 8.32 | 0 | 0 | 5 | 0 | 0 | 0 |
| 4/18/2007 | 132.24 | 8.58 | 1 | 0 | 6 | 0 | 0 | 0 |
| 4/19/2007 | 141.30 | 8.00 | 1 | 0 | 1 | 0 | 0 | 0 |
| 4/20/2007 | 133.37 | 8.79 | 0 | 0 | 5 | 0 | 0 | 1 |
| 4/21/2007 | 122.61 | 8.50 | 1 | 0 | 16 | 0 | 0 | 2 |
| 4/22/2007 | 156.03 | 9.06 | 3 | 0 | 26 | 0 | 0 | 18 |
| 4/23/2007 | 166.22 | 10.09 | 6 | 0 | 24 | 0 | 0 | 93 |
| 4/24/2007 | 147.81 | 11.02 | 8 | 0 | 28 | 0 | 0 | 381 |
| 4/25/2007 | 129.97 | 12.03 | 17 | 0 | 29 | 0 | 1 | 493 |
| 4/26/2007 | 117.23 | 11.76 | 4 | 0 | 21 | 0 | 0 | 210 |
| 4/27/2007 | 106.47 | 12.31 | 7 | 0 | 5 | 0 | 0 | 355 |
| 4/28/2007 | 99.39 | 12.41 | 22 | 0 | 14 | 1 | 0 | 647 |
| 4/29/2007 | 95.14 | 13.39 | 28 | 0 | 13 | 0 | 1 | 717 |
| 4/30/2007 | 92.88 | 13.64 | 17 | 0 | 8 | 0 | 1 | 47 |
| 5/1/2007 | 90.61 | 12.43 | 22 | 0 | 5 | 0 | 0 | 11 |
| 5/2/2007 | 92.03 | 11.17 | 2 | 0 | 0 | 0 | 0 | 2 |

| Date | Discharge (cms) | Temp (C°) | Chinook | Coho | Steelhead | Cutthroat | Lamprey | Sucker |
|---|---|---|---|---|---|---|---|---|
| 5/3/2007 | 97.41 | 10.02 | 3 | 0 | 4 | 0 | 0 | 0 |
| 5/4/2007 | 97.69 | 9.89 | 0 | 0 | 4 | 0 | 0 | 0 |
| 5/5/2007 | 94.58 | 10.10 | 2 | 0 | 2 | 0 | 0 | 0 |
| 5/6/2007 | 87.50 | 11.92 | 14 | 0 | 3 | 0 | 0 | 1 |
| 5/7/2007 | 83.25 | 13.43 | 50 | 0 | 12 | 0 | 1 | 297 |
| 5/8/2007 | 81.84 | 14.79 | 48 | 0 | 5 | 1 | 1 | 476 |
| 5/9/2007 | 82.12 | 14.86 | 17 | 0 | 0 | 0 | 1 | 26 |
| 5/10/2007 | 82.97 | 13.99 | 7 | 0 | 2 | 1 | 0 | 236 |
| 5/11/2007 | 82.69 | 14.19 | 75 | 0 | 12 | 0 | 1 | 138 |
| 5/12/2007 | 79.00 | 12.38 | 66 | 0 | 9 | 0 | 0 | 25 |
| 5/13/2007 | 76.17 | 13.25 | 13 | 0 | 3 | 0 | 0 | 1 |
| 5/14/2007 | 73.06 | 12.88 | 4 | 0 | 1 | 0 | 0 | 1 |
| 5/15/2007 | 71.08 | 14.09 | 10 | 0 | 8 | 0 | 0 | 176 |
| 5/16/2007 | 68.24 | 14.89 | 30 | 0 | 0 | 0 | 0 | 0 |
| 5/17/2007 | 65.41 | 15.45 | 9 | 0 | 9 | 0 | 0 | 222 |
| 5/18/2007 | 64.00 | 15.97 | 95 | 0 | 9 | 0 | 0 | 93 |
| 5/19/2007 | 62.58 | 15.69 | 72 | 0 | 8 | 0 | 1 | 87 |
| 5/20/2007 | 61.45 | 14.32 | 34 | 0 | 3 | 0 | 0 | 8 |
| 5/21/2007 | 64.56 | 13.49 | 4 | 0 | 0 | 0 | 0 | 0 |
| 5/22/2007 | 63.71 | 13.32 | 7 | 0 | 1 | 1 | 0 | 9 |
| 5/23/2007 | 62.01 | 14.15 | 27 | 0 | 0 | 0 | 0 | 0 |
| 5/24/2007 | 57.77 | 15.81 | 42 | 0 | 15 | 1 | 0 | 374 |
| 5/25/2007 | 55.78 | 16.65 | 216 | 0 | 19 | 0 | 2 | 66 |
| 5/26/2007 | 54.65 | 16.74 | 109 | 0 | 13 | 0 | 7 | 126 |
| 5/27/2007 | 54.37 | 16.66 | 109 | 0 | 16 | 0 | 2 | 159 |
| 5/28/2007 | 53.80 | 15.76 | 52 | 0 | 9 | 1 | 0 | 2 |
| 5/29/2007 | 54.09 | 16.52 | 45 | 0 | 6 | 1 | 0 | 7 |
| 5/30/2007 | 51.82 | 18.10 | 28 | 0 | 32 | 1 | 0 | 405 |
| 5/31/2007 | 51.54 | 19.09 | 76 | 0 | 9 | 0 | 2 | 87 |
| 6/1/2007 | 51.54 | 19.74 | 70 | 0 | 18 | 1 | 15 | 57 |
| 6/2/2007 | 51.54 | 19.96 | 57 | 0 | 8 | 0 | 15 | 29 |
| 6/3/2007 | 50.40 | 20.63 | 64 | 0 | 16 | 2 | 8 | 66 |
| 6/4/2007 | 48.99 | 20.55 | 60 | 0 | 15 | 3 | 24 | 483 |
| 6/5/2007 | 49.84 | 19.28 | 28 | 0 | 13 | 0 | 12 | 87 |
| 6/6/2007 | 59.18 | 17.46 | 23 | 0 | 12 | 0 | 4 | 40 |
| 6/7/2007 | 62.01 | 16.18 | 10 | 0 | 5 | 0 | 0 | 7 |
| 6/8/2007 | 56.92 | 16.07 | 14 | 0 | 4 | 0 | 0 | 23 |
| 6/9/2007 | 52.10 | 16.58 | 19 | 0 | 12 | 0 | 2 | 235 |
| 6/10/2007 | 52.67 | 16.20 | 10 | 0 | 12 | 0 | 0 | 147 |
| 6/11/2007 | 54.65 | 16.19 | 1 | 0 | 2 | 0 | 1 | 45 |
| 6/12/2007 | 50.97 | 17.34 | 0 | 0 | 0 | 0 | 0 | 0 |
| 6/13/2007 | 48.14 | 18.55 | 0 | 0 | 0 | 0 | 0 | 0 |
| 6/14/2007 | 46.16 | 18.54 | 6 | 0 | 3 | 0 | 4 | 838 |
| 6/15/2007 | 44.17 | 19.31 | 60 | 0 | 74 | 2 | 3 | 50 |
| 6/16/2007 | 41.63 | 19.16 | 52 | 0 | 46 | 0 | 3 | 29 |

| Date | Discharge (cms) | Temp (C°) | Chinook | Coho | Steelhead | Cutthroat | Lamprey | Sucker |
|---|---|---|---|---|---|---|---|---|
| 6/17/2007 | 40.21 | 17.54 | 37 | 0 | 28 | 0 | 1 | 7 |
| 6/18/2007 | 39.93 | 18.70 | 19 | 0 | 17 | 0 | 1 | 3 |
| 6/19/2007 | 39.36 | 18.90 | 4 | 0 | 2 | 1 | 0 | 27 |
| 6/20/2007 | 38.51 | 20.17 | 36 | 0 | 76 | 1 | 1 | 246 |
| 6/21/2007 | 37.38 | 20.12 | 32 | 0 | 36 | 1 | 1 | 23 |
| 6/22/2007 | 36.53 | 19.72 | 23 | 0 | 54 | 2 | 2 | 15 |
| 6/23/2007 | 35.68 | 18.92 | 5 | 0 | 26 | 1 | 2 | 1 |
| 6/24/2007 | 35.40 | 18.25 | 15 | 0 | 34 | 0 | 0 | 0 |
| 6/25/2007 | 35.68 | 17.85 | 6 | 0 | 7 | 0 | 0 | 0 |
| 6/26/2007 | 35.68 | 19.11 | 0 | 0 | 0 | 0 | 0 | 0 |
| 6/27/2007 | 35.40 | 19.34 | 4 | 0 | 0 | 3 | 1 | 9 |
| 6/28/2007 | 35.11 | 20.00 | 24 | 0 | 75 | 1 | 0 | 11 |
| 6/29/2007 | 35.40 | 19.19 | 22 | 0 | 59 | 1 | 2 | 5 |
| 6/30/2007 | 35.40 | 18.44 | 5 | 0 | 36 | 1 | 0 | 0 |
| 7/1/2007 | 35.11 | 19.71 | 12 | 0 | 88 | 1 | 2 | 2 |
| 7/2/2007 | 34.55 | 20.24 | 4 | 0 | 51 | 0 | 2 | 0 |
| 7/3/2007 | 33.41 | 20.91 | 13 | 0 | 27 | 0 | 6 | 0 |
| 7/4/2007 | 33.13 | 22.78 | 0 | 0 | 0 | 0 | 0 | 0 |
| 7/5/2007 | 32.56 | 23.28 | 20 | 0 | 104 | 0 | 0 | 14 |
| 7/6/2007 | 31.71 | 23.84 | 9 | 0 | 46 | 2 | 2 | 6 |
| 7/7/2007 | 31.43 | 23.71 | 5 | 0 | 31 | 2 | 3 | 4 |
| 7/8/2007 | 30.87 | 23.56 | 2 | 0 | 16 | 0 | 1 | 1 |
| 7/9/2007 | 30.58 | 23.77 | 1 | 0 | 20 | 1 | 0 | 8 |
| 7/10/2007 | 29.73 | 24.34 | 1 | 0 | 6 | 1 | 1 | 2 |
| 7/11/2007 | 29.17 | 25.10 | 0 | 0 | 3 | 1 | 1 | 7 |
| 7/12/2007 | 30.30 | 23.82 | 1 | 0 | 1 | 1 | 1 | 15 |
| 7/13/2007 | 30.58 | 22.47 | 0 | 0 | 3 | 0 | 1 | 3 |
| 7/14/2007 | 29.17 | 23.31 | 1 | 0 | 1 | 0 | 1 | 20 |
| 7/15/2007 | 29.17 | 23.64 | 0 | 0 | 0 | 0 | 0 | 0 |
| 7/16/2007 | 29.17 | 23.23 | 2 | 0 | 1 | 1 | 0 | 1 |
| 7/17/2007 | 29.45 | 22.01 | 1 | 0 | 1 | 0 | 0 | 0 |
| 7/18/2007 | 31.43 | 20.72 | 0 | 0 | 1 | 0 | 0 | 1 |
| 7/19/2007 | 36.25 | 20.28 | 0 | 0 | 0 | 0 | 0 | 0 |
| 7/20/2007 | 36.25 | 19.73 | 5 | 0 | 8 | 0 | 0 | 35 |
| 7/21/2007 | 31.43 | 19.43 | 8 | 0 | 14 | 0 | 0 | 3 |
| 7/22/2007 | 29.45 | 20.38 | 5 | 0 | 27 | 1 | 1 | 1 |
| 7/23/2007 | 28.88 | 21.99 | 15 | 0 | 40 | 1 | 0 | 0 |
| 7/24/2007 | 28.88 | 21.90 | 6 | 0 | 60 | 5 | 0 | 0 |
| 7/25/2007 | 28.60 | 21.94 | 4 | 0 | 48 | 1 | 0 | 0 |
| 7/26/2007 | 28.20 | 22.33 | 7 | 0 | 70 | 4 | 1 | 0 |
| 7/27/2007 | 28.18 | 22.02 | 1 | 0 | 38 | 1 | 0 | 2 |
| 7/28/2007 | 28.12 | 22.75 | 5 | 0 | 39 | 2 | 1 | 0 |
| 7/29/2007 | 27.75 | 22.45 | 3 | 0 | 22 | 2 | 0 | 0 |
| 7/30/2007 | 27.47 | 21.16 | 2 | 0 | 11 | 1 | 0 | 0 |
| 7/31/2007 | 27.16 | 22.02 | 2 | 0 | 5 | 0 | 0 | 0 |

| Date | Discharge (cms) | Temp (C°) | Chinook | Coho | Steelhead | Cutthroat | Lamprey | Sucker |
|---|---|---|---|---|---|---|---|---|
| 8/1/2007 | 26.96 | 22.47 | 1 | 0 | 11 | 0 | 0 | 0 |
| 8/2/2007 | 26.87 | 23.05 | 6 | 0 | 16 | 0 | 2 | 0 |
| 8/3/2007 | 26.73 | 22.77 | 8 | 0 | 15 | 2 | 1 | 0 |
| 8/4/2007 | 26.65 | 22.59 | 4 | 0 | 2 | 3 | 0 | 1 |
| 8/5/2007 | 26.67 | 22.09 | 2 | 0 | 5 | 1 | 1 | 0 |
| 8/6/2007 | 26.28 | 20.73 | 0 | 0 | 8 | 2 | 0 | 0 |
| 8/7/2007 | 25.99 | 19.47 | 0 | 0 | 1 | 0 | 0 | 0 |
| 8/8/2007 | 25.63 | 20.52 | 3 | 0 | 5 | 0 | 0 | 0 |
| 8/9/2007 | 25.43 | 21.26 | 4 | 0 | 9 | 0 | 1 | 0 |
| 8/10/2007 | 25.40 | 20.96 | 2 | 0 | 7 | 2 | 0 | 0 |
| 8/11/2007 | 25.34 | 21.06 | 3 | 0 | 6 | 0 | 0 | 0 |
| 8/12/2007 | 25.32 | 20.57 | 10 | 0 | 19 | 0 | 0 | 0 |
| 8/13/2007 | 25.23 | 20.64 | 10 | 0 | 25 | 0 | 0 | 0 |
| 8/14/2007 | 25.15 | 21.09 | 12 | 0 | 14 | 0 | 0 | 0 |
| 8/15/2007 | 25.12 | 21.51 | 5 | 0 | 20 | 0 | 0 | 0 |
| 8/16/2007 | 24.81 | 21.36 | 6 | 0 | 29 | 0 | 0 | 0 |
| 8/17/2007 | 24.41 | 20.51 | 7 | 0 | 14 | 0 | 0 | 0 |
| 8/18/2007 | 24.38 | 20.82 | 1 | 0 | 19 | 1 | 0 | 0 |
| 8/19/2007 | 26.76 | 20.24 | 2 | 0 | 16 | 0 | 0 | 0 |
| 8/20/2007 | 30.30 | 19.71 | 1 | 0 | 3 | 0 | 0 | 0 |
| 8/21/2007 | 28.32 | 19.14 | 0 | 0 | 0 | 0 | 0 | 0 |
| 8/22/2007 | 27.04 | 19.71 | 0 | 0 | 0 | 0 | 0 | 0 |
| 8/23/2007 | 26.56 | 19.49 | 1 | 0 | 0 | 0 | 0 | 0 |
| 8/24/2007 | 26.02 | 20.62 | 3 | 0 | 51 | 0 | 0 | 0 |
| 8/25/2007 | 25.68 | 21.16 | 1 | 0 | 12 | 1 | 1 | 0 |
| 8/26/2007 | 25.32 | 21.39 | 11 | 0 | 28 | 0 | 0 | 0 |
| 8/27/2007 | 24.95 | 20.18 | 4 | 0 | 12 | 0 | 0 | 0 |
| 8/28/2007 | 24.81 | 20.22 | 3 | 0 | 22 | 0 | 0 | 0 |
| 8/29/2007 | 24.72 | 20.19 | 4 | 0 | 14 | 0 | 0 | 0 |
| 8/30/2007 | 24.58 | 21.58 | 0 | 0 | 9 | 0 | 0 | 0 |
| 8/31/2007 | 24.52 | 21.36 | 0 | 0 | 7 | 1 | 0 | 0 |
| 9/1/2007 | 24.32 | 21.20 | 2 | 0 | 9 | 1 | 0 | 0 |
| 9/2/2007 | 24.24 | 20.82 | 4 | 0 | 16 | 0 | 0 | 0 |
| 9/3/2007 | 24.21 | 21.09 | 3 | 0 | 5 | 0 | 0 | 0 |
| 9/4/2007 | 24.86 | 21.22 | 3 | 0 | 6 | 3 | 0 | 0 |
| 9/5/2007 | 25.20 | 20.11 | 5 | 1 | 11 | 0 | 0 | 0 |
| 9/6/2007 | 25.32 | 19.55 | 2 | 0 | 9 | 1 | 0 | 0 |
| 9/7/2007 | 24.83 | 19.40 | 2 | 0 | 3 | 0 | 0 | 0 |
| 9/8/2007 | 24.55 | 19.08 | 1 | 0 | 6 | 0 | 0 | 0 |
| 9/9/2007 | 24.38 | 18.89 | 0 | 0 | 6 | 0 | 0 | 0 |
| 9/10/2007 | 24.18 | 18.75 | 1 | 0 | 8 | 0 | 0 | 0 |
| 9/11/2007 | 24.07 | 18.77 | 4 | 0 | 6 | 0 | 0 | 0 |
| 9/12/2007 | 24.07 | 18.60 | 3 | 0 | 9 | 0 | 0 | 0 |
| 9/13/2007 | 24.15 | 18.14 | 0 | 0 | 4 | 0 | 0 | 0 |
| 9/14/2007 | 24.27 | 17.10 | 0 | 1 | 3 | 0 | 0 | 0 |

| Date | Discharge (cms) | Temp (C°) | Chinook | Coho | Steelhead | Cutthroat | Lamprey | Sucker |
|---|---|---|---|---|---|---|---|---|
| 9/15/2007 | 24.35 | 17.24 | 1 | 1 | 4 | 0 | 0 | 0 |
| 9/16/2007 | 24.30 | 17.25 | 0 | 1 | 6 | 0 | 0 | 0 |
| 9/17/2007 | 24.52 | 16.65 | 1 | 0 | 4 | 0 | 0 | 0 |
| 9/18/2007 | 24.52 | 16.60 | 1 | 1 | 1 | 0 | 0 | 0 |
| 9/19/2007 | 24.49 | 16.23 | 0 | 0 | 0 | 0 | 0 | 0 |
| 9/20/2007 | 24.32 | 16.00 | 1 | 2 | 4 | 1 | 0 | 0 |
| 9/21/2007 | 24.38 | 15.59 | 1 | 1 | 2 | 0 | 0 | 0 |
| 9/22/2007 | 24.32 | 15.45 | 0 | 5 | 1 | 0 | 0 | 1 |
| 9/23/2007 | 24.18 | 15.19 | 2 | 4 | 2 | 0 | 0 | 0 |
| 9/24/2007 | 24.13 | 14.49 | 0 | 0 | 3 | 0 | 0 | 0 |
| 9/25/2007 | 24.07 | 14.51 | 0 | 2 | 1 | 0 | 0 | 0 |
| 9/26/2007 | 24.38 | 14.63 | 0 | 2 | 5 | 0 | 0 | 0 |
| 9/27/2007 | 24.52 | 14.95 | 2 | 2 | 3 | 0 | 0 | 0 |
| 9/28/2007 | 25.03 | 14.80 | 6 | 2 | 4 | 1 | 0 | 0 |
| 9/29/2007 | 25.88 | 13.23 | 2 | 7 | 5 | 0 | 0 | 0 |
| 9/30/2007 | 27.24 | 13.28 | 5 | 4 | 5 | 0 | 0 | 0 |
| 10/1/2007 | 31.15 | 12.73 | 2 | 4 | 3 | 0 | 0 | 0 |
| 10/2/2007 | 36.81 | 12.71 | 4 | 18 | 21 | 0 | 0 | 0 |
| 10/3/2007 | 30.87 | 13.49 | 6 | 11 | 26 | 1 | 0 | 0 |
| 10/4/2007 | 34.83 | 13.09 | 5 | 19 | 17 | 0 | 0 | 0 |
| 10/5/2007 | 41.34 | 12.35 | 5 | 25 | 11 | 0 | 0 | 1 |
| 10/6/2007 | 35.96 | 11.31 | 0 | 11 | 6 | 0 | 0 | 0 |
| 10/7/2007 | 31.15 | 11.57 | 0 | 11 | 3 | 0 | 0 | 0 |
| 10/8/2007 | 28.88 | 12.84 | 2 | 6 | 16 | 0 | 0 | 0 |
| 10/9/2007 | 28.32 | 12.84 | 1 | 12 | 8 | 0 | 0 | 0 |
| 10/10/2007 | 28.88 | 12.75 | 3 | 13 | 5 | 1 | 0 | 0 |
| 10/11/2007 | 30.30 | 12.28 | 4 | 17 | 5 | 1 | 0 | 0 |
| 10/12/2007 | 31.43 | 12.25 | 2 | 15 | 5 | 0 | 0 | 0 |
| 10/13/2007 | 30.58 | 12.37 | 1 | 10 | 5 | 0 | 0 | 0 |
| 10/14/2007 | 29.73 | 12.35 | 2 | 13 | 1 | 0 | 0 | 0 |
| 10/15/2007 | 30.02 | 12.17 | 1 | 2 | 1 | 0 | 0 | 0 |
| 10/16/2007 | 31.15 | 11.44 | 0 | 0 | 0 | 0 | 0 | 0 |
| 10/17/2007 | 37.10 | 10.63 | 1 | 23 | 10 | 0 | 0 | 0 |
| 10/18/2007 | 49.55 | 10.29 | 12 | 58 | 16 | 0 | 0 | 3 |
| 10/19/2007 | 229.08 | 10.56 | 8 | 63 | 18 | 0 | 1 | 3 |
| 10/20/2007 | 169.62 | 10.70 | 8 | 77 | 8 | 0 | 0 | 0 |
| 10/21/2007 | 113.55 | 9.93 | 7 | 138 | 11 | 0 | 0 | 0 |
| 10/22/2007 | 80.70 | 9.82 | 0 | 48 | 1 | 0 | 0 | 0 |
| 10/23/2007 | 64.28 | 10.05 | 0 | 0 | 0 | 2 | 0 | 0 |
| 10/24/2007 | 55.50 | 10.42 | 18 | 211 | 27 | 1 | 0 | 0 |
| 10/25/2007 | 49.84 | 9.93 | 0 | 67 | 11 | 0 | 0 | 0 |
| 10/26/2007 | 45.59 | 9.56 | 1 | 43 | 4 | 0 | 0 | 0 |
| 10/27/2007 | 41.91 | 9.44 | 5 | 11 | 1 | 0 | 0 | 0 |
| 10/28/2007 | 39.64 | 8.93 | 3 | 6 | 0 | 0 | 0 | 0 |
| 10/29/2007 | 38.51 | 8.74 | 1 | 5 | 4 | 1 | 0 | 0 |

| Date | Discharge (cms) | Temp (C°) | Chinook | Coho | Steelhead | Cutthroat | Lamprey | Sucker |
|---|---|---|---|---|---|---|---|---|
| 10/30/2007 | 37.66 | 9.15 | 2 | 21 | 4 | 0 | 0 | 0 |
| 10/31/2007 | 37.10 | 9.85 | 1 | 30 | 14 | 0 | 0 | 0 |
| 11/1/2007 | 35.96 | 9.91 | 3 | 18 | 2 | 0 | 0 | 0 |
| 11/2/2007 | 35.40 | 9.40 | 2 | 3 | 2 | 0 | 0 | 0 |
| 11/3/2007 | 34.55 | 9.19 | 0 | 4 | 3 | 0 | 0 | 0 |
| 11/4/2007 | 33.13 | 8.37 | 2 | 2 | 1 | 0 | 0 | 0 |
| 11/5/2007 | 32.00 | 7.96 | 0 | 2 | 0 | 0 | 0 | 0 |
| 11/6/2007 | 31.15 | 8.13 | 2 | 2 | 3 | 0 | 0 | 0 |
| 11/7/2007 | 30.58 | 8.17 | 0 | 2 | 0 | 0 | 0 | 0 |
| 11/8/2007 | 31.15 | 8.14 | 1 | 1 | 0 | 0 | 0 | 0 |
| 11/9/2007 | 31.43 | 8.45 | 1 | 8 | 4 | 1 | 0 | 0 |
| 11/10/2007 | 32.85 | 8.51 | 2 | 1 | 1 | 0 | 0 | 0 |
| 11/11/2007 | 39.08 | 8.46 | 1 | 5 | 6 | 0 | 0 | 0 |
| 11/12/2007 | 38.51 | 8.79 | 2 | 25 | 4 | 0 | 0 | 0 |
| 11/13/2007 | 86.37 | 8.81 | 2 | 67 | 34 | 0 | 0 | 0 |
| 11/14/2007 | 85.23 | 8.32 | 1 | 310 | 28 | 0 | 0 | 0 |
| 11/15/2007 | 60.03 | 8.15 | 1 | 114 | 28 | 4 | 0 | 0 |
| 11/16/2007 | 53.80 | 8.52 | 1 | 18 | 15 | 0 | 0 | 0 |
| 11/17/2007 | 230.22 | 9.61 | 3 | 47 | 22 | 0 | 0 | 0 |
| 11/18/2007 | 351.13 | 10.45 | 2 | 60 | 14 | 0 | 0 | 0 |
| 11/19/2007 | 396.44 | 9.80 | 0 | 12 | 1 | 1 | 0 | 0 |
| 11/20/2007 | 237.01 | 8.75 | 0 | 9 | 3 | 0 | 0 | 0 |
| 11/21/2007 | 152.06 | 7.83 | 0 | 4 | 5 | 0 | 0 | 0 |
| 11/22/2007 | 109.87 | 6.92 | 0 | 3 | 12 | 0 | 0 | 0 |
| 11/23/2007 | 89.76 | 5.85 | 1 | 1 | 1 | 0 | 0 | 0 |
| 11/24/2007 | 78.44 | 5.09 | 0 | 0 | 2 | 0 | 0 | 0 |
| 11/25/2007 | 70.51 | 4.91 | 0 | 0 | 10 | 0 | 0 | 0 |
| 11/26/2007 | 64.28 | 5.24 | 0 | 0 | 6 | 0 | 0 | 0 |
| 11/27/2007 | 63.43 | 5.44 | 0 | 0 | 4 | 0 | 0 | 0 |
| 11/28/2007 | 60.60 | 5.67 | 0 | 1 | 0 | 1 | 0 | 0 |
| 11/29/2007 | 61.16 | 5.94 | 0 | 3 | 11 | 0 | 0 | 0 |
| 11/30/2007 | 77.87 | 5.85 | 0 | 4 | 8 | 0 | 0 | 0 |
| 12/1/2007 | 72.21 | 5.31 | 0 | 0 | 7 | 0 | 0 | 0 |
| 12/2/2007 | 64.56 | 5.46 | 0 | 3 | 6 | 0 | 0 | 0 |
| 12/3/2007 | 76.46 | 6.90 | 0 | 18 | 9 | 0 | 0 | 0 |
| 12/4/2007 | 152.63 | 7.86 | 1 | 6 | 38 | 0 | 0 | 0 |
| 12/5/2007 | 135.35 | 8.69 | 0 | 10 | 83 | 2 | 0 | 0 |
| 12/6/2007 | 108.74 | 8.38 | 0 | 7 | 39 | 0 | 0 | 0 |
| 12/7/2007 | 127.14 | 8.08 | 0 | 2 | 44 | 0 | 0 | 0 |
| 12/8/2007 | 120.35 | 7.41 | 0 | 0 | 27 | 0 | 0 | 0 |
| 12/9/2007 | 102.22 | 6.49 | 0 | 0 | 12 | 0 | 0 | 0 |
| 12/10/2007 | 89.20 | 5.39 | 0 | 0 | 5 | 0 | 0 | 0 |
| 12/11/2007 | 79.29 | 4.91 | 0 | 0 | 4 | 0 | 0 | 0 |
| 12/12/2007 | 71.64 | 4.58 | 0 | 0 | 3 | 0 | 0 | 0 |
| 12/13/2007 | 65.98 | 4.51 | 0 | 0 | 5 | 0 | 0 | 0 |

| Date | Discharge (cms) | Temp (C°) | Chinook | Coho | Steelhead | Cutthroat | Lamprey | Sucker |
|---|---|---|---|---|---|---|---|---|
| 12/14/2007 | 60.88 | 4.52 | 0 | 0 | 2 | 0 | 0 | 0 |
| 12/15/2007 | 58.05 | 4.88 | 1 | 0 | 3 | 1 | 0 | 0 |
| 12/16/2007 | 56.63 | 4.97 | 0 | 0 | 6 | 0 | 0 | 0 |
| 12/17/2007 | 63.71 | 5.20 | 0 | 0 | 16 | 0 | 0 | 0 |
| 12/18/2007 | 79.85 | 5.94 | 0 | 0 | 28 | 0 | 0 | 0 |
| 12/19/2007 | 132.81 | 6.22 | 0 | 0 | 64 | 0 | 0 | 0 |
| 12/20/2007 | 328.48 | 6.72 | 0 | 0 | 16 | 0 | 0 | 0 |
| 12/21/2007 | 207.28 | 6.26 | 0 | 1 | 18 | 0 | 0 | 0 |
| 12/22/2007 | 141.58 | 5.63 | 0 | 0 | 26 | 0 | 0 | 0 |
| 12/23/2007 | 150.93 | 6.29 | 0 | 0 | 60 | 0 | 0 | 0 |
| 12/24/2007 | 498.38 | 6.92 | 0 | 1 | 17 | 0 | 0 | 0 |
| 12/25/2007 | 300.16 | 6.14 | 0 | 0 | 11 | 0 | 0 | 0 |
| 12/26/2007 | 206.71 | 5.49 | 0 | 0 | 8 | 0 | 0 | 0 |
| 12/27/2007 | 161.12 | 4.98 | 0 | 0 | 9 | 0 | 0 | 0 |
| 12/28/2007 | 146.11 | 5.06 | 0 | 0 | 25 | 0 | 0 | 0 |
| 12/29/2007 | 172.17 | 5.63 | 0 | 0 | 65 | 0 | 0 | 0 |
| 12/30/2007 | 265.90 | 6.08 | 0 | 0 | 20 | 0 | 0 | 0 |
| 12/31/2007 | 199.35 | 5.90 | 0 | 0 | 19 | 0 | 0 | 0 |
| 1/1/2008 | 146.96 | 5.04 | 0 | 0 | 9 | 0 | 0 | 0 |
| 1/2/2008 | 126.01 | 5.03 | 0 | 0 | 36 | 0 | 0 | 0 |
| 1/3/2008 | 126.01 | 5.77 | 0 | 0 | 150 | 0 | 0 | 0 |
| 1/4/2008 | 269.58 | 6.18 | 0 | 0 | 39 | 0 | 0 | 0 |
| 1/5/2008 | 464.40 | 6.65 | 0 | 0 | 13 | 0 | 0 | 0 |
| 1/6/2008 | 294.50 | 6.20 | 0 | 0 | 14 | 0 | 0 | 0 |
| 1/7/2008 | 217.19 | 5.59 | 0 | 0 | 18 | 0 | 0 | 0 |
| 1/8/2008 | 262.50 | 5.46 | 0 | 0 | 8 | 0 | 0 | 0 |
| 1/9/2008 | 345.47 | 5.94 | 0 | 0 | 65 | 0 | 0 | 0 |
| 1/10/2008 | 319.98 | 6.71 | 0 | 0 | 40 | 0 | 0 | 0 |
| 1/11/2008 | 450.24 | 6.83 | 0 | 0 | 64 | 0 | 0 | 0 |
| 1/12/2008 | 368.12 | 7.10 | 0 | 0 | 42 | 0 | 0 | 0 |
| 1/13/2008 | 359.62 | 6.91 | 0 | 0 | 28 | 0 | 0 | 0 |
| 1/14/2008 | 262.50 | 6.16 | 0 | 0 | 25 | 0 | 0 | 0 |
| 1/15/2008 | 234.18 | 6.11 | 0 | 0 | 43 | 0 | 0 | 0 |
| 1/16/2008 | 196.24 | 5.15 | 0 | 0 | 10 | 0 | 0 | 0 |
| 1/17/2008 | 156.59 | 4.11 | 0 | 0 | 2 | 0 | 0 | 0 |
| 1/18/2008 | 133.09 | 4.11 | 0 | 0 | 6 | 0 | 0 | 0 |
| 1/19/2008 | 117.80 | 4.28 | 0 | 0 | 14 | 0 | 0 | 0 |
| 1/20/2008 | 111.57 | 4.80 | 0 | 0 | 21 | 0 | 0 | 0 |
| 1/21/2008 | 104.21 | 4.72 | 0 | 0 | 6 | 0 | 0 | 0 |
| 1/22/2008 | 93.45 | 3.34 | 0 | 0 | 0 | 0 | 0 | 0 |
| 1/23/2008 | 86.37 | 1.87 | 0 | 0 | 0 | 0 | 0 | 0 |
| 1/24/2008 | 81.55 | 1.31 | 0 | 0 | 0 | 0 | 0 | 0 |
| 1/25/2008 | 78.44 | 2.21 | 0 | 0 | 0 | 0 | 0 | 0 |
| 1/26/2008 | 75.32 | 2.94 | 0 | 0 | 2 | 0 | 0 | 0 |
| 1/27/2008 | 124.59 | 3.10 | 0 | 0 | 5 | 0 | 0 | 0 |

| Date | Discharge (cms) | Temp (C°) | Chinook | Coho | Steelhead | Cutthroat | Lamprey | Sucker |
|---|---|---|---|---|---|---|---|---|
| 1/28/2008 | 165.37 | 3.72 | 0 | 0 | 8 | 0 | 0 | 0 |
| 1/29/2008 | 154.61 | 4.10 | 0 | 0 | 4 | 0 | 0 | 0 |
| 1/30/2008 | 148.66 | 3.61 | 0 | 0 | 3 | 0 | 0 | 0 |
| 1/31/2008 | 195.95 | 4.07 | 0 | 0 | 1 | 0 | 0 | 0 |
| 2/1/2008 | 246.36 | 4.90 | 0 | 0 | 22 | 0 | 0 | 0 |
| 2/2/2008 | 201.90 | 4.99 | 0 | 0 | 60 | 0 | 0 | 0 |
| 2/3/2008 | 250.04 | 4.66 | 0 | 0 | 5 | 0 | 0 | 0 |
| 2/4/2008 | 170.75 | 5.20 | 0 | 0 | 58 | 0 | 0 | 0 |
| 2/5/2008 | 133.37 | 5.25 | 0 | 0 | 147 | 0 | 0 | 0 |
| 2/6/2008 | 130.82 | 5.49 | 0 | 0 | 140 | 0 | 0 | 0 |
| 2/7/2008 | 160.84 | 5.86 | 0 | 0 | 237 | 0 | 0 | 0 |
| 2/8/2008 | 218.32 | 6.00 | 0 | 0 | 123 | 0 | 0 | 0 |
| 2/9/2008 | 165.94 | 6.42 | 0 | 0 | 245 | 0 | 0 | 0 |
| 2/10/2008 | 157.72 | 5.97 | 0 | 0 | 148 | 0 | 0 | 0 |
| 2/11/2008 | 171.60 | 6.23 | 0 | 0 | 2 | 0 | 0 | 0 |
| 2/12/2008 | 182.64 | 6.44 | 0 | 0 | 80 | 0 | 0 | 0 |
| 2/13/2008 | 204.16 | 6.51 | 0 | 0 | 141 | 0 | 0 | 0 |
| 2/14/2008 | 184.63 | 5.87 | 0 | 0 | 9 | 0 | 0 | 0 |
| 2/15/2008 | 150.08 | 5.28 | 0 | 0 | 1 | 0 | 0 | 0 |
| 2/16/2008 | 138.75 | 5.62 | 0 | 0 | 3 | 0 | 0 | 0 |
| 2/17/2008 | 148.95 | 5.49 | 0 | 0 | 2 | 0 | 0 | 0 |
| 2/18/2008 | 154.33 | 5.87 | 0 | 0 | 15 | 0 | 0 | 0 |
| 2/19/2008 | 168.49 | 5.62 | 0 | 0 | 16 | 0 | 0 | 0 |
| 2/20/2008 | 177.55 | 6.79 | 0 | 0 | 251 | 0 | 0 | 0 |
| 2/21/2008 | 174.71 | 6.26 | 0 | 0 | 85 | 0 | 0 | 0 |
| 2/22/2008 | 159.14 | 6.42 | 0 | 0 | 152 | 0 | 0 | 0 |
| 2/23/2008 | 144.13 | 6.27 | 0 | 0 | 31 | 0 | 0 | 0 |
| 2/24/2008 | 131.39 | 6.27 | 0 | 0 | 61 | 0 | 0 | 0 |
| 2/25/2008 | 133.09 | 7.16 | 0 | 0 | 323 | 0 | 0 | 0 |
| 2/26/2008 | 158.29 | 7.06 | 0 | 0 | 203 | 0 | 0 | 0 |
| 2/27/2008 | 157.72 | 7.39 | 0 | 0 | 246 | 0 | 0 | 0 |
| 2/28/2008 | 166.50 | 7.34 | 0 | 0 | 176 | 0 | 0 | 0 |
| 2/29/2008 | 177.55 | 7.05 | 0 | 0 | 185 | 1 | 0 | 0 |
| 3/1/2008 | 254.85 | 7.08 | 0 | 0 | 179 | 0 | 0 | 0 |
| 3/2/2008 | 238.14 | 7.07 | 0 | 0 | 92 | 0 | 0 | 0 |
| 3/3/2008 | 183.49 | 6.19 | 0 | 0 | 41 | 0 | 0 | 0 |
| 3/4/2008 | 154.04 | 7.01 | 0 | 0 | 132 | 0 | 0 | 0 |
| 3/5/2008 | 133.66 | 6.70 | 0 | 0 | 16 | 0 | 0 | 0 |
| 3/6/2008 | 119.78 | 5.98 | 0 | 0 | 6 | 0 | 0 | 0 |
| 3/7/2008 | 112.13 | 5.84 | 0 | 0 | 5 | 0 | 0 | 0 |
| 3/8/2008 | 115.82 | 7.61 | 0 | 0 | 113 | 0 | 0 | 0 |
| 3/9/2008 | 133.37 | 7.84 | 0 | 0 | 431 | 0 | 0 | 0 |
| 3/10/2008 | 139.32 | 7.64 | 0 | 0 | 248 | 1 | 0 | 0 |
| 3/11/2008 | 176.41 | 8.06 | 0 | 0 | 356 | 0 | 0 | 0 |
| 3/12/2008 | 179.81 | 7.58 | 0 | 0 | 151 | 0 | 0 | 0 |

| Date | Discharge (cms) | Temp (C°) | Chinook | Coho | Steelhead | Cutthroat | Lamprey | Sucker |
|---|---|---|---|---|---|---|---|---|
| 3/13/2008 | 178.68 | 7.51 | 0 | 0 | 153 | 0 | 0 | 0 |
| 3/14/2008 | 209.54 | 6.90 | 0 | 0 | 61 | 0 | 0 | 0 |
| 3/15/2008 | 231.63 | | 0 | 0 | 11 | 0 | 0 | 0 |
| 3/16/2008 | 214.64 | | 0 | 0 | 34 | 0 | 0 | 0 |
| 3/17/2008 | 180.66 | 6.76 | 0 | 0 | 21 | 1 | 0 | 0 |
| 3/18/2008 | 223.42 | 7.21 | 0 | 0 | 94 | 0 | 0 | 0 |
| 3/19/2008 | 487.05 | 7.15 | 0 | 0 | 9 | 0 | 0 | 0 |
| 3/20/2008 | 390.77 | 6.98 | 0 | 0 | 14 | 0 | 0 | 0 |
| 3/21/2008 | 279.77 | 7.38 | 0 | 0 | 32 | 0 | 0 | 0 |
| 3/22/2008 | 218.61 | 7.32 | 0 | 0 | 66 | 0 | 0 | 0 |
| 3/23/2008 | 188.02 | 7.50 | 0 | 0 | 230 | 0 | 0 | 0 |
| 3/24/2008 | 192.27 | 7.85 | 0 | 0 | 208 | 0 | 0 | 0 |
| 3/25/2008 | 190.57 | 7.67 | 0 | 0 | 63 | 0 | 0 | 0 |
| 3/26/2008 | 181.23 | 7.03 | 0 | 0 | 39 | 0 | 0 | 0 |
| 3/27/2008 | 175.85 | 6.54 | 0 | 0 | 2 | 0 | 0 | 0 |
| 3/28/2008 | 165.65 | 6.06 | 0 | 0 | 1 | 0 | 0 | 0 |
| 3/29/2008 | 156.03 | 5.61 | 0 | 0 | 2 | 0 | 0 | 0 |
| 3/30/2008 | 137.34 | 6.62 | 0 | 0 | 16 | 0 | 0 | 0 |
| 3/31/2008 | 122.33 | 6.61 | 0 | 0 | 6 | 0 | 0 | 0 |
| 4/1/2008 | 110.15 | 7.20 | 0 | 0 | 33 | 0 | 0 | 0 |
| 4/2/2008 | 107.04 | 7.69 | 0 | 0 | 47 | 1 | 0 | 0 |
| 4/3/2008 | 108.45 | 7.94 | 0 | 0 | 15 | 0 | 0 | 0 |
| 4/4/2008 | 114.40 | 7.88 | 0 | 0 | 54 | 0 | 0 | 0 |
| 4/5/2008 | 119.21 | 7.30 | 0 | 0 | 67 | 0 | 0 | 1 |
| 4/6/2008 | 115.82 | 7.89 | 0 | 0 | 189 | 0 | 0 | 0 |
| 4/7/2008 | 125.16 | 7.78 | 0 | 0 | 131 | 0 | 0 | 0 |
| 4/8/2008 | 126.01 | 7.96 | 0 | 0 | 78 | 0 | 0 | 0 |
| 4/9/2008 | 124.03 | 7.95 | 0 | 0 | 86 | 1 | 0 | 0 |
| 4/10/2008 | 126.86 | 8.97 | 0 | 0 | 116 | 0 | 0 | 27 |
| 4/11/2008 | 126.58 | 8.33 | 0 | 0 | 14 | 0 | 0 | 23 |
| 4/12/2008 | 142.15 | 9.91 | 0 | 0 | 163 | 2 | 0 | 306 |
| 4/13/2008 | 203.88 | 9.30 | 0 | 0 | 157 | 0 | 0 | 43 |
| 4/14/2008 | 233.61 | 8.34 | 0 | 0 | 24 | 0 | 0 | 0 |
| 4/15/2008 | 193.69 | 7.18 | 0 | 0 | 1 | 0 | 0 | 0 |
| 4/16/2008 | 154.04 | 7.99 | 0 | 0 | 7 | 0 | 0 | 0 |
| 4/17/2008 | 135.07 | 9.25 | 1 | 0 | 56 | 0 | 0 | 0 |
| 4/18/2008 | 138.75 | 8.75 | 1 | 0 | 112 | 1 | 0 | 1 |
| 4/19/2008 | 139.04 | 7.71 | 0 | 0 | 6 | 0 | 0 | 0 |
| 4/20/2008 | 132.52 | 6.46 | 0 | 0 | 2 | 0 | 0 | 0 |
| 4/21/2008 | 120.63 | 5.83 | 0 | 0 | 0 | 0 | 0 | 0 |
| 4/22/2008 | 112.13 | 6.15 | 0 | 0 | 0 | 0 | 0 | 0 |
| 4/23/2008 | 139.60 | 7.15 | 0 | 0 | 7 | 0 | 0 | 0 |
| 4/24/2008 | 150.93 | 7.98 | 0 | 0 | 27 | 0 | 0 | 0 |
| 4/25/2008 | 147.81 | 8.07 | 2 | 0 | 54 | 0 | 0 | 0 |
| 4/26/2008 | 141.30 | 9.21 | 1 | 0 | 63 | 0 | 0 | 40 |

| Date | Discharge (cms) | Temp (C°) | Chinook | Coho | Steelhead | Cutthroat | Lamprey | Sucker |
|---|---|---|---|---|---|---|---|---|
| 4/27/2008 | 163.11 | 9.73 | 1 | 0 | 97 | 0 | 0 | 66 |
| 4/28/2008 | 205.58 | 9.55 | 0 | 0 | 40 | 0 | 0 | 0 |
| 4/29/2008 | 222.00 | 9.01 | 2 | 0 | 25 | 0 | 0 | 0 |
| 4/30/2008 | 210.11 | 7.60 | 1 | 0 | 0 | 0 | 0 | 0 |
| 5/1/2008 | 184.63 | 7.74 | 0 | 0 | 0 | 1 | 0 | 0 |
| 5/2/2008 | 167.64 | 9.35 | 9 | 0 | 18 | 0 | 0 | 0 |
| 5/3/2008 | 167.07 | 10.20 | 20 | 0 | 21 | 0 | 0 | 12 |
| 5/4/2008 | 172.17 | 10.17 | 5 | 0 | 13 | 0 | 0 | 65 |
| 5/5/2008 | 184.34 | 10.88 | 17 | 0 | 19 | 0 | 0 | 78 |
| 5/6/2008 | 208.13 | 10.28 | 16 | 0 | 17 | 0 | 0 | 0 |
| 5/7/2008 | 205.86 | 9.30 | 2 | 0 | 4 | 0 | 0 | 14 |
| 5/8/2008 | 196.80 | 9.10 | 3 | 0 | 2 | 0 | 0 | 0 |
| 5/9/2008 | 176.70 | 8.93 | 0 | 0 | 1 | 0 | 0 | 0 |
| 5/10/2008 | 157.72 | 9.69 | 10 | 0 | 11 | 0 | 0 | 0 |
| 5/11/2008 | 161.41 | 10.14 | 38 | 0 | 15 | 0 | 0 | 0 |
| 5/12/2008 | 160.84 | 9.36 | 2 | 0 | 0 | 0 | 0 | 0 |
| 5/13/2008 | 150.08 | 9.63 | 0 | 0 | 0 | 0 | 0 | 0 |
| 5/14/2008 | 150.08 | 10.53 | 0 | 0 | 0 | 0 | 0 | 0 |
| 5/15/2008 | 179.53 | 10.66 | 14 | 0 | 29 | 0 | 1 | 378 |
| 5/16/2008 | 241.83 | 11.90 | 45 | 0 | 13 | 1 | 1 | 208 |
| 5/17/2008 | 286.00 | 11.02 | 19 | 0 | 5 | 0 | 0 | 0 |
| 5/18/2008 | 294.50 | 10.73 | 25 | 0 | 6 | 0 | 0 | 0 |
| 5/19/2008 | 262.21 | 10.39 | 0 | 0 | 0 | 0 | 0 | 0 |
| 5/20/2008 | 242.11 | 10.44 | 0 | 0 | 0 | 0 | 0 | 0 |
| 5/21/2008 | 215.49 | 9.18 | 0 | 0 | 0 | 0 | 0 | 0 |
| 5/22/2008 | 184.34 | 8.32 | 20 | 0 | 2 | 0 | 0 | 1 |
| 5/23/2008 | 160.27 | 8.50 | 1 | 0 | 2 | 0 | 0 | 0 |
| 5/24/2008 | 142.72 | 10.02 | 17 | 0 | 8 | 0 | 0 | 0 |
| 5/25/2008 | 133.94 | 10.33 | 121 | 0 | 27 | 1 | 0 | 0 |
| 5/26/2008 | 131.39 | 9.94 | 13 | 0 | 6 | 0 | 0 | 0 |
| 5/27/2008 | 127.99 | 10.56 | 11 | 0 | 0 | 0 | 0 | 1 |
| 5/28/2008 | 140.73 | 10.82 | 27 | 0 | 5 | 0 | 0 | 0 |
| 5/29/2008 | 173.30 | 9.48 | 10 | 0 | 8 | 0 | 0 | 3 |
| 5/30/2008 | 167.07 | 10.83 | 38 | 0 | 9 | 0 | 0 | 0 |
| 5/31/2008 | 154.04 | 10.43 | 197 | 0 | 38 | 0 | 0 | 1 |
| 6/1/2008 | 150.36 | 9.94 | 5 | 0 | 0 | 0 | 0 | 0 |
| 6/2/2008 | 141.02 | 11.48 | 49 | 0 | 17 | 0 | 0 | 0 |
| 6/3/2008 | 142.72 | 11.19 | 99 | 0 | 14 | 0 | 0 | 1 |
| 6/4/2008 | 194.82 | 10.28 | 18 | 0 | 4 | 0 | 0 | 0 |
| 6/5/2008 | 176.70 | 9.39 | 5 | 0 | 1 | 0 | 0 | 0 |
| 6/6/2008 | 155.18 | 9.40 | 12 | 0 | 2 | 0 | 0 | 0 |
| 6/7/2008 | 146.68 | 10.26 | 19 | 0 | 11 | 0 | 0 | 0 |
| 6/8/2008 | 141.02 | 11.38 | 44 | 0 | 27 | 0 | 0 | 2 |
| 6/9/2008 | 133.37 | 11.06 | 7 | 0 | 4 | 0 | 0 | 3 |
| 6/10/2008 | 124.59 | 10.37 | 48 | 0 | 6 | 0 | 0 | 2 |

| Date | Discharge (cms) | Temp (C°) | Chinook | Coho | Steelhead | Cutthroat | Lamprey | Sucker |
|---|---|---|---|---|---|---|---|---|
| 6/11/2008 | 115.25 | 9.73 | 3 | 0 | 0 | 0 | 0 | 3 |
| 6/12/2008 | 107.04 | 12.68 | 92 | 0 | 47 | 0 | 0 | 18 |
| 6/13/2008 | 103.64 | 13.62 | 100 | 0 | 49 | 0 | 1 | 371 |
| 6/14/2008 | 106.19 | 14.56 | 108 | 0 | 54 | 2 | 1 | 364 |
| 6/15/2008 | 108.17 | 14.72 | 104 | 0 | 111 | 0 | 2 | 440 |
| 6/16/2008 | 107.04 | 13.83 | 4 | 0 | 6 | 3 | 0 | 17 |
| 6/17/2008 | 106.19 | 14.73 | 71 | 0 | 151 | 2 | 0 | 380 |
| 6/18/2008 | 104.77 | 13.65 | 34 | 0 | 70 | 0 | 1 | 8 |
| 6/19/2008 | 96.28 | 13.66 | 27 | 0 | 11 | 0 | 0 | 73 |
| 6/20/2008 | 93.45 | 15.32 | 84 | 0 | 155 | 1 | 2 | 319 |
| 6/21/2008 | 92.03 | 15.11 | 93 | 0 | 123 | 1 | 0 | 176 |
| 6/22/2008 | 92.03 | 14.02 | 58 | 0 | 106 | 0 | 2 | 8 |
| 6/23/2008 | 88.91 | 14.25 | 3 | 0 | 12 | 1 | 0 | 0 |
| 6/24/2008 | 85.80 | 15.19 | 0 | 0 | 0 | 0 | 0 | 0 |
| 6/25/2008 | 82.69 | 15.47 | 24 | 0 | 47 | 1 | 0 | 151 |
| 6/26/2008 | 78.44 | 16.16 | 44 | 0 | 151 | 2 | 0 | 82 |
| 6/27/2008 | 75.61 | 16.27 | 35 | 0 | 125 | 2 | 0 | 376 |
| 6/28/2008 | 72.49 | 17.80 | 22 | 0 | 118 | 1 | 1 | 639 |
| 6/29/2008 | 74.19 | 18.73 | 44 | 0 | 158 | 1 | 0 | 309 |
| 6/30/2008 | 73.34 | 18.35 | 25 | 0 | 88 | 0 | 1 | 202 |
| 7/1/2008 | 72.21 | 18.57 | 5 | 0 | 18 | 1 | 0 | 0 |
| 7/2/2008 | 69.66 | 18.45 | 15 | 0 | 64 | 4 | 0 | 342 |
| 7/3/2008 | 67.11 | 18.28 | 37 | 0 | 136 | 2 | 0 | 239 |
| 7/4/2008 | 63.71 | 18.83 | 28 | 0 | 89 | 2 | 0 | 462 |
| 7/5/2008 | 58.90 | 19.15 | 22 | 0 | 48 | 2 | 1 | 207 |
| 7/6/2008 | 55.22 | 19.14 | 13 | 0 | 33 | 2 | 0 | 78 |
| 7/7/2008 | 53.80 | 19.27 | 3 | 0 | 15 | 1 | 1 | 7 |
| 7/8/2008 | 52.95 | 20.12 | 0 | 0 | 0 | 1 | 0 | 0 |
| 7/9/2008 | 52.10 | 20.43 | 12 | 0 | 6 | 1 | 0 | 345 |
| 7/10/2008 | 50.97 | 20.53 | 6 | 0 | 111 | 4 | 1 | 69 |
| 7/11/2008 | 51.54 | 19.88 | 8 | 0 | 51 | 7 | 1 | 16 |
| 7/12/2008 | 50.97 | 19.84 | 3 | 0 | 39 | 6 | 0 | 45 |
| 7/13/2008 | 48.14 | 20.19 | 7 | 0 | 36 | 2 | 2 | 10 |
| 7/14/2008 | 45.31 | 20.41 | 10 | 0 | 38 | 10 | 1 | 38 |
| 7/15/2008 | 44.46 | 20.66 | 3 | 0 | 9 | 6 | 2 | 9 |
| 7/16/2008 | 43.61 | 20.65 | 1 | 0 | 10 | 5 | 0 | 3 |
| 7/17/2008 | 42.76 | 20.93 | 1 | 0 | 7 | 1 | 1 | 0 |
| 7/18/2008 | 42.48 | 20.76 | 1 | 0 | 11 | 2 | 0 | 4 |
| 7/19/2008 | 40.78 | 20.36 | 4 | 0 | 3 | 2 | 0 | 0 |
| 7/20/2008 | 39.08 | 19.81 | 6 | 0 | 6 | 0 | 0 | 1 |
| 7/21/2008 | 38.79 | 20.16 | 10 | 0 | 17 | 2 | 1 | 3 |
| 7/22/2008 | 39.64 | 19.53 | 18 | 0 | 36 | 3 | 0 | 0 |
| 7/23/2008 | 39.64 | 18.37 | 6 | 0 | 14 | 1 | 0 | 0 |
| 7/24/2008 | 39.36 | 19.13 | 6 | 0 | 33 | 0 | 0 | 1 |
| 7/25/2008 | 39.08 | 19.45 | 7 | 0 | 49 | 2 | 1 | 0 |

| Date | Discharge (cms) | Temp (C°) | Chinook | Coho | Steelhead | Cutthroat | Lamprey | Sucker |
|---|---|---|---|---|---|---|---|---|
| 7/26/2008 | 37.94 | 19.59 | 11 | 0 | 46 | 1 | 0 | 0 |
| 7/27/2008 | 36.81 | 20.09 | 17 | 0 | 84 | 7 | 0 | 1 |
| 7/28/2008 | 36.25 | 19.99 | 11 | 0 | 43 | 2 | 0 | 0 |
| 7/29/2008 | 35.68 | 19.88 | 17 | 0 | 28 | 4 | 0 | 3 |
| 7/30/2008 | 35.11 | 19.34 | 4 | 0 | 38 | 4 | 0 | 2 |
| 7/31/2008 | 34.55 | 19.77 | 9 | 0 | 35 | 4 | 1 | 0 |
| 8/1/2008 | 35.11 | 19.99 | 10 | 0 | 42 | 1 | 0 | 2 |
| 8/2/2008 | 33.70 | 19.93 | 6 | 0 | 27 | 1 | 0 | 0 |
| 8/3/2008 | 32.85 | 19.30 | 2 | 0 | 14 | 0 | 0 | 0 |
| 8/4/2008 | 32.28 | 19.72 | 8 | 0 | 13 | 3 | 0 | 2 |
| 8/5/2008 | 31.71 | 20.09 | 7 | 0 | 19 | 1 | 0 | 2 |
| 8/6/2008 | 31.15 | 20.84 | 6 | 0 | 37 | 1 | 0 | 0 |
| 8/7/2008 | 30.87 | 20.92 | 9 | 0 | 33 | 3 | 0 | 0 |
| 8/8/2008 | 30.58 | 20.40 | 4 | 0 | 18 | 5 | 0 | 0 |
| 8/9/2008 | 30.30 | 20.16 | 5 | 0 | 29 | 0 | 0 | 1 |
| 8/10/2008 | 30.30 | 19.79 | 7 | 0 | 11 | 0 | 0 | 0 |
| 8/11/2008 | 30.02 | 20.25 | 5 | 0 | 19 | 0 | 1 | 0 |
| 8/12/2008 | 30.02 | 20.68 | 12 | 0 | 29 | 5 | 0 | 0 |
| 8/13/2008 | 29.73 | 21.01 | 12 | 0 | 17 | 3 | 0 | 0 |
| 8/14/2008 | 30.02 | 21.59 | 15 | 0 | 16 | 2 | 0 | 2 |
| 8/15/2008 | 31.15 | 22.35 | 13 | 0 | 16 | 1 | 1 | 1 |
| 8/16/2008 | 32.00 | 22.69 | 17 | 0 | 14 | 1 | 0 | 0 |
| 8/17/2008 | 32.56 | 22.86 | 18 | 0 | 22 | 0 | 0 | 1 |
| 8/18/2008 | 32.85 | 20.35 | 15 | 0 | 7 | 0 | 0 | 0 |
| 8/19/2008 | 32.28 | 19.11 | 0 | 0 | 3 | 0 | 0 | 0 |
| 8/20/2008 | 31.71 | 18.89 | 0 | 0 | 1 | 0 | 0 | 0 |
| 8/21/2008 | 31.71 | 18.44 | 1 | 0 | 0 | 0 | 0 | 0 |
| 8/22/2008 | 32.56 | 18.00 | 4 | 0 | 0 | 0 | 0 | 0 |
| 8/23/2008 | 31.15 | 18.56 | 3 | 0 | 3 | 0 | 0 | 0 |
| 8/24/2008 | 30.87 | 19.22 | 10 | 0 | 6 | 1 | 0 | 0 |
| 8/25/2008 | 30.58 | 19.15 | 13 | 0 | 12 | 2 | 0 | 0 |
| 8/26/2008 | 29.73 | 18.33 | 6 | 0 | 9 | 0 | 0 | 0 |
| 8/27/2008 | 28.60 | 18.50 | 4 | 0 | 14 | 0 | 0 | 0 |
| 8/28/2008 | 28.32 | 18.59 | 16 | 0 | 13 | 2 | 0 | 0 |
| 8/29/2008 | 27.75 | 19.08 | 9 | 0 | 19 | 2 | 0 | 0 |
| 8/30/2008 | 27.81 | 19.47 | 20 | 0 | 15 | 2 | 0 | 0 |
| 8/31/2008 | 27.98 | 18.06 | 7 | 0 | 5 | 0 | 0 | 0 |
| 9/1/2008 | 28.88 | 17.24 | 1 | 0 | 5 | 0 | 0 | 0 |
| 9/2/2008 | 29.45 | 16.71 | 2 | 0 | 8 | 1 | 0 | 0 |
| 9/3/2008 | 29.73 | 16.61 | 3 | 1 | 4 | 0 | 0 | 0 |
| 9/4/2008 | 30.30 | 16.90 | 8 | 0 | 5 | 0 | 0 | 0 |
| 9/5/2008 | 30.87 | 17.18 | 8 | 1 | 12 | 3 | 0 | 0 |
| 9/6/2008 | 30.30 | 17.44 | 1 | 1 | 9 | 0 | 0 | 0 |
| 9/7/2008 | 30.02 | 17.65 | 7 | 0 | 15 | 1 | 0 | 0 |
| 9/8/2008 | 29.73 | 17.94 | 12 | 2 | 16 | 2 | 0 | 0 |

| Date | Discharge (cms) | Temp (C°) | Chinook | Coho | Steelhead | Cutthroat | Lamprey | Sucker |
|---|---|---|---|---|---|---|---|---|
| 9/9/2008 | 30.02 | 17.88 | 2 | 5 | 15 | 0 | 1 | 0 |
| 9/10/2008 | 29.73 | 17.57 | 4 | 4 | 6 | 0 | 0 | 0 |
| 9/11/2008 | 29.45 | 17.24 | 1 | 3 | 3 | 0 | 0 | 0 |
| 9/12/2008 | 28.88 | 17.18 | 1 | 2 | 4 | 0 | 0 | 0 |
| 9/13/2008 | 28.32 | 17.03 | 0 | 4 | 3 | 0 | 0 | 0 |
| 9/14/2008 | 26.08 | 16.87 | 3 | 2 | 3 | 0 | 0 | 0 |
| 9/15/2008 | 25.51 | 17.13 | 1 | 3 | 2 | 0 | 0 | 0 |
| 9/16/2008 | 25.66 | 17.22 | 4 | 1 | 5 | 0 | 0 | 0 |
| 9/17/2008 | 26.19 | 17.63 | 2 | 5 | 0 | 0 | 0 | 0 |
| 9/18/2008 | 26.31 | 17.25 | 0 | 0 | 0 | 0 | 0 | 0 |
| 9/19/2008 | 26.53 | 15.82 | 0 | 7 | 3 | 0 | 0 | 0 |
| 9/20/2008 | 26.96 | 15.86 | 3 | 10 | 10 | 1 | 0 | 0 |
| 9/21/2008 | 27.69 | 15.63 | 2 | 8 | 4 | 0 | 0 | 0 |
| 9/22/2008 | 27.89 | 15.15 | 1 | 6 | 10 | 0 | 0 | 0 |
| 9/23/2008 | 27.92 | 14.33 | 3 | 7 | 3 | 0 | 0 | 0 |
| 9/24/2008 | 28.18 | 14.44 | 2 | 3 | 9 | 1 | 0 | 0 |
| 9/25/2008 | 28.29 | 14.71 | 2 | 9 | 5 | 0 | 0 | 0 |
| 9/26/2008 | 28.15 | 14.55 | 3 | 12 | 7 | 0 | 0 | 0 |
| 9/27/2008 | 27.89 | 14.26 | 6 | 8 | 4 | 0 | 0 | 0 |
| 9/28/2008 | 27.89 | 14.16 | 6 | 8 | 2 | 0 | 0 | 0 |
| 9/29/2008 | 27.64 | 14.00 | 3 | 8 | 2 | 0 | 0 | 0 |
| 9/30/2008 | 27.21 | 14.34 | 0 | 0 | 0 | 0 | 0 | 0 |
| 10/1/2008 | 27.04 | 14.48 | 5 | 20 | 19 | 1 | 0 | 0 |
| 10/2/2008 | 27.35 | 14.37 | 5 | 19 | 10 | 1 | 0 | 0 |
| 10/3/2008 | 28.09 | 14.19 | 6 | 32 | 13 | 0 | 0 | 0 |
| 10/4/2008 | 30.58 | 13.53 | 12 | 57 | 16 | 2 | 0 | 2 |
| 10/5/2008 | 45.31 | 13.12 | 23 | 61 | 23 | 0 | 0 | 0 |
| 10/6/2008 | 38.51 | 13.64 | 28 | 68 | 33 | 0 | 0 | 6 |
| 10/7/2008 | 33.13 | 14.44 | 19 | 56 | 17 | 1 | 0 | 5 |
| 10/8/2008 | 30.87 | 13.32 | 2 | 37 | 15 | 1 | 0 | 0 |
| 10/9/2008 | 29.73 | 12.25 | 0 | 22 | 4 | 0 | 0 | 0 |
| 10/10/2008 | 29.73 | 10.52 | 1 | 6 | 1 | 0 | 0 | 0 |
| 10/11/2008 | 30.58 | 9.56 | 0 | 3 | 1 | 0 | 0 | 0 |
| 10/12/2008 | 30.58 | 9.37 | 0 | 3 | 1 | 0 | 0 | 0 |
| 10/13/2008 | 30.02 | 9.28 | 0 | 1 | 0 | 0 | 0 | 0 |
| 10/14/2008 | 30.30 | 9.34 | 2 | 5 | 2 | 0 | 0 | 0 |
| 10/15/2008 | 29.73 | 9.13 | 1 | 5 | 5 | 0 | 0 | 0 |
| 10/16/2008 | 32.00 | 10.42 | 1 | 12 | 5 | 0 | 0 | 0 |
| 10/17/2008 | 29.45 | 10.84 | 5 | 41 | 19 | 1 | 0 | 0 |
| 10/18/2008 | 28.32 | 10.77 | 8 | 39 | 10 | 0 | 0 | 0 |
| 10/19/2008 | 28.32 | 10.90 | 1 | 13 | 11 | 1 | 0 | 0 |
| 10/20/2008 | 28.60 | 11.22 | 0 | 7 | 2 | 0 | 0 | 0 |
| 10/21/2008 | 28.60 | 10.93 | 0 | 0 | 0 | 0 | 0 | 0 |
| 10/22/2008 | 28.88 | 10.52 | 0 | 0 | 0 | 0 | 0 | 0 |
| 10/23/2008 | 28.60 | 9.93 | 1 | 12 | 0 | 0 | 0 | 0 |

| Date | Discharge (cms) | Temp (C°) | Chinook | Coho | Steelhead | Cutthroat | Lamprey | Sucker |
|---|---|---|---|---|---|---|---|---|
| 10/24/2008 | 27.69 | 9.29 | 0 | 15 | 1 | 0 | 0 | 0 |
| 10/25/2008 | 27.64 | 8.89 | 0 | 4 | 4 | 0 | 0 | 0 |
| 10/26/2008 | 27.64 | 9.15 | 0 | 4 | 1 | 1 | 0 | 0 |
| 10/27/2008 | 27.61 | 9.21 | 0 | 7 | 2 | 0 | 0 | 0 |
| 10/28/2008 | 27.58 | 9.21 | 0 | 11 | 8 | 0 | 0 | 0 |
| 10/29/2008 | 27.52 | 9.78 | 0 | 11 | 10 | 0 | 0 | 0 |
| 10/30/2008 | 27.52 | 9.87 | 1 | 17 | 6 | 0 | 0 | 0 |
| 10/31/2008 | 27.86 | 9.68 | 2 | 13 | 5 | 0 | 0 | 0 |
| 11/1/2008 | 27.89 | 10.24 | 1 | 29 | 15 | 0 | 0 | 0 |
| 11/2/2008 | 28.60 | 10.33 | 0 | 46 | 8 | 0 | 0 | 0 |
| 11/3/2008 | 32.56 | 10.00 | 0 | 39 | 15 | 0 | 0 | 0 |
| 11/4/2008 | 54.65 | 9.54 | 1 | 369 | 53 | 1 | 0 | 0 |
| 11/5/2008 | 72.49 | 8.62 | 3 | 831 | 33 | 0 | 0 | 0 |
| 11/6/2008 | 105.91 | 8.27 | 2 | 267 | 18 | 0 | 0 | 0 |
| 11/7/2008 | 101.09 | 8.81 | 2 | 413 | 26 | 2 | 0 | 0 |
| 11/8/2008 | 69.66 | 9.04 | 0 | 509 | 37 | 0 | 0 | 0 |
| 11/9/2008 | 88.63 | 8.81 | 0 | 221 | 15 | 0 | 0 | 0 |
| 11/10/2008 | 75.32 | 8.69 | 2 | 178 | 16 | 1 | 0 | 0 |
| 11/11/2008 | 67.11 | 8.76 | 0 | 70 | 18 | 1 | 0 | 0 |
| 11/12/2008 | 261.36 | 9.29 | 0 | 122 | 9 | 1 | 2 | 1 |
| 11/13/2008 | 204.45 | 9.94 | 1 | 66 | 8 | 2 | 0 | 2 |
| 11/14/2008 | 172.17 | 9.61 | 0 | 41 | 20 | 0 | 0 | 1 |
| 11/15/2008 | 105.62 | 8.54 | 0 | 30 | 12 | 0 | 0 | 0 |
| 11/16/2008 | 77.59 | 8.28 | 0 | 12 | 6 | 0 | 0 | 0 |
| 11/17/2008 | 62.58 | 8.02 | 0 | 10 | 7 | 0 | 0 | 0 |
| 11/18/2008 | 53.52 | 8.05 | 0 | 14 | 15 | 0 | 0 | 0 |
| 11/19/2008 | 48.70 | 8.34 | 0 | 11 | 15 | 0 | 0 | 0 |
| 11/20/2008 | 46.72 | 8.42 | 0 | 2 | 7 | 0 | 0 | 0 |
| 11/21/2008 | 52.39 | 8.03 | 0 | 6 | 6 | 0 | 0 | 0 |
| 11/22/2008 | 52.95 | 7.89 | 0 | 3 | 9 | 0 | 0 | 0 |
| 11/23/2008 | 53.24 | 7.24 | 0 | 0 | 1 | 0 | 0 | 0 |
| 11/24/2008 | 50.12 | 6.52 | 0 | 0 | 0 | 0 | 0 | 0 |
| 11/25/2008 | 46.72 | 6.25 | 0 | 0 | 1 | 0 | 0 | 0 |
| 11/26/2008 | 44.46 | 6.42 | 0 | 0 | 1 | 0 | 0 | 0 |
| 11/27/2008 | 41.91 | 6.96 | 0 | 1 | 2 | 0 | 0 | 0 |
| 11/28/2008 | 39.08 | 7.36 | 0 | 0 | 2 | 0 | 0 | 0 |
| 11/29/2008 | 36.53 | 7.22 | 0 | 1 | 2 | 0 | 0 | 0 |
| 11/30/2008 | 35.68 | 7.27 | 0 | 0 | 7 | 0 | 0 | 0 |
| 12/1/2008 | 35.11 | 7.38 | 0 | 0 | 1 | 0 | 0 | 0 |
| 12/2/2008 | 35.11 | 7.38 | 0 | 0 | 4 | 0 | 0 | 0 |
| 12/3/2008 | 38.79 | 6.93 | 0 | 0 | 2 | 0 | 0 | 0 |
| 12/4/2008 | 37.10 | 7.10 | 0 | 0 | 6 | 0 | 0 | 0 |
| 12/5/2008 | 35.96 | 6.59 | 0 | 0 | 3 | 0 | 0 | 0 |
| 12/6/2008 | 35.11 | 5.32 | 0 | 0 | 0 | 0 | 0 | 0 |
| 12/7/2008 | 34.26 | 4.49 | 0 | 0 | 0 | 0 | 0 | 0 |

| Date | Discharge (cms) | Temp (C°) | Chinook | Coho | Steelhead | Cutthroat | Lamprey | Sucker |
|---|---|---|---|---|---|---|---|---|
| 12/8/2008 | 33.98 | 4.65 | 0 | 0 | 1 | 0 | 0 | 0 |
| 12/9/2008 | 34.26 | 4.96 | 0 | 0 | 0 | 0 | 0 | 0 |
| 12/10/2008 | 33.41 | 4.90 | 0 | 0 | 0 | 0 | 0 | 0 |
| 12/11/2008 | 32.85 | 4.64 | 0 | 0 | 0 | 0 | 0 | 0 |
| 12/12/2008 | 32.85 | 4.65 | 0 | 0 | 0 | 0 | 0 | 0 |
| 12/13/2008 | 43.04 | 4.24 | 0 | 0 | 0 | 0 | 0 | 0 |
| 12/14/2008 | 48.99 | 4.08 | 0 | 0 | 1 | 0 | 0 | 0 |
| 12/15/2008 | 45.59 | 3.69 | 0 | 0 | 0 | 0 | 0 | 0 |
| 12/16/2008 | 41.63 | 2.55 | 0 | 0 | 0 | 0 | 0 | 0 |
| 12/17/2008 | 37.94 | 1.84 | 0 | 0 | 0 | 0 | 0 | 0 |
| 12/18/2008 | 41.34 | 1.75 | 0 | 0 | 0 | 0 | 0 | 0 |
| 12/19/2008 | 48.42 | 1.38 | 0 | 0 | 0 | 0 | 0 | 0 |
| 12/20/2008 | 43.04 | 2.06 | 0 | 0 | 0 | 0 | 0 | 0 |
| 12/21/2008 | 64.00 | 3.30 | 0 | 1 | 3 | 0 | 0 | 0 |
| 12/22/2008 | 188.31 | 3.95 | 0 | 5 | 0 | 0 | 0 | 0 |
| 12/23/2008 | 135.92 | 4.45 | 0 | 0 | 24 | 0 | 0 | 0 |
| 12/24/2008 | 101.37 | 4.53 | 0 | 1 | 43 | 0 | 0 | 0 |
| 12/25/2008 | 134.79 | 4.47 | 0 | 2 | 16 | 0 | 0 | 0 |
| 12/26/2008 | 100.52 | 4.33 | 0 | 4 | 19 | 0 | 0 | 0 |
| 12/27/2008 | 106.19 | 4.85 | 0 | 2 | 60 | 0 | 0 | 0 |
| 12/28/2008 | 727.74 | 5.56 | 0 | 0 | 5 | 0 | 0 | 0 |
| 12/29/2008 | 1299.74 | 7.05 | 0 | 0 | 0 | 0 | 0 | 0 |
| 12/30/2008 | 685.27 | 6.77 | 0 | 0 | 0 | 0 | 0 | 0 |
| 12/31/2008 | 342.63 | 6.69 | 0 | 0 | 8 | 0 | 0 | 0 |
| 1/1/2009 | 308.65 | 6.81 | 0 | 0 | 42 | 0 | 0 | 0 |
| 1/2/2009 | 690.93 | 7.19 | 0 | 0 | 0 | 0 | 0 | 0 |
| 1/3/2009 | 447.41 | 6.29 | 0 | 0 | 2 | 0 | 0 | 0 |
| 1/4/2009 | 263.91 | 5.01 | 0 | 0 | 1 | 0 | 0 | 0 |
| 1/5/2009 | 274.67 | 5.14 | 0 | 0 | 7 | 0 | 0 | 0 |
| 1/6/2009 | 433.25 | 6.64 | 0 | 0 | 73 | 0 | 0 | 0 |
| 1/7/2009 | 475.72 | 7.22 | 0 | 0 | 121 | 0 | 0 | 0 |
| 1/8/2009 | 467.23 | 7.35 | 0 | 0 | 132 | 0 | 0 | 0 |
| 1/9/2009 | 438.91 | 6.77 | 0 | 0 | 13 | 0 | 0 | 0 |
| 1/10/2009 | 283.17 | 6.09 | 0 | 0 | 5 | 0 | 0 | 0 |
| 1/11/2009 | 207.56 | 5.95 | 0 | 0 | 79 | 0 | 0 | 0 |
| 1/12/2009 | 177.55 | 5.95 | 0 | 0 | 54 | 0 | 0 | 0 |
| 1/13/2009 | 184.91 | 5.94 | 0 | 0 | 55 | 0 | 0 | 0 |
| 1/14/2009 | 180.10 | 5.64 | 0 | 0 | 37 | 0 | 0 | 0 |
| 1/15/2009 | 179.25 | 5.28 | 0 | 0 | 16 | 0 | 0 | 0 |
| 1/16/2009 | 176.13 | 5.19 | 0 | 0 | 17 | 0 | 0 | 0 |
| 1/17/2009 | 177.26 | 4.93 | 0 | 0 | 8 | 0 | 0 | 0 |
| 1/18/2009 | 169.90 | 4.94 | 0 | 0 | 8 | 0 | 0 | 0 |
| 1/19/2009 | 157.72 | 4.86 | 0 | 0 | 15 | 0 | 0 | 0 |
| 1/20/2009 | 143.85 | 4.81 | 0 | 0 | 9 | 0 | 0 | 0 |
| 1/21/2009 | 129.12 | 4.75 | 0 | 0 | 10 | 0 | 0 | 0 |

| Date | Discharge (cms) | Temp (C°) | Chinook | Coho | Steelhead | Cutthroat | Lamprey | Sucker |
|---|---|---|---|---|---|---|---|---|
| 1/22/2009 | 118.08 | 5.66 | 0 | 0 | 129 | 1 | 0 | 0 |
| 1/23/2009 | 114.97 | 6.29 | 0 | 0 | 191 | 0 | 0 | 0 |
| 1/24/2009 | 111.85 | 6.39 | 0 | 0 | 153 | 0 | 0 | 0 |
| 1/25/2009 | 113.27 | 6.45 | 0 | 0 | 88 | 0 | 0 | 0 |
| 1/26/2009 | 106.19 | 5.58 | 0 | 0 | 5 | 0 | 0 | 0 |
| 1/27/2009 | 94.30 | 4.82 | 0 | 0 | 2 | 0 | 0 | 0 |
| 1/28/2009 | 92.88 | 4.74 | 0 | 0 | 1 | 0 | 0 | 0 |
| 1/29/2009 | 92.60 | 4.65 | 0 | 0 | 1 | 0 | 0 | 0 |
| 1/30/2009 | 87.50 | 5.24 | 0 | 0 | 4 | 0 | 0 | 0 |
| 1/31/2009 | 85.23 | 4.94 | 0 | 0 | 0 | 0 | 0 | 0 |
| 2/1/2009 | 82.69 | 4.53 | 0 | 0 | 4 | 0 | 0 | 0 |
| 2/2/2009 | 78.44 | 4.52 | 0 | 0 | 0 | 0 | 0 | 0 |
| 2/3/2009 | 73.91 | 4.54 | 0 | 0 | 1 | 0 | 0 | 0 |
| 2/4/2009 | 74.19 | 4.92 | 0 | 0 | 3 | 0 | 0 | 0 |
| 2/5/2009 | 76.46 | 5.41 | 0 | 0 | 10 | 0 | 0 | 0 |
| 2/6/2009 | 79.57 | 6.41 | 0 | 0 | 116 | 0 | 0 | 0 |
| 2/7/2009 | 87.50 | 6.55 | 0 | 0 | 193 | 0 | 0 | 0 |
| 2/8/2009 | 84.95 | 6.49 | 0 | 0 | 27 | 0 | 0 | 0 |
| 2/9/2009 | 80.70 | 5.59 | 0 | 0 | 0 | 0 | 0 | 0 |
| 2/10/2009 | 74.47 | 4.74 | 0 | 0 | 1 | 0 | 0 | 0 |
| 2/11/2009 | 72.21 | 4.47 | 0 | 0 | 0 | 0 | 0 | 0 |
| 2/12/2009 | 68.81 | 4.75 | 0 | 0 | 0 | 0 | 0 | 0 |
| 2/13/2009 | 68.81 | 4.77 | 0 | 0 | 0 | 0 | 0 | 0 |
| 2/14/2009 | 67.96 | 5.28 | 0 | 0 | 6 | 0 | 0 | 0 |
| 2/15/2009 | 66.26 | 5.59 | 0 | 0 | 0 | 0 | 0 | 0 |
| 2/16/2009 | 65.13 | 6.07 | 0 | 0 | 23 | 0 | 0 | 0 |
| 2/17/2009 | 69.09 | 6.61 | 0 | 0 | 134 | 0 | 0 | 0 |
| 2/18/2009 | 69.94 | 6.76 | 0 | 0 | 49 | 0 | 0 | 0 |
| 2/19/2009 | 66.83 | 6.09 | 0 | 0 | 7 | 0 | 0 | 0 |
| 2/20/2009 | 63.43 | 5.90 | 0 | 0 | 3 | 0 | 0 | 0 |
| 2/21/2009 | 61.16 | 5.91 | 0 | 0 | 3 | 0 | 0 | 0 |
| 2/22/2009 | 63.71 | 6.22 | 0 | 0 | 43 | 0 | 0 | 0 |
| 2/23/2009 | 93.16 | 6.93 | 0 | 0 | 728 | 0 | 0 | 0 |
| 2/24/2009 | 138.19 | 7.20 | 0 | 0 | 331 | 0 | 0 | 0 |
| 2/25/2009 | 133.66 | 7.22 | 0 | 0 | 496 | 0 | 0 | 0 |
| 2/26/2009 | 242.68 | 6.61 | 0 | 0 | 18 | 0 | 0 | 0 |
| 2/27/2009 | 198.78 | 6.46 | 0 | 0 | 20 | 0 | 0 | 0 |
| 2/28/2009 | 147.81 | 5.88 | 0 | 0 | 6 | 0 | 0 | 0 |
| 3/1/2009 | 128.28 | 6.84 | 0 | 0 | 101 | 0 | 0 | 0 |
| 3/2/2009 | 137.90 | 7.83 | 0 | 0 | 374 | 0 | 0 | 0 |
| 3/3/2009 | 157.16 | 7.67 | 0 | 0 | 236 | 0 | 0 | 0 |
| 3/4/2009 | 148.95 | 6.78 | 0 | 0 | 24 | 0 | 0 | 0 |
| 3/5/2009 | 135.35 | 6.36 | 0 | 0 | 17 | 0 | 0 | 0 |
| 3/6/2009 | 136.77 | 6.78 | 0 | 0 | 38 | 0 | 0 | 0 |
| 3/7/2009 | 130.26 | 6.15 | 0 | 0 | 10 | 0 | 0 | 0 |

| Date | Discharge (cms) | Temp (C°) | Chinook | Coho | Steelhead | Cutthroat | Lamprey | Sucker |
|---|---|---|---|---|---|---|---|---|
| 3/8/2009 | 119.78 | 6.31 | 0 | 0 | 10 | 0 | 0 | 0 |
| 3/9/2009 | 111.57 | 5.61 | 0 | 0 | 0 | 0 | 0 | 0 |
| 3/10/2009 | 101.66 | 5.58 | 0 | 0 | 3 | 0 | 0 | 0 |
| 3/11/2009 | 93.16 | 5.21 | 0 | 0 | 0 | 0 | 0 | 0 |
| 3/12/2009 | 87.50 | 6.15 | 0 | 0 | 6 | 0 | 0 | 0 |
| 3/13/2009 | 90.33 | 7.10 | 0 | 0 | 26 | 0 | 0 | 0 |
| 3/14/2009 | 104.21 | 6.65 | 0 | 0 | 154 | 0 | 0 | 0 |
| 3/15/2009 | 188.02 | 6.53 | 0 | 0 | 107 | 0 | 0 | 0 |
| 3/16/2009 | 529.53 | 6.74 | 0 | 0 | 13 | 0 | 0 | 0 |
| 3/17/2009 | 342.63 | 7.22 | 0 | 0 | 22 | 0 | 0 | 0 |
| 3/18/2009 | 247.21 | 8.20 | 0 | 0 | 244 | 0 | 0 | 0 |
| 3/19/2009 | 200.48 | 7.92 | 0 | 0 | 260 | 1 | 0 | 0 |
| 3/20/2009 | 180.66 | 8.62 | 0 | 0 | 301 | 2 | 0 | 0 |
| 3/21/2009 | 176.13 | 8.35 | 0 | 0 | 132 | 0 | 0 | 0 |
| 3/22/2009 | 182.08 | 7.97 | 0 | 0 | 41 | 0 | 0 | 0 |
| 3/23/2009 | 163.95 | 6.66 | 0 | 0 | 5 | 0 | 0 | 0 |
| 3/24/2009 | 145.55 | 7.02 | 0 | 0 | 25 | 0 | 0 | 0 |
| 3/25/2009 | 146.96 | 7.43 | 0 | 0 | 40 | 0 | 0 | 0 |
| 3/26/2009 | 198.50 | 7.99 | 0 | 0 | 135 | 0 | 0 | 0 |
| 3/27/2009 | 198.50 | 8.75 | 0 | 0 | 77 | 0 | 0 | 0 |
| 3/28/2009 | 171.03 | 8.70 | 0 | 0 | 132 | 0 | 0 | 0 |
| 3/29/2009 | 186.89 | 8.17 | 0 | 0 | 43 | 0 | 0 | 0 |
| 3/30/2009 | 175.28 | 6.79 | 0 | 0 | 2 | 0 | 0 | 0 |
| 3/31/2009 | 152.06 | 6.81 | 0 | 0 | 1 | 0 | 0 | 0 |
| 4/1/2009 | 137.62 | 6.76 | 0 | 0 | 9 | 0 | 0 | 0 |
| 4/2/2009 | 148.10 | 7.53 | 1 | 0 | 33 | 0 | 0 | 0 |
| 4/3/2009 | 239.84 | 7.28 | 0 | 0 | 33 | 0 | 0 | 0 |
| 4/4/2009 | 206.43 | 7.54 | 0 | 0 | 13 | 0 | 0 | 0 |
| 4/5/2009 | 175.28 | 8.42 | 0 | 0 | 53 | 0 | 0 | 0 |
| 4/6/2009 | 154.89 | 9.36 | 0 | 0 | 166 | 0 | 0 | 0 |
| 4/7/2009 | 148.38 | 8.87 | 0 | 0 | 175 | 0 | 0 | 0 |
| 4/8/2009 | 144.42 | 8.43 | 1 | 0 | 78 | 0 | 0 | 0 |
| 4/9/2009 | 143.28 | 8.63 | 0 | 0 | 103 | 0 | 0 | 0 |
| 4/10/2009 | 160.56 | 8.71 | 1 | 0 | 44 | 0 | 0 | 0 |
| 4/11/2009 | 145.27 | 9.02 | 0 | 0 | 52 | 0 | 0 | 0 |
| 4/12/2009 | 128.56 | 8.89 | 0 | 0 | 60 | 0 | 0 | 0 |
| 4/13/2009 | 135.92 | 9.22 | 1 | 0 | 95 | 0 | 0 | 0 |
| 4/14/2009 | 148.66 | 8.03 | 0 | 0 | 4 | 1 | 0 | 0 |
| 4/15/2009 | 141.02 | 7.39 | 0 | 0 | 4 | 0 | 0 | 0 |
| 4/16/2009 | 129.12 | 7.40 | 0 | 0 | 0 | 0 | 0 | 0 |
| 4/17/2009 | 119.21 | 8.59 | 0 | 0 | 16 | 0 | 0 | 0 |
| 4/18/2009 | 114.97 | 10.01 | 1 | 0 | 85 | 0 | 0 | 77 |
| 4/19/2009 | 112.42 | 11.11 | 3 | 0 | 132 | 1 | 0 | 583 |
| 4/20/2009 | 116.67 | 12.40 | 14 | 0 | 92 | 0 | 0 | 836 |
| 4/21/2009 | 134.22 | 13.20 | 12 | 0 | 51 | 0 | 0 | 501 |

| Date | Discharge (cms) | Temp (C°) | Chinook | Coho | Steelhead | Cutthroat | Lamprey | Sucker |
|---|---|---|---|---|---|---|---|---|
| 4/22/2009 | 145.83 | 12.81 | 17 | 0 | 29 | 1 | 0 | 426 |
| 4/23/2009 | 138.47 | 11.41 | 1 | 0 | 4 | 0 | 0 | 6 |
| 4/24/2009 | 122.05 | 9.96 | 1 | 0 | 3 | 0 | 0 | 0 |
| 4/25/2009 | 106.47 | 8.84 | 0 | 0 | 1 | 0 | 0 | 0 |
| 4/26/2009 | 94.30 | 8.80 | 1 | 0 | 3 | 0 | 0 | 0 |
| 4/27/2009 | 86.65 | 9.72 | 9 | 0 | 11 | 0 | 0 | 0 |
| 4/28/2009 | 84.95 | 9.48 | 16 | 0 | 11 | 0 | 0 | 0 |
| 4/29/2009 | 85.80 | 9.41 | 7 | 0 | 1 | 0 | 0 | 0 |
| 4/30/2009 | 79.00 | 10.12 | 4 | 0 | 4 | 1 | 0 | 0 |
| 5/1/2009 | 72.77 | 11.07 | 35 | 0 | 26 | 0 | 0 | 51 |
| 5/2/2009 | 75.04 | 10.93 | 57 | 0 | 9 | 0 | 0 | 222 |
| 5/3/2009 | 96.28 | 11.27 | 47 | 0 | 12 | 0 | 0 | 166 |
| 5/4/2009 | 111.29 | 10.09 | 0 | 0 | 0 | 0 | 0 | 11 |
| 5/5/2009 | 219.17 | 9.39 | 0 | 0 | 0 | 0 | 0 | 0 |
| 5/6/2009 | 244.09 | 9.54 | 2 | 0 | 5 | 0 | 0 | 2 |
| 5/7/2009 | 229.08 | 10.07 | 10 | 0 | 6 | 0 | 0 | 0 |
| 5/8/2009 | 192.55 | 9.81 | 7 | 0 | 2 | 1 | 0 | 0 |
| 5/9/2009 | 158.57 | 11.06 | 52 | 0 | 8 | 1 | 0 | 0 |
| 5/10/2009 | 137.62 | 11.83 | 30 | 0 | 3 | 0 | 0 | 14 |
| 5/11/2009 | 125.44 | 11.15 | 3 | 0 | 1 | 0 | 0 | 13 |
| 5/12/2009 | 116.95 | 11.49 | 58 | 0 | 8 | 0 | 0 | 49 |
| 5/13/2009 | 109.87 | 10.11 | 1 | 0 | 0 | 0 | 0 | 131 |
| 5/14/2009 | 110.72 | 10.51 | 7 | 0 | 4 | 0 | 0 | 1 |
| 5/15/2009 | 128.28 | 13.05 | 162 | 0 | 12 | 1 | 0 | 30 |
| 5/16/2009 | 117.80 | 13.57 | 110 | 0 | 7 | 0 | 0 | 321 |
| 5/17/2009 | 114.97 | 12.61 | 57 | 0 | 5 | 0 | 0 | 103 |
| 5/18/2009 | 119.78 | 13.91 | 65 | 0 | 5 | 1 | 0 | 887 |
| 5/19/2009 | 124.03 | 14.22 | 56 | 0 | 0 | 2 | 0 | 0 |
| 5/20/2009 | 113.27 | 12.21 | 34 | 0 | 3 | 0 | 0 | 159 |
| 5/21/2009 | 99.11 | 13.47 | 78 | 0 | 1 | 2 | 0 | 53 |
| 5/22/2009 | 92.60 | 13.15 | 291 | 0 | 4 | 0 | 0 | 67 |
| 5/23/2009 | 91.75 | 13.19 | 163 | 0 | 9 | 0 | 0 | 55 |
| 5/24/2009 | 88.63 | 13.41 | 197 | 0 | 11 | 0 | 0 | 64 |
| 5/25/2009 | 85.80 | 13.29 | 37 | 0 | 4 | 0 | 0 | 21 |
| 5/26/2009 | 82.40 | 15.03 | 0 | 0 | 0 | 0 | 0 | 0 |
| 5/27/2009 | 79.57 | 15.21 | 223 | 0 | 20 | 1 | 0 | 514 |
| 5/28/2009 | 76.46 | 15.49 | 241 | 0 | 11 | 2 | 0 | 258 |
| 5/29/2009 | 75.32 | 16.86 | 133 | 0 | 6 | 2 | 0 | 269 |
| 5/30/2009 | 77.59 | 17.42 | 72 | 0 | 5 | 0 | 0 | 355 |
| 5/31/2009 | 76.17 | 17.60 | 137 | 0 | 9 | 3 | 0 | 372 |
| 6/1/2009 | 74.47 | 17.42 | 27 | 0 | 2 | 2 | 0 | 41 |
| 6/2/2009 | 80.42 | 17.07 | 22 | 0 | 0 | 1 | 3 | 249 |
| 6/3/2009 | 82.12 | 16.21 | 198 | 0 | 24 | 0 | 0 | 289 |
| 6/4/2009 | 82.69 | 15.68 | 93 | 0 | 15 | 0 | 0 | 100 |
| 6/5/2009 | 85.52 | 14.85 | 64 | 0 | 6 | 0 | 1 | 11 |

| Date | Discharge (cms) | Temp (C°) | Chinook | Coho | Steelhead | Cutthroat | Lamprey | Sucker |
|---|---|---|---|---|---|---|---|---|
| 6/6/2009 | 77.02 | 15.09 | 86 | 0 | 16 | 0 | 0 | 121 |
| 6/7/2009 | 69.09 | 15.72 | 61 | 0 | 15 | 0 | 0 | 29 |
| 6/8/2009 | 64.85 | 16.20 | 14 | 0 | 6 | 0 | 0 | 1 |
| 6/9/2009 | 61.45 | 17.25 | 16 | 0 | 0 | 0 | 1 | 381 |
| 6/10/2009 | 58.90 | 15.99 | 87 | 0 | 39 | 0 | 0 | 15 |
| 6/11/2009 | 57.20 | 16.51 | 65 | 0 | 36 | 0 | 0 | 75 |
| 6/12/2009 | 59.75 | 16.91 | 66 | 0 | 30 | 0 | 0 | 323 |
| 6/13/2009 | 62.30 | 16.23 | 59 | 0 | 35 | 0 | 0 | 53 |
| 6/14/2009 | 62.01 | 16.57 | 52 | 0 | 33 | 0 | 1 | 138 |
| 6/15/2009 | 60.03 | 17.77 | 24 | 0 | 5 | 0 | 2 | 980 |
| 6/16/2009 | 57.20 | 17.96 | 53 | 0 | 36 | 1 | 9 | 167 |
| 6/17/2009 | 53.52 | 17.66 | 39 | 0 | 25 | 0 | 0 | 44 |
| 6/18/2009 | 51.54 | 17.76 | 44 | 0 | 47 | 1 | 2 | 37 |
| 6/19/2009 | 51.25 | 18.82 | 48 | 0 | 57 | 1 | 5 | 927 |
| 6/20/2009 | 50.12 | 17.56 | 57 | 0 | 55 | 2 | 0 | 18 |
| 6/21/2009 | 49.27 | 16.35 | 39 | 0 | 36 | 0 | 0 | 4 |
| 6/22/2009 | 48.99 | 15.69 | 12 | 0 | 25 | 1 | 2 | 0 |
| 6/23/2009 | 48.42 | 17.01 | 0 | 0 | 0 | 0 | 0 | 0 |
| 6/24/2009 | 45.59 | 17.87 | 22 | 0 | 9 | 2 | 3 | 193 |
| 6/25/2009 | 41.34 | 19.10 | 47 | 0 | 100 | 2 | 0 | 32 |
| 6/26/2009 | 39.08 | 18.77 | 44 | 0 | 76 | 0 | 2 | 11 |
| 6/27/2009 | 38.51 | 19.27 | 40 | 0 | 52 | 2 | 0 | 18 |
| 6/28/2009 | 37.66 | 19.57 | 45 | 0 | 92 | 2 | 0 | 2 |
| 6/29/2009 | 36.81 | 19.45 | 16 | 0 | 25 | 3 | 0 | 0 |
| 6/30/2009 | 36.25 | 19.47 | 11 | 0 | 2 | 0 | 6 | 20 |
| 7/1/2009 | 35.96 | 20.03 | 42 | 0 | 128 | 1 | 0 | 14 |
| 7/2/2009 | 35.68 | 20.20 | 26 | 0 | 74 | 1 | 8 | 17 |
| 7/3/2009 | 35.40 | 20.97 | 24 | 0 | 61 | 0 | 1 | 10 |
| 7/4/2009 | 35.40 | 21.34 | 27 | 0 | 52 | 0 | 11 | 9 |
| 7/5/2009 | 35.11 | 21.76 | 27 | 0 | 65 | 0 | 4 | 2 |
| 7/6/2009 | 34.55 | 21.19 | 7 | 0 | 44 | 2 | 1 | 0 |
| 7/7/2009 | 33.98 | 20.27 | 2 | 0 | 11 | 0 | 7 | 0 |
| 7/8/2009 | 33.41 | 19.94 | 0 | 0 | 1 | 0 | 0 | 1 |
| 7/9/2009 | 33.13 | 19.81 | 0 | 0 | 0 | 0 | 0 | 0 |
| 7/10/2009 | 33.13 | 20.01 | 12 | 0 | 45 | 0 | 3 | 0 |
| 7/11/2009 | 33.13 | 19.80 | 13 | 0 | 47 | 0 | 0 | 0 |
| 7/12/2009 | 33.41 | 19.10 | 25 | 0 | 100 | 1 | 0 | 2 |
| 7/13/2009 | 33.13 | 18.49 | 6 | 0 | 20 | 0 | 1 | 0 |
| 7/14/2009 | 33.41 | 19.38 | 0 | 0 | 0 | 2 | 2 | 0 |
| 7/15/2009 | 32.28 | 19.28 | 16 | 0 | 44 | 3 | 0 | 1 |
| 7/16/2009 | 31.43 | 20.72 | 22 | 0 | 212 | 2 | 0 | 1 |
| 7/17/2009 | 30.87 | 21.46 | 38 | 0 | 189 | 4 | 12 | 2 |
| 7/18/2009 | 30.30 | 21.51 | 39 | 0 | 127 | 2 | 1 | 3 |
| 7/19/2009 | 29.73 | 21.77 | 31 | 0 | 61 | 1 | 7 | 0 |
| 7/20/2009 | 29.45 | 21.68 | 22 | 0 | 52 | 2 | 0 | 1 |

| Date | Discharge (cms) | Temp (C°) | Chinook | Coho | Steelhead | Cutthroat | Lamprey | Sucker |
|---|---|---|---|---|---|---|---|---|
| 7/21/2009 | 29.17 | 22.21 | 10 | 0 | 36 | 2 | 8 | 1 |
| 7/22/2009 | 28.88 | 22.08 | 3 | 0 | 22 | 1 | 2 | 1 |
| 7/23/2009 | 29.17 | 21.47 | 5 | 0 | 10 | 1 | 7 | 0 |
| 7/24/2009 | 28.60 | 21.61 | 3 | 0 | 7 | 0 | 0 | 2 |
| 7/25/2009 | 28.60 | 22.48 | 1 | 0 | 1 | 0 | 0 | 0 |
| 7/26/2009 | 28.60 | 22.98 | 3 | 0 | 6 | 1 | 2 | 0 |
| 7/27/2009 | 27.98 | 23.57 | 2 | 0 | 3 | 2 | 8 | 0 |
| 7/28/2009 | 27.47 | 24.41 | 5 | 0 | 4 | 1 | 2 | 1 |
| 7/29/2009 | 26.90 | 24.93 | 4 | 0 | 5 | 2 | 2 | 2 |
| 7/30/2009 | 26.62 | 24.62 | 1 | 0 | 4 | 7 | 7 | 0 |
| 7/31/2009 | 25.91 | 24.05 | 1 | 0 | 1 | 1 | 5 | 0 |
| 8/1/2009 | 25.26 | 23.48 | 0 | 0 | 0 | 2 | 0 | 0 |
| 8/2/2009 | 24.61 | 23.44 | 0 | 0 | 0 | 0 | 0 | 0 |
| 8/3/2009 | 25.40 | 23.09 | 0 | 0 | 0 | 0 | 0 | 0 |
| 8/4/2009 | 26.19 | 23.18 | 0 | 0 | 0 | 0 | 0 | 0 |
| 8/5/2009 | 25.15 | 21.91 | 0 | 0 | 1 | 0 | 0 | 0 |
| 8/6/2009 | 24.86 | 20.03 | 0 | 0 | 1 | 0 | 0 | 0 |
| 8/7/2009 | 25.51 | 18.56 | 0 | 0 | 0 | 0 | 1 | 0 |
| 8/8/2009 | 27.01 | 18.39 | 23 | 0 | 0 | 0 | 0 | 0 |
| 8/9/2009 | 27.13 | 18.65 | 50 | 0 | 5 | 0 | 0 | 0 |
| 8/10/2009 | 25.99 | 19.42 | 75 | 0 | 33 | 0 | 1 | 0 |
| 8/11/2009 | 25.57 | 20.25 | 85 | 0 | 81 | 1 | 0 | 0 |
| 8/12/2009 | 24.83 | 20.94 | 68 | 0 | 98 | 4 | 0 | 0 |
| 8/13/2009 | 24.58 | 20.73 | 60 | 0 | 60 | 2 | 0 | 0 |
| 8/14/2009 | 24.32 | 19.40 | 25 | 0 | 40 | 0 | 1 | 0 |
| 8/15/2009 | 24.24 | 19.17 | 26 | 0 | 36 | 1 | 0 | 0 |
| 8/16/2009 | 23.70 | 19.16 | 50 | 0 | 40 | 0 | 0 | 0 |
| 8/17/2009 | 23.19 | 19.31 | 45 | 0 | 34 | 1 | 0 | 0 |
| 8/18/2009 | 24.44 | 20.07 | 66 | 0 | 32 | 1 | 0 | 0 |
| 8/19/2009 | 25.06 | 20.46 | 55 | 0 | 40 | 1 | 0 | 0 |
| 8/20/2009 | 25.06 | 20.94 | 48 | 0 | 18 | 0 | 0 | 0 |
| 8/21/2009 | 24.78 | 20.79 | 49 | 0 | 16 | 0 | 0 | 0 |
| 8/22/2009 | 24.32 | 20.10 | 27 | 0 | 16 | 0 | 0 | 0 |
| 8/23/2009 | 23.84 | 19.83 | 24 | 0 | 10 | 1 | 0 | 0 |
| 8/24/2009 | 23.93 | 19.54 | 13 | 0 | 9 | 0 | 0 | 0 |
| 8/25/2009 | 23.87 | 19.49 | 9 | 0 | 8 | 1 | 0 | 1 |
| 8/26/2009 | 23.62 | 19.44 | 18 | 1 | 10 | 0 | 0 | 0 |
| 8/27/2009 | 23.22 | 20.08 | 11 | 0 | 20 | 0 | 0 | 0 |
| 8/28/2009 | 23.05 | 19.95 | 16 | 0 | 12 | 0 | 0 | 0 |
| 8/29/2009 | 23.16 | 18.46 | 8 | 0 | 10 | 0 | 0 | 0 |
| 8/30/2009 | 23.47 | 19.04 | 15 | 0 | 19 | 0 | 0 | 0 |
| 8/31/2009 | 28.32 | 19.25 | 12 | 1 | 6 | 0 | 0 | 0 |
| 9/1/2009 | 24.24 | | 0 | 0 | 0 | 0 | 0 | 0 |
| 9/2/2009 | 24.35 | | 0 | 0 | 0 | 0 | 0 | 0 |
| 9/3/2009 | 24.27 | | 0 | 0 | 0 | 0 | 0 | 0 |

| Date | Discharge (cms) | Temp (C°) | Chinook | Coho | Steelhead | Cutthroat | Lamprey | Sucker |
|---|---|---|---|---|---|---|---|---|
| 9/4/2009 | 23.93 | | 0 | 0 | 0 | 0 | 0 | 0 |
| 9/5/2009 | 24.92 | | 0 | 0 | 0 | 0 | 0 | 0 |
| 9/6/2009 | 25.12 | | 0 | 0 | 0 | 0 | 0 | 0 |
| 9/7/2009 | 25.00 | | 0 | 0 | 0 | 0 | 0 | 0 |
| 9/8/2009 | 24.66 | | 0 | 0 | 0 | 0 | 0 | 0 |
| 9/9/2009 | 23.64 | | 0 | 0 | 0 | 0 | 0 | 0 |
| 9/10/2009 | 22.51 | | 0 | 0 | 0 | 0 | 0 | 0 |
| 9/11/2009 | 21.80 | | 0 | 0 | 0 | 0 | 0 | 0 |
| 9/12/2009 | 21.95 | | 0 | 0 | 0 | 0 | 0 | 0 |
| 9/13/2009 | 22.85 | | 0 | 0 | 0 | 0 | 0 | 0 |
| 9/14/2009 | 22.71 | 18.21 | 35 | 17 | 39 | 1 | 0 | 0 |
| 9/15/2009 | 22.63 | 18.24 | 6 | 24 | 25 | 0 | 0 | 0 |
| 9/16/2009 | 22.60 | 18.47 | 6 | 14 | 22 | 0 | 0 | 0 |
| 9/17/2009 | 23.11 | 17.94 | 6 | 12 | 12 | 0 | 0 | 0 |
| 9/18/2009 | 23.67 | 17.62 | 3 | 8 | 7 | 0 | 0 | 1 |
| 9/19/2009 | 23.93 | 17.36 | 3 | 17 | 10 | 0 | 0 | 0 |
| 9/20/2009 | 23.93 | 16.59 | 3 | 16 | 4 | 0 | 0 | 0 |
| 9/21/2009 | 23.47 | 16.55 | 2 | 22 | 6 | 0 | 0 | 0 |
| 9/22/2009 | 23.02 | 16.76 | 5 | 33 | 8 | 0 | 0 | 0 |
| 9/23/2009 | 22.48 | 16.84 | 14 | 60 | 11 | 0 | 0 | 0 |
| 9/24/2009 | 22.26 | 16.29 | 4 | 83 | 6 | 0 | 0 | 0 |
| 9/25/2009 | 22.34 | 15.77 | 2 | 110 | 7 | 0 | 0 | 0 |
| 9/26/2009 | 22.34 | 15.77 | 2 | 55 | 4 | 0 | 0 | 0 |
| 9/27/2009 | 22.40 | 15.95 | 6 | 86 | 9 | 0 | 0 | 1 |
| 9/28/2009 | 22.37 | 15.40 | 4 | 105 | 4 | 0 | 0 | 1 |
| 9/29/2009 | 22.96 | 14.62 | 1 | 123 | 12 | 0 | 0 | 0 |
| 9/30/2009 | 23.70 | 13.52 | 2 | 115 | 2 | 0 | 0 | 0 |
| 10/1/2009 | 24.75 | 13.35 | 5 | 34 | 3 | 0 | 0 | 0 |
| 10/2/2009 | 24.69 | 13.03 | 6 | 25 | 4 | 0 | 0 | 0 |
| 10/3/2009 | 24.35 | 12.25 | 6 | 18 | 2 | 0 | 0 | 0 |
| 10/4/2009 | 24.38 | 10.88 | 5 | 5 | 1 | 0 | 0 | 0 |
| 10/5/2009 | 24.32 | 10.01 | 2 | 7 | 0 | 0 | 0 | 0 |
| 10/6/2009 | 24.32 | 10.45 | 3 | 12 | 2 | 0 | 0 | 0 |
| 10/7/2009 | 24.38 | 11.02 | 10 | 25 | 2 | 0 | 0 | 0 |
| 10/8/2009 | 24.13 | 11.17 | 5 | 27 | 2 | 0 | 0 | 0 |
| 10/9/2009 | 23.79 | 11.18 | 6 | 26 | 2 | 1 | 0 | 0 |
| 10/10/2009 | 23.45 | 10.91 | 4 | 22 | 1 | 0 | 0 | 0 |
| 10/11/2009 | 23.19 | 10.73 | 5 | 13 | 1 | 0 | 0 | 0 |
| 10/12/2009 | 23.42 | 10.06 | 0 | 0 | 0 | 0 | 0 | 0 |
| 10/13/2009 | 26.65 | 9.83 | 0 | 0 | 0 | 0 | 0 | 0 |
| 10/14/2009 | 29.17 | 9.97 | 8 | 233 | 27 | 0 | 0 | 0 |
| 10/15/2009 | 39.36 | 10.89 | 35 | 931 | 26 | 0 | 0 | 0 |
| 10/16/2009 | 34.26 | 11.79 | 26 | 535 | 23 | 2 | 0 | 0 |
| 10/17/2009 | 27.89 | 12.82 | 19 | 182 | 14 | 0 | 0 | 7 |
| 10/18/2009 | 26.25 | 12.62 | 4 | 68 | 4 | 0 | 0 | 0 |

| Date | Discharge (cms) | Temp (C°) | Chinook | Coho | Steelhead | Cutthroat | Lamprey | Sucker |
|---|---|---|---|---|---|---|---|---|
| 10/19/2009 | 26.22 | 12.50 | 1 | 38 | 1 | 0 | 0 | 0 |
| 10/20/2009 | 25.06 | 11.97 | 0 | 0 | 0 | 1 | 0 | 0 |
| 10/21/2009 | 24.81 | 11.79 | 1 | 68 | 5 | 0 | 0 | 0 |
| 10/22/2009 | 25.09 | 11.59 | 2 | 62 | 5 | 0 | 0 | 0 |
| 10/23/2009 | 25.57 | 11.32 | 0 | 45 | 1 | 0 | 0 | 0 |
| 10/24/2009 | 27.07 | 10.84 | 1 | 48 | 0 | 0 | 0 | 1 |
| 10/25/2009 | 28.88 | 11.04 | 1 | 57 | 5 | 0 | 0 | 1 |
| 10/26/2009 | 26.90 | 11.41 | 0 | 18 | 1 | 0 | 0 | 0 |
| 10/27/2009 | 33.13 | 10.17 | 0 | 115 | 1 | 1 | 0 | 0 |
| 10/28/2009 | 43.04 | 9.41 | 3 | 803 | 4 | 1 | 0 | 0 |
| 10/29/2009 | 36.53 | 9.42 | 1 | 155 | 5 | 1 | 0 | 0 |
| 10/30/2009 | 51.54 | 9.47 | 3 | 109 | 3 | 0 | 0 | 0 |
| 10/31/2009 | 51.25 | 9.73 | 5 | 889 | 11 | 0 | 0 | 0 |
| 11/1/2009 | 49.84 | 9.74 | 2 | 247 | 8 | 0 | 0 | 1 |
| 11/2/2009 | 42.48 | 9.96 | 0 | 115 | 13 | 0 | 0 | 0 |
| 11/3/2009 | 35.11 | 9.93 | 2 | 27 | 0 | 0 | 0 | 0 |
| 11/4/2009 | 31.15 | 9.31 | 0 | 0 | 3 | 0 | 0 | 0 |
| 11/5/2009 | 29.17 | 9.09 | 0 | 12 | 1 | 0 | 0 | 0 |
| 11/6/2009 | 30.30 | 9.06 | 0 | 23 | 4 | 0 | 0 | 0 |
| 11/7/2009 | 58.90 | 9.00 | 1 | 95 | 9 | 0 | 0 | 0 |
| 11/8/2009 | 54.93 | 9.12 | 1 | 539 | 6 | 0 | 0 | 0 |
| 11/9/2009 | 51.82 | 8.81 | 0 | 161 | 3 | 0 | 0 | 0 |
| 11/10/2009 | 43.04 | 8.08 | 0 | 20 | 0 | 0 | 0 | 0 |
| 11/11/2009 | 45.31 | 8.15 | 0 | 0 | 0 | 0 | 0 | 0 |
| 11/12/2009 | 46.72 | 7.81 | 0 | 0 | 0 | 0 | 0 | 0 |
| 11/13/2009 | 51.54 | 7.13 | 1 | 24 | 1 | 0 | 0 | 0 |
| 11/14/2009 | 47.57 | 6.71 | 0 | 11 | 2 | 0 | 0 | 0 |
| 11/15/2009 | 41.06 | 6.35 | 1 | 8 | 0 | 0 | 0 | 0 |
| 11/16/2009 | 37.38 | 6.38 | 0 | 1 | 0 | 0 | 0 | 0 |
| 11/17/2009 | 39.64 | 7.04 | 0 | 0 | 0 | 0 | 0 | 0 |
| 11/18/2009 | 127.43 | 7.01 | 0 | 3 | 3 | 0 | 0 | 0 |
| 11/19/2009 | 113.27 | 7.31 | 0 | 385 | 18 | 0 | 0 | 0 |
| 11/20/2009 | 99.11 | 7.13 | 0 | 274 | 9 | 0 | 0 | 0 |
| 11/21/2009 | 84.95 | 6.38 | 0 | 116 | 2 | 0 | 0 | 0 |
| 11/22/2009 | 107.60 | 6.85 | 0 | 154 | 5 | 0 | 0 | 0 |
| 11/23/2009 | 155.74 | 6.58 | 0 | 36 | 1 | 0 | 0 | 0 |
| 11/24/2009 | 79.29 | 7.01 | 0 | 18 | 2 | 0 | 0 | 0 |
| 11/25/2009 | 65.98 | 6.56 | 0 | 72 | 17 | 0 | 0 | 0 |
| 11/26/2009 | 65.13 | 6.36 | 0 | 40 | 13 | 0 | 0 | 0 |
| 11/27/2009 | 109.30 | 7.04 | 0 | 145 | 30 | 0 | 0 | 0 |
| 11/28/2009 | 158.01 | 7.38 | 0 | 80 | 11 | 0 | 0 | 0 |
| 11/29/2009 | 100.81 | 6.88 | 0 | 50 | 6 | 0 | 0 | 0 |
| 11/30/2009 | 77.02 | 6.31 | 0 | 11 | 7 | 0 | 0 | 0 |
| 12/1/2009 | 64.85 | 5.89 | 0 | 3 | 5 | 0 | 0 | 0 |
| 12/2/2009 | 55.78 | 5.87 | 0 | 3 | 2 | 0 | 0 | 0 |

| Date | Discharge (cms) | Temp (C°) | Chinook | Coho | Steelhead | Cutthroat | Lamprey | Sucker |
|---|---|---|---|---|---|---|---|---|
| 12/3/2009 | 49.27 | 5.53 | 0 | 0 | 3 | 0 | 0 | 0 |
| 12/4/2009 | 45.87 | 4.58 | 0 | 0 | 0 | 0 | 0 | 0 |
| 12/5/2009 | 43.04 | 4.12 | 0 | 1 | 0 | 0 | 0 | 0 |
| 12/6/2009 | 40.78 | 3.47 | 0 | 0 | 0 | 0 | 0 | 0 |
| 12/7/2009 | 38.51 | 2.20 | 0 | 0 | 0 | 0 | 0 | 0 |
| 12/8/2009 | 35.96 | 0.48 | 0 | 0 | 0 | 0 | 0 | 0 |
| 12/9/2009 | 35.11 | -0.06 | 0 | 0 | 0 | 0 | 0 | 0 |
| 12/10/2009 | 34.26 | -0.03 | 0 | 0 | 0 | 0 | 0 | 0 |
| 12/11/2009 | 33.70 | -0.06 | 0 | 0 | 0 | 0 | 0 | 0 |
| 12/12/2009 | 35.96 | -0.05 | 0 | 0 | 0 | 0 | 0 | 0 |
| 12/13/2009 | 34.83 | 0.16 | 0 | 0 | 0 | 0 | 0 | 0 |
| 12/14/2009 | 34.55 | 0.80 | 0 | 0 | 0 | 0 | 0 | 0 |
| 12/15/2009 | 39.93 | 3.21 | 0 | 0 | 1 | 0 | 0 | 0 |
| 12/16/2009 | 111.85 | 4.55 | 0 | 0 | 9 | 0 | 0 | 0 |
| 12/17/2009 | 110.15 | 6.01 | 0 | 22 | 24 | 0 | 0 | 0 |
| 12/18/2009 | 94.01 | 6.78 | 0 | 24 | 37 | 0 | 0 | 0 |
| 12/19/2009 | 76.46 | 7.15 | 0 | 11 | 23 | 0 | 0 | 0 |
| 12/20/2009 | 69.66 | 7.41 | 0 | 9 | 44 | 0 | 0 | 0 |
| 12/21/2009 | 82.69 | 7.48 | 0 | 11 | 57 | 0 | 0 | 0 |
| 12/22/2009 | 131.96 | 6.93 | 0 | 2 | 28 | 0 | 0 | 0 |
| 12/23/2009 | 106.19 | 5.64 | 0 | 0 | 4 | 0 | 0 | 0 |
| 12/24/2009 | 84.67 | 4.90 | 0 | 0 | 1 | 0 | 0 | 0 |
| 12/25/2009 | 77.87 | 4.42 | 0 | 0 | 0 | 0 | 0 | 0 |
| 12/26/2009 | 73.06 | 4.08 | 0 | 0 | 0 | 0 | 0 | 0 |
| 12/27/2009 | 65.98 | 4.02 | 0 | 0 | 1 | 0 | 0 | 0 |
| 12/28/2009 | 59.75 | 4.33 | 0 | 0 | 1 | 0 | 0 | 0 |
| 12/29/2009 | 56.07 | 4.43 | 0 | 0 | 2 | 0 | 0 | 0 |
| 12/30/2009 | 65.13 | 4.38 | 0 | 0 | 4 | 0 | 0 | 0 |
| 12/31/2009 | 108.74 | 4.83 | 0 | 0 | 20 | 0 | 0 | 0 |
| 1/1/2010 | 620.14 | 6.78 | 0 | 0 | 2 | 0 | 0 | 0 |
| 1/2/2010 | 387.94 | 7.50 | 0 | 0 | 14 | 0 | 0 | 0 |
| 1/3/2010 | 214.36 | 7.12 | 0 | 2 | 71 | 0 | 0 | 0 |
| 1/4/2010 | 148.66 | 7.01 | 0 | 1 | 74 | 0 | 0 | 0 |
| 1/5/2010 | 122.05 | 7.35 | 0 | 3 | 203 | 0 | 0 | 0 |
| 1/6/2010 | 208.13 | 8.05 | 0 | 1 | 113 | 0 | 0 | 0 |
| 1/7/2010 | 274.67 | 8.44 | 0 | 1 | 78 | 0 | 0 | 0 |
| 1/8/2010 | 193.40 | 8.26 | 0 | 0 | 189 | 0 | 0 | 0 |
| 1/9/2010 | 159.42 | 8.39 | 0 | 0 | 119 | 0 | 0 | 0 |
| 1/10/2010 | 133.37 | 8.32 | 0 | 0 | 108 | 0 | 0 | 0 |
| 1/11/2010 | 112.42 | 7.97 | 0 | 0 | 74 | 0 | 0 | 0 |
| 1/12/2010 | 104.21 | 8.01 | 0 | 0 | 73 | 0 | 0 | 0 |
| 1/13/2010 | 123.18 | 8.67 | 0 | 0 | 96 | 0 | 0 | 0 |
| 1/14/2010 | 135.92 | 7.88 | 0 | 0 | 30 | 0 | 0 | 0 |
| 1/15/2010 | 120.91 | 7.54 | 0 | 0 | 42 | 0 | 0 | 0 |
| 1/16/2010 | 121.20 | 7.47 | 0 | 0 | 67 | 0 | 0 | 0 |

| Date | Discharge (cms) | Temp (C°) | Chinook | Coho | Steelhead | Cutthroat | Lamprey | Sucker |
|---|---|---|---|---|---|---|---|---|
| 1/17/2010 | 148.38 | 8.04 | 0 | 0 | 134 | 0 | 0 | 0 |
| 1/18/2010 | 143.00 | 7.63 | 0 | 0 | 48 | 0 | 0 | 0 |
| 1/19/2010 | 127.14 | 7.36 | 0 | 0 | 48 | 0 | 0 | 0 |
| 1/20/2010 | 106.47 | 7.47 | 0 | 0 | 29 | 0 | 0 | 0 |
| 1/21/2010 | 92.03 | 7.21 | 0 | 0 | 18 | 0 | 0 | 0 |
| 1/22/2010 | 84.38 | 7.30 | 0 | 0 | 20 | 0 | 0 | 0 |
| 1/23/2010 | 76.17 | 6.81 | 0 | 0 | 8 | 0 | 0 | 0 |
| 1/24/2010 | 73.06 | 6.50 | 0 | 0 | 8 | 0 | 0 | 0 |
| 1/25/2010 | 120.91 | 6.65 | 0 | 0 | 45 | 0 | 0 | 0 |
| 1/26/2010 | 126.58 | 7.51 | 0 | 0 | 122 | 0 | 0 | 0 |
| 1/27/2010 | 116.67 | 7.51 | 0 | 0 | 38 | 0 | 0 | 0 |
| 1/28/2010 | 103.64 | 7.39 | 0 | 0 | 33 | 0 | 0 | 0 |
| 1/29/2010 | 91.18 | 7.05 | 0 | 0 | 49 | 0 | 0 | 0 |
| 1/30/2010 | 83.82 | 7.59 | 0 | 0 | 92 | 0 | 0 | 0 |
| 1/31/2010 | 80.14 | 8.02 | 0 | 0 | 84 | 0 | 0 | 0 |
| 2/1/2010 | 77.30 | 7.47 | 0 | 0 | 24 | 0 | 0 | 0 |
| 2/2/2010 | 88.35 | 7.12 | 0 | 0 | 17 | 0 | 0 | 0 |
| 2/3/2010 | 92.03 | 7.22 | 0 | 0 | 43 | 0 | 0 | 0 |
| 2/4/2010 | 85.80 | 7.42 | 0 | 0 | 66 | 1 | 0 | 0 |
| 2/5/2010 | 82.12 | 7.78 | 0 | 0 | 72 | 0 | 0 | 0 |
| 2/6/2010 | 77.30 | 7.52 | 0 | 0 | 46 | 0 | 0 | 0 |
| 2/7/2010 | 73.91 | 7.70 | 0 | 0 | 144 | 0 | 0 | 0 |
| 2/8/2010 | 69.38 | 7.84 | 0 | 0 | 97 | 0 | 0 | 0 |
| 2/9/2010 | 65.98 | 7.55 | 0 | 0 | 20 | 0 | 0 | 0 |
| 2/10/2010 | 62.58 | 7.07 | 0 | 0 | 2 | 0 | 0 | 0 |
| 2/11/2010 | 62.01 | 6.93 | 0 | 0 | 10 | 0 | 0 | 0 |
| 2/12/2010 | 68.24 | 7.47 | 0 | 0 | 82 | 0 | 0 | 0 |
| 2/13/2010 | 86.08 | 7.60 | 0 | 0 | 354 | 0 | 0 | 0 |
| 2/14/2010 | 91.75 | 7.73 | 0 | 0 | 208 | 0 | 0 | 0 |
| 2/15/2010 | 114.12 | 8.39 | 0 | 0 | 550 | 0 | 0 | 4 |
| 2/16/2010 | 116.10 | 8.95 | 0 | 0 | 304 | 0 | 0 | 15 |
| 2/17/2010 | 101.94 | 8.76 | 0 | 0 | 235 | 0 | 0 | 3 |
| 2/18/2010 | 90.05 | 8.78 | 0 | 0 | 114 | 0 | 0 | 0 |
| 2/19/2010 | 80.70 | 7.79 | 0 | 0 | 8 | 0 | 0 | 0 |
| 2/20/2010 | 73.06 | 6.99 | 0 | 0 | 1 | 0 | 0 | 0 |
| 2/21/2010 | 67.11 | 6.38 | 0 | 0 | 2 | 0 | 0 | 0 |
| 2/22/2010 | 60.88 | 5.82 | 0 | 0 | 1 | 0 | 0 | 0 |
| 2/23/2010 | 57.77 | 5.76 | 0 | 0 | 0 | 0 | 0 | 0 |
| 2/24/2010 | 62.01 | 6.26 | 0 | 0 | 6 | 0 | 0 | 0 |
| 2/25/2010 | 80.99 | 7.64 | 0 | 0 | 117 | 0 | 0 | 0 |
| 2/26/2010 | 96.56 | 7.90 | 0 | 0 | 484 | 0 | 0 | 0 |
| 2/27/2010 | 92.60 | 8.33 | 0 | 0 | 33 | 0 | 0 | 0 |
| 2/28/2010 | 82.69 | 8.48 | 0 | 0 | 102 | 0 | 0 | 0 |
| 3/1/2010 | 73.91 | 7.63 | 0 | 0 | 50 | 0 | 0 | 0 |
| 3/2/2010 | 67.96 | 8.20 | 0 | 0 | 74 | 0 | 0 | 0 |

| Date | Discharge (cms) | Temp (C°) | Chinook | Coho | Steelhead | Cutthroat | Lamprey | Sucker |
|---|---|---|---|---|---|---|---|---|
| 3/3/2010 | 67.39 | 8.22 | 0 | 0 | 161 | 0 | 0 | 0 |
| 3/4/2010 | 67.96 | 8.38 | 0 | 0 | 80 | 0 | 0 | 0 |
| 3/5/2010 | 65.41 | 8.48 | 0 | 0 | 60 | 0 | 0 | 0 |
| 3/6/2010 | 62.01 | 8.33 | 0 | 0 | 16 | 0 | 0 | 0 |
| 3/7/2010 | 58.62 | 7.94 | 0 | 0 | 14 | 0 | 0 | 0 |
| 3/8/2010 | 57.77 | 7.72 | 0 | 0 | 13 | 0 | 0 | 0 |
| 3/9/2010 | 56.07 | 7.30 | 0 | 0 | 5 | 0 | 0 | 0 |
| 3/10/2010 | 58.33 | 7.08 | 0 | 0 | 5 | 0 | 0 | 0 |
| 3/11/2010 | 57.20 | 7.00 | 0 | 0 | 4 | 1 | 0 | 0 |
| 3/12/2010 | 73.34 | 6.98 | 0 | 0 | 29 | 0 | 0 | 0 |
| 3/13/2010 | 124.88 | 7.06 | 0 | 0 | 55 | 0 | 0 | 0 |
| 3/14/2010 | 107.60 | 7.08 | 0 | 0 | 17 | 0 | 0 | 0 |
| 3/15/2010 | 94.58 | 7.33 | 0 | 0 | 10 | 0 | 0 | 0 |
| 3/16/2010 | 95.43 | 7.91 | 0 | 0 | 0 | 0 | 0 | 0 |
| 3/17/2010 | 93.45 | 8.14 | 0 | 0 | 251 | 0 | 0 | 0 |
| 3/18/2010 | 82.40 | 8.75 | 0 | 0 | 223 | 0 | 0 | 1 |
| 3/19/2010 | 73.91 | 8.77 | 0 | 0 | 198 | 0 | 0 | 1 |
| 3/20/2010 | 67.68 | 9.13 | 1 | 0 | 423 | 0 | 0 | 7 |
| 3/21/2010 | 63.15 | 9.15 | 3 | 0 | 434 | 3 | 0 | 46 |
| 3/22/2010 | 67.68 | 9.56 | 1 | 0 | 222 | 2 | 0 | 27 |
| 3/23/2010 | 69.38 | 9.54 | 0 | 0 | 26 | 0 | 0 | 15 |
| 3/24/2010 | 65.41 | 9.75 | 0 | 0 | 95 | 0 | 0 | 0 |
| 3/25/2010 | 70.79 | 9.42 | 6 | 0 | 151 | 0 | 0 | 2 |
| 3/26/2010 | 116.67 | 8.53 | 0 | 0 | 129 | 0 | 0 | 3 |
| 3/27/2010 | 151.50 | 8.63 | 0 | 0 | 31 | 0 | 0 | 0 |
| 3/28/2010 | 135.92 | 8.56 | 2 | 0 | 66 | 0 | 0 | 0 |
| 3/29/2010 | 175.00 | 8.67 | 0 | 0 | 158 | 0 | 0 | 0 |
| 3/30/2010 | 385.11 | 7.86 | 0 | 0 | 3 | 0 | 0 | 0 |
| 3/31/2010 | 294.50 | 7.57 | 0 | 0 | 6 | 0 | 0 | 0 |
| 4/1/2010 | 206.43 | 7.94 | 0 | 0 | 19 | 0 | 0 | 0 |
| 4/2/2010 | 188.02 | 7.70 | 0 | 0 | 18 | 0 | 0 | 0 |
| 4/3/2010 | 242.96 | 7.24 | 0 | 0 | 5 | 0 | 0 | 0 |
| 4/4/2010 | 190.57 | 7.37 | 1 | 0 | 32 | 1 | 0 | 0 |
| 4/5/2010 | 158.86 | 7.55 | 0 | 0 | 49 | 0 | 0 | 0 |
| 4/6/2010 | 139.89 | 7.83 | 0 | 0 | 41 | 0 | 0 | 0 |
| 4/7/2010 | 133.94 | 9.04 | 0 | 0 | 105 | 0 | 0 | 0 |
| 4/8/2010 | 153.48 | 9.01 | 5 | 0 | 233 | 3 | 0 | 0 |
| 4/9/2010 | 151.50 | 8.28 | 0 | 0 | 20 | 1 | 0 | 0 |
| 4/10/2010 | 127.14 | 7.90 | 0 | 0 | 16 | 0 | 0 | 0 |
| 4/11/2010 | 134.51 | 8.08 | 6 | 0 | 119 | 0 | 0 | 0 |
| 4/12/2010 | 142.72 | 8.80 | 6 | 0 | 91 | 0 | 0 | 0 |
| 4/13/2010 | 149.51 | 9.22 | 8 | 0 | 144 | 0 | 0 | 13 |
| 4/14/2010 | 154.61 | 9.26 | 3 | 0 | 17 | 0 | 0 | 15 |
| 4/15/2010 | 169.33 | 9.55 | 8 | 0 | 137 | 0 | 0 | 6 |
| 4/16/2010 | 153.19 | 10.14 | 7 | 0 | 94 | 0 | 0 | 91 |

| Date | Discharge (cms) | Temp (C°) | Chinook | Coho | Steelhead | Cutthroat | Lamprey | Sucker |
|---|---|---|---|---|---|---|---|---|
| 4/17/2010 | 138.75 | 10.38 | 8 | 0 | 53 | 0 | 0 | 67 |
| 4/18/2010 | 128.84 | 11.20 | 7 | 0 | 80 | 0 | 0 | 42 |
| 4/19/2010 | 120.35 | 10.39 | 15 | 0 | 39 | 1 | 0 | 13 |
| 4/20/2010 | 122.90 | 9.90 | 20 | 0 | 35 | 1 | 0 | 5 |
| 4/21/2010 | 142.72 | 9.36 | 0 | 0 | 5 | 0 | 0 | 0 |
| 4/22/2010 | 131.67 | 9.68 | 8 | 0 | 8 | 0 | 0 | 0 |
| 4/23/2010 | 112.98 | 10.78 | 18 | 0 | 44 | 0 | 0 | 0 |
| 4/24/2010 | 98.83 | 11.26 | 28 | 0 | 40 | 0 | 0 | 4 |
| 4/25/2010 | 89.20 | 11.63 | 31 | 0 | 34 | 0 | 0 | 10 |
| 4/26/2010 | 82.12 | 10.51 | 0 | 0 | 2 | 0 | 0 | 4 |
| 4/27/2010 | 132.24 | 9.56 | 0 | 0 | 0 | 0 | 0 | 0 |
| 4/28/2010 | 251.17 | 8.86 | 5 | 0 | 8 | 0 | 0 | 0 |
| 4/29/2010 | 245.79 | 8.34 | 0 | 0 | 1 | 0 | 0 | 0 |
| 4/30/2010 | 242.68 | 8.91 | 6 | 0 | 12 | 0 | 0 | 0 |
| 5/1/2010 | 205.86 | 9.77 | 35 | 0 | 20 | 0 | 0 | 0 |
| 5/2/2010 | 178.40 | 10.81 | 74 | 0 | 28 | 0 | 0 | 12 |
| 5/3/2010 | 156.88 | 9.85 | 1 | 0 | 1 | 0 | 0 | 1 |
| 5/4/2010 | 167.92 | 9.52 | 0 | 0 | 0 | 0 | 0 | 0 |
| 5/5/2010 | 150.36 | 9.20 | 1 | 0 | 0 | 0 | 0 | 0 |
| 5/6/2010 | 129.41 | 9.62 | 5 | 0 | 2 | 0 | 0 | 0 |
| 5/7/2010 | 113.83 | 11.23 | 53 | 0 | 23 | 0 | 0 | 1 |
| 5/8/2010 | 101.37 | 12.10 | 95 | 0 | 28 | 0 | 0 | 223 |
| 5/9/2010 | 93.73 | 12.23 | 116 | 0 | 17 | 0 | 0 | 327 |
| 5/10/2010 | 94.58 | 11.28 | 1 | 0 | 0 | 0 | 0 | 127 |
| 5/11/2010 | 120.91 | 9.74 | 0 | 0 | 0 | 0 | 0 | 0 |
| 5/12/2010 | 124.31 | 9.69 | 0 | 0 | 0 | 0 | 0 | 0 |
| 5/13/2010 | 108.74 | 12.14 | 13 | 0 | 1 | 1 | 0 | 169 |
| 5/14/2010 | 99.39 | 13.90 | 372 | 0 | 31 | 0 | 0 | 1533 |
| 5/15/2010 | 94.01 | 14.19 | 320 | 0 | 38 | 0 | 0 | 526 |
| 5/16/2010 | 89.76 | 14.63 | 201 | 0 | 16 | 0 | 0 | 315 |
| 5/17/2010 | 91.75 | 14.00 | 31 | 0 | 2 | 0 | 0 | 45 |
| 5/18/2010 | 90.90 | 13.48 | 0 | 0 | 0 | 1 | 0 | 0 |
| 5/19/2010 | 85.52 | 12.79 | 21 | 0 | 0 | 0 | 0 | 90 |
| 5/20/2010 | 91.75 | 12.11 | 77 | 0 | 11 | 0 | 0 | 3 |
| 5/21/2010 | 101.09 | 10.35 | 9 | 0 | 2 | 0 | 0 | 0 |
| 5/22/2010 | 133.94 | 9.27 | 7 | 0 | 0 | 0 | 0 | 0 |
| 5/23/2010 | 140.17 | 10.01 | 21 | 0 | 1 | 0 | 0 | 0 |
| 5/24/2010 | 129.12 | 9.96 | 1 | 0 | 0 | 0 | 0 | 0 |
| 5/25/2010 | 116.38 | 9.75 | 22 | 0 | 4 | 0 | 0 | 0 |
| 5/26/2010 | 132.81 | 10.15 | 2 | 0 | 0 | 0 | 0 | 0 |
| 5/27/2010 | 155.18 | 11.91 | 212 | 0 | 24 | 0 | 0 | 1 |
| 5/28/2010 | 150.93 | 11.19 | 58 | 0 | 10 | 1 | 0 | 0 |
| 5/29/2010 | 129.97 | 11.89 | 22 | 0 | 3 | 0 | 0 | 2 |
| 5/30/2010 | 111.00 | 12.91 | 308 | 0 | 24 | 0 | 0 | 117 |
| 5/31/2010 | 99.68 | 12.35 | 16 | 0 | 7 | 0 | 0 | 30 |

| Date | Discharge (cms) | Temp (C°) | Chinook | Coho | Steelhead | Cutthroat | Lamprey | Sucker |
|---|---|---|---|---|---|---|---|---|
| 6/1/2010 | 96.56 | 12.78 | 34 | 0 | 2 | 2 | 0 | 92 |
| 6/2/2010 | 118.65 | 12.59 | 46 | 0 | 14 | 0 | 0 | 41 |
| 6/3/2010 | 472.89 | 10.41 | 11 | 0 | 12 | 0 | 0 | 0 |
| 6/4/2010 | 566.34 | 10.31 | 0 | 0 | 0 | 0 | 0 | 0 |
| 6/5/2010 | 430.42 | 10.59 | 7 | 0 | 6 | 0 | 0 | 0 |
| 6/6/2010 | 277.79 | 12.07 | 22 | 0 | 13 | 0 | 0 | 0 |
| 6/7/2010 | 214.92 | 12.08 | 8 | 0 | 0 | 0 | 0 | 0 |
| 6/8/2010 | 173.58 | 12.86 | 51 | 0 | 1 | 1 | 0 | 0 |
| 6/9/2010 | 149.80 | 12.82 | 138 | 0 | 25 | 0 | 0 | 59 |
| 6/10/2010 | 161.69 | 12.86 | 231 | 0 | 50 | 0 | 0 | 8 |
| 6/11/2010 | 187.46 | 11.96 | 70 | 0 | 10 | 0 | 0 | 1 |
| 6/12/2010 | 162.54 | 13.22 | 119 | 0 | 40 | 1 | 0 | 22 |
| 6/13/2010 | 136.49 | 15.04 | 237 | 0 | 61 | 1 | 0 | 338 |
| 6/14/2010 | 118.36 | 14.73 | 43 | 0 | 10 | 0 | 0 | 88 |
| 6/15/2010 | 105.62 | 15.22 | 113 | 0 | 51 | 2 | 0 | 165 |
| 6/16/2010 | 95.14 | 13.77 | 159 | 0 | 95 | 0 | 0 | 44 |
| 6/17/2010 | 87.22 | 13.29 | 29 | 0 | 26 | 0 | 0 | 6 |
| 6/18/2010 | 79.29 | 13.09 | 125 | 0 | 87 | 0 | 0 | 18 |
| 6/19/2010 | 75.32 | 13.81 | 83 | 0 | 72 | 0 | 0 | 17 |
| 6/20/2010 | 71.36 | 14.01 | 121 | 0 | 131 | 1 | 0 | 67 |
| 6/21/2010 | 67.39 | 14.06 | 11 | 0 | 26 | 0 | 1 | 6 |
| 6/22/2010 | 63.71 | 15.19 | 22 | 0 | 10 | 1 | 0 | 470 |
| 6/23/2010 | 61.16 | 16.42 | 117 | 0 | 174 | 1 | 0 | 136 |
| 6/24/2010 | 57.48 | 16.95 | 45 | 0 | 39 | 5 | 0 | 451 |
| 6/25/2010 | 55.50 | 18.14 | 79 | 0 | 135 | 4 | 1 | 209 |
| 6/26/2010 | 53.24 | 18.37 | 85 | 0 | 113 | 8 | 0 | 309 |
| 6/27/2010 | 51.25 | 18.84 | 61 | 0 | 98 | 6 | 0 | 324 |
| 6/28/2010 | 50.12 | 19.54 | 27 | 0 | 40 | 1 | 0 | 64 |
| 6/29/2010 | 48.99 | 19.57 | 25 | 0 | 57 | 2 | 0 | 128 |
| 6/30/2010 | 48.42 | 17.96 | 25 | 0 | 80 | 4 | 1 | 30 |
| 7/1/2010 | 47.01 | 18.33 | 77 | 0 | 166 | 1 | 0 | 215 |
| 7/2/2010 | 44.74 | 17.84 | 58 | 0 | 128 | 2 | 0 | 37 |
| 7/3/2010 | 44.74 | 17.16 | 22 | 0 | 62 | 1 | 0 | 1 |
| 7/4/2010 | 43.32 | 17.76 | 26 | 0 | 54 | 1 | 0 | 123 |
| 7/5/2010 | 41.34 | 18.30 | 51 | 0 | 97 | 2 | 0 | 202 |
| 7/6/2010 | 40.49 | 18.65 | 40 | 0 | 46 | 2 | 0 | 25 |
| 7/7/2010 | 39.93 | 19.64 | 36 | 0 | 54 | 1 | 1 | 175 |
| 7/8/2010 | 38.51 | 20.58 | 65 | 0 | 70 | 4 | 2 | 215 |
| 7/9/2010 | 37.38 | 21.79 | 62 | 0 | 88 | 5 | 10 | 670 |
| 7/10/2010 | 36.81 | 21.74 | 46 | 0 | 70 | 6 | 5 | 25 |
| 7/11/2010 | 35.96 | 22.21 | 30 | 0 | 33 | 4 | 16 | 70 |
| 7/12/2010 | 35.68 | 22.51 | 23 | 0 | 31 | 6 | 6 | 223 |
| 7/13/2010 | 35.40 | 20.92 | 11 | 0 | 15 | 3 | 3 | 17 |
| 7/14/2010 | 34.83 | 20.60 | 1 | 0 | 5 | 5 | 0 | 12 |
| 7/15/2010 | 33.98 | 21.25 | 2 | 0 | 9 | 2 | 0 | 26 |

| Date | Discharge (cms) | Temp (C°) | Chinook | Coho | Steelhead | Cutthroat | Lamprey | Sucker |
|---|---|---|---|---|---|---|---|---|
| 7/16/2010 | 33.13 | 22.07 | 6 | 0 | 2 | 1 | 1 | 18 |
| 7/17/2010 | 32.56 | 22.04 | 0 | 0 | 1 | 2 | 2 | 12 |
| 7/18/2010 | 32.28 | 21.85 | 7 | 0 | 5 | 0 | 1 | 4 |
| 7/19/2010 | 31.71 | 21.15 | 0 | 0 | 1 | 0 | 7 | 2 |
| 7/20/2010 | 31.71 | 20.95 | 0 | 0 | 0 | 0 | 0 | 0 |
| 7/21/2010 | 31.43 | 20.52 | 1 | 0 | 1 | 0 | 0 | 2 |
| 7/22/2010 | 30.87 | 21.14 | 16 | 0 | 4 | 0 | 0 | 12 |
| 7/23/2010 | 30.02 | 21.64 | 23 | 0 | 6 | 0 | 0 | 23 |
| 7/24/2010 | 29.17 | 21.93 | 23 | 0 | 8 | 0 | 0 | 2 |
| 7/25/2010 | 28.60 | 22.64 | 33 | 0 | 10 | 1 | 3 | 0 |
| 7/26/2010 | 28.12 | 23.16 | 22 | 0 | 12 | 1 | 3 | 1 |
| 7/27/2010 | 27.55 | 23.36 | 19 | 0 | 4 | 0 | 7 | 0 |
| 7/28/2010 | 27.52 | 23.62 | 7 | 0 | 3 | 0 | 2 | 1 |
| 7/29/2010 | 28.01 | 23.37 | 6 | 0 | 3 | 0 | 1 | 0 |
| 7/30/2010 | 28.03 | 22.83 | 3 | 0 | 1 | 0 | 1 | 1 |
| 7/31/2010 | 27.75 | 21.70 | 1 | 0 | 2 | 0 | 0 | 0 |
| 8/1/2010 | 27.30 | 21.25 | 4 | 0 | 3 | 0 | 0 | 0 |
| 8/2/2010 | 26.79 | 21.01 | 10 | 0 | 3 | 0 | 0 | 0 |
| 8/3/2010 | 26.14 | 21.89 | 12 | 0 | 19 | 0 | 0 | 0 |
| 8/4/2010 | 26.02 | 21.56 | 22 | 0 | 28 | 1 | 1 | 0 |
| 8/5/2010 | 26.02 | 21.47 | 24 | 0 | 19 | 1 | 0 | 0 |
| 8/6/2010 | 25.91 | 22.03 | 28 | 0 | 34 | 0 | 1 | 0 |
| 8/7/2010 | 25.97 | 22.17 | 29 | 0 | 25 | 1 | 0 | 0 |
| 8/8/2010 | 25.82 | 22.14 | 13 | 0 | 38 | 0 | 0 | 0 |
| 8/9/2010 | 25.77 | 20.99 | 16 | 0 | 29 | 0 | 1 | 0 |
| 8/10/2010 | 25.63 | 20.70 | 6 | 0 | 16 | 0 | 0 | 0 |
| 8/11/2010 | 25.46 | 20.89 | 6 | 0 | 26 | 2 | 0 | 0 |
| 8/12/2010 | 24.61 | 21.45 | 12 | 0 | 36 | 0 | 0 | 0 |
| 8/13/2010 | 24.18 | 21.79 | 9 | 0 | 45 | 1 | 0 | 0 |
| 8/14/2010 | 24.07 | 22.35 | 15 | 0 | 46 | 1 | 1 | 0 |
| 8/15/2010 | 23.96 | 22.57 | 20 | 0 | 33 | 1 | 0 | 0 |
| 8/16/2010 | 23.76 | 22.40 | 8 | 0 | 29 | 0 | 1 | 0 |
| 8/17/2010 | 23.47 | 22.97 | 6 | 0 | 11 | 0 | 0 | 0 |
| 8/18/2010 | 22.88 | 22.48 | 5 | 0 | 14 | 1 | 1 | 0 |
| 8/19/2010 | 22.40 | 21.42 | 4 | 0 | 4 | 0 | 0 | 0 |
| 8/20/2010 | 22.34 | 21.36 | 2 | 0 | 3 | 1 | 0 | 0 |
| 8/21/2010 | 22.26 | 20.73 | 4 | 0 | 6 | 0 | 0 | 0 |
| 8/22/2010 | 22.14 | 19.57 | 21 | 0 | 6 | 0 | 0 | 0 |
| 8/23/2010 | 22.06 | 19.38 | 28 | 0 | 17 | 1 | 1 | 0 |
| 8/24/2010 | 21.86 | 19.59 | 17 | 0 | 16 | 0 | 0 | 0 |
| 8/25/2010 | 21.18 | 20.21 | 18 | 0 | 9 | 0 | 0 | 0 |
| 8/26/2010 | 20.36 | 20.85 | 29 | 1 | 14 | 2 | 0 | 0 |
| 8/27/2010 | 20.44 | 20.23 | 34 | 0 | 20 | 0 | 2 | 0 |
| 8/28/2010 | 20.50 | 19.25 | 13 | 0 | 19 | 1 | 0 | 0 |
| 8/29/2010 | 20.87 | 18.32 | 7 | 0 | 11 | 1 | 0 | 0 |

| Date | Discharge (cms) | Temp (C°) | Chinook | Coho | Steelhead | Cutthroat | Lamprey | Sucker |
|---|---|---|---|---|---|---|---|---|
| 8/30/2010 | 21.49 | 17.21 | 2 | 0 | 7 | 0 | 0 | 0 |
| 8/31/2010 | 24.83 | 16.63 | 4 | 0 | 1 | 0 | 0 | 0 |
| 9/1/2010 | 25.71 | 17.49 | 0 | 0 | 0 | 2 | 0 | 0 |
| 9/2/2010 | 23.93 | 17.91 | 30 | 0 | 48 | 1 | 0 | 0 |
| 9/3/2010 | 23.73 | 19.17 | 56 | 1 | 66 | 1 | 0 | 0 |
| 9/4/2010 | 23.73 | 19.34 | 55 | 2 | 46 | 4 | 0 | 0 |
| 9/5/2010 | 22.71 | 18.56 | 32 | 5 | 47 | 1 | 0 | 0 |
| 9/6/2010 | 22.20 | 18.24 | 12 | 3 | 35 | 0 | 0 | 0 |
| 9/7/2010 | 22.57 | 17.67 | 13 | 2 | 31 | 0 | 0 | 0 |
| 9/8/2010 | 23.98 | 16.85 | 2 | 1 | 17 | 0 | 0 | 0 |
| 9/9/2010 | 28.60 | 16.36 | 3 | 2 | 12 | 0 | 0 | 0 |
| 9/10/2010 | 29.45 | 16.18 | 1 | 0 | 7 | 1 | 0 | 0 |
| 9/11/2010 | 27.78 | 16.51 | 8 | 4 | 22 | 0 | 0 | 0 |
| 9/12/2010 | 26.48 | 16.70 | 3 | 5 | 24 | 0 | 0 | 0 |
| 9/13/2010 | 25.74 | 16.85 | 12 | 15 | 43 | 0 | 0 | 0 |
| 9/14/2010 | 25.20 | 16.98 | 4 | 13 | 40 | 0 | 0 | 0 |
| 9/15/2010 | 25.51 | 17.49 | 15 | 14 | 46 | 0 | 0 | 0 |
| 9/16/2010 | 24.32 | 17.23 | 12 | 16 | 18 | 0 | 0 | 0 |
| 9/17/2010 | 24.41 | 17.08 | 15 | 16 | 22 | 0 | 0 | 0 |
| 9/18/2010 | 25.88 | 16.57 | 7 | 24 | 17 | 2 | 0 | 0 |
| 9/19/2010 | 32.85 | 16.22 | 14 | 25 | 31 | 1 | 0 | 2 |
| 9/20/2010 | 47.86 | 16.06 | 84 | 79 | 38 | 0 | 0 | 12 |
| 9/21/2010 | 39.36 | 15.58 | 43 | 64 | 23 | 0 | 0 | 4 |
| 9/22/2010 | 32.56 | 15.36 | 9 | 40 | 6 | 1 | 0 | 0 |
| 9/23/2010 | 29.73 | 15.52 | 3 | 39 | 12 | 0 | 0 | 0 |
| 9/24/2010 | 27.98 | 15.51 | 4 | 28 | 10 | 0 | 0 | 0 |
| 9/25/2010 | 26.48 | 16.12 | 6 | 61 | 14 | 1 | 0 | 0 |
| 9/26/2010 | 25.34 | 16.27 | 7 | 79 | 15 | 1 | 0 | 0 |
| 9/27/2010 | 25.06 | 16.76 | 2 | 101 | 7 | 0 | 0 | 0 |
| 9/28/2010 | 25.80 | 17.12 | 16 | 210 | 13 | 0 | 0 | 0 |
| 9/29/2010 | 24.72 | 16.93 | 10 | 140 | 18 | 1 | 0 | 0 |
| 9/30/2010 | 24.15 | 17.42 | 9 | 148 | 14 | 0 | 0 | 0 |
| 10/1/2010 | 24.18 | 17.53 | 9 | 185 | 8 | 0 | 0 | 0 |
| 10/2/2010 | 24.04 | 16.32 | 6 | 128 | 9 | 0 | 0 | 0 |
| 10/3/2010 | 24.04 | 15.23 | 4 | 49 | 6 | 0 | 0 | 0 |
| 10/4/2010 | 24.41 | 14.82 | 4 | 44 | 0 | 0 | 0 | 0 |
| 10/5/2010 | 24.72 | 14.49 | 0 | 5 | 0 | 0 | 0 | 0 |
| 10/6/2010 | 24.98 | 14.09 | 0 | 0 | 2 | 0 | 0 | 0 |
| 10/7/2010 | 24.27 | 14.37 | 0 | 0 | 0 | 0 | 0 | 0 |
| 10/8/2010 | 23.81 | 14.80 | 5 | 71 | 5 | 0 | 0 | 0 |
| 10/9/2010 | 24.01 | 14.78 | 13 | 113 | 9 | 1 | 0 | 0 |
| 10/10/2010 | 25.03 | 14.59 | 8 | 155 | 9 | 1 | 0 | 0 |
| 10/11/2010 | 26.25 | 13.56 | 0 | 66 | 2 | 0 | 0 | 0 |
| 10/12/2010 | 26.25 | 13.21 | 0 | 9 | 1 | 0 | 0 | 0 |
| 10/13/2010 | 24.98 | 13.04 | 0 | 0 | 0 | 0 | 0 | 0 |

| Date | Discharge (cms) | Temp (C°) | Chinook | Coho | Steelhead | Cutthroat | Lamprey | Sucker |
|---|---|---|---|---|---|---|---|---|
| 10/14/2010 | 26.28 | 12.93 | 4 | 258 | 10 | 0 | 0 | 0 |
| 10/15/2010 | 26.31 | 12.75 | 3 | 303 | 9 | 0 | 0 | 0 |
| 10/16/2010 | 26.45 | 12.32 | 5 | 122 | 0 | 0 | 0 | 0 |
| 10/17/2010 | 25.66 | 11.85 | 1 | 50 | 2 | 0 | 0 | 0 |
| 10/18/2010 | 25.43 | 11.41 | 1 | 15 | 0 | 0 | 0 | 0 |
| 10/19/2010 | 24.07 | 11.11 | 2 | 2 | 0 | 0 | 0 | 0 |
| 10/20/2010 | 22.91 | 11.12 | 0 | 6 | 0 | 0 | 0 | 0 |
| 10/21/2010 | 23.22 | 11.03 | 0 | 0 | 0 | 0 | 0 | 0 |
| 10/22/2010 | 23.73 | 11.24 | 12 | 77 | 4 | 0 | 0 | 0 |
| 10/23/2010 | 24.72 | 10.97 | 6 | 51 | 7 | 1 | 0 | 0 |
| 10/24/2010 | 35.96 | 10.99 | 22 | 149 | 16 | 0 | 0 | 0 |
| 10/25/2010 | 105.91 | 9.97 | 8 | 493 | 9 | 0 | 0 | 0 |
| 10/26/2010 | 97.69 | 9.55 | 9 | 1532 | 16 | 0 | 0 | 0 |
| 10/27/2010 | 84.38 | 8.72 | 0 | 46 | 0 | 0 | 0 | 0 |
| 10/28/2010 | 56.92 | 8.61 | 0 | 2 | 2 | 0 | 0 | 0 |
| 10/29/2010 | 65.13 | 9.24 | 1 | 297 | 11 | 0 | 0 | 0 |
| 10/30/2010 | 67.39 | 9.29 | 7 | 2399 | 58 | 1 | 0 | 0 |
| 10/31/2010 | 54.93 | 9.59 | 7 | 680 | 17 | 0 | 0 | 0 |
| 11/1/2010 | 71.08 | 9.96 | 2 | 171 | 7 | 0 | 0 | 0 |
| 11/2/2010 | 58.05 | 11.23 | 0 | 0 | 0 | 0 | 0 | 1 |
| 11/3/2010 | 49.27 | 11.91 | 0 | 5 | 8 | 2 | 0 | 0 |
| 11/4/2010 | 42.48 | 10.88 | 1 | 159 | 2 | 0 | 0 | 0 |
| 11/5/2010 | 38.23 | 10.63 | 1 | 304 | 9 | 0 | 0 | 0 |
| 11/6/2010 | 36.81 | 10.79 | 0 | 84 | 6 | 0 | 0 | 0 |
| 11/7/2010 | 65.13 | 10.91 | 5 | 100 | 9 | 0 | 0 | 0 |
| 11/8/2010 | 126.29 | 9.97 | 5 | 342 | 10 | 0 | 0 | 1 |
| 11/9/2010 | 91.75 | 8.93 | 0 | 114 | 4 | 0 | 0 | 0 |
| 11/10/2010 | 169.33 | 8.73 | 0 | 24 | 1 | 0 | 0 | 1 |
| 11/11/2010 | 139.32 | 8.77 | 0 | 92 | 6 | 1 | 0 | 0 |
| 11/12/2010 | 89.76 | 8.60 | 0 | 47 | 5 | 0 | 0 | 0 |
| 11/13/2010 | 70.79 | 8.32 | 0 | 25 | 4 | 0 | 0 | 0 |
| 11/14/2010 | 61.73 | 8.28 | 0 | 18 | 6 | 0 | 0 | 0 |
| 11/15/2010 | 66.26 | 8.56 | 0 | 8 | 0 | 0 | 0 | 0 |
| 11/16/2010 | 71.92 | 9.11 | 0 | 0 | 0 | 0 | 0 | 0 |
| 11/17/2010 | 74.76 | 8.85 | 0 | 0 | 0 | 0 | 0 | 0 |
| 11/18/2010 | 89.48 | 8.66 | 0 | 2 | 12 | 0 | 0 | 0 |
| 11/19/2010 | 125.44 | 7.89 | 0 | 25 | 7 | 0 | 0 | 0 |
| 11/20/2010 | 97.13 | 7.40 | 0 | 8 | 6 | 0 | 0 | 0 |
| 11/21/2010 | 81.55 | 6.91 | 0 | 11 | 1 | 0 | 0 | 0 |
| 11/22/2010 | 85.23 | 6.52 | 0 | 7 | 5 | 0 | 0 | 0 |
| 11/23/2010 | 131.39 | 6.12 | 0 | 5 | 1 | 0 | 0 | 0 |
| 11/24/2010 | 86.08 | 5.22 | 0 | 4 | 0 | 0 | 0 | 0 |
| 11/25/2010 | 67.68 | 4.67 | 0 | 2 | 0 | 0 | 0 | 0 |
| 11/26/2010 | 60.31 | 4.62 | 0 | 0 | 0 | 0 | 0 | 0 |
| 11/27/2010 | 102.51 | 5.23 | 0 | 1 | 2 | 0 | 0 | 0 |

| Date | Discharge (cms) | Temp (C°) | Chinook | Coho | Steelhead | Cutthroat | Lamprey | Sucker |
|---|---|---|---|---|---|---|---|---|
| 11/28/2010 | 147.25 | 5.84 | 0 | 3 | 1 | 0 | 0 | 0 |
| 11/29/2010 | 110.15 | 6.30 | 0 | 0 | 0 | 0 | 0 | 0 |
| 11/30/2010 | 91.18 | 6.51 | 0 | 0 | 0 | 0 | 0 | 0 |
| 12/1/2010 | 242.96 | 6.99 | 0 | 0 | 0 | 0 | 0 | 0 |
| 12/2/2010 | 356.79 | 7.88 | 0 | 0 | 0 | 1 | 0 | 0 |
| 12/3/2010 | 288.83 | 7.39 | 0 | 28 | 26 | 1 | 0 | 0 |
| 12/4/2010 | 182.64 | 6.98 | 0 | 33 | 36 | 0 | 0 | 0 |
| 12/5/2010 | 148.10 | 7.25 | 0 | 29 | 47 | 0 | 0 | 0 |
| 12/6/2010 | 153.76 | 7.36 | 0 | 15 | 32 | 1 | 0 | 0 |
| 12/7/2010 | 165.37 | 7.83 | 0 | 12 | 53 | 0 | 0 | 0 |
| 12/8/2010 | 162.26 | 8.05 | 0 | 6 | 38 | 0 | 0 | 0 |
| 12/9/2010 | 190.29 | 8.15 | 0 | 6 | 43 | 0 | 0 | 0 |
| 12/10/2010 | 236.16 | 8.27 | 0 | 4 | 36 | 1 | 0 | 0 |
| 12/11/2010 | 311.49 | 8.20 | 0 | 8 | 12 | 0 | 0 | 0 |
| 12/12/2010 | 447.41 | 8.87 | 0 | 4 | 24 | 1 | 0 | 0 |
| 12/13/2010 | 512.53 | 9.28 | 0 | 1 | 28 | 0 | 0 | 0 |
| 12/14/2010 | 620.14 | 9.01 | 0 | 0 | 0 | 0 | 0 | 0 |
| 12/15/2010 | 447.41 | 7.97 | 0 | 0 | 3 | 0 | 0 | 0 |
| 12/16/2010 | 286.00 | 7.19 | 0 | 0 | 1 | 0 | 0 | 0 |
| 12/17/2010 | 207.00 | 6.30 | 0 | 0 | 2 | 0 | 0 | 0 |
| 12/18/2010 | 183.21 | 6.35 | 0 | 1 | 15 | 0 | 0 | 0 |
| 12/19/2010 | 217.76 | 6.90 | 0 | 0 | 26 | 0 | 0 | 0 |
| 12/20/2010 | 213.79 | 6.79 | 0 | 0 | 24 | 0 | 0 | 0 |
| 12/21/2010 | 190.57 | 6.98 | 0 | 0 | 21 | 0 | 0 | 0 |
| 12/22/2010 | 164.52 | 7.31 | 0 | 1 | 48 | 0 | 0 | 0 |
| 12/23/2010 | 149.51 | 6.77 | 0 | 0 | 20 | 0 | 0 | 0 |
| 12/24/2010 | 128.56 | 6.87 | 0 | 0 | 32 | 0 | 0 | 0 |
| 12/25/2010 | 115.82 | 7.02 | 0 | 0 | 65 | 0 | 0 | 0 |
| 12/26/2010 | 142.15 | 6.99 | 0 | 0 | 34 | 0 | 0 | 0 |
| 12/27/2010 | 157.72 | 6.89 | 0 | 0 | 22 | 0 | 0 | 0 |
| 12/28/2010 | 325.64 | 7.30 | 0 | 0 | 23 | 1 | 0 | 0 |
| 12/29/2010 | 767.39 | 7.88 | 0 | 0 | 0 | 0 | 0 | 0 |
| 12/30/2010 | 379.45 | 7.31 | 0 | 0 | 0 | 0 | 0 | 0 |
| 12/31/2010 | 237.01 | 6.04 | 0 | 0 | 0 | 0 | 0 | 0 |
| 1/1/2011 | 183.49 | 5.26 | 0 | 0 | 0 | 0 | 0 | 0 |
| 1/2/2011 | 151.21 | 5.37 | 0 | 0 | 0 | 0 | 0 | 0 |
| 1/3/2011 | 129.41 | 5.30 | 0 | 0 | 6 | 0 | 0 | 0 |
| 1/4/2011 | 113.55 | 5.03 | 0 | 0 | 6 | 0 | 0 | 0 |
| 1/5/2011 | 104.49 | 5.15 | 0 | 0 | 11 | 0 | 0 | 0 |
| 1/6/2011 | 95.99 | 5.36 | 0 | 0 | 19 | 0 | 0 | 0 |
| 1/7/2011 | 95.99 | 5.34 | 0 | 0 | 18 | 0 | 0 | 0 |
| 1/8/2011 | 101.94 | 5.68 | 0 | 0 | 19 | 0 | 0 | 0 |
| 1/9/2011 | 105.06 | 5.55 | 0 | 0 | 11 | 0 | 0 | 0 |
| 1/10/2011 | 94.86 | 5.16 | 0 | 0 | 3 | 0 | 0 | 0 |
| 1/11/2011 | 87.78 | 4.39 | 0 | 0 | 0 | 0 | 0 | 0 |

| Date | Discharge (cms) | Temp (C°) | Chinook | Coho | Steelhead | Cutthroat | Lamprey | Sucker |
|---|---|---|---|---|---|---|---|---|
| 1/12/2011 | 82.40 | 4.75 | 0 | 0 | 1 | 0 | 0 | 0 |
| 1/13/2011 | 101.09 | 6.20 | 0 | 0 | 81 | 0 | 0 | 0 |
| 1/14/2011 | 328.48 | 7.16 | 0 | 0 | 66 | 0 | 0 | 0 |
| 1/15/2011 | 254.00 | 7.68 | 0 | 0 | 161 | 0 | 0 | 0 |
| 1/16/2011 | 1047.72 | 8.15 | 0 | 0 | 3 | 0 | 0 | 0 |
| 1/17/2011 | 1104.36 | 8.45 | 0 | 0 | 0 | 0 | 0 | 0 |
| 1/18/2011 | 512.53 | 7.98 | 0 | 0 | 0 | 0 | 0 | 0 |
| 1/19/2011 | 379.45 | 7.86 | 0 | 0 | 16 | 0 | 0 | 0 |
| 1/20/2011 | 300.16 | 7.21 | 0 | 0 | 24 | 0 | 0 | 0 |
| 1/21/2011 | 235.03 | 6.80 | 0 | 0 | 60 | 0 | 0 | 0 |
| 1/22/2011 | 196.80 | 7.58 | 0 | 0 | 224 | 0 | 0 | 0 |
| 1/23/2011 | 167.35 | 7.00 | 0 | 0 | 189 | 1 | 0 | 0 |
| 1/24/2011 | 146.40 | 7.09 | 0 | 0 | 142 | 0 | 0 | 0 |
| 1/25/2011 | 130.82 | 6.70 | 0 | 0 | 68 | 0 | 0 | 0 |
| 1/26/2011 | 118.08 | 6.08 | 0 | 0 | 18 | 0 | 0 | 0 |
| 1/27/2011 | 108.45 | 6.21 | 0 | 0 | 22 | 0 | 0 | 0 |
| 1/28/2011 | 102.79 | 6.37 | 0 | 0 | 29 | 0 | 0 | 0 |
| 1/29/2011 | 98.83 | 6.58 | 0 | 0 | 31 | 0 | 0 | 0 |
| 1/30/2011 | 93.45 | 6.96 | 0 | 0 | 77 | 0 | 0 | 0 |
| 1/31/2011 | 87.22 | 6.89 | 0 | 0 | 85 | 0 | 0 | 0 |
| 2/1/2011 | 80.70 | 7.23 | 0 | 0 | 40 | 0 | 0 | 0 |
| 2/2/2011 | 75.89 | 6.19 | 0 | 0 | 3 | 0 | 0 | 0 |
| 2/3/2011 | 71.92 | 5.23 | 0 | 0 | 1 | 0 | 0 | 0 |
| 2/4/2011 | 68.53 | 5.20 | 0 | 0 | 2 | 0 | 0 | 0 |
| 2/5/2011 | 65.70 | 6.53 | 0 | 0 | 10 | 0 | 0 | 0 |
| 2/6/2011 | 67.39 | 7.63 | 0 | 0 | 119 | 0 | 0 | 0 |
| 2/7/2011 | 66.83 | 7.82 | 0 | 0 | 311 | 0 | 0 | 0 |
| 2/8/2011 | 67.96 | 7.23 | 0 | 0 | 60 | 0 | 0 | 0 |
| 2/9/2011 | 66.26 | 6.52 | 0 | 0 | 2 | 0 | 0 | 0 |
| 2/10/2011 | 62.58 | 5.60 | 0 | 0 | 0 | 0 | 0 | 0 |
| 2/11/2011 | 60.03 | 5.03 | 0 | 0 | 0 | 0 | 0 | 0 |
| 2/12/2011 | 58.62 | 5.79 | 0 | 0 | 3 | 0 | 0 | 0 |
| 2/13/2011 | 55.78 | 6.70 | 0 | 0 | 8 | 0 | 0 | 0 |
| 2/14/2011 | 58.05 | 7.01 | 0 | 0 | 8 | 0 | 0 | 0 |
| 2/15/2011 | 198.22 | 6.63 | 0 | 0 | 0 | 0 | 0 | 0 |
| 2/16/2011 | 220.87 | 5.97 | 0 | 0 | 11 | 0 | 0 | 0 |
| 2/17/2011 | 163.67 | 5.63 | 0 | 0 | 0 | 0 | 0 | 0 |
| 2/18/2011 | 132.52 | 6.20 | 0 | 0 | 60 | 0 | 0 | 0 |
| 2/19/2011 | 112.98 | 5.87 | 0 | 0 | 43 | 0 | 0 | 0 |
| 2/20/2011 | 101.09 | 6.13 | 0 | 0 | 54 | 0 | 0 | 0 |
| 2/21/2011 | 91.75 | 6.56 | 0 | 0 | 52 | 0 | 0 | 0 |
| 2/22/2011 | 85.23 | 6.13 | 0 | 0 | 20 | 0 | 0 | 0 |
| 2/23/2011 | 85.23 | 5.79 | 0 | 0 | 4 | 0 | 0 | 0 |
| 2/24/2011 | 88.91 | 5.31 | 0 | 0 | 0 | 0 | 0 | 0 |
| 2/25/2011 | 82.97 | 4.47 | 0 | 0 | 0 | 0 | 0 | 0 |

| Date | Discharge (cms) | Temp (C°) | Chinook | Coho | Steelhead | Cutthroat | Lamprey | Sucker |
|---|---|---|---|---|---|---|---|---|
| 2/26/2011 | 75.04 | 3.42 | 0 | 0 | 0 | 0 | 0 | 0 |
| 2/27/2011 | 70.23 | 4.08 | 0 | 0 | 0 | 0 | 0 | 0 |
| 2/28/2011 | 68.81 | 5.00 | 0 | 0 | 3 | 0 | 0 | 0 |
| 3/1/2011 | 77.59 | 5.76 | 0 | 0 | 1 | 0 | 0 | 0 |
| 3/2/2011 | 111.00 | 6.81 | 0 | 0 | 326 | 0 | 0 | 0 |
| 3/3/2011 | 143.57 | 6.79 | 0 | 0 | 278 | 0 | 0 | 0 |
| 3/4/2011 | 134.79 | 7.08 | 0 | 0 | 252 | 0 | 0 | 0 |
| 3/5/2011 | 305.82 | 7.01 | 0 | 0 | 99 | 0 | 0 | 0 |
| 3/6/2011 | 336.97 | 7.18 | 0 | 0 | 242 | 0 | 0 | 0 |
| 3/7/2011 | 231.91 | 7.53 | 0 | 0 | 305 | 0 | 0 | 0 |
| 3/8/2011 | 222.29 | 7.22 | 0 | 0 | 214 | 0 | 0 | 0 |
| 3/9/2011 | 393.60 | 7.55 | 0 | 0 | 73 | 0 | 0 | 0 |
| 3/10/2011 | 433.25 | 7.76 | 0 | 0 | 232 | 0 | 0 | 0 |
| 3/11/2011 | 453.07 | 7.44 | 0 | 0 | 6 | 0 | 0 | 0 |
| 3/12/2011 | 282.89 | 7.35 | 0 | 0 | 49 | 0 | 0 | 0 |
| 3/13/2011 | 217.47 | 7.82 | 0 | 0 | 494 | 3 | 0 | 0 |
| 3/14/2011 | 233.61 | 7.57 | 0 | 0 | 91 | 0 | 0 | 0 |
| 3/15/2011 | 269.01 | 7.70 | 0 | 0 | 167 | 0 | 0 | 0 |
| 3/16/2011 | 438.91 | 7.41 | 0 | 0 | 1 | 0 | 0 | 0 |
| 3/17/2011 | 370.95 | 6.90 | 0 | 0 | 4 | 0 | 0 | 0 |
| 3/18/2011 | 273.26 | 7.32 | 0 | 0 | 31 | 1 | 0 | 0 |
| 3/19/2011 | 223.99 | 7.27 | 0 | 0 | 40 | 0 | 0 | 0 |
| 3/20/2011 | 183.78 | 7.39 | 0 | 0 | 159 | 0 | 0 | 0 |
| 3/21/2011 | 184.34 | 7.81 | 0 | 0 | 393 | 1 | 0 | 0 |
| 3/22/2011 | 187.46 | 7.77 | 0 | 0 | 48 | 0 | 0 | 0 |
| 3/23/2011 | 166.79 | 7.33 | 0 | 0 | 17 | 0 | 0 | 0 |
| 3/24/2011 | 151.78 | 7.75 | 0 | 0 | 98 | 0 | 0 | 0 |
| 3/25/2011 | 140.17 | 7.71 | 0 | 0 | 145 | 0 | 0 | 0 |
| 3/26/2011 | 148.95 | 7.86 | 0 | 0 | 122 | 0 | 0 | 0 |
| 3/27/2011 | 182.36 | 7.40 | 0 | 0 | 51 | 1 | 0 | 0 |
| 3/28/2011 | 197.93 | 7.79 | 0 | 0 | 105 | 1 | 0 | 0 |
| 3/29/2011 | 217.47 | 7.90 | 0 | 0 | 221 | 0 | 0 | 0 |
| 3/30/2011 | 351.13 | 8.45 | 0 | 0 | 53 | 0 | 0 | 0 |
| 3/31/2011 | 342.63 | 9.11 | 0 | 0 | 237 | 0 | 0 | 6 |
| 4/1/2011 | 302.99 | 9.27 | 0 | 0 | 296 | 1 | 0 | 0 |
| 4/2/2011 | 294.50 | 8.87 | 0 | 0 | 69 | 0 | 0 | 0 |
| 4/3/2011 | 244.94 | 7.72 | 0 | 0 | 4 | 0 | 0 | 0 |
| 4/4/2011 | 197.09 | 7.86 | 0 | 0 | 37 | 0 | 0 | 0 |
| 4/5/2011 | 297.33 | 8.58 | 2 | 0 | 51 | 0 | 0 | 0 |
| 4/6/2011 | 328.48 | 7.62 | 0 | 0 | 3 | 0 | 0 | 0 |
| 4/7/2011 | 300.16 | 6.77 | 0 | 0 | 1 | 0 | 0 | 0 |
| 4/8/2011 | 243.52 | 7.60 | 0 | 0 | 18 | 0 | 0 | 0 |
| 4/9/2011 | 202.18 | 7.83 | 0 | 0 | 27 | 0 | 0 | 1 |
| 4/10/2011 | 175.85 | 8.22 | 0 | 0 | 145 | 0 | 0 | 0 |
| 4/11/2011 | 172.17 | 8.76 | 1 | 0 | 97 | 1 | 0 | 0 |

| Date | Discharge (cms) | Temp (C°) | Chinook | Coho | Steelhead | Cutthroat | Lamprey | Sucker |
|---|---|---|---|---|---|---|---|---|
| 4/12/2011 | 173.58 | 8.43 | 0 | 0 | 51 | 0 | 0 | 0 |
| 4/13/2011 | 155.18 | 8.42 | 0 | 0 | 77 | 0 | 0 | 0 |
| 4/14/2011 | 144.13 | 8.19 | 0 | 0 | 19 | 0 | 0 | 0 |
| 4/15/2011 | 148.95 | 7.96 | 0 | 0 | 37 | 0 | 0 | 0 |
| 4/16/2011 | 319.98 | 8.02 | 1 | 0 | 54 | 0 | 0 | 0 |
| 4/17/2011 | 461.56 | 8.21 | 0 | 0 | 35 | 1 | 0 | 0 |
| 4/18/2011 | 359.62 | 8.54 | 0 | 0 | 33 | 0 | 0 | 0 |
| 4/19/2011 | 264.48 | 8.33 | 0 | 0 | 32 | 0 | 0 | 0 |
| 4/20/2011 | 210.68 | 8.19 | 0 | 0 | 83 | 0 | 0 | 0 |
| 4/21/2011 | 184.06 | 8.67 | 3 | 0 | 91 | 0 | 0 | 0 |
| 4/22/2011 | 157.72 | 8.93 | 1 | 0 | 61 | 0 | 0 | 0 |
| 4/23/2011 | 141.87 | 9.20 | 1 | 0 | 151 | 0 | 0 | 0 |
| 4/24/2011 | 147.53 | 9.04 | 3 | 0 | 75 | 0 | 0 | 1 |
| 4/25/2011 | 179.25 | 8.89 | 12 | 0 | 106 | 0 | 0 | 0 |
| 4/26/2011 | 247.49 | 8.20 | 0 | 0 | 8 | 0 | 0 | 0 |
| 4/27/2011 | 216.34 | 9.09 | 2 | 0 | 29 | 0 | 0 | 0 |
| 4/28/2011 | 187.17 | 9.11 | 12 | 0 | 25 | 0 | 0 | 0 |
| 4/29/2011 | 187.74 | 7.94 | 0 | 0 | 4 | 0 | 0 | 0 |
| 4/30/2011 | 197.93 | 7.96 | 0 | 0 | 1 | 0 | 0 | 0 |
| 5/1/2011 | 182.93 | 9.35 | 1 | 0 | 31 | 0 | 0 | 0 |
| 5/2/2011 | 168.20 | 9.69 | 15 | 0 | 50 | 0 | 0 | 9 |
| 5/3/2011 | 156.31 | 10.34 | 12 | 0 | 33 | 1 | 0 | 6 |
| 5/4/2011 | 141.58 | 11.19 | 23 | 0 | 23 | 0 | 0 | 226 |
| 5/5/2011 | 137.90 | 11.74 | 66 | 0 | 29 | 1 | 0 | 480 |
| 5/6/2011 | 145.27 | 10.57 | 39 | 0 | 22 | 0 | 0 | 163 |
| 5/7/2011 | 150.65 | 9.70 | 27 | 0 | 4 | 0 | 0 | 0 |
| 5/8/2011 | 155.74 | 9.44 | 0 | 0 | 0 | 0 | 0 | 0 |
| 5/9/2011 | 150.36 | 9.93 | 21 | 0 | 7 | 0 | 0 | 0 |
| 5/10/2011 | 137.34 | 10.80 | 20 | 0 | 9 | 0 | 0 | 0 |
| 5/11/2011 | 133.37 | 10.82 | 85 | 0 | 24 | 0 | 0 | 22 |
| 5/12/2011 | 156.03 | 11.43 | 69 | 0 | 9 | 1 | 0 | 32 |
| 5/13/2011 | 157.72 | 11.36 | 39 | 0 | 3 | 0 | 0 | 33 |
| 5/14/2011 | 149.80 | 9.93 | 16 | 0 | 1 | 0 | 0 | 0 |
| 5/15/2011 | 142.72 | 9.25 | 3 | 0 | 0 | 0 | 0 | 0 |
| 5/16/2011 | 142.15 | 9.97 | 17 | 0 | 1 | 0 | 0 | 0 |
| 5/17/2011 | 138.75 | 9.86 | 140 | 0 | 6 | 0 | 0 | 0 |
| 5/18/2011 | 141.58 | 9.96 | 87 | 0 | 7 | 0 | 0 | 0 |
| 5/19/2011 | 133.66 | 11.55 | 65 | 0 | 5 | 0 | 0 | 2 |
| 5/20/2011 | 127.99 | 12.52 | 251 | 0 | 5 | 0 | 0 | 233 |
| 5/21/2011 | 123.18 | 12.39 | 134 | 0 | 7 | 0 | 0 | 561 |
| 5/22/2011 | 120.35 | 11.26 | 32 | 0 | 4 | 0 | 0 | 162 |
| 5/23/2011 | 113.83 | 10.63 | 22 | 0 | 0 | 0 | 0 | 0 |
| 5/24/2011 | 108.74 | 11.06 | 27 | 0 | 1 | 0 | 0 | 0 |
| 5/25/2011 | 111.85 | 11.20 | 28 | 0 | 0 | 0 | 0 | 15 |
| 5/26/2011 | 137.62 | 10.00 | 47 | 0 | 4 | 0 | 0 | 1 |

| Date | Discharge (cms) | Temp (C°) | Chinook | Coho | Steelhead | Cutthroat | Lamprey | Sucker |
|---|---|---|---|---|---|---|---|---|
| 5/27/2011 | 147.81 | 9.73 | 17 | 0 | 2 | 0 | 0 | 0 |
| 5/28/2011 | 206.71 | 9.23 | 2 | 0 | 1 | 0 | 0 | 0 |
| 5/29/2011 | 192.84 | 8.93 | 0 | 0 | 0 | 0 | 0 | 0 |
| 5/30/2011 | 169.05 | 8.97 | 0 | 0 | 0 | 0 | 0 | 0 |
| 5/31/2011 | 181.23 | 9.47 | 11 | 0 | 0 | 0 | 0 | 0 |
| 6/1/2011 | 236.45 | 9.17 | 23 | 0 | 0 | 0 | 0 | 0 |
| 6/2/2011 | 253.72 | 9.39 | 28 | 0 | 6 | 0 | 0 | 0 |
| 6/3/2011 | 214.36 | 11.21 | 339 | 0 | 2 | 0 | 0 | 0 |
| 6/4/2011 | 180.38 | 10.94 | 468 | 0 | 10 | 0 | 0 | 4 |
| 6/5/2011 | 157.72 | 11.58 | 370 | 0 | 8 | 0 | 0 | 19 |
| 6/6/2011 | 150.36 | 11.43 | 85 | 0 | 1 | 0 | 0 | 2 |
| 6/7/2011 | 141.02 | 11.02 | 75 | 0 | 1 | 1 | 0 | 9 |
| 6/8/2011 | 132.81 | 13.93 | 57 | 0 | 3 | 0 | 0 | 103 |
| 6/9/2011 | 126.58 | 13.79 | 400 | 0 | 7 | 0 | 0 | 889 |
| 6/10/2011 | 123.46 | 13.03 | 423 | 0 | 12 | 0 | 0 | 328 |
| 6/11/2011 | 122.61 | 13.72 | 167 | 0 | 15 | 0 | 0 | 204 |
| 6/12/2011 | 117.80 | 12.88 | 180 | 0 | 9 | 2 | 0 | 60 |
| 6/13/2011 | 125.73 | 11.99 | 17 | 0 | 2 | 0 | 0 | 0 |
| 6/14/2011 | 126.58 | 13.01 | 96 | 0 | 2 | 0 | 1 | 27 |
| 6/15/2011 | 120.06 | 15.02 | 150 | 0 | 16 | 0 | 0 | 42 |
| 6/16/2011 | 113.27 | 13.84 | 178 | 0 | 19 | 2 | 0 | 48 |
| 6/17/2011 | 107.32 | 14.08 | 342 | 0 | 41 | 1 | 0 | 42 |
| 6/18/2011 | 101.09 | 13.41 | 321 | 0 | 51 | 1 | 0 | 26 |
| 6/19/2011 | 99.68 | 13.97 | 81 | 0 | 30 | 0 | 0 | 15 |
| 6/20/2011 | 101.09 | 13.70 | 115 | 0 | 12 | 1 | 0 | 316 |
| 6/21/2011 | 101.37 | 17.28 | 105 | 0 | 55 | 0 | 1 | 191 |
| 6/22/2011 | 103.07 | 16.50 | 401 | 0 | 118 | 2 | 3 | 336 |
| 6/23/2011 | 105.34 | 15.62 | 231 | 0 | 51 | 3 | 4 | 254 |
| 6/24/2011 | 99.96 | 15.23 | 71 | 0 | 31 | 5 | 0 | 138 |
| 6/25/2011 | 92.03 | 14.82 | 83 | 0 | 22 | 2 | 1 | 79 |
| 6/26/2011 | 86.65 | 15.11 | 81 | 0 | 34 | 0 | 1 | 84 |
| 6/27/2011 | 83.53 | 15.47 | 52 | 0 | 14 | 1 | 2 | 55 |
| 6/28/2011 | 87.50 | 16.04 | 82 | 0 | 48 | 1 | 0 | 260 |
| 6/29/2011 | 95.14 | 15.01 | 97 | 0 | 139 | 1 | 0 | 58 |
| 6/30/2011 | 89.48 | 14.82 | 48 | 0 | 53 | 0 | 0 | 16 |
| 7/1/2011 | 85.52 | 15.27 | 48 | 0 | 44 | 1 | 0 | 52 |
| 7/2/2011 | 80.99 | 16.18 | 101 | 0 | 47 | 1 | 4 | 159 |
| 7/3/2011 | 77.59 | 16.82 | 131 | 0 | 59 | 0 | 5 | 118 |
| 7/4/2011 | 76.74 | 17.59 | 32 | 0 | 33 | 3 | 3 | 113 |
| 7/5/2011 | 74.76 | 17.80 | 32 | 0 | 39 | 5 | 1 | 450 |
| 7/6/2011 | 73.34 | 18.26 | 69 | 0 | 77 | 4 | 1 | 361 |
| 7/7/2011 | 69.66 | 18.68 | 83 | 0 | 109 | 4 | 1 | 282 |
| 7/8/2011 | 67.96 | 18.39 | 45 | 0 | 87 | 1 | 2 | 80 |
| 7/9/2011 | 65.13 | 18.10 | 49 | 0 | 57 | 1 | 0 | 79 |
| 7/10/2011 | 60.88 | 17.91 | 76 | 0 | 75 | 1 | 0 | 76 |

| Date | Discharge (cms) | Temp (C°) | Chinook | Coho | Steelhead | Cutthroat | Lamprey | Sucker |
|---|---|---|---|---|---|---|---|---|
| 7/11/2011 | 58.33 | 17.80 | 34 | 0 | 48 | 0 | 0 | 1 |
| 7/12/2011 | 56.92 | 17.58 | 0 | 0 | 0 | 1 | 0 | 0 |
| 7/13/2011 | 55.78 | 16.95 | 21 | 0 | 5 | 0 | 0 | 155 |
| 7/14/2011 | 55.78 | 17.00 | 17 | 0 | 100 | 0 | 0 | 202 |
| 7/15/2011 | 52.67 | 17.78 | 50 | 0 | 113 | 2 | 0 | 1940 |
| 7/16/2011 | 51.54 | 18.10 | 53 | 0 | 98 | 3 | 0 | 846 |
| 7/17/2011 | 52.39 | 17.22 | 33 | 0 | 62 | 3 | 0 | 101 |
| 7/18/2011 | 52.95 | 16.48 | 3 | 0 | 25 | 1 | 0 | 1 |
| 7/19/2011 | 79.57 | 16.45 | 0 | 0 | 0 | 1 | 0 | 0 |
| 7/20/2011 | 90.61 | 15.09 | 55 | 0 | 16 | 0 | 0 | 1442 |
| 7/21/2011 | 68.53 | 16.84 | 37 | 0 | 197 | 3 | 0 | 451 |
| 7/22/2011 | 60.88 | 17.68 | 62 | 0 | 115 | 5 | 1 | 638 |
| 7/23/2011 | 56.92 | 18.52 | 27 | 0 | 35 | 1 | 1 | 153 |
| 7/24/2011 | 54.09 | 19.37 | 62 | 0 | 79 | 15 | 2 | 137 |
| 7/25/2011 | 50.40 | 19.87 | 34 | 0 | 38 | 5 | 5 | 0 |
| 7/26/2011 | 48.99 | 19.24 | 0 | 0 | 0 | 2 | 0 | 0 |
| 7/27/2011 | 48.14 | 19.01 | 52 | 0 | 21 | 9 | 0 | 14 |
| 7/28/2011 | 47.01 | 19.27 | 33 | 0 | 105 | 17 | 0 | 3 |
| 7/29/2011 | 45.31 | 19.48 | 52 | 0 | 97 | 41 | 1 | 12 |
| 7/30/2011 | 44.74 | 20.08 | 55 | 0 | 47 | 40 | 3 | 21 |
| 7/31/2011 | 44.17 | 20.43 | 63 | 0 | 46 | 33 | 2 | 9 |
| 8/1/2011 | 43.32 | 20.96 | 46 | 0 | 45 | 21 | 1 | 16 |
| 8/2/2011 | 42.19 | 20.76 | 23 | 0 | 26 | 28 | 4 | 4 |
| 8/3/2011 | 41.91 | 20.24 | 18 | 0 | 14 | 16 | 2 | 0 |
| 8/4/2011 | 41.06 | 20.01 | 16 | 0 | 19 | 12 | 0 | 1 |
| 8/5/2011 | 40.21 | 19.33 | 2 | 0 | 13 | 7 | 0 | 0 |
| 8/6/2011 | 39.36 | 19.42 | 1 | 0 | 9 | 7 | 0 | 2 |
| 8/7/2011 | 38.79 | 19.74 | 7 | 0 | 9 | 2 | 0 | 2 |
| 8/8/2011 | 38.51 | 19.23 | 12 | 0 | 15 | 4 | 1 | 1 |
| 8/9/2011 | 38.51 | 18.56 | 5 | 0 | 10 | 1 | 0 | 0 |
| 8/10/2011 | 37.66 | 19.11 | 11 | 0 | 14 | 5 | 0 | 0 |
| 8/11/2011 | 36.81 | 19.58 | 18 | 0 | 15 | 5 | 0 | 0 |
| 8/12/2011 | 35.96 | 19.67 | 17 | 0 | 39 | 6 | 0 | 0 |
| 8/13/2011 | 35.68 | 19.25 | 18 | 0 | 41 | 6 | 0 | 1 |
| 8/14/2011 | 35.68 | 18.93 | 16 | 0 | 25 | 2 | 0 | 1 |
| 8/15/2011 | 35.40 | 19.43 | 22 | 0 | 48 | 2 | 0 | 0 |
| 8/16/2011 | 35.40 | 19.70 | 23 | 0 | 36 | 5 | 0 | 0 |
| 8/17/2011 | 35.11 | 19.69 | 35 | 0 | 32 | 2 | 0 | 0 |
| 8/18/2011 | 34.26 | 19.50 | 17 | 0 | 22 | 4 | 0 | 0 |
| 8/19/2011 | 34.26 | 19.54 | 12 | 0 | 30 | 1 | 0 | 0 |
| 8/20/2011 | 33.70 | 19.68 | 18 | 0 | 27 | 5 | 0 | 0 |
| 8/21/2011 | 33.70 | 20.06 | 26 | 0 | 20 | 3 | 1 | 0 |
| 8/22/2011 | 33.41 | 20.24 | 25 | 1 | 27 | 2 | 0 | 0 |
| 8/23/2011 | 33.13 | 20.53 | 25 | 0 | 22 | 3 | 0 | 0 |
| 8/24/2011 | 33.13 | 20.79 | 22 | 0 | 20 | 3 | 0 | 0 |

| Date | Discharge (cms) | Temp (C°) | Chinook | Coho | Steelhead | Cutthroat | Lamprey | Sucker |
|---|---|---|---|---|---|---|---|---|
| 8/25/2011 | 33.13 | 21.04 | 18 | 0 | 16 | 5 | 0 | 0 |
| 8/26/2011 | 33.13 | 21.62 | 12 | 0 | 12 | 6 | 1 | 0 |
| 8/27/2011 | 32.85 | 21.47 | 17 | 0 | 10 | 4 | 1 | 0 |
| 8/28/2011 | 32.56 | 21.11 | 9 | 0 | 9 | 1 | 0 | 0 |
| 8/29/2011 | 32.28 | 20.14 | 2 | 0 | 10 | 0 | 1 | 0 |
| 8/30/2011 | 32.00 | 18.83 | 2 | 0 | 2 | 0 | 0 | 0 |
| 8/31/2011 | 31.71 | 18.03 | 0 | 0 | 2 | 1 | 0 | 0 |
| 9/1/2011 | 31.15 | 17.60 | 0 | 0 | 2 | 0 | 0 | 0 |
| 9/2/2011 | 31.15 | 18.01 | 5 | 0 | 5 | 0 | 0 | 0 |
| 9/3/2011 | 31.15 | 17.76 | 15 | 0 | 4 | 0 | 0 | 0 |
| 9/4/2011 | 31.43 | 17.99 | 32 | 0 | 19 | 0 | 0 | 0 |
| 9/5/2011 | 32.00 | 18.51 | 55 | 1 | 16 | 1 | 0 | 0 |
| 9/6/2011 | 32.00 | 18.67 | 49 | 1 | 23 | 0 | 0 | 0 |
| 9/7/2011 | 31.71 | 18.84 | 34 | 0 | 12 | 1 | 0 | 0 |
| 9/8/2011 | 31.71 | 19.10 | 39 | 2 | 12 | 1 | 0 | 0 |
| 9/9/2011 | 32.28 | 19.39 | 39 | 2 | 9 | 2 | 0 | 0 |
| 9/10/2011 | 32.28 | 19.20 | 32 | 6 | 16 | 0 | 0 | 0 |
| 9/11/2011 | 32.00 | 19.33 | 45 | 0 | 10 | 2 | 0 | 0 |
| 9/12/2011 | 32.00 | 18.85 | 22 | 2 | 12 | 0 | 0 | 0 |
| 9/13/2011 | 32.00 | 18.13 | 11 | 3 | 4 | 0 | 0 | 0 |
| 9/14/2011 | 32.56 | 17.65 | 6 | 2 | 1 | 1 | 0 | 0 |
| 9/15/2011 | 32.28 | 17.23 | 4 | 3 | 1 | 0 | 0 | 0 |
| 9/16/2011 | 32.00 | 16.73 | 0 | 3 | 4 | 1 | 0 | 0 |
| 9/17/2011 | 32.28 | 16.50 | 3 | 5 | 4 | 0 | 0 | 0 |
| 9/18/2011 | 31.43 | 16.23 | 0 | 4 | 2 | 0 | 0 | 0 |
| 9/19/2011 | 31.15 | 16.60 | 10 | 3 | 11 | 1 | 0 | 0 |
| 9/20/2011 | 30.87 | 16.89 | 5 | 7 | 13 | 1 | 0 | 0 |
| 9/21/2011 | 30.87 | 16.90 | 11 | 12 | 6 | 2 | 0 | 0 |
| 9/22/2011 | 30.87 | 16.67 | 13 | 22 | 9 | 0 | 0 | 0 |
| 9/23/2011 | 30.87 | 16.63 | 7 | 16 | 15 | 1 | 0 | 0 |
| 9/24/2011 | 30.58 | 17.13 | 20 | 24 | 2 | 0 | 0 | 0 |
| 9/25/2011 | 30.30 | 16.59 | 18 | 19 | 8 | 0 | 0 | 0 |
| 9/26/2011 | 30.30 | 15.50 | 4 | 1 | 0 | 0 | 0 | 0 |
| 9/27/2011 | 30.87 | 15.76 | 0 | 0 | 0 | 0 | 0 | 0 |
| 9/28/2011 | 30.87 | 14.85 | 0 | 0 | 0 | 0 | 0 | 0 |
| 9/29/2011 | 30.87 | 14.82 | 0 | 0 | 0 | 0 | 0 | 0 |
| 9/30/2011 | 30.87 | 15.74 | 21 | 25 | 17 | 0 | 0 | 0 |
| 10/1/2011 | 30.58 | 15.44 | 21 | 55 | 18 | 2 | 0 | 0 |
| 10/2/2011 | 30.58 | 15.09 | 13 | 58 | 11 | 0 | 0 | 0 |
| 10/3/2011 | 31.43 | 14.68 | 11 | 20 | 4 | 1 | 0 | 0 |
| 10/4/2011 | 33.98 | 13.92 | 0 | 0 | 0 | 0 | 0 | 0 |
| 10/5/2011 | 36.81 | 13.04 | 0 | 0 | 0 | 0 | 0 | 0 |
| 10/6/2011 | 37.38 | 12.19 | 0 | 0 | 0 | 0 | 0 | 0 |
| 10/7/2011 | 34.26 | 12.03 | 16 | 232 | 36 | 0 | 0 | 0 |
| 10/8/2011 | 33.13 | 12.53 | 10 | 101 | 9 | 2 | 0 | 0 |

| Date | Discharge (cms) | Temp (C°) | Chinook | Coho | Steelhead | Cutthroat | Lamprey | Sucker |
|---|---|---|---|---|---|---|---|---|
| 10/9/2011 | 32.28 | 12.92 | 9 | 124 | 10 | 1 | 0 | 0 |
| 10/10/2011 | 32.56 | 13.25 | 5 | 35 | 3 | 0 | 0 | 0 |
| 10/11/2011 | 41.06 | 12.84 | 0 | 0 | 0 | 0 | 0 | 0 |
| 10/12/2011 | 44.17 | 12.35 | 3 | 5 | 7 | 0 | 0 | 0 |
| 10/13/2011 | 37.38 | 12.33 | 0 | 70 | 3 | 0 | 0 | 2 |
| 10/14/2011 | 34.26 | 12.70 | 7 | 272 | 28 | 0 | 0 | 0 |
| 10/15/2011 | 33.98 | 12.75 | 10 | 257 | 10 | 1 | 0 | 0 |
| 10/16/2011 | 33.41 | 12.10 | 3 | 87 | 6 | 0 | 0 | 0 |
| 10/17/2011 | 32.85 | 11.92 | 2 | 19 | 3 | 0 | 0 | 0 |
| 10/18/2011 | 32.28 | 12.21 | 2 | 3 | 12 | 0 | 0 | 0 |
| 10/19/2011 | 32.85 | 12.22 | 0 | 0 | 0 | 0 | 0 | 0 |
| 10/20/2011 | 32.85 | 10.75 | 0 | 20 | 2 | 0 | 0 | 0 |
| 10/21/2011 | 31.43 | 11.58 | 0 | 30 | 3 | 0 | 0 | 0 |
| 10/22/2011 | 31.15 | 12.03 | 0 | 0 | 0 | 0 | 0 | 0 |
| 10/23/2011 | 30.58 | 11.60 | 6 | 139 | 14 | 1 | 0 | 0 |
| 10/24/2011 | 30.30 | 11.42 | 2 | 20 | 2 | 0 | 0 | 0 |
| 10/25/2011 | 30.30 | 10.28 | 0 | 0 | 0 | 0 | 0 | 0 |
| 10/26/2011 | 30.02 | 9.42 | 0 | 0 | 0 | 0 | 0 | 0 |
| 10/27/2011 | 29.73 | 8.89 | 0 | 0 | 0 | 0 | 0 | 0 |
| 10/28/2011 | 29.73 | 8.65 | 0 | 2 | 3 | 0 | 0 | 0 |
| 10/29/2011 | 30.02 | 8.97 | 0 | 0 | 0 | 0 | 0 | 0 |
| 10/30/2011 | 30.87 | 9.63 | 1 | 17 | 8 | 0 | 0 | 0 |
| 10/31/2011 | 31.15 | 9.97 | 1 | 5 | 2 | 0 | 0 | 0 |
| 11/1/2011 | 32.28 | 9.76 | 0 | 0 | 0 | 0 | 0 | 0 |
| 11/2/2011 | 31.43 | 9.08 | 0 | 0 | 0 | 0 | 0 | 0 |
| 11/3/2011 | 31.43 | 8.79 | 0 | 14 | 4 | 0 | 0 | 0 |
| 11/4/2011 | 31.15 | 8.03 | 0 | 3 | 2 | 0 | 0 | 0 |
| 11/5/2011 | 30.30 | 7.76 | 0 | 4 | 1 | 0 | 0 | 0 |
| 11/6/2011 | 30.58 | 7.75 | 0 | 5 | 0 | 0 | 0 | 0 |
| 11/7/2011 | 31.43 | 7.84 | 0 | 2 | 0 | 0 | 0 | 0 |
| 11/8/2011 | 30.87 | 7.53 | 0 | 0 | 0 | 0 | 0 | 0 |
| 11/9/2011 | 30.30 | 7.32 | 0 | 3 | 0 | 0 | 0 | 0 |
| 11/10/2011 | 29.45 | 7.06 | 0 | 1 | 0 | 0 | 0 | 0 |
| 11/11/2011 | 29.45 | 6.88 | 0 | 3 | 0 | 0 | 0 | 0 |
| 11/12/2011 | 29.17 | 7.07 | 1 | 4 | 1 | 0 | 0 | 0 |
| 11/13/2011 | 29.45 | 7.30 | 0 | 5 | 0 | 0 | 0 | 0 |
| 11/14/2011 | 29.45 | 7.44 | 0 | 0 | 0 | 0 | 0 | 0 |
| 11/15/2011 | 29.73 | 7.80 | 0 | 0 | 2 | 0 | 0 | 0 |
| 11/16/2011 | 30.87 | 8.10 | 0 | 0 | 0 | 0 | 0 | 0 |
| 11/17/2011 | 35.68 | 8.35 | 0 | 27 | 19 | 0 | 0 | 0 |
| 11/18/2011 | 83.53 | 7.76 | 0 | 0 | 0 | 1 | 0 | 0 |
| 11/19/2011 | 64.00 | 6.96 | 2 | 935 | 50 | 0 | 0 | 0 |
| 11/20/2011 | 47.29 | 6.20 | 0 | 226 | 1 | 0 | 0 | 0 |
| 11/21/2011 | 41.63 | 5.66 | 0 | 28 | 0 | 0 | 0 | 0 |
| 11/22/2011 | 92.60 | 6.39 | 0 | 0 | 0 | 0 | 0 | 0 |

| Date | Discharge (cms) | Temp (C°) | Chinook | Coho | Steelhead | Cutthroat | Lamprey | Sucker |
|---|---|---|---|---|---|---|---|---|
| 11/23/2011 | 160.84 | 7.47 | 0 | 258 | 13 | 0 | 0 | 0 |
| 11/24/2011 | 144.13 | 7.72 | 0 | 1438 | 76 | 0 | 0 | 0 |
| 11/25/2011 | 122.05 | 7.33 | 0 | 1022 | 80 | 1 | 0 | 0 |
| 11/26/2011 | 108.74 | 7.36 | 0 | 296 | 46 | 0 | 0 | 0 |
| 11/27/2011 | 77.30 | 7.30 | 0 | 84 | 5 | 1 | 0 | 0 |
| 11/28/2011 | 68.81 | 7.75 | 1 | 19 | 53 | 0 | 0 | 0 |
| 11/29/2011 | 61.73 | 7.94 | 0 | 0 | 0 | 0 | 0 | 0 |
| 11/30/2011 | 56.07 | 7.92 | 0 | 42 | 8 | 0 | 0 | 0 |
| 12/1/2011 | 51.54 | 7.26 | 0 | 0 | 0 | 0 | 0 | 0 |
| 12/2/2011 | 46.72 | 6.14 | 0 | 18 | 0 | 0 | 0 | 0 |
| 12/3/2011 | 43.89 | 5.12 | 0 | 1 | 0 | 0 | 0 | 0 |
| 12/4/2011 | 41.91 | 5.05 | 0 | 0 | 1 | 0 | 0 | 0 |
| 12/5/2011 | 39.93 | 4.59 | 0 | 0 | 0 | 0 | 0 | 0 |
| 12/6/2011 | 38.23 | 3.56 | 0 | 0 | 0 | 0 | 0 | 0 |
| 12/7/2011 | 35.68 | 2.53 | 0 | 0 | 0 | 0 | 0 | 0 |
| 12/8/2011 | 34.55 | 2.60 | 0 | 0 | 0 | 0 | 0 | 0 |
| 12/9/2011 | 33.98 | 2.91 | 0 | 0 | 0 | 0 | 0 | 0 |
| 12/10/2011 | 33.41 | 3.03 | 0 | 1 | 0 | 0 | 0 | 0 |
| 12/11/2011 | 33.13 | 3.08 | 0 | 0 | 0 | 0 | 0 | 0 |
| 12/12/2011 | 32.56 | 3.26 | 0 | 0 | 0 | 0 | 0 | 0 |
| 12/13/2011 | 32.28 | 2.57 | 0 | 0 | 0 | 0 | 0 | 0 |
| 12/14/2011 | 32.28 | 2.05 | 0 | 0 | 0 | 0 | 0 | 0 |
| 12/15/2011 | 32.28 | 2.44 | 0 | 0 | 0 | 0 | 0 | 0 |
| 12/16/2011 | 31.71 | 3.24 | 0 | 0 | 0 | 0 | 0 | 0 |
| 12/17/2011 | 31.71 | 3.65 | 0 | 2 | 1 | 0 | 0 | 0 |
| 12/18/2011 | 31.43 | 3.85 | 0 | 1 | 0 | 0 | 0 | 0 |
| 12/19/2011 | 31.43 | 4.12 | 0 | 1 | 0 | 0 | 0 | 0 |
| 12/20/2011 | 31.15 | 4.87 | 0 | 3 | 1 | 1 | 0 | 0 |
| 12/21/2011 | 31.15 | 4.89 | 0 | 13 | 4 | 0 | 0 | 0 |
| 12/22/2011 | 31.15 | 3.51 | 0 | 2 | 0 | 0 | 0 | 0 |
| 12/23/2011 | 30.02 | 2.56 | 0 | 0 | 0 | 0 | 0 | 0 |
| 12/24/2011 | 29.73 | 1.85 | 0 | 0 | 0 | 0 | 0 | 0 |
| 12/25/2011 | 29.17 | 2.04 | 0 | 0 | 0 | 0 | 0 | 0 |
| 12/26/2011 | 29.73 | 3.27 | 0 | 1 | 0 | 0 | 0 | 0 |
| 12/27/2011 | 30.58 | 4.19 | 0 | 1 | 0 | 0 | 0 | 0 |
| 12/28/2011 | 35.96 | 5.87 | 0 | 29 | 7 | 0 | 0 | 0 |
| 12/29/2011 | 170.18 | 6.63 | 0 | 113 | 55 | 0 | 0 | 0 |
| 12/30/2011 | 450.24 | 7.78 | 0 | 17 | 16 | 0 | 0 | 0 |
| 12/31/2011 | 368.12 | 7.13 | 0 | 0 | 0 | 0 | 0 | 0 |
| 1/1/2012 | 181.79 | 6.03 | 0 | 12 | 24 | 0 | 0 | 0 |
| 1/2/2012 | 128.28 | 6.19 | 0 | 15 | 104 | 0 | 0 | 0 |
| 1/3/2012 | 100.81 | 6.32 | 0 | 12 | 99 | 1 | 0 | 0 |
| 1/4/2012 | 82.12 | 6.14 | 0 | 4 | 87 | 0 | 0 | 0 |
| 1/5/2012 | 72.21 | 6.32 | 0 | 1 | 192 | 0 | 0 | 0 |
| 1/6/2012 | 67.96 | 6.27 | 0 | 0 | 96 | 1 | 0 | 0 |

| Date | Discharge (cms) | Temp (C°) | Chinook | Coho | Steelhead | Cutthroat | Lamprey | Sucker |
|---|---|---|---|---|---|---|---|---|
| 1/7/2012 | 62.58 | 6.50 | 0 | 0 | 166 | 1 | 0 | 0 |
| 1/8/2012 | 58.62 | 5.62 | 0 | 0 | 6 | 0 | 0 | 0 |
| 1/9/2012 | 54.37 | 4.87 | 0 | 0 | 5 | 0 | 0 | 0 |
| 1/10/2012 | 51.25 | 5.05 | 0 | 0 | 5 | 0 | 0 | 0 |
| 1/11/2012 | 48.99 | 5.11 | 0 | 0 | 8 | 0 | 0 | 0 |
| 1/12/2012 | 47.01 | 4.29 | 0 | 0 | 0 | 0 | 0 | 0 |
| 1/13/2012 | 45.02 | 3.34 | 0 | 0 | 0 | 0 | 0 | 0 |
| 1/14/2012 | 43.89 | 2.69 | 0 | 0 | 0 | 0 | 0 | 0 |
| 1/15/2012 | 43.32 | 3.22 | 0 | 0 | 0 | 0 | 0 | 0 |
| 1/16/2012 | 43.04 | 3.58 | 0 | 0 | 0 | 0 | 0 | 0 |
| 1/17/2012 | 41.91 | 3.97 | 0 | 0 | 0 | 0 | 0 | 0 |
| 1/18/2012 | 78.44 | 4.65 | 0 | 0 | 29 | 0 | 0 | 0 |
| 1/19/2012 | 1107.19 | 6.76 | 0 | 0 | 0 | 0 | 0 | 0 |
| 1/20/2012 | 832.52 | 7.61 | 0 | 0 | 0 | 0 | 0 | 0 |
| 1/21/2012 | 620.14 | 7.56 | 0 | 0 | 0 | 0 | 0 | 0 |
| 1/22/2012 | 399.27 | 6.61 | 0 | 0 | 6 | 0 | 0 | 0 |
| 1/23/2012 | 286.00 | 6.71 | 0 | 0 | 38 | 0 | 0 | 0 |
| 1/24/2012 | 223.42 | 6.78 | 0 | 0 | 264 | 0 | 0 | 0 |
| 1/25/2012 | 455.90 | 7.13 | 0 | 0 | 163 | 0 | 0 | 0 |
| 1/26/2012 | 492.71 | 7.97 | 0 | 0 | 217 | 0 | 0 | 0 |
| 1/27/2012 | 424.75 | 6.72 | 0 | 0 | 9 | 0 | 0 | 0 |
| 1/28/2012 | 267.59 | 5.57 | 0 | 0 | 4 | 0 | 0 | 0 |
| 1/29/2012 | 199.92 | 5.59 | 0 | 0 | 56 | 0 | 0 | 0 |
| 1/30/2012 | 209.26 | 6.91 | 0 | 0 | 227 | 0 | 0 | 0 |
| 1/31/2012 | 204.45 | 7.16 | 0 | 0 | 463 | 0 | 0 | 0 |
| 2/1/2012 | 175.00 | 7.10 | 0 | 0 | 235 | 0 | 0 | 0 |
| 2/2/2012 | 166.22 | 6.78 | 0 | 0 | 138 | 0 | 0 | 0 |
| 2/3/2012 | 144.13 | 6.05 | 0 | 0 | 26 | 0 | 0 | 0 |
| 2/4/2012 | 123.74 | 5.63 | 0 | 0 | 15 | 0 | 0 | 0 |
| 2/5/2012 | 109.59 | 5.43 | 0 | 0 | 21 | 0 | 0 | 0 |
| 2/6/2012 | 97.13 | 5.10 | 0 | 0 | 4 | 0 | 0 | 0 |
| 2/7/2012 | 89.76 | 5.30 | 0 | 0 | 27 | 0 | 0 | 0 |
| 2/8/2012 | 85.80 | 5.99 | 0 | 0 | 167 | 0 | 0 | 0 |
| 2/9/2012 | 92.88 | 7.20 | 0 | 0 | 544 | 0 | 0 | 0 |
| 2/10/2012 | 94.30 | 7.93 | 0 | 0 | 538 | 1 | 0 | 0 |
| 2/11/2012 | 96.28 | 8.13 | 0 | 0 | 284 | 0 | 0 | 0 |
| 2/12/2012 | 95.43 | 7.23 | 0 | 0 | 71 | 0 | 0 | 0 |
| 2/13/2012 | 90.61 | 6.83 | 0 | 0 | 17 | 0 | 0 | 0 |
| 2/14/2012 | 84.95 | 6.64 | 0 | 0 | 24 | 0 | 0 | 0 |
| 2/15/2012 | 82.69 | 6.19 | 0 | 0 | 3 | 0 | 0 | 0 |
| 2/16/2012 | 77.02 | 5.58 | 0 | 0 | 1 | 0 | 0 | 0 |
| 2/17/2012 | 73.91 | 6.26 | 0 | 0 | 7 | 0 | 0 | 0 |
| 2/18/2012 | 93.73 | 7.03 | 0 | 0 | 159 | 0 | 0 | 0 |
| 2/19/2012 | 111.57 | 6.64 | 0 | 0 | 104 | 0 | 0 | 0 |
| 2/20/2012 | 116.67 | 6.16 | 0 | 0 | 71 | 0 | 0 | 0 |

| Date | Discharge (cms) | Temp (C°) | Chinook | Coho | Steelhead | Cutthroat | Lamprey | Sucker |
|---|---|---|---|---|---|---|---|---|
| 2/21/2012 | 247.21 | 6.58 | 0 | 0 | 38 | 0 | 0 | 0 |
| 2/22/2012 | 271.28 | 7.89 | 0 | 0 | 186 | 0 | 0 | 0 |
| 2/23/2012 | 297.33 | 7.54 | 0 | 0 | 93 | 0 | 0 | 0 |
| 2/24/2012 | 216.62 | 7.11 | 0 | 0 | 172 | 0 | 0 | 0 |
| 2/25/2012 | 203.88 | 6.79 | 0 | 0 | 102 | 0 | 0 | 0 |
| 2/26/2012 | 180.38 | 5.71 | 0 | 0 | 4 | 0 | 0 | 0 |
| 2/27/2012 | 153.48 | 5.56 | 0 | 0 | 10 | 0 | 0 | 0 |
| 2/28/2012 | 139.32 | 5.53 | 0 | 0 | 17 | 0 | 0 | 0 |
| 2/29/2012 | 162.54 | 5.41 | 0 | 0 | 5 | 0 | 0 | 0 |
| 3/1/2012 | 153.76 | 5.53 | 0 | 0 | 3 | 0 | 0 | 0 |
| 3/2/2012 | 137.05 | 6.16 | 0 | 0 | 54 | 0 | 0 | 0 |
| 3/3/2012 | 123.46 | 7.03 | 0 | 0 | 201 | 0 | 0 | 0 |
| 3/4/2012 | 143.00 | 7.12 | 0 | 0 | 199 | 0 | 0 | 0 |
| 3/5/2012 | 180.38 | 6.60 | 0 | 0 | 149 | 0 | 0 | 0 |
| 3/6/2012 | 215.77 | 6.33 | 0 | 0 | 62 | 0 | 0 | 0 |
| 3/7/2012 | 175.28 | 6.33 | 0 | 0 | 25 | 0 | 0 | 0 |
| 3/8/2012 | 144.98 | 6.68 | 0 | 0 | 68 | 0 | 0 | 0 |
| 3/9/2012 | 149.80 | 7.43 | 0 | 0 | 220 | 0 | 0 | 0 |
| 3/10/2012 | 166.79 | 7.46 | 0 | 0 | 475 | 0 | 0 | 0 |
| 3/11/2012 | 173.58 | 7.71 | 0 | 0 | 484 | 0 | 0 | 0 |
| 3/12/2012 | 177.26 | 6.77 | 0 | 0 | 51 | 0 | 0 | 0 |
| 3/13/2012 | 190.29 | 6.74 | 0 | 0 | 53 | 0 | 0 | 0 |
| 3/14/2012 | 189.72 | 6.64 | 0 | 0 | 62 | 0 | 0 | 0 |
| 3/15/2012 | 258.25 | 7.14 | 0 | 0 | 224 | 0 | 0 | 0 |
| 3/16/2012 | 682.44 | 7.38 | 0 | 0 | 6 | 0 | 0 | 0 |
| 3/17/2012 | 470.06 | 7.41 | 0 | 0 | 43 | 0 | 0 | 0 |
| 3/18/2012 | 308.65 | 6.83 | 0 | 0 | 23 | 0 | 0 | 0 |
| 3/19/2012 | 230.22 | 5.98 | 0 | 0 | 12 | 0 | 0 | 0 |
| 3/20/2012 | 277.22 | 6.09 | 0 | 0 | 3 | 0 | 0 | 0 |
| 3/21/2012 | 770.22 | 7.02 | 0 | 0 | 3 | 0 | 0 | 0 |
| 3/22/2012 | 1030.73 | 7.24 | 0 | 0 | 0 | 0 | 0 | 0 |
| 3/23/2012 | 506.87 | 7.37 | 0 | 0 | 6 | 0 | 0 | 0 |
| 3/24/2012 | 331.31 | 7.45 | 0 | 0 | 79 | 0 | 0 | 0 |
| 3/25/2012 | 257.68 | 7.50 | 0 | 0 | 368 | 0 | 0 | 0 |
| 3/26/2012 | 220.59 | 7.68 | 0 | 0 | 443 | 2 | 0 | 0 |
| 3/27/2012 | 188.59 | 7.98 | 0 | 0 | 626 | 0 | 0 | 0 |
| 3/28/2012 | 189.44 | 8.20 | 0 | 0 | 421 | 0 | 0 | 0 |
| 3/29/2012 | 182.08 | 8.37 | 0 | 0 | 417 | 0 | 0 | 0 |
| 3/30/2012 | 642.79 | 8.15 | 0 | 0 | 47 | 0 | 0 | 0 |
| 3/31/2012 | 841.01 | 7.78 | 0 | 0 | 0 | 0 | 0 | 0 |
| 4/1/2012 | 549.35 | 7.41 | 0 | 0 | 1 | 0 | 0 | 0 |
| 4/2/2012 | 382.28 | 7.30 | 0 | 0 | 2 | 0 | 0 | 0 |
| 4/3/2012 | 308.65 | 7.60 | 1 | 0 | 17 | 1 | 0 | 0 |
| 4/4/2012 | 280.34 | 7.45 | 0 | 0 | 16 | 1 | 0 | 0 |
| 4/5/2012 | 246.07 | 7.32 | 0 | 0 | 34 | 0 | 0 | 0 |

| Date | Discharge (cms) | Temp (C°) | Chinook | Coho | Steelhead | Cutthroat | Lamprey | Sucker |
|---|---|---|---|---|---|---|---|---|
| 4/6/2012 | 214.64 | 7.63 | 0 | 0 | 127 | 0 | 0 | 0 |
| 4/7/2012 | 188.02 | 7.96 | 0 | 0 | 178 | 2 | 0 | 0 |
| 4/8/2012 | 169.62 | 8.19 | 0 | 0 | 371 | 1 | 0 | 0 |
| 4/9/2012 | 157.44 | 8.55 | 0 | 0 | 256 | 0 | 0 | 0 |
| 4/10/2012 | 152.34 | 8.80 | 0 | 0 | 262 | 0 | 0 | 4 |
| 4/11/2012 | 166.50 | 8.99 | 0 | 0 | 259 | 0 | 0 | 5 |
| 4/12/2012 | 180.10 | 8.96 | 0 | 0 | 94 | 0 | 0 | 0 |
| 4/13/2012 | 173.87 | 8.35 | 0 | 0 | 39 | 1 | 0 | 0 |
| 4/14/2012 | 167.07 | 9.38 | 0 | 0 | 47 | 0 | 0 | 0 |
| 4/15/2012 | 158.29 | 8.96 | 0 | 0 | 153 | 0 | 0 | 1 |
| 4/16/2012 | 181.79 | 9.08 | 1 | 0 | 119 | 0 | 0 | 0 |
| 4/17/2012 | 334.14 | 8.37 | 3 | 0 | 2 | 0 | 0 | 2 |
| 4/18/2012 | 282.60 | 8.87 | 0 | 0 | 34 | 0 | 0 | 0 |
| 4/19/2012 | 280.34 | 9.01 | 0 | 0 | 86 | 1 | 0 | 0 |
| 4/20/2012 | 336.97 | 9.86 | 1 | 0 | 48 | 1 | 0 | 3 |
| 4/21/2012 | 291.66 | 10.63 | 0 | 0 | 130 | 0 | 0 | 58 |
| 4/22/2012 | 256.55 | 11.26 | 3 | 0 | 84 | 2 | 0 | 27 |
| 4/23/2012 | 241.83 | 12.20 | 2 | 0 | 81 | 2 | 0 | 120 |
| 4/24/2012 | 242.39 | 11.20 | 6 | 0 | 37 | 0 | 0 | 36 |
| 4/25/2012 | 212.09 | 10.67 | 3 | 0 | 46 | 0 | 0 | 6 |
| 4/26/2012 | 222.29 | 10.00 | 2 | 0 | 23 | 1 | 0 | 0 |
| 4/27/2012 | 237.30 | 8.88 | 0 | 0 | 0 | 0 | 0 | 0 |
| 4/28/2012 | 203.88 | 10.08 | 6 | 0 | 20 | 1 | 0 | 0 |
| 4/29/2012 | 176.13 | 10.73 | 2 | 0 | 21 | 0 | 0 | 0 |
| 4/30/2012 | 165.94 | 11.25 | 40 | 0 | 41 | 0 | 0 | 50 |
| 5/1/2012 | 182.36 | 10.70 | 4 | 0 | 6 | 0 | 0 | 31 |
| 5/2/2012 | 167.07 | 9.52 | 3 | 0 | 5 | 0 | 0 | 0 |
| 5/3/2012 | 163.67 | 9.49 | 26 | 0 | 11 | 0 | 0 | 0 |
| 5/4/2012 | 228.52 | 9.32 | 8 | 0 | 3 | 0 | 0 | 0 |
| 5/5/2012 | 222.57 | 9.41 | 6 | 0 | 6 | 0 | 0 | 0 |
| 5/6/2012 | 191.71 | 10.34 | 11 | 0 | 8 | 0 | 0 | 0 |
| 5/7/2012 | 165.94 | 10.41 | 1 | 0 | 2 | 0 | 0 | 0 |
| 5/8/2012 | 150.08 | 12.06 | 0 | 0 | 0 | 0 | 0 | 0 |
| 5/9/2012 | 142.72 | 12.15 | 35 | 0 | 28 | 0 | 0 | 52 |
| 5/10/2012 | 133.09 | 12.07 | 28 | 0 | 7 | 0 | 0 | 65 |
| 5/11/2012 | 122.05 | 11.77 | 19 | 0 | 3 | 0 | 0 | 18 |
| 5/12/2012 | 114.12 | 13.24 | 210 | 0 | 30 | 0 | 0 | 43 |
| 5/13/2012 | 109.87 | 14.34 | 326 | 0 | 35 | 0 | 0 | 1708 |
| 5/14/2012 | 110.44 | 13.54 | 38 | 0 | 7 | 0 | 0 | 177 |
| 5/15/2012 | 112.42 | 14.36 | 105 | 0 | 34 | 0 | 0 | 931 |
| 5/16/2012 | 112.42 | 14.98 | 261 | 0 | 21 | 1 | 0 | 181 |
| 5/17/2012 | 108.74 | 14.39 | 290 | 0 | 22 | 1 | 2 | 87 |
| 5/18/2012 | 100.24 | 14.04 | 121 | 0 | 18 | 2 | 0 | 14 |
| 5/19/2012 | 92.88 | 13.51 | 72 | 0 | 8 | 0 | 0 | 1 |
| 5/20/2012 | 86.08 | 13.52 | 166 | 0 | 14 | 3 | 0 | 10 |

| Date | Discharge (cms) | Temp (C°) | Chinook | Coho | Steelhead | Cutthroat | Lamprey | Sucker |
|---|---|---|---|---|---|---|---|---|
| 5/21/2012 | 83.25 | 13.36 | 25 | 0 | 3 | 1 | 0 | 4 |
| 5/22/2012 | 88.91 | 12.58 | 0 | 0 | 0 | 1 | 0 | 0 |
| 5/23/2012 | 91.75 | 11.59 | 57 | 0 | 14 | 0 | 0 | 3 |
| 5/24/2012 | 88.35 | 11.61 | 109 | 0 | 10 | 1 | 0 | 0 |
| 5/25/2012 | 119.50 | 10.30 | 32 | 0 | 7 | 0 | 0 | 0 |
| 5/26/2012 | 147.25 | 10.12 | 27 | 0 | 5 | 0 | 0 | 0 |
| 5/27/2012 | 133.66 | 12.60 | 234 | 0 | 20 | 0 | 0 | 6 |
| 5/28/2012 | 109.59 | 11.96 | 17 | 0 | 2 | 1 | 0 | 7 |
| 5/29/2012 | 96.84 | 11.50 | 96 | 0 | 11 | 1 | 0 | 23 |
| 5/30/2012 | 89.48 | 15.84 | 192 | 0 | 9 | 1 | 0 | 727 |
| 5/31/2012 | 83.82 | 15.11 | 360 | 0 | 16 | 9 | 3 | 332 |
| 6/1/2012 | 81.55 | 16.60 | 238 | 0 | 38 | 4 | 0 | 138 |
| 6/2/2012 | 82.12 | 16.95 | 144 | 0 | 18 | 2 | 5 | 283 |
| 6/3/2012 | 83.53 | 15.32 | 121 | 0 | 22 | 4 | 3 | 220 |
| 6/4/2012 | 88.63 | 13.83 | 2 | 0 | 1 | 0 | 0 | 6 |
| 6/5/2012 | 166.22 | 12.21 | 3 | 0 | 0 | 0 | 0 | 9 |
| 6/6/2012 | 154.89 | 12.36 | 18 | 0 | 13 | 0 | 0 | 2 |
| 6/7/2012 | 132.24 | 12.69 | 121 | 0 | 27 | 0 | 0 | 8 |
| 6/8/2012 | 135.92 | 12.16 | 104 | 0 | 19 | 0 | 0 | 1 |
| 6/9/2012 | 135.92 | 12.45 | 45 | 0 | 17 | 0 | 0 | 0 |
| 6/10/2012 | 133.09 | 13.00 | 32 | 0 | 27 | 1 | 0 | 0 |
| 6/11/2012 | 114.68 | 13.09 | 118 | 0 | 13 | 0 | 0 | 5 |
| 6/12/2012 | 101.94 | 14.12 | 0 | 0 | 0 | 0 | 0 | 0 |
| 6/13/2012 | 93.45 | 15.16 | 0 | 0 | 0 | 0 | 0 | 0 |
| 6/14/2012 | 86.37 | 15.91 | 435 | 0 | 194 | 0 | 3 | 737 |
| 6/15/2012 | 82.12 | 16.00 | 362 | 0 | 141 | 1 | 5 | 330 |
| 6/16/2012 | 77.30 | 16.33 | 230 | 0 | 54 | 0 | 0 | 472 |
| 6/17/2012 | 73.62 | 16.89 | 227 | 0 | 64 | 1 | 2 | 463 |
| 6/18/2012 | 70.79 | 17.45 | 63 | 0 | 61 | 0 | 0 | 280 |
| 6/19/2012 | 69.66 | 16.48 | 0 | 0 | 0 | 0 | 0 | 0 |
| 6/20/2012 | 67.11 | 17.11 | 41 | 0 | 50 | 1 | 0 | 115 |
| 6/21/2012 | 64.00 | 17.06 | 165 | 0 | 122 | 0 | 3 | 588 |
| 6/22/2012 | 64.00 | 16.86 | 128 | 0 | 94 | 0 | 2 | 305 |
| 6/23/2012 | 75.89 | 15.63 | 49 | 0 | 62 | 1 | 2 | 31 |
| 6/24/2012 | 82.12 | 14.70 | 31 | 0 | 31 | 0 | 0 | 2 |
| 6/25/2012 | 77.87 | 14.48 | 0 | 0 | 0 | 0 | 0 | 0 |
| 6/26/2012 | 78.72 | 13.83 | 0 | 0 | 0 | 0 | 0 | 0 |
| 6/27/2012 | 79.00 | 14.02 | 22 | 0 | 43 | 0 | 0 | 0 |
| 6/28/2012 | 73.91 | 15.74 | 131 | 0 | 156 | 0 | 0 | 993 |
| 6/29/2012 | 69.09 | 15.77 | 98 | 0 | 121 | 0 | 2 | 631 |
| 6/30/2012 | 65.41 | 16.26 | 66 | 0 | 79 | 0 | 1 | 378 |
| 7/1/2012 | 64.56 | 16.99 | 95 | 0 | 72 | 1 | 3 | 1463 |
| 7/2/2012 | 62.30 | 17.77 | 105 | 0 | 59 | 0 | 2 | 1232 |
| 7/3/2012 | 58.62 | 17.83 | 66 | 0 | 52 | 2 | 0 | 235 |
| 7/4/2012 | 56.07 | 17.90 | 48 | 0 | 35 | 4 | 2 | 289 |

| Date | Discharge (cms) | Temp (C°) | Chinook | Coho | Steelhead | Cutthroat | Lamprey | Sucker |
|---|---|---|---|---|---|---|---|---|
| 7/5/2012 | 54.37 | 18.20 | 53 | 0 | 44 | 3 | 3 | 792 |
| 7/6/2012 | 53.52 | 18.76 | 62 | 0 | 61 | 2 | 8 | 1024 |
| 7/7/2012 | 52.39 | 19.43 | 74 | 0 | 57 | 4 | 2 | 547 |
| 7/8/2012 | 49.55 | 20.15 | 103 | 0 | 70 | 5 | 19 | 311 |
| 7/9/2012 | 48.14 | 20.92 | 58 | 0 | 38 | 3 | 11 | 32 |
| 7/10/2012 | 46.44 | 20.45 | 56 | 0 | 73 | 14 | 0 | 0 |
| 7/11/2012 | 45.31 | 20.79 | 0 | 0 | 0 | 2 | 1 | 141 |
| 7/12/2012 | 44.17 | 21.57 | 55 | 0 | 77 | 7 | 0 | 94 |
| 7/13/2012 | 43.61 | 20.99 | 23 | 0 | 49 | 6 | 20 | 32 |
| 7/14/2012 | 42.48 | 21.28 | 26 | 0 | 43 | 10 | 3 | 31 |
| 7/15/2012 | 41.34 | 20.99 | 26 | 0 | 23 | 11 | 30 | 96 |
| 7/16/2012 | 41.06 | 19.17 | 3 | 0 | 6 | 2 | 0 | 0 |
| 7/17/2012 | 40.78 | 19.19 | 6 | 0 | 9 | 0 | 0 | 1 |
| 7/18/2012 | 42.19 | 18.36 | 14 | 0 | 14 | 0 | 0 | 0 |
| 7/19/2012 | 41.63 | 19.18 | 12 | 0 | 13 | 0 | 0 | 28 |
| 7/20/2012 | 41.06 | 19.72 | 34 | 0 | 55 | 0 | 0 | 268 |
| 7/21/2012 | 39.93 | 19.67 | 24 | 0 | 41 | 1 | 7 | 11 |
| 7/22/2012 | 39.36 | 19.54 | 20 | 0 | 49 | 0 | 7 | 3 |
| 7/23/2012 | 38.51 | 18.36 | 3 | 0 | 19 | 1 | 1 | 0 |
| 7/24/2012 | 37.38 | 19.09 | 0 | 0 | 0 | 1 | 0 | 0 |
| 7/25/2012 | 37.10 | 20.18 | 64 | 0 | 136 | 0 | 1 | 23 |
| 7/26/2012 | 36.81 | 20.85 | 55 | 0 | 96 | 0 | 1 | 34 |
| 7/27/2012 | 35.68 | 20.62 | 53 | 0 | 71 | 3 | 2 | 8 |
| 7/28/2012 | 35.40 | 19.87 | 32 | 0 | 47 | 3 | 0 | 2 |
| 7/29/2012 | 35.68 | 19.48 | 30 | 0 | 33 | 0 | 0 | 5 |
| 7/30/2012 | 36.25 | 19.19 | 20 | 0 | 34 | 2 | 1 | 12 |
| 7/31/2012 | 35.96 | 19.79 | 39 | 0 | 22 | 2 | 0 | 29 |
| 8/1/2012 | 35.68 | 19.86 | 35 | 0 | 38 | 5 | 0 | 23 |
| 8/2/2012 | 34.55 | 20.37 | 21 | 0 | 35 | 2 | 0 | 3 |
| 8/3/2012 | 33.98 | 20.85 | 21 | 0 | 26 | 4 | 2 | 2 |
| 8/4/2012 | 33.98 | 21.41 | 24 | 0 | 19 | 1 | 2 | 6 |
| 8/5/2012 | 33.70 | 22.03 | 30 | 0 | 36 | 8 | 1 | 21 |
| 8/6/2012 | 33.13 | 21.55 | 18 | 0 | 32 | 0 | 1 | 1 |
| 8/7/2012 | 33.13 | 21.22 | 5 | 0 | 18 | 3 | 0 | 3 |
| 8/8/2012 | 32.85 | 20.57 | 6 | 0 | 7 | 4 | 0 | 6 |
| 8/9/2012 | 32.56 | 20.67 | 2 | 0 | 3 | 2 | 1 | 4 |
| 8/10/2012 | 32.56 | 20.44 | 1 | 0 | 5 | 1 | 0 | 2 |
| 8/11/2012 | 32.28 | 20.62 | 2 | 0 | 6 | 2 | 0 | 6 |
| 8/12/2012 | 32.28 | 20.61 | 17 | 0 | 10 | 1 | 0 | 0 |
| 8/13/2012 | 32.28 | 21.39 | 15 | 0 | 4 | 0 | 0 | 0 |
| 8/14/2012 | 30.58 | 21.58 | 13 | 0 | 10 | 4 | 1 | 0 |
| 8/15/2012 | 30.30 | 21.86 | 8 | 0 | 8 | 4 | 1 | 4 |
| 8/16/2012 | 30.30 | 22.61 | 6 | 0 | 6 | 4 | 5 | 5 |
| 8/17/2012 | 30.30 | 22.32 | 9 | 0 | 4 | 2 | 2 | 5 |
| 8/18/2012 | 29.73 | 21.90 | 2 | 0 | 0 | 0 | 1 | 2 |

| Date | Discharge (cms) | Temp (C°) | Chinook | Coho | Steelhead | Cutthroat | Lamprey | Sucker |
|---|---|---|---|---|---|---|---|---|
| 8/19/2012 | 28.88 | 20.95 | 1 | 0 | 1 | 0 | 0 | 1 |
| 8/20/2012 | 28.29 | 20.73 | 0 | 0 | 1 | 0 | 0 | 0 |
| 8/21/2012 | 28.29 | 20.77 | 0 | 0 | 0 | 0 | 1 | 0 |
| 8/22/2012 | 28.60 | 20.40 | 9 | 0 | 2 | 0 | 0 | 0 |
| 8/23/2012 | 28.23 | 19.92 | 29 | 0 | 1 | 0 | 0 | 0 |
| 8/24/2012 | 28.15 | 19.28 | 40 | 0 | 1 | 0 | 0 | 0 |
| 8/25/2012 | 28.23 | 18.94 | 42 | 0 | 4 | 0 | 0 | 0 |
| 8/26/2012 | 28.15 | 18.76 | 36 | 0 | 14 | 0 | 0 | 0 |
| 8/27/2012 | 27.98 | 18.79 | 41 | 0 | 10 | 1 | 0 | 0 |
| 8/28/2012 | 28.12 | 18.96 | 46 | 0 | 17 | 0 | 0 | 0 |
| 8/29/2012 | 28.01 | 18.46 | 29 | 0 | 12 | 1 | 0 | 0 |
| 8/30/2012 | 28.06 | 18.30 | 57 | 0 | 19 | 0 | 0 | 0 |
| 8/31/2012 | 27.67 | 18.41 | 45 | 0 | 18 | 2 | 0 | 0 |
| 9/1/2012 | 27.27 | 17.98 | 30 | 0 | 16 | 1 | 0 | 0 |
| 9/2/2012 | 27.01 | 17.79 | 23 | 1 | 23 | 1 | 0 | 0 |
| 9/3/2012 | 26.65 | 17.67 | 15 | 0 | 19 | 0 | 0 | 0 |
| 9/4/2012 | 26.62 | 17.88 | 44 | 0 | 23 | 0 | 0 | 0 |
| 9/5/2012 | 26.36 | 18.06 | 73 | 0 | 17 | 0 | 0 | 0 |
| 9/6/2012 | 27.84 | 18.57 | 33 | 0 | 17 | 0 | 0 | 0 |
| 9/7/2012 | 28.88 | 18.62 | 36 | 0 | 24 | 1 | 0 | 0 |
| 9/8/2012 | 29.45 | 18.51 | 41 | 2 | 14 | 0 | 0 | 0 |
| 9/9/2012 | 29.45 | 17.91 | 24 | 0 | 15 | 0 | 0 | 0 |
| 9/10/2012 | 29.73 | 17.34 | 18 | 0 | 19 | 0 | 0 | 0 |
| 9/11/2012 | 29.45 | 16.88 | 7 | 1 | 9 | 0 | 0 | 0 |
| 9/12/2012 | 28.88 | 16.65 | 6 | 0 | 5 | 0 | 0 | 0 |
| 9/13/2012 | 29.17 | 16.66 | 4 | 1 | 7 | 0 | 0 | 0 |
| 9/14/2012 | 28.88 | 17.25 | 24 | 3 | 12 | 1 | 0 | 0 |
| 9/15/2012 | 28.88 | 17.49 | 38 | 2 | 17 | 1 | 0 | 0 |
| 9/16/2012 | 28.60 | 17.29 | 25 | 3 | 8 | 1 | 0 | 0 |
| 9/17/2012 | 28.29 | 17.35 | 15 | 4 | 10 | 0 | 0 | 0 |
| 9/18/2012 | 27.95 | 17.38 | 13 | 2 | 7 | 0 | 0 | 1 |
| 9/19/2012 | 27.78 | 17.11 | 19 | 5 | 28 | 1 | 0 | 0 |
| 9/20/2012 | 27.67 | 16.54 | 7 | 5 | 25 | 0 | 0 | 0 |
| 9/21/2012 | 27.67 | 15.92 | 6 | 5 | 17 | 1 | 0 | 0 |
| 9/22/2012 | 27.78 | 16.09 | 9 | 6 | 15 | 0 | 0 | 0 |
| 9/23/2012 | 27.89 | 16.56 | 12 | 2 | 11 | 0 | 0 | 0 |
| 9/24/2012 | 27.75 | 16.14 | 16 | 17 | 15 | 0 | 0 | 0 |
| 9/25/2012 | 27.84 | 15.79 | 6 | 19 | 7 | 1 | 0 | 0 |
| 9/26/2012 | 27.75 | 15.54 | 7 | 17 | 8 | 0 | 0 | 0 |
| 9/27/2012 | 27.69 | 15.34 | 14 | 23 | 12 | 0 | 0 | 0 |
| 9/28/2012 | 27.81 | 15.53 | 12 | 38 | 10 | 0 | 0 | 0 |
| 9/29/2012 | 27.21 | 15.71 | 19 | 33 | 17 | 0 | 0 | 0 |
| 9/30/2012 | 27.16 | 15.78 | 12 | 46 | 9 | 0 | 0 | 0 |
| 10/1/2012 | 27.24 | 15.31 | 4 | 13 | 3 | 0 | 0 | 0 |
| 10/2/2012 | 26.99 | 15.35 | 0 | 0 | 0 | 0 | 0 | 0 |

| Date | Discharge (cms) | Temp (C°) | Chinook | Coho | Steelhead | Cutthroat | Lamprey | Sucker |
|---|---|---|---|---|---|---|---|---|
| 10/3/2012 | 27.01 | 14.65 | 3 | 14 | 14 | 1 | 0 | 0 |
| 10/4/2012 | 26.99 | 13.60 | 7 | 43 | 10 | 0 | 0 | 0 |
| 10/5/2012 | 26.90 | 13.16 | 6 | 20 | 19 | 0 | 0 | 0 |
| 10/6/2012 | 26.84 | 12.66 | 5 | 22 | 10 | 0 | 0 | 0 |
| 10/7/2012 | 26.79 | 12.29 | 4 | 29 | 7 | 0 | 0 | 0 |
| 10/8/2012 | 26.87 | 11.95 | 7 | 27 | 9 | 0 | 0 | 0 |
| 10/9/2012 | 26.84 | 11.57 | 4 | 8 | 16 | 0 | 0 | 0 |
| 10/10/2012 | 26.96 | 11.54 | 8 | 15 | 3 | 0 | 0 | 0 |
| 10/11/2012 | 27.01 | 11.53 | 6 | 25 | 5 | 1 | 0 | 0 |
| 10/12/2012 | 27.75 | 11.45 | 12 | 28 | 12 | 0 | 0 | 0 |
| 10/13/2012 | 31.15 | 12.14 | 30 | 57 | 14 | 0 | 0 | 1 |
| 10/14/2012 | 31.71 | 12.34 | 32 | 119 | 31 | 0 | 0 | 0 |
| 10/15/2012 | 31.43 | 13.20 | 48 | 99 | 21 | 0 | 0 | 2 |
| 10/16/2012 | 56.63 | 13.50 | 45 | 193 | 52 | 0 | 0 | 7 |
| 10/17/2012 | 60.88 | 12.27 | 56 | 397 | 44 | 1 | 0 | 15 |
| 10/18/2012 | 37.10 | 12.15 | 25 | 249 | 29 | 1 | 0 | 0 |
| 10/19/2012 | 31.43 | 12.11 | 4 | 47 | 15 | 0 | 0 | 0 |
| 10/20/2012 | 32.56 | 11.68 | 3 | 58 | 18 | 0 | 0 | 0 |
| 10/21/2012 | 37.94 | 10.53 | 1 | 44 | 7 | 0 | 0 | 0 |
| 10/22/2012 | 36.53 | 10.00 | 0 | 18 | 6 | 0 | 0 | 0 |
| 10/23/2012 | 46.44 | 9.59 | 1 | 42 | 4 | 0 | 0 | 0 |
| 10/24/2012 | 48.99 | 9.24 | 2 | 47 | 4 | 0 | 0 | 0 |
| 10/25/2012 | 52.95 | 9.22 | 1 | 38 | 3 | 0 | 0 | 0 |
| 10/26/2012 | 45.31 | 9.40 | 1 | 40 | 4 | 0 | 0 | 0 |
| 10/27/2012 | 39.93 | 9.32 | 1 | 42 | 5 | 0 | 0 | 0 |
| 10/28/2012 | 52.95 | 9.65 | 6 | 73 | 8 | 0 | 0 | 0 |
| 10/29/2012 | 87.22 | 10.24 | 7 | 484 | 47 | 0 | 0 | 1 |
| 10/30/2012 | 70.79 | 10.77 | 6 | 331 | 24 | 0 | 0 | 2 |
| 10/31/2012 | 77.87 | 10.70 | 4 | 69 | 8 | 0 | 0 | 0 |
| 11/1/2012 | 65.70 | 10.47 | 1 | 49 | 21 | 0 | 0 | 1 |
| 11/2/2012 | 58.33 | 10.04 | 0 | 60 | 12 | 0 | 0 | 0 |
| 11/3/2012 | 49.84 | 10.27 | 0 | 32 | 16 | 0 | 0 | 0 |
| 11/4/2012 | 43.61 | 11.15 | 0 | 18 | 10 | 0 | 0 | 0 |
| 11/5/2012 | 40.21 | 11.24 | 2 | 28 | 10 | 0 | 0 | 0 |
| 11/6/2012 | 37.66 | 11.34 | 2 | 24 | 15 | 0 | 0 | 0 |
| 11/7/2012 | 35.96 | 11.05 | 0 | 4 | 6 | 0 | 0 | 0 |
| 11/8/2012 | 35.11 | 9.67 | 0 | 1 | 3 | 0 | 0 | 0 |
| 11/9/2012 | 34.83 | 8.73 | 0 | 0 | 1 | 0 | 0 | 0 |
| 11/10/2012 | 34.83 | 8.23 | 0 | 0 | 0 | 0 | 0 | 0 |
| 11/11/2012 | 34.26 | 7.97 | 0 | 0 | 0 | 0 | 0 | 0 |
| 11/12/2012 | 33.98 | 7.68 | 0 | 0 | 1 | 0 | 0 | 0 |
| 11/13/2012 | 41.06 | 7.99 | 0 | 0 | 0 | 0 | 0 | 0 |
| 11/14/2012 | 47.29 | 8.10 | 0 | 2 | 3 | 0 | 0 | 0 |
| 11/15/2012 | 43.04 | 8.43 | 0 | 5 | 2 | 0 | 0 | 0 |
| 11/16/2012 | 39.64 | 7.81 | 0 | 0 | 5 | 0 | 0 | 0 |

| Date | Discharge (cms) | Temp (C°) | Chinook | Coho | Steelhead | Cutthroat | Lamprey | Sucker |
|---|---|---|---|---|---|---|---|---|
| 11/17/2012 | 38.23 | 7.46 | 0 | 0 | 0 | 0 | 0 | 0 |
| 11/18/2012 | 45.02 | 7.19 | 0 | 0 | 5 | 0 | 0 | 0 |
| 11/19/2012 | 48.99 | 8.28 | 0 | 14 | 11 | 0 | 0 | 0 |
| 11/20/2012 | 637.13 | 9.01 | 0 | 11 | 2 | 0 | 0 | 0 |
| 11/21/2012 | 572.00 | 9.40 | 0 | 8 | 0 | 0 | 0 | 0 |
| 11/22/2012 | 297.33 | 8.61 | 0 | 47 | 3 | 0 | 0 | 0 |
| 11/23/2012 | 171.88 | 8.13 | 0 | 54 | 30 | 0 | 0 | 0 |
| 11/24/2012 | 151.21 | 8.55 | 0 | 54 | 54 | 0 | 0 | 0 |
| 11/25/2012 | 220.02 | 9.00 | 0 | 28 | 22 | 0 | 0 | 0 |
| 11/26/2012 | 171.03 | 8.55 | 0 | 36 | 25 | 0 | 0 | 0 |
| 11/27/2012 | 127.71 | 7.66 | 0 | 9 | 26 | 0 | 0 | 0 |
| 11/28/2012 | 101.37 | 7.76 | 0 | 7 | 20 | 0 | 0 | 0 |
| 11/29/2012 | 115.53 | 8.21 | 0 | 10 | 35 | 0 | 0 | 0 |
| 11/30/2012 | 464.40 | 9.17 | 0 | 1 | 6 | 0 | 0 | 0 |
| 12/1/2012 | 424.75 | 9.40 | 0 | 3 | 16 | 0 | 0 | 0 |
| 12/2/2012 | 798.54 | 9.20 | 0 | 1 | 0 | 0 | 0 | 0 |
| 12/3/2012 | 489.88 | 8.96 | 0 | 1 | 6 | 0 | 0 | 0 |
| 12/4/2012 | 470.06 | 9.05 | 0 | 2 | 7 | 0 | 0 | 0 |
| 12/5/2012 | 906.14 | 9.53 | 0 | 0 | 0 | 0 | 0 | 0 |
| 12/6/2012 | 478.55 | 8.78 | 0 | 1 | 9 | 0 | 0 | 0 |
| 12/7/2012 | 288.83 | 7.98 | 0 | 1 | 5 | 0 | 0 | 0 |
| 12/8/2012 | 212.09 | 7.85 | 0 | 0 | 45 | 0 | 0 | 0 |
| 12/9/2012 | 168.49 | 7.49 | 0 | 0 | 44 | 0 | 0 | 0 |
| 12/10/2012 | 141.30 | 7.54 | 0 | 0 | 94 | 0 | 0 | 0 |
| 12/11/2012 | 123.46 | 7.55 | 0 | 1 | 67 | 0 | 0 | 0 |
| 12/12/2012 | 133.37 | 7.09 | 0 | 0 | 29 | 0 | 0 | 0 |
| 12/13/2012 | 125.73 | 6.55 | 0 | 0 | 14 | 0 | 0 | 0 |
| 12/14/2012 | 112.98 | 5.94 | 0 | 0 | 9 | 0 | 0 | 0 |
| 12/15/2012 | 104.49 | 5.47 | 0 | 0 | 5 | 0 | 0 | 0 |
| 12/16/2012 | 114.97 | 5.65 | 0 | 0 | 7 | 0 | 0 | 0 |
| 12/17/2012 | 269.01 | 6.23 | 0 | 0 | 4 | 0 | 0 | 0 |
| 12/18/2012 | 267.31 | 6.53 | 0 | 0 | 9 | 0 | 0 | 0 |
| 12/19/2012 | 183.78 | 6.15 | 0 | 0 | 26 | 0 | 0 | 0 |
| 12/20/2012 | 202.47 | 6.00 | 0 | 0 | 34 | 0 | 0 | 0 |
| 12/21/2012 | 231.91 | 6.42 | 0 | 0 | 21 | 0 | 0 | 0 |
| 12/22/2012 | 228.52 | 6.71 | 0 | 0 | 68 | 0 | 0 | 0 |
| 12/23/2012 | 250.60 | 6.67 | 0 | 0 | 38 | 0 | 0 | 0 |
| 12/24/2012 | 249.75 | 6.90 | 0 | 0 | 39 | 0 | 0 | 0 |
| 12/25/2012 | 196.52 | 6.90 | 0 | 0 | 88 | 0 | 0 | 0 |
| 12/26/2012 | 207.00 | 7.05 | 0 | 0 | 104 | 0 | 0 | 0 |
| 12/27/2012 | 219.74 | 7.18 | 0 | 0 | 88 | 0 | 0 | 0 |
| 12/28/2012 | 182.93 | 6.56 | 0 | 0 | 37 | 0 | 0 | 0 |
| 12/29/2012 | 149.23 | 6.40 | 0 | 0 | 100 | 0 | 0 | 0 |
| 12/30/2012 | 124.88 | 6.21 | 0 | 0 | 86 | 0 | 0 | 0 |
| 12/31/2012 | 109.30 | 5.54 | 0 | 0 | 11 | 0 | 0 | 0 |

| Date | Discharge (cms) | Temp (C°) | Chinook | Coho | Steelhead | Cutthroat | Lamprey | Sucker |
|---|---|---|---|---|---|---|---|---|
| 1/1/2013 | 98.26 | 5.27 | 0 | 0 | 4 | 0 | 0 | 0 |
| 1/2/2013 | 89.20 | 4.48 | 0 | 0 | 1 | 0 | 0 | 0 |
| 1/3/2013 | 84.67 | 4.17 | 0 | 0 | 2 | 0 | 0 | 0 |
| 1/4/2013 | 82.69 | 4.36 | 0 | 0 | 0 | 0 | 0 | 0 |
| 1/5/2013 | 86.08 | 4.74 | 0 | 0 | 5 | 0 | 0 | 0 |
| 1/6/2013 | 92.60 | 5.68 | 0 | 0 | 22 | 0 | 0 | 0 |
| 1/7/2013 | 93.45 | 6.20 | 0 | 0 | 51 | 0 | 0 | 0 |
| 1/8/2013 | 135.35 | 6.77 | 0 | 0 | 205 | 0 | 0 | 0 |
| 1/9/2013 | 160.56 | 6.97 | 0 | 0 | 236 | 0 | 0 | 0 |
| 1/10/2013 | 184.91 | 5.96 | 0 | 0 | 23 | 0 | 0 | 0 |
| 1/11/2013 | 154.33 | 5.40 | 0 | 0 | 4 | 0 | 0 | 0 |
| 1/12/2013 | 127.43 | 4.67 | 0 | 0 | 1 | 0 | 0 | 0 |
| 1/13/2013 | 107.60 | 3.49 | 0 | 0 | 0 | 0 | 0 | 0 |
| 1/14/2013 | 94.58 | 2.51 | 0 | 0 | 0 | 0 | 0 | 0 |
| 1/15/2013 | 84.95 | 1.85 | 0 | 0 | 0 | 0 | 0 | 0 |
| 1/16/2013 | 81.84 | 2.19 | 0 | 0 | 0 | 0 | 0 | 0 |
| 1/17/2013 | 86.93 | 2.97 | 0 | 0 | 1 | 0 | 0 | 0 |
| 1/18/2013 | 107.04 | 3.37 | 0 | 0 | 0 | 0 | 0 | 0 |
| 1/19/2013 | 123.74 | 3.89 | 0 | 0 | 2 | 0 | 0 | 0 |
| 1/20/2013 | 126.86 | 4.17 | 0 | 0 | 5 | 0 | 0 | 0 |
| 1/21/2013 | 120.06 | 3.97 | 0 | 0 | 1 | 0 | 0 | 0 |
| 1/22/2013 | 113.55 | 3.83 | 0 | 0 | 0 | 0 | 0 | 0 |
| 1/23/2013 | 114.12 | 4.71 | 0 | 0 | 25 | 0 | 0 | 0 |
| 1/24/2013 | 160.56 | 5.88 | 0 | 0 | 113 | 0 | 0 | 0 |
| 1/25/2013 | 214.08 | 6.83 | 0 | 0 | 199 | 0 | 0 | 0 |
| 1/26/2013 | 390.77 | 7.07 | 0 | 0 | 86 | 0 | 0 | 0 |
| 1/27/2013 | 275.52 | 6.67 | 0 | 0 | 57 | 0 | 0 | 0 |
| 1/28/2013 | 243.52 | 6.11 | 0 | 0 | 15 | 0 | 0 | 0 |
| 1/29/2013 | 259.67 | 6.15 | 0 | 0 | 16 | 0 | 0 | 0 |
| 1/30/2013 | 258.25 | 6.94 | 0 | 0 | 209 | 0 | 0 | 0 |
| 1/31/2013 | 216.62 | 7.16 | 0 | 0 | 452 | 0 | 0 | 0 |
| 2/1/2013 | 186.61 | 7.13 | 0 | 0 | 357 | 0 | 0 | 0 |
| 2/2/2013 | 169.90 | 6.57 | 0 | 0 | 90 | 0 | 0 | 0 |
| 2/3/2013 | 163.95 | 6.60 | 0 | 0 | 115 | 0 | 0 | 0 |
| 2/4/2013 | 149.80 | 6.88 | 0 | 0 | 107 | 0 | 0 | 0 |
| 2/5/2013 | 138.47 | 6.98 | 0 | 0 | 128 | 0 | 0 | 0 |
| 2/6/2013 | 137.62 | 7.14 | 0 | 0 | 142 | 0 | 0 | 0 |
| 2/7/2013 | 134.51 | 6.90 | 0 | 0 | 122 | 0 | 0 | 0 |
| 2/8/2013 | 124.59 | 6.47 | 0 | 0 | 10 | 0 | 0 | 0 |
| 2/9/2013 | 109.87 | 6.03 | 0 | 0 | 7 | 0 | 0 | 0 |
| 2/10/2013 | 100.24 | 5.94 | 0 | 0 | 4 | 0 | 0 | 0 |
| 2/11/2013 | 92.88 | 6.26 | 0 | 0 | 3 | 0 | 0 | 0 |
| 2/12/2013 | 86.37 | 6.95 | 0 | 0 | 54 | 0 | 0 | 0 |
| 2/13/2013 | 82.12 | 7.24 | 0 | 0 | 183 | 0 | 0 | 0 |
| 2/14/2013 | 80.14 | 7.65 | 0 | 0 | 62 | 0 | 0 | 0 |

| Date | Discharge (cms) | Temp (C°) | Chinook | Coho | Steelhead | Cutthroat | Lamprey | Sucker |
|---|---|---|---|---|---|---|---|---|
| 2/15/2013 | 80.42 | 7.40 | 0 | 0 | 86 | 0 | 0 | 0 |
| 2/16/2013 | 83.53 | 6.73 | 0 | 0 | 1 | 0 | 0 | 0 |
| 2/17/2013 | 85.23 | 6.54 | 0 | 0 | 3 | 0 | 0 | 0 |
| 2/18/2013 | 80.99 | 6.43 | 0 | 0 | 3 | 0 | 0 | 0 |
| 2/19/2013 | 78.15 | 6.08 | 0 | 0 | 0 | 0 | 0 | 0 |
| 2/20/2013 | 74.76 | 6.18 | 0 | 0 | 2 | 0 | 0 | 0 |
| 2/21/2013 | 71.36 | 6.49 | 0 | 0 | 17 | 0 | 0 | 0 |
| 2/22/2013 | 70.51 | 6.55 | 0 | 0 | 27 | 0 | 0 | 0 |
| 2/23/2013 | 100.81 | 6.41 | 0 | 0 | 361 | 0 | 0 | 0 |
| 2/24/2013 | 103.07 | 6.53 | 0 | 0 | 37 | 0 | 0 | 0 |
| 2/25/2013 | 96.56 | 6.62 | 0 | 0 | 73 | 0 | 0 | 0 |
| 2/26/2013 | 102.51 | 6.44 | 0 | 0 | 71 | 0 | 0 | 0 |
| 2/27/2013 | 97.69 | 6.64 | 0 | 0 | 115 | 0 | 0 | 0 |
| 2/28/2013 | 93.73 | 7.13 | 0 | 0 | 589 | 0 | 0 | 0 |
| 3/1/2013 | 165.37 | 7.86 | 0 | 0 | 757 | 0 | 0 | 0 |
| 3/2/2013 | 164.52 | 7.86 | 0 | 0 | 729 | 2 | 0 | 0 |
| 3/3/2013 | 154.04 | 8.17 | 0 | 0 | 374 | 0 | 0 | 0 |
| 3/4/2013 | 135.92 | 7.76 | 0 | 0 | 101 | 0 | 0 | 0 |
| 3/5/2013 | 118.08 | 6.68 | 0 | 0 | 12 | 0 | 0 | 0 |
| 3/6/2013 | 134.79 | 7.17 | 0 | 0 | 231 | 0 | 0 | 0 |
| 3/7/2013 | 138.75 | 7.70 | 0 | 0 | 72 | 0 | 0 | 0 |
| 3/8/2013 | 123.18 | 6.52 | 0 | 0 | 3 | 0 | 0 | 0 |
| 3/9/2013 | 109.59 | 7.26 | 0 | 0 | 19 | 0 | 0 | 0 |
| 3/10/2013 | 96.84 | 7.55 | 0 | 0 | 49 | 1 | 0 | 0 |
| 3/11/2013 | 90.90 | 8.21 | 0 | 0 | 170 | 0 | 0 | 0 |
| 3/12/2013 | 90.05 | 8.44 | 0 | 0 | 297 | 1 | 0 | 0 |
| 3/13/2013 | 92.60 | 9.12 | 0 | 0 | 437 | 1 | 0 | 1 |
| 3/14/2013 | 96.84 | 8.98 | 0 | 0 | 239 | 0 | 0 | 0 |
| 3/15/2013 | 101.94 | 9.59 | 0 | 0 | 219 | 0 | 0 | 8 |
| 3/16/2013 | 100.81 | 9.06 | 0 | 0 | 113 | 0 | 0 | 1 |
| 3/17/2013 | 106.47 | 9.03 | 0 | 0 | 69 | 0 | 0 | 0 |
| 3/18/2013 | 98.54 | 8.16 | 0 | 0 | 3 | 0 | 0 | 0 |
| 3/19/2013 | 89.48 | 7.81 | 0 | 0 | 5 | 0 | 0 | 0 |
| 3/20/2013 | 97.41 | 8.06 | 0 | 0 | 11 | 0 | 0 | 0 |
| 3/21/2013 | 183.49 | 7.72 | 0 | 0 | 88 | 0 | 0 | 0 |
| 3/22/2013 | 149.80 | 7.13 | 0 | 0 | 7 | 0 | 0 | 0 |
| 3/23/2013 | 124.03 | 7.26 | 0 | 0 | 5 | 0 | 0 | 0 |
| 3/24/2013 | 109.59 | 7.27 | 0 | 0 | 14 | 0 | 0 | 0 |
| 3/25/2013 | 99.96 | 7.74 | 0 | 0 | 22 | 0 | 0 | 0 |
| 3/26/2013 | 94.86 | 8.46 | 0 | 0 | 160 | 0 | 0 | 0 |
| 3/27/2013 | 92.88 | 9.16 | 0 | 0 | 160 | 0 | 0 | 0 |
| 3/28/2013 | 96.28 | 9.49 | 0 | 0 | 288 | 0 | 0 | 5 |
| 3/29/2013 | 104.21 | 10.87 | 0 | 0 | 146 | 1 | 0 | 193 |
| 3/30/2013 | 107.32 | 10.78 | 0 | 0 | 142 | 0 | 0 | 65 |
| 3/31/2013 | 108.74 | 10.46 | 1 | 0 | 191 | 0 | 0 | 56 |

| Date | Discharge (cms) | Temp (C°) | Chinook | Coho | Steelhead | Cutthroat | Lamprey | Sucker |
|---|---|---|---|---|---|---|---|---|
| 4/1/2013 | 127.71 | 9.86 | 0 | 0 | 130 | 0 | 0 | 6 |
| 4/2/2013 | 148.38 | 10.20 | 0 | 0 | 72 | 0 | 0 | 7 |
| 4/3/2013 | 136.20 | 9.77 | 1 | 0 | 34 | 0 | 0 | 0 |
| 4/4/2013 | 124.88 | 9.83 | 1 | 0 | 52 | 0 | 0 | 0 |
| 4/5/2013 | 148.38 | 9.83 | 4 | 0 | 102 | 0 | 0 | 0 |
| 4/6/2013 | 169.33 | 9.52 | 0 | 0 | 26 | 0 | 0 | 0 |
| 4/7/2013 | 235.88 | 8.87 | 1 | 0 | 13 | 0 | 0 | 0 |
| 4/8/2013 | 272.69 | 8.16 | 0 | 0 | 1 | 0 | 0 | 0 |
| 4/9/2013 | 221.44 | 8.64 | 0 | 0 | 2 | 0 | 0 | 0 |
| 4/10/2013 | 183.78 | 8.64 | 0 | 0 | 9 | 0 | 0 | 0 |
| 4/11/2013 | 163.11 | 9.48 | 0 | 0 | 22 | 0 | 0 | 0 |
| 4/12/2013 | 143.00 | 9.71 | 0 | 0 | 45 | 0 | 0 | 0 |
| 4/13/2013 | 127.71 | 9.52 | 4 | 0 | 21 | 0 | 0 | 0 |
| 4/14/2013 | 117.23 | 8.87 | 0 | 0 | 6 | 0 | 0 | 0 |
| 4/15/2013 | 107.32 | 8.73 | 3 | 0 | 9 | 1 | 0 | 0 |
| 4/16/2013 | 102.22 | 8.41 | 0 | 0 | 0 | 0 | 0 | 0 |
| 4/17/2013 | 94.30 | 9.40 | 2 | 0 | 10 | 0 | 0 | 0 |
| 4/18/2013 | 88.91 | 10.33 | 3 | 0 | 28 | 0 | 0 | 4 |
| 4/19/2013 | 87.50 | 9.74 | 8 | 0 | 31 | 0 | 0 | 1 |
| 4/20/2013 | 98.83 | 11.21 | 1 | 0 | 28 | 0 | 0 | 157 |
| 4/21/2013 | 101.94 | 11.71 | 10 | 0 | 66 | 0 | 0 | 243 |
| 4/22/2013 | 95.71 | 12.87 | 13 | 0 | 31 | 1 | 0 | 412 |
| 4/23/2013 | 88.63 | 12.90 | 5 | 0 | 19 | 0 | 0 | 292 |
| 4/24/2013 | 84.10 | 12.41 | 14 | 0 | 20 | 0 | 0 | 92 |
| 4/25/2013 | 80.99 | 12.85 | 31 | 0 | 21 | 0 | 0 | 56 |
| 4/26/2013 | 78.72 | 13.94 | 19 | 0 | 21 | 0 | 0 | 164 |
| 4/27/2013 | 78.72 | 14.57 | 35 | 0 | 27 | 0 | 0 | 353 |
| 4/28/2013 | 78.15 | 14.25 | 23 | 0 | 14 | 1 | 0 | 89 |
| 4/29/2013 | 76.46 | 13.85 | 85 | 0 | 13 | 0 | 0 | 17 |
| 4/30/2013 | 76.17 | 13.14 | 12 | 0 | 3 | 0 | 0 | 4 |
| 5/1/2013 | 72.21 | 12.99 | 26 | 0 | 2 | 0 | 0 | 0 |
| 5/2/2013 | 67.39 | 13.36 | 43 | 0 | 1 | 0 | 0 | 1 |
| 5/3/2013 | 65.70 | 14.06 | 67 | 0 | 8 | 1 | 1 | 29 |
| 5/4/2013 | 64.56 | 14.96 | 116 | 0 | 11 | 0 | 0 | 111 |
| 5/5/2013 | 64.56 | 15.72 | 161 | 0 | 12 | 1 | 0 | 255 |
| 5/6/2013 | 66.26 | 15.80 | 29 | 0 | 4 | 0 | 1 | 35 |
| 5/7/2013 | 65.13 | 16.17 | 0 | 0 | 0 | 0 | 0 | 0 |
| 5/8/2013 | 65.13 | 15.68 | 37 | 0 | 21 | 4 | 0 | 1127 |
| 5/9/2013 | 64.85 | 16.96 | 160 | 0 | 7 | 0 | 0 | 748 |
| 5/10/2013 | 64.28 | 16.93 | 124 | 0 | 12 | 0 | 0 | 1374 |
| 5/11/2013 | 63.15 | 17.76 | 121 | 0 | 10 | 1 | 0 | 998 |
| 5/12/2013 | 62.58 | 17.46 | 114 | 0 | 5 | 0 | 1 | 908 |
| 5/13/2013 | 61.45 | 17.74 | 28 | 0 | 1 | 0 | 0 | 37 |
| 5/14/2013 | 60.03 | 16.56 | 0 | 0 | 0 | 1 | 0 | 0 |
| 5/15/2013 | 56.92 | 15.71 | 21 | 0 | 5 | 0 | 0 | 48 |

| Date | Discharge (cms) | Temp (C°) | Chinook | Coho | Steelhead | Cutthroat | Lamprey | Sucker |
|---|---|---|---|---|---|---|---|---|
| 5/16/2013 | 54.09 | 14.98 | 195 | 0 | 6 | 0 | 0 | 1 |
| 5/17/2013 | 54.93 | 14.44 | 169 | 0 | 10 | 1 | 0 | 1 |
| 5/18/2013 | 55.22 | 13.86 | 107 | 0 | 6 | 0 | 0 | 0 |
| 5/19/2013 | 52.95 | 13.93 | 91 | 0 | 6 | 1 | 0 | 1 |
| 5/20/2013 | 50.97 | 14.43 | 88 | 0 | 8 | 0 | 0 | 2 |
| 5/21/2013 | 49.27 | 15.19 | 0 | 0 | 0 | 0 | 0 | 0 |
| 5/22/2013 | 53.52 | 12.70 | 46 | 0 | 6 | 0 | 0 | 225 |
| 5/23/2013 | 58.33 | 11.72 | 38 | 0 | 8 | 0 | 0 | 27 |
| 5/24/2013 | 56.92 | 11.93 | 52 | 0 | 9 | 0 | 0 | 0 |
| 5/25/2013 | 58.90 | 13.19 | 156 | 0 | 15 | 0 | 0 | 0 |
| 5/26/2013 | 57.77 | 14.54 | 293 | 0 | 35 | 0 | 0 | 1300 |
| 5/27/2013 | 69.38 | 14.37 | 240 | 0 | 28 | 0 | 0 | 1205 |
| 5/28/2013 | 103.92 | 14.29 | 54 | 0 | 8 | 0 | 0 | 506 |
| 5/29/2013 | 99.96 | 13.24 | 41 | 0 | 4 | 0 | 0 | 191 |
| 5/30/2013 | 103.92 | 14.04 | 56 | 0 | 5 | 0 | 0 | 0 |
| 5/31/2013 | 91.18 | 14.63 | 29 | 0 | 17 | 2 | 0 | 36 |
| 6/1/2013 | 77.59 | 15.58 | 179 | 0 | 27 | 1 | 1 | 1983 |
| 6/2/2013 | 67.68 | 15.45 | 248 | 0 | 44 | 0 | 0 | 843 |
| 6/3/2013 | 62.58 | 16.72 | 171 | 0 | 21 | 1 | 1 | 1906 |
| 6/4/2013 | 58.62 | 18.26 | 81 | 0 | 5 | 0 | 0 | 1577 |
| 6/5/2013 | 55.22 | 18.18 | 143 | 0 | 27 | 1 | 4 | 2700 |
| 6/6/2013 | 52.67 | 19.11 | 187 | 0 | 22 | 5 | 3 | 1718 |
| 6/7/2013 | 49.55 | 20.16 | 119 | 0 | 30 | 6 | 12 | 1274 |
| 6/8/2013 | 47.86 | 20.07 | 158 | 0 | 34 | 10 | 18 | 1088 |
| 6/9/2013 | 46.72 | 19.80 | 82 | 0 | 22 | 2 | 12 | 180 |
| 6/10/2013 | 44.74 | 19.64 | 1 | 0 | 0 | 2 | 18 | 7 |
| 6/11/2013 | 43.89 | 18.70 | 60 | 0 | 11 | 2 | 0 | 101 |
| 6/12/2013 | 42.76 | 17.16 | 34 | 0 | 16 | 0 | 0 | 1 |
| 6/13/2013 | 41.91 | 16.22 | 0 | 0 | 0 | 0 | 0 | 0 |
| 6/14/2013 | 41.34 | 17.20 | 30 | 0 | 12 | 0 | 2 | 5 |
| 6/15/2013 | 40.78 | 17.66 | 98 | 0 | 20 | 0 | 0 | 1215 |
| 6/16/2013 | 39.64 | 18.26 | 131 | 0 | 38 | 0 | 2 | 261 |
| 6/17/2013 | 38.51 | 18.31 | 49 | 0 | 15 | 0 | 6 | 1 |
| 6/18/2013 | 37.94 | 18.84 | 34 | 0 | 8 | 1 | 2 | 19 |
| 6/19/2013 | 39.93 | 18.08 | 90 | 0 | 80 | 2 | 0 | 3 |
| 6/20/2013 | 46.44 | 16.99 | 67 | 0 | 57 | 0 | 1 | 0 |
| 6/21/2013 | 43.32 | 16.96 | 66 | 0 | 50 | 0 | 0 | 14 |
| 6/22/2013 | 39.64 | 17.60 | 104 | 0 | 47 | 0 | 2 | 79 |
| 6/23/2013 | 38.79 | 17.99 | 142 | 0 | 66 | 0 | 4 | 63 |
| 6/24/2013 | 38.23 | 17.33 | 59 | 0 | 48 | 0 | 1 | 0 |
| 6/25/2013 | 39.93 | 16.61 | 10 | 0 | 11 | 1 | 0 | 0 |
| 6/26/2013 | 42.76 | 16.18 | 29 | 0 | 29 | 0 | 0 | 0 |
| 6/27/2013 | 42.48 | 17.16 | 0 | 0 | 0 | 0 | 0 | 0 |
| 6/28/2013 | 40.21 | 19.66 | 131 | 0 | 104 | 1 | 1 | 401 |
| 6/29/2013 | 37.94 | 20.82 | 87 | 0 | 72 | 5 | 21 | 39 |

| Date | Discharge (cms) | Temp (C°) | Chinook | Coho | Steelhead | Cutthroat | Lamprey | Sucker |
|---|---|---|---|---|---|---|---|---|
| 6/30/2013 | 37.10 | 21.91 | 98 | 0 | 72 | 8 | 44 | 9 |
| 7/1/2013 | 36.25 | 23.41 | 73 | 0 | 62 | 12 | 169 | 7 |
| 7/2/2013 | 35.40 | 23.99 | 55 | 0 | 36 | 17 | 249 | 5 |
| 7/3/2013 | 34.26 | 24.37 | 22 | 0 | 21 | 14 | 111 | 1 |
| 7/4/2013 | 33.98 | 23.84 | 5 | 0 | 3 | 3 | 66 | 0 |
| 7/5/2013 | 32.56 | 22.72 | 3 | 0 | 0 | 2 | 45 | 0 |
| 7/6/2013 | 31.43 | 22.00 | 1 | 0 | 2 | 1 | 13 | 0 |
| 7/7/2013 | 31.71 | 21.77 | 1 | 0 | 0 | 0 | 10 | 0 |
| 7/8/2013 | 31.71 | 22.40 | 3 | 0 | 1 | 1 | 2 | 9 |
| 7/9/2013 | 31.15 | 22.30 | 1 | 0 | 0 | 2 | 3 | 11 |
| 7/10/2013 | 30.58 | 22.99 | 5 | 0 | 1 | 0 | 19 | 0 |
| 7/11/2013 | 30.02 | 22.95 | 2 | 0 | 2 | 1 | 42 | 1 |
| 7/12/2013 | 29.73 | 22.22 | 1 | 0 | 0 | 0 | 12 | 3 |
| 7/13/2013 | 29.73 | 21.58 | 0 | 0 | 0 | 1 | 3 | 3 |
| 7/14/2013 | 29.45 | 21.43 | 1 | 0 | 0 | 0 | 1 | 1 |
| 7/15/2013 | 29.45 | 22.35 | 2 | 0 | 1 | 0 | 1 | 6 |
| 7/16/2013 | 28.88 | 21.96 | 14 | 0 | 1 | 0 | 6 | 2 |
| 7/17/2013 | 28.32 | 21.37 | 18 | 0 | 1 | 0 | 7 | 1 |
| 7/18/2013 | 28.03 | 21.74 | 15 | 0 | 1 | 0 | 0 | 2 |
| 7/19/2013 | 27.52 | 22.29 | 12 | 0 | 4 | 1 | 2 | 5 |
| 7/20/2013 | 27.10 | 22.58 | 9 | 0 | 2 | 0 | 10 | 2 |
| 7/21/2013 | 26.82 | 22.78 | 12 | 0 | 3 | 1 | 16 | 0 |
| 7/22/2013 | 26.42 | 23.17 | 6 | 0 | 2 | 1 | 6 | 2 |
| 7/23/2013 | 26.11 | 23.13 | 9 | 0 | 2 | 0 | 1 | 2 |
| 7/24/2013 | 26.19 | 23.55 | 3 | 0 | 2 | 2 | 1 | 3 |
| 7/25/2013 | 26.31 | 23.90 | 7 | 0 | 0 | 1 | 3 | 7 |
| 7/26/2013 | 26.16 | 23.72 | 0 | 0 | 0 | 0 | 0 | 3 |
| 7/27/2013 | 25.85 | 23.17 | 0 | 0 | 0 | 0 | 1 | 0 |
| 7/28/2013 | 25.88 | 22.53 | 1 | 0 | 0 | 0 | 0 | 1 |
| 7/29/2013 | 26.08 | 21.63 | 0 | 0 | 1 | 0 | 0 | 2 |
| 7/30/2013 | 25.94 | 21.34 | 0 | 0 | 0 | 0 | 0 | 1 |
| 7/31/2013 | 25.66 | 21.12 | 0 | 0 | 3 | 0 | 1 | 0 |
| 8/1/2013 | 25.49 | 19.95 | 1 | 0 | 1 | 0 | 0 | 0 |
| 8/2/2013 | 25.66 | 19.39 | 0 | 0 | 3 | 0 | 0 | 0 |
| 8/3/2013 | 26.02 | 20.20 | 4 | 0 | 0 | 0 | 0 | 3 |
| 8/4/2013 | 25.51 | 20.56 | 14 | 0 | 7 | 0 | 1 | 1 |
| 8/5/2013 | 25.20 | 21.50 | 43 | 0 | 21 | 0 | 2 | 0 |
| 8/6/2013 | 24.86 | 22.23 | 76 | 0 | 29 | 0 | 6 | 1 |
| 8/7/2013 | 24.10 | 21.57 | 56 | 0 | 45 | 1 | 20 | 0 |
| 8/8/2013 | 23.70 | 19.30 | 25 | 0 | 13 | 0 | 0 | 0 |
| 8/9/2013 | 24.24 | 20.45 | 28 | 0 | 35 | 0 | 1 | 0 |
| 8/10/2013 | 24.95 | 19.87 | 34 | 0 | 27 | 0 | 0 | 2 |
| 8/11/2013 | 26.56 | 19.83 | 32 | 0 | 22 | 0 | 0 | 0 |
| 8/12/2013 | 27.10 | 19.65 | 17 | 0 | 29 | 0 | 1 | 0 |
| 8/13/2013 | 25.34 | 19.38 | 47 | 0 | 19 | 1 | 0 | 0 |

| Date | Discharge (cms) | Temp (C°) | Chinook | Coho | Steelhead | Cutthroat | Lamprey | Sucker |
|---|---|---|---|---|---|---|---|---|
| 8/14/2013 | 24.86 | 20.29 | 38 | 0 | 39 | 0 | 3 | 0 |
| 8/15/2013 | 24.66 | 20.71 | 42 | 0 | 31 | 0 | 18 | 0 |
| 8/16/2013 | 24.52 | 21.07 | 61 | 0 | 30 | 0 | 11 | 1 |
| 8/17/2013 | 23.98 | 21.20 | 45 | 0 | 26 | 0 | 5 | 0 |
| 8/18/2013 | 23.53 | 21.20 | 42 | 0 | 34 | 2 | 0 | 0 |
| 8/19/2013 | 23.28 | 21.73 | 36 | 0 | 20 | 1 | 3 | 0 |
| 8/20/2013 | 22.80 | 22.07 | 35 | 0 | 15 | 0 | 5 | 0 |
| 8/21/2013 | 22.80 | 21.56 | 16 | 0 | 12 | 2 | 7 | 0 |
| 8/22/2013 | 22.82 | 20.84 | 14 | 0 | 6 | 2 | 0 | 0 |
| 8/23/2013 | 24.52 | 19.90 | 4 | 0 | 4 | 0 | 0 | 0 |
| 8/24/2013 | 29.17 | 19.65 | 7 | 0 | 5 | 0 | 0 | 0 |
| 8/25/2013 | 26.33 | 19.14 | 6 | 0 | 2 | 0 | 0 | 1 |
| 8/26/2013 | 27.24 | 19.27 | 7 | 0 | 3 | 0 | 0 | 0 |
| 8/27/2013 | 27.95 | 19.68 | 36 | 0 | 4 | 0 | 0 | 0 |
| 8/28/2013 | 25.77 | 20.03 | 58 | 0 | 13 | 0 | 0 | 1 |
| 8/29/2013 | 25.37 | 20.02 | 81 | 0 | 16 | 0 | 0 | 0 |
| 8/30/2013 | 24.27 | 20.06 | 54 | 1 | 15 | 2 | 1 | 0 |
| 8/31/2013 | 23.98 | 20.55 | 31 | 0 | 18 | 0 | 0 | 0 |
| 9/1/2013 | 23.90 | 20.82 | 46 | 0 | 12 | 1 | 1 | 0 |
| 9/2/2013 | 23.90 | 20.57 | 62 | 0 | 19 | 0 | 0 | 0 |
| 9/3/2013 | 26.90 | 20.36 | 12 | 0 | 5 | 0 | 1 | 0 |
| 9/4/2013 | 26.14 | | 0 | 0 | 0 | 0 | 0 | 0 |
| 9/5/2013 | 24.47 | | 0 | 0 | 0 | 0 | 0 | 0 |
| 9/6/2013 | 25.32 | | 0 | 0 | 0 | 0 | 0 | 0 |
| 9/7/2013 | 26.45 | | 0 | 0 | 0 | 0 | 0 | 0 |
| 9/8/2013 | 25.51 | | 0 | 0 | 0 | 0 | 0 | 0 |
| 9/9/2013 | 23.93 | | 0 | 0 | 0 | 0 | 0 | 0 |
| 9/10/2013 | 24.72 | | 0 | 0 | 0 | 0 | 0 | 0 |
| 9/11/2013 | 24.38 | | 0 | 0 | 0 | 0 | 0 | 0 |
| 9/12/2013 | 24.15 | | 0 | 0 | 0 | 0 | 0 | 0 |
| 9/13/2013 | 24.13 | | 0 | 0 | 0 | 0 | 0 | 0 |
| 9/14/2013 | 24.13 | | 0 | 0 | 0 | 0 | 0 | 0 |
| 9/15/2013 | 24.13 | | 0 | 0 | 0 | 0 | 0 | 0 |
| 9/16/2013 | 24.13 | | 0 | 0 | 0 | 0 | 0 | 0 |
| 9/17/2013 | 24.24 | | 0 | 0 | 0 | 0 | 0 | 0 |
| 9/18/2013 | 23.11 | | 0 | 0 | 0 | 0 | 0 | 0 |
| 9/19/2013 | 23.42 | 17.97 | 9 | 3 | 6 | 1 | 0 | 0 |
| 9/20/2013 | 25.32 | 17.00 | 12 | 5 | 7 | 0 | 0 | 0 |
| 9/21/2013 | 26.56 | 16.20 | 16 | 4 | 4 | 1 | 0 | 0 |
| 9/22/2013 | 26.62 | 15.61 | 7 | 5 | 2 | 0 | 0 | 0 |
| 9/23/2013 | 27.13 | 15.24 | 3 | 11 | 1 | 0 | 0 | 0 |
| 9/24/2013 | 30.30 | 15.03 | 3 | 12 | 7 | 0 | 0 | 0 |
| 9/25/2013 | 37.94 | 14.79 | 17 | 26 | 3 | 0 | 0 | 0 |
| 9/26/2013 | 46.44 | 13.98 | 36 | 26 | 12 | 0 | 0 | 0 |
| 9/27/2013 | 35.96 | 13.66 | 18 | 39 | 9 | 1 | 0 | 0 |

| Date | Discharge (cms) | Temp (C°) | Chinook | Coho | Steelhead | Cutthroat | Lamprey | Sucker |
|---|---|---|---|---|---|---|---|---|
| 9/28/2013 | 32.85 | 13.54 | 8 | 20 | 1 | 1 | 0 | 0 |
| 9/29/2013 | 65.70 | 13.13 | 16 | 32 | 4 | 0 | 0 | 0 |
| 9/30/2013 | 225.40 | 12.24 | 14 | 9 | 1 | 0 | 0 | 0 |
| 10/1/2013 | 120.63 | 12.03 | 84 | 138 | 10 | 1 | 0 | 4 |
| 10/2/2013 | 72.77 | 11.30 | 46 | 93 | 14 | 3 | 0 | 0 |
| 10/3/2013 | 60.03 | 11.21 | 18 | 61 | 4 | 1 | 0 | 0 |
| 10/4/2013 | 57.48 | 11.11 | 10 | 30 | 6 | 3 | 0 | 0 |
| 10/5/2013 | 49.84 | 11.29 | 10 | 21 | 2 | 1 | 0 | 0 |
| 10/6/2013 | 44.17 | 11.41 | 10 | 40 | 4 | 3 | 0 | 0 |
| 10/7/2013 | 39.64 | 11.37 | 14 | 49 | 2 | 10 | 0 | 0 |
| 10/8/2013 | 37.66 | 10.75 | 10 | 18 | 1 | 3 | 0 | 0 |
| 10/9/2013 | 37.10 | 10.34 | 3 | 12 | 1 | 4 | 0 | 0 |
| 10/10/2013 | 39.36 | 10.92 | 1 | 13 | 3 | 3 | 0 | 0 |
| 10/11/2013 | 35.96 | 11.04 | 7 | 14 | 6 | 1 | 0 | 0 |
| 10/12/2013 | 33.98 | 10.88 | 3 | 10 | 5 | 1 | 0 | 0 |
| 10/13/2013 | 32.56 | 10.77 | 1 | 6 | 3 | 1 | 0 | 0 |
| 10/14/2013 | 32.00 | 10.42 | 1 | 8 | 1 | 0 | 0 | 0 |
| 10/15/2013 | 31.71 | 10.01 | 1 | 9 | 2 | 1 | 0 | 0 |
| 10/16/2013 | 31.15 | 9.80 | 0 | 5 | 3 | 0 | 0 | 0 |
| 10/17/2013 | 30.58 | 9.75 | 3 | 3 | 2 | 0 | 0 | 0 |
| 10/18/2013 | 30.30 | 10.00 | 3 | 3 | 2 | 0 | 0 | 0 |
| 10/19/2013 | 29.73 | 10.09 | 3 | 7 | 1 | 2 | 0 | 0 |
| 10/20/2013 | 29.45 | 10.06 | 4 | 6 | 5 | 1 | 0 | 0 |
| 10/21/2013 | 29.17 | 10.19 | 5 | 5 | 4 | 0 | 0 | 0 |
| 10/22/2013 | 29.17 | 10.46 | 3 | 7 | 7 | 0 | 0 | 0 |
| 10/23/2013 | 29.17 | 10.20 | 3 | 12 | 1 | 2 | 0 | 0 |
| 10/24/2013 | 29.17 | 10.15 | 2 | 2 | 5 | 0 | 0 | 0 |
| 10/25/2013 | 29.17 | 10.10 | 3 | 20 | 6 | 1 | 0 | 0 |
| 10/26/2013 | 29.45 | 10.18 | 4 | 13 | 6 | 0 | 0 | 0 |
| 10/27/2013 | 28.88 | 10.08 | 1 | 4 | 0 | 1 | 0 | 0 |
| 10/28/2013 | 29.17 | 9.79 | 2 | 4 | 1 | 0 | 0 | 0 |
| 10/29/2013 | 30.30 | 9.39 | 1 | 4 | 0 | 0 | 0 | 0 |
| 10/30/2013 | 29.17 | 9.02 | 0 | 1 | 1 | 1 | 0 | 0 |
| 10/31/2013 | 28.60 | 9.12 | 2 | 5 | 1 | 1 | 0 | 0 |
| 11/1/2013 | 28.01 | 9.40 | 0 | 1 | 0 | 1 | 0 | 0 |
| 11/2/2013 | 28.60 | 9.33 | 0 | 2 | 0 | 0 | 0 | 0 |
| 11/3/2013 | 36.53 | 9.17 | 1 | 19 | 1 | 0 | 0 | 0 |
| 11/4/2013 | 42.76 | 9.04 | 2 | 34 | 3 | 1 | 0 | 0 |
| 11/5/2013 | 39.64 | 9.01 | 1 | 73 | 6 | 1 | 0 | 0 |
| 11/6/2013 | 60.88 | 9.21 | 7 | 103 | 12 | 1 | 0 | 0 |
| 11/7/2013 | 65.98 | 9.50 | 3 | 542 | 16 | 1 | 0 | 0 |
| 11/8/2013 | 86.93 | 9.34 | 4 | 236 | 16 | 4 | 0 | 0 |
| 11/9/2013 | 69.66 | 9.35 | 1 | 270 | 11 | 1 | 0 | 0 |
| 11/10/2013 | 52.95 | 9.44 | 1 | 148 | 4 | 0 | 0 | 0 |
| 11/11/2013 | 45.02 | 9.44 | 1 | 81 | 4 | 0 | 0 | 0 |

| Date | Discharge (cms) | Temp (C°) | Chinook | Coho | Steelhead | Cutthroat | Lamprey | Sucker |
|---|---|---|---|---|---|---|---|---|
| 11/12/2013 | 40.78 | 8.67 | 1 | 8 | 6 | 0 | 0 | 0 |
| 11/13/2013 | 38.51 | 9.21 | 1 | 15 | 7 | 0 | 0 | 0 |
| 11/14/2013 | 36.53 | 9.46 | 0 | 19 | 8 | 0 | 0 | 0 |
| 11/15/2013 | 34.55 | 9.28 | 0 | 11 | 4 | 1 | 0 | 0 |
| 11/16/2013 | 35.40 | 8.91 | 1 | 10 | 4 | 0 | 0 | 0 |
| 11/17/2013 | 45.31 | 8.57 | 1 | 3 | 5 | 0 | 0 | 0 |
| 11/18/2013 | 46.72 | 8.07 | 0 | 3 | 1 | 0 | 0 | 0 |
| 11/19/2013 | 94.01 | 8.06 | 0 | 5 | 8 | 0 | 0 | 0 |
| 11/20/2013 | 387.94 | 9.08 | 0 | 92 | 1 | 0 | 0 | 0 |
| 11/21/2013 | 180.10 | 8.12 | 0 | 106 | 4 | 0 | 0 | 0 |
| 11/22/2013 | 108.74 | 6.14 | 0 | 29 | 3 | 0 | 0 | 0 |
| 11/23/2013 | 81.27 | 5.17 | 0 | 5 | 1 | 0 | 0 | 0 |
| 11/24/2013 | 66.83 | 4.69 | 0 | 0 | 0 | 0 | 0 | 0 |
| 11/25/2013 | 57.48 | 4.49 | 0 | 0 | 0 | 0 | 0 | 0 |
| 11/26/2013 | 51.25 | 4.47 | 0 | 0 | 0 | 0 | 0 | 0 |
| 11/27/2013 | 46.72 | 4.60 | 0 | 1 | 0 | 0 | 0 | 0 |
| 11/28/2013 | 43.61 | 4.93 | 0 | 1 | 0 | 0 | 0 | 0 |
| 11/29/2013 | 40.78 | 5.17 | 0 | 4 | 1 | 0 | 0 | 0 |
| 11/30/2013 | 39.08 | 5.35 | 0 | 8 | 4 | 0 | 0 | 0 |
| 12/1/2013 | 37.66 | 5.93 | 0 | 12 | 1 | 0 | 0 | 0 |
| 12/2/2013 | 41.91 | 6.92 | 0 | 20 | 10 | 0 | 0 | 0 |
| 12/3/2013 | 119.78 | 7.10 | 0 | 108 | 17 | 0 | 0 | 0 |
| 12/4/2013 | 81.84 | 6.17 | 0 | 7 | 1 | 0 | 0 | 0 |
| 12/5/2013 | 64.56 | 3.75 | 0 | 1 | 0 | 0 | 0 | 0 |
| 12/6/2013 | 55.22 | 2.31 | 0 | 0 | 0 | 0 | 0 | 0 |
| 12/7/2013 | 43.32 | 1.42 | 0 | 0 | 0 | 0 | 0 | 0 |
| 12/8/2013 | 30.30 | 0.53 | 0 | 0 | 0 | 0 | 0 | 0 |
| 12/9/2013 | 30.87 | 0.28 | 0 | 0 | 0 | 0 | 0 | 0 |
| 12/10/2013 | 34.55 | 0.24 | 0 | 0 | 0 | 0 | 0 | 0 |
| 12/11/2013 | 39.93 | 0.24 | 0 | 0 | 0 | 0 | 0 | 0 |
| 12/12/2013 | 38.79 | 0.36 | 0 | 0 | 0 | 0 | 0 | 0 |
| 12/13/2013 | 37.10 | 1.27 | 0 | 0 | 0 | 0 | 0 | 0 |
| 12/14/2013 | 35.40 | 2.73 | 0 | 0 | 0 | 0 | 0 | 0 |
| 12/15/2013 | 37.38 | 3.35 | 0 | 0 | 1 | 0 | 0 | 0 |
| 12/16/2013 | 38.79 | 3.48 | 0 | 0 | 1 | 0 | 0 | 0 |
| 12/17/2013 | 42.19 | 3.51 | 0 | 0 | 0 | 0 | 0 | 0 |
| 12/18/2013 | 42.76 | 3.50 | 0 | 0 | 0 | 0 | 0 | 0 |
| 12/19/2013 | 42.76 | 3.49 | 0 | 0 | 0 | 0 | 0 | 0 |
| 12/20/2013 | 41.34 | 3.60 | 0 | 0 | 0 | 0 | 0 | 0 |
| 12/21/2013 | 46.44 | 4.51 | 0 | 2 | 1 | 0 | 0 | 0 |
| 12/22/2013 | 67.68 | 5.37 | 1 | 16 | 4 | 0 | 0 | 0 |
| 12/23/2013 | 64.56 | 5.53 | 0 | 20 | 1 | 0 | 0 | 0 |
| 12/24/2013 | 62.58 | 6.11 | 0 | 25 | 21 | 0 | 0 | 0 |
| 12/25/2013 | 62.30 | 6.32 | 0 | 12 | 9 | 0 | 0 | 0 |
| 12/26/2013 | 56.63 | 5.42 | 0 | 3 | 0 | 0 | 0 | 0 |

| Date | Discharge (cms) | Temp (C°) | Chinook | Coho | Steelhead | Cutthroat | Lamprey | Sucker |
|---|---|---|---|---|---|---|---|---|
| 12/27/2013 | 51.82 | 4.59 | 0 | 1 | 0 | 0 | 0 | 0 |
| 12/28/2013 | 48.14 | 4.21 | 0 | 0 | 0 | 0 | 0 | 0 |
| 12/29/2013 | 44.17 | 4.53 | 0 | 1 | 3 | 0 | 0 | 0 |
| 12/30/2013 | 41.91 | 4.57 | 0 | 0 | 0 | 0 | 0 | 0 |
| 12/31/2013 | 40.21 | 4.18 | 0 | 0 | 0 | 0 | 0 | 0 |
| 1/1/2014 | 38.79 | 4.22 | 0 | 0 | 0 | 0 | 0 | 0 |
| 1/2/2014 | 37.38 | 4.38 | 0 | 0 | 1 | 0 | 0 | 0 |
| 1/3/2014 | 36.53 | 4.74 | 0 | 0 | 1 | 0 | 0 | 0 |
| 1/4/2014 | 35.68 | 4.70 | 0 | 0 | 0 | 0 | 0 | 0 |
| 1/5/2014 | 35.11 | 4.85 | 0 | 0 | 1 | 0 | 0 | 0 |
| 1/6/2014 | 34.55 | 3.79 | 0 | 0 | 0 | 0 | 0 | 0 |
| 1/7/2014 | 33.70 | 3.76 | 0 | 0 | 0 | 0 | 0 | 0 |
| 1/8/2014 | 35.40 | 4.76 | 0 | 0 | 0 | 0 | 0 | 0 |
| 1/9/2014 | 55.78 | 6.04 | 0 | 5 | 28 | 0 | 0 | 0 |
| 1/10/2014 | 93.73 | 6.71 | 0 | 45 | 99 | 0 | 0 | 0 |
| 1/11/2014 | 99.11 | 7.35 | 0 | 21 | 156 | 0 | 0 | 0 |
| 1/12/2014 | 211.24 | 7.32 | 0 | 1 | 55 | 1 | 0 | 0 |
| 1/13/2014 | 201.90 | 7.84 | 0 | 1 | 89 | 0 | 0 | 0 |
| 1/14/2014 | 163.67 | 7.39 | 0 | 4 | 88 | 0 | 0 | 0 |
| 1/15/2014 | 122.33 | 6.49 | 0 | 0 | 54 | 0 | 0 | 0 |
| 1/16/2014 | 99.11 | 5.99 | 0 | 1 | 36 | 0 | 0 | 0 |
| 1/17/2014 | 85.52 | 5.42 | 0 | 0 | 6 | 0 | 0 | 0 |
| 1/18/2014 | 75.61 | 5.17 | 0 | 0 | 7 | 0 | 0 | 0 |
| 1/19/2014 | 68.24 | 5.14 | 0 | 0 | 7 | 0 | 0 | 0 |
| 1/20/2014 | 62.30 | 5.25 | 0 | 0 | 9 | 0 | 0 | 0 |
| 1/21/2014 | 57.48 | 5.16 | 0 | 0 | 3 | 0 | 0 | 0 |
| 1/22/2014 | 53.80 | 5.02 | 0 | 0 | 3 | 0 | 0 | 0 |
| 1/23/2014 | 50.69 | 5.45 | 0 | 0 | 3 | 0 | 0 | 0 |
| 1/24/2014 | 47.86 | 5.05 | 0 | 0 | 0 | 0 | 0 | 0 |
| 1/25/2014 | 45.31 | 4.62 | 0 | 0 | 0 | 0 | 0 | 0 |
| 1/26/2014 | 43.32 | 4.65 | 0 | 0 | 0 | 0 | 0 | 0 |
| 1/27/2014 | 41.63 | 4.68 | 0 | 0 | 0 | 0 | 0 | 0 |
| 1/28/2014 | 40.78 | 5.44 | 0 | 0 | 6 | 0 | 0 | 0 |
| 1/29/2014 | 58.62 | 6.95 | 0 | 0 | 129 | 1 | 0 | 0 |
| 1/30/2014 | 147.25 | 7.72 | 0 | 0 | 419 | 1 | 0 | 0 |
| 1/31/2014 | 113.27 | 7.38 | 0 | 0 | 241 | 1 | 0 | 0 |
| 2/1/2014 | 91.18 | 7.07 | 0 | 0 | 28 | 0 | 0 | 0 |
| 2/2/2014 | 77.87 | 6.50 | 0 | 0 | 17 | 0 | 0 | 0 |
| 2/3/2014 | 70.23 | 6.30 | 0 | 0 | 10 | 1 | 0 | 0 |
| 2/4/2014 | 65.13 | 6.30 | 0 | 0 | 14 | 0 | 0 | 0 |
| 2/5/2014 | 60.03 | 6.00 | 0 | 0 | 0 | 0 | 0 | 0 |
| 2/6/2014 | 57.20 | 5.28 | 0 | 0 | 0 | 0 | 0 | 0 |
| 2/7/2014 | 64.28 | 5.14 | 0 | 0 | 3 | 0 | 0 | 0 |
| 2/8/2014 | 150.65 | 5.88 | 0 | 0 | 115 | 0 | 0 | 0 |
| 2/9/2014 | 302.99 | 7.92 | 0 | 0 | 87 | 0 | 0 | 0 |

| Date | Discharge (cms) | Temp (C°) | Chinook | Coho | Steelhead | Cutthroat | Lamprey | Sucker |
|---|---|---|---|---|---|---|---|---|
| 2/10/2014 | 205.58 | 7.93 | 0 | 0 | 429 | 0 | 0 | 0 |
| 2/11/2014 | 156.59 | 7.95 | 0 | 0 | 453 | 0 | 0 | 0 |
| 2/12/2014 | 441.74 | 8.14 | 0 | 0 | 156 | 1 | 0 | 0 |
| 2/13/2014 | 787.21 | 8.67 | 0 | 0 | 0 | 0 | 0 | 0 |
| 2/14/2014 | 860.83 | 8.82 | 0 | 0 | 0 | 0 | 0 | 0 |
| 2/15/2014 | 959.94 | 8.60 | 0 | 0 | 0 | 0 | 0 | 0 |
| 2/16/2014 | 699.43 | 8.36 | 0 | 0 | 0 | 0 | 0 | 0 |
| 2/17/2014 | 433.25 | 7.85 | 0 | 0 | 7 | 0 | 0 | 0 |
| 2/18/2014 | 421.92 | 8.10 | 0 | 0 | 31 | 0 | 0 | 0 |
| 2/19/2014 | 546.52 | 7.88 | 0 | 0 | 4 | 0 | 0 | 0 |
| 2/20/2014 | 362.46 | 7.58 | 0 | 0 | 26 | 0 | 0 | 0 |
| 2/21/2014 | 269.86 | 8.11 | 0 | 0 | 175 | 1 | 0 | 0 |
| 2/22/2014 | 222.00 | 7.98 | 0 | 0 | 320 | 1 | 0 | 0 |
| 2/23/2014 | 186.32 | 7.08 | 0 | 0 | 73 | 0 | 0 | 0 |
| 2/24/2014 | 161.12 | 7.98 | 0 | 0 | 320 | 0 | 0 | 0 |
| 2/25/2014 | 145.27 | 8.89 | 0 | 0 | 653 | 2 | 0 | 0 |
| 2/26/2014 | 132.81 | 8.90 | 0 | 0 | 449 | 0 | 0 | 0 |
| 2/27/2014 | 123.18 | 9.16 | 0 | 0 | 313 | 0 | 0 | 3 |
| 2/28/2014 | 117.51 | 8.91 | 0 | 0 | 204 | 0 | 0 | 1 |
| 3/1/2014 | 111.00 | 8.90 | 0 | 0 | 98 | 0 | 0 | 0 |
| 3/2/2014 | 117.80 | 9.04 | 0 | 0 | 150 | 0 | 0 | 0 |
| 3/3/2014 | 180.94 | 9.30 | 0 | 0 | 197 | 0 | 0 | 0 |
| 3/4/2014 | 286.00 | 9.46 | 0 | 0 | 75 | 0 | 0 | 5 |
| 3/5/2014 | 211.53 | 9.56 | 0 | 0 | 282 | 0 | 0 | 4 |
| 3/6/2014 | 256.55 | 9.48 | 0 | 0 | 121 | 0 | 0 | 1 |
| 3/7/2014 | 385.11 | 9.16 | 0 | 0 | 22 | 0 | 0 | 4 |
| 3/8/2014 | 263.91 | 8.76 | 0 | 0 | 70 | 0 | 0 | 0 |
| 3/9/2014 | 331.31 | 9.35 | 0 | 0 | 128 | 0 | 0 | 2 |
| 3/10/2014 | 767.39 | 9.48 | 0 | 0 | 3 | 0 | 0 | 0 |
| 3/11/2014 | 543.68 | 8.32 | 0 | 0 | 0 | 0 | 0 | 0 |
| 3/12/2014 | 334.14 | 8.27 | 0 | 0 | 5 | 0 | 0 | 0 |
| 3/13/2014 | 242.96 | 8.61 | 0 | 0 | 77 | 0 | 0 | 0 |
| 3/14/2014 | 195.39 | 9.04 | 0 | 0 | 170 | 0 | 0 | 0 |
| 3/15/2014 | 165.37 | 9.38 | 0 | 0 | 164 | 0 | 0 | 0 |
| 3/16/2014 | 143.00 | 9.33 | 1 | 0 | 253 | 0 | 0 | 0 |
| 3/17/2014 | 140.17 | 9.50 | 0 | 0 | 114 | 0 | 0 | 1 |
| 3/18/2014 | 130.82 | 8.82 | 0 | 0 | 20 | 1 | 0 | 0 |
| 3/19/2014 | 118.65 | 8.54 | 1 | 0 | 17 | 0 | 0 | 0 |
| 3/20/2014 | 108.74 | 9.19 | 0 | 0 | 53 | 0 | 0 | 0 |
| 3/21/2014 | 99.96 | 8.83 | 0 | 0 | 25 | 0 | 0 | 0 |
| 3/22/2014 | 94.01 | 8.41 | 0 | 0 | 19 | 0 | 0 | 0 |
| 3/23/2014 | 87.78 | 8.62 | 0 | 0 | 31 | 0 | 0 | 0 |
| 3/24/2014 | 82.40 | 9.18 | 0 | 0 | 105 | 0 | 0 | 0 |
| 3/25/2014 | 79.00 | 9.37 | 0 | 0 | 319 | 1 | 0 | 1 |
| 3/26/2014 | 81.27 | 9.39 | 0 | 0 | 100 | 1 | 0 | 3 |

| Date | Discharge (cms) | Temp (C°) | Chinook | Coho | Steelhead | Cutthroat | Lamprey | Sucker |
|---|---|---|---|---|---|---|---|---|
| 3/27/2014 | 106.19 | 9.42 | 1 | 0 | 263 | 1 | 0 | 5 |
| 3/28/2014 | 202.75 | 8.62 | 0 | 0 | 143 | 0 | 0 | 0 |
| 3/29/2014 | 529.53 | 8.81 | 0 | 0 | 3 | 0 | 0 | 0 |
| 3/30/2014 | 365.29 | 8.72 | 0 | 0 | 21 | 0 | 0 | 0 |
| 3/31/2014 | 268.16 | 8.07 | 0 | 0 | 10 | 0 | 0 | 0 |
| 4/1/2014 | 210.68 | 8.40 | 0 | 0 | 32 | 0 | 0 | 0 |
| 4/2/2014 | 169.05 | 9.43 | 0 | 0 | 58 | 1 | 0 | 0 |
| 4/3/2014 | 143.85 | 9.27 | 0 | 0 | 131 | 0 | 0 | 0 |
| 4/4/2014 | 126.58 | 9.73 | 1 | 0 | 172 | 0 | 0 | 0 |
| 4/5/2014 | 120.91 | 9.63 | 0 | 0 | 125 | 0 | 0 | 0 |
| 4/6/2014 | 134.51 | 10.20 | 6 | 0 | 91 | 0 | 0 | 20 |
| 4/7/2014 | 151.21 | 11.12 | 1 | 0 | 117 | 0 | 0 | 240 |
| 4/8/2014 | 138.47 | 11.52 | 5 | 0 | 97 | 1 | 0 | 207 |
| 4/9/2014 | 126.58 | 11.44 | 9 | 0 | 99 | 0 | 0 | 219 |
| 4/10/2014 | 115.53 | 11.64 | 0 | 0 | 23 | 1 | 0 | 4 |
| 4/11/2014 | 106.19 | 12.23 | 4 | 0 | 50 | 1 | 0 | 3 |
| 4/12/2014 | 98.83 | 11.41 | 11 | 0 | 70 | 0 | 0 | 14 |
| 4/13/2014 | 91.75 | 12.15 | 7 | 0 | 27 | 0 | 0 | 35 |
| 4/14/2014 | 84.95 | 12.22 | 31 | 0 | 42 | 0 | 0 | 28 |
| 4/15/2014 | 80.42 | 12.68 | 8 | 0 | 58 | 0 | 0 | 64 |
| 4/16/2014 | 76.46 | 13.15 | 26 | 0 | 35 | 3 | 0 | 55 |
| 4/17/2014 | 73.62 | 11.89 | 21 | 0 | 16 | 1 | 0 | 29 |
| 4/18/2014 | 78.15 | 12.14 | 39 | 0 | 28 | 0 | 0 | 12 |
| 4/19/2014 | 73.34 | 11.58 | 54 | 0 | 23 | 0 | 0 | 4 |
| 4/20/2014 | 69.09 | 11.65 | 11 | 0 | 7 | 0 | 0 | 0 |
| 4/21/2014 | 66.54 | 12.22 | 51 | 0 | 30 | 1 | 0 | 244 |
| 4/22/2014 | 69.09 | 12.20 | 95 | 0 | 12 | 0 | 0 | 72 |
| 4/23/2014 | 76.74 | 11.34 | 27 | 0 | 3 | 0 | 0 | 23 |
| 4/24/2014 | 145.55 | 10.28 | 28 | 0 | 3 | 0 | 0 | 4 |
| 4/25/2014 | 244.37 | 9.67 | 1 | 0 | 4 | 0 | 0 | 7 |
| 4/26/2014 | 181.23 | 9.43 | 0 | 0 | 1 | 0 | 0 | 6 |
| 4/27/2014 | 154.61 | 9.45 | 2 | 0 | 6 | 0 | 0 | 1 |
| 4/28/2014 | 171.32 | 9.88 | 9 | 0 | 5 | 0 | 0 | 0 |
| 4/29/2014 | 157.16 | 11.62 | 23 | 0 | 7 | 0 | 0 | 5 |
| 4/30/2014 | 133.66 | 13.50 | 40 | 0 | 20 | 0 | 0 | 778 |
| 5/1/2014 | 117.51 | 14.24 | 96 | 0 | 18 | 2 | 0 | 793 |
| 5/2/2014 | 105.62 | 14.62 | 108 | 0 | 10 | 0 | 0 | 394 |
| 5/3/2014 | 94.86 | 14.05 | 196 | 0 | 9 | 2 | 0 | 108 |
| 5/4/2014 | 92.31 | 13.79 | 122 | 0 | 2 | 0 | 0 | 53 |
| 5/5/2014 | 99.11 | 13.04 | 42 | 0 | 4 | 0 | 0 | 18 |
| 5/6/2014 | 102.79 | 12.84 | 37 | 0 | 5 | 0 | 0 | 7 |
| 5/7/2014 | 92.88 | 12.82 | 35 | 0 | 2 | 0 | 0 | 2 |
| 5/8/2014 | 86.65 | 12.12 | 109 | 0 | 11 | 0 | 0 | 19 |
| 5/9/2014 | 151.21 | 11.68 | 109 | 0 | 2 | 0 | 0 | 0 |
| 5/10/2014 | 276.66 | 10.02 | 11 | 0 | 0 | 0 | 0 | 0 |

| Date | Discharge (cms) | Temp (C°) | Chinook | Coho | Steelhead | Cutthroat | Lamprey | Sucker |
|---|---|---|---|---|---|---|---|---|
| 5/11/2014 | 275.24 | 10.31 | 1 | 0 | 0 | 0 | 0 | 2 |
| 5/12/2014 | 202.75 | 11.27 | 0 | 0 | 0 | 0 | 0 | 0 |
| 5/13/2014 | 161.69 | 14.01 | 41 | 0 | 5 | 0 | 0 | 89 |
| 5/14/2014 | 132.81 | 15.04 | 186 | 0 | 8 | 1 | 0 | 342 |
| 5/15/2014 | 116.67 | 15.39 | 255 | 0 | 14 | 3 | 0 | 262 |
| 5/16/2014 | 105.34 | 15.58 | 122 | 0 | 11 | 1 | 0 | 282 |
| 5/17/2014 | 96.28 | 16.02 | 276 | 0 | 13 | 1 | 0 | 423 |
| 5/18/2014 | 93.73 | 15.19 | 227 | 0 | 4 | 1 | 1 | 156 |
| 5/19/2014 | 103.36 | 14.56 | 54 | 0 | 0 | 0 | 0 | 15 |
| 5/20/2014 | 96.84 | 14.48 | 48 | 0 | 2 | 1 | 0 | 10 |
| 5/21/2014 | 88.35 | 14.90 | 143 | 0 | 10 | 1 | 0 | 126 |
| 5/22/2014 | 82.40 | 16.25 | 216 | 0 | 15 | 0 | 0 | 804 |
| 5/23/2014 | 78.15 | 16.21 | 228 | 0 | 17 | 0 | 0 | 693 |
| 5/24/2014 | 74.47 | 16.02 | 132 | 0 | 11 | 2 | 2 | 168 |
| 5/25/2014 | 70.51 | 16.93 | 138 | 0 | 5 | 1 | 0 | 390 |
| 5/26/2014 | 67.11 | 17.23 | 164 | 0 | 9 | 5 | 0 | 340 |
| 5/27/2014 | 65.13 | 16.63 | 199 | 0 | 18 | 5 | 0 | 448 |
| 5/28/2014 | 63.71 | 15.78 | 40 | 0 | 4 | 1 | 0 | 27 |
| 5/29/2014 | 64.00 | 15.30 | 13 | 0 | 9 | 0 | 0 | 0 |
| 5/30/2014 | 60.60 | 15.88 | 70 | 0 | 16 | 1 | 0 | 186 |
| 5/31/2014 | 56.92 | 15.99 | 99 | 0 | 16 | 0 | 0 | 367 |
| 6/1/2014 | 55.78 | 16.17 | 105 | 0 | 15 | 0 | 0 | 500 |
| 6/2/2014 | 54.09 | 16.50 | 31 | 0 | 13 | 0 | 0 | 53 |
| 6/3/2014 | 52.67 | 17.56 | 79 | 0 | 4 | 0 | 0 | 837 |
| 6/4/2014 | 52.39 | 17.13 | 23 | 0 | 1 | 0 | 2 | 47 |
| 6/5/2014 | 50.97 | 18.28 | 105 | 0 | 17 | 1 | 2 | 3330 |
| 6/6/2014 | 49.55 | 18.35 | 84 | 0 | 13 | 1 | 0 | 1444 |
| 6/7/2014 | 48.14 | 18.69 | 98 | 0 | 12 | 8 | 1 | 2101 |
| 6/8/2014 | 47.29 | 19.10 | 89 | 0 | 12 | 7 | 3 | 2773 |
| 6/9/2014 | 46.44 | 19.52 | 88 | 0 | 14 | 6 | 1 | 1404 |
| 6/10/2014 | 45.31 | 19.23 | 65 | 0 | 14 | 4 | 0 | 822 |
| 6/11/2014 | 43.32 | 19.27 | 39 | 0 | 17 | 0 | 2 | 822 |
| 6/12/2014 | 41.63 | 18.91 | 48 | 0 | 12 | 0 | 0 | 835 |
| 6/13/2014 | 42.48 | 17.47 | 27 | 0 | 12 | 0 | 0 | 48 |
| 6/14/2014 | 44.17 | 16.88 | 14 | 0 | 7 | 0 | 0 | 62 |
| 6/15/2014 | 43.32 | 16.80 | 10 | 0 | 4 | 0 | 0 | 255 |
| 6/16/2014 | 41.91 | 16.19 | 29 | 0 | 18 | 0 | 16 | 7 |
| 6/17/2014 | 42.19 | 15.58 | 45 | 0 | 23 | 1 | 0 | 2 |
| 6/18/2014 | 43.89 | 16.18 | 37 | 0 | 11 | 0 | 0 | 36 |
| 6/19/2014 | 41.06 | 17.63 | 66 | 0 | 44 | 0 | 2 | 1365 |
| 6/20/2014 | 39.93 | 18.41 | 74 | 0 | 54 | 0 | 37 | 876 |
| 6/21/2014 | 38.51 | 18.46 | 53 | 0 | 45 | 1 | 0 | 39 |
| 6/22/2014 | 37.66 | 18.97 | 38 | 0 | 54 | 3 | 1 | 74 |
| 6/23/2014 | 37.38 | 19.51 | 41 | 0 | 37 | 0 | 0 | 39 |
| 6/24/2014 | 37.10 | 19.51 | 20 | 0 | 26 | 0 | 12 | 14 |

| Date | Discharge (cms) | Temp (C°) | Chinook | Coho | Steelhead | Cutthroat | Lamprey | Sucker |
|---|---|---|---|---|---|---|---|---|
| 6/25/2014 | 37.38 | 19.60 | 61 | 0 | 77 | 1 | 12 | 6 |
| 6/26/2014 | 44.17 | 18.67 | 55 | 0 | 60 | 1 | 5 | 119 |
| 6/27/2014 | 47.86 | 18.49 | 30 | 0 | 48 | 2 | 5 | 207 |
| 6/28/2014 | 44.17 | 18.62 | 35 | 0 | 49 | 0 | 2 | 55 |
| 6/29/2014 | 41.91 | 18.66 | 25 | 0 | 53 | 2 | 0 | 3 |
| 6/30/2014 | 39.08 | 19.91 | 18 | 0 | 49 | 3 | 1 | 24 |
| 7/1/2014 | 37.66 | 21.14 | 22 | 0 | 39 | 2 | 13 | 10 |
| 7/2/2014 | 36.53 | 22.48 | 39 | 0 | 58 | 4 | 64 | 11 |
| 7/3/2014 | 35.40 | 21.81 | 31 | 0 | 53 | 2 | 115 | 2 |
| 7/4/2014 | 35.11 | 19.84 | 11 | 0 | 30 | 0 | 0 | 0 |
| 7/5/2014 | 35.40 | 21.06 | 14 | 0 | 31 | 0 | 5 | 1 |
| 7/6/2014 | 34.26 | 22.37 | 19 | 0 | 35 | 1 | 4 | 2 |
| 7/7/2014 | 33.70 | 23.25 | 19 | 0 | 31 | 2 | 20 | 4 |
| 7/8/2014 | 33.70 | 23.91 | 9 | 0 | 6 | 1 | 86 | 1 |
| 7/9/2014 | 33.70 | 23.75 | 2 | 0 | 15 | 1 | 51 | 1 |
| 7/10/2014 | 33.41 | 23.90 | 5 | 0 | 10 | 1 | 64 | 3 |
| 7/11/2014 | 32.85 | 23.38 | 3 | 0 | 4 | 1 | 21 | 0 |
| 7/12/2014 | 32.28 | 22.16 | 0 | 0 | 1 | 0 | 0 | 0 |
| 7/13/2014 | 32.56 | 22.70 | 1 | 0 | 4 | 0 | 10 | 2 |
| 7/14/2014 | 32.28 | 22.54 | 2 | 0 | 4 | 0 | 0 | 0 |
| 7/15/2014 | 32.00 | 23.32 | 1 | 0 | 5 | 0 | 7 | 1 |
| 7/16/2014 | 30.58 | 24.97 | 1 | 0 | 3 | 0 | 20 | 1 |
| 7/17/2014 | 30.30 | 22.97 | 4 | 0 | 2 | 0 | 16 | 0 |
| 7/18/2014 | 29.73 | 23.94 | 3 | 0 | 2 | 0 | 13 | 0 |
| 7/19/2014 | 29.17 | 24.15 | 2 | 0 | 2 | 0 | 0 | 1 |
| 7/20/2014 | 29.45 | 24.56 | 1 | 0 | 1 | 0 | 0 | 0 |
| 7/21/2014 | 29.45 | 23.30 | 1 | 0 | 0 | 0 | 1 | 1 |
| 7/22/2014 | 29.45 | 21.27 | 0 | 0 | 0 | 0 | 0 | 0 |
| 7/23/2014 | 30.30 | 20.75 | 0 | 0 | 0 | 0 | 0 | 0 |
| 7/24/2014 | 30.87 | 19.71 | 0 | 0 | 4 | 0 | 0 | 0 |
| 7/25/2014 | 31.71 | 20.24 | 0 | 0 | 0 | 0 | 0 | 0 |
| 7/26/2014 | 30.02 | 20.76 | 10 | 0 | 7 | 0 | 0 | 0 |
| 7/27/2014 | 29.17 | 21.85 | 20 | 0 | 19 | 0 | 0 | 0 |
| 7/28/2014 | 28.32 | 22.03 | 22 | 0 | 24 | 0 | 0 | 0 |
| 7/29/2014 | 28.12 | 23.75 | 51 | 0 | 68 | 0 | 4 | 0 |
| 7/30/2014 | 27.78 | 24.05 | 22 | 0 | 44 | 0 | 56 | 0 |
| 7/31/2014 | 27.67 | 24.53 | 12 | 0 | 29 | 0 | 49 | 0 |
| 8/1/2014 | 27.52 | 24.36 | 4 | 0 | 11 | 1 | 0 | 0 |
| 8/2/2014 | 27.64 | 24.77 | 1 | 0 | 8 | 0 | 9 | 0 |
| 8/3/2014 | 27.33 | 24.58 | 0 | 0 | 5 | 0 | 12 | 0 |
| 8/4/2014 | 27.10 | 23.73 | 0 | 0 | 2 | 0 | 3 | 0 |
| 8/5/2014 | 26.79 | 23.22 | 1 | 0 | 1 | 1 | 3 | 0 |
| 8/6/2014 | 27.47 | 23.09 | 0 | 0 | 1 | 0 | 0 | 0 |
| 8/7/2014 | 27.30 | 23.54 | 0 | 0 | 2 | 1 | 0 | 1 |
| 8/8/2014 | 26.48 | 23.47 | 0 | 0 | 0 | 0 | 0 | 0 |

| Date | Discharge (cms) | Temp (C°) | Chinook | Coho | Steelhead | Cutthroat | Lamprey | Sucker |
|---|---|---|---|---|---|---|---|---|
| 8/9/2014 | 26.11 | 23.12 | 0 | 0 | 0 | 0 | 0 | 0 |
| 8/10/2014 | 25.99 | 23.18 | 1 | 0 | 0 | 0 | 0 | 0 |
| 8/11/2014 | 26.16 | 23.64 | 1 | 0 | 0 | 0 | 0 | 0 |
| 8/12/2014 | 26.62 | 23.89 | 1 | 0 | 0 | 0 | 0 | 0 |
| 8/13/2014 | 27.58 | 22.28 | 1 | 0 | 1 | 0 | 0 | 0 |
| 8/14/2014 | 28.32 | 21.16 | 1 | 0 | 2 | 1 | 0 | 0 |
| 8/15/2014 | 27.69 | 21.35 | 0 | 0 | 1 | 0 | 0 | 0 |
| 8/16/2014 | 27.21 | 21.99 | 3 | 0 | 0 | 0 | 0 | 0 |
| 8/17/2014 | 26.79 | 23.02 | 7 | 0 | 5 | 0 | 0 | 0 |
| 8/18/2014 | 26.48 | 23.05 | 18 | 0 | 13 | 0 | 0 | 0 |
| 8/19/2014 | 26.19 | 23.27 | 20 | 0 | 16 | 0 | 0 | 0 |
| 8/20/2014 | 25.46 | 22.93 | 24 | 0 | 15 | 1 | 0 | 0 |
| 8/21/2014 | 25.32 | 22.27 | 4 | 0 | 6 | 0 | 0 | 0 |
| 8/22/2014 | 24.86 | 22.06 | 11 | 0 | 5 | 0 | 2 | 0 |
| 8/23/2014 | 25.00 | 21.79 | 4 | 0 | 1 | 0 | 0 | 0 |
